# A Genome-Wide Survey for Host Response of Silkworm, *Bombyx mori* during Pathogen *Bacillus bombyseptieus* Infection

**DOI:** 10.1371/journal.pone.0008098

**Published:** 2009-12-01

**Authors:** Lulin Huang, Tingcai Cheng, Pingzhen Xu, Daojun Cheng, Ting Fang, Qingyou Xia

**Affiliations:** 1 Institute of Sericulture and Systems Biology, Southwest University, Chongqing, China; 2 Institute of Agronomy and Life Science, Chongqing University, Chongqing, China; 3 Institute of Economic Crops Breeding and Cultivation, Sichuan Academy of Agricultural Sciences, Chengdu, China; Technical University Munich, Germany

## Abstract

Host-pathogen interactions are complex relationships, and a central challenge is to reveal the interactions between pathogens and their hosts. *Bacillus bombysepticus* (*Bb*) which can produces spores and parasporal crystals was firstly separated from the corpses of the infected silkworms (*Bombyx mori*). *Bb* naturally infects the silkworm can cause an acute fuliginosa septicaemia and kill the silkworm larvae generally within one day in the hot and humid season. *Bb* pathogen of the silkworm can be used for investigating the host responses after the infection. Gene expression profiling during four time-points of silkworm whole larvae after *Bb* infection was performed to gain insight into the mechanism of *Bb*-associated host whole body effect. Genome-wide survey of the host genes demonstrated many genes and pathways modulated after the infection. GO analysis of the induced genes indicated that their functions could be divided into 14 categories. KEGG pathway analysis identified that six types of basal metabolic pathway were regulated, including genetic information processing and transcription, carbohydrate metabolism, amino acid and nitrogen metabolism, nucleotide metabolism, metabolism of cofactors and vitamins, and xenobiotic biodegradation and metabolism. Similar to *Bacillus thuringiensis* (*Bt*), *Bb* can also induce a silkworm poisoning-related response. In this process, genes encoding midgut peritrophic membrane proteins, aminopeptidase N receptors and sodium/calcium exchange protein showed modulation. For the first time, we found that *Bb* induced a lot of genes involved in juvenile hormone synthesis and metabolism pathway upregulated. *Bb* also triggered the host immune responses, including cellular immune response and serine protease cascade melanization response. Real time PCR analysis showed that *Bb* can induce the silkworm systemic immune response, mainly by the Toll pathway. Anti-microorganism peptides (AMPs), including of Attacin, Lebocin, Enbocin, Gloverin and Moricin families, were upregulated at 24 hours post the infection.

## Introduction

A lot species of bacteria belonging to the genus *Bacillus* have established a systemic infection in a variety of hosts including humans, animals and insects [1]–[3]. Some species of *Bacillus* are investigated more than others for their closer connection with human beings. For example, *Bacillus thuringiensis*, as a good model, was well studied on its insecticidal mechanism by producing crystal proteins and has been developed as commercial biological products to control insect pests [4]–[6]. *Bacillus cereus* can produce food poisoning toxins generally causes summer food poisoning [7], [8]. Studies revealed that fur gene of *Bacillus cereus* regulates iron metabolism and is required for full virulence [9]. *Bacillus anthracis* infection results in the anthrax disease of human and animals [10], [11]. In the process of infection host macrophages, *B. anthracis* can rapidly adapt to the intracellular environment, and modulate its metabolic pathways such as energy metabolism and biosynthesis of cofactors for its intracellular growth [12]. For other pathogens, systematic gene expression such as basal metabolic pathways also can be modulated during its infection. For example, during the developmental cycle and iron depletion-mediated persistence of *Chlamydophila pneumoniae*, its transcriptome changes in many functional groups such as the cell envelope and the translation machinery [13].

On the host side, hosts' behaviours can be significantly dominated by pathogens infection. *B. anthracis* ames spores also significantly affect the expression of approximately 580 host genes in murine lung, spleen, and heart tissues at 8- and 48-h time points [14]. Recently, the genome-wide analysis of the interaction between the endosymbiotic bacterium *Wolbachia* and its *Drosophila* host also showed involvement of antimicrobial humoral response and negative regulation of cell proliferation of its host [15]. Studies on transcriptional response of *Choristoneura fumiferana* to Cry1Ab protoxin from *B. thuringiensis* showed a number of metabolic and stress-related genes that were either transcriptionally enhanced or repressed after protoxin exposure, including DNA polymerase processivity factor1, fatty acid binding, cytoskeletal constituent, serine proteinase inhibitor, serpin, translation initiation factor and so on [16].

*Bacillus* remains the major pathogenic bacterium isolated in majority of areas for *Bombyx mori* [17]. Among them, *Bacillus bombysepticus* (*Bb*) was first separated from the sick silkworm larvae cadavers and identified by Hartman in 1931 [18]. As a Gram-positive bacterium, *Bb* can produce spores and parasporal crystals. *Bb* is a resistant species among silkworm bacterial pathogens in the natural environment. *Bb* natural infection the silkworm results a typical symptom of a disease: a peutz first appears on its thoracoabdominal region or the first 1–3 abdominal aspect, and then expands to the whole body. So far, however, the pathogenesis of *Bb* or interaction between *Bb* and its host silkworm is rarely reported.

The silkworm genome contains about 14,623 genes and larvae multiple tissue transcriptional data were obtained using a 22,987 oligonucleotide probe microarray [19]–[21]. The genome-wide analysis of model insects showed that the numbers of immunity-related genes in *A. gamibae* and *D. melanogaster* are greater than those in *B. mori* and *A. mellifera*, but their innate immune signal transduction pathways are rather primitive [22], [23]. Using the particular advantages of GeneChips, *Bb* infection can be used to survey the host silkworm genome-wide responses, including its innate immune response to the pathogen at transcriptional level and to provide another detailed comprehension of the interaction between a *Bacillus* pathogen and its host. The results demonstrated that *Bb* induced the host strong response. A lot of basal metabolic pathways were significantly modulated. Genes related to poisoning that might be a key to silkworm fuliginosa septicaemia, are also regulated. Furthermore, genes of juvenile hormone synthesis and metabolism related showed upregulation, suggesting that juvenile hormone participate in host modulation during the infection. Moreover, host cellular and systemic immune responses are also induced.

## Results

### An Overview of *Bb* Oral Infection

#### *Bb* is close to *B. cereus* and *Bt.* by its 16S rRNA gene sequence analysis

**Figure 1:**
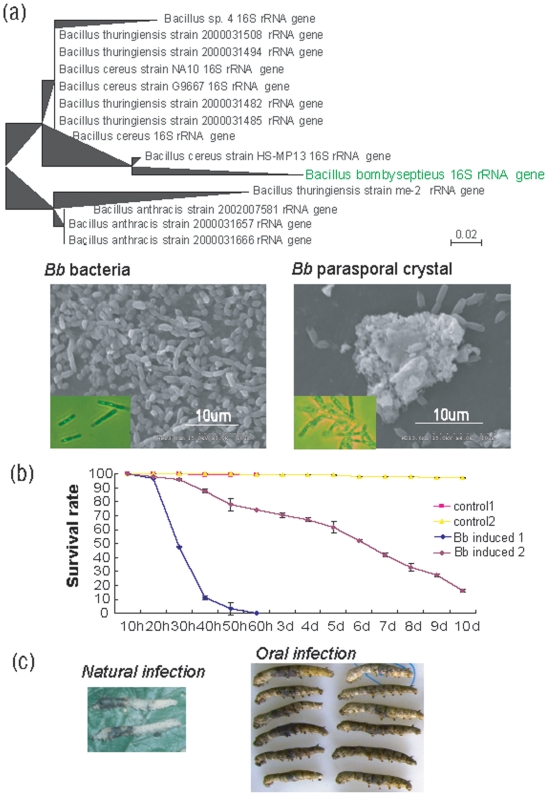
Phylogenetic tree of 16S rRNA sequences and survival curve of *Bb* infection the silkworm. (a) Phylogenetic tree with the sequences of 16S rRNA gene of *Bb* and its related species. *Bb* can produce spores and parasporal crystal. (b) Survival curve of *Bb* oral infection silkworm on day 3 of the fifth instar. Control 1: ddH_2_O feed silkworm rearing under the condition of temperature of 30°C and humidity of 90%. Control 2: ddH_2_O feed silkworm rearing under the condition of temperature of 25°C and humidity of 70%. Induced 1: *Bb* feed silkworm rearing under the condition of temperature of 30°C and humidity of 90%. Induced 2: *Bb* feed silkworm rearing under the condition of temperature of 25°C and humidity of 70%. (c) Natural infection and oral infection both can cause silkworm cuticle peutz.

We cloned a 1.5-kb sequence of the 16S rRNA gene of *Bb* using universal primers and registered it in GenBank (accession number: GQ281063). Searching GenBank, this is the first registration sequence of *bombysepticus* species. After comparison of the *Bb* 16S rRNA gene sequence with 16S rRNA sequences in the NCBI database, the resulting phylogenetic tree clearly indicated that *Bb* belongs to *Bacillus* (Fig. 1a). The dendrogram showed that *Bb* is similar to *Bacillus cereus* and that *Bt. Bacillus anthracis* is a distant relative. As a typical species of *Bacillus*, *Bb* can produce spores and parasporal crystals (Fig. 1a). The extraordinary versatility of *Bacillus* species is reflected by their ability to survive in nature. As a consequence, they are virulent toward insects and humans [24]. However, *Bb* is the first bacterium of this genus found to be highly pathogenic by natural infection for the silkworm in the sericulture production.

#### *Bb* strain cultured in LB medium is pathogenic to the silkworm

To determine the pathogenicity of the *Bb* strain cultured in LB medium, survival ratios were obtained by oral infection using silkworm larvae at day 3 of the fifth instar. The results showed that *Bb* induced >50% mortality within 30 hours after oral infection and the remaining hosts died within about 60 hours under the rearing condition of temperature of 30°C and humidity of 90%. From 20–40 h, there was a significant reduction in survival from about 90% to 10% under this condition. Rearing under the temperature of 25°C and humidity of 70%, the host died much more slowly. However, most of the silkworms died during the two infection conditions, indicating that this *Bb* line is pathogenic for the silkworm (Fig. 1b). The results demonstrated that the pathogenicity of *Bb* strain cultured in LB medium warrants further analysis. Comparison of the corpses of larvae dies naturally or due to oral infection showed that that the peutz of natural infection larvae was mainly in the anterior chest, whereas that of oral infection larvae was much larger around the middle chest, caused by substantial microorganism invasion of the digestive tract after oral infection (Fig. 1c). The peutz pattern of *Bb* oral infection larvae indicated a stronger reaction than natural infection.

#### *Bb* induces strong silkworm response and changes in expression profiles

**Figure 2:**
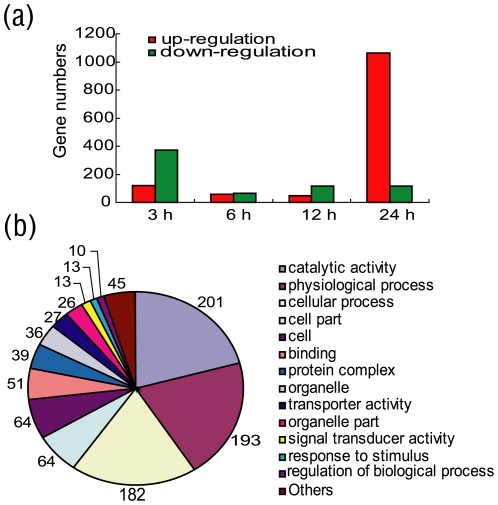
General statistics on the genes regulated after *Bb* oral infection. (a) The number of up- and down-regulated genes after *Bb* oral infection at 3 h, 6 h, 12 h and 24 h time points (h: hours post infection). (b) GO categories of total *Bb* oral induced genes.

*Bb* oral infection can change the expression of many silkworm genes, as shown by transcriptional analysis. A total of 2,436 genes were modulated, using a 2.0-fold cut-off, between 3 and 24 hours post-infection. At the beginning of the infection (3 hours post-infection, 3 hpi), the number of regulated genes showed a small peak owing to a large-scale microbial attack. At this time, 120 genes were upregulated and 374 were downregulated. At the mid-point of the infection (6 hpi to 12 hpi), *Bb* was counterattacked by the host defense system and the reproduction level of the surviving mircoorganisms in the silkworm midgut was much slower than in the medium, so the gene expression changes were relatively weak; 124 and 164 genes were modulated at 6 hpi and 12 hpi, respectively. At 6 hpi, the numbers of upregulated and downregulated genes were similar (59 and 65, respectively). At 12 hpi, the number of upregulated genes was approximately half the number of downregulated genes (49 and 115, respectively). At 24 hpi, as bacteria adapt to the internal environment of the host midgut, they enter exponential growth phase and increase bacterial toxic production. Thus, the induced gene number peaked. These genes included many metabolic system- and immune system-related genes. At this time, 1,063 genes were upregulated and 980 were downregulated (Fig. 2a).

**Figure 3:**
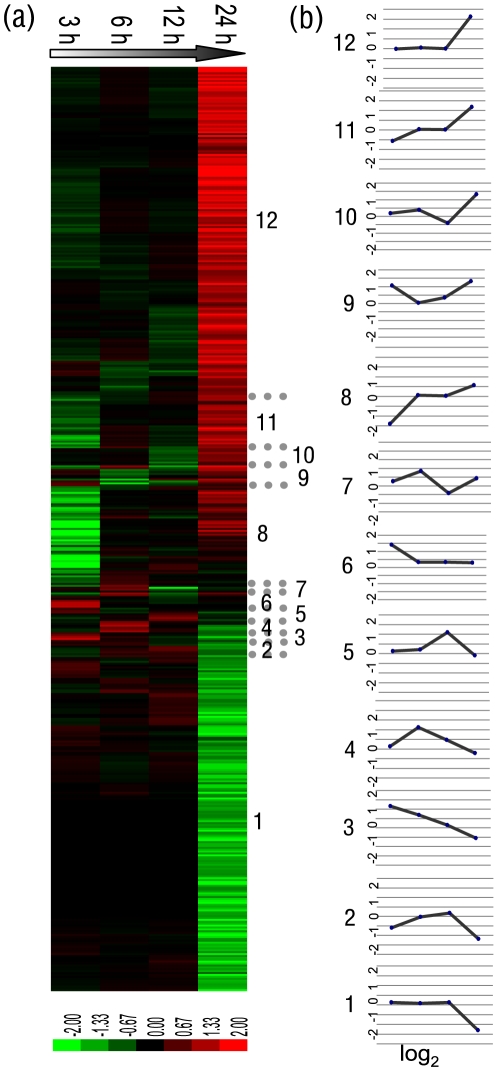
Differentially regulated genes during *Bb* infection. (a) Clustered expression profiles of regulated 2436 genes taken 2.0-fold cutoff criterion from 3 h to 24 h. For expanded ratios and annotation details, see Table S2. (b) Mean expression values of genes (log_2_) located in the defined clusters.

Overall, the cluster analysis of expression profiles of all induced genes showed time-specific patterns. By average linkage of hierarchical cluster analysis, 12 clusters of gene expression profiles could be defined using *Bb* oral infection microarray data (Fig. 3). Among the clusters, cluster 1 and cluster 12 were interesting for the significant down- and up-regulation, respectively, of large numbers of genes at 24 hpi. Their mean log_2_ ratios were close to −1.5 and 1.5. Clusters 8 and 11 were also notable for their dynamic exchange from down- to upregulation from 3 hpi to 24 hpi. Clusters 3 and 6 were significantly upregulated at 3 hpi. Clusters 7 and 4 were significant upregulated at 6 hpi, and cluster 5 was significantly upregulated at 12 hpi. The ratios and annotations of all these genes are shown in Table S2.

By GO analysis of these induced genes, their functions could be classified to 14 categories (Fig. 2b) [25]–[27]. Among these 14 families, catalytic activity, physiological process and cellular process were the largest, with 201, 193 and 182 members, respectively. These data indicate that *Bb* oral infection of the silkworm could induce a large number of enzymes involved in many basal metabolic pathways. The induced genes were then analyzed further.

#### *Bb* induces a strong midgut response–by tissue expression analysis

**Figure 4:**
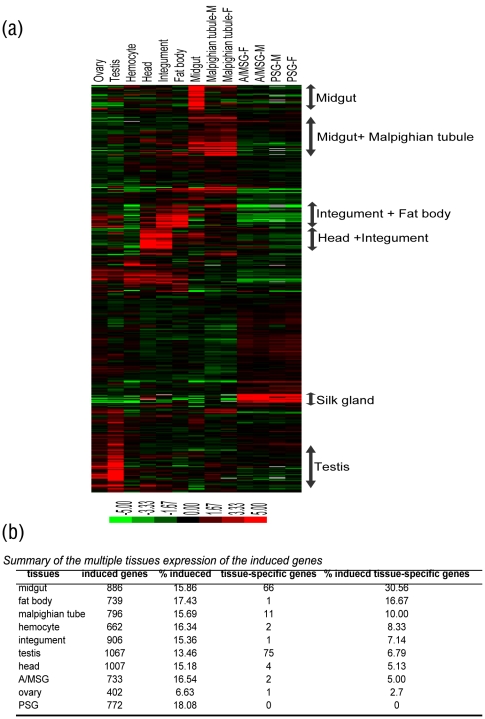
Tissues expression data on day 3 of the fifth instar of the induced genes. (a) Cluster of multiple tissues expression data of the induced genes. For details of these data, see Table S3. (b) The summary of the multiple tissues expression of the induced genes.

Pathogen infections always have tissue-specific features [28]. Multiple tissue expression data from day 3 of the fifth instar showed that 1,403 of the 2,436 induced genes have multiple tissue expression, indicating that almost all of the silkworm tissues were affected by the infection [19]. At the same time, *Bb* induced genes that showed some organizational preference, as more genes were highly expressed in the midgut, integument and testis (Fig. 4a). Of the 1,403 genes, 886 (63%) genes were expressed in the midgut (signal value>400), of which 68 were midgut tissue-specific; these 68 accounted for 30.56% of all the midgut specific genes, the highest level for all tissues (Fig. 4b). Many of the induced midgut genes encode metalloproteinases, hydrolases, lipases and chitin structural proteins. The midgut, as the direct infection organ, showed a relatively high level of tissue-specific gene expression.

### Basal Metabolic Pathways Involved after *Bb* Infection

**Figure 5:**
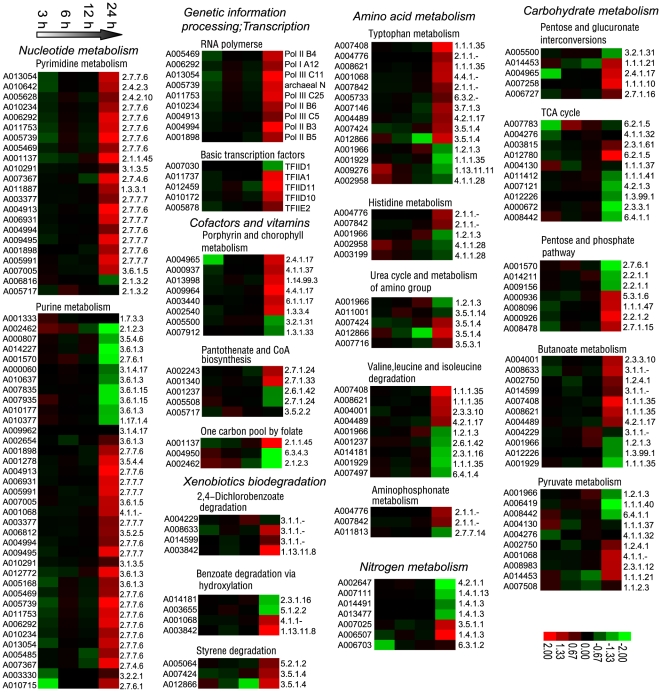
Cluster analysis of basal metabolism pathways. Detailed expression profiles of functionally pathways related sets of genes. The gene ID of SilkDB are shown on the left (for each gene ID, we used the letter A to substitute letters BGIBMGA to simplify the expression) and the serial number of KEGG database are shown on the right. For a detailed view, see Table S1 and Table S4.

The basic metabolism is important for organisms to maintain their normal physiological activities. Analysis of pathogen-induced host basal metabolic pathways will help us to investigate the interaction between them. Using the significant standard criteria of pathway prediction value P<0.05 and induced ratios>2 or <0.5, we searched the KEGG database to filter out the host metabolic pathway-related genes [29]–[33]. In total, six types of basal metabolic systems were identified after the infection, including genetic information processing and transcription, nucleic acid metabolism, metabolism of cofactors and vitamins, xenobiotics biodegradation and metabolism, amino acid metabolism and nitrogen metabolism, and carbohydrate metabolism (Fig. 5). These pathways are involved in many normal silkworm physiological metabolic processes. The detailed enzyme names, gene IDs, KEGG prediction P-values and their typical catalytic reactions are shown in Table S1, and the ratios of the pathway groups are shown in Table S4.

#### Basic genetic information processing and transcription genes

Nucleotide biosynthesis is critical for growth of bacteria in human blood [34]. For the silkworm, both the maintenance of normal growth and development for itself and proliferation of bacteria in its hemolymph need nucleotide acids for the transmission of genetic information and protein synthesis. As a result, the genes encoding RNA polymerases and basic transcription factors were modulated (Fig. 5). Nine RNA polymerases were upregulated at 24 hpi, including eukaryotic Pol II B4, eukaryotic Pol I A12, eukaryotic Pol III C11, archaeal N, eukaryotic Pol III C25, eukaryotic Pol II B6, eukaryotic Pol III C5, eukaryotic Pol II B3 and eukaryotic Pol II B5. Similarly, basic transcription factors were also induced by *Bb* infection, including TFIID11, TFIID1, TFIIA1, TFIIE2 and TFIID10. Only TFIID1 was downregulated, and the other five genes were upregulated. These data indicated that the synthesis of nucleic acids and proteins was increased after infection. The basic genetic information processing and transcription genes upregulation after *Bb* infection are consistent with those of Neill and Ridpath for ubiquitous viral pathogen of cattle bovine viral diarrhoea virus (BVDV)-infected Madin-Darby bovine kidney cells, indicating the modulation of these genes might be general after pathogens infection [35].

#### Pyrimidine and purine metabolism

*In vivo*, nucleic acid metabolism has an important role [36]. Disruption of an organism's nucleic acid metabolism can cause serious diseases such as gout in humans [37]. Twenty-two genes involved in pyrimidine metabolism pathway were induced after the infection (Fig. 5), including 11 types of enzymes that can catalyze at least 11 typical biochemical reactions (Table S1). The largest subfamily was that of RNA uridylyltransferase (EC 2.7.7.6), containing nine members. The second largest subfamily was DNA deoxynucleotidyltransferase (DNA-directed) (EC 2.7.7.7), containing four members. These two subfamilies are also involved in the purine metabolism pathway. Twenty of the 22 genes were upregulated. More genes of the purine metabolism pathway were modulated; 37 were modulated, 26 of which were upregulated. These genes included 15 types of enzymes that catalyze at least 15 typical biochemical reactions in the purine pathway. Genes coding 15 types of enzymes were upregulated, including 5′-phosphoribosyl-5-amino-4-imidazolecarboxamide formyltransferase (EC 2.1.2.3), nucleoside-triphosphatase (EC 3.6.1.15) and so on. These results also show that *Bb* infection accelerates the silkworm nucleic acid metabolism.

#### Cofactor and vitamin metabolism

Vitamins are essential small compounds to maintain normal activities. After *Bb* infection, cofactor and vitamin metabolic pathway genes were modulated, including genes from the porphyrin and chlorophyll metabolism pathways, pantothenate and CoA biosynthesis pathways and one carbon pool by folate. Eight genes encoding enzymes involved in porphyrin and chlorophyll metabolism were regulated, of which six and two were up- and downregulated, respectively (Fig. 5 and Table S4). They encoded eight types of enzymes in this pathway, including uroporphyrinogen-III carboxy-lyase (EC 4.1.1.37), hydrolases (EC 3.2.1.31), NADP+7, 8-oxidoreductase (EC 1.3.1.33) (Table S1). Five genes related to pantothenate and CoA biosynthesis were modulated, including 3′-dephospho-CoA 3′-phosphotransferase (EC 2.7.1.24), 2-oxoglutarate aminotransferase (EC 2.6.1.42), (R)-pantothenate 4′phosphotransferase (EC 2.7.1.33) and 5, 6-dihydropyrimidine amidohydrolase (EC 3.5.2.2). In addition, three genes related to one carbon pool by folate were also modulated, including tetrahydrofolate ligase (EC 6.3.4.3), dUMP C-methyltransferase (EC 2.1.1.45) and transferases (EC 2.1.2.3). These results show that the three cofactor and vitamin metabolic pathways were sensitive to the infection.

#### Xenobiotics biodegradation and metabolism

Pathways involved in xenobiotic biodegradation including 2,4-dichlorobenzoate degradation, benzoate degradation via hydroxylation and styrene degradation were induced after *Bb* oral infection; most of these genes were upregulated (Fig. 5, Table S1 and Table S4). The 2,4-dichlorobenzoate degradation pathway-related genes encoding hydrolases (EC 3.1.1.-) and oxygen 4, 5-oxidoreductase (EC 1.13.11.8) were modulated. The expression of oxygen 4, 5-oxidoreductase was increased more than six-fold at 24 hpi, when the bacteria produced large quantities of bacterial toxins within the silkworm host, indicating that oxygen 4,5-oxidoreductase might be involved in the detoxification of bacterial toxins such as parasporal crystal [38]. Four genes involved in benzoate degradation via the hydroxylation pathway were also regulated. In addition to the above-mentioned oxygen 4, 5-oxidoreductase gene, acyl-CoA:acetyl-CoA C-acyltransferase (EC 2.3.1.16), mandelate racemase (EC 5.1.2.2) and lyases (EC 4.1.1-) were also regulated. For example, lyases, which have also been shown to be related to the pyruvate metabolism pathway, were highly expressed in the midgut and malpighian tubules; at 24 hpi, expression levels were more than three times the baseline levels. Thus, we speculated that lyases might be involved in the detoxification of midgut and malpighian tubules. In addition, three genes involved in the styrene degradation pathway were modulated, including acylamide amidohydrolase (EC 3.5.1.4) and 4-maleylacetoacetate cis-trans-isomerase (EC 5.2.1.2). Also, three members of the cytochrome family involved in detoxification, including the cytochrome P450 family 4 (A001003) and cytochrome P450 (A013237, A013241), were upregulated. These results illustrated that *Bb* infection accelerated the xenobiotics biodegradation and metabolism, probably mainly caused by the released bacterial toxins.

#### Amino acid metabolism and nitrogen metabolism

Amino acids are important molecules in every organism. Pathways of tryptophan metabolism, histidine metabolism, valine, leucine and isoleucine degradation, urea cycle and amino group metabolism, and aminophosphonate metabolism involved in amino acid metabolism and nitrogen metabolism were modulated by the infection (Fig. 5, Table S1 and Table S4). Of the genes involved in amino acid metabolism, more were upregulated than downregulated. For example, 14 genes involved in the tryptophan metabolism pathway, including (S)-3-hydroxyacyl-CoA: NAD+ oxidoreductase (EC 1.1.1.35), transferases (EC 2.1.1.-), and acylamide amidohydrolase (EC 3.5.1.4), eleven of the 14 genes were upregulated and only 3 were downregulated. These results show that *Bb* infection promoted the amino acid metabolism. Nitrogen metabolism is a basic pathway that maintains the balance of nitrogen in organisms. After *Bb* infection, all of the seven nitrogen-pathway regulated genes including NAD (P) +oxidoreductase (EC 1.4.1.3) were downregulated, indicating that nitrogen metabolism was slowed by the infection.

#### Carbohydrate metabolism

Carbohydrate metabolism is a basal metabolic process and provides carbon and energy for organisms [39], [40]. Enzyme-encoding genes of basic carbohydrate metabolic pathways were modulated by the infection, including those responsible for pentose and glucuronate interconversions, the citrate cycle (TCA cycle), pyruvate metabolism, the pentose phosphate pathway and butanoate metabolism (Fig. 5, Table S1 and Table S4). Many genes related to these pathways were upregulated. For example, for the pentose and glucuronate interconversions pathways, five genes were induced, including glucuronosyltransferase (EC 2.4.1.17), beta-D-glucuronoside glucuronosohydrolase (EC 3.2.1.31), NAD (P)+ 1-oxidoreductase (EC 1.1.1.21), NADP+ 4-oxidoreductase (EC 1.1.1.10) and L (or D)-ribulose 5-phosphotransferase (EC 2.7.1.16). Four of these five genes were upregulated at 24 hpi. For example, NADP+ 4-oxidoreductase (EC 1.1.1.10) was highly expressed in the fat body, and the expression was increased by a factor of four. The fat body is the site of energy storage, thus we speculated that the energy metabolism of the silkworm fat body was accelerated. Ten genes involved in the TCA cycle were regulated, including CoA ligase (EC 6.2.1.5), NAD+ oxidoreductase (EC 1.1.1.41), oxaloacetate carboxy-lyase (EC 4.1.1.32), and carbon-dioxide ligase (EC 6.4.1.1), however, many of which were downregulated at 24 hpi. Similar to amino acid metabolism, for carbohydrate metabolism, more genes were upregulated than downregulated, indicating that carbohydrate metabolism was accelerated to provide more energy.

### Similar to *Bt*, *Bb* Can Induce Silkworm Larvae Poisoning Related Response

**Figure 6:**
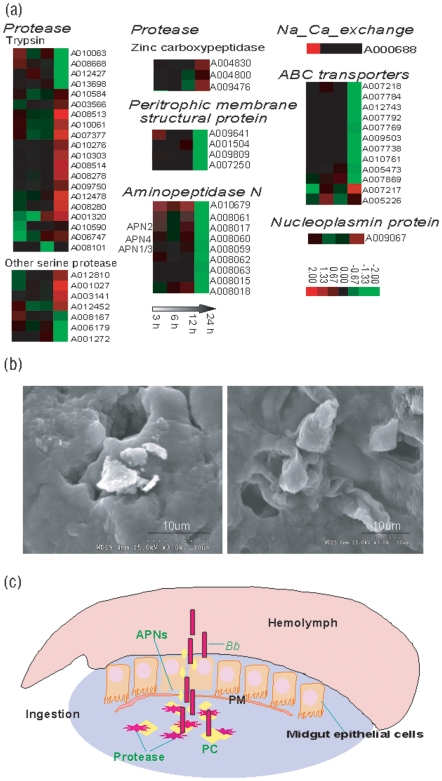
Functional groups of silkworm poisoning related genes. (a) Cluster analysis of silkworm poisoning related genes referencing to *Bacillus thuringiensis* toxins. For a detailed view of the cluster ratios, see Table S4. (b) SEM pictures of the silkworm middle gut epithelium after *Bb* infection. The pictures showed *Bb* crystals accumulated in the host intestinal epithelial cells and pores were formed in the host intestinal epithelial cells after *Bb* oral infection. (c) Schematic overview of the process that *Bb* damage silkworm midgut, into the hemolymph. After oral infection, *Bb* interred the ingestion of silkworm. The parasporal crystal (PC) produced by *Bb* could be digested by midgut proteases. The digested PC could pass through the peritrophic membrane (PM) to bind the aminopeptidase N receptors (APNs) of the midgut epithelial cells to damage them. *Bb* can go to hemolymph from the damaged midgut.

As a typical *Bacillus*, *Bb* can produce spores and crystal toxins. *Bb* toxins and spores are thought to induce a similar host response compared to *Bt*. The poisoning happened in the midgut. First, when *Bb* enters the midgut of the silkworm, parasporal crystal was released and degraded by proteases of the host midgut. The dissolved monomer of the toxin is activated and poisonous [41]–[47]. Parasporal crystal, as a type of protein, can be degraded by serine proteases [48]. As expected, the host expression levels of proteases including trypsins, other serine proteases, and zinc carboxypeptidase were regulated (Fig. 6a). Trypsins, as a type of serine protease that can selectively hydrolyze proteins, were considered to be the main hydrolases responsible for *Bt* toxin hydrolysis in the insect midgut [49], [50]. In our study, 20 trypsins were induced after the infection, many of which were upregulated. For example, A008513, with tissue expression only in the midgut, was upregulated by more than 5-fold at 24 hpi. Other serine proteases, the usual role of which is to disrupt macromolecular protein peptide bonds, were also regulated, including seven other serine proteases, including peptidase_S24 (PF00717, A001027 and A012810), peptidase_S28 (PF05577, A012452 and A008167), peptidase_S51 (PF03575, A003141) and peptidase_S9 (PF00326, A006179 and A001272). Among these seven proteases, A001027, A012810, A012452 and A003141 were significantly upregulated at 24 hpi. Three members of the zinc carboxypeptidase family (Peptidase_M14, PF00246), with the general function of hydrolysis of carboxyl terminal amino acids, were only or highly expressed in the midgut and were also upregulated after the infection [51].

The dissolved *Bb* crystal toxin can damage the silkworm midgut peritrophic membrane (PM) [52]. The PM of the insect midgut is considered as a non-cell semi-permeable membrane mainly composed of chitins and proteins [53], [54]. The PM can promote insect digestion and prevent the invasion of pathogenic microorganisms by forming a natural barrier of midgut epithelial cells. After the infection, four PM structural protein genes (domain CBM_14, PF01607), with high tissue expression only in the midgut, were downregulated at 24 hpi (Fig. 6a). Among them, the expression level of A009641 showed a dynamic change from more than twice of up-regulation at 3 hpi to about twice of down-regulation at 24 hpi. The results indicated that, with time, the production of PM proteins decreased, resulting in damage of the PM and allowing passage of the *Bb* crystal toxin. Thus, the toxins can be observed accumulated on the host intestinal epithelial cells (Fig. 6b).

The activated toxin can bind the metallopeptidase receptors of the brush-border membrane vesicles of the host midgut epithelial cells, which allows it to cross the cell membrane [55]. Aminopeptidase N receptors (APNs), members of the zinc metallopeptidase M1 family (PF01433), are a specific type of exonuclease that can digest proteins or peptides from their N-terminal amino acids. Previous studies have shown that the APNs of insects are the receptors for the *Cry* toxin [56]–[58]. A total of 16 APNs containing the Peptidase_M1 domain can be identified in the silkworm genome, 3 of which have also been identified as *Cry* toxin receptors: *BmAPN1* (A008059, NCBI No. AF084257 and AF352574), *BmAPN2* (A008017, NCBI No. AB011497) and *BmAPN4* (A008060, NCBI No. AB013400) [44], [46], [59]. In this study, nine APN receptors were modulated by the infection (Fig. 6a). Most showed dynamic expression changes, which manifested as a low level of upregulation at 3 hpi and downregulation at 24 hpi. A010679 and A008061 showed more intense upregulation than the other genes; the increase in expression level was approximately double at 3 hpi and 12 hpi. Three of the above-mentioned *Cry* toxin receptors, however, showed weak upregulation from 3 hpi to 12 hpi than A010679 and A008061. This result reflected the selective binding features of the silkworm APNs.

Some genes involved in infiltration balance also showed regulation after the infection. For example, the sodium/calcium exchange protein A000688 was upregulated by a factor of about seven at 3 hpi, indicating that *Bb* can rapidly create a sodium/calcium exchange imbalance [60] (Fig. 6a). ABC transporters are involved in the transmembrane export or import of a wide variety of substrates from small ions to macromolecules [61], [62]. After *Bb* oral infection, 12 ABC transporter-encoding genes were regulated. Most were downregulated by the infection, indicating that exchange between the outside and inside of the membrane was weakened. A nucleoplasmin protein, A009067, which is only expressed in the midgut, showed upregulation at 24 hpi. Nucleoplasmin, a nuclear molecular chaperone, is usually considered to be involved in nucleosome assembly, chromatin reconstruction, material transport, and cell apoptosis [63], [64]. The upregulation of this gene might indicate increased exchange between the nucleus and cytoplasm after the infection. Finally, the infiltration balance and material exchange of the insect body are disrupted. Pores can be observed in the host intestinal epithelial cells and intestinal epithelial cells microvillus were vacuolated (Fig. 6c). Infiltration balance disorder is the main cause of insect death due to *Cry* toxin [65]. Thus, these results indicate that *Bb* can poison the silkworm via a similar mechanism as the *Bt Cry* toxin.

### *Bb* Induced Juvenile Hormone Synthesis and Metabolism-Related Gene Upregulation

**Figure 7:**
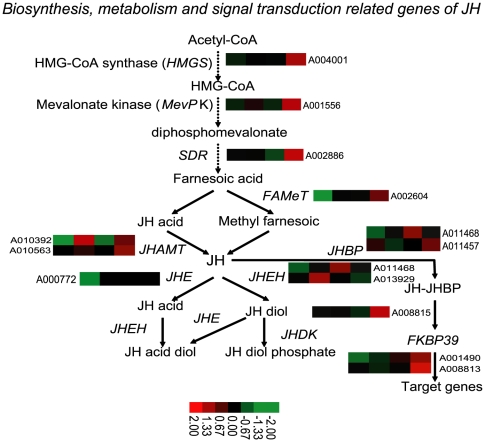
Diagram showing the induced biosynthesis, metabolism and signal transduction related genes of juvenile hormone in the silkworm. The expression pattern of each gene was indicated near the gene name. Diagram of the pathway is referenced form Schooley and Baker, 1985. For a detailed view of the cluster ratios, see Table S4.

Juvenile hormone (JH) is the main hormone that regulates maintenance of the physical form of larvae, pigment occurrence in larvae and reproduction activities [66], [67]. The silkworm JH biosynthetic pathway can be divided into two main stages: the early steps, up to farnesyl diphosphate (FPP) formation, belong to the mevalonate pathway, and the late steps control the conversion of FPP into JH [68]. JH is then metabolized to JH acid diol and JH diol phosphate [69]. On the other hand, JH can bind to JH-binding proteins and regulate target gene expression. So far, little is known about the pathogen infection and modulation of insect JH. Interestingly, in this analysis, a lot of JH synthesis, metabolism and JH-binding genes were shown to be upregulated after *Bb* oral infection (Fig. 7).

The expression of the HMG-CoA synthase gene (*HMGS*, A004001), responsible for catalysis of acetoacethl-CoA to HMG-CoA, was increased by nearly three-fold [70]. Phosphomevalonate kinase gene (*MevPK*, A001556), encoding an enzyme that converts phosphomevalonate to diphosphomevalonate, was upregulated by more than two-fold [68]. Short-chain dehydrogenase gene (*SDR*, A002886), which converts farnesal to farnesoic acid, was upregulated by more than three-fold [71]. The JH acid methyltransferase (JHAMT) is an enzyme that converts JH acids or inactive precursors of JHs to active JHs at the final step of JH biosynthesis pathway in insects [72]. One of the two JHAMT coding gene (A010563) was upregulated from 3 hpi to 24 hpi, whereas the other (A010392) was downregulated from 3 hpi to 24 hpi. At the same time, the Farnesoic acid O-methyltransferase (*FAMeT*, A002604), which is involved in the conversion of farnesoic to methyl farnesoate, was downregulated, indicating that the JH acid branch pathway might be a method of JH biosynthetic after *Bb* infection [73].

Similarly, some genes related to JH metabolism were also modulated. Two JH epoxide hydrolase genes (JHEH, A011468 and A013929), involved in the regulation from JH to JH diol, were upregulated at 12 hpi and 6 hpi, respectively [74]. However, JH esterase (*JHE*, A000772) on HJ to JH acid diol pathway branch, showed downregulation from 3 hpi to 24 hpi. Two genes coding JH diol kinase (JHDK, A008813 and A008815), involved in the conversion of JH diol to JH diol phosphate, both showed more than three-fold upregulation at 24 hpi [69]. JH binding protein (JHBP) can bind to JH and transport JH from the corpus allatum to target tissues [75]. Two genes encoding JHBPs (A011457 and A011458) were upregulated from 3 hpi to 24 hpi. FKBP39, a *Drosophila* homolog, can bind the immunosuppressive drug FK506 and mediate the binding of the target element of JH and showed a dynamic upregulation from 3 hpi to 24 hpi [76].

The general function of insect JH is to maintain larvae morphology. After a microorganism infection, insects will pupate earlier than normal and lay eggs to preserve their future generations. However, after a pathogenic granulovirus infection, the insect cannot pupate, indicating that the JH concentration is maintained at a high level after the infection [77]. At the same time, we detected a lot of JH-related genes which were also upregulated after NPV oral infection (data not shown). In addition, synthetic JH can improve the production of NPV in insect cells, indicating the role of JH in the reproduction of NPV [78], [79]. Hence, we speculate that *Bb* might active silkworm JH synthesis, metabolism and binding related genes to extend the silkworm larvae stage and provide nutrients for its reproduction, similar to NPV.

### *Bb* Induced Silkworm Immune Response

#### *Bb* can induce silkworm cellular response

Once a pathogenic microorganism crosses the natural barrier of its host, it has to face a strong host immune response. For insects, the non-specific cellular immune response, which is mainly mediated by hemocytes including crystocyte, plasmatocyte and lamellocyte, is the first barrier to prevent and remove most viruses and many bacteria, parasites and fungal infections [80]. Many immune effectors, such as lysozyme, lectin, and scavenger receptors (SCRs), also work for the cellular immune response [81].

**Figure 8:**
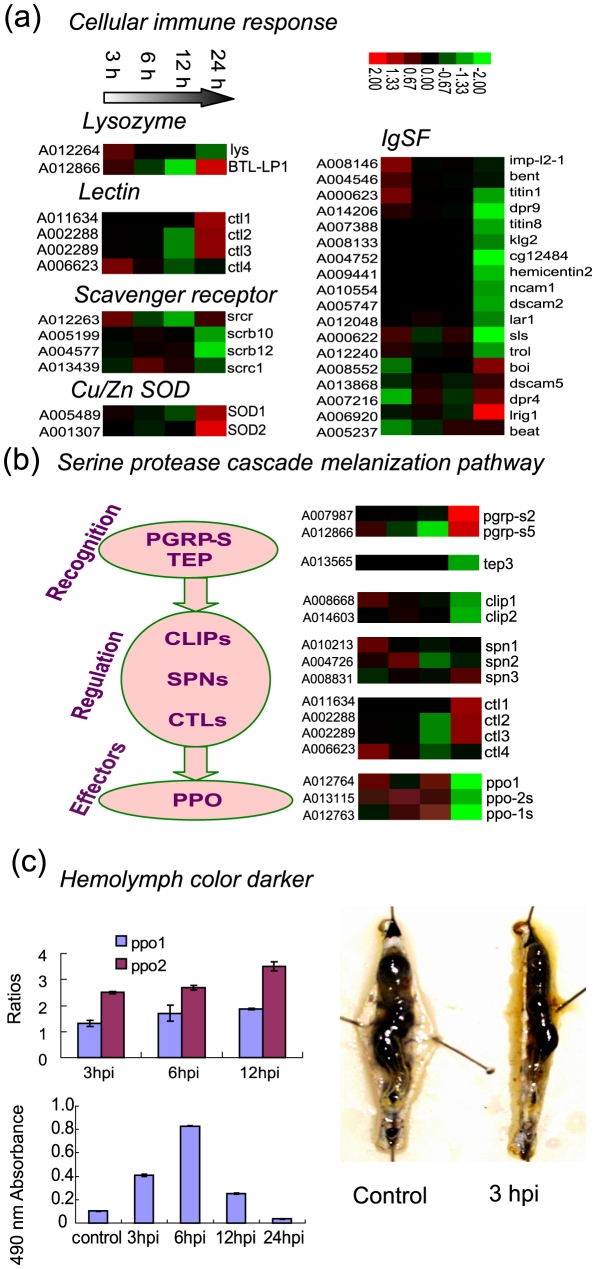
*Bb* induced silkworm cellular immune response and serine protease cascade melanization pathway. (a) Cluster of cellular immune response families. (b) Cluster diagram of the induced synthesis of melanin by serine protease cascade melanization pathway related genes. For a detailed view of the cluster ratios, see Table S4. (c) Real time PCR analysis of ppo1 and ppo2 ratios of *Bb* infected whole larvae comparing to non-induced control and 490 nm absorbance of hemolymph during the infection, and the picture of the hemolymph melanization 3 h after *Bb* oral infection.

After *Bb* enters the silkworm hemolymph, the cellular immune response is triggered (Fig. 8a, Table S4). The expression levels of two lysozymes, which can dissolve and kill bacteria, were modulated. For example, the expression of A007987, a bacteriophage T7 lysozyme-like protein 1 (BTL-LP1), was upregulated more than three-fold at 24 hpi. The encapsulation process involved in cell adhesion, can lead to bacterial death [80]. Lectins, a type of hemonectin, can condense microorganisms while immune response [82], [83]. The condensed microorganisms can then easily be phagocytosed or encapsulated by plasmatocytes and cystocytes, ultimately leading to the formation of black knots. Four of the 21 identified C-type lectin genes were regulated dynamically [23]. Also, C-type lectins (CLTs) participate in signal regulation, whereas the immune signal is transduced during melanization [84]. Of the SCRs, 2 of the 13 identified SCR class B and one class C member were modulated. One class B SCR gene, known to be involved in the phagocytosis of microorganisms in *Drosophila*, SCRB10, was upregulated at 3 hpi and 24 hpi. In addition, Cu/Zn superoxide dismutase (SOD1), which can limit parasite development in *Anopheles*, was also upregulation during the infection [23]. Another SOD gene, SOD2, was upregulated too at 24 hpi. Members of the immunoglobulin superfamily (IgSF), which are related to the specific immune response in vertebrate blood, can also be induced in insects in response to pathogens [85]. After *Bb* oral infection, the expression levels of 18 IgSF genes were modulated. *Boi* (A008552), a signal transduction protein, showed upregulation at 24 hpi. Lrig1 (A006920), a leucine-rich repeat immunoglobulin involved in the pathogen response, was also upregulated at 24 hpi [86]. However, two dscam genes (dscam2 and dscam5), known to be involved in cell adherence, were shown to be downregulated in this study [87], [88].

#### *Bb* induced host serine protease cascade melanization pathway

Melanin formation in the hemolymph is caused by the serine protease cascade melanization pathway after pathogen infection [89]–[91]. After *Bb* oral infection has induced an immune response, the serine protease cascade melanization pathway was activated. First, expression of two of the six peptidoglycan recognition protein short-type proteins, PGRP-S2 and PGRP-S5, was induced [23](Fig. 8b). PGRP-S2 (A007987), which is only expressed in the midgut, showed almost five-fold upregulation at 24 hpi. PGRP-S5 (A012866), which is expressed at high levels in the integument and fat body, showed more than three-fold up-regulation at 24 hpi, indicating that *Bb* can be recognized by PGRP in the fat body and integument [23]. However, none of the six long-type PGRPs showed melanization-related regulation in *Drosophila*, and four of the β-glucan recognition proteins (βGRP) involved in the PPO-activating system in the silkworm in previous study, showed significant regulation in this analysis [92]. PGRPs are also related to the Toll and Imd signal transduction pathways of the insect innate immune system [93]. Thioester-containing proteins (TEPs) also showed recognition receptor activity in *A. gambiae* during malaria parasite infection [84]. Unlike *A. gambiae*, which contains 15 TEPs, the silkworm only contains three TEPs. During *Bb* infection, only TEP3 (A013565), which has no orthologs in *Drosophila* and *Anopheles*, was downregulated at 24 hpi. After microorganism recognition, regulators of the serine protease cascade, including the CLIP serine proteases (CLIPs), serpins (SPNs) and CTLs showed modulation [84]. Expression levels of two of the 15 silkworm CLIPs, CLIP1 (A008668) and CLIP2 (A014603), were regulated [23]. CLIP1, which is only expressed in the integument and head, showed about twice of up- to down- regulation from 3 hpi to 24 hpi. Expression levels of three of the 26 serpins, SPN1(A010213), SPN2 (A004726) and SPN3 (A008831), were regulated [23]. The expression level of SPN1, which is highly expressed in the integument, head and hemocyte, was doubled during the early stages of infection (3 hpi). Finally, melanization effectors, including three prophenoloxidase genes (proPOs, PPOs), were activated. PPO1 (A012764) and the PPO1 subunit (PPO-1S, A012763), which are expressed at very high levels in the hemolymph, were upregulated from 3 hpi to 12 hpi. The PPO-2 subunit (A013115), whish is highly expressed in the hemolymph and head, also showed upregulation from 3 hpi to 12 hpi [94], [95]. The real time PCR analysis of PPO1 (A012764) and PPO2 (A013115) supported the above result (Fig. 8c). Activated PPO can catalyze the oxidation of mono- and diphenoles to orthoquinones, which non-enzymatically polymerize to melanin. These results show that many of the melanization pathway genes were upregulated from 3 hpi to 12 hpi, indicating that melanization occurred during the early stages of infection. Also, the dissected body and 490 nm hemolymph absorbance showed that the host hemolymph became markedly darker compared with uninfected insects at the early stage of infection (Fig. 8c).

#### *Bb* induced host systemic immune response

**Figure 9:**
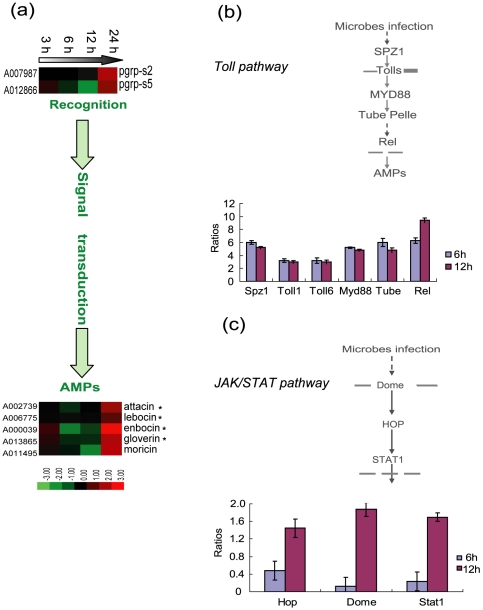
*Bb* induced silkworm systemic immune response. (a)*Bb* can be recognized by short-type of peptidoglycan recognition proteins (PGRPs) PGRP-S2 and PGRP-S5, then, mainly through Toll signal pathway, antimicrobial peptide coding genes were induced (the asterisk indicated a family, for the probe can not distinguish a member out from highly homologous sequences). (b) Toll pathway signal transduction diagrammatic sketch and real time PCR ratios of corresponding genes. (c) JAK/STAT sinal transduction diagrammatic sketch and real time PCR ratios of corresponding genes. For a detailed view of the cluster ratios, see Table S4.

To combat microorganism infections, the insects rely on multiple innate defence reactions such as local and systemic immune responses [96]. Systemic immune responses involve pathogen recognition, signal transduction, and AMP expression. In this analysis, two short PGRPs were upregulated as expounded in serine protease cascade melanization pathway section. The insect systemic immune response, involves signal transduction of the Toll, Imd, and JAK/STAT pathways [80], [97]. However, for the silkworm, little is known about the corresponding pathway by which AMPs are induced. To determine this question, we searched the microarray data of the silkworm Toll, Imd and JAK/STAT signaling pathway genes. However, microarray data did not show any regulation due to its much lower sensitivity (signal values of most of them are less than 400 and therefore can not be identified “expression” to current standards) (Table S6). So, the real-time RT-PCR analysis of the genes indicating the regulation of innate immunity (*SPZ1*, *Toll1*, *Toll6*, *Myd88*, *Tube* and *Rel* of Toll pathway and *Hop*, *Dome* and *Stat1* of JAK/STAT) was performed (Fig. 9b, c, Table S6). Comparison the microarray data and the real time PCR data, although the degree of modulation was different, the tendency of regulation of most genes were similar. Because previous RT-PCR analysis of the IMD pathway genes showed very weak modulation, we gave up its testing by real time PCR analysis (data not shown).

Most of our knowledge of the Toll pathway was obtained from the initial studies in *Drosophila*, and it has been shown to be activated by fungi and Gram-positive bacterial infection. The Toll receptor is activated upon binding by a cleaved form of spätzle, which is proteolytically processed after activation by secreted recognition molecules (PGRP-SA, GNBP1) [98], [99]. Mature spätzle binds as a dimer to Toll, thereby inducing its dimerization at the plasma membrane. This causes the recruitment of three death-domain-containing proteins, MyD88, Tube and Pelle [100]–[102]. Then, the Rel transcription factors are released from the cytoplasm to the nucleus to active APMs expression [103], [104]. In the silkworm, the Toll pathway is conserved in terms of innate immunity [23]. Our real time PCR analysis showed that *Spz1*, *Toll1*, *Toll6*, *MyD88*, *Tube* and *RelA* can be induced after *Bb* infection at 6 hpi and 12 hpi (Fig. 9b). *Spz1*, mature peptide injection of which to the silkworm can significantly upregulate transcription of eight antimicrobial peptide genes (*attacin* 2, *cecropin A1*, *-B1*, *-D1*, *gloverin A5*, *-B*, *lebocin*-3, and *moricin-A1*), are highly expressed at the beginning of the infection and showed marked upregulation from 3 hpi to 48 hpi [99]. The Toll receptor *Toll1*, *Toll6* also showed upregulation, as did the adapters *MyD88* and *Tube*. *MyD88* is only expressed after *Bb* infection. The Rel transcription factor *RelA*, which can markedly activate *lebocin 4*, was upregulated more than 6 times [105]. Thus, we can speculate that the silkworm Toll pathway can be activated by *Bb* oral infection.

The JAK/STAT pathway, originally identified through its role in embryonic segmentation, has three main cellular components in *Drosophila*: the receptor Domeless, the Janus Kinase (JAK) Hopscotch, and the STAT transcriptional factor [106]–[108]. This pathway is thought to participate in antiviral and inflammatory responses [109], [110]. After *Bb* oral infection, the membrane receptor *Dome* was upregulated at 12 hpi (Fig. 9c). *Hop* and stat1 also showed upregulation 12 hpi. This results illustrated that *Bb* oral infections can cause a weak JAK/STAT1 pathway response.

After that, AMP genes, including those of the attacin, enbocin, gloverin, lebocin and moricin subfamilies, showed upregulation at 24 hpi, indicating that *Bb* can induce the silkworm systemic immune response. The moricin subfamily, which shows the greatest expression levels in the malpighian tubules, showed the greatest upregulation at 24 hpi; the expression level was increased more than eight-fold. In general, these results show that *Bb* is recognized by silkworm PGRPs and the signals can be transduced mainly by the Toll pathway, leading to the production of AMPs.

## Discussion

*B. bombysepticus* is closely related to *B. cereus* and *B. thuringiensis*, and is a typical natural pathogen of the silkworm *B. mori*. The host transcriptional analysis after *Bb* oral infection presented here will help us to understand the relationship between the *Bacillus* pathogen and *B. mori* host. In this report, we found that many pathways involved in the silkworm physiological functions were changed after the infection.

First, the basal metabolic pathways were most involved. The results demonstrated that *Bb* can affect six types of basal metabolic system-related genes, leading to overexpression or reduced expression. These types include genetic information processing and transcription, carbohydrate metabolism, amino acid metabolism and nitrogen metabolism, nucleotide metabolism, metabolism of cofactors and vitamins, and xenobiotic biodegradation and metabolism. During *Bb* infection, particularly after the pathogen entered the hemolymph, the silkworm has to meet the basic material and energy needs of *Bb* growth and reproduction, leading to the upregulation of some metabolic pathway genes. During *Bb* infection, a partial energy originally used for silk protein synthesis will be assigned to microorganism reproduction. A previous study on the functional genomics of *Buchnera* and the ecology of aphid hosts showed that *Buchnera* have lost many capabilities, indicating that the host must compensate for gene losses and integrate symbiont functions into the mutualistic system [111]. However, the mechanisms of the basal metabolic pathways modulation for both host and pathogen are not exactly known, becoming a particularly intriguing question to investigate.

*Bb* can produce spores and parasporal crystal. Therefore, it is thought to have similar pathogenicity to *Bt*, and a further study has confirmed this. Seven of the nine APNs showed substantial or modest upregulation from 3 hpi to 12 hpi. Three APNs for *Bt* toxins (A008017, A008060 and A008059) did not show the highest expression levels in this analysis, but did show modest upregulation. These results demonstrated the specific relationship between host APNs and pathogen toxins. However, at 24 hpi, all of the nine APNs showed downregulation. The SEM picture showed that toxins accumulated on intestinal epithelial cells and the midgut epithelial cells and microvillus had been damaged by the toxins. Thus, the infiltration balance was broken.

In higher vertebrates, immunomodulator hormones such as glucocorticoids and growth hormones can communicate between the immune and nervous systems during infection [112]. In insects, although relationships between hormones and pathogens infection are rarely studied, insects can be regulated by JH during development and metamorphosis is well known [68]. Interestingly, JH biosynthesis- and metabolism-related genes were upregulated after the infection in this study. Also, many of these genes were upregulated after *B. bassiana* and NPV oral infection of the silkworm (data not shown). Previous studies have shown that JH expression is beneficial for NPV reproduction. Indeed, after a powerful pathogen infection, JH regulation is an effective method to prevent host insect metamorphosis and provide material and energy for pathogen reproduction. At the same time, we found the ecdysone receptor gene 20E (A006767) was downregulated at 24 hpi. Further studies are required to resolve this issue.

Melanin accumulation can form peutz after pathogen infection, and this generally occurs in the insect midgut and integument. Peutz formation after bacterial infection is universal. A *Bb* natural infection can lead to the formation of a typical peutz of the silkworm thoracic cuticle. In addition to encapsulation, serpin cascade melanization genes and the silkworm tyrosine hydroxylase-coding gene (*TH*, A000563), which can catalyze tyrosine to dopa and then to dopamine melanin, were upregulated from 3 hpi to 6 hpi [113]. Three hours after *Bb* oral infection, the hemolymph was substantially darker, indicating that the infection signal can be rapidly transduced to the hemolymph from the midgut and that hemolymph melanization occurs during the early stages of infection. Thus, after clotting, encapsulation of bacteria, as well as the melanotic encapsulation caused by the PPO cascade and tyrosine melanization pathway, the cuticle peutz is formed [80]. The hemolymph clots and melanin can accumulate in the chest integument and finally cause cuticle peutz on the silkworm corpse. Furthermore, we also detected 20 cuticle-protein-encoding genes that were upregulated at 24 hpi. These might be related to formation of the cuticle peutz.

*Bb* induced the silkworm systemic immune response. By real time PCR analysis, we found that *Bb*, as a Gram-positive bacterium, can induce the silkworm Toll pathway, which is similar to results from *Drosophila* [114]. Most AMPs, including those of the attacin, lebocin, enbocin, gloverin and moricin families, showed upregulation at 24 hpi. At this time, the microorganisms had passed through the midgut into the hemolymph and other organs, becoming increasingly likely to evade the host's immune responses. After the oral infection, the AMP expression pattern differed to that seen after direct injection; the latter can rapidly induce AMP upregulation in the fat body (data not shown). It remains to be determined in the silkworm whether AMPs are induced by the Toll signal pathway or the interaction with Imd and/or JAK/STAT pathway.

### Concluding Remarks

*B. bombysepticus* oral infection the host silkworm triggered a strong host response. Basal metabolic pathways were most involved after the infection, including those of genetic information processing and transcription, carbohydrate metabolism, amino acid metabolism and nitrogen metabolism, nucleotide metabolism, metabolism of cofactors and vitamins, and xenobiotic biodegradation and metabolism. Similar to *Bt*, *Bb* can induce the modulation of silkworm poisoning-related genes, such as APNs. Interestingly, the host JH synthesis, metabolism and binding-related genes showed to be upregulated after the infection. On the other hand, the silkworm immune responses, including the cellular immune response and melanization and the systemic immune response were also induced. The relationship between *Bb* and the silkworm can be used as a model to investigate pathogen-host interaction.

## Materials and Methods

### Insect Strain

The Chinese silkworm strain *Dazao* was used in this study. The silkworm was reared at a stable temperature of 25°C. The larvae stopped feeding on day 3 of the fifth instar for the infection experiments.

### Bacterial Strain

A strain of bacterium *Bb* was kindly provided by Professor Yanwen Wang (silkworm Diseases Laboratory of Shandong Agriculture University, China). This strain was separated from the corpses of silkworms that had died due to *Bb* natural infection in Daiyue district, Taian city, Shandong province, China.

### 16S rRNA PCR Amplification, Sequencing and Phylogeny

The 16S rRNA gene was PCR amplified with universal primers using the Pfu DNA polymerase according to the manufacturer's instructions (Takara). The PCR product was fully sequenced, and each nucleotide of both strands was read at least twice. Phylogenetic trees were first constructed using the NCBI database and the Blast and tree construction programs. Then, several sequences representing their species were selected for the final tree reconstruction using Mega 4.0 software. Primers used in this report were shown in Table S5.

### Silkworm Oral Infection by *Bb* Bacterium

For insects, direct bacterium injection to the body cavity is not thought to be a natural infection process. To overcome this limitation, a method of oral infection was developed [115]. In this study, oral infection method was used.

Approximately 250 day 3 of the fifth instar larvae were placed in a petri dish without food to ensure hunger before infection. Bacteria were concentrated from an overnight culture in LB medium with 100 µg/ml ampicillin to avoid contamination with other type of bacterium. Then, the bacterial pellet was washed three times with diluted water to extract the bacterial toxin before optical density (OD) assessment. About 125 grams of artificial feed and 50 ml of concentrated bacterial solution (OD_600_≈100) were thoroughly mixed in a beaker and were then cut into fine grains and given to the silkworms. After 3 hours, most of the bacterial meal had been eaten by the larvae. Then, the larvae were divided into four groups and transferred to four large petri dishes and reared with normal artificial feed. One group was raised at 25°C with approximately 70% humidity, and larvae were collected at different time intervals after infection for microarray analysis and real time PCR analysis. The other three groups were raised at 30°C with approximately 90% humidity, and dead larvae were counted at different time intervals for calculation of survival rate. For the survival rate raised under the condition of temperature of 25°C and humidity of 70%, 150 fifth instar larvae were fed with the same concentration of *Bb* as before, and raised under the condition of temperature of 25°C and humidity of 70%, and dead larvae were counted at different time intervals for calculation of survival rate. For the non-induced control, the same volume of ddH_2_O was mixed in the feed for the silkworm and the rearing conditions kept the same as *Bb* induced.

### Analysis of mRNA Expression Using Oligonucleotide Arrays

*RNA Extraction*. For infection material, three larvae were collected at different time points and snap-frozen in liquid nitrogen immediately as one sample. Three independent samples were got. After homogenation of the larvae in liquid nitrogen, the resulting powders were added to 2.0 ml centrifuge tubes (each containing approximately 0.1 g), TRIzol reagent (Invitrogen) was added and total RNA was extracted according to the manufacturer's instructions. The total RNA templates were quantified by spectrophotometry and subjected to 1.0% formaldehyde denatured agarose gel electrophoresis. Then, samples were precipitated in 100% ethanol and sent to CapitalBio Corp for microarray analysis or stored at −80°C for further analysis.

*Microarray Hybridization and Original Data Normalization*. Gene expression analysis was performed using the Affymetrix Silkworm GeneChip kit according to instructions in the Affymetrix GeneChip expression manual. The microarray hybridization and data normalization analysis were performed by CapitalBio Corp [19]. Procedures were performed as described in detail on the website of CapitalBio (http://www.capitalbio.com). Briefly, total RNA was purified using NucleoSpin® RNA clean-up kit (MACHEREY-NAGEL, Germany). Then, formaldehyde denaturing gel electrophoresis was used to detect the RNA quality. The cDNA targets were prepared from 5 µg of total RNA and were labeled with a fluorescent dye (Cy5 and Cy3-dCTP). Analyses were performed twice per sample, using a dyereversal procedure in which cDNA from the control was labeled with Cy3 and cDNA from *Bb* induced was labeled with Cy5. In the second analysis, control cDNA was labeled with Cy5 and cDNA from *Bb* induced was labeled with Cy3. This dye reversal helps to minimize error due to fluor-associated bias. Labeled cDNA were hybridized to the 23k silkworm genome oligonucleotide chip (CapitalBio), which has 22,987 oligonucleotide 70-mer probes. Chips were scanned using a Lux-Scan 10KA dual pathways laser scanner (CapitalBio), and images were analyzed by LuxScan3.0 image analysis software. At least two independent replicates were performed.

### Data Analysis

The microarray data of multiple tissues expression of day 3 of the fifth instar were downloaded from the silkworm genome database (http://www.silkdb.org/microarray/download.html) (10,393 active transcripts) [excel]. The expression data of each gene in each tissue were averaged form four to six repeats. For each gene, if its averaged expression signal was more than 400, it was considered having expression. Genes only showed expression in one tissue or more than 10 times than other tissues were considered as tissues specific genes.

The transcript values of infected larvae were subtracted from those of unchallenged control larvae to account for the development-regulated genes in the further steps. The data from the independent experiments were then averaged. Transcripts were selected when they displayed at least a 2-fold change in expression level compared with control larvae. The induced gene ontology analysis was predicted using the online molecule annotation system of CapitalBio Corp (http://www.capitalbio.com/zh-hans/support/MAS). The typical enzyme-catalyzed reactions were predicted using the online pathway relationship database KEGG (http://www.genome.jp/kegg/). All the data used reporting this report is presented in Table S2.

### Real Time PCR Confirmation of Microarray Data

Seven pairs of primers were designed to confirm microarray data. The primers sequences are listed in additional file 5. The real time RT-PCR confirmation results were performed using the SYBR Premix Ex Taq kit (TaKaRa, China) and each reaction was prepared in 25 µl containing 70 ng cDNA (2 µ1), SYBR Premix Ex Taq 12.5 µ1, 10 µM each of sense and anti-sense primers 0.5 µ1. After 40 cycles of amplification, the results were read by ABI Prism 7000 Sequence Detection System (Applied Biosystems). The real time RT-PCR was performed in duplicate for at least three biological replicates. For internal standardization primers, sw22934 (transcription initiation factor 2 gene) was used [116]. For each pair of primers, cDNA samples of four time points of induced and non-induced were performed.

### Gene Identification

Genes were identified using the proteins of SilkDB (http://silkworm.swu.edu.cn/silkdb/) to blast homologs in NCBI database (http://www.ncbi.nlm.nih.gov/) and PFAM domain database (http://pfam.sanger.ac.uk/search). E-values less than 1e-5 were used. For most silkworm immune related genes used in this study, we referenced Tanaka *et al* who identified silkworm innate immunity genes [23].

### Scanning Electron Microscopy (SEM)

Infected silkworm larvae guts were dissected into 0.9% NaCl medium. The intestinal contents were removed and the guts were washed in 0.9% NaCl medium for twice to wash off all the intestinal contents. The *Bb* bacteria were cultured in LB solid medium for 36 hours. The cleared guts and *Bb* bacteria were immediately fixed with 2.5% glutaraldehyde for 2 hours. The fixed samples were rinsed in 0.01 M phosphate buffer (pH 7.4) for 20 minutes for three times. The samples were post fixed in 1% osmium tetroxide for 2 hours and rinsed in ddH_2_O for 15 minutes for three times. Then, the fixed samples were immersed in a series of ethanol-water washes (30%, 50%, 60%, 70%, 80%, 90% and 100%) for 15 minutes per gradient and immersed in a series of tert-butyl alcohol (50%, 75% and 100%) gradient dehydration for 10 minutes per gradient and immersed in solvents of tert-butyl alcohol: acetonitrile 2∶1 and tert-butyl alcohol: acetonitrile 1∶1 for 10 minutes respectively. Finally, the samples were kept in 100% acetonitrile solvent. Samples were dried using a critical point drying apparatus and CO_2_ coated specimens with gold/palladium (60-40) using a sputter coater. Remove the samples from the dryer and attach them to SEM. The samples were observed with Hitachi S-3000N scanning electron microscope (Japan).

### Hemolymph Absorbance Detection

Three infected silkworm hemolymph were got together in 1.5 ml tube at different time points. Three samples were got for each time point. About 10 µg phenylthiourea was added into each sample tube immediately to prevent hemolymph melanization. 490 nm absorbance of hemolymph was detected using DU® 800 UV/Visible spectrophotometer (USA).

## Supporting Information

Table S1 *Bb* induced enzymes involved in general metabolism of silkworm by KEGG prediction.
(0.06 MB PDF)

Table S2 Ratios and anatation of all *Bb* induced genes.
(0.27 MB PDF)

Table S3 Multiple tissues expression data of the induced genes.
(0.17 MB PDF)

Table S4 The ratios of genes mentioned in this report.
(0.03 MB PDF)

Table S5 Primers used for cloning the 16S rRNA gene clone, real time PCR analysis.
(0.01 MB PDF)

Table S6 The microarray data and Real-Time PCR data of innate immune signaling genes.
(0.01 MB PDF)
