# Supplementary material for: A Genome-Wide Survey for Host Response of Silkworm, Bombyx mori during Pathogen Bacillus bombyseptieus Infection
Source: PLoS One. 2009 Dec 1;4(12):e8098. doi: 10.1371/journal.pone.0008098 (PMC2780328; doi:10.1371/journal.pone.0008098)
Supplement: Table S1 — Bb induced enzymes involved in general metabolism of silkworm by KEGG prediction. (0.06 MB PDF) [file pone.0008098.s001.pdf]

Table S1

***Bb* induced enzymes involved in general metabolism of silkworm by KEGG prediction**

Gene	Pvalue	Enzyme code	Enzyme	Typical Reaction
**Pyrimidine metabolism**				
BGIBMGA013054<br>BGIBMGA010234<br>BGIBMGA006292<br>BGIBMGA011753<br>BGIBMGA005739<br>BGIBMGA005469<br>BGIBMGA004913<br>BGIBMGA004994<br>BGIBMGA001898	6.00E-06	EC 2.7.7.6	RNA uridylyltransferase	UTP + RNA <=> Diphosphate + RNA
BGIBMGA010642	6.00E-06	EC 2.4.2.3	phosphate alpha-D-ribosyltransferas	Uridine + Orthophosphate <=> Uracil + alpha-D-Ribose 1-phosphate
BGIBMGA005628	6.00E-06	EC 2.4.2.10	Orotidine-5'-pphospho-alpha-D-ribosyl-transferasehosphate	Orotidine 5'-phosphate + Diphosphate <=> Orotate +5-Phospho-alpha-D-ribose 1-diphosphate
BGIBMGA001137	6.00E-06	EC 2.1.1.45	dUMP C-methyltransferase	dUMP + 5,10-Methylenetetrahydrofolate <=> Dihydrofolate + dTMP
BGIBMGA010291	6.00E-06	EC 3.1.3.5	Thymidylate 5'-phosphohydrolase	dTMP + H2O <=> Thymidine + Orthophosphate
BGIBMGA007367	6.00E-06	EC 2.7.4.6	CDP phosphotransferase	ATP + CDP <=> ADP + CTP
BGIBMGA011887	6.00E-06	EC1.3.3.1	oxygen oxidoreductase	(S)-Dihydroorotate + Oxygen <=> Orotate + H2O2
BGIBMGA003377<br>BGIBMGA006931<br>BGIBMGA009495<br>BGIBMGA005991	6.00E-06	EC 2.7.7.7	DNA deoxynucleotidyltransferase (DNA-directed)	dCTP + DNA <=> Diphosphate + DNA
BGIBMGA007005	6.00E-06	EC 3.6.1.5	CTP phosphohydrolase	CTP + H2O <=> CDP + Orthophosphate
BGIBMGA006816	6.00E-06	EC 2.1.3.2	L-aspartate carbamoyltransferase	Carbamoyl phosphate + L-Aspartate <=> Orthophosphate +N-Carbamoyl-L-aspartate
BGIBMGA005717	6.00E-06	EC 3.5.2.2	5,6-Dihydrouracil amidohydrolase	5,6-Dihydrouracil + H2O <=> 3-Ureidopropionate
**Purine metabolism**				
BGIBMGA001333	1.90E-05	EC 1.7.3.3	oxygen oxidoreductase	Urate + Oxygen + H2O <=> 5-Hydroxyisourate + H2O2
BGIBMGA002462	1.90E-05	EC 2.1.2.3	5'-phosphoribosyl-5-amino-4-imidazolecarboxamide formyltransferase	10-Formyltetrahydrofolate +1-(5'-Phosphoribosyl)-5-amino-4-imidazolecarboxamide <=> Tetrahydrofolate +1-(5'-Phosphoribosyl)-5-formamido-4-imidazolecarboxamide
BGIBMGA000807	1.90E-05	EC3.5.4.6	AMP aminohydrolase	AMP + H2O <=> IMP + NH3
BGIBMGA014227<br>BGIBMGA010637<br>BGIBMGA010177<br>BGIBMGA002654<br>BGIBMGA012772<br>BGIBMGA005168<br>BGIBMGA010715	1.90E-05	EC 3.6.1.3	ATP phosphohydrolase	ATP + H2O <=> ADP + Orthophosphate
BGIBMGA007835	1.90E-05	EC 3.6.1.15	nucleoside-triphosphatase	NTP + H2O = NDP + phosphate
BGIBMGA010377	1.90E-05	EC 1.17.1.4	NAD+ oxidoreductase	Hypoxanthine + NAD+ + H2O <=> Xanthine + NADH + H+
BGIBMGA007935	1.90E-05	EC 6.3.2.6	Ligases	ATP + 5-amino-1-(5-phospho-D-ribosyl)imidazole-4-carboxylate +L-aspartate = ADP + phosphate +(S)-2-[5-amino-1-(5-phospho-D-ribosyl)imidazole-4-carboxamido]succinate
BGIBMGA001278	1.90E-05	EC 3.5.4.4	adenosine deaminase;deoxyadenosine deaminase	adenosine + H2O = inosine + NH3
BGIBMGA006812	1.90E-05	EC 3.5.2.5	allantoinase	(S)-allantoin + H2O = allantoate
BGIBMGA010291	1.90E-05	EC 3.1.3.5	5'-ribonucleotide phosphohydrolase	a 5'-ribonucleotide + H2O = a ribonucleoside + phosphate

BGIBMGA005485	1.90E-05	EC 3.6.1.13	ADP-ribose ribophosphohydrolase	ADP-ribose + H2O = AMP + D-ribose 5-phosphate
BGIBMGA007367	1.90E-05	EC 2.7.4.6	nucleoside-diphosphate phosphotransferase	ATP + nucleoside diphosphate = ADP + nucleoside triphosphate
BGIBMGA003330	1.90E-05	EC 3.2.2.1	purine-nucleoside ribohydrolase	a purine nucleoside + H2O = D-ribose + a purine base
BGIBMGA001898<br>BGIBMGA004913<br>BGIBMGA004994<br>BGIBMGA005469<br>BGIBMGA005739<br>BGIBMGA011753<br>BGIBMGA006292<br>BGIBMGA010234<br>BGIBMGA013054	1.90E-05	EC 2.7.7.6	RNA uridylyltransferase	UTP + RNA <=> Diphosphate + RNA
BGIBMGA006931<br>BGIBMGA005991<br>BGIBMGA003377<br>BGIBMGA009495	1.90E-05	EC 2.7.7.7	DNA deoxynucleotidyltransferase (DNA-directed)	dCTP + DNA <=> Diphosphate + DNA
BGIBMGA001570	1.90E-05	EC 2.7.6.1	ribose-phosphate pyrophosphokina	ATP + D-ribose 5-phosphate = AMP + 5-phospho-α-D-ribose 1-diphosphate
BGIBMGA000060<br>BGIBMGA009962	1.90E-05	EC 3.1.4.17	3',5'-cyclic-nucleotide phosphodiest	nucleoside 3',5'-cyclic phosphate + H2O = nucleoside 5'-phosphate
BGIBMGA007005	1.90E-05	EC 3.6.1.5	diphosphoinositol-polyphosphate di	ATP + 2 H2O = AMP + 2 phosphate
BGIBMGA001068	1.90E-05	EC 4.1.1.-	lyases	a lot of
BGIBMGA010715	1.90E-05	EC 3.6.1.3	adenosinetriphosphatase	ATP + H2O = ADP + phosphate
**Pentose and glucuronate interconversions**				
BGIBMGA004965	0.001236	2.4.1.17	glucuronosyltransferase	UDP-glucuronate + acceptor = UDP + acceptor beta-D-glucuronoside
BGIBMGA005500	0.001236	EC 3.2.1.31	beta-D-glucuronoside glucuronosohydrolase	a beta-D-glucuronoside + H2O = D-glucuronate + an alcohol
BGIBMGA014453	0.001236	EC 1.1.1.21	NAD(P)+ 1-oxidoreductase	alditol + NAD(P)+ = aldose + NAD(P)H + H+
BGIBMGA007258	0.001236	EC 1.1.1.10	NADP+ 4-oxidoreductase	xylitol + NADP+ = L-xylulose + NADPH + H+
BGIBMGA006727	0.001236	EC 2.7.1.16	L(or D)-ribulose 5-phosphotransfera	ATP + L(or D)-ribulose = ADP + L(or D)-ribulose 5-phosphate
**Citrate cycle (TCA cycle)**				
BGIBMGA011412	0.006392	EC 1.1.1.41	NAD+ oxidoreductase	isocitrate + NAD+ = 2-oxoglutarate + CO2 + NADH
BGIBMGA003815	0.006392	EC 2.3.1.61	enzyme-N6-(dihydrolipoyl)lysine S-succinyltransferase	succinyl-CoA + enzyme N6-(dihydrolipoyl)lysine = CoA + enzyme N6-(S-succinyldihydrolipoyl)lysine
BGIBMGA007783<br>BGIBMGA012780	0.006392	EC 6.2.1.5	CoA ligase	ATP + succinate + CoA = ADP + phosphate + succinyl-CoA
BGIBMGA007121	0.006392	EC 4.2.1.3	citrate(isocitrate) hydro-lyase	citrate = isocitrate
BGIBMGA004276	0.006392	EC 4.1.1.32	oxaloacetate carboxy-lyase	GTP + oxaloacetate = GDP + phosphoenolpyruvate + CO2
BGIBMGA004130	0.006392	EC 1.1.1.37	NAD+ oxidoreductase	(S)-malate + NAD+ = oxaloacetate + NADH + H+
BGIBMGA012226	0.006392	EC 1.3.99.1	acceptor oxidoreductase	succinate + acceptor = fumarate + reduced acceptor
BGIBMGA008442	0.006392	EC 6.4.1.1	carbon-dioxide ligase	ATP + pyruvate + HCO3- = ADP + phosphate + oxaloacetate
BGIBMGA000672	0.006392	EC 2.3.3.1	oxaloacetate C-acetyltransferase	acetyl-CoA + H2O + oxaloacetate = citrate + CoA
**Pyruvate metabolism**				
BGIBMGA002750	0.010729	EC 1.2.4.1	Oxidoreductases	pyruvate + [dihydrolipoyllysine-residue acetyltransferase] lipoyllysine = [dihydrolipoyllysine-residue acetyltransferase] S-acetyldihydrolipoyllysine + CO2
BGIBMGA001966	0.010729	EC 1.2.1.3	NAD+ oxidoreductase	an aldehyde + NAD+ + H2O = an acid + NADH + H+
BGIBMGA006419	0.010729	EC 1.1.1.40	NADP+ oxidoreductase	(S)-malate + NADP+ = pyruvate + CO2 + NADPH
BGIBMGA004276	0.010729	EC 4.1.1.32	oxaloacetate carboxy-lyase	GTP + oxaloacetate = GDP + phosphoenolpyruvate + CO2

BGIBMGA014453	0.010729	EC 1.1.1.21	NAD(P)+ 1-oxidoreductase	alditol + NAD(P)+ = aldose + NAD(P)H + H+
BGIBMGA004130	0.010729	EC 1.1.1.37	NAD+ oxidoreductase	(S)-malate + NAD+ = oxaloacetate + NADH + H+
BGIBMGA008983	0.010729	EC 2.3.1.12	Transferases	acetyl-CoA + enzyme N6-(dihydrolipoyl)lysine = CoA + enzyme N6-(S-acetyldihydrolipoyl)lysine
BGIBMGA007508	0.010729	EC 1.1.2.3	ferricytochrome-c 2-oxidoreductase	(S)-lactate + 2 ferricytochrome c = pyruvate + 2 ferrocytochrome c + 2 H+
BGIBMGA001068	0.010729	EC 4.1.1.-	Lyases	Pyruvate + CO2 <=> 2-Hydroxyethylenedicarboxylate
BGIBMGA008442	0.010729	EC 6.4.1.1	carbon-dioxide ligase	ATP + pyruvate + HCO3- = ADP + phosphate + oxaloacetate
**Pentose phosphate pathway**				
BGIBMGA008096	0.019729	EC 1.1.1.47	NAD(P)+ 1-oxidoreductase	beta-D-glucose + NAD(P)+ = D-glucono-1,5-lactone + NAD(P)H + H+
BGIBMGA001570	0.019729	EC 2.7.6.1	D-ribose-5-phosphate diphosphotransferase	ATP + D-ribose 5-phosphate = AMP + 5-phospho-alpha-D-ribose 1-diphosphate
BGIBMGA014211<br>BGIBMGA009156	0.019729	EC 2.2.1.1	D-glyceraldehyde-3-phosphate glycolaldehydetransferase	sedoheptulose 7-phosphate + D-glyceraldehyde 3-phosphate = D-ribose 5-phosphate + D-xylulose 5-phosphate
BGIBMGA000936	0.019729	EC 5.3.1.6	D-ribose-5-phosphate aldose-ketose-isomerase	D-ribose 5-phosphate = D-ribulose 5-phosphate
BGIBMGA000926	0.019729	EC 2.2.1.2	D-glyceraldehyde-3-phosphate glyceronetransferase	sedoheptulose 7-phosphate + D-glyceraldehyde 3-phosphate = D-erythrose 4-phosphate + D-fructose 6-phosphate
BGIBMGA008478	0.019729	EC 2.7.1.15	D-ribose 5-phosphotransferase	ATP + D-ribose = ADP + D-ribose 5-phosphate
**Butanoate metabolism**				
BGIBMGA002750	0.03102	EC 1.2.4.1	acetyltransferase-lipoyllysine 2-oxidoreductase	pyruvate + [dihydrolipoyllysine-residue acetyltransferase] lipoyllysine = [dihydrolipoyllysine-residue acetyltransferase]S-acetyldihydrolipoyllysine + CO2
BGIBMGA007408<br>BGIBMGA008621<br>BGIBMGA001929	0.03102	EC 1.1.1.35	NAD+ oxidoreductase	(S)-3-hydroxyacyl-CoA + NAD+ = 3-oxoacyl-CoA + NADH + H+
BGIBMGA001966	0.03102	EC 1.2.1.3	NAD+ oxidoreductase	an aldehyde + NAD+ + H2O = an acid + NADH + H+
BGIBMGA004001	0.03102	EC 2.3.3.10	acetoacetyl-CoA C-acetyltransferas	acetyl-CoA + H2O + acetoacetyl-CoA =(S)-3-hydroxy-3-methylglutaryl-CoA + CoA
BGIBMGA004229<br>BGIBMGA008633<br>BGIBMGA014599	0.03102	EC 3.1.1.-	Hydrolases	4-Sulfolactone + HO- <=> HSO3- + 2-Maleylacetate
BGIBMGA004489	0.03102	EC 4.2.1.17	(3S)-3-hydroxyacyl-CoA hydro-lyas	(3S)-3-hydroxyacyl-CoA = trans-2(or 3)-enoyl-CoA + H2O
BGIBMGA012226	0.03102	EC 1.3.99.1	acceptor oxidoreductase	succinate + acceptor = fumarate + reduced acceptor
**Tryptophan metabolism**				
BGIBMGA007408<br>BGIBMGA008621<br>BGIBMGA001929	0.001744	EC 1.1.1.35	(S)-3-hydroxyacyl-CoA:NAD+ oxidoreductase	(S)-3-hydroxyacyl-CoA + NAD+ = 3-oxoacyl-CoA + NADH + H+
BGIBMGA001966	0.001744	EC 1.2.1.3	NAD+ oxidoreductase	an aldehyde + NAD+ + H2O = an acid + NADH + H+
BGIBMGA004776<br>BGIBMGA007842	0.001744	EC 2.1.1.-	Transferases	L-Histidine + S-Adenosyl-L-methionine <=> N(pi)-Methyl-L-histidine + S-Adenosyl-L-homocysteine
BGIBMGA005733	0.001744	EC 6.3.2.-	Ligases	ATP + 2,3-Dihydroxybenzoate <=> Diphosphate + (2,3-Dihydroxybenzoyl)adenylate
BGIBMGA001068	0.001744	EC 4.4.1.-	Lyases	S-(Indolylmethylthiohydroximoyl)-L-cysteine + H2O <=> Indolylmethylthiohydroximate + Pyruvate + NH3
BGIBMGA007424<br>BGIBMGA012866	0.001744	EC 3.5.1.4	acylamide amidohydrolase	a monocarboxylic acid amide + H2O = a monocarboxylate + NH3
BGIBMGA007146	0.001744	EC 3.7.1.3	L-kynurenine hydrolase	L-kynurenine + H2O = anthranilate + L-alanine
BGIBMGA004489	0.001744	EC 4.2.1.17	(3S)-3-hydroxyacyl-CoA hydro-lyas	(3S)-3-hydroxyacyl-CoA = trans-2(or 3)-enoyl-CoA + H2O
BGIBMGA002958	0.001744	EC 4.1.1.28	aromatic-L-amino-acid carboxy-lyase	3,4-dihydroxy-L-phenylalanine = dopamine + CO2
**Histidine metabolism**				
BGIBMGA001966	0.005648	EC 1.2.1.3	NAD+ oxidoreductase	an aldehyde + NAD+ + H2O = an acid + NADH + H+

BGIBMGA004776<br>BGIBMGA007842	0.005648	EC 2.1.1.-	Transferases	L-Histidine + S-Adenosyl-L-methionine <=><br>N(pi)-Methyl-L-histidine + S-Adenosyl-L-homocysteine
BGIBMGA002958<br>BGIBMGA003199	0.005648	EC 4.1.1.28	aromatic-L-amino-acid carboxy-lyase	3,4-dihydroxy-L-phenylalanine = dopamine + CO2
**Valine, leucine and isoleucine degradation**				
BGIBMGA007408<br>BGIBMGA008621<br>BGIBMGA001929	0.025613	EC 1.1.1.35	(S)-3-hydroxyacyl-CoA:NAD+ oxidoreductase	(S)-3-hydroxyacyl-CoA + NAD+ = 3-oxoacyl-CoA + NADH + H+
BGIBMGA001966	0.025613	EC 1.2.1.3	NAD+ oxidoreductase	an aldehyde + NAD+ + H2O = an acid + NADH + H+
BGIBMGA014181	0.025613	EC 2.3.1.16	acetyl-CoA C-acyltransferase	acyl-CoA + acetyl-CoA = CoA + 3-oxoacyl-CoA
BGIBMGA004001	0.025613	EC 2.3.3.10	acetoacetyl-CoA C-acetyltransferase	acetyl-CoA + H2O + acetoacetyl-CoA =(S)-3-hydroxy-3-methylglutaryl-CoA + CoA
BGIBMGA001237	0.025613	EC 2.6.1.42	2-oxoglutarate aminotransferase	L-leucine + 2-oxoglutarate = 4-methyl-2-oxopentanoate + L-glutamate
BGIBMGA004489	0.025613	EC 4.2.1.17	(3S)-3-hydroxyacyl-CoA hydro-lyase	(3S)-3-hydroxyacyl-CoA = trans-2(or 3)-enoyl-CoA + H2O
BGIBMGA007497	0.025613	EC 6.4.1.4	carbon-dioxide ligase	ATP + 3-methylcrotonoyl-CoA + HCO3- = ADP + phosphate +3-methylglutaconyl-CoA
**Urea cycle and metabolism of amino group**				
BGIBMGA001966	0.038333	EC 1.2.1.3	NAD+ oxidoreductase	an aldehyde + NAD+ + H2O = an acid + NADH + H+
BGIBMGA007424<br>BGIBMGA012866	0.038333	EC 3.5.1.4	acylamide amidohydrolase	a monocarboxylic acid amide + H2O = a monocarboxylate + NH3
BGIBMGA011001	0.038333	EC 3.5.1.14	N-acyl-L-amino-acid amidohydrolase	an N-acyl-L-amino acid + H2O = a carboxylate + an L-amino acid
BGIBMGA007716	0.038333	EC 3.5.3.1	L-arginine amidinohydrolase	L-arginine + H2O = L-ornithine + urea
**Aminophosphonate metabolism**				
BGIBMGA011813	0.02516	EC 2.7.7.14	ethanolamine-phosphate cytidylyltransferase	CTP + ethanolamine phosphate = diphosphate + CDP-ethanolamine
BGIBMGA004776<br>BGIBMGA007842	0.02516	EC 2.1.1.-	Transferases	L-Histidine + S-Adenosyl-L-methionine <=><br>N(pi)-Methyl-L-histidine + S-Adenosyl-L-homocysteine
**Nitrogen metabolism**				
BGIBMGA014491<br>BGIBMGA006507<br>BGIBMGA013477	0.025926	EC 1.4.1.3	NAD(P)+oxidoreductase	L-glutamate + H2O + NAD(P)+ = 2-oxoglutarate + NH3 + NAD(P)H + H+
BGIBMGA002647	0.025926	EC 4.2.1.1	carbonate hydro-lyase	H2CO3 = CO2 + H2O
BGIBMGA007111	0.025926	EC 1.4.1.13	NADP+ oxidoreductase	2 L-glutamate + NADP+ = L-glutamine + 2-oxoglutarate + NADPH + H+
BGIBMGA007025	0.025926	EC 3.5.1.1	L-asparagine amidohydrolase	L-asparagine + H2O = L-aspartate + NH3
BGIBMGA006703	0.025926	EC 6.3.1.2	ammonia ligase	ATP + L-glutamate + NH3 = ADP + phosphate + L-glutamine
**2,4-Dichlorobenzoate degradation**				
BGIBMGA004229<br>BGIBMGA008633<br>BGIBMGA014599	0.011242	EC 3.1.1.-	Hydrolases	a lot of
BGIBMGA003842	0.011242	EC 1.13.11.8	oxygen 4,5-oxidoreductase	protocatechuate + O2 = 4-carboxy-2-hydroxymuconate semialdehyde
**Benzoate degradation via hydroxylation**				
BGIBMGA014181	0.019888	EC 2.3.1.16	acyl-CoA:acetyl-CoA C-acyltransferase	acyl-CoA + acetyl-CoA = CoA + 3-oxoacyl-CoA
BGIBMGA003655	0.019888	EC 5.1.2.2	mandelate racemase	(S)-mandelate = (R)-mandelate
BGIBMGA001068	0.019888	EC 4.1.1-	Lyases	a lot of
BGIBMGA003842	0.019888	EC 1.13.11.8	oxygen 4,5-oxidoreductase	protocatechuate + O2 = 4-carboxy-2-hydroxymuconate semialdehyde
**Styrene degradation**				

BGIBMGA007424 BGIBMGA012866	0.044869	EC 3.5.1.4	acylamide amidohydrolase	a monocarboxylic acid amide + H2O = a monocarboxylate + NH3
BGIBMGA005064	0.044869	EC 5.2.1.2	4-maleylacetoacetate cis-trans-isom	4-maleylacetoacetate = 4-fumarylacetoacetate
**Porphyrin and chlorophyll metabolism**				
BGIBMGA004965	0.005202	EC 2.4.1.17	glucuronosyltransferase	UDP-glucuronate + acceptor = UDP + acceptor beta-D-glucuronoside
BGIBMGA009964	0.005202	EC 4.4.1.17	Lyases	holocytochrome c = apocytochrome c + heme
BGIBMGA000937	0.005202	EC 4.1.1.37	uroporphyrinogen-III carboxy-lyase	uroporphyrinogen III = coproporphyrinogen III + 4 CO2
BGIBMGA005500	0.005202	EC 3.2.1.31	Hydrolases	a beta-D-glucuronoside + H2O = D-glucuronate + an alcohol
BGIBMGA013998	0.005202	EC 1.14.99.3	Oxidoreductases	heme + 3 AH2 + 3 O2 = biliverdin + Fe2+ + CO + 3 A + 3 H2O
BGIBMGA007912	0.005202	EC 1.3.1.33	NADP+ 7,8-oxidoreductase	chlorophyllide a + NADP+ = protochlorophyllide + NADPH + H+
BGIBMGA003440	0.005202	EC 6.1.1.17	tRNAGlu ligase (AMP-forming)	ATP + L-glutamate + tRNAGlu = AMP + diphosphate + L-glutamyl-tRNAGlu
BGIBMGA002540	0.005202	EC 1.3.3.4	oxygen oxidoreductase	protoporphyrinogen IX + 3 O2 = protoporphyrin IX + 3 H2O2
**Pantothenate and CoA biosynthesis**				
BGIBMGA002243 BGIBMGA005508	0.010427	EC 2.7.1.24	3'-dephospho-CoA 3'-phosphotransferase	ATP + 3'-dephospho-CoA = ADP + CoA
BGIBMGA001237	0.010427	EC 2.6.1.42	2-oxoglutarate aminotransferase	L-leucine + 2-oxoglutarate = 4-methyl-2-oxopentanoate + L-glutamate
BGIBMGA001340	0.010427	EC 2.7.1.33	(R)-pantothenate 4'phosphotransferase	ATP + (R)-pantothenate = ADP + (R)-4'-phosphopantothenate
BGIBMGA005717	0.010427	EC 3.5.2.2	5,6-dihydropyrimidine amidohydrolase	5,6-dihydrouracil + H2O = 3-ureidopropanoate
**One carbon pool by folate**				
BGIBMGA004950	0.031689	EC 6.3.4.3	tetrahydrofolate ligase	ATP + formate + tetrahydrofolate = ADP + phosphate +10-formyltetrahydrofolate
BGIBMGA001137	0.031689	EC 2.1.1.45	dUMP C-methyltransferase	5,10-methylenetetrahydrofolate + dUMP = dihydrofolate + dTMP
BGIBMGA002462	0.031689	EC 2.1.2.3	Transferases	10-formyltetrahydrofolate +5-amino-1-(5-phospho-D-ribosyl)imidazole-4-carboxamide = tetrahydrofolate +5-formamido-1-(5-phospho-D-ribosyl)imidazole-4-carboxamide
