# Supplementary material for: A Genome-Wide Survey for Host Response of Silkworm, Bombyx mori during Pathogen Bacillus bombyseptieus Infection
Source: PLoS One. 2009 Dec 1;4(12):e8098. doi: 10.1371/journal.pone.0008098 (PMC2780328; doi:10.1371/journal.pone.0008098)
Supplement: Table S2 — Ratios and anatation of all Bb induced genes. (0.27 MB PDF) [file pone.0008098.s002.pdf]

Table S2

Ratios and anatation of all *Bb* induced genes

Probe	Name	3 h	6 h	12 h	24 h	E-value	Annotation
sw09154	BGIBMGA000068	0.4821	0.46355	0.76555	14.9329	Bmb025244	4.00E-60
sw06120	BGIBMGA009132	1	1.4081	1	10.268	0	gi|10242162|gb|AAG15316.1| beta tubulin [Notothenia coriiceps]
sw13662	BGIBMGA010288	1	1.3618	2.75155	9.87	2.00E-43	gi|4530427|gb|AAD22027.1| antennal-enriched UDP-glycosyltransferase [Drosophila melanogaster]
sw10407	BGIBMGA010569	1.30985	1.38105	1.21125	9.4806	1.00E-09	gi|72006769|ref|XP_782274.1| PREDICTED: similar to mitochondrial ribosomal protein L46 (predicted) [Strongylocentrotus purpuratus]
sw17791	BGIBMGA006893	1.81845	1.4161	0.64985	9.20615	No hits found	
sw19970	BGIBMGA013834	2.0471	1.67165	2.15655	8.8402	1.00E-53	gi|74229818|ref|YP_309022.1| ecdysone glucose transferase (egt) [Trichoplusia ni SNPV] ecdysone glucose transferase (egt) [Trichoplusia ni SNPV]
sw15442	BGIBMGA002979	0.8267	1.1066	1.02635	8.68885	1.00E-32	gi|72071125|ref|XP_794946.1| PREDICTED: similar to carbonyl reductase 3, partial [Strongylocentrotus purpuratus]
sw15815	BGIBMGA011495	2.1127	1.5315	0.66685	8.1517	2.00E-14	gi|20138941|sp|P82818|MOR1_BOMMO Moricin-1 precursor moricin [Bombyx mori]
sw11479	BGIBMGA010207	0.97105	1.05325	1.383	8.1504	2.00E-28	gi|18417421|ref|NP_568302.1| S-adenosylmethionine-dependent methyltransferase [Arabidopsis thaliana] unknown protein [Arabidopsis thaliana] unknown protein [Arabidopsis thaliana]
sw08325	BGIBMGA006462	0.8277	1.12195	1	7.92665	1.00E-86	gi|72014480|ref|XP_783380.1| PREDICTED: similar to Translation initiation factor eIF-2B alpha subunit (eIF-2B GDP-GTP exchange factor) [Strongylocentrotus purpuratus]
sw01773	BGIBMGA005658	0.9721	0.5052	0.37945	7.8362	5.00E-93	gi|52421205|dbj|BAD51473.1| gloverin-like protein 1 [Bombyx mori]
sw21466	BGIBMGA000837	1.5345	1.15445	1.46355	7.79595	Bmb020074	6.00E-69
sw04084	BGIBMGA006805	0.9338	1.0415	0.8619	7.77445	4.00E-58	gi|56788798|gb|AAH88474.1| WD40 protein Ciao1 (predicted) [Rattus norvegicus] WD40 protein Ciao1 (predicted) [Rattus norvegicus]
sw16997	BGIBMGA007299	0.85585	1.21805	0.9166	7.5102	2.00E-61	gi|4680679|gb|AAD27729.1| CGI-20 protein [Homo sapiens]
sw10334	BGIBMGA001496	1.0025	1.15365	1.30385	7.4953	2.00E-34	gi|58396113|ref|XP_321671.2| ENSANGP00000011708 [Anopheles gambiae str. PEST] ENSANGP00000011708 [Anopheles gambiae str. PEST]
sw04532	BGIBMGA014244	1.5091	1.6585	1.9517	7.15805	5.00E-17	gi|45550460|ref|NP_611375.2| CG15094-PA, isoform A [Drosophila melanogaster] CG15094-PA, isoform A [Drosophila melanogaster]
sw13311	BGIBMGA012038	0.82825	0.961	0.9307	7.12655	e-104	gi|32450641|gb|AAH54300.1| Osgep-prov protein [Xenopus laevis] Probable O-sialoglycoprotein endopeptidase
sw09873	BGIBMGA002492	0.86235	1.07675	0.827	7.0131	5.00E-12	gi|54638565|gb|EAL27967.1| GA18941-PA [Drosophila pseudoobscura]
sw17221	BGIBMGA007845	0.7962	1.01745	0.82795	6.5563	3.00E-23	gi|54645591|gb|EAL34329.1| GA12045-PA [Drosophila pseudoobscura]
sw10647	BGIBMGA000281	2.9171	1.47705	1.1584	6.53775	2.00E-54	gi|3608259|dbj|BAA33195.1| cuticle protein [Bombyx mori]
sw03580	BGIBMGA010930	1.31075	1.2657	1.7451	6.4873	5.00E-65	gi|87248489|gb|ABD36297.1| rRNA processing protein Ebp2 [Bombyx mori]
sw10315	BGIBMGA006200	0.90015	1.2047	0.80575	6.2053	5.00E-63	gi|3170241|gb|AAC18057.1| TU37B2 [Anopheles gambiae]
sw10771	BGIBMGA003177	1.41385	1.16195	1.2861	6.0671	4.00E-72	gi|66519665|ref|XP_393830.2| PREDICTED: similar to angio-associated migratory cell protein [Apis mellifera]
sw08579	BGIBMGA003842	0.75425	0.6444	0.7357	6.05845	9.00E-44	gi|77814698|ref|ZP_00813954.1| Catalytic LigB subunit of aromatic ring-opening dioxygenase [Shewanella putrefaciens CN-32] Catalytic LigB subunit of aromatic ring-opening dioxygenase [Shewanella putrefaciens CN-32]

sw22144	BGIBMGA004091	0.99695	1.1136	0.7859	6.0561	2.00E-16	gi|21430068|gb|AAM50712.1| GM16138p [Drosophila melanogaster] CG40127-PA.3 [Drosophila melanogaster] CG40127-PA.3 [Drosophila melanogaster]
sw06224	BGIBMGA001805	1.2051	1.03965	1.00685	6.0322	4.00E-75	gi|73997238|ref|XP_534855.2| PREDICTED: similar to mitochondrial ribosomal protein S35 [Canis familiaris]
sw12539	BGIBMGA004834	1.01355	1.2302	1.3682	6.0197	4.00E-28	gi|50740272|ref|XP_419413.1| PREDICTED: similar to CGI-115 protein [Gallus gallus]
sw16054	BGIBMGA001137	0.7686	0.87105	1.2503	5.96815	Bmb037837	2.00E-81
sw11597	BGIBMGA001131	1.0424	1.17535	1.2059	5.94735	2.00E-48	gi|71724847|gb|AAZ38885.1| 3' exoribonuclease [Xenopus laevis]
sw21303	BGIBMGA002783	1.25765	1.42015	1.5501	5.9309	No hits found	
sw01095	BGIBMGA011561	1.5206	0.6888	1.00545	5.88535	No hits found	
sw16412	BGIBMGA009964	1.12705	1.17355	1.3214	5.8593	5.00E-44	gi|54020928|ref|NP_001005708.1| holocytochrome c synthase (cytochrome c heme-lyase)
sw17034	BGIBMGA009262	0.8918	1.0836	1.1378	5.77555	5.00E-59	gi|73979520|ref|XP_849027.1| PREDICTED: similar to Deoxycytidylate deaminase (dCMP deaminase) isoform 1 [Canis familiaris]
sw14427	BGIBMGA000925	0.68505	1.00685	0.7108	5.75855	3.00E-35	gi|48139949|ref|XP_397061.1| PREDICTED: similar to ENSANGP00000014483 [Apis mellifera]
sw18369	BGIBMGA011727	2.173	1.0237	1.2144	5.7494	4.00E-05	gi|6560657|gb|AAF16706.1| putative cuticle protein [Manduca sexta]
sw03360	BGIBMGA004913	1.23895	1.39015	1.29165	5.7308	5.00E-96	gi|68401632|ref|XP_685461.1| PREDICTED: similar to RNA polymerase 1-1 isoform 1 [Danio rerio] RNA polymerase 1-1 [Danio rerio] RNA polymerase 1-1 [Danio rerio]
sw00547	BGIBMGA002335	0.66465	0.99205	0.78555	5.72835	2.00E-39	gi|66504524|ref|XP_623875.1| PREDICTED: similar to GA11196-PA [Apis mellifera]
sw20519	BGIBMGA010638	0.84235	0.96365	0.97715	5.676	1.00E-37	gi|21292306|gb|EAA04451.1| ENSANGP00000021617 [Anopheles gambiae str. PEST] ENSANGP00000021617 [Anopheles gambiae str. PEST]
sw14877	BGIBMGA000350	0.8162	1.113	0.92975	5.67565	2.00E-13	gi|89473776|gb|ABD72700.1| unknown [Acyrthosiphon pisum]
sw20283	BGIBMGA012125	0.94635	1.2979	1.28935	5.62805	8.00E-06	gi|73958242|ref|XP_848469.1| PREDICTED: similar to CG15877-PA [Canis familiaris]
sw08750	BGIBMGA010225	1.1596	1.1734	1.0893	5.58125	e-161	gi|87248557|gb|ABD36331.1| peroxisomal biogenesis factor 3 [Bombyx mori]
sw21920	BGIBMGA006855	0.94105	1.13475	0.8208	5.4299	5.00E-70	gi|37182119|gb|AAQ88862.1| DFIT212 [Homo sapiens]
sw17103	BGIBMGA001561	0.7134	0.68985	0.7184	5.21745	No hits found	
sw12094	BGIBMGA006918	1.02865	1.32225	1.35345	5.2109	No hits found	
sw16458	BGIBMGA006735	0.84335	1.04675	0.87775	5.1433	Bmb041868	2.00E-47
sw03972	BGIBMGA008513	1.31615	1	0.9673	5.1106	No hits found	
sw06396	BGIBMGA006806	0.97785	1.0539	1.00415	5.104	1.00E-67	gi|40215783|gb|AAL39701.2| LD28566p [Drosophila melanogaster]
sw03300	BGIBMGA013243	0.72195	0.91495	0.85715	5.07955	1.00E-22	gi|76643547|ref|XP_875018.1| PREDICTED: similar to Mitochondrial import inner membrane translocase subunit Tim22 isoform 2 [Bos taurus]
sw05146	BGIBMGA002439	1.2721	1.37505	1.5179	5.04485	e-107	gi|66556699|ref|XP_624203.1| PREDICTED: similar to GA11422-PA, partial [Apis mellifera]
sw01772	BGIBMGA000464	1.1085	1.0477	1.04175	5.0383	1.00E-69	gi|55237266|gb|EAA12840.2| ENSANGP00000019242 [Anopheles gambiae str. PEST] ENSANGP00000019242 [Anopheles gambiae str. PEST]
sw15455	BGIBMGA011999	0.7669	0.847	0.5541	4.9469	8.00E-15	gi|53734221|gb|AAH83655.1| Mitochondrial ATP synthase regulatory component factor B [Rattus norvegicus] mitochondrial ATP synthase regulatory component factor B
sw22332	BGIBMGA004847	0.65875	0.9139	0.99995	4.9277	e-120	gi|47086987|ref|NP_998500.1| SEC13-like 1 [Danio rerio] SEC13-like 1 [Danio rerio]

<table>
<tr><td>sw22347</td><td>BGIBMGA012999</td><td>1.8581</td><td>2.6587</td><td>4.22965</td><td>4.9108</td><td>e-122</td><td>gi|48094603|ref|XP_394220.1| PREDICTED: similar to CG9518-PA [Apis mellifera]</td></tr>
<tr><td>sw06780</td><td>BGIBMGA001507</td><td>1.3057</td><td>2.0168</td><td>2.0887</td><td>4.89775</td><td>Bmb034436</td><td>2.00E-54</td></tr>
<tr><td>sw21623</td><td>BGIBMGA006899</td><td>0.9358</td><td>1.0279</td><td>0.9017</td><td>4.8919</td><td>Bmb044274</td><td>9.00E-12</td></tr>
<tr><td>sw18085</td><td>BGIBMGA012780</td><td>0.9419</td><td>1.09045</td><td>1.29165</td><td>4.88875</td><td>e-114</td><td>gi|73980931|ref|XP_532985.2| PREDICTED: similar to succinate-CoA ligase, GDP-forming, alpha subunit [Canis familiaris]</td></tr>
<tr><td>sw21727</td><td>BGIBMGA007987</td><td>1.1756</td><td>1.15925</td><td>1.0606</td><td>4.88565</td><td>8.00E-87</td><td>gi|13359092|dbj|BAB33295.1| Bacteriophage T7 lysozyme-like protein 2 (BTL-LP2) [Bombyx mori]</td></tr>
<tr><td>sw13022</td><td>BGIBMGA002880</td><td>0.814</td><td>1.10085</td><td>0.70235</td><td>4.87635</td><td>No hits found</td><td></td></tr>
<tr><td>sw14285</td><td>BGIBMGA013625</td><td>1.15125</td><td>1.01765</td><td>1.01745</td><td>4.84265</td><td>9.00E-22</td><td>gi|73967373|ref|XP_854028.1| PREDICTED: similar to candidate tumor suppressor in ovarian cancer 2 [Canis familiaris]</td></tr>
<tr><td>sw21881</td><td>BGIBMGA014064</td><td>1.16355</td><td>1.7853</td><td>1.11005</td><td>4.82495</td><td>4.00E-40</td><td>gi|50418445|gb|AAH77670.1| MGC89796 protein [Xenopus tropicalis] MGC89796 protein [Xenopus tropicalis] Novel protein similar to hypothetcal protein LOC56902 [Xenopus tropicalis]</td></tr>
<tr><td>sw05948</td><td>BGIBMGA005489</td><td>2.23905</td><td>1.84965</td><td>1.3703</td><td>4.8233</td><td>7.00E-39</td><td>gi|56790262|ref|NP_571369.1| superoxide dismutase 1, soluble [Danio rerio] Cu/Zn-superoxide dismutase [Danio rerio] Superoxide dismutase 1, soluble [Danio rerio] PREDICTED: similar to Cu/Zn-superoxide dismutase</td></tr>
<tr><td>sw07313</td><td>BGIBMGA010376</td><td>0.7587</td><td>0.91525</td><td>0.85165</td><td>4.8185</td><td>8.00E-35</td><td>gi|2815410|dbj|BAA24526.1| similar to Drosophila l(3)S12 [Bombyx mori]</td></tr>
<tr><td>sw09785</td><td>BGIBMGA010318</td><td>1.312</td><td>1.33425</td><td>1.29985</td><td>4.77525</td><td>Bmb035915</td><td>1.00E-91</td></tr>
<tr><td>sw07058</td><td>BGIBMGA011328</td><td>0.97915</td><td>1.0938</td><td>0.8651</td><td>4.74945</td><td>Bmb041739</td><td>3.00E-10</td></tr>
<tr><td>sw12525</td><td>BGIBMGA010309</td><td>0.8526</td><td>1.08895</td><td>1.13425</td><td>4.749</td><td>1.00E-36</td><td>gi|49904020|gb|AAH76688.1| MGC79718 protein [Xenopus tropicalis] MGC79718 protein [Xenopus tropicalis]</td></tr>
<tr><td>sw09702</td><td>BGIBMGA003155</td><td>1.0923</td><td>1.0193</td><td>0.9663</td><td>4.73675</td><td>1.00E-24</td><td>gi|54035102|gb|AAH84076.1| LOC494992 protein [Xenopus laevis]</td></tr>
<tr><td>sw01217</td><td>BGIBMGA009305</td><td>1</td><td>2.2798</td><td>1</td><td>4.7284</td><td>No hits found</td><td></td></tr>
<tr><td>sw10048</td><td>BGIBMGA009486</td><td>0.77315</td><td>2.4063</td><td>1.58695</td><td>4.7274</td><td>Bmb041345</td><td>e-119</td></tr>
<tr><td>sw15650</td><td>BGIBMGA000330</td><td>2.37955</td><td>0.8064</td><td>1.8387</td><td>4.71715</td><td>No hits found</td><td></td></tr>
<tr><td>sw14758</td><td>BGIBMGA007642</td><td>0.9575</td><td>1.17845</td><td>1.0088</td><td>4.71</td><td>1.00E-19</td><td>gi|15718247|emb|CAA83138.2| Hypothetical protein T16H12.5a [Caenorhabditis elegans] BTB and MATH domain containing family member (bath-43) [Caenorhabditis elegans] Hypothetical protein T16H12.5 in chromosome III</td></tr>
<tr><td>sw10047</td><td>BGIBMGA008110</td><td>6.8849</td><td>1</td><td>1</td><td>4.67525</td><td>Bmb041294</td><td>No hits found</td></tr>
<tr><td>sw05369</td><td>BGIBMGA008620</td><td>1.08005</td><td>1.0117</td><td>1.0926</td><td>4.6519</td><td>2.00E-51</td><td>gi|87248529|gb|ABD36317.1| mitochondria-associated granulocyte macrophage CSF signaling molecule [Bombyx mori]</td></tr>
<tr><td>sw02921</td><td>BGIBMGA000800</td><td>0.74475</td><td>0.9466</td><td>0.8724</td><td>4.64455</td><td>7.00E-43</td><td>gi|76641811|ref|XP_616687.2| PREDICTED: similar to Clathrin coat assembly protein AP17 (Clathrin coat associated protein AP17) (Plasma membrane adaptor AP-2 17 kDa protein)</td></tr>
<tr><td>sw12746</td><td>BGIBMGA011007</td><td>0.67415</td><td>0.93555</td><td>0.66105</td><td>4.62415</td><td>3.00E-55</td><td>gi|84781676|ref|NP_001034092.1| 5-methyltetrahydrofolate-homocysteine methyltransferase reductase [Rattus norvegicus] Mtrr protein [Rattus norvegicus]</td></tr>
<tr><td>sw17264</td><td>BGIBMGA003520</td><td>0.9035</td><td>1.08285</td><td>0.899</td><td>4.6146</td><td>6.00E-08</td><td>gi|52346150|ref|NP_001005117.1| novel protein similar to mitochondrial ribosomal protein L53 mrpl53 [Xenopus tropicalis] Novel protein similar to mitochondrial ribosomal protein L53 mrpl53</td></tr>
<tr><td>sw16046</td><td>BGIBMGA011324</td><td>0.83605</td><td>1.01135</td><td>0.9949</td><td>4.6096</td><td>Bmb037794</td><td>8.00E-07</td></tr>
<tr><td>sw22880</td><td>BGIBMGA010437</td><td>1.1852</td><td>1.17225</td><td>1.06705</td><td>4.60875</td><td>8.00E-62</td><td>gi|72006802|ref|XP_783718.1| PREDICTED: similar to beta-tubulin cofactor C [Strongylocentrotus purpuratus]</td></tr>
<tr><td>sw17911</td><td>BGIBMGA011592</td><td>1.0353</td><td>1.0663</td><td>1.18465</td><td>4.5845</td><td>2.00E-55</td><td>gi|56270497|gb|AAH87528.1| LOC496097 protein [Xenopus laevis]</td></tr>
<tr><td>sw09105</td><td>BGIBMGA009395</td><td>1.15365</td><td>1.3337</td><td>1.70365</td><td>4.55455</td><td>1.00E-49</td><td>gi|51480426|gb|AAH80252.1| WD repeat domain 18 [Danio rerio] WD repeat domain 18 [Danio rerio]</td></tr>
<tr><td>sw07146</td><td>BGIBMGA007739</td><td>0.8679</td><td>0.94455</td><td>1.1636</td><td>4.54955</td><td>5.00E-07</td><td>gi|72091540|ref|XP_791287.1| PREDICTED: similar to butyrate-induced transcript 1 like (19.4 kD) (3G364) [Strongylocentrotus purpuratus]</td></tr>
</table>

sw13530	BGIBMGA006177	0.718	0.81695	0.66475	4.4744	2.00E-22	gi|6684113|gb|AAF23489.1| 312 protein [Drosophila melanogaster]
sw15089	BGIBMGA003987	0.78365	0.94545	0.9424	4.46545	7.00E-64	gi|47523746|ref|NP_999508.1| quinoid dihydropteridine reductase [Sus scrofa] quinoid dihydropteridine reductase [Sus scrofa] Dihydropteridine reductase (HDHPR) (Quinoid dihydropteridine reductase)
sw10444	BGIBMGA010151	0.8559	1.06235	0.876	4.465	1.00E-33	gi|57089809|ref|XP_537340.1| PREDICTED: similar to tumor necrosis factor superfamily, member 5-induced protein 1 [Canis familiaris]
sw08478	BGIBMGA005882	0.5603	0.83685	0.86265	4.45825	2.00E-28	gi|54645763|gb|EAL34501.1| GA12251-PA [Drosophila pseudoobscura]
sw04014	BGIBMGA005693	1	2.0388	1.71045	4.45345	No hits found	
sw10815	BGIBMGA003838	0.97085	1.018	0.67025	4.44385	2.00E-19	gi|73969379|ref|XP_531710.2| PREDICTED: similar to serine hydrolase-like 2 [Canis familiaris]
sw16472	BGIBMGA010577	1.323	1.2836	1.13815	4.4428	Bmb042047	1.00E-13
sw17152	BGIBMGA009118	0.72305	1.0022	0.9122	4.42585	5.00E-56	gi|57032918|gb|AAH88818.1| LOC496302 protein [Xenopus laevis]
sw07768	BGIBMGA007559	0.7547	0.83265	0.75115	4.396	6.00E-32	gi|66556167|ref|XP_624817.1| PREDICTED: similar to Ribosomal protein S29 [Apis mellifera]
sw18132	BGIBMGA006966	0.896	1.10025	0.8674	4.36665	9.00E-32	gi|48104167|ref|XP_392921.1| PREDICTED: similar to nuclear transport factor 2 [Apis mellifera]
sw16002	BGIBMGA002835	1.33445	1.1532	1.05165	4.3348	3.00E-66	gi|72010073|ref|XP_786273.1| PREDICTED: similar to claudin 12 [Strongylocentrotus purpuratus]
sw19410	BGIBMGA001027	0.89605	1.2749	1.09135	4.3253	6.00E-41	gi|57905728|ref|XP_551123.1| ENSANGP00000027831 [Anopheles gambiae str. PEST] ENSANGP00000027831 [Anopheles gambiae str. PEST]
sw10082	BGIBMGA009068	0.823	1.02165	0.83465	4.31995	No hits found	
sw11463	BGIBMGA007233	1.02	1.0915	1.20605	4.3172	No hits found	
sw13390	BGIBMGA007367	1.11365	1.22515	0.861	4.30325	2.00E-41	gi|12804745|gb|AAH01808.1| Nucleoside diphosphate kinase type 6 [Homo sapiens] Nucleoside diphosphate kinase type 6 [Homo sapiens] nucleoside diphosphate kinase type 6
sw18512	BGIBMGA005162	0.83115	0.9651	1.1618	4.2965	2.00E-23	gi|62083501|gb|AAX62475.1| mitochondrial ribosomal protein L11 [Lysiphlebus testaceipes]
sw07841	BGIBMGA010651	1.38435	1.49075	1.3233	4.2944	2.00E-27	gi|52789335|gb|AAH83014.1| LOC494842 protein [Xenopus laevis]
sw22048	BGIBMGA013200	0.7698	1.0694	1.07635	4.27535	3.00E-68	gi|66552883|ref|XP_625026.1| PREDICTED: similar to ENSANGP00000012063 [Apis mellifera]
sw20415	BGIBMGA012624	1.52565	1.11905	1.34265	4.272	2.00E-91	gi|62286614|sp|Q60HC5|DHC24_MACFA 24-dehydrocholesterol reductase precursor (3-beta-hydroxysterol delta-24-reductase)
sw17940	BGIBMGA006931	1.2198	1.31025	1.2858	4.269	No hits found	
sw13056	BGIBMGA011645	1.1104	1.28025	1.39555	4.2538	2.00E-17	gi|22297524|gb|AAK32113.2| Mpv17-like protein [Mus musculus] Mpv17 transgene, kidney disease mutant-like [Mus musculus] Mpv17l protein [Mus musculus]
sw13666	BGIBMGA012460	0.91845	1.1253	0.998	4.2367	No hits found	
sw17771	BGIBMGA003095	2.5833	1.3329	1.97195	4.23325	2.00E-18	gi|290935|gb|AAC37204.1| cuticle protein 66 Larval/pupal rigid cuticle protein 66 precursor (HCCP66)
sw04275	BGIBMGA010208	0.85085	0.865	1.23865	4.21965	No hits found	
sw09102	BGIBMGA009901	1.24245	1.1374	1.58715	4.21515	5.00E-29	gi|87248537|gb|ABD36321.1| mitochondrial matrix protein p33 [Bombyx mori]
sw08920	BGIBMGA009051	0.73615	0.93465	0.74585	4.21495	1.00E-22	gi|72061380|ref|XP_789080.1| PREDICTED: similar to disulfide isomerase [Strongylocentrotus purpuratus]
sw06189	BGIBMGA011234	1.00925	1.0255	1.1417	4.20425	No hits found	
sw08778	BGIBMGA010977	1.28135	1.26905	0.94345	4.1625	7.00E-12	gi|409430|gb|AAA29312.1| ecdysteroid regulated protein

sw13706	BGIBMGA010819	0.90285	1.0661	0.50775	4.15685	2.00E-44	gi|72022689|ref|XP_795397.1| PREDICTED: similar to Mitochondrial 28S ribosomal protein S25 (S25mt) (MRP-S25) [Strongylocentrotus purpuratus]
sw11888	BGIBMGA009983	1.58655	1.48875	1.52385	4.1394	e-100	gi|30580383|sp|Q9VZE6|BXDC2_DROME Brix domain-containing protein 2 homolog (Ribosome biogenesis protein Brix homolog)
sw01903	BGIBMGA006154	0.6878	0.9907	0.79025	4.12635	2.00E-35	gi|87248405|gb|ABD36255.1| suppressor of Lec15 glycosylation mutation-like protein [Bombyx mori]
sw00830	BGIBMGA002078	0.88505	1.1494	0.8921	4.12245	1.00E-18	gi|55632157|ref|XP_520201.1| PREDICTED: similar to haloacid dehalogenase-like hydrolase domain containing 3; chromosome 9 open reading frame 158 [Pan troglodytes]
sw18036	BGIBMGA003762	0.7267	0.9089	0.9568	4.11825	1.00E-42	gi|14603259|gb|AAH10089.1| ASMTL protein [Homo sapiens]
sw09281	BGIBMGA007741	0.86445	1.0596	0.88165	4.1066	6.00E-23	gi|55238957|gb|EAA11211.2| ENSANGP00000017823 [Anopheles gambiae str. PEST] ENSANGP00000017823 [Anopheles gambiae str. PEST]
sw18363	BGIBMGA011884	0.88905	1.073	0.9493	4.0937	5.00E-37	gi|27819763|gb|AAL68373.2| SD01117p [Drosophila melanogaster]
sw05953	BGIBMGA007258	1.1382	1.11185	1.11955	4.09205	5.00E-71	gi|48139441|ref|XP_397008.1| PREDICTED: similar to Diacetyl/L-xylulose reductase [Apis mellifera]
sw05099	BGIBMGA012981	0.5653	0.92815	0.74575	4.07675	3.00E-65	gi|89273430|emb|CAJ82650.1| transmembrane emp24 domain trafficking protein 2 [Xenopus tropicalis]
sw11923	BGIBMGA009834	0.80675	1.50395	1.33015	4.0751	No hits found	
sw14629	BGIBMGA004075	4.6135	2.34935	1.9541	4.0746	No hits found	
sw03192	BGIBMGA008176	0.76595	0.74295	0.61635	4.07235	3.00E-17	gi|400673|sp|P31420|OMBP_MANSE Ommochrome-binding protein precursor (OBP) (YCP) ommochrome-binding protein
sw09013	BGIBMGA013029	0.8649	0.88355	0.9334	4.071	2.00E-05	gi|4733885|gb|AAD28638.1| unknown [Drosophila melanogaster]
sw00926	BGIBMGA005991	1.2557	1.1018	1.15155	4.0685	2.00E-36	gi|73973167|ref|XP_532185.2| PREDICTED: similar to DNA primase large subunit, 58kDa isoform 1 [Canis familiaris]
sw17483	BGIBMGA010061	2.1242	1.42655	1.43805	4.0534	2.00E-64	gi|6690636|gb|AAF24228.1| trypsin-like PiT2c precursor [Plodia interpunctella]
sw06001	BGIBMGA002215	0.91935	0.9811	1.30515	4.05285	7.00E-42	gi|72006105|ref|XP_783220.1| PREDICTED: similar to Mitochondrial import inner membrane translocase subunit Tim17 B (JM3) [Strongylocentrotus purpuratus]
sw01600	BGIBMGA003524	0.9754	0.81525	0.7923	4.0406	No hits found	
sw17903	BGIBMGA009888	1.1189	0.96665	1.178	4.0295	5.00E-60	gi|61583224|gb|AAX47004.1| transformer-2 protein F [Bombyx mori]
sw04267	BGIBMGA008689	1.4616	1.25695	1.34655	4.0281	Bmb030313	9.00E-10
sw12191	BGIBMGA006919	0.99875	1.01995	1.09275	4.0259	Bmb010764	4.00E-65
sw13139	BGIBMGA003537	0.58995	0.9654	0.586	4.02295	1.00E-25	gi|72015284|ref|XP_782825.1| PREDICTED: similar to putative protein, with a coiled coil-4 domain, of ancient origin (2H170) [Strongylocentrotus purpuratus]
sw17449	BGIBMGA003475	0.82175	1.35645	1.08945	4.0224	e-102	gi|12328436|dbj|BAB21109.1| elongation factor 1 delta [Bombyx mori]
sw11688	BGIBMGA008813	1.23705	1.08965	1.33775	4.0178	4.00E-26	gi|49532884|dbj|BAD26677.1| Juvenile hormone diol kinase [Plutella xylostella]
sw20069	BGIBMGA000924	0.91415	1.0906	1.0202	4.013	2.00E-87	gi|87248355|gb|ABD36230.1| ribosomal protein L22 [Bombyx mori]
sw12176	BGIBMGA008312	0.80895	0.75175	0.999	4.00105	e-119	gi|87248567|gb|ABD36336.1| mitochondrial prohibitin complex protein 2 [Bombyx mori]
sw01486	BGIBMGA013268	0.74455	1.0512	0.8324	3.99825	5.00E-07	gi|66499360|ref|XP_396507.2| PREDICTED: similar to CG11550-PA [Apis mellifera]
sw11663	BGIBMGA001222	0.9246	1.0078	0.81735	3.98315	2.00E-15	gi|49522624|gb|AAH75581.1| MGC89563 protein [Xenopus tropicalis] MGC89563 protein [Xenopus tropicalis]
sw16038	BGIBMGA010118	0.98005	1.1465	1.2156	3.98165	4.00E-17	gi|55246170|gb|EAL41993.1| ENSANGP00000027810 [Anopheles gambiae str. PEST] ENSANGP00000027810 [Anopheles gambiae str. PEST]

sw08598	BGIBMGA007010	0.80945	1.2399	0.71395	3.9806	1.00E-15	gi|49257982|gb|AAH74167.1| MGC81952 protein [Xenopus laevis] Charged multivesicular body protein 6-1 (Chromatin modifying protein 6-1)
sw03808	BGIBMGA005319	0.5746	0.95895	0.92705	3.95505	2.00E-44	gi|27501446|ref|NP_003668.2| density-regulated protein [Homo sapiens] DENR protein [Homo sapiens] Density-regulated protein (DRP) (DRP1 protein) (Smooth muscle cell associated protein 3) (SMAP-3) SMAP-3 [Homo sapiens]
sw07050	BGIBMGA006662	1.2035	1.0268	1.35605	3.942	4.00E-07	gi|510500|emb|CAA50796.1| GCR 1 protein [Drosophila melanogaster]
sw08933	BGIBMGA013364	0.8714	1.1236	1.14995	3.9358	4.00E-60	gi|6671690|ref|NP_031646.1| carbonyl reductase 1 [Mus musculus] Carbonyl reductase [NADPH] 1 (NADPH-dependent carbonyl reductase 1) carbonyl reductase
sw17045	BGIBMGA000375	1.0199	1.2083	1.0551	3.9354	2.00E-35	gi|80478497|gb|AAI09352.1| Dihydrouridine synthase 4-like [Mus musculus]
sw08947	BGIBMGA004112	0.7442	0.91845	0.69535	3.93305	Bmb022039	e-146
sw15293	BGIBMGA007878	1.0656	1.2752	0.9476	3.9297	Bmb031647	5.00E-13
sw06899	BGIBMGA009307	0.79465	1.0541	0.8801	3.9246	4.00E-78	gi|62860202|ref|NP_001016646.1| thioredoxin-like 4A [Xenopus tropicalis] thioredoxin-like 4A [Xenopus tropicalis]
sw16033	BGIBMGA014453	1.93	1.14875	0.78355	3.923	6.00E-84	gi|62529862|gb|AAX85203.1| aldo/keto reductase [Aedes aegypti]
sw15849	BGIBMGA008868	1.21075	1.1895	1.0667	3.91485	Bmb035884	1.00E-19
sw09825	BGIBMGA008699	1.036	1.1849	1.0907	3.9112	1.00E-76	gi|56270488|gb|AAH87491.1| LOC496075 protein [Xenopus laevis]
sw20745	BGIBMGA000364	0.9692	1.0694	0.986	3.9094	7.00E-43	gi|72086794|ref|XP_793560.1| PREDICTED: similar to lactation elevated 1, partial [Strongylocentrotus purpuratus]
sw22684	BGIBMGA000276	2.0627	1.6147	1	3.90655	7.00E-50	gi|3608259|dbj|BAA33195.1| cuticle protein [Bombyx mori]
sw17005	BGIBMGA006242	0.69705	0.9564	0.9037	3.8852	1.00E-35	gi|24647481|ref|NP_650561.1| CG14903-PA [Drosophila melanogaster] CG14903-PA [Drosophila melanogaster]
sw16983	BGIBMGA006984	0.78	0.9604	0.8421	3.8827	2.00E-83	gi|87248133|gb|ABD36119.1| G10 protein [Bombyx mori]
sw14681	BGIBMGA006292	0.9122	1.22015	1.0464	3.88025	7.00E-67	gi|89521458|gb|ABD76573.1| transcription-associated zinc ribbon protein [Bombyx mori]
sw00338	BGIBMGA005391	0.88295	1.0498	0.9083	3.8798	7.00E-22	gi|73586862|gb|AAI03209.1| Pyridoxine 5'-phosphate oxidase [Bos taurus] pyridoxine 5'-phosphate oxidase [Bos taurus] Pyridoxine-5'-phosphate oxidase (Pyridoxamine-phosphate oxidase)
sw09145	BGIBMGA013011	0.8424	1.26215	0.96275	3.86375	No hits found	
sw21775	BGIBMGA005079	1.03775	1.21875	0.99545	3.8513	No hits found	
sw07663	BGIBMGA006799	1.1023	1.2427	1.6249	3.83725	7.00E-30	gi|54641496|gb|EAL30246.1| GA10532-PA [Drosophila pseudoobscura]
sw13347	BGIBMGA004278	1.07305	1.0967	1.0178	3.837	9.00E-18	gi|18203570|sp|Q9WTQ8|TIM23_MOUSE Mitochondrial import inner membrane translocase subunit Tim23 translocase of inner mitochondrial membrane 23 homolog
sw14472	BGIBMGA002755	0.7449	0.94925	0.8946	3.83025	5.00E-73	gi|62857721|ref|NP_001016870.1| SUMO-1 activating enzyme subunit 1 [Xenopus tropicalis] SMT3 suppressor of mif two 3 homolog 1 (yeast) [Xenopus tropicalis]
sw05605	BGIBMGA001623	0.9114	1.05535	0.9138	3.82895	1.00E-13	gi|23508790|ref|NP_701458.1| Zinc finger transcription factor (krox1) [Plasmodium falciparum 3D7] Krox-like protein [Plasmodium falciparum 3D7] Zinc finger transcription factor (krox1) [Plasmodium falciparum 3D7]
sw03347	BGIBMGA005636	1.05755	1.03405	1.2643	3.8256	8.00E-88	gi|14603167|gb|AAH10048.1| RNA, U3 small nucleolar interacting protein 2 [Homo sapiens] RNA, U3 small nucleolar interacting protein 2 [Homo sapiens] RNA, U3 small nucleolar interacting protein 2
sw09479	BGIBMGA011469	0.68955	0.79465	0.7999	3.8204	2.00E-24	gi|76628923|ref|XP_588837.2| PREDICTED: similar to chromosome 2 open reading frame 7 [Bos taurus]
sw05879	BGIBMGA005208	1.4507	0.8478	0.8166	3.8173	6.00E-26	gi|76630146|ref|XP_582319.2| PREDICTED: similar to retinol dehydrogenase 14 (all-trans and 9-cis) [Bos taurus]
sw15610	BGIBMGA006376	0.6895	0.76355	0.87075	3.8069	9.00E-16	gi|1362727|pir||B56211 progesterone receptor-related protein p23 - chicken
sw01677	BGIBMGA011771	0.6309	0.759	0.9597	3.80325	8.00E-28	gi|47225269|emb|CAG09769.1| unnamed protein product [Tetraodon nigroviridis]

sw18010	BGIBMGA012134	1.18375	1.35815	1.2874	3.80225	6.00E-14	gi|27374388|gb|AAO01125.1| CG13030-PA [Drosophila willistoni]
sw13521	BGIBMGA011070	0.8927	1.1285	0.9746	3.8009	3.00E-41	gi|45360855|ref|NP_989103.1| Nif3l1bp1-prov protein [Xenopus tropicalis] Nif3l1bp1-prov protein [Xenopus tropicalis]
sw18194	BGIBMGA009614	1.3675	1.2788	0.9787	3.79655	2.00E-35	gi|87248347|gb|ABD36226.1| lysosomal thiol reductase IP30 precursor isoform 1 [Bombyx mori]
sw06046	BGIBMGA010845	0.8194	1.1804	0.94285	3.79605	1.00E-20	gi|48140447|ref|XP_397115.1| PREDICTED: similar to ENSANGP00000014264 [Apis mellifera]
sw12429	BGIBMGA006771	0.6558	0.781	0.8036	3.7781	2.00E-46	gi|50759473|ref|XP_417658.1| PREDICTED: similar to peptidyl prolyl isomerase H; cyclophilin H; rotamase H [Gallus gallus]
sw11486	BGIBMGA000412	0.7044	0.97255	1.03	3.7749	7.00E-43	gi|12653357|gb|AAH00448.1| THUMP domain containing 1 [Homo sapiens] THUMP domain containing 1 [Homo sapiens] THUMP domain-containing protein 1
sw14453	BGIBMGA011281	0.8466	1.1639	0.7589	3.7645	Bmb025362	6.00E-18
sw13670	BGIBMGA011532	0.9167	1.142	1.2154	3.75325	2.00E-52	gi|82999323|ref|XP_920157.1| PREDICTED: similar to Protein FRA10AC1 homolog [Mus musculus]
sw07451	BGIBMGA011737	0.8821	0.9949	1.00635	3.73595	2.00E-23	gi|47938836|gb|AAH71528.1| General transcription factor IIA, 1, 19/37kDa [Danio rerio]
sw15319	BGIBMGA013759	0.918	1.03985	0.856	3.73525	No hits found	
sw13384	BGIBMGA012571	0.52445	0.9904	0.65455	3.73465	3.00E-31	gi|1016766|gb|AAA79181.1| signal recognition particle 19 kDa protein
sw18770	BGIBMGA012376	0.95855	1.00605	1.0005	3.71535	9.00E-07	gi|39591361|emb|CAE73415.1| Hypothetical protein CBG20858 [Caenorhabditis briggsae]
sw15870	BGIBMGA006100	0.65725	0.9475	0.7754	3.7095	Bmb036020	3.00E-35
sw09660	BGIBMGA004919	1.03815	1.1799	0.9264	3.7032	7.00E-78	gi|72006159|ref|XP_785239.1| PREDICTED: similar to bisphosphate nucleotidase 1 [Strongylocentrotus purpuratus]
sw12216	BGIBMGA012075	0.78255	1.05675	1.0026	3.70265	3.00E-36	gi|7542478|gb|AAF63472.1| H protein [Oryctolagus cuniculus] Glycine cleavage system H protein, mitochondrial precursor
sw01819	BGIBMGA012772	0.80015	0.8565	1.19915	3.69495	0	gi|73986746|ref|XP_533895.2| PREDICTED: similar to DEAD (Asp-Glu-Ala-Asp) box polypeptide 39 isoform 1 [Canis familiaris]
sw14711	BGIBMGA007955	1.32535	1.44	1.63805	3.6877	No hits found	
sw09767	BGIBMGA012459	0.7214	0.9415	0.5915	3.6876	2.00E-42	gi|58569313|gb|AAW79027.1| GekBS181P [Gekko japonicus]
sw04603	BGIBMGA003390	0.7291	0.9446	0.9034	3.675	2.00E-40	gi|85720047|gb|ABC75592.1| N-acetyltransferase 9 [Ictalurus punctatus]
sw06731	BGIBMGA004099	0.86205	1.03945	0.7686	3.6723	Bmb033309	1.00E-34
sw03726	BGIBMGA007259	0.8022	1.08195	1.0462	3.6712	2.00E-44	gi|48139441|ref|XP_397008.1| PREDICTED: similar to Diacetyl/L-xylulose reductase [Apis mellifera]
sw15217	BGIBMGA012934	0.99465	1.24035	1.1165	3.6656	9.00E-74	gi|41388936|gb|AAH65899.1| Actin related protein 2/3 complex subunit 4 [Danio rerio] actin related protein 2/3 complex, subunit 4, like [Danio rerio] Actin related protein 2/3 complex, subunit 4, like
sw16492	BGIBMGA010768	1.6068	2.23365	1.84785	3.66425	Bmb042252	2.00E-07
sw01325	BGIBMGA011759	2.7865	1.66795	1.26465	3.6627	4.00E-35	gi|28261395|gb|AAO32819.1| putative cuticle protein [Manduca sexta] putative cuticle protein [Manduca sexta]
sw04436	BGIBMGA001197	1.10135	1.1996	1.07385	3.6625	1.00E-23	gi|72013764|ref|XP_784928.1| PREDICTED: similar to Translation initiation factor eIF-2B epsilon subunit (eIF-2B GDP-GTP exchange factor) [Strongylocentrotus purpuratus]
sw11296	BGIBMGA009849	1.51205	1.1164	1.4853	3.6599	e-107	gi|7688192|emb|CAB89811.1| RNA 3'-terminal phosphate cyclase-like protein [Homo sapiens]
sw18237	BGIBMGA005557	0.72085	1.2755	1.35295	3.64915	1.00E-60	gi|87248267|gb|ABD36186.1| ATP binding protein [Bombyx mori]
sw01709	BGIBMGA010387	0.74205	1.03645	0.91	3.64905	9.00E-84	gi|5815357|gb|AAD52652.1| J domain containing protein [Bombyx mori] J domain containing protein

sw04647	BGIBMGA004933	0.751	0.97595	0.7222	3.64715	5.00E-06	gi|12653931|gb|AAH00759.1| NTF2-like export factor 1 [Homo sapiens] NTF2-like export factor 1 [Homo sapiens] NTF2-like export factor 1 [Homo sapiens] NTF2-like export factor 1 [Homo sapiens] NXT1
sw20346	BGIBMGA007678	1.5597	0.8628	1.3605	3.63995	8.00E-44	gi|66514096|ref|XP_392261.2| PREDICTED: similar to ENSANGP00000015840 [Apis mellifera]
sw03016	BGIBMGA002151	0.8939	1.0902	1.0261	3.6391	7.00E-30	gi|70984713|ref|XP_747863.1| peptidyl prolyl cis-trans isomerase [Aspergillus fumigatus Af293] peptidyl prolyl cis-trans isomerase (CypC), putative [Aspergillus fumigatus Af293]
sw00595	BGIBMGA003062	1.8259	1.33625	1.12435	3.6383	1.00E-21	gi|2961127|gb|AAC05666.1| putative pupal-specific cuticular protein CP2b [Anopheles gambiae]
sw06251	BGIBMGA011764	1.68945	1.58705	1.23255	3.63475	No hits found	
sw03246	BGIBMGA011869	1.0103	1.3309	0.9941	3.63335	5.00E-06	gi|21356467|ref|NP_649411.1| CG14450-PA [Drosophila melanogaster] RE67859p [Drosophila melanogaster] CG14450-PA [Drosophila melanogaster]
sw05747	BGIBMGA006794	0.9452	1.23065	1.10885	3.6298	3.00E-42	gi|7340062|gb|AAF61067.1| testis enhanced gene transcript-like protein [Paralichthys olivaceus] Probable Bax inhibitor-1 (BI-1)
sw14297	BGIBMGA003840	0.70715	0.66265	1.01615	3.62745	2.00E-28	gi|17945870|gb|AAL48981.1| RE39488p [Drosophila melanogaster]
sw17300	BGIBMGA007123	1.0195	0.99915	0.9744	3.6251	No hits found	
sw17048	BGIBMGA003698	0.6904	1.1089	0.8297	3.62485	3.00E-21	gi|28574095|ref|NP_652562.2| CG18661-PA [Drosophila melanogaster] CG18661-PA [Drosophila melanogaster]
sw13398	BGIBMGA002540	0.92545	1.0469	1.0954	3.6243	1.00E-52	gi|6006703|gb|AAF00577.1| protoporphyrinogen oxidase [Drosophila melanogaster]
sw05265	BGIBMGA001278	0.99985	1.1231	0.95765	3.6181	6.00E-50	gi|34899120|ref|NP_910906.1| putative adenosine deaminase [Oryza sativa (japonica cultivar-group)] putative adenosine deaminase [Oryza sativa (japonica cultivar-group)]
sw08480	BGIBMGA000681	4.27915	1.57315	2.479	3.6104	1.00E-14	gi|6706437|emb|CAB66004.1| Gly-rich protein [Drosophila melanogaster]
sw03796	BGIBMGA004009	0.7396	0.8941	0.8191	3.59665	1.00E-38	gi|4505369|ref|NP_002486.1| NADH dehydrogenase (ubiquinone) Fe-S protein 4, 18kDa (NADH-coenzyme Q reductase) [Homo sapiens] NADH:ubiquinone oxidoreductase 18 kDa IP subunit
sw12210	BGIBMGA000948	1.0209	1.18395	0.74875	3.59435	7.00E-61	gi|87248261|gb|ABD36183.1| anaphase promoting complex subunit 10 [Bombyx mori]
sw04194	BGIBMGA007193	0.9186	1.14815	1.0677	3.5941	3.00E-72	gi|40352976|gb|AAH64593.1| DCP2 protein [Homo sapiens]
sw20297	BGIBMGA004524	1	1.4537	1.83195	3.5869	2.00E-35	gi|62637998|gb|AAX92638.1| glucose transporter 8 [Solenopsis invicta]
sw13821	BGIBMGA011019	0.7455	1.00075	1.0145	3.58675	e-120	gi|87248121|gb|ABD36113.1| endothelial-monocyte activating polypeptide II [Bombyx mori]
sw15711	BGIBMGA004218	0.7422	0.99545	0.796	3.5789	1.00E-63	gi|73992280|ref|XP_867233.1| PREDICTED: similar to Dolichol-phosphate mannosyltransferase (Dolichol-phosphate mannose synthase) (Dolichyl-phosphate beta-D-mannosyltransferase)
sw11028	BGIBMGA010841	0.91325	0.99595	0.83235	3.57765	4.00E-13	gi|25012207|gb|AAN71219.1| GM25447p [Drosophila melanogaster]
sw21957	BGIBMGA000086	1.02405	1.2405	1.1237	3.57555	3.00E-57	gi|66500131|ref|XP_392131.2| PREDICTED: similar to MGC68697 protein [Apis mellifera]
sw14956	BGIBMGA005943	0.8959	0.95345	0.8299	3.5745	3.00E-98	gi|55235413|gb|EAA14740.2| ENSANGP00000015949 [Anopheles gambiae str. PEST] ENSANGP00000015949 [Anopheles gambiae str. PEST]
sw17819	BGIBMGA010975	0.8697	1.19725	1.1201	3.566	5.00E-49	gi|74095893|ref|NP_001027774.1| FRG1 protein [Takifugu rubripes] FRG1 [Takifugu rubripes] FRG1 [Takifugu rubripes] FRG1 protein
sw11182	BGIBMGA004978	0.9477	0.97135	0.89185	3.5588	1.00E-23	gi|55239530|gb|EAA10573.2| ENSANGP00000021605 [Anopheles gambiae str. PEST] ENSANGP00000021605 [Anopheles gambiae str. PEST]
sw21012	BGIBMGA006507	0.6543	0.93395	0.78225	3.5569	2.00E-92	gi|3283068|gb|AAC72298.1| reverse transcriptase [Caenorhabditis elegans]
sw20307	BGIBMGA006760	0.80795	1.12135	0.8075	3.5563	6.00E-12	gi|47217972|emb|CAG02255.1| unnamed protein product [Tetraodon nigroviridis]
sw17807	BGIBMGA010560	1	0.4504	0.53785	3.5443	0	gi|73994438|ref|XP_849294.1| PREDICTED: similar to phosphatidylinositol transfer protein, membrane-associated 2 isoform 2 [Canis familiaris]
sw10037	BGIBMGA007549	1.00735	1.08195	0.5945	3.537	5.00E-09	gi|76642627|ref|XP_593949.2| PREDICTED: similar to tigger transposable element derived 1 [Bos taurus]

sw10747	BGIBMGA009830	1.1275	1.45765	1.40725	3.5298	e-112	gi|50416646|gb|AAH77665.1| Integrin beta 4 binding protein [Xenopus tropicalis] integrin beta 4 binding protein [Xenopus tropicalis] integrin beta 4 binding protein [Xenopus tropicalis] integrin beta 4 binding protein [Xenopus tropicalis]
sw18585	BGIBMGA010745	1.37205	1.0557	1.40475	3.52605	e-102	gi|49899179|gb|AAH75765.1| IMP4, U3 small nucleolar ribonucleoprotein, homolog [Danio rerio] IMP4, U3 small nucleolar ribonucleoprotein, homolog [Danio rerio]
sw22978	BGIBMGA005460	0.9712	1.10585	1.1601	3.5258	1.00E-95	gi|68397044|ref|XP_708002.1| PREDICTED: similar to Ftsj homolog isoform 2 [Danio rerio] PREDICTED: similar to Ftsj homolog isoform 2 [Danio rerio]
sw07699	BGIBMGA009454	0.82615	1.407	1	3.5196	1.00E-64	gi|76630905|ref|XP_873154.1| PREDICTED: similar to chromosome 9 open reading frame 78 isoform 2 isoform 2 [Bos taurus]
sw00299	BGIBMGA007734	1.02835	1.1505	1.25265	3.51875	8.00E-90	gi|66509665|ref|XP_397030.2| PREDICTED: similar to bystin [Apis mellifera]
sw20314	BGIBMGA012866	1.54545	0.83625	0.338	3.50685	1.00E-72	gi|13359090|dbj|BAB33294.1| Bacteriophage T7 lysozyme-like protein 1 (BTL-LP1) [Bombyx mori]
sw14167	BGIBMGA003386	0.6473	1.14135	1.03695	3.4988	3.00E-14	gi|9930610|gb|AAG02114.1| steroid receptor RNA activator isoform 1 [Homo sapiens]
sw05478	BGIBMGA009559	0.65585	0.895	0.77325	3.4921	9.00E-32	gi|62648312|ref|XP_342783.2| PREDICTED: similar to CGI-141 protein [Rattus norvegicus]
sw04453	BGIBMGA009033	1.10195	1.2061	1.01635	3.4918	1.00E-58	gi|7862150|gb|AAF70499.1| 3-dehydroecdysone 3alpha-reductase [Spodoptera littoralis]
sw22960	BGIBMGA010563	2.26375	1.46315	2.29005	3.48925	6.00E-42	gi|37591031|dbj|BAC98835.1| juvenile hormone acid methyltransferase [Bombyx mori]
sw22947	BGIBMGA009009	1.0098	1.1762	1.30555	3.4869	3.00E-31	gi|34870767|ref|XP_220504.2| PREDICTED: similar to novel zinc finger protein [Rattus norvegicus]
sw12949	BGIBMGA009279	1	1	1	3.45635	2.00E-06	gi|26006205|dbj|BAC41445.1| mKIAA0701 protein [Mus musculus]
sw08936	BGIBMGA006859	0.91025	0.9405	0.79585	3.45035	3.00E-10	gi|68364782|ref|XP_690709.1| PREDICTED: similar to mitochondrial NADH-ubiquinone oxidoreductase ESSS subunit precursor [Danio rerio]
sw09007	BGIBMGA014563	1.2087	1.2125	1.28455	3.44805	8.00E-51	gi|55641019|ref|XP_510046.1| PREDICTED: similar to chromosome 14 open reading frame 169; NO66 [Pan troglodytes]
sw13748	BGIBMGA001943	0.8259	1.11345	0.932	3.44715	3.00E-38	gi|50539666|ref|NP_001002298.1| arsA arsenite transporter, ATP-binding, homolog 1 [Danio rerio] ArsA arsenite transporter, ATP-binding, homolog 1 [Danio rerio]
sw11723	BGIBMGA008621	1.2601	1.21135	1.3012	3.44515	6.00E-81	gi|72006882|ref|XP_780014.1| PREDICTED: similar to 3-hydroxyacyl-CoA dehydrogenase type II (Type II HADH) (Scully protein) isoform 1 [Strongylocentrotus purpuratus]
sw03582	BGIBMGA010723	0.752	0.8222	0.99735	3.44095	1.00E-36	gi|50417589|gb|AAH77664.1| MGC89748 protein [Xenopus tropicalis] MGC89748 protein [Xenopus tropicalis] small nuclear ribonucleoprotein D2 polypeptide 16.5kDa [Xenopus tropicalis]
sw08576	BGIBMGA012606	1.87615	0.71925	0.99895	3.434	No hits found	
sw03207	BGIBMGA007490	1.02715	1.0692	1.072	3.425	e-178	gi|90309026|gb|ABA43638.2| glyceraldehyde-3-phosphate dehydrogenase [Bombyx mori]
sw08771	BGIBMGA011824	0.69305	0.90815	0.7418	3.42335	2.00E-36	gi|51261910|gb|AAH79933.1| MGC79564 protein [Xenopus tropicalis] MGC79564 protein [Xenopus tropicalis] ARD1 homolog A, N-acetyltransferase
sw09115	BGIBMGA013923	0.7921	0.89075	0.8561	3.42315	8.00E-42	gi|73960888|ref|XP_547528.2| PREDICTED: similar to mitochondrial ribosomal protein L24 isoform 1 [Canis familiaris]
sw13447	BGIBMGA010734	1	1	1	3.42255	No hits found	
sw04091	BGIBMGA002546	0.9758	1	0.76305	3.4214	5.00E-24	gi|72092440|ref|XP_790987.1| PREDICTED: similar to peptide deformylase-like protein [Strongylocentrotus purpuratus]
sw09558	BGIBMGA011493	0.8274	1.24935	0.89325	3.41835	6.00E-36	gi|266752|sp|P29702|PFTA_BOVIN Protein farnesyltransferase/geranylgeranyltransferase type I alpha subunit (CAAX farnesyltransferase alpha subunit)
sw09837	BGIBMGA009504	0.69285	1.04265	0.8559	3.41405	Bmb036914	9.00E-25
sw21496	BGIBMGA008878	0.75275	1.1699	0.84725	3.4119	Bmb025395	1.00E-24
sw11603	BGIBMGA011856	0.96625	1.02235	0.83465	3.41155	8.00E-38	gi|77403939|gb|ABA81848.1| AT16804p [Drosophila melanogaster] CG10809-PA [Drosophila melanogaster] CG10809-PA [Drosophila melanogaster]
sw22043	BGIBMGA013864	1.09445	0.59215	0.5785	3.4076	5.00E-98	gi|52421209|dbj|BAD51475.1| gloverin-like protein 3 [Bombyx mori]

sw03657	BGIBMGA012563	0.91325	1.34765	1	3.405	6.00E-18	gi|66560076|ref|XP_624348.1| PREDICTED: similar to CG12114-PA [Apis mellifera]
sw17253	BGIBMGA012850	0.82675	1.1189	0.92825	3.39575	7.00E-08	gi|68373919|ref|XP_691048.1| PREDICTED: similar to zinc finger protein 569 [Danio rerio]
sw15517	BGIBMGA010229	0.98975	1.1503	1.0609	3.3931	2.00E-10	gi|54311324|gb|AAH84843.1| LOC495376 protein [Xenopus laevis]
sw08542	BGIBMGA009802	0.85365	1.2953	1.32085	3.38035	4.00E-09	gi|21292650|gb|EAA04795.1| ENSANGP00000019625 [Anopheles gambiae str. PEST] ENSANGP00000019625 [Anopheles gambiae str. PEST]
sw16856	BGIBMGA004846	0.87925	0.97295	1.12755	3.3802	Bmb048286	6.00E-30
sw01422	BGIBMGA002563	1.5304	0.9244	0.9523	3.3738	No hits found	
sw13103	BGIBMGA012537	0.9488	1.29205	1.0426	3.36615	e-113	gi|87248541|gb|ABD36323.1| mitochondrial ribosomal protein L32 [Bombyx mori]
sw06690	BGIBMGA004838	1.12365	1.14865	1.15845	3.3652	2.00E-13	gi|50756073|ref|XP_415003.1| PREDICTED: similar to mitochondrial ribosomal protein S5; mitochondrial 28S ribosomal protein S5 [Gallus gallus]
sw04653	BGIBMGA007630	1	1.2298	1	3.3647	5.00E-31	gi|76608529|ref|XP_883507.1| PREDICTED: similar to ZK1058.5 isoform 2 [Bos taurus]
sw07293	BGIBMGA010193	0.6423	0.9298	0.8057	3.3647	9.00E-59	gi|87248225|gb|ABD36165.1| short-chain dehydrogenease/reductase [Bombyx mori]
sw01825	BGIBMGA007173	0.96575	1.21945	0.9587	3.3543	1.00E-69	gi|66553058|ref|XP_623865.1| PREDICTED: similar to Syntaxin 18 (predicted) [Apis mellifera]
sw21459	BGIBMGA000039	0.7354	0.1754	0.29065	3.3526	No hits found	
sw11297	BGIBMGA009893	0.9997	1.19465	1.227	3.34885	5.00E-64	gi|72008188|ref|XP_781285.1| PREDICTED: similar to Exosome complex exonuclease RRP40 (Ribosomal RNA processing protein 40) (Exosome component 3) (p10) [Strongylocentrotus purpuratus]
sw12164	BGIBMGA007676	1.49775	1.3862	1.09835	3.3473	0	gi|62647472|ref|XP_216173.3| PREDICTED: similar to ubiquitin specific protease 39 [Rattus norvegicus]
sw17728	BGIBMGA008663	1.1776	1.1008	0.85105	3.34595	No hits found	
sw04446	BGIBMGA005384	0.70515	0.87545	0.7639	3.34545	2.00E-15	gi|12803607|gb|AAH02638.1| P53-inducible cell-survival factor [Homo sapiens] P53-inducible cell-survival factor [Homo sapiens] p53-inducible cell-survival factor
sw18213	BGIBMGA006083	0.7592	0.8457	1.0532	3.34255	4.00E-25	gi|76608452|ref|XP_874747.1| PREDICTED: similar to Carbonyl reductase [NADPH] 1 (NADPH-dependent carbonyl reductase 1)
sw18236	BGIBMGA006653	0.8619	1.009	1.07835	3.34035	No hits found	
sw04526	BGIBMGA007025	1.2407	0.98165	1.0806	3.33975	Bmb037524	6.00E-53
sw14459	BGIBMGA007146	1.3606	1.47215	1.1077	3.33585	e-124	gi|49329405|gb|AAT60051.1| kynureninase [Bacillus thuringiensis serovar konkukian str. 97-27] kynureninase [Bacillus thuringiensis serovar konkukian str. 97-27]
sw22928	BGIBMGA002423	0.95615	1.1013	0.91865	3.33535	2.00E-34	gi|48098602|ref|XP_394113.1| PREDICTED: similar to CG1458-PA [Apis mellifera]
sw13044	BGIBMGA011078	0.68935	1.19075	0.61055	3.33015	6.00E-05	gi|6560645|gb|AAF16700.1| juvenile hormone binding protein precursor-like protein [Manduca sexta]
sw14233	BGIBMGA007266	1.00725	1.28295	0.9557	3.3296	Bmb023690	9.00E-52
sw19198	BGIBMGA006638	1.0057	1.17755	1.1188	3.3173	7.00E-22	gi|87248471|gb|ABD36288.1| calcitonin gene-related peptide-receptor component protein [Bombyx mori]
sw13830	BGIBMGA004023	0.7419	0.8781	0.9364	3.31335	3.00E-44	gi|25013122|gb|AAN71660.1| SD14834p [Drosophila melanogaster]
sw13406	BGIBMGA000112	0.9892	1.10855	1.05295	3.3108	3.00E-40	gi|90103466|gb|ABD85577.1| unknown [Ictalurus punctatus]
sw22297	BGIBMGA011616	2.30325	1.5702	3.03355	3.3076	5.00E-23	gi|72016315|ref|XP_783630.1| PREDICTED: similar to Fibrillin-2 precursor, partial [Strongylocentrotus purpuratus]
sw00457	BGIBMGA006631	0.96355	1.14375	1.01485	3.30645	9.00E-19	gi|56270469|gb|AAH87416.1| LOC496023 protein [Xenopus laevis]

sw16286	BGIBMGA006754	0.84045	1.08315	0.87625	3.3052	Bmb040031	e-161
sw04869	BGIBMGA011061	0.9434	0.9536	0.81315	3.30495	7.00E-12	gi|76676986|ref|XP_591925.2| PREDICTED: similar to CG12379-PA [Bos taurus]
sw02779	BGIBMGA008137	1	0.8413	1	3.28305	1.00E-48	gi|21064791|gb|AAM29625.1| RH67819p [Drosophila melanogaster]
sw17094	BGIBMGA011031	0.86565	1.21335	0.7647	3.2795	4.00E-20	gi|21357917|ref|NP_650514.1| CG4287-PA [Drosophila melanogaster] GH08991p [Drosophila melanogaster] CG4287-PA [Drosophila melanogaster]
sw10439	BGIBMGA003563	0.87915	0.9284	0.88725	3.27885	3.00E-06	gi|72146037|ref|XP_797557.1| PREDICTED: similar to Myc-induced mitochondria protein [Strongylocentrotus purpuratus]
sw15421	BGIBMGA000293	1.198	1.17985	1.081	3.27705	6.00E-31	gi|14211570|dbj|BAB56112.1| conserved ERA-like GTPase [Homo sapiens]
sw18742	BGIBMGA009671	0.8869	0.90415	1.00255	3.27705	8.00E-47	gi|72005686|ref|XP_780645.1| PREDICTED: similar to isochorismatase domain containing 1 [Strongylocentrotus purpuratus]
sw05567	BGIBMGA012403	1.48865	1.2945	1.48925	3.27485	9.00E-10	gi|54636883|gb|EAL26286.1| GA10354-PA [Drosophila pseudoobscura]
sw09446	BGIBMGA002417	0.8522	1.17685	0.85535	3.27375	Bmb030081	4.00E-17
sw07006	BGIBMGA011450	1.0402	1.3244	1.11625	3.2687	Bmb040045	2.00E-84
sw05257	BGIBMGA009618	0.8471	0.78115	0.9092	3.26655	No hits found	
sw11364	BGIBMGA006632	0.91005	1.0876	1.17785	3.2657	3.00E-29	gi|72005635|ref|XP_785582.1| PREDICTED: similar to WD repeat domain 58 [Strongylocentrotus purpuratus]
sw09679	BGIBMGA011697	0.8127	1.0748	0.8774	3.2649	3.00E-20	gi|41054111|ref|NP_956148.1| ring finger protein 146 [Danio rerio] Ring finger protein 146 [Danio rerio]
sw14835	BGIBMGA002933	0.402	0.92545	0.68325	3.26435	No hits found	
sw06597	BGIBMGA003527	0.4496	1.17275	0.6386	3.2471	8.00E-51	gi|25012624|gb|AAN71409.1| RE44531p [Drosophila melanogaster]
sw05314	BGIBMGA001283	1.06515	1.3651	1.4077	3.24645	3.00E-21	gi|27695162|gb|AAH43795.1| Mak16l-prov protein [Xenopus laevis] MAK16-like protein RBM13 B (RNA binding motif protein 13-like B)
sw10989	BGIBMGA013497	1.20925	0.89395	1.2117	3.24645	5.00E-65	gi|27462217|gb|AAO15385.1| DAP3 [Drosophila melanogaster]
sw22147	BGIBMGA003616	1.65545	1.78335	1.1662	3.24465	2.00E-18	gi|72111613|ref|XP_791607.1| PREDICTED: similar to phospholipid scramblase 1 [Strongylocentrotus purpuratus]
sw08911	BGIBMGA013542	2.05445	1.6801	0.61215	3.2444	Bmb021438	No hits found
sw09671	BGIBMGA006920	0.74975	0.92775	0.69595	3.2443	1.00E-23	gi|72047001|ref|XP_787000.1| PREDICTED: similar to programmed cell death 6 (1F340), partial [Strongylocentrotus purpuratus]
sw15014	BGIBMGA007408	0.8938	0.829	0.7816	3.244	9.00E-38	gi|74002101|ref|XP_535685.2| PREDICTED: similar to Short chain 3-hydroxyacyl-CoA dehydrogenase, mitochondrial precursor (HCDH) (Medium and short chain L-3-hydroxyacyl-coenzyme A dehydrogenase) isoform 1 [Canis familiaris]
sw05030	BGIBMGA004008	0.80415	0.9498	0.8453	3.2354	2.00E-07	gi|72024737|ref|XP_799052.1| PREDICTED: similar to coiled-coil-helix-coiled-coil-helix domain containing 1 [Strongylocentrotus purpuratus]
sw06168	BGIBMGA006477	0.6352	0.7954	0.994	3.2348	3.00E-29	gi|72005469|ref|XP_785869.1| PREDICTED: similar to prefoldin 5 [Strongylocentrotus purpuratus]
sw14679	BGIBMGA006574	1.0909	1.04885	1.35755	3.2331	3.00E-73	gi|68358710|ref|XP_688730.1| PREDICTED: similar to heat shock 70kDa protein 14 isoform 1 [Danio rerio]
sw20681	BGIBMGA012873	0.8395	1.0868	0.8646	3.23285	2.00E-10	gi|20071201|gb|AAH26744.1| Vacuolar protein sorting 37B [Mus musculus] vacuolar protein sorting 37B [Mus musculus]
sw09038	BGIBMGA010866	0.7214	0.87715	0.79915	3.22645	5.00E-91	gi|32029913|ref|ZP_00132857.1| COG0451: Nucleoside-diphosphate-sugar epimerases [Haemophilus somnus 2336] COG0451: Nucleoside-diphosphate-sugar epimerases [Haemophilus somnus 129PT]
sw14128	BGIBMGA000882	0.57585	0.9283	0.7977	3.22165	1.00E-32	gi|87248177|gb|ABD36141.1| ribosomal protein L27 [Bombyx mori]
sw14985	BGIBMGA000506	0.68965	0.9115	0.85555	3.2169	4.00E-32	gi|74006183|ref|XP_545715.2| PREDICTED: similar to lysophospholipase-like 1 [Canis familiaris]

sw03143	BGIBMGA001444	1	0.8157	1.31645	3.21425	3.00E-91	gi|23096118|dbj|BAC16225.1| cuticle protein [Bombyx mori]
sw05818	BGIBMGA009816	1.0969	1.2013	1.45765	3.21265	e-163	gi|50758198|ref|XP_415805.1| PREDICTED: similar to splicing factor Prp8 [Gallus gallus]
sw17569	BGIBMGA007973	1	0.94995	1	3.20855	No hits found	
sw04361	BGIBMGA007744	0.8762	0.8888	0.6847	3.2076	7.00E-09	gi|46249482|gb|AAH68670.1| MGC81062 protein [Xenopus laevis] Sld5 [Xenopus laevis]
sw11939	BGIBMGA006198	1.0896	1.0756	1.0358	3.20755	3.00E-32	gi|41529182|dbj|BAD08441.1| mitochondrial ribosomal protein S18-2 [Sus scrofa] mitochondrial ribosomal protein S18-2 [Sus scrofa]
sw10616	BGIBMGA000801	1.31455	1.258	0.976	3.20745	No hits found	
sw20320	BGIBMGA005955	0.7528	1.07655	0.9219	3.20585	3.00E-23	gi|27465077|gb|AAO12862.1| Camar1 transposase [Chymomyza amoena]
sw10358	BGIBMGA011578	0.95425	1.2916	1.19235	3.20015	4.00E-07	gi|62859459|ref|NP_001016090.1| hypothetical protein LOC548844 [Xenopus tropicalis]
sw03481	BGIBMGA007780	1	1.89925	1	3.1979	1.00E-47	gi|77686775|ref|ZP_00802100.1| Indigoidine synthase A like protein [Alkaliphilus metalliredigenes QYMF] Indigoidine synthase A like protein [Alkaliphilus metalliredigenes QYMF]
sw05264	BGIBMGA014607	0.75155	0.9502	0.71735	3.1974	8.00E-47	gi|87248417|gb|ABD36261.1| translocase of inner mitochondrial membrane 8 homolog b [Bombyx mori]
sw15298	BGIBMGA013785	1.10375	1.13005	0.9218	3.1906	No hits found	
sw13309	BGIBMGA007688	0.98595	1.07175	0.8774	3.18185	7.00E-47	gi|48097408|ref|XP_391891.1| PREDICTED: similar to zinc finger, ZZ domain containing 3 [Apis mellifera]
sw03458	BGIBMGA010016	0.97745	0.9744	1.0889	3.1805	4.00E-54	gi|87248389|gb|ABD36247.1| rotamase Pin1 [Bombyx mori]
sw06454	BGIBMGA012774	0.95925	1.3744	1.2024	3.17425	8.00E-32	gi|24642095|ref|NP_572999.1| CG15027-PA [Drosophila melanogaster] CG15027-PA [Drosophila melanogaster]
sw04870	BGIBMGA008492	1.0187	1.80415	1.7541	3.17275	8.00E-16	gi|54645636|gb|EAL34374.1| GA10125-PA [Drosophila pseudoobscura]
sw18921	BGIBMGA009495	0.7584	0.9024	0.877	3.1722	0	gi|54290087|dbj|BAD61055.1| RFC40 [Bombyx mori]
sw13377	BGIBMGA004056	0.8585	0.8798	0.93145	3.16985	2.00E-21	gi|62642817|ref|XP_226716.3| PREDICTED: similar to Pentatricopeptide repeat domain 2 [Rattus norvegicus]
sw05505	BGIBMGA003422	0.74285	0.83585	0.57815	3.16935	5.00E-11	gi|55243996|gb|EAA06017.3| ENSANGP00000017964 [Anopheles gambiae str. PEST] ENSANGP00000017964 [Anopheles gambiae str. PEST]
sw06665	BGIBMGA003070	0.8652	0.99665	0.8712	3.1649	4.00E-28	gi|14250353|gb|AAH08607.1| Translocase of inner mitochondrial membrane 13 [Homo sapiens] translocase of inner mitochondrial membrane 13 [Homo sapiens] TIMM13b [Homo sapiens] small zinc finger-like protein
sw01123	BGIBMGA011459	1.05665	1.13105	0.9106	3.1648	Bmb016767	4.00E-25
sw14615	BGIBMGA005519	0.87205	0.95965	1.08345	3.16445	1.00E-04	gi|42733535|dbj|BAD11364.1| TOM22 [Rattus norvegicus] TOM22 protein [Rattus norvegicus] TOM22 protein [Rattus norvegicus]
sw08356	BGIBMGA002886	1.06135	1.1467	0.78055	3.15895	3.00E-51	gi|55669751|pdb|1SNY|A Chain A, Carbonyl Reductase Sniffer Of D. Melanogaster
sw13511	BGIBMGA005615	0.66375	0.8502	0.7176	3.15825	8.00E-50	gi|72121083|gb|AAZ63269.1| Zinc-containing alcohol dehydrogenase superfamily [Ralstonia eutropha JMP134] Zinc-containing alcohol dehydrogenase superfamily [Ralstonia eutropha JMP134]
sw07771	BGIBMGA012478	0.7941	0.8599	1.05255	3.1573	9.00E-30	gi|2331217|gb|AAB66878.1| trypsin [Anopheles stephensi]
sw03543	BGIBMGA007702	0.90555	1.09455	1.1075	3.14595	6.00E-79	gi|2285788|dbj|BAA21658.1| Multiprotein bridging factor 1 [Bombyx mori]
sw08382	BGIBMGA002720	0.7397	1.1591	0.69985	3.14255	No hits found	
sw15595	BGIBMGA001490	0.8492	1.18575	1.81515	3.14135	e-133	gi|68160236|gb|AAY86706.1| 45 kDa immunophilin FKBP45 [Bombyx mori]
sw16230	BGIBMGA002445	0.83335	1.2076	1.26985	3.1344	3.00E-36	gi|89268189|emb|CAJ83671.1| adenosine deaminase, tRNA-specific 1 [Xenopus tropicalis]

sw08133	BGIBMGA012474	0.7398	0.9055	0.9205	3.1324	1.00E-37	gi|66539173|ref|XP_624932.1| PREDICTED: similar to Huntingtin interacting protein K, partial [Apis mellifera]
sw14639	BGIBMGA011782	1.0933	1.07795	1.0757	3.13045	1.00E-41	gi|73958726|ref|XP_861741.1| PREDICTED: similar to exonuclease NEF-sp isoform 5 [Canis familiaris]
sw09583	BGIBMGA009113	0.6736	1.1058	0.98635	3.12725	2.00E-16	gi|76658212|ref|XP_887786.1| PREDICTED: similar to Nucleoside diphosphate-linked moiety X motif 8, mitochondrial precursor (Nudix motif 8) isoform 2 [Bos taurus]
sw21578	BGIBMGA013054	0.811	1.17275	1.2009	3.1246	Bmb040067	e-123
sw16042	BGIBMGA004870	1.10735	1.25075	1.2345	3.12435	No hits found	
sw03532	BGIBMGA009360	0.9198	1.1711	0.90865	3.1235	1.00E-33	gi|66504312|ref|XP_395230.2| PREDICTED: similar to DNA polymerase gamma [Apis mellifera]
sw04517	BGIBMGA008815	1.0526	1.17035	0.9006	3.1204	7.00E-63	gi|49532884|dbj|BAD26677.1| Juvenile hormone diol kinase [Plutella xylostella]
sw07091	BGIBMGA004280	1.2673	0.96145	1.0756	3.11805	Bmb042783	2.00E-81
sw07852	BGIBMGA005306	0.39475	1.0466	0.65895	3.11385	2.00E-07	gi|72014822|ref|XP_783246.1| PREDICTED: similar to sarcolemma associated protein [Strongylocentrotus purpuratus]
sw14170	BGIBMGA001806	0.9467	1.20165	1.1921	3.1078	e-106	gi|66499810|ref|XP_624522.1| PREDICTED: similar to cleavage and polyadenylation specific factor 5 [Apis mellifera]
sw12428	BGIBMGA006568	0.8183	0.8749	0.92195	3.10595	1.00E-48	gi|55245504|gb|EAA04466.2| ENSANGP00000018430 [Anopheles gambiae str. PEST] ENSANGP00000018430 [Anopheles gambiae str. PEST]
sw08641	BGIBMGA008494	0.94315	1.1391	0.9906	3.0993	Bmb017333	1.00E-20
sw08766	BGIBMGA011338	0.94795	0.9728	1.0491	3.09535	8.00E-98	gi|38176158|ref|NP_003849.2| cyclin K [Homo sapiens] Cyclin K [Homo sapiens] cyclin K [Homo sapiens] cyclin K [Homo sapiens] cyclin K [synthetic construct] cyclin K [synthetic construct]
sw09618	BGIBMGA008478	0.79275	1.25265	1.09185	3.09505	Bmb032873	3.00E-29
sw15985	BGIBMGA012059	1.26765	1.276	1.35535	3.08205	8.00E-40	gi|27462213|gb|AAO15383.1| multiple hat domains [Mus musculus]
sw06451	BGIBMGA002985	0.7175	1.0562	0.84065	3.0789	3.00E-73	gi|3983135|gb|AAC83820.1| platelet-activating factor acetylhydrolase alpha subunit homolog [Drosophila melanogaster]
sw08302	BGIBMGA005659	0.8183	1.03505	0.86765	3.07785	1.00E-17	gi|25013071|gb|AAN71628.1| RH67738p [Drosophila melanogaster]
sw08827	BGIBMGA012029	0.7413	0.92205	0.63255	3.07705	2.00E-42	gi|87248631|gb|ABD36368.1| mitochondrial thioredoxin 2 [Bombyx mori]
sw19580	BGIBMGA010370	1.6497	0.88645	0.71975	3.07385	3.00E-47	gi|4160012|gb|AAD05225.1| dissatisfaction [Drosophila melanogaster]
sw01104	BGIBMGA000320	1.0181	0.8504	1.1564	3.07275	2.00E-23	gi|68371190|ref|XP_696017.1| PREDICTED: similar to programmed cell death 2 [Danio rerio]
sw06213	BGIBMGA010349	1.87405	1.715	1.4625	3.0638	4.00E-21	gi|15146427|gb|AAK84661.1| retinoid-inducible serine carboxypeptidase precursor [Rattus norvegicus] serine carboxypeptidase 1 [Rattus norvegicus] Retinoid-inducible serine carboxypeptidase precursor (Serine carboxypeptidase 1)
sw15398	BGIBMGA009239	0.711	0.89365	0.8618	3.05875	Bmb032363	No hits found
sw09920	BGIBMGA001163	1	1	1	3.0581	e-127	gi|48094345|ref|XP_394147.1| PREDICTED: similar to cyclic AMP-dependent catalytic subunit (41.4 kD) (kin-1) [Apis mellifera]
sw14215	BGIBMGA000926	0.6608	0.81265	0.79755	3.0561	9.00E-46	gi|12002054|gb|AAG43169.1| transaldolase [Rattus norvegicus] Transaldolase
sw14910	BGIBMGA009706	1.07145	0.9293	0.91015	3.05515	3.00E-33	gi|55632729|ref|XP_520400.1| PREDICTED: similar to NADH dehydrogenase (ubiquinone) 1 alpha subcomplex, 8, 19kDa; NADH-ubiquinone oxidoreductase 19 kDa subunit; NADH:ubiquinone oxidoreductase PGIV subunit
sw11857	BGIBMGA007452	1.2332	1.23325	0.9803	3.0485	2.00E-09	gi|49618945|gb|AAT68057.1| pinball wizard [Danio rerio] tryptophan rich basic protein [Danio rerio]
sw04522	BGIBMGA002766	1.18965	1.3139	1.38135	3.04575	2.00E-40	gi|62858747|ref|NP_001016308.1| apoptosis antagonizing transcription factor [Xenopus tropicalis]
sw15787	BGIBMGA001340	0.84125	0.963	0.8362	3.03355	6.00E-90	gi|66515816|ref|XP_392546.2| PREDICTED: similar to Pantothenate kinase 4 (Pantothenic acid kinase 4) (hPanK4), partial [Apis mellifera]

sw10500	BGIBMGA012093	0.8445	0.8319	0.97855	3.0333	e-167	gi|6448449|dbj|BAA86901.1| mitochondrial carrier homolog [Bombyx mori]
sw17844	BGIBMGA005739	0.4853	0.83325	0.6231	3.03025	3.00E-31	gi|68362888|ref|XP_697922.1| PREDICTED: similar to DNA-directed RNA polymerase II 7.6 kDa polypeptide (RPB10) (RPB7.6) (RPABC5) [Danio rerio]
sw13376	BGIBMGA001464	0.94405	0.9608	1.17395	3.02825	1.00E-76	gi|55640387|ref|XP_509840.1| PREDICTED: similar to oxidase (cytochrome c) assembly 1-like [Pan troglodytes]
sw14544	BGIBMGA005023	0.82485	1.1401	0.91945	3.0271	1.00E-23	gi|71153817|sp|Q9NX58|LYAR_HUMAN Cell growth-regulating nucleolar protein
sw08769	BGIBMGA013896	0.8972	0.9976	1.2133	3.0229	2.00E-75	gi|56270437|gb|AAH87307.1| LOC495942 protein [Xenopus laevis]
sw00137	BGIBMGA010231	3.29465	0.5963	1.481	3.02175	2.00E-24	gi|87248419|gb|ABD36262.1| larval cuticle protein [Bombyx mori]
sw16228	BGIBMGA006401	0.5594	0.67325	0.84125	3.01805	Bmb039284	8.00E-19
sw16854	BGIBMGA007767	1.41845	0.73505	0.87545	3.0115	Bmb048271	1.00E-74
sw18883	BGIBMGA005897	0.79005	1.3136	1.1784	3.01135	2.00E-92	gi|28317069|gb|AAO39553.1| RE01362p [Drosophila melanogaster]
sw06760	BGIBMGA012554	0.9475	0.88025	0.78175	3.01105	Bmb034003	8.00E-11
sw21407	BGIBMGA006219	0.7739	1.0804	0.75605	3.0108	4.00E-06	gi|62644944|ref|XP_575113.1| PREDICTED: similar to Hypothetical UPF0184 protein C9orf16 homolog [Rattus norvegicus]
sw14203	BGIBMGA011656	0.9584	1.2901	1.2879	3.0102	2.00E-38	gi|54636912|gb|EAL26315.1| GA11566-PA [Drosophila pseudoobscura]
sw11583	BGIBMGA001068	1.0169	1.02585	1.18075	3.00565	8.00E-95	gi|87248327|gb|ABD36216.1| fumarylacetoacetate hydrolase isoform A [Bombyx mori]
sw21178	BGIBMGA001792	0.7409	1.0765	0.9675	3.00075	1.00E-24	gi|73949006|ref|XP_544251.2| PREDICTED: similar to Artemis protein (DNA cross-link repair 1C protein) (SNM1-like protein) (A-SCID protein) (hSNM1C) isoform 1 [Canis familiaris]
sw17304	BGIBMGA001343	1.2077	1.09835	0.9301	3.0005	6.00E-45	gi|58476597|gb|AAH89985.1| Fused toes (predicted) [Rattus norvegicus] fused toes (predicted) [Rattus norvegicus]
sw13351	BGIBMGA000332	1.44705	0.6839	1.02845	2.9922	5.00E-20	gi|3913392|sp|O02388|CU22_BOMMO Larval cuticle protein LCP-22 precursor LCP22 [Bombyx mori]
sw22950	BGIBMGA000940	1.1445	1.19465	0.926	2.9866	1.00E-28	gi|66499360|ref|XP_396507.2| PREDICTED: similar to CG11550-PA [Apis mellifera]
sw04913	BGIBMGA003663	1	1.085	1	2.98565	2.00E-40	gi|50750445|ref|XP_421997.1| PREDICTED: similar to methionine aminopeptidase 1D; similar to methionine aminopeptidase-like 1 [Gallus gallus]
sw16343	BGIBMGA007263	1.08265	1.374	1.25415	2.9851	4.00E-05	gi|24582874|ref|NP_609236.2| CG13096-PA [Drosophila melanogaster] CG13096-PA [Drosophila melanogaster]
sw12549	BGIBMGA008574	1.07775	1.54095	0.92855	2.9847	5.00E-06	gi|21617857|ref|NP_660193.1| CG15107-PA [Drosophila melanogaster] LD14312p [Drosophila melanogaster] CG15107-PA [Drosophila melanogaster]
sw09644	BGIBMGA004054	0.9977	1.27535	1.0227	2.98465	Bmb033291	2.00E-91
sw10427	BGIBMGA010129	1.11935	1.10495	1.26995	2.9818	e-161	gi|66552237|ref|XP_623630.1| PREDICTED: similar to ribonuclease L inhibitor homolog [Apis mellifera]
sw03985	BGIBMGA009934	1.1318	0.95975	0.7892	2.98015	No hits found	
sw16645	BGIBMGA010621	1	0.94375	1.375	2.9801	e-178	gi|73988085|ref|XP_862223.1| PREDICTED: similar to Ribonucleoside-diphosphate reductase large subunit (Ribonucleoside-diphosphate reductase M1 subunit) (Ribonucleotide reductase large chain) isoform 5 [Canis familiaris]
sw10573	BGIBMGA010920	1.28975	1.42965	1.00545	2.97835	4.00E-08	gi|55245618|gb|EAL41836.1| ENSANGP00000027947 [Anopheles gambiae str. PEST] ENSANGP00000027947 [Anopheles gambiae str. PEST]
sw07976	BGIBMGA011696	0.8792	0.92125	1.00585	2.97785	8.00E-16	gi|48102233|ref|XP_392756.1| PREDICTED: similar to GA14405-PA [Apis mellifera]
sw17810	BGIBMGA012127	0.88685	1.1207	1.52025	2.97695	1.00E-65	gi|89886082|ref|NP_989268.2| glutamate-rich WD repeat containing 1 [Xenopus tropicalis] glutamate-rich WD repeat containing 1 [Xenopus tropicalis]
sw12206	BGIBMGA000338	1.2652	0.6625	1.1206	2.97355	1.00E-34	gi|47605413|sp|Q7M4F3|CUD2_SCHGR Endocuticle structural glycoprotein SgAbd-2

sw14293	BGIBMGA010919	1.42745	1.7126	1.1242	2.97145	2.00E-20	gi|54637590|gb|EAL26992.1| GA19958-PA [Drosophila pseudoobscura]
sw16101	BGIBMGA011899	0.78455	1.1833	0.88885	2.96975	2.00E-11	gi|82498917|ref|ZP_00884371.1| diadenosine tetraphosphate (Ap4A) hydrolase and other HIT family hydrolases [Caldicellulosiruptor saccharolyticus DSM 8903] diadenosine tetraphosphate
sw15166	BGIBMGA012118	1.04305	1.1025	1.11055	2.9672	Bmb030649	e-104
sw17076	BGIBMGA011846	0.76605	1.0312	0.7128	2.9672	8.00E-40	gi|50753715|ref|XP_414101.1| PREDICTED: similar to hypothetical protein FLJ38101 [Gallus gallus] PREDICTED: similar to hypothetical protein FLJ38101 [Gallus gallus]
sw21910	BGIBMGA000616	0.8103	1.03495	0.9071	2.9664	8.00E-25	gi|54641436|gb|EAL30186.1| GA21196-PA [Drosophila pseudoobscura]
sw14801	BGIBMGA014257	1.4169	1.44805	0.9596	2.9625	No hits found	
sw12651	BGIBMGA006160	0.5586	1.03655	1.44745	2.96225	e-170	gi|76496246|gb|ABA43639.1| L-threonine dehydrogenase [Bombyx mori]
sw22908	BGIBMGA009021	0.86305	1.07115	0.9062	2.95935	2.00E-49	gi|66564402|ref|XP_396161.2| PREDICTED: similar to Sip-prov protein [Apis mellifera]
sw17113	BGIBMGA000061	0.65185	0.84705	0.70765	2.94795	5.00E-30	gi|62859501|ref|NP_001017111.1| mitochondrial ribosomal protein L35 [Xenopus tropicalis]
sw02239	BGIBMGA000664	0.7748	1.6878	1.283	2.94785	6.00E-42	gi|87248467|gb|ABD36286.1| unknown [Bombyx mori]
sw18482	BGIBMGA009726	0.95295	0.923	1.2149	2.9461	e-132	gi|87248625|gb|ABD36365.1| translin [Bombyx mori]
sw20510	BGIBMGA008676	0.74485	0.6635	0.71575	2.9431	No hits found	
sw20836	BGIBMGA005710	0.4171	0.77975	1.0747	2.94	e-109	gi|474339|gb|AAA17752.1| reverse transcriptase [Bombyx mori]
sw10418	BGIBMGA010137	0.7156	1.08425	0.89055	2.93975	3.00E-16	gi|24667277|ref|NP_649195.1| CG13813-PA [Drosophila melanogaster] IP11764p [Drosophila melanogaster] CG13813-PA [Drosophila melanogaster]
sw16297	BGIBMGA000936	0.75095	0.98915	1.0466	2.9376	2.00E-80	gi|55250104|gb|AAH85542.1| Ribose 5-phosphate isomerase A (ribose 5-phosphate epimerase) [Danio rerio] ribose 5-phosphate isomerase A (ribose 5-phosphate epimerase) [Danio rerio]
sw10381	BGIBMGA010620	0.78545	1.10255	0.89415	2.9361	6.00E-57	gi|89268101|emb|CAJ83820.1| syntaxin 5A [Xenopus tropicalis]
sw14247	BGIBMGA008971	1.5026	1.59105	1	2.93585	3.00E-22	gi|24643546|ref|NP_608400.1| CG15453-PA [Drosophila melanogaster] CG15453-PA [Drosophila melanogaster]
sw17423	BGIBMGA014117	0.9256	0.9292	0.9705	2.93545	8.00E-71	gi|62857375|ref|NP_001017177.1| dullard homolog [Xenopus tropicalis] dullard homolog (Xenopus laevis) [Xenopus tropicalis]
sw17297	BGIBMGA006604	0.87295	0.9047	0.86655	2.935	3.00E-23	gi|72113620|ref|XP_795139.1| PREDICTED: similar to cytochrome b5 domain-containing protein like (3F409) [Strongylocentrotus purpuratus]
sw20342	BGIBMGA004100	0.724	0.98425	0.8814	2.93145	8.00E-22	gi|76779920|gb|AAI06315.1| Flj13912-prov protein [Xenopus laevis] Flj13912-prov protein [Xenopus laevis] Psf3 [Xenopus laevis]
sw05469	BGIBMGA010478	1.1384	1.1261	1.30635	2.9282	1.00E-11	gi|54644737|gb|EAL33477.1| GA20198-PA [Drosophila pseudoobscura]
sw10611	BGIBMGA012747	1.34995	1.49155	1.35915	2.9258	1.00E-09	gi|2791944|emb|CAA76124.1| Drosophila multidendritic neurons sodium channel 1 [Drosophila melanogaster]
sw05749	BGIBMGA006173	1.0239	1.36025	1.18215	2.92505	3.00E-95	gi|8050580|gb|AAF71710.1| transcription elongation factor TFIIS [Drosophila virilis]
sw13299	BGIBMGA013605	1.01995	1.16855	1.66615	2.92025	2.00E-53	gi|62859519|ref|NP_001016076.1| eukaryotic translation initiation factor 4E member 2 [Xenopus tropicalis] novel protein similar to eukaryotic translation initiation factor 4E member 2 (eif4e2) [Xenopus tropicalis]
sw10058	BGIBMGA013141	0.75475	1.13215	1.15105	2.9199	No hits found	
sw09177	BGIBMGA007961	0.8957	1.69955	1.47515	2.9148	9.00E-59	gi|76608291|ref|XP_615387.2| PREDICTED: similar to Developmentally regulated RNA-binding protein 1 (RB-1) [Bos taurus]
sw15425	BGIBMGA012547	1.11695	1.2948	1.32355	2.9124	6.00E-47	gi|4753704|emb|CAB42049.1| Nmd3 protein [Drosophila melanogaster]
sw09540	BGIBMGA001920	0.7609	1.01835	0.82335	2.90675	Bmb031410	4.00E-25

sw12323	BGIBMGA003141	0.93965	1.0655	0.92035	2.9058	9.00E-56	gi|56269514|gb|AAH87353.1| Aad-A-prov protein [Xenopus laevis] alpha-aspartyl dipeptidase Alpha-aspartyl dipeptidase (Asp-specific dipeptidase) (Dipeptidase E)
sw09815	BGIBMGA007711	0.87205	1.1595	1.0248	2.9055	4.00E-13	gi|49899853|gb|AAH76885.1| MGC88947 protein [Xenopus tropicalis] MGC88947 protein [Xenopus tropicalis]
sw12338	BGIBMGA002601	2.38615	1.7417	1.23435	2.90505	No hits found	
sw14359	BGIBMGA001581	1.0146	1.0942	1.286	2.9049	7.00E-63	gi|74354109|gb|AAI02594.1| Mortality factor 4 like 2 [Bos taurus] mortality factor 4 like 2 [Bos taurus]
sw13966	BGIBMGA012520	1.0226	0.9896	1.16155	2.9037	5.00E-61	gi|17946007|gb|AAL49047.1| RE50565p [Drosophila melanogaster]
sw10979	BGIBMGA005216	1.174	1.51195	1.3552	2.90235	No hits found	
sw09861	BGIBMGA012857	1.09565	1.08055	1.02055	2.9023	Bmb037283	4.00E-23
sw04248	BGIBMGA007766	3.6876	2.844	2.26695	2.901	e-108	gi|32997080|dbj|BAC79386.1| glycerophosphoryl diester phosphodiesterase [Bombyx mori]
sw03199	BGIBMGA013120	0.7967	0.87775	0.81305	2.90045	2.00E-29	gi|5031597|ref|NP_005710.1| actin related protein 2/3 complex subunit 3 [Homo sapiens] PREDICTED: similar to Actin-related protein 2/3 complex subunit 3 (ARP2/3 complex 21 kDa subunit) (p21-ARC) isoform 1
sw11551	BGIBMGA000102	0.8571	0.88045	0.83145	2.8992	Bmb006555	7.00E-10
sw11902	BGIBMGA009775	1.84945	1.10005	1.1312	2.89855	2.00E-15	gi|3913399|sp|Q24998|CUP52_GALME Pupal cuticle protein PCP52 precursor (GMPCP52) pupal cuticule protein [Galleria mellonella]
sw06092	BGIBMGA008514	1.02765	1.024	1.07705	2.8975	1.00E-79	gi|609526|gb|AAA58743.1| chymotrypsinogen chymotrypsin
sw12396	BGIBMGA003920	0.8528	0.84435	0.65565	2.89165	1.00E-15	gi|74001269|ref|XP_850052.1| PREDICTED: similar to lipase, member I [Canis familiaris]
sw21445	BGIBMGA013862	1.8573	2.11865	0.8267	2.8915	7.00E-09	gi|76665796|ref|XP_587609.2| PREDICTED: similar to UDP-glucuronosyltransferase 2B4 precursor (UDPGT) (Hyodeoxycholic acid) (HLUG25) (UDPGTh-1) isoform 1 [Bos taurus]
sw08056	BGIBMGA002243	1.11865	1.17655	0.94905	2.89105	2.00E-56	gi|66548725|ref|XP_623164.1| PREDICTED: similar to ENSANGP00000017632 [Apis mellifera]
sw06600	BGIBMGA011753	0.67895	1.00685	0.79895	2.88895	Bmb030745	1.00E-41
sw02668	BGIBMGA008562	0.80795	1.01845	1	2.88485	Bmb046946	1.00E-14
sw20582	BGIBMGA004001	0.97395	1.17135	1.1154	2.88085	e-148	gi|1772495|emb|CAA52032.1| hydroxymethylglutaryl-CoA synthase [Blattella germanica] Hydroxymethylglutaryl-CoA synthase 1 (HMG-CoA synthase 1) (3-hydroxy-3-methylglutaryl coenzyme A synthase 1)
sw05128	BGIBMGA001008	1.361	1.00995	1.0833	2.87775	1.00E-19	gi|18860533|ref|NP_573377.1| CG8051-PA [Drosophila melanogaster] SD10554p [Drosophila melanogaster] CG8051-PA [Drosophila melanogaster]
sw07225	BGIBMGA012569	0.951	1.1153	1.0194	2.87355	4.00E-27	gi|62661030|ref|XP_344287.2| PREDICTED: similar to J domain of DnaJ-like-protein 1 - rat [Rattus norvegicus] J domain of DnaJ-like-protein 1 - rat
sw09098	BGIBMGA012728	1.1387	1.09055	1.0801	2.87275	e-110	gi|38679389|gb|AAR26516.1| antennal esterase [Mamestra brassicae]
sw15434	BGIBMGA003244	0.9499	1	1	2.87255	No hits found	
sw10412	BGIBMGA010150	1.1649	1.28135	1.27095	2.8725	2.00E-15	gi|24644274|ref|NP_649550.1| CG14671-PA [Drosophila melanogaster] CG14671-PA [Drosophila melanogaster]
sw11916	BGIBMGA006700	1.31625	1.1321	1.0831	2.87155	4.00E-16	gi|72014989|ref|XP_782823.1| PREDICTED: similar to solute carrier family 25, member 35 [Strongylocentrotus purpuratus]
sw13199	BGIBMGA013998	0.7642	0.95805	0.7933	2.8666	2.00E-44	gi|55700047|dbj|BAD69622.1| heme oxygenase [Apis mellifera] heme oxygenase [Apis mellifera]
sw01022	BGIBMGA003330	1.19345	2.03655	1	2.8663	Bmb014895	3.00E-22
sw04880	BGIBMGA006828	0.8917	1.01535	0.82915	2.86195	4.00E-75	gi|7688673|gb|AAF67477.1| putative 55 kDa protein [Homo sapiens]
sw19005	BGIBMGA003004	0.69715	0.85725	1.19785	2.8606	Bmb028789	2.00E-82

sw07489	BGIBMGA006500	0.9682	1.02025	0.9918	2.85915	5.00E-25	gi|56541182|gb|AAH87570.1| Hypothetical protein LOC496625 [Xenopus tropicalis] hypothetical protein LOC496625 [Xenopus tropicalis] novel protein containing RING finger [Xenopus tropicalis]
sw16162	BGIBMGA007613	1	1.6978	1	2.85885	1.00E-37	gi|55240040|gb|EAA10029.2| ENSANGP00000000640 [Anopheles gambiae str. PEST] ENSANGP00000000640 [Anopheles gambiae str. PEST]
sw11657	BGIBMGA003536	0.80345	1.01455	0.83785	2.85745	1.00E-23	gi|66514013|ref|XP_394387.2| PREDICTED: similar to Protein FAM20B precursor [Apis mellifera]
sw18291	BGIBMGA013676	1.0292	1.2041	0.9982	2.85545	1.00E-08	gi|30387346|ref|NP_848425.1| unknown [Choristoneura fumiferana MNPV] unknown [Choristoneura fumiferana MNPV]
sw22170	BGIBMGA003027	1.24735	1.32545	1.282	2.8524	1.00E-18	gi|50924436|ref|XP_472578.1| OSJNBa0006B20.1 [Oryza sativa (japonica cultivar-group)] OSJNBa0006B20.1 [Oryza sativa (japonica cultivar-group)]
sw14429	BGIBMGA012834	1.01075	1.1058	1.01435	2.851	6.00E-45	gi|73994713|ref|XP_863164.1| PREDICTED: similar to NAD-dependent deacetylase sirtuin-4 (SIR2-like protein 4) isoform 3 [Canis familiaris]
sw08912	BGIBMGA003590	1.2338	0.72505	0.9817	2.85	No hits found	
sw03742	BGIBMGA007424	0.93305	1.00635	1.45905	2.84965	3.00E-83	gi|55235786|gb|EAA14577.2| ENSANGP00000020940 [Anopheles gambiae str. PEST] ENSANGP00000020940 [Anopheles gambiae str. PEST]
sw08173	BGIBMGA013304	0.1102	1.05785	0.61205	2.84585	No hits found	
sw01504	BGIBMGA001273	1.23195	1.26535	1.3782	2.8458	4.00E-51	gi|68396543|ref|XP_682831.1| PREDICTED: similar to trimethyllysine hydroxylase, epsilon [Danio rerio]
sw11056	BGIBMGA007527	1.0675	1.1989	0.7888	2.84555	4.00E-10	gi|400673|sp|P31420|OMBP_MANSE Ommochrome-binding protein precursor (OBP) (YCP) ommochrome-binding protein
sw20497	BGIBMGA013985	0.91345	0.91905	0.9171	2.8431	4.00E-38	gi|66522177|ref|XP_393590.2| PREDICTED: similar to Odag-pending-prov protein [Apis mellifera]
sw14638	BGIBMGA011699	1.69525	1.28	1.08965	2.8412	No hits found	
sw03654	BGIBMGA003153	0.92385	1.06795	1.1794	2.83825	Bmb016214	2.00E-99
sw15877	BGIBMGA007493	1.2232	1.0576	0.98165	2.83415	Bmb036074	8.00E-48
sw16419	BGIBMGA008493	0.79095	1.03635	0.87555	2.83305	3.00E-14	gi|33329087|gb|AAQ09944.1| phosphonoformate immuno-associated protein 2 [Homo sapiens] LSMD1 protein [Homo sapiens] LSMD1 protein [Homo sapiens] LSMD1 protein [Homo sapiens]
sw08118	BGIBMGA013449	0.77845	0.90915	1.1347	2.82895	2.00E-25	gi|829221|emb|CAA29061.1| histone H2 A.F/Z [Strongylocentrotus purpuratus] Histone H2A variant
sw20751	BGIBMGA007261	1.22165	1.184	1.0856	2.8273	3.00E-24	gi|89257466|gb|ABD64957.1| hydrolase, NUDIX family protein [Brassica oleracea]
sw03106	BGIBMGA008525	0.79295	0.9173	0.8743	2.8264	5.00E-41	gi|24642023|ref|NP_572974.1| CG14407-PA [Drosophila melanogaster] RH03087p [Drosophila melanogaster] CG14407-PA [Drosophila melanogaster]
sw02182	BGIBMGA014475	0.9184	1.04185	0.9647	2.82585	4.00E-23	gi|50755675|ref|XP_414848.1| PREDICTED: similar to Growth factor, erv1 (S. cerevisiae)-like (augmenter of liver regeneration) [Gallus gallus]
sw04574	BGIBMGA007972	1.13345	1.1866	1.1417	2.8245	6.00E-34	gi|72088711|ref|XP_795347.1| PREDICTED: similar to chronic myelogenous leukemia tumor antigen 66 [Strongylocentrotus purpuratus]
sw12245	BGIBMGA007849	0.91025	1.11085	0.79125	2.82445	2.00E-69	gi|66534975|ref|XP_624114.1| PREDICTED: similar to Peptidylprolyl isomerase-like protein 3, isoform PPIL3b [Apis mellifera]
sw21866	BGIBMGA006264	1.04065	1.0008	0.8121	2.82135	5.00E-37	gi|66506576|ref|XP_394468.2| PREDICTED: similar to Biphenyl hydrolase-like (serine hydrolase, breast epithelial mucin-associated antigen) [Apis mellifera]
sw12242	BGIBMGA006081	1.0411	0.98785	1.2552	2.8191	3.00E-98	gi|38679389|gb|AAR26516.1| antennal esterase [Mamestra brassicae]
sw21174	BGIBMGA010227	0.8875	1.146	1.2645	2.81625	2.00E-60	gi|57506562|dbj|BAD86652.1| reverse transcriptase [Bombyx mori]
sw06572	BGIBMGA003596	0.8626	1.07595	0.98995	2.81455	9.00E-26	gi|76651259|ref|XP_589337.2| PREDICTED: similar to Autosomal Highly Conserved Protein [Bos taurus]
sw00738	BGIBMGA000339	1.1584	0.9385	0.5097	2.8127	3.00E-07	gi|47605408|sp|Q7M4E8|CUD6_SCHGR Endocuticle structural protein SgAbd-6
sw15742	BGIBMGA007962	0.82635	0.99125	0.80525	2.8109	5.00E-35	gi|49250357|gb|AAH74666.1| MGC69249 protein [Xenopus tropicalis] MGC69249 protein [Xenopus tropicalis]

sw08557	BGIBMGA014189	0.99785	1.17085	1.32215	2.80645	4.00E-55	gi|55239363|gb|EAL40125.1| ENSANGP00000029489 [Anopheles gambiae str. PEST] ENSANGP00000029489 [Anopheles gambiae str. PEST]
sw07804	BGIBMGA004644	0.95735	1.14935	1.00835	2.8039	4.00E-40	gi|73952516|ref|XP_536345.2| PREDICTED: similar to translin-associated factor X isoform 1 [Canis familiaris]
sw17943	BGIBMGA003065	1.5741	0.5357	0.66185	2.80295	3.00E-18	gi|290935|gb|AAC37204.1| cuticle protein 66 Larval/pupal rigid cuticle protein 66 precursor (HCCP66)
sw14566	BGIBMGA008982	0.8542	1.00465	0.9087	2.8022	e-124	gi|66503797|ref|XP_624046.1| PREDICTED: similar to solute carrier family 41 member 1 [Apis mellifera]
sw22014	BGIBMGA009885	1.1845	1.1234	1.54995	2.8019	e-127	gi|72014529|ref|XP_786173.1| PREDICTED: similar to DEAD (Asp-Glu-Ala-Asp) box polypeptide 47 [Strongylocentrotus purpuratus]
sw08359	BGIBMGA009817	0.6547	0.94475	0.67015	2.8016	1.00E-68	gi|55700852|dbj|BAD69791.1| Replication protein A small subunit [Bombyx mori]
sw09330	BGIBMGA013774	1.44635	1.12355	1.5468	2.7999	4.00E-27	gi|24581316|ref|NP_722867.1| CG17224-PA, isoform A [Drosophila melanogaster] CG17224-PB, isoform B [Drosophila melanogaster] CG17224-PB, isoform B
sw15691	BGIBMGA011457	1.93685	1.22695	0.85835	2.7992	2.00E-23	gi|6560645|gb|AAF16700.1| juvenile hormone binding protein precursor-like protein [Manduca sexta]
sw14228	BGIBMGA011009	1.0296	1	1.14835	2.79155	Bmb023634	6.00E-92
sw10803	BGIBMGA012262	2.1921	1.13055	0.76535	2.78335	3.00E-10	gi|27462828|gb|AAO15603.1| sensory neuron membrane protein [Mamestra brassicae]
sw15045	BGIBMGA013171	1.1032	0.65385	0.7609	2.77715	5.00E-23	gi|17224425|gb|AAL36972.1| phosphorylase kinase gamma [Homo sapiens] Phosphorylase kinase, gamma 1 (muscle) [Homo sapiens] Phosphorylase kinase, gamma 1 (muscle)
sw06207	BGIBMGA002407	0.49905	1.2055	0.8424	2.77105	3.00E-15	gi|74356259|gb|AAI04710.1| LOC500420 protein [Rattus norvegicus]
sw19267	BGIBMGA003995	0.67005	0.9288	0.52095	2.77065	2.00E-10	gi|68427052|ref|XP_687434.1| PREDICTED: similar to stimulated by retinoic acid 13 [Danio rerio]
sw18568	BGIBMGA002973	0.8514	1.01655	0.92365	2.7661	e-151	gi|87248341|gb|ABD36223.1| interphase cytoplasmic foci protein 45 [Bombyx mori]
sw12688	BGIBMGA012456	1.3023	1.5136	1.43105	2.7603	6.00E-46	gi|17861638|gb|AAL39296.1| GH16993p [Drosophila melanogaster]
sw15388	BGIBMGA010998	1.12125	1.28755	0.78255	2.7585	3.00E-32	gi|15082309|gb|AAH12060.1| Guanine nucleotide binding protein beta-subunit-like polypeptide [Homo sapiens] guanine nucleotide binding protein beta-subunit-like polypeptide [Homo sapiens] G-protein beta subunit-like protein
sw21851	BGIBMGA013220	0.6794	1.38565	1.42155	2.75175	Bmb024303	1.00E-60
sw12704	BGIBMGA009902	0.87325	0.989	0.8855	2.74625	6.00E-68	gi|87248643|gb|ABD36374.1| Wibg protein [Bombyx mori]
sw03426	BGIBMGA005533	0.995	1.0176	0.89255	2.74435	2.00E-06	gi|54644959|gb|EAL33699.1| GA13693-PA [Drosophila pseudoobscura]
sw08785	BGIBMGA005011	0.9942	1.07075	0.9266	2.7434	7.00E-06	gi|45552839|ref|NP_995945.1| CG33229-PA [Drosophila melanogaster] LD20362p [Drosophila melanogaster] CG33229-PA [Drosophila melanogaster]
sw15882	BGIBMGA011762	1.37045	1.0816	1.01765	2.7394	No hits found	
sw18206	BGIBMGA006055	0.87525	1.01585	0.7624	2.7377	0	gi|87248311|gb|ABD36208.1| dermal papilla derived protein 13 [Bombyx mori]
sw20945	BGIBMGA010303	1.14895	1.0944	1.13545	2.73285	6.00E-52	gi|2738865|gb|AAB94558.1| hemocyte protease-2 [Manduca sexta]
sw08864	BGIBMGA005939	0.89715	1.11115	0.8128	2.73255	1.00E-71	gi|48094717|ref|XP_392172.1| PREDICTED: similar to ENSANGP00000016945 [Apis mellifera]
sw21707	BGIBMGA004038	0.8866	1.09775	1.1704	2.73205	4.00E-81	gi|87248487|gb|ABD36296.1| microtubule-associated protein RP/EB family member 3 [Bombyx mori]
sw09424	BGIBMGA002501	1.00535	0.95805	0.74515	2.72905	2.00E-20	gi|74356259|gb|AAI04710.1| LOC500420 protein [Rattus norvegicus]
sw13074	BGIBMGA003719	1.59705	1.19605	1.90195	2.72675	No hits found	
sw16773	BGIBMGA013074	1	1.0652	1	2.72515	3.00E-16	gi|74353801|gb|AAI01851.1| Asparagine-linked glycosylation 6 homolog (yeast, alpha-1,3,-glucosyltransferase) [Rattus norvegicus] asparagine-linked glycosylation 6 homolog (yeast, alpha-1,3,-glucosyltransferase) [Rattus norvegicus]

sw21570	BGIBMGA004527	0.42065	2.07875	1.3684	2.72475	7.00E-27	gi|62637998|gb|AAX92638.1| glucose transporter 8 [Solenopsis invicta]
sw01530	BGIBMGA014149	1	0.76955	1.0619	2.7225	3.00E-42	gi|2970687|gb|AAC06038.1| beta-glucosidase precursor [Spodoptera frugiperda]
sw12423	BGIBMGA006751	0.8287	1.0399	1.31105	2.722	e-124	gi|87248587|gb|ABD36346.1| GTP-binding nuclear protein Ran [Bombyx mori]
sw11453	BGIBMGA012371	1	1	1	2.72095	2.00E-13	gi|11121441|emb|CAC14873.1| zinc/iron regulated transporter-related protein 1, DZIP1 protein [Drosophila melanogaster]
sw04090	BGIBMGA012496	0.8735	0.6389	0.9352	2.71875	9.00E-41	gi|1136134|gb|AAC46955.1| DmORC2
sw09876	BGIBMGA008291	1.2653	1.22725	1.12595	2.7179	Bmb037681	6.00E-35
sw20480	BGIBMGA002208	1.11415	1.0367	1.1288	2.71755	7.00E-37	gi|54638579|gb|EAL27981.1| GA20391-PA [Drosophila pseudoobscura]
sw09327	BGIBMGA008661	0.8652	0.96885	0.9886	2.717	Bmb027876	3.00E-36
sw00966	BGIBMGA010906	0.8838	0.8988	1.14165	2.71445	Bmb014069	4.00E-46
sw16619	BGIBMGA011775	0.7545	0.98095	0.8485	2.713	Bmb044249	No hits found
sw01257	BGIBMGA003303	1.2534	1.21285	0.905	2.7129	No hits found	
sw20650	BGIBMGA003462	1.06595	1.13555	1.0171	2.7119	5.00E-43	gi|72137689|ref|XP_797513.1| PREDICTED: similar to RNA binding motif protein 17 [Strongylocentrotus purpuratus]
sw09566	BGIBMGA010322	1.34975	2.16305	1.6701	2.7115	3.00E-57	gi|28317202|gb|AAO39608.1| GH22674p [Drosophila melanogaster]
sw03812	BGIBMGA007280	1.0947	1.46615	1.2448	2.7039	4.00E-23	gi|3004821|gb|AAC39088.1| putative inorganic phosphate cotransporter [Drosophila ananassae] Putative inorganic phosphate cotransporter
sw05344	BGIBMGA000922	1.107	1.0337	1.00455	2.70365	1.00E-05	gi|50761690|ref|XP_424803.1| PREDICTED: similar to mitochondrial ribosomal protein S27; mitochondrial 28S ribosomal protein S27 [Gallus gallus]
sw06559	BGIBMGA012123	1.08265	0.9453	1	2.70315	5.00E-56	gi|88946778|ref|ZP_01149846.1| cysteine synthase [Desulfotomaculum reducens MI-1] cysteine synthase [Desulfotomaculum reducens MI-1]
sw20968	BGIBMGA009469	0.95715	1.109	1.23735	2.70195	e-113	gi|4493354|emb|CAB39165.1| nucleolar protein, putative [Drosophila melanogaster]
sw08457	BGIBMGA005096	1.22905	1.14605	0.7664	2.7015	7.00E-14	gi|4090964|gb|AAD09279.1| immune-related Hdd1 [Hyphantria cunea]
sw16441	BGIBMGA009987	0.7401	1.0698	0.7422	2.7014	1.00E-14	gi|72004372|ref|XP_784178.1| PREDICTED: similar to NADH dehydrogenase (ubiquinone) 1 beta subcomplex, 7, 18kDa [Strongylocentrotus purpuratus]
sw07598	BGIBMGA004250	0.88225	1.05005	1.1466	2.70135	No hits found	
sw10316	BGIBMGA007796	0.7526	1.1086	0.68945	2.6984	2.00E-19	gi|27374248|gb|AAO01007.1| CG15863-PA [Drosophila erecta]
sw05343	BGIBMGA001221	1.11345	1.02125	0.976	2.6966	7.00E-19	gi|27374230|gb|AAO00992.1| CG9951-PA [Drosophila erecta]
sw12534	BGIBMGA000555	1.43135	0.9433	1.0677	2.6939	6.00E-25	gi|55241087|gb|EAL40600.1| ENSANGP00000027190 [Anopheles gambiae str. PEST] ENSANGP00000027190 [Anopheles gambiae str. PEST]
sw22224	BGIBMGA014599	1.6495	1.45385	1.63975	2.69265	2.00E-50	gi|66520183|ref|XP_392696.2| PREDICTED: similar to carboxylesterase [Apis mellifera]
sw09138	BGIBMGA006761	0.82945	0.99	0.91675	2.69045	1.00E-29	gi|60552380|gb|AAH91052.1| MGC108292 protein [Xenopus tropicalis] MGC108292 protein [Xenopus tropicalis] novel protein [Xenopus tropicalis]
sw18970	BGIBMGA000498	1.21205	1.64615	1	2.68815	2.00E-20	gi|38176144|gb|AAR13011.1| clock [Danaus plexippus]
sw03051	BGIBMGA009043	1.78365	1.2098	1.7273	2.68585	7.00E-64	gi|73971831|ref|XP_854862.1| PREDICTED: similar to Glyoxylate reductase/hydroxypyruvate reductase [Canis familiaris]
sw09429	BGIBMGA002394	0.87505	1.0975	1.32725	2.68055	1.00E-32	gi|50369534|gb|AAH76042.1| Metap1 protein [Danio rerio]

sw12031	BGIBMGA007005	1.02945	0.90165	0.89675	2.6802	3.00E-17	gi|27370858|gb|AAH41215.1| MGC52693 protein [Xenopus laevis] apyrase [Xenopus laevis]
sw15847	BGIBMGA003466	0.7652	0.92435	0.7104	2.67485	Bmb035873	6.00E-11
sw03160	BGIBMGA013274	0.94245	0.88725	0.82915	2.67365	1.00E-32	gi|85857490|gb|ABC86281.1| RE09672p [Drosophila melanogaster]
sw19365	BGIBMGA010098	0.3586	0.7338	0.84295	2.6727	2.00E-32	gi|17864686|ref|NP_525007.1| UDP-glycosyltransferase 37c1 CG8652-PA [Drosophila melanogaster] EG:EG0003.4 [Drosophila melanogaster] CG8652-PA [Drosophila melanogaster]
sw20309	BGIBMGA005439	0.89935	0.86295	0.77505	2.66875	3.00E-68	gi|83638667|gb|AAI09717.1| NADH dehydrogenase (ubiquinone) Fe-S protein 7, 20kDa [Bos taurus] NADH dehydrogenase (ubiquinone) Fe-S protein 7, 20kDa
sw06772	BGIBMGA011509	0.92775	1.2461	0.9406	2.6677	Bmb034208	2.00E-33
sw07509	BGIBMGA001188	0.9717	1.028	0.98545	2.6676	3.00E-67	gi|66529825|ref|XP_624000.1| PREDICTED: similar to Exosome complex exonuclease RRP43 (Ribosomal RNA processing protein 43) (Exosome component 8) (p9) (Opa-interacting protein 2) [Apis mellifera]
sw06240	BGIBMGA011822	0.98065	1.07085	1.1869	2.66615	0	gi|72106184|ref|XP_790294.1| PREDICTED: similar to guanine nucleotide binding protein-like 2 (nucleolar) [Strongylocentrotus purpuratus]
sw13043	BGIBMGA011820	1.41415	1.07185	1.06015	2.6623	Bmb015947	4.00E-48
sw18743	BGIBMGA003456	1.28995	1.40715	0.97965	2.66135	3.00E-34	gi|73999552|ref|XP_535120.2| PREDICTED: similar to lactate dehydrogenase A -like [Canis familiaris]
sw03236	BGIBMGA005544	0.7791	0.9581	0.90535	2.6582	4.00E-18	gi|72109917|ref|XP_793600.1| PREDICTED: similar to Rab geranylgeranyltransferase, beta subunit [Strongylocentrotus purpuratus]
sw14542	BGIBMGA006118	0.92755	1.0159	0.9842	2.65725	No hits found	
sw03443	BGIBMGA010459	1.02285	1.1099	1.401	2.657	3.00E-08	gi|50761351|ref|XP_424699.1| PREDICTED: similar to arrestin domain containing 3 [Gallus gallus]
sw12075	BGIBMGA008398	1.3236	1.3255	0.8944	2.65155	8.00E-06	gi|9964462|ref|NP_064930.1| putative core protein [Amsacta moorei entomopoxvirus] AMV148 [Amsacta moorei entomopoxvirus]
sw21098	BGIBMGA003658	0.90435	1.05615	1.1157	2.64815	1.00E-49	gi|50370224|gb|AAH76913.1| MGC89088 protein [Xenopus tropicalis] MGC89088 protein [Xenopus tropicalis]
sw11644	BGIBMGA002809	0.87235	1.1026	0.88895	2.6478	1.00E-11	gi|25012983|gb|AAN71576.1| RH42446p [Drosophila melanogaster]
sw17190	BGIBMGA011501	0.74675	1.07105	0.812	2.64555	3.00E-14	gi|66508472|ref|XP_624484.1| PREDICTED: similar to CG2046-PA [Apis mellifera]
sw09003	BGIBMGA008459	1.38835	1.4287	1.03675	2.6422	e-112	gi|7758|emb|CAA41263.1| crn [Drosophila melanogaster]
sw15238	BGIBMGA010627	1.13295	0.6917	0.77695	2.64055	Bmb031258	No hits found
sw18021	BGIBMGA002948	0.996	1.10075	1.10435	2.63925	Bmb023843	1.00E-18
sw13455	BGIBMGA013100	0.8726	0.9892	1.28455	2.6363	4.00E-42	gi|74007795|ref|XP_534749.2| PREDICTED: similar to small nuclear ribonucleoprotein D3 [Canis familiaris]
sw17156	BGIBMGA000931	0.99775	1.12355	0.9198	2.6357	e-123	gi|25013002|gb|AAN71585.1| RH46192p [Drosophila melanogaster]
sw13946	BGIBMGA001506	1.1311	2.08765	2.1563	2.63455	2.00E-60	gi|72172203|gb|AAZ66799.1| lipase [Samia cynthia ricini] lipase-1 [Bombyx mori] lipase [Bombyx mori]
sw11846	BGIBMGA000250	1.3523	1.39345	1.40135	2.63295	2.00E-27	gi|24666794|ref|NP_649121.1| CG9290-PA [Drosophila melanogaster] CG9290-PA [Drosophila melanogaster]
sw22385	BGIBMGA010824	0.90455	0.57225	0.7441	2.63295	No hits found	
sw07544	BGIBMGA013713	0.36805	0.8332	0.788	2.63	3.00E-21	gi|31324444|gb|AAP47228.1| Sec61p gamma subunit [Gryllotalpa orientalis] Protein transport protein SEC61 gamma subunit
sw04230	BGIBMGA010017	1.0163	1.01555	1.22315	2.6263	3.00E-77	gi|87248299|gb|ABD36202.1| COMMD4 protein [Bombyx mori]
sw04425	BGIBMGA012348	0.67655	1.09585	1.2681	2.6233	Bmb034386	1.00E-28

sw01563	BGIBMGA009760	0.5754	0.7759	0.8862	2.6227	4.00E-84	gi|87248373|gb|ABD36239.1| nonclathrin coat protein zeta 1-COP [Bombyx mori]
sw14729	BGIBMGA005936	1.1297	1.1862	1.0978	2.62255	No hits found	
sw22885	BGIBMGA006723	0.9003	1.37795	1.19165	2.621	5.00E-24	gi|56269559|gb|AAH87483.1| LOC496069 protein [Xenopus laevis]
sw12065	BGIBMGA007864	0.7271	0.6181	0.9974	2.6208	1.00E-55	gi|72021217|ref|XP_795004.1| PREDICTED: similar to GLIS family zinc finger 2 [Strongylocentrotus purpuratus]
sw09947	BGIBMGA001265	0.79585	0.9675	1.21435	2.6198	No hits found	
sw17077	BGIBMGA004734	1.09235	1.0698	0.9154	2.61615	1.00E-16	gi|5809683|gb|AAD40352.2| RP140-upstream [Drosophila melanogaster]
sw20245	BGIBMGA001372	1.4071	1.28445	1.52265	2.61495	1.00E-51	gi|38679389|gb|AAR26516.1| antennal esterase [Mamestra brassicae]
sw12542	BGIBMGA012452	1.4982	0.85765	0.9194	2.61475	5.00E-59	gi|54639776|gb|EAL29178.1| GA14959-PA [Drosophila pseudoobscura]
sw14514	BGIBMGA007239	1.43545	1.1856	1.5324	2.6101	Bmb025879	e-159
sw09645	BGIBMGA004059	0.7145	0.76295	0.89315	2.6074	Bmb033292	2.00E-40
sw08543	BGIBMGA005547	0.7449	0.877	1.1365	2.6012	Bmb016029	6.00E-05
sw00511	BGIBMGA000921	0.8692	0.966	0.78805	2.6004	1.00E-26	gi|85857748|gb|ABC86409.1| IP09454p [Drosophila melanogaster]
sw09864	BGIBMGA002754	1.3072	1.0372	1.05735	2.5984	No hits found	
sw05029	BGIBMGA001918	0.79345	0.96085	0.85155	2.5979	4.00E-95	gi|72004713|ref|XP_785235.1| PREDICTED: similar to phosphatidylinositol glycan, class K (predicted) [Strongylocentrotus purpuratus]
sw11034	BGIBMGA010172	0.8264	0.96515	0.79255	2.59665	1.00E-40	gi|458674|gb|AAC46479.1| TFIID 22 kDa subunit
sw20207	BGIBMGA000305	0.73605	0.9895	0.8516	2.5966	8.00E-66	gi|57506562|dbj|BAD86652.1| reverse transcriptase [Bombyx mori]
sw13235	BGIBMGA005559	0.7564	1.0194	1.0977	2.5963	1.00E-58	gi|66533332|ref|XP_625023.1| PREDICTED: similar to ARMET-like protein precursor [Apis mellifera]
sw03695	BGIBMGA010450	0.9086	1.03075	0.9245	2.59545	No hits found	
sw13320	BGIBMGA010325	1.06055	1.03885	1.05545	2.59295	Bmb017661	2.00E-24
sw22830	BGIBMGA005485	1.12465	1.25555	0.86575	2.5913	Bmb048151	4.00E-18
sw03365	BGIBMGA004830	1.57225	1.4632	1.4773	2.5897	2.00E-90	gi|74831719|emb|CAJ30028.1| carboxypeptidase B precursor [Helicoverpa zea]
sw15675	BGIBMGA010533	1.1621	0.69145	0.819	2.5862	No hits found	
sw14565	BGIBMGA008983	0.85765	0.8998	1.0321	2.5808	6.00E-19	gi|266685|sp|P08461|ODP2_RAT Dihydrolipoyllysine-residue acetyltransferase component of pyruvate dehydrogenase complex (E2) (Dihydrolipoamide acetyltransferase component of pyruvate dehydrogenase complex)
sw15717	BGIBMGA002330	1.0664	1.158	0.7962	2.5797	4.00E-79	gi|87248205|gb|ABD36155.1| proline synthetase co-transcribed bacterial-like protein [Bombyx mori]
sw07840	BGIBMGA001209	1.00695	1.09295	0.95845	2.5746	2.00E-46	gi|62857779|ref|NP_001017238.1| COP9 constitutive photomorphogenic homolog subunit 3 [Xenopus tropicalis] COP9 constitutive photomorphogenic homolog subunit 3 (Arabidopsis) [Xenopus tropicalis]
sw22940	BGIBMGA011742	0.5229	0.9643	0.87655	2.5742	No hits found	
sw10552	BGIBMGA006840	0.6821	0.9187	1.1223	2.5722	3.00E-57	gi|87248491|gb|ABD36298.1| eukaryotic translation initiation factor 1A [Bombyx mori]
sw15011	BGIBMGA007723	0.7364	0.72785	0.8108	2.5673	2.00E-64	gi|10443892|gb|AAG17630.1| esterase D [Sus scrofa] esterase D [Sus scrofa] Esterase D esterase D [Sus scrofa]

sw05520	BGIBMGA003988	1.06065	1.24775	1.1147	2.56685	No hits found	
sw15490	BGIBMGA002064	0.99615	1.20675	1.0183	2.565	1.00E-09	gi|55620992|ref|XP_526288.1| PREDICTED: similar to adenylate cyclase 5; adenylyl cyclase type V [Pan troglodytes]
sw12099	BGIBMGA002943	0.9217	0.92815	1.1606	2.56225	1.00E-20	gi|56118552|ref|NP_001007992.1| hpgd-prov protein [Xenopus tropicalis] Hpgd-prov protein [Xenopus tropicalis]
sw03003	BGIBMGA010141	0.78245	0.97275	1	2.56215	8.00E-17	gi|87248419|gb|ABD36262.1| larval cuticle protein [Bombyx mori]
sw19438	BGIBMGA011975	1.15365	1.4438	1.3214	2.56205	2.00E-06	gi|54641702|gb|EAL30452.1| GA21682-PA [Drosophila pseudoobscura]
sw10834	BGIBMGA007944	1.3197	1.1197	1.16735	2.5605	3.00E-81	gi|66555732|ref|XP_623838.1| PREDICTED: similar to Zgc:92633 [Apis mellifera]
sw17661	BGIBMGA008016	1.49455	0.8778	0.8958	2.5569	4.00E-27	gi|48096076|ref|XP_392394.1| PREDICTED: similar to WAP four-disulfide core domain 2 [Apis mellifera] PREDICTED: similar to WAP four-disulfide core domain 2 [Apis mellifera]
sw13779	BGIBMGA003236	0.81055	1.1993	1.00395	2.55615	2.00E-14	gi|50730609|ref|XP_416969.1| PREDICTED: similar to cDNA sequence BC006662 [Gallus gallus]
sw08964	BGIBMGA001766	1.5144	1.0683	1.17375	2.5555	1.00E-75	gi|72159781|ref|XP_798408.1| PREDICTED: similar to phosphatidylserine decarboxylase [Strongylocentrotus purpuratus]
sw14879	BGIBMGA011458	2.3441	1.43735	1.63085	2.5535	2.00E-40	gi|505621|gb|AAA21588.1| high affinity nuclear juvenile hormone binding protein
sw09721	BGIBMGA006739	0.9123	1.1519	1.20065	2.55235	No hits found	
sw05062	BGIBMGA005455	1.12675	0.96825	0.934	2.55115	4.00E-13	gi|51709682|ref|XP_485502.1| PREDICTED: similar to coiled-coil-helix-coiled-coil-helix domain containing 2 [Mus musculus] PREDICTED: similar to coiled-coil-helix-coiled-coil-helix domain containing 2 [Mus musculus]
sw03386	BGIBMGA004871	1.2034	1.50605	1.25075	2.54745	Bmb010006	No hits found
sw03480	BGIBMGA005494	1.0119	1.2789	0.96785	2.54675	1.00E-07	gi|75704935|gb|ABA24611.1| ABC-type phosphate/phosphonate transport system periplasmic component-like
sw16427	BGIBMGA012936	0.98945	1.3559	1.3518	2.54615	2.00E-10	gi|66556093|ref|XP_397089.2| PREDICTED: similar to CG3224-PA [Apis mellifera]
sw20893	BGIBMGA003440	0.8657	0.843	0.88935	2.545	2.00E-61	gi|56118905|ref|NP_001008042.1| MGC79577 protein [Xenopus tropicalis] MGC79577 protein [Xenopus tropicalis]
sw14278	BGIBMGA007219	1.21935	1.17865	1.3716	2.5375	4.00E-08	gi|62656459|ref|XP_220582.3| PREDICTED: similar to ubiquitin specific protease 43 [Rattus norvegicus]
sw12863	BGIBMGA010210	0.7222	0.96735	1.013	2.53605	5.00E-32	gi|56269224|gb|AAH87482.1| LOC496068 protein [Xenopus laevis]
sw10448	BGIBMGA008074	1.01585	0.90745	0.8168	2.5312	1.00E-08	gi|4505797|ref|NP_002634.1| phosphatidylinositol glycan, class F isoform 1 [Homo sapiens] Phosphatidylinositol glycan, class F, isoform 1 [Homo sapiens] PIGF
sw15029	BGIBMGA012719	0.7848	0.987	1.2037	2.5312	Bmb029558	2.00E-08
sw09668	BGIBMGA003014	1.03705	0.82735	0.67155	2.5296	7.00E-16	gi|15222687|ref|NP_173958.1| B5 #6 [Arabidopsis thaliana] putative cytochrome b5 protein [Arabidopsis thaliana] cytochrome b5 [Arabidopsis thaliana] cytochrome b5
sw20302	BGIBMGA000287	0.933	1.1841	1.14595	2.5286	2.00E-48	gi|50926880|gb|AAH78974.1| Osgepl1 protein [Rattus norvegicus]
sw21955	BGIBMGA013567	1.39025	1.25035	1.2365	2.5275	0	gi|18253047|gb|AAL62468.1| ribosomal protein L3 [Spodoptera frugiperda]
sw20598	BGIBMGA004800	2.03385	2.15015	1.67795	2.52615	2.00E-90	gi|57506558|dbj|BAD86650.1| reverse transcriptase [Bombyx mori]
sw13853	BGIBMGA011074	1.66445	1.1724	1.5427	2.525	9.00E-14	gi|66550509|ref|XP_624977.1| PREDICTED: similar to interferon gamma-inducible protein 30 [Apis mellifera]
sw14742	BGIBMGA013269	2.84135	1.51605	1.00965	2.5249	1.00E-35	gi|66499360|ref|XP_396507.2| PREDICTED: similar to CG11550-PA [Apis mellifera]
sw14040	BGIBMGA001536	0.8994	1.15275	0.94105	2.5227	8.00E-88	gi|76639590|ref|XP_580699.2| PREDICTED: similar to Beta-ureidopropionase (Beta-alanine synthase) (N-carbamoyl-beta-alanine amidohydrolase) (BUP-1) [Bos taurus]
sw03701	BGIBMGA010032	0.772	1	1.00175	2.5219	7.00E-11	gi|21594291|gb|AAH32101.1| Jagunal homolog 1 [Homo sapiens]

sw19175	BGIBMGA008280	0.6639	0.82295	0.77955	2.5218	4.00E-38	gi|60735591|gb|AAX35812.1| midgut chymotrypsin [Spodoptera exigua]
sw09581	BGIBMGA003430	0.94195	0.857	0.7378	2.5203	No hits found	
sw13071	BGIBMGA011456	2.44405	0.8438	1.4277	2.5197	3.00E-10	gi|70905642|gb|AAZ14281.1| proteophosphoglycan 5 [Leishmania major strain Friedlin] Leishmania major strain Friedlin proteophosphoglycan 5 [Leishmania major strain Friedlin]
sw15879	BGIBMGA012025	1.4312	1.44445	1.45095	2.5154	9.00E-37	gi|62859003|ref|NP_001017054.1| PAK1 interacting protein 1 [Xenopus tropicalis]
sw04389	BGIBMGA008671	0.9382	1.17755	0.89555	2.5121	7.00E-75	gi|2570798|gb|AAB82275.1| cyclin H [Drosophila melanogaster]
sw09140	BGIBMGA000440	1.1209	1.1479	0.91705	2.51055	2.00E-34	gi|72124941|ref|XP_793194.1| PREDICTED: similar to mitochondrial ribosomal protein L47 [Strongylocentrotus purpuratus]
sw20657	BGIBMGA009800	1.07005	0.86935	0.871	2.50665	Bmb035870	8.00E-34
sw07808	BGIBMGA004333	1.02385	1.1245	1.0191	2.5054	2.00E-19	gi|35193192|gb|AAH58574.1| Dystrobrevin binding protein 1 [Mus musculus] dystrobrevin binding protein 1 [Mus musculus] Dystrobrevin binding protein 1
sw15040	BGIBMGA013693	0.87775	0.9875	0.96005	2.50375	3.00E-15	gi|20306641|gb|AAH28523.1| Ict1 protein [Mus musculus] Immature colon carcinoma transcript 1 protein precursor
sw16055	BGIBMGA001578	0.9529	1.20335	1.0424	2.5022	Bmb037848	6.00E-24
sw08635	BGIBMGA003028	0.7106	0.8574	0.8753	2.5002	e-101	gi|87248385|gb|ABD36245.1| proteasome beta-subunit [Bombyx mori]
sw09737	BGIBMGA006975	0.8017	0.9009	1.08255	2.4999	Bmb034988	6.00E-42
sw10850	BGIBMGA013703	1.0191	1.18615	1.0361	2.4993	7.00E-40	gi|50759728|ref|XP_417753.1| PREDICTED: similar to CGI-94 protein; comparative gene identification transcript 94 [Gallus gallus]
sw04240	BGIBMGA000381	0.882	1.0049	1.00255	2.49885	2.00E-10	gi|87248553|gb|ABD36329.1| nuclear migration protein nudC [Bombyx mori]
sw11361	BGIBMGA012068	1.22505	0.82295	1.05535	2.4977	3.00E-11	gi|62751745|ref|NP_001015768.1| MGC108303 protein [Xenopus tropicalis] MGC108303 protein [Xenopus tropicalis]
sw22855	BGIBMGA014416	1.11415	0.92345	1.06525	2.4961	4.00E-07	gi|76262498|gb|ABA41399.1| pherophorin-C1 protein precursor [Chlamydomonas reinhardtii]
sw06858	BGIBMGA011718	0.9611	1.13165	0.73145	2.49585	No hits found	
sw08546	BGIBMGA011455	1.3573	0.59905	0.97615	2.49175	No hits found	
sw10814	BGIBMGA004073	1.10285	0.72125	0.6911	2.4914	No hits found	
sw06776	BGIBMGA003962	1.1897	1.2387	1.13175	2.4896	1.00E-07	gi|58395480|ref|XP_321280.2| ENSANGP00000008441 [Anopheles gambiae str. PEST] ENSANGP00000008441 [Anopheles gambiae str. PEST]
sw09363	BGIBMGA009985	1.06065	1.0518	0.82385	2.4895	2.00E-24	gi|49522321|gb|AAH75305.1| Brain and reproductive organ-expressed (TNFRSF1A modulator) [Xenopus tropicalis] brain and reproductive organ-expressed (TNFRSF1A modulator) [Xenopus tropicalis]
sw12802	BGIBMGA013641	1.10945	1.31605	1.11665	2.4894	4.00E-20	gi|17901861|gb|AAL47714.1| synaptic vesicle protein 2B [Rattus norvegicus] synaptic vesicle glycoprotein 2b [Rattus norvegicus] synaptic vesicle protein 2B
sw22797	BGIBMGA000239	1.306	1	1.31655	2.4883	Bmb043445	No hits found
sw04649	BGIBMGA013375	1.6956	0.97145	1.3158	2.48755	Bmb041349	No hits found
sw13451	BGIBMGA003404	0.484	1.24305	1.0731	2.48725	Bmb018503	1.00E-15
sw07381	BGIBMGA004055	0.94385	1.1345	0.9608	2.4871	3.00E-47	gi|20330554|gb|AAM19101.1| PRP4 kinase [Homo sapiens] serine/threonine-protein kinase [Homo sapiens] Serine/threonine-protein kinase PRP4 homolog (PRP4 pre-mRNA-processing factor 4 homolog) (PRP4 kinase)
sw01751	BGIBMGA012490	1.2474	1.314	1.31585	2.48705	No hits found	
sw08671	BGIBMGA008732	1.00915	1.2958	1.0188	2.4869	No hits found	

sw13204	BGIBMGA007669	1.06795	1.23485	1.2978	2.48555	9.00E-39	gi|66554920|ref|XP_395755.2| PREDICTED: similar to estrogen receptor binding protein [Apis mellifera]
sw07664	BGIBMGA011093	0.965	1.2346	1.18425	2.4837	2.00E-55	gi|1934852|emb|CAA73031.1| putative organic cation transporter [Drosophila melanogaster]
sw13824	BGIBMGA003855	1.2189	0.7727	1.0059	2.48285	No hits found	
sw15808	BGIBMGA013786	1.1918	1.0391	1.0639	2.48235	Bmb035627	5.00E-13
sw09073	BGIBMGA004657	0.67855	0.7135	0.7093	2.4815	4.00E-65	gi|50748430|ref|XP_421242.1| PREDICTED: similar to Proteasome subunit alpha type 6 (Proteasome iota chain) (Macropain iota chain) (Multicatalytic endopeptidase complex iota chain) [Gallus gallus]
sw07946	BGIBMGA000875	1.1881	1.3617	1.47805	2.4811	3.00E-63	gi|38679389|gb|AAR26516.1| antennal esterase [Mamestra brassicae]
sw15426	BGIBMGA010010	0.9272	0.47635	0.86345	2.4807	8.00E-47	gi|87248401|gb|ABD36253.1| sericotropin [Bombyx mori]
sw13773	BGIBMGA004306	0.83125	0.9389	0.76405	2.4801	No hits found	
sw13194	BGIBMGA003325	1.1829	1.50055	1.18355	2.47865	No hits found	
sw11751	BGIBMGA006530	0.67635	0.8655	0.78905	2.4784	Bmb007822	No hits found
sw06973	BGIBMGA014622	1.4251	1.1281	1.4232	2.47775	7.00E-14	gi|73975357|ref|XP_539297.2| PREDICTED: similar to UDP-glucuronosyltransferase 2A1 precursor, microsomal [Canis familiaris]
sw10526	BGIBMGA000627	0.882	1.0975	0.88945	2.47395	e-100	gi|22474521|dbj|BAC10625.1| ubiquitin conjugating enzyme-like protein [Bombyx mori] ubiquitin conjugating enzyme-like protein [Bombyx mori]
sw03131	BGIBMGA009732	0.86435	0.7711	1.0642	2.47295	e-145	gi|66519017|ref|XP_393258.2| PREDICTED: similar to gARPX [Apis mellifera]
sw10652	BGIBMGA012761	1.03195	0.93345	1.0812	2.4694	e-100	gi|24641702|ref|NP_572865.1| CG2453-PA [Drosophila melanogaster] CG2453-PA [Drosophila melanogaster] Ubiquinone biosynthesis methyltransferase COQ5, mitochondrial precursor
sw05464	BGIBMGA013414	1	1	1	2.4683	No hits found	
sw13472	BGIBMGA013491	1.09075	1.06145	0.92675	2.46795	3.00E-40	gi|66525531|ref|XP_624212.1| PREDICTED: similar to Nucleoporin Nup43 (p42) [Apis mellifera]
sw16263	BGIBMGA006887	0.9271	0.9869	0.93835	2.46545	Bmb039770	1.00E-05
sw16529	BGIBMGA000361	1.1866	1.18895	0.9078	2.4627	Bmb042787	No hits found
sw08533	BGIBMGA010576	1	1.06105	1	2.46215	e-103	gi|158001|gb|AAA28730.1| numb peptide (put.); putative
sw08430	BGIBMGA010740	0.75325	1.033	1.9917	2.46195	Bmb014557	e-117
sw17132	BGIBMGA005126	0.7895	0.9502	1.1252	2.4587	1.00E-65	gi|6636388|gb|AAF20167.1| tryptophanyl-tRNA synthetase [Drosophila melanogaster]
sw01098	BGIBMGA001465	1.2643	1.47385	1.6359	2.4584	No hits found	
sw03653	BGIBMGA003165	0.95445	0.98795	0.94965	2.45745	e-134	gi|87248663|gb|ABD36384.1| transmembrane BAX inhibitor motif-containing protein 5 [Bombyx mori]
sw01302	BGIBMGA012247	0.8353	0.8109	0.8377	2.4554	4.00E-53	gi|3367669|emb|CAA19735.1| EG:73D1.1 [Drosophila melanogaster]
sw09643	BGIBMGA006822	1.09555	1.4226	0.84605	2.453	7.00E-19	gi|33186814|tpe|CAD67592.1| TPA: putative C6.1A-like protease [Mus musculus] C6.1A-like [Mus musculus]
sw09557	BGIBMGA006260	1	1.1749	1.3798	2.45075	7.00E-27	gi|28317322|gb|AAO39657.1| AT01548p [Drosophila melanogaster]
sw08391	BGIBMGA001550	0.82105	0.89435	0.9106	2.4451	8.00E-18	gi|1705720|sp|P51953|CDK7_CARAU Cell division protein kinase 7 (40 kDa protein kinase) (P40 MO15) (CDC2/CDK2,4-activating kinase) MO15(cdk7) kinase [Carassius auratus] cdc2-related protein p40 MO15
sw07488	BGIBMGA006498	0.8926	1.1184	1	2.44455	2.00E-05	gi|50748926|ref|XP_421460.1| PREDICTED: similar to chromosome 14 open reading frame 138 [Gallus gallus]

sw12728	BGIBMGA009042	0.728	1.16635	0.89655	2.4426	1.00E-09	gi|5852418|gb|AAD54066.1| putative 2-hydroxyacid dehydrogenase [Homo sapiens]
sw05413	BGIBMGA007413	0.8786	1.02235	0.8544	2.4385	e-104	gi|50737124|ref|XP_419160.1| PREDICTED: similar to sudD suppressor of bimD6 homolog isoform 1; homolog of the Aspergillus nidulans sudD gene product
sw06853	BGIBMGA006056	1.0273	0.94045	1.28615	2.433	4.00E-29	gi|50746777|ref|XP_420650.1| PREDICTED: similar to nucleolar protein family A, member 1; H/ACA small nucleolar RNPs [Gallus gallus]
sw10865	BGIBMGA001022	1.2468	1.08635	1.17635	2.42775	7.00E-12	gi|758796|gb|AAC46959.1| Sh23 [Schistosoma haematobium] 23 kDa integral membrane protein (Sh23)
sw14229	BGIBMGA013895	0.9117	0.9107	1.1458	2.42715	Bmb023648	No hits found
sw09682	BGIBMGA003552	1.28875	1.09955	1.1482	2.4257	No hits found	
sw15939	BGIBMGA001217	0.9121	1.05805	0.96765	2.41805	1.00E-83	gi|25013002|gb|AAN71585.1| RH46192p [Drosophila melanogaster]
sw10402	BGIBMGA005926	0.7454	0.9876	0.8976	2.41755	1.00E-61	gi|62652296|ref|XP_576244.1| PREDICTED: phosphatidylserine synthase 1 (predicted) [Rattus norvegicus]
sw05328	BGIBMGA004646	0.9441	1.0282	1.27845	2.41655	1.00E-42	gi|72015359|ref|XP_780636.1| PREDICTED: similar to budding uninhibited by benzimidazoles 3 homolog isoform 1 [Strongylocentrotus purpuratus]
sw16069	BGIBMGA008416	0.89735	0.8564	0.9172	2.412	2.00E-24	gi|1314248|gb|AAA99718.1| NADH:cytochrome c reductase
sw01248	BGIBMGA009750	1.43475	1.3775	1.53135	2.4111	5.00E-19	gi|158512|gb|AAA28918.1| type II transmembrane protein
sw10878	BGIBMGA010285	1.51855	1.08915	1.03225	2.41055	1.00E-83	gi|32029913|ref|ZP_00132857.1| COG0451: Nucleoside-diphosphate-sugar epimerases [Haemophilus somnus 2336] COG0451: Nucleoside-diphosphate-sugar epimerases [Haemophilus somnus 129PT]
sw14403	BGIBMGA007889	0.8986	1.1072	1.04815	2.41055	e-123	gi|50759828|ref|XP_417800.1| PREDICTED: similar to Eukaryotic translation initiation factor 3 subunit 2 (eIF-3 beta) (eIF3 p36) (eIF3i) (TGF-beta receptor interacting protein 1) (TRIP-1) [Gallus gallus]
sw04364	BGIBMGA008096	0.7581	0.69685	0.95475	2.4095	e-105	gi|87248227|gb|ABD36166.1| short-chain dehydrogenease/reductase 2 [Bombyx mori]
sw13402	BGIBMGA001115	0.904	1.2773	1.06385	2.40795	Bmb018159	2.00E-44
sw06424	BGIBMGA011079	0.374	1.59225	0.24925	2.40665	3.00E-07	gi|6560645|gb|AAF16700.1| juvenile hormone binding protein precursor-like protein [Manduca sexta]
sw06402	BGIBMGA007256	1.65215	1.42945	0.9402	2.406	1.00E-74	gi|66516898|ref|XP_391826.2| PREDICTED: similar to yellow [Apis mellifera]
sw08850	BGIBMGA009355	0.9743	1.13865	0.84715	2.40515	5.00E-43	gi|55236609|gb|EAA13645.2| ENSANGP00000015954 [Anopheles gambiae str. PEST] ENSANGP00000015954 [Anopheles gambiae str. PEST]
sw01649	BGIBMGA009063	0.59135	0.5273	0.73125	2.4049	1.00E-37	gi|66517091|ref|XP_397364.2| PREDICTED: similar to ENSANGP00000027134 [Apis mellifera]
sw08409	BGIBMGA010653	0.83485	0.9485	0.90255	2.40405	5.00E-10	gi|38505259|ref|NP_081247.2| mitochondrial ribosomal protein L48 isoform 1 [Mus musculus]
sw04971	BGIBMGA011776	0.6742	1.02205	0.93225	2.40225	3.00E-15	gi|62859829|ref|NP_001017011.1| programmed cell death 5 [Xenopus tropicalis]
sw14663	BGIBMGA005158	0.7342	0.9889	0.7119	2.40195	Bmb026823	2.00E-44
sw07939	BGIBMGA003088	1.14795	1.42925	1.0263	2.4019	No hits found	
sw08909	BGIBMGA005205	1.0697	0.88905	1.192	2.40175	9.00E-24	gi|3929381|sp|Q24491|RX21_DROME RNA binding protein Rsf1 (RNA binding protein Rox21) RNA binding protein
sw08373	BGIBMGA001476	1.08225	1.2534	0.72125	2.40115	Bmb013847	3.00E-86
sw22877	BGIBMGA010595	0.9663	1.06165	1.19205	2.40045	1.00E-88	gi|73996969|ref|XP_851691.1| PREDICTED: similar to heat shock 70kD protein binding protein [Canis familiaris]
sw13266	BGIBMGA008342	1.0062	1.1024	1.1623	2.39865	e-141	gi|46249602|gb|AAH68838.1| MGC81483 protein [Xenopus laevis] NEDD8-activating enzyme E1 regulatory subunit (Amyloid protein-binding protein 1) (APP-BP1)
sw15132	BGIBMGA003335	0.6283	0.74015	0.71225	2.39805	2.00E-73	gi|2055299|dbj|BAA19760.1| proteasome subunit Y [Xenopus laevis]

sw13806	BGIBMGA014209	1.0371	1.156	1.1833	2.39775	3.00E-83	gi|55242911|gb|EAA07041.2| ENSANGP00000016729 [Anopheles gambiae str. PEST] ENSANGP00000016729 [Anopheles gambiae str. PEST]
sw15265	BGIBMGA001964	0.95475	1.2052	1.05325	2.3975	2.00E-72	gi|28317060|gb|AAO39549.1| RE03380p [Drosophila melanogaster]
sw11068	BGIBMGA010147	0.8696	1.16595	1.291	2.3972	5.00E-48	gi|37595364|gb|AAQ94568.1| potassium channel modulatory factor 1 [Danio rerio] Similar to potassium channel modulatory factor 1 [Danio rerio] potassium channel modulatory factor 1 [Danio rerio]
sw08127	BGIBMGA013114	0.542	0.7798	0.8022	2.39615	2.00E-17	gi|34883246|ref|XP_347254.1| PREDICTED: similar to Williams-Beuren syndrome critical region protein 21 [Rattus norvegicus] PREDICTED: similar to Williams-Beuren syndrome critical region protein 21 [Rattus norvegicus]
sw22975	BGIBMGA008095	0.7365	0.74645	0.77405	2.39555	e-141	gi|87248227|gb|ABD36166.1| short-chain dehydrogenease/reductase 2 [Bombyx mori]
sw18289	BGIBMGA007842	1.1387	1.13865	1.34265	2.39445	2.00E-44	gi|68356394|ref|XP_707135.1| PREDICTED: similar to SKB1 homolog isoform 3 [Danio rerio]
sw10858	BGIBMGA001938	0.71895	1.05685	0.7663	2.3942	Bmb002061	2.00E-11
sw00530	BGIBMGA001307	0.70065	0.6014	0.70435	2.3935	1.00E-85	gi|40949813|gb|AAR97568.1| Cu/Zn SOD [Bombyx mori] Superoxide dismutase [Cu-Zn]
sw15224	BGIBMGA004496	0.80965	1	1	2.3929	3.00E-57	gi|72073999|ref|XP_791465.1| PREDICTED: similar to Tetratricopeptide repeat protein 4 (TPR repeat protein 4), partial [Strongylocentrotus purpuratus]
sw18176	BGIBMGA001303	0.6632	0.77195	0.78345	2.3922	1.00E-45	gi|50746955|ref|XP_420692.1| PREDICTED: similar to tumor suppressor candidate 3 isoform b [Gallus gallus]
sw05172	BGIBMGA012707	1.2016	0.8592	1.21955	2.39055	4.00E-24	gi|74006595|ref|XP_859139.1| PREDICTED: similar to spermine synthase isoform 4 [Canis familiaris]
sw13037	BGIBMGA011731	1.92055	1.48025	1.29535	2.38995	No hits found	
sw13591	BGIBMGA005469	0.90375	1.1894	0.95545	2.38955	3.00E-42	gi|83754534|pdb|2C35|G Chain G, Subunits Rpb4 And Rpb7 Of Human Rna Polymerase Ii Chain E, Subunits Rpb4 And Rpb7 Of Human Rna Polymerase Ii Chain C, Subunits Rpb4 And Rpb7 Of Human Rna Polymerase Ii Chain A
sw18769	BGIBMGA012827	0.8926	1.0213	0.9166	2.3879	Bmb013219	2.00E-27
sw01127	BGIBMGA010276	1.25865	1.26425	1.34445	2.3877	Bmb016838	1.00E-51
sw10000	BGIBMGA007518	0.80125	0.91265	0.9714	2.3867	5.00E-82	gi|89269046|emb|CAJ81570.1| TAR DNA binding protein [Xenopus tropicalis]
sw20846	BGIBMGA012268	0.8286	1.0328	0.8875	2.3856	8.00E-26	gi|66505114|ref|XP_624237.1| PREDICTED: similar to GA14985-PA [Apis mellifera]
sw09749	BGIBMGA002917	1.0399	1.0831	0.89385	2.3855	8.00E-75	gi|51859336|gb|AAH81422.1| Lin7c protein [Danio rerio] lin7c protein [Danio rerio] neuroepithelial polarity protein [Danio rerio]
sw15446	BGIBMGA014522	1.06435	1.40155	1.1249	2.3851	No hits found	
sw08935	BGIBMGA007083	0.9264	1.08305	0.89715	2.38465	2.00E-46	gi|66539089|ref|XP_624856.1| PREDICTED: similar to Potassium channel tetramerisation domain containing 5, partial [Apis mellifera]
sw01324	BGIBMGA004489	1.1834	1.02695	0.92925	2.38455	2.00E-27	gi|68697259|emb|CAJ14152.1| putative dodecenoylCoA deltaisomerase [Anopheles gambiae]
sw02948	BGIBMGA004162	0.87045	1.0012	0.827	2.3828	1.00E-44	gi|55641779|ref|XP_510278.1| PREDICTED: similar to chromosome 15 open reading frame 24; chromosome 15 hypothetical ATG/GTP binding protein [Pan troglodytes]
sw11158	BGIBMGA011920	0.8187	1.19005	1.58025	2.38175	2.00E-75	gi|847869|gb|AAA67954.1| zinc finger protein
sw18473	BGIBMGA006784	0.97535	0.4775	0.9897	2.3813	5.00E-06	gi|37704389|ref|NP_780751.2| rotatin [Mus musculus]
sw00139	BGIBMGA010142	1.33735	0.85485	1.02335	2.3804	6.00E-31	gi|1706194|sp|P80683|CUA3A_TENMO Larval cuticle protein A3A (TM-A3A) (TM-LCP A3A)
sw11173	BGIBMGA010908	0.90935	1.1003	1.00565	2.37975	8.00E-85	gi|57090201|ref|XP_537470.1| PREDICTED: similar to tRNA-(N1G37) methyltransferase [Canis familiaris]
sw20935	BGIBMGA006615	1.14065	1.1393	0.9764	2.3792	1.00E-11	gi|55234466|gb|EAA00473.3| ENSANGP00000014085 [Anopheles gambiae str. PEST] ENSANGP00000014085 [Anopheles gambiae str. PEST]
sw12388	BGIBMGA001556	0.74865	1.04245	0.73865	2.3791	2.00E-86	gi|87248203|gb|ABD36154.1| phosphomevalonate kinase [Bombyx mori]

sw13433	BGIBMGA001257	0.77045	0.8959	0.97965	2.37765	e-122	gi|66529639|ref|XP_624926.1| PREDICTED: similar to Hypothetical protein MGC73254 [Apis mellifera]
sw18570	BGIBMGA010396	0.9835	0.9022	0.9817	2.3763	7.00E-35	gi|68360882|ref|XP_709703.1| PREDICTED: similar to splicing factor, arginine/serine-rich 7 isoform 7 [Danio rerio]
sw15779	BGIBMGA005949	0.7595	1.07345	1.02775	2.37555	8.00E-31	gi|87248079|gb|ABD36092.1| coiled-coil domain containing 25 protein [Bombyx mori]
sw14966	BGIBMGA003669	0.8499	1.1517	1.0892	2.37475	1.00E-33	gi|87248493|gb|ABD36299.1| eukaryotic translation initiation factor 3 subunit 6 [Bombyx mori]
sw06193	BGIBMGA006980	1.00535	1.1179	1.148	2.3734	No hits found	
sw11250	BGIBMGA003398	0.6223	0.80095	0.72625	2.37225	Bmb004624	3.00E-27
sw03707	BGIBMGA010584	2.0209	1.4018	1.62485	2.37195	e-141	gi|87248295|gb|ABD36200.1| chymotrypsinogen [Bombyx mori]
sw04986	BGIBMGA004441	1.2459	0.964	1.1721	2.36615	3.00E-25	gi|54311237|gb|AAH84811.1| LOC495348 protein [Xenopus laevis]
sw04521	BGIBMGA011508	0.74295	0.94105	1.0988	2.36555	e-118	gi|74001082|ref|XP_856031.1| PREDICTED: similar to T-complex protein 1, theta subunit (TCP-1-theta) (CCT-theta) isoform 3 [Canis familiaris]
sw15884	BGIBMGA006588	1.46795	1.48185	1.4751	2.36505	1.00E-45	gi|73995433|ref|XP_543480.2| PREDICTED: similar to pescadillo homolog 1, containing BRCT domain [Canis familiaris]
sw22214	BGIBMGA012171	0.93855	1.0765	1.1641	2.36475	1.00E-15	gi|6446579|gb|AAA21249.2| beta-heavy-spectrin [Drosophila melanogaster]
sw12660	BGIBMGA000813	1.24105	0.9184	1.39915	2.36435	4.00E-22	gi|54636559|gb|EAL25962.1| GA15878-PA [Drosophila pseudoobscura]
sw03204	BGIBMGA000937	0.70975	0.89055	0.8685	2.3642	2.00E-55	gi|73977144|ref|XP_861870.1| PREDICTED: similar to Uroporphyrinogen decarboxylase (URO-D) (UPD) isoform 4 [Canis familiaris]
sw01725	BGIBMGA006812	1.106	1.0812	1.1884	2.36345	1.00E-27	gi|25246706|gb|AAN72834.1| allantoinase [Ctenocephalides felis]
sw15536	BGIBMGA013340	0.8645	1.1495	1.0016	2.3627	3.00E-25	gi|6164599|gb|AAF04459.1| dihydrofolate reductase [Heliothis virescens] Dihydrofolate reductase
sw11692	BGIBMGA006875	0.9063	1.05945	1.0775	2.3613	3.00E-18	gi|66513264|ref|XP_392851.2| PREDICTED: similar to GA17793-PA [Apis mellifera]
sw06498	BGIBMGA001907	1.42715	0.99955	1.14375	2.36085	1.00E-53	gi|57966866|ref|XP_562320.1| ENSANGP00000027535 [Anopheles gambiae str. PEST] ENSANGP00000027535 [Anopheles gambiae str. PEST]
sw18757	BGIBMGA003438	1	1.0499	1.422	2.35945	2.00E-24	gi|37537238|gb|AAH23738.2| Kelch domain containing 4 [Mus musculus] Kelch domain-containing protein 4
sw00146	BGIBMGA001664	1	1.08615	1.02705	2.35825	5.00E-59	gi|73976676|ref|XP_539554.2| PREDICTED: similar to Solute carrier family 2, facilitated glucose transporter, member 1 (Glucose transporter type 1, erythrocyte/brain) [Canis familiaris]
sw06175	BGIBMGA002730	1.22005	1.227	1.23535	2.3577	1.00E-38	gi|60677859|gb|AAX33436.1| RE32747p [Drosophila melanogaster]
sw08978	BGIBMGA007363	1.1663	1.2517	1.3585	2.35665	1.00E-57	gi|15213838|gb|AAK92194.1| ribosomal protein S26 [Spodoptera frugiperda]
sw21874	BGIBMGA005655	1.1068	1.02915	1.00085	2.35555	6.00E-20	gi|23128321|ref|ZP_00110172.1| COG4886: Leucine-rich repeat (LRR) protein [Nostoc punctiforme PCC 73102]
sw15929	BGIBMGA013069	1.10575	0.99185	1.06605	2.3519	2.00E-14	gi|24645182|ref|NP_649837.1| CG8359-PA [Drosophila melanogaster] LD09733p [Drosophila melanogaster] CG8359-PA [Drosophila melanogaster]
sw14703	BGIBMGA011314	0.8734	1.27365	1.17655	2.34955	2.00E-43	gi|18859921|ref|NP_573044.1| CG7872-PA [Drosophila melanogaster] LD24870p [Drosophila melanogaster] CG7872-PA [Drosophila melanogaster]
sw06821	BGIBMGA013924	1.2808	1.57665	1.78045	2.3495	5.00E-17	gi|24640056|ref|NP_727067.1| CG32750-PA [Drosophila melanogaster] CG32750-PA [Drosophila melanogaster] Vanin-like protein 3 precursor
sw10357	BGIBMGA011682	1.12185	1.20385	0.98775	2.3493	2.00E-40	gi|74004004|ref|XP_535873.2| PREDICTED: similar to peroxisomal D3,D2-enoyl-CoA isomerase isoform 1 [Canis familiaris]
sw17127	BGIBMGA002361	0.7056	0.66355	0.7648	2.34735	2.00E-76	gi|87248251|gb|ABD36178.1| WD repeat domain 61 [Bombyx mori]
sw01470	BGIBMGA002232	1	1.14165	1.2442	2.34595	No hits found	

sw03560	BGIBMGA002721	0.83235	0.98635	1.00825	2.3436	Bmb014019	5.00E-26
sw18664	BGIBMGA001966	1.06205	0.9283	1.1479	2.3422	5.00E-98	gi|132545|sp|P01122|RHO_APLCA RAS-like GTP-binding protein RHO rho protein
sw03200	BGIBMGA007267	0.75395	0.94095	0.74545	2.34035	6.00E-24	gi|50755667|ref|XP_414844.1| PREDICTED: similar to NADH-ubiquinone oxidoreductase PDSW subunit (Complex I-PDSW) (CI-PDSW) [Gallus gallus]
sw04152	BGIBMGA007873	1.1519	1.0026	0.713	2.34	No hits found	
sw10585	BGIBMGA006143	1	1.01855	1	2.33505	No hits found	
sw10650	BGIBMGA013784	0.76385	1.30625	0.8591	2.33295	2.00E-48	gi|10863157|gb|AAG23916.1| imprinted and ancient [Mus musculus]
sw15825	BGIBMGA009012	0.79415	0.81475	1.02315	2.32895	Bmb035709	5.00E-55
sw03736	BGIBMGA001509	1.2058	1.08815	1.1597	2.32825	e-106	gi|72011152|ref|XP_780300.1| PREDICTED: similar to Guanine nucleotide releasing protein (GNRP) (P140 Ras-GRF) [Strongylocentrotus purpuratus]
sw18390	BGIBMGA003500	1.26	1.484	1.4213	2.3269	2.00E-79	gi|9527|emb|CAA47358.1| luciferase [Luciola lateralis] Luciferin 4-monooxygenase (Luciferase)
sw05196	BGIBMGA009700	1.2477	1.08665	0.976	2.3236	6.00E-07	gi|73952314|ref|XP_852456.1| PREDICTED: similar to Cytochrome c oxidase polypeptide VIIc, mitochondrial precursor [Canis familiaris]
sw05842	BGIBMGA005678	2.288	1.03225	1.12805	2.32195	No hits found	
sw01621	BGIBMGA013585	0.84575	0.9463	0.9632	2.32165	5.00E-47	gi|85857460|gb|ABC86266.1| RE45749p [Drosophila melanogaster]
sw21951	BGIBMGA001202	1.739	2.17935	2.6849	2.3214	e-125	gi|87248175|gb|ABD36140.1| muscle LIM protein [Bombyx mori]
sw00581	BGIBMGA011651	1.05865	0.99945	0.96025	2.3198	5.00E-36	gi|50603837|gb|AAH78404.1| Cytokine induced apoptosis inhibitor 1 [Danio rerio] cytokine induced apoptosis inhibitor 1 [Danio rerio]
sw00797	BGIBMGA000202	0.871	0.73855	0.58105	2.31945	No hits found	
sw11070	BGIBMGA012808	1.0123	1.28205	1.20455	2.31905	e-147	gi|55236224|gb|EAA14338.2| ENSANGP00000006039 [Anopheles gambiae str. PEST] ENSANGP00000006039 [Anopheles gambiae str. PEST]
sw09461	BGIBMGA004078	1.08165	0.912	1.11435	2.31865	4.00E-82	gi|72007484|ref|XP_781161.1| PREDICTED: similar to mitochondrial ribosomal protein S9 [Strongylocentrotus purpuratus]
sw09066	BGIBMGA009169	1.00945	0.64275	1.05615	2.3173	No hits found	
sw08193	BGIBMGA008284	0.80255	0.90275	1.0213	2.3167	2.00E-30	gi|68421636|ref|XP_683350.1| PREDICTED: similar to ELAV-like 2 isoform 1 isoform 1 [Danio rerio]
sw01521	BGIBMGA011634	1	1	1	2.315	3.00E-18	gi|83763951|emb|CAJ12162.1| cg12111 protein [Drosophila simulans]
sw21778	BGIBMGA011804	1.1491	1.39195	1.42375	2.31255	No hits found	
sw13440	BGIBMGA002304	1.1867	1.0756	1.23555	2.31215	Bmb018393	7.00E-23
sw13175	BGIBMGA004515	0.9027	1.67455	1.44745	2.3107	e-104	gi|56378323|dbj|BAD74198.1| heat shock protein hsp23.7 [Bombyx mori]
sw14953	BGIBMGA010827	1.04885	1.13835	1.26335	2.30955	8.00E-12	gi|57107227|ref|XP_534981.1| PREDICTED: similar to exosome component 1 isoform 1 [Canis familiaris]
sw04110	BGIBMGA004573	0.66985	0.73375	0.8717	2.3079	3.00E-21	gi|62083383|gb|AAX62416.1| small nuclear ribonucleoprotein G [Lysiphlebus testaceipes]
sw15235	BGIBMGA011810	1.08815	1.0286	1.293	2.30455	7.00E-27	gi|4972738|gb|AAD34764.1| unknown [Drosophila melanogaster]
sw18276	BGIBMGA000236	0.75375	0.83945	0.65015	2.3043	3.00E-15	gi|26788031|emb|CAD58772.1| novel protein similar to human isovaleryl Coenzyme A dehydrogenase (IVD) [Danio rerio] Isovaleryl Coenzyme A dehydrogenase [Danio rerio]
sw09911	BGIBMGA002406	0.95795	1.27435	1.10525	2.3022	Bmb038389	4.00E-92

sw05068	BGIBMGA007535	0.84575	1.0148	1.00445	2.2999	8.00E-05	gi|54641026|gb|EAL29777.1| GA12543-PA [Drosophila pseudoobscura]
sw05584	BGIBMGA009067	1.1001	1.34675	1.27775	2.2997	9.00E-60	gi|58395618|ref|XP_321387.2| ENSANGP00000011567 [Anopheles gambiae str. PEST] ENSANGP00000011567 [Anopheles gambiae str. PEST]
sw06006	BGIBMGA000713	0.1349	0.586	0.6147	2.29745	Bmb018933	No hits found
sw22871	BGIBMGA002309	1.08325	1.1373	1.47225	2.29595	4.00E-96	gi|87248551|gb|ABD36328.1| nucleoplasmin isoform 2 [Bombyx mori]
sw10352	BGIBMGA005930	0.94875	1.14605	1.1182	2.29525	0	gi|87248441|gb|ABD36273.1| WD40 protein [Bombyx mori]
sw12837	BGIBMGA002083	0.9991	0.8748	0.67545	2.29445	8.00E-62	gi|1526415|dbj|BAA09449.1| actin [Chlamydomonas reinhardtii] Actin actin [Chlamydomonas reinhardtii]
sw00334	BGIBMGA008278	0.88665	0.8061	0.8839	2.2906	6.00E-08	gi|56199440|gb|AAV84209.1| chymotrypsin [Culicoides sonorensis]
sw15960	BGIBMGA006158	0.9567	0.90485	1.1805	2.28965	e-113	gi|87248645|gb|ABD36375.1| prohibitin protein WPH [Bombyx mori]
sw06569	BGIBMGA012979	0.99295	0.8611	0.843	2.2883	3.00E-14	gi|55242208|gb|EAL40896.1| ENSANGP00000027230 [Anopheles gambiae str. PEST] ENSANGP00000012173 [Anopheles gambiae str. PEST] ENSANGP00000027230
sw07764	BGIBMGA010952	0.91515	1.0075	0.80375	2.28465	No hits found	
sw05181	BGIBMGA007733	1.3369	1.551	1.3551	2.28395	8.00E-15	gi|66517218|ref|XP_396168.2| PREDICTED: similar to RE58116p [Apis mellifera]
sw20723	BGIBMGA010101	1.20065	1.6396	1.3243	2.2837	No hits found	
sw12097	BGIBMGA002944	1.12845	1.35575	0.48835	2.2824	6.00E-28	gi|66518230|ref|XP_623818.1| PREDICTED: similar to putative alcohol dehydrogenase [Apis mellifera]
sw22256	BGIBMGA011840	1.2008	1.1654	1	2.28175	2.00E-06	gi|55649967|ref|XP_524400.1| PREDICTED: similar to zinc finger protein 524 [Pan troglodytes]
sw05506	BGIBMGA001335	1.555	1.35185	1.31225	2.27985	Bmb009933	e-105
sw03914	BGIBMGA010735	0.93445	1.05135	1.56335	2.27895	Bmb022011	3.00E-33
sw10405	BGIBMGA013918	0.82425	0.9437	0.73695	2.278	2.00E-54	gi|1834425|emb|CAA71871.1| Trf-proximal protein [Drosophila melanogaster]
sw15827	BGIBMGA006344	0.80915	0.85005	1.1544	2.27675	Bmb035726	6.00E-06
sw14986	BGIBMGA007019	1.00825	0.94285	0.95315	2.2761	2.00E-12	gi|50758414|ref|XP_415911.1| PREDICTED: similar to Mitochondrial ribosomal protein S23 [Gallus gallus]
sw03337	BGIBMGA004596	0.89835	1.031	1.12695	2.27585	9.00E-24	gi|18693297|gb|AAL78310.1| PDZ domain containing protein NHERF-2 [Oryctolagus cuniculus] Na(+)/H(+) exchange regulatory cofactor NHE-RF2 (PDZ domain containing protein NHERF-2)
sw04512	BGIBMGA001898	0.94095	0.9373	0.8535	2.2757	6.00E-75	gi|1060912|dbj|BAA07406.1| RPB5 [Homo sapiens]
sw07203	BGIBMGA003702	0.9556	1.27335	1.30025	2.27505	Bmb046601	3.00E-14
sw07321	BGIBMGA013551	0.90295	1.02045	0.9083	2.27495	3.00E-37	gi|66552919|ref|XP_625097.1| PREDICTED: similar to CG6884-PA [Apis mellifera]
sw13579	BGIBMGA013514	1.3195	1.19995	1.26575	2.2724	2.00E-06	gi|37681861|gb|AAQ97808.1| hypothetical protein BC013949 [Danio rerio] Zgc:77492 protein [Danio rerio] hypothetical protein LOC393537 [Danio rerio] Hypothetical protein MGC65939 [Danio rerio]
sw09044	BGIBMGA005628	0.9761	1.3278	1.0366	2.27155	e-157	gi|87248245|gb|ABD36175.1| uridine 5'-monophosphate synthase [Bombyx mori]
sw04365	BGIBMGA008111	1.111	1.23385	0.7611	2.2691	6.00E-46	gi|7657611|ref|NP_055095.1| DnaJ (Hsp40) homolog, subfamily C, member 8 [Homo sapiens] SPF31 [Homo sapiens]
sw04717	BGIBMGA013458	1.0529	1.0996	0.9505	2.26815	Bmb043844	3.00E-11
sw20508	BGIBMGA002971	0.91105	1.099	1.0143	2.26785	9.00E-64	gi|87313157|gb|ABD37875.1| catsup protein [Drosophila melanogaster]

sw08924	BGIBMGA000603	0.63495	1	0.92285	2.2661	2.00E-18	gi|73966446|ref|XP_852670.1| PREDICTED: similar to sperm associated antigen 9 isoform 1 isoform 2 [Canis familiaris]
sw12797	BGIBMGA006727	1.0466	1.4222	1.19375	2.2653	e-173	gi|66509122|ref|XP_394574.2| PREDICTED: similar to CG11594-PA, isoform A [Apis mellifera]
sw18095	BGIBMGA010291	1.4322	1.48075	1.6351	2.2649	Bmb037490	7.00E-46
sw10478	BGIBMGA001554	1.50905	2.07925	1.9729	2.26425	3.00E-17	gi|19698929|gb|AAL91200.1| unknown protein [Arabidopsis thaliana] unknown protein [Arabidopsis thaliana]
sw10996	BGIBMGA011897	0.96065	1.0196	1.14885	2.26395	No hits found	
sw08134	BGIBMGA003351	1.13415	0.98995	1.3533	2.2636	1.00E-48	gi|54290089|dbj|BAD61056.1| MCM7 [Bombyx mori]
sw07899	BGIBMGA006869	0.9021	1.1187	0.3674	2.2625	2.00E-41	gi|55243516|gb|EAA06508.2| ENSANGP00000014874 [Anopheles gambiae str. PEST] ENSANGP00000014874 [Anopheles gambiae str. PEST]
sw21457	BGIBMGA007844	1.0404	1.2234	1.10845	2.2618	2.00E-08	gi|27374352|gb|AAO01093.1| Surf6-PA [Drosophila willistoni]
sw08984	BGIBMGA010658	0.9033	1.0095	0.9799	2.26085	Bmb022624	9.00E-07
sw16289	BGIBMGA012810	0.8472	1.0839	1.1496	2.26025	5.00E-53	gi|14211845|ref|NP_115938.1| IMP2 inner mitochondrial membrane protease-like [Homo sapiens] inner mitochondrial membrane peptidase 2
sw06218	BGIBMGA011887	0.90465	0.9005	0.9633	2.26015	Bmb023115	1.00E-94
sw11664	BGIBMGA001223	0.94985	1.1198	1.06915	2.25735	0	gi|6014919|sp|O61305|DBP80_DROME DEAD-box helicase Dbp80 DEAD-box helicase [Drosophila melanogaster]
sw06330	BGIBMGA008593	1.07975	1.20945	1.235	2.2529	No hits found	
sw22089	BGIBMGA011721	1.54605	0.8652	1.1719	2.25195	2.00E-18	gi|56308438|ref|NP_649115.2| CG18294-PA [Drosophila melanogaster] RE51966p [Drosophila melanogaster] CG18294-PA [Drosophila melanogaster]
sw15316	BGIBMGA004827	0.9464	1.0722	0.99635	2.25175	6.00E-06	gi|27356613|gb|AAO06952.1| MSTP052 [Homo sapiens]
sw02241	BGIBMGA004738	1	1	0.72155	2.2514	2.00E-30	gi|60677713|gb|AAX33363.1| RH65810p [Drosophila melanogaster]
sw05296	BGIBMGA008248	1.0007	0.83925	1.13795	2.251	3.00E-46	gi|6594153|emb|CAB63528.1| EG:BACH59J11.1 [Drosophila melanogaster]
sw14362	BGIBMGA000722	0.829	0.89965	0.87285	2.24855	Bmb024668	5.00E-18
sw11111	BGIBMGA008714	1.05795	0.97815	1.08745	2.24695	5.00E-64	gi|72137308|ref|XP_793386.1| PREDICTED: similar to mitochondrial ribosomal protein L45 [Strongylocentrotus purpuratus]
sw16738	BGIBMGA005505	0.8071	1.3127	0.94295	2.2469	Bmb046240	4.00E-11
sw14091	BGIBMGA002654	1.44375	1.17685	1.50235	2.24375	2.00E-78	gi|499204|gb|AAC14192.1| D-E-A-D box protein [Drosophila melanogaster]
sw16445	BGIBMGA000383	0.72365	1.07595	0.9863	2.2425	5.00E-19	gi|73960510|ref|XP_537169.2| PREDICTED: similar to odorant response abnormal 4 isoform 1 [Canis familiaris]
sw05319	BGIBMGA005692	1.0139	1.1851	1.53875	2.2423	3.00E-54	gi|66499215|ref|XP_624770.1| PREDICTED: similar to ENSANGP00000015190 [Apis mellifera]
sw00284	BGIBMGA004975	1.4421	1.11715	1.03405	2.23945	8.00E-05	gi|67846976|gb|AAY82180.1| CG6547 [Drosophila melanogaster]
sw01671	BGIBMGA007377	0.8036	0.5139	0.5572	2.239	5.00E-72	gi|61191881|gb|AAX39408.1| serine protease [Bombyx mandarina]
sw18899	BGIBMGA014171	0.8411	0.3805	0.77045	2.23705	4.00E-29	gi|102972|pir||A28068 microvitellogenin precursor - tobacco hornworm MICROVITELLOGENIN PRECURSOR microvitellogenin microvitellogenin
sw13873	BGIBMGA007726	1.08005	0.9678	0.9294	2.23555	e-150	gi|28277919|gb|AAH45990.1| Bcs1l protein [Danio rerio]
sw03556	BGIBMGA009259	1.39155	1.23065	0.79195	2.23475	Bmb013903	5.00E-05

sw16561	BGIBMGA012567	0.888	1.03705	0.9021	2.2345	Bmb043350	4.00E-34
sw00924	BGIBMGA003924	0.83575	0.823	0.8703	2.2332	e-116	gi|87248369|gb|ABD36237.1| NADH dehydrogenase (ubiquinone) Fe-S protein 8 [Bombyx mori]
sw07571	BGIBMGA004776	1	1	1	2.2287	e-139	gi|76616634|ref|XP_872845.1| PREDICTED: similar to heterogeneous nuclear ribonucleoprotein methyltransferase-like 4 [Bos taurus]
sw04011	BGIBMGA011062	0.7367	1.0387	0.752	2.22815	2.00E-37	gi|7657313|ref|NP_055277.1| Lsm1 protein [Homo sapiens] Lsm1 protein [Homo sapiens] Lsm1 protein [Homo sapiens] CaSm [Homo sapiens] U6 snRNA-associated Sm-like protein LSm1 (Small nuclear ribonuclear CaSm)
sw03046	BGIBMGA011994	0.7632	1.00395	0.6681	2.22375	3.00E-21	gi|25012393|gb|AAN71305.1| RE11282p [Drosophila melanogaster]
sw16221	BGIBMGA001916	0.7264	0.9271	0.83345	2.2219	Bmb039234	4.00E-34
sw06147	BGIBMGA009052	1.2195	1.03885	1.04735	2.22025	1.00E-84	gi|72163576|ref|XP_793131.1| PREDICTED: similar to Glyoxylate reductase/hydroxypyruvate reductase [Strongylocentrotus purpuratus]
sw10495	BGIBMGA002752	1.06775	1	0.9236	2.21945	1.00E-17	gi|56269188|gb|AAH87435.1| LOC496040 protein [Xenopus laevis]
sw13572	BGIBMGA012671	0.9722	1.11595	0.885	2.21885	4.00E-08	gi|27682127|ref|XP_215228.1| PREDICTED: similar to Ubiquitin-like protein 4 (Ubiquitin-like protein GDX) [Rattus norvegicus]
sw04473	BGIBMGA013018	1.1785	1.22645	1.2148	2.218	Bmb035495	1.00E-12
sw15920	BGIBMGA001889	0.9262	0.8325	0.9043	2.2176	No hits found	
sw04166	BGIBMGA008400	1.1199	1.1543	1.3285	2.2169	No hits found	
sw21888	BGIBMGA013792	1.39595	1.0894	1.1388	2.21445	3.00E-69	gi|70909519|emb|CAJ17183.1| ribosomal protein S11e [Eucinetus sp. APV-2005]
sw21243	BGIBMGA009696	0.9637	1.10795	0.92385	2.2131	Bmb032323	5.00E-24
sw12116	BGIBMGA012032	0.83225	1.0832	1.1484	2.2123	2.00E-25	gi|50416634|gb|AAH77653.1| Heat shock 10kDa protein 1 (chaperonin 10) [Xenopus tropicalis] heat shock 10kDa protein 1 (chaperonin 10) [Xenopus tropicalis] heat shock 10kDa protein 1 (chaperonin 10) [Xenopus tropicalis]
sw03938	BGIBMGA001708	0.6963	0.5718	0.755	2.2116	1.00E-33	gi|66525368|ref|XP_392073.2| PREDICTED: similar to Colorectal mutant cancer protein (MCC protein) [Apis mellifera]
sw01900	BGIBMGA004612	1.94495	1.5786	1.92665	2.21125	0	gi|46358051|dbj|BAD15163.1| heat shock protein [Antheraea yamamai]
sw08152	BGIBMGA006935	1.22465	1.26095	1.25155	2.21125	1.00E-32	gi|24640068|ref|NP_572300.1| CG14446-PA [Drosophila melanogaster] CG14446-PA [Drosophila melanogaster]
sw12581	BGIBMGA003415	0.905	0.89835	0.9727	2.2112	3.00E-45	gi|60594462|pdb|2BIU|X Chain X, Crystal Structure Of Human Cyclophilin D At 1.7 A Resolution, Dmso Complex Chain X, Crystal Structure Of Human Cyclophilin D At 1.7 A Resolution
sw14722	BGIBMGA010720	0.98345	1.04865	1.2907	2.211	1.00E-34	gi|83022250|ref|XP_918113.1| PREDICTED: similar to zinc finger protein 420 [Mus musculus]
sw20012	BGIBMGA012172	0.77885	0.82385	1.0531	2.211	2.00E-08	gi|73982258|ref|XP_533172.2| PREDICTED: similar to thioredoxin-related transmembrane protein 2 isoform 1 [Canis familiaris]
sw04868	BGIBMGA005374	1.1328	1.09765	0.98005	2.2062	2.00E-43	gi|72007676|ref|XP_785301.1| PREDICTED: similar to polynucleotide kinase 3-phosphatase [Strongylocentrotus purpuratus]
sw07971	BGIBMGA013182	0.94595	1.2033	0.7714	2.206	No hits found	
sw01642	BGIBMGA011514	0.76265	1.17205	0.8983	2.20455	4.00E-10	gi|50749218|ref|XP_421538.1| PREDICTED: similar to nuclear receptor binding factor 2; nuclear receptor binding factor-2; comodulator of PPAR and RXR [Gallus gallus]
sw04041	BGIBMGA002430	1.19375	0.97915	1.25405	2.2018	Bmb025306	e-118
sw04644	BGIBMGA003119	1.23915	1.07805	1.16075	2.199	9.00E-55	gi|68362350|ref|XP_696178.1| PREDICTED: similar to Elongation factor Ts, mitochondrial precursor (EF-Ts) (EF-TsMt) (2A3-2) [Danio rerio]
sw22252	BGIBMGA010793	0.87065	0.78745	1	2.1977	Bmb024272	1.00E-24
sw08472	BGIBMGA010234	0.7818	0.9708	0.8741	2.19625	2.00E-48	gi|87248209|gb|ABD36157.1| DNA-directed RNA polymerase subunit 6-like protein [Bombyx mori]

sw09637	BGIBMGA005145	1.102	1.04335	0.92655	2.1949	6.00E-24	gi|55297200|dbj|BAD68874.1| glycine cleavage T protein-like [Oryza sativa (japonica cultivar-group)] glycine cleavage T protein-like [Oryza sativa (japonica cultivar-group)]
sw22882	BGIBMGA005064	1.22665	1.05575	0.9807	2.19385	e-124	gi|85740627|gb|ABC79691.1| glutathione S-transferase 4 [Bombyx mori]
sw18808	BGIBMGA012505	0.78265	0.85265	0.8679	2.1932	9.00E-17	gi|71031052|ref|XP_765168.1| protein disulfide isomerase [Theileria parva strain Muguga] protein disulfide isomerase [Theileria parva]
sw05420	BGIBMGA000360	0.9091	1.19575	0.81675	2.1905	5.00E-17	gi|57918265|ref|XP_556948.1| ENSANGP00000026833 [Anopheles gambiae str. PEST] ENSANGP00000026833 [Anopheles gambiae str. PEST]
sw18382	BGIBMGA003336	0.7564	1.0092	0.95625	2.19025	5.00E-91	gi|76651937|ref|XP_882268.1| PREDICTED: similar to RNA (guanine-7-) methyltransferase isoform 4 [Bos taurus]
sw16288	BGIBMGA011566	0.7301	1.01965	0.937	2.1878	5.00E-14	gi|50749705|ref|XP_421721.1| PREDICTED: similar to DPCD protein [Gallus gallus]
sw17084	BGIBMGA010398	0.9278	1.0241	1	2.1868	8.00E-05	gi|66510656|ref|XP_393387.2| PREDICTED: similar to ENSANGP00000008952 [Apis mellifera]
sw20128	BGIBMGA005411	0.8433	0.9568	1.0302	2.1863	Bmb033703	e-123
sw09541	BGIBMGA006802	1.2295	1.2016	1.11895	2.18415	9.00E-24	gi|57033124|gb|AAH88913.1| LOC496323 protein [Xenopus laevis]
sw18672	BGIBMGA013624	1.09625	1.06015	1.25025	2.183	3.00E-72	gi|57096845|ref|XP_532690.1| PREDICTED: similar to tigger transposable element derived 4 [Canis familiaris]
sw22325	BGIBMGA002288	1.0308	1.0051	0.4899	2.1828	6.00E-67	gi|9651929|gb|AAF91316.1| immulectin-2 [Manduca sexta]
sw08246	BGIBMGA000520	1	1	1	2.18275	6.00E-72	gi|68989037|dbj|BAE06188.1| glycine rich protein [Bombyx mori]
sw07403	BGIBMGA004741	1.143	1.2685	0.89985	2.18065	6.00E-65	gi|74009016|ref|XP_538183.2| PREDICTED: similar to HIV TAT specific factor 1 [Canis familiaris]
sw01366	BGIBMGA005369	1.17705	1.0279	1.11475	2.18045	3.00E-19	gi|1244518|gb|AAA93257.1| allatostatin neuropeptide precursor [Pseudaletia unipuncta]
sw06276	BGIBMGA003661	1.26135	0.7638	0.89995	2.1804	No hits found	
sw09973	BGIBMGA003909	0.81455	1.07925	0.8694	2.17955	5.00E-30	gi|38492611|pdb|1OE0|D Chain D, Crystal Structure Of Drosophila Deoxyribonucleoside Kinase In Complex With Dttp Chain C, Crystal Structure Of Drosophila Deoxyribonucleoside Kinase In Complex With Dttp Chain B
sw15259	BGIBMGA001921	0.8379	0.93205	1.05495	2.17905	2.00E-85	gi|558568|emb|CAA50674.1| actin related protein [Drosophila melanogaster] actin-related protein
sw03335	BGIBMGA009111	1.0233	1.05355	0.97665	2.17545	2.00E-24	gi|20177053|gb|AAM12288.1| RE13587p [Drosophila melanogaster]
sw07936	BGIBMGA006800	1.2314	1.33215	0.9734	2.1741	7.00E-38	gi|58476259|gb|AAH89602.1| 1700023B02Rik protein [Mus musculus]
sw15396	BGIBMGA009714	3.9267	1.615	1.2362	2.17255	Bmb032353	No hits found
sw11108	BGIBMGA002242	0.0501	0.6385	0.6398	2.17145	No hits found	
sw17801	BGIBMGA001829	1.0979	1.24205	1.1656	2.1714	3.00E-35	gi|82884290|ref|XP_903599.1| PREDICTED: XPMC2 prevents mitotic catastrophe 2 homolog isoform 5 [Mus musculus]
sw13760	BGIBMGA001159	0.94765	0.99935	0.82945	2.17015	7.00E-94	gi|50418088|gb|AAH77615.1| MGC84611 protein [Xenopus laevis] Vacuolar protein sorting protein 36
sw11720	BGIBMGA007100	0.9727	0.867	0.80195	2.16975	2.00E-89	gi|14041150|emb|CAC38761.1| leukotriene B4 [Geodia cydonium]
sw12317	BGIBMGA009476	1.1448	1.21215	0.84735	2.16955	5.00E-44	gi|74831719|emb|CAJ30028.1| carboxypeptidase B precursor [Helicoverpa zea]
sw18489	BGIBMGA010278	1.46225	1.54515	1.5254	2.16855	1.00E-16	gi|18129616|ref|NP_082504.1| PIN2/TRF1-interacting protein [Mus musculus] Pin2-interacting protein X1 [Mus musculus] LPTS1 [Mus musculus] Pin2-interacting protein X1 (TRF1-interacting protein 1)
sw11674	BGIBMGA001711	0.8581	0.9512	0.88055	2.1683	4.00E-63	gi|55244531|gb|EAA05402.2| ENSANGP00000019750 [Anopheles gambiae str. PEST] ENSANGP00000019750 [Anopheles gambiae str. PEST]
sw16454	BGIBMGA013305	1	1	1	2.1643	Bmb041849	6.00E-43

sw04142	BGIBMGA006085	1.37935	1.0062	1.707	2.1625	0	gi|6433838|emb|CAB60723.1| DNop5 protein [Drosophila melanogaster]
sw03846	BGIBMGA012204	1.1828	1.0006	0.97045	2.1601	1.00E-64	gi|71725396|ref|NP_001025161.1| D14Ertd209e protein [Mus musculus]
sw06845	BGIBMGA009073	0.7973	1.0846	0.78215	2.15985	8.00E-10	gi|1836157|gb|AAB46908.1| fungal protease inhibitor F; FPI-F [Bombyx mori] Fungal protease inhibitor F precursor (FPI-F) fungal protease-specific inhibitor-F [Bombyx mori]
sw17051	BGIBMGA012550	0.51685	0.79585	0.74475	2.15955	5.00E-96	gi|87248607|gb|ABD36356.1| signal peptidase 18 kDa subunit [Bombyx mori]
sw03909	BGIBMGA008211	1.17635	0.95555	1.1138	2.1587	1.00E-13	gi|73980977|ref|XP_855121.1| PREDICTED: similar to bolA-like 3 [Canis familiaris]
sw09544	BGIBMGA007716	1.2122	1.0999	0.9352	2.15855	e-180	gi|87248077|gb|ABD36091.1| arginase [Bombyx mori]
sw19539	BGIBMGA006159	0.758	0.96695	1.0401	2.15725	9.00E-63	gi|68394187|ref|XP_687166.1| PREDICTED: similar to inosine triphosphatase isoform a [Danio rerio]
sw01149	BGIBMGA006032	1.38115	0.97885	1.10795	2.15705	2.00E-18	gi|61889092|ref|NP_077325.2| adenylate kinase 1 [Rattus norvegicus] Adenylate kinase 1 [Rattus norvegicus]
sw13759	BGIBMGA002179	0.608	1.11675	0.8875	2.15675	No hits found	
sw02931	BGIBMGA000282	0.7687	0.88885	0.6396	2.1551	2.00E-16	gi|117622|sp|P11734|CU08_LOCMI Cuticle protein 8 (LM-8) (LM-ACP 8)
sw07325	BGIBMGA011686	1.04165	1.18405	0.70475	2.1528	5.00E-76	gi|66517442|ref|XP_623871.1| PREDICTED: similar to ENSANGP00000013944 [Apis mellifera]
sw12193	BGIBMGA013506	1.25545	1.28805	1.1581	2.1506	5.00E-55	gi|72098763|ref|XP_799251.1| PREDICTED: similar to Protein C20orf43 [Strongylocentrotus purpuratus]
sw16074	BGIBMGA008956	0.9734	1.23915	0.90935	2.14855	Bmb038011	e-104
sw16663	BGIBMGA004848	1.28045	1.2879	1.224	2.14725	5.00E-44	gi|55247392|gb|EAA01895.3| ENSANGP00000013804 [Anopheles gambiae str. PEST] ENSANGP00000015666 [Anopheles gambiae str. PEST] ENSANGP00000015666 [Anopheles gambiae str. PEST]
sw08168	BGIBMGA004994	1.02585	1.09985	1.1477	2.14525	e-143	gi|40645085|dbj|BAD06461.1| homologue of DNA-directed RNA polymerase II subunit [Antheraea pernyi] homologue of DNA-directed RNA polymerase II subunit [Antheraea pernyi]
sw02528	BGIBMGA003564	0.72985	0.6492	0.8163	2.1418	Bmb043020	No hits found
sw08622	BGIBMGA000668	0.87785	0.9543	0.70695	2.14	1.00E-18	gi|49900160|gb|AAH77030.1| MGC89869 protein [Xenopus tropicalis] MGC89869 protein [Xenopus tropicalis]
sw18227	BGIBMGA008049	0.9308	1.04315	1.4078	2.1396	2.00E-07	gi|66531974|ref|XP_393396.2| PREDICTED: similar to putative monoacylglycerol acyltransferase 1 [Apis mellifera]
sw03037	BGIBMGA010345	1.88725	2.05275	1.71725	2.1388	No hits found	
sw18400	BGIBMGA004727	0.22395	0.6916	0.4914	2.1383	Bmb027076	4.00E-05
sw09770	BGIBMGA006329	0.7855	1.00645	0.8829	2.13815	6.00E-21	gi|66910579|gb|AAH97374.1| ADP-ribosylation factor-like 6 interacting protein 4 [Rattus norvegicus] ADP-ribosylation factor-like 6 interacting protein 4 [Rattus norvegicus]
sw19962	BGIBMGA010938	1.44945	1.369	0.7186	2.13725	4.00E-68	gi|57966866|ref|XP_562320.1| ENSANGP00000027535 [Anopheles gambiae str. PEST] ENSANGP00000027535 [Anopheles gambiae str. PEST]
sw07883	BGIBMGA012714	0.71465	0.8088	0.8629	2.13685	5.00E-13	gi|28077083|ref|NP_291081.2| amnionless [Mus musculus] amnionless precursor protein [Mus musculus] amnionless precursor protein [Mus musculus] Amnionless [Mus musculus] Amnionless protein precursor
sw15413	BGIBMGA005336	1.4101	1.2596	1.1751	2.13555	3.00E-31	gi|58395895|ref|XP_321568.2| ENSANGP00000011533 [Anopheles gambiae str. PEST] ENSANGP00000011533 [Anopheles gambiae str. PEST]
sw07985	BGIBMGA000419	1.628	1.7097	1.2878	2.13515	1.00E-46	gi|72156944|ref|XP_794386.1| PREDICTED: similar to Lambda-crystallin homolog [Strongylocentrotus purpuratus]
sw14783	BGIBMGA002939	0.7981	1.1418	0.96835	2.135	5.00E-31	gi|56118552|ref|NP_001007992.1| hpgd-prov protein [Xenopus tropicalis] Hpgd-prov protein [Xenopus tropicalis]
sw06082	BGIBMGA005878	0.9099	1.0804	1.1478	2.13235	5.00E-60	gi|228300|prf||1802389A transcription factor IIE:SUBUNIT=beta
sw18220	BGIBMGA009106	1.4047	1.16185	0.98535	2.1309	4.00E-70	gi|3514020|gb|AAC34097.1| glutathione transferase; PiGSTII [Platynota idaeusalis]

sw22924	BGIBMGA001060	0.7506	0.8669	0.79815	2.13015	4.00E-37	gi|3065729|gb|AAC14276.1| clathrin light chain [Drosophila melanogaster]
sw09598	BGIBMGA010739	0.99455	1.1308	1.70585	2.12935	Bmb032483	e-113
sw08971	BGIBMGA002768	0.9068	1.11755	0.8439	2.12875	2.00E-10	gi|89268302|emb|CAJ83054.1| suppressor of Ty 7 (S. cerevisiae)-like;STAF65(gamma) [Xenopus tropicalis]
sw13208	BGIBMGA009776	1.90535	1.04695	1.0717	2.12715	2.00E-25	gi|6560689|gb|AAF16722.1| putative cuticle protein [Manduca sexta]
sw09714	BGIBMGA003010	1	1	1	2.12625	1.00E-69	gi|50734127|ref|XP_418979.1| PREDICTED: similar to Werner helicasae interacting protein 1; Werner syndrome homolog (human) interacting protein [Gallus gallus]
sw20862	BGIBMGA009285	0.91965	1.0347	1.1505	2.126	Bmb009445	1.00E-38
sw14169	BGIBMGA014214	0.4841	0.7689	0.8131	2.12525	No hits found	
sw05142	BGIBMGA000365	1.1546	1.1735	1.2839	2.1251	4.00E-40	gi|72086744|ref|XP_792890.1| PREDICTED: similar to RAB, member of RAS oncogene family-like 3 [Strongylocentrotus purpuratus]
sw03474	BGIBMGA005425	0.80235	0.92485	1.1197	2.1232	Bmb011946	1.00E-68
sw16063	BGIBMGA012718	0.8898	1.1182	1.2806	2.12285	2.00E-36	gi|76621180|ref|XP_613288.2| PREDICTED: similar to solute carrier family 35, member E1 [Bos taurus]
sw19533	BGIBMGA005431	1	1	0.87455	2.1215	2.00E-40	gi|62471655|ref|NP_001014495.1| pickpocket 13 CG33508-PA [Drosophila melanogaster] pickpocket 13 [Drosophila melanogaster] CG33508-PA [Drosophila melanogaster]
sw05309	BGIBMGA006778	0.8327	1.0596	0.71245	2.12115	No hits found	
sw15615	BGIBMGA012200	0.80445	1.01505	1.09215	2.1191	4.00E-06	gi|72141202|ref|XP_791628.1| PREDICTED: similar to mitochondrial ribosomal protein L43 isoform b [Strongylocentrotus purpuratus]
sw07662	BGIBMGA007301	1.23265	1.1855	0.9169	2.1181	1.00E-24	gi|23308325|gb|AAN18132.1| At5g47240/MQL5_10 [Arabidopsis thaliana] AT5g47240/MQL5_10 [Arabidopsis thaliana]
sw11627	BGIBMGA011108	1.43175	1.48205	1.2007	2.11715	3.00E-36	gi|78058375|gb|ABB17552.1| NADH-dependent retinal reductase [Oryctolagus cuniculus]
sw13068	BGIBMGA008020	0.95905	0.9941	1.134	2.11635	2.00E-28	gi|68404433|ref|XP_694901.1| PREDICTED: similar to cubilin, partial [Danio rerio]
sw06692	BGIBMGA007632	0.97965	1.07365	1	2.113	6.00E-26	gi|55664893|emb|CAH70763.1| novel protein (FLJ13150) [Homo sapiens] C1orf82 protein [Homo sapiens]
sw11800	BGIBMGA008749	1.3631	1.06635	1.1529	2.11235	3.00E-94	gi|87248451|gb|ABD36278.1| adenylate cyclase [Bombyx mori]
sw18756	BGIBMGA000254	1.00125	1	0.8761	2.11225	8.00E-37	gi|55245976|gb|EAA04147.2| ENSANGP00000013342 [Anopheles gambiae str. PEST] ENSANGP00000013342 [Anopheles gambiae str. PEST]
sw11935	BGIBMGA008301	0.84525	0.9884	0.84605	2.1113	2.00E-19	gi|29124593|gb|AAH49041.1| Mitochondrial ribosomal protein L14 [Danio rerio] mitochondrial ribosomal protein L14 [Danio rerio]
sw14651	BGIBMGA007269	0.3166	1.0556	0.89875	2.1101	3.00E-16	gi|72067168|ref|XP_795216.1| PREDICTED: similar to putative protein of bilaterial origin (49.2 kD) (3G698), partial [Strongylocentrotus purpuratus]
sw05401	BGIBMGA012017	0.7111	0.79425	0.8676	2.10895	No hits found	
sw16679	BGIBMGA009157	0.97995	1.05955	0.8194	2.10765	Bmb045273	7.00E-20
sw08752	BGIBMGA000157	1.2406	1.11285	0.95245	2.10705	4.00E-27	gi|76654393|ref|XP_876322.1| PREDICTED: similar to Transcription factor MafK (Erythroid transcription factor NF-E2 p18 subunit) [Bos taurus]
sw17949	BGIBMGA003741	0.7984	0.73215	0.7132	2.10615	3.00E-12	gi|72086632|ref|XP_791549.1| PREDICTED: similar to interferon gamma inducible protein 30 [Strongylocentrotus purpuratus]
sw08626	BGIBMGA008003	1.1495	1.2593	1.26135	2.10515	7.00E-11	gi|2827498|emb|CAA15707.1| EG:30B8.3 [Drosophila melanogaster]
sw06638	BGIBMGA005004	0.95575	1.06035	1.4765	2.10475	1.00E-19	gi|11121443|emb|CAC14874.1| zinc/iron regulated transporter-related protein 3, DZIP3 protein [Drosophila melanogaster]
sw21738	BGIBMGA009907	0.7359	0.8809	1.16655	2.10295	0	gi|49022793|dbj|BAD23983.1| broad-complex Z1-isoform [Bombyx mori] broad-complex B-Z1 isoform [Bombyx mori] broad-complex A-Z1 isoform [Bombyx mori] broad-complex Z1-isoform [Bombyx mori] Broad-Complex isoform Z1

sw14041	BGIBMGA001437	0.87095	0.9736	1.0348	2.10255	4.00E-80	gi|12654229|gb|AAH00934.1| Eukaryotic translation initiation factor 2 beta [Homo sapiens]
sw12550	BGIBMGA008222	1.07345	1.11515	0.7422	2.10105	2.00E-05	gi|33285891|gb|AAQ01563.1| promoting protein [Bombyx mori]
sw22929	BGIBMGA001351	1.8716	1.08665	1.16855	2.09995	2.00E-24	gi|62646831|ref|XP_579660.1| PREDICTED: aldo-keto reductase family 1, member D1 [Rattus norvegicus]
sw11669	BGIBMGA008893	1	1.19675	0.83055	2.09555	8.00E-31	gi|50760443|ref|XP_418024.1| PREDICTED: similar to Transcription initiation factor TFIID subunit 11 (Transcription initiation factor TFIID 28 kDa subunit) (TAF(II)28) (TAFII-28) (TAFII28) (TFIID subunit p30-beta) [Gallus gallus]
sw18379	BGIBMGA010388	1.1116	1.13135	1.309	2.0945	e-165	gi|38014373|gb|AAH60375.1| WD repeats and SOF domain containing 1 [Mus musculus] WD repeats and SOF domain containing 1 [Mus musculus]
sw15483	BGIBMGA005372	0.82225	0.9053	0.78275	2.09225	5.00E-28	gi|55642481|ref|XP_510542.1| PREDICTED: similar to Mesoderm development candidate 2 (UNQ1911/PRO4369) [Pan troglodytes] MESDC2 protein [Homo sapiens] MESDC2 protein [Homo sapiens] Y081
sw15076	BGIBMGA004021	1.11555	1.20605	1.3461	2.09185	No hits found	
sw21935	BGIBMGA003566	1.1065	1.4469	1.3035	2.09185	2.00E-99	gi|464960|sp|P35047|TRYC_MANSE Trypsin, alkaline C precursor
sw21310	BGIBMGA009925	1.16125	0.89465	1	2.09175	2.00E-79	gi|48094605|ref|XP_394221.1| PREDICTED: similar to ENSANGP00000024305 [Apis mellifera]
sw11036	BGIBMGA010197	0.52045	0.90595	0.73125	2.08785	1.00E-21	gi|76638444|ref|XP_580345.2| PREDICTED: similar to short coiled-coil protein [Bos taurus]
sw16392	BGIBMGA002349	0.84825	1.02495	0.77275	2.087	Bmb041108	2.00E-10
sw15596	BGIBMGA005274	1.08425	1.16075	0.77275	2.0861	Bmb033885	1.00E-53
sw06684	BGIBMGA002353	0.99935	0.9917	1.161	2.08535	e-104	gi|87248527|gb|ABD36316.1| lysophospholipase [Bombyx mori]
sw06803	BGIBMGA012859	0.89855	1.01225	0.9068	2.0851	Bmb035041	2.00E-76
sw05929	BGIBMGA003562	0.94725	0.93135	0.8557	2.08405	3.00E-30	gi|89272445|emb|CAJ83085.1| NADH dehydrogenase (ubiquinone) 1 alpha subcomplex, 5 [Xenopus tropicalis]
sw11628	BGIBMGA011105	0.9849	1	1	2.0833	No hits found	
sw22686	BGIBMGA005953	1.2381	1.1816	1.1779	2.0831	1.00E-67	gi|55234500|gb|EAA43295.2| ENSANGP00000022827 [Anopheles gambiae str. PEST] ENSANGP00000022827 [Anopheles gambiae str. PEST]
sw16116	BGIBMGA009046	1.26135	1.47885	1.2874	2.08155	8.00E-09	gi|1280151|gb|AAA98017.1| Nudix family protein 3 [Caenorhabditis elegans] NuDiX family member (ndx-3) [Caenorhabditis elegans] Nudix hydrolase 3
sw00949	BGIBMGA009329	1	1	1.0616	2.0807	1.00E-67	gi|58569859|gb|AAW79050.1| GekBS204P [Gekko japonicus]
sw15954	BGIBMGA002150	1.35635	1.36145	1.3165	2.0806	4.00E-05	gi|89271263|emb|CAJ82958.1| serum response factor binding protein 1 [Xenopus tropicalis]
sw11427	BGIBMGA011500	0.96845	0.9231	1.4399	2.07995	Bmb005745	e-106
sw19244	BGIBMGA000269	1.6252	0.66225	0.91305	2.07955	6.00E-28	gi|117622|sp|P11734|CU08_LOCMI Cuticle protein 8 (LM-8) (LM-ACP 8)
sw01225	BGIBMGA002001	1.05705	1.09535	0.9823	2.0794	No hits found	
sw13724	BGIBMGA006525	1.01035	1	0.88775	2.07735	4.00E-14	gi|62857681|ref|NP_001016776.1| DEAD (Asp-Glu-Ala-Asp) box polypeptide 18 [Xenopus tropicalis] DEAD (Asp-Glu-Ala-Asp) box polypeptide 18 [Xenopus tropicalis]
sw13775	BGIBMGA002945	1.28555	1.1993	1.07835	2.0768	7.00E-13	gi|66518233|ref|XP_392596.2| PREDICTED: similar to putative alcohol dehydrogenase [Apis mellifera]
sw16220	BGIBMGA009115	0.93695	1.1023	0.73275	2.07645	Bmb039222	8.00E-05
sw07775	BGIBMGA010385	0.83045	1.0916	0.93815	2.0764	2.00E-38	gi|85857488|gb|ABC86280.1| RE08574p [Drosophila melanogaster]
sw13145	BGIBMGA008021	1	1	1	2.07625	No hits found	

sw06309	BGIBMGA009041	0.955	1.1642	0.92185	2.0759	8.00E-18	gi|72171239|ref|XP_783401.1| PREDICTED: similar to zinc finger, HIT domain containing 1 [Strongylocentrotus purpuratus]
sw08844	BGIBMGA006775	1.0683	0.9359	0.95405	2.0759	1.00E-79	gi|6016491|sp|P55796|LEB3_BOMMO Lebocin-3 precursor (LEB 3) lebocin 3 [Bombyx mori]
sw18784	BGIBMGA003377	1.0114	0.996	1.0742	2.0736	4.00E-86	gi|72072160|ref|XP_787339.1| PREDICTED: similar to replication factor C subunit RFC4 [Strongylocentrotus purpuratus]
sw13224	BGIBMGA013781	1.139	1.14905	0.7665	2.0734	3.00E-07	gi|3868784|dbj|BAA34219.1| MBF2 [Samia cynthia]
sw21082	BGIBMGA005521	1.1005	1.1813	1.2816	2.07265	Bmb021757	2.00E-44
sw02328	BGIBMGA001396	0.7687	0.95055	0.74415	2.07175	4.00E-12	gi|72045503|ref|XP_797188.1| PREDICTED: similar to butyrate-induced transcript 1 [Strongylocentrotus purpuratus]
sw00838	BGIBMGA010590	0.9947	1.8814	1.8615	2.0716	9.00E-90	gi|51094376|gb|AAT95356.1| trypsin III precursor [Sesamia nonagrioides]
sw17121	BGIBMGA012220	0.8696	1.08255	0.99725	2.0715	2.00E-20	gi|66512914|ref|XP_623446.1| PREDICTED: similar to Tetratricopeptide repeat protein 1 (TPR repeat protein 1) [Apis mellifera]
sw00917	BGIBMGA004302	0.79015	1.0056	1.2162	2.0709	e-120	gi|27462592|gb|AAO15491.1| eIF2 alpha subunit [Spodoptera frugiperda]
sw18990	BGIBMGA005827	0.8581	1.011	1.0819	2.06895	7.00E-33	gi|66518233|ref|XP_392596.2| PREDICTED: similar to putative alcohol dehydrogenase [Apis mellifera]
sw16014	BGIBMGA004044	1.6034	1	2.6336	2.0674	Bmb037504	5.00E-11
sw11213	BGIBMGA005002	0.8297	1.18	0.83805	2.06725	2.00E-94	gi|72048292|ref|XP_784158.1| PREDICTED: similar to potassium channel tetramerisation domain containing 3 [Strongylocentrotus purpuratus]
sw15512	BGIBMGA004019	0.68675	0.98245	0.81985	2.06715	7.00E-42	gi|49523218|gb|AAH75271.1| Thyroid autoantigen 70kDa (Ku antigen) [Xenopus tropicalis] thyroid autoantigen 70kDa (Ku antigen) [Xenopus tropicalis]
sw01796	BGIBMGA012009	1.2347	0.9317	0.9907	2.0652	Bmb028063	No hits found
sw12743	BGIBMGA008595	0.66385	0.6982	0.74595	2.0638	2.00E-85	gi|68390942|ref|XP_683479.1| PREDICTED: similar to proteasome 26S non-ATPase subunit 8 [Danio rerio]
sw15831	BGIBMGA006266	1	1	1	2.0635	No hits found	
sw06503	BGIBMGA011204	1.03045	0.9386	1.02885	2.06245	2.00E-78	gi|13360618|dbj|BAB34581.1| periplasmic glucose-1-phosphatase [Escherichia coli O157:H7] periplasmic glucose-1-phosphatase [Escherichia coli O157:H7] periplasmic glucose-1-phosphatase
sw06403	BGIBMGA013366	0.83885	0.9135	0.71995	2.0607	1.00E-60	gi|66550162|ref|XP_396097.2| PREDICTED: similar to Zgc:103697 [Apis mellifera]
sw17345	BGIBMGA006913	1.0225	0.94225	0.89265	2.0601	7.00E-15	gi|9864185|gb|AAG01337.1| Crossveinless 2 [Drosophila melanogaster]
sw15802	BGIBMGA010446	1.3912	1.21075	1.29625	2.0597	Bmb035584	1.00E-40
sw13997	BGIBMGA003583	1	1.6518	1	2.05765	Bmb022107	4.00E-07
sw13300	BGIBMGA008477	0.7111	1.03885	0.75545	2.0567	9.00E-45	gi|62083517|gb|AAX62483.1| mitochondrial ribosomal protein L17 [Lysiphlebus testaceipes]
sw22672	BGIBMGA003402	0.61755	0.73525	0.7417	2.05635	Bmb028151	2.00E-48
sw11289	BGIBMGA000898	0.98995	1.01045	1.0686	2.05485	Bmb004833	4.00E-40
sw20321	BGIBMGA000232	0.7756	0.90565	0.8371	2.0538	1.00E-38	gi|6560653|gb|AAF16704.1| calcyphosine-like protein [Manduca sexta]
sw16093	BGIBMGA008194	0.8624	0.94485	0.9479	2.0521	1.00E-31	gi|66500960|ref|XP_395396.2| PREDICTED: similar to ENSANGP00000024290 [Apis mellifera]
sw22941	BGIBMGA005488	0.7896	0.85845	0.9228	2.0508	e-126	gi|87248211|gb|ABD36158.1| replication factor C (activator 1) 5 [Bombyx mori]
sw19766	BGIBMGA007829	1.0696	1.36325	1.40585	2.0475	e-162	gi|6691117|gb|AAF24495.1| SP71 [Drosophila melanogaster]

sw03637	BGIBMGA009232	1.4407	1.0586	1.0158	2.0473	9.00E-30	gi|71557964|gb|AAZ37175.1| aldose 1-epimerase [Pseudomonas syringae pv. phaseolicola 1448A] aldose 1-epimerase [Pseudomonas syringae pv. phaseolicola 1448A]
sw07546	BGIBMGA005799	1.04505	0.89325	1.05215	2.0451	5.00E-44	gi|34871962|ref|XP_341067.1| PREDICTED: similar to Williams-Beuren syndrome chromosome region 16 homolog [Rattus norvegicus]
sw10137	BGIBMGA005565	0.5099	1.22355	0.54005	2.04355	Bmb044116	5.00E-16
sw03095	BGIBMGA009277	1.1129	0.9976	0.90815	2.04345	Bmb003877	7.00E-22
sw12631	BGIBMGA006990	1.21425	1.30555	1.30935	2.04265	1.00E-40	gi|55242673|gb|EAA07189.2| ENSANGP00000013724 [Anopheles gambiae str. PEST] ENSANGP00000013724 [Anopheles gambiae str. PEST]
sw03975	BGIBMGA006641	0.73255	1.1262	0.89385	2.04065	e-118	gi|87248395|gb|ABD36250.1| ras-related GTP-binding protein 4b [Bombyx mori]
sw02046	BGIBMGA009240	1.24405	1.06895	0.7687	2.04055	4.00E-35	gi|62859495|ref|NP_001017001.1| NMDA receptor regulated 1-like [Xenopus tropicalis]
sw05918	BGIBMGA003136	0.9144	1.03445	1.1242	2.04045	4.00E-12	gi|28839294|gb|AAH47776.1| FLJ31795 protein [Homo sapiens]
sw06198	BGIBMGA011602	1.08725	1.0242	0.79495	2.0399	1.00E-08	gi|66516901|ref|XP_393666.2| PREDICTED: similar to mKIAA0981 protein [Apis mellifera]
sw09451	BGIBMGA007508	2.2004	1.42785	1.2052	2.0384	5.00E-76	gi|73991331|ref|XP_542897.2| PREDICTED: similar to Hydroxyacid oxidase 1 (HAOX1) (Glycolate oxidase) (GOX) isoform 1 [Canis familiaris]
sw03170	BGIBMGA012069	1.36325	1.44845	1.47215	2.0364	e-127	gi|72141411|ref|XP_793660.1| PREDICTED: similar to RIO kinase 1 [Strongylocentrotus purpuratus]
sw05224	BGIBMGA004064	1.10595	1.0799	0.65365	2.0362	0	gi|87248335|gb|ABD36220.1| glutamyl-tRNA amidotransferase subunit B [Bombyx mori]
sw21123	BGIBMGA000056	1	1.0062	1	2.03525	No hits found	
sw01951	BGIBMGA000951	1.25055	1.2173	1.26465	2.03415	No hits found	
sw14689	BGIBMGA014140	1.5652	1.2809	1.57725	2.03415	No hits found	
sw22883	BGIBMGA000955	1.20195	1.20675	1.10365	2.03405	5.00E-37	gi|27819803|gb|AAO24950.1| RE53824p [Drosophila melanogaster]
sw12476	BGIBMGA002640	0.6549	0.7802	0.78455	2.033	5.00E-66	gi|54035222|gb|AAH84116.1| LOC495025 protein [Xenopus laevis]
sw20366	BGIBMGA013929	0.8701	0.64285	0.824	2.0304	5.00E-25	gi|90025232|gb|ABD85119.1| juvenile hormone epoxide hydrolase [Spodoptera exigua]
sw11949	BGIBMGA007545	0.84555	1.2806	1.6103	2.03015	5.00E-56	gi|38679389|gb|AAR26516.1| antennal esterase [Mamestra brassicae]
sw09590	BGIBMGA007098	1.172	1.11775	0.8985	2.02955	Bmb032267	5.00E-89
sw11955	BGIBMGA002750	1.11465	0.8622	1.08295	2.02895	1.00E-70	gi|25012844|gb|AAN71511.1| RH05604p [Drosophila melanogaster]
sw12248	BGIBMGA008807	1.21135	0.72715	1	2.02765	1.00E-64	gi|57914803|ref|XP_555244.1| ENSANGP00000027403 [Anopheles gambiae str. PEST] ENSANGP00000027403 [Anopheles gambiae str. PEST]
sw04590	BGIBMGA005733	1.1634	1.2771	1.0759	2.0274	Bmb039328	3.00E-52
sw21942	BGIBMGA006982	0.3242	0.8611	0.8828	2.02655	No hits found	
sw10670	BGIBMGA013129	0.3553	1.1206	0.7204	2.0257	7.00E-14	gi|66511554|ref|XP_393207.2| PREDICTED: similar to glucocerebrosidase precursor [Apis mellifera]
sw01501	BGIBMGA008552	0.53265	1	1	2.02405	7.00E-31	gi|77455216|gb|ABA86417.1| CG9211 [Drosophila erecta]
sw01054	BGIBMGA013884	0.9779	1.0388	1.16345	2.0235	2.00E-27	gi|17863026|gb|AAL39990.1| SD09147p [Drosophila melanogaster]
sw21138	BGIBMGA007107	1.0425	0.9319	1.2476	2.02195	0	gi|87248243|gb|ABD36174.1| TPR-repeat protein [Bombyx mori]

sw16490	BGIBMGA014278	1.48445	1.1758	0.993	2.02115	Bmb042238	No hits found
sw13603	BGIBMGA003202	1.1971	1.2367	1.1439	2.02075	4.00E-29	gi|4007492|gb|AAC95306.1| SNF4/AMP-activated protein kinase gamma subunit; SNF4A; noncatalytic subunit of the SNF/AMPK complex [Drosophila melanogaster]
sw11307	BGIBMGA013398	0.7905	1.0672	1.29785	2.02015	No hits found	
sw09395	BGIBMGA004965	0.20365	0.63825	0.65895	2.01865	2.00E-59	gi|22138087|gb|AAM93421.1| ORF 4 [Spodoptera littoralis nucleopolyhedrovirus]
sw19688	BGIBMGA007361	1.41945	1.22995	0.99695	2.01845	1.00E-16	gi|47523722|ref|NP_999496.1| L-3-hydroxyacyl-CoA dehydrogenase [Sus scrofa] L-3-hydroxyacyl-CoA dehydrogenase [Sus scrofa] Short chain 3-hydroxyacyl-CoA dehydrogenase
sw00316	BGIBMGA003815	1	1.08595	1.22755	2.01795	e-112	gi|220659|dbj|BAA14397.1| dihydrolipoamide succinyltransferase [Rattus norvegicus]
sw16057	BGIBMGA010948	1.2905	1.0674	0.82485	2.0176	Bmb037901	1.00E-17
sw13618	BGIBMGA000777	0.8499	0.9974	1.09255	2.01735	7.00E-77	gi|66512713|ref|XP_394697.2| PREDICTED: similar to carboxylesterase [Apis mellifera]
sw03397	BGIBMGA009613	1.1371	1.0537	1.1303	2.01645	8.00E-27	gi|26986579|ref|NP_758476.1| prolyl-tRNA synthetase (mitochondrial)(putative) [Mus musculus] CDNA sequence BC027073 [Mus musculus] Probable prolyl-tRNA synthetase, mitochondrial precursor (Proline--tRNA ligase) (ProRS)
sw11377	BGIBMGA012905	0.8598	1.0147	1.10975	2.0158	4.00E-28	gi|48104416|ref|XP_395776.1| PREDICTED: similar to small GTPase Ran binding protein 1; RanBP1 [Apis mellifera]
sw03140	BGIBMGA007977	0.94285	1.1189	1.34635	2.01455	6.00E-68	gi|76632367|ref|XP_868926.1| PREDICTED: similar to chromosome 10 open reading frame 97 isoform 2 [Bos taurus]
sw18133	BGIBMGA009182	0.97195	1.10875	0.88315	2.01425	2.00E-11	gi|72077797|ref|XP_783578.1| PREDICTED: similar to NADH dehydrogenase (ubiquinone) 1 beta subcomplex 3 [Strongylocentrotus purpuratus]
sw06948	BGIBMGA012590	0.1895	0.60005	0.8042	2.0137	No hits found	
sw13832	BGIBMGA012241	0.2067	0.5821	0.7234	2.0136	3.00E-08	gi|21357209|ref|NP_648881.1| CG4893-PA [Drosophila melanogaster] RE55690p [Drosophila melanogaster] RE39465p [Drosophila melanogaster] CG4893-PA [Drosophila melanogaster] Hypothetical protein CG4893
sw06115	BGIBMGA005687	0.91045	1.2451	1.28705	2.0118	2.00E-75	gi|16877082|gb|AAH16818.1| Phosphomannomutase 1 [Homo sapiens] Phosphomannomutase 1 [Homo sapiens] OTTHUMP00000028766 [Homo sapiens] PMM1 [Homo sapiens] phosphomannomutase
sw09793	BGIBMGA000803	0.91535	1.0547	1.07175	2.01055	Bmb036034	6.00E-61
sw13862	BGIBMGA009465	0.92615	1.16985	1.06955	2.01035	5.00E-16	gi|55243014|gb|EAA06835.2| ENSANGP00000019057 [Anopheles gambiae str. PEST] ENSANGP00000019057 [Anopheles gambiae str. PEST]
sw20478	BGIBMGA001956	1.0064	1.03045	1.08095	2.01005	2.00E-54	gi|28569894|dbj|BAC57926.1| reverse transcriptase [Bombyx mori]
sw13350	BGIBMGA000331	3.0576	0.79175	1.4101	2.0093	4.00E-24	gi|85857724|gb|ABC86397.1| IP09958p [Drosophila melanogaster]
sw05428	BGIBMGA004926	1.025	1.14345	1.0486	2.00845	e-114	gi|66519538|ref|XP_393739.2| PREDICTED: similar to CG6194-PA [Apis mellifera]
sw05239	BGIBMGA013876	1	1	1	2.007	No hits found	
sw19411	BGIBMGA009554	0.82175	0.73335	0.98795	2.0039	9.00E-95	gi|76576582|gb|ABA53944.1| interleukin enhancer binding factor 2 [Ctenopharyngodon idella]
sw22661	BGIBMGA013207	0.406	0.9129	0.339	1.99965	2.00E-05	gi|74835158|dbj|BAE44464.1| non-LTR retrotransposon CATS [Bombyx mori]
sw18380	BGIBMGA004567	2.4561	2.6275	2.37325	1.99215	8.00E-45	gi|62637998|gb|AAX92638.1| glucose transporter 8 [Solenopsis invicta]
sw14949	BGIBMGA007192	2.15215	1.88055	1.6135	1.98365	3.00E-30	gi|89273996|emb|CAJ81644.1| novel protein [Xenopus tropicalis] nin one binding protein [Xenopus tropicalis]
sw20178	BGIBMGA012263	2.46505	0.9876	0.54905	1.96755	1.00E-07	gi|66550559|ref|XP_397430.2| PREDICTED: similar to sensory neuron membrane protein 1 [Apis mellifera]
sw05253	BGIBMGA002865	0.3305	1	0.65885	1.9608	No hits found	
sw16237	BGIBMGA006221	2.87735	1.8662	1.7472	1.9405	2.00E-85	gi|16758368|ref|NP_446041.1| RAB14, member RAS oncogene family [Rattus norvegicus] Ras-related protein Rab-14 [Sus scrofa] small GTP binding protein RAB14 [Homo sapiens] Ras-related protein Rab-14

sw01401	BGIBMGA014092	0.4223	0.65125	0.6544	1.9386	No hits found	
sw20518	BGIBMGA011987	0.42725	0.7452	0.87945	1.92895	3.00E-31	gi|72027838|ref|XP_794566.1| PREDICTED: similar to RNA pseudouridylate synthase domain containing 1 [Strongylocentrotus purpuratus]
sw09592	BGIBMGA006282	0.45845	0.6816	0.72985	1.8994	No hits found	
sw19025	BGIBMGA004509	0.32325	1.2454	0.9246	1.88205	1.00E-39	gi|55238404|gb|EAA11457.2| ENSANGP00000007253 [Anopheles gambiae str. PEST] ENSANGP00000007253 [Anopheles gambiae str. PEST]
sw10798	BGIBMGA007677	1.92275	1.15735	2.6117	1.87125	Bmb001707	e-113
sw17805	BGIBMGA002747	0.56795	0.4119	0.5943	1.85365	e-112	gi|994849|gb|AAB34519.1| attacin [Bombyx mori] Attacin precursor (Nuecin) nuecin [Bombyx mori]
sw06356	BGIBMGA013920	0.1621	0.632	0.5895	1.8354	5.00E-87	gi|66500267|ref|XP_623297.1| PREDICTED: hypothetical protein XP_623294 [Apis mellifera]
sw22099	BGIBMGA005830	0.4342	1.049	0.8879	1.83205	3.00E-32	gi|66518233|ref|XP_392596.2| PREDICTED: similar to putative alcohol dehydrogenase [Apis mellifera]
sw02728	BGIBMGA009558	0.40415	0.3566	1	1.8314	Bmb048846	8.00E-08
sw14234	BGIBMGA005803	0.1035	0.54945	0.5441	1.81435	No hits found	
sw16564	BGIBMGA011226	0.4485	0.97835	1.34815	1.79695	Bmb043407	4.00E-18
sw07605	BGIBMGA001007	2.0245	1.3865	1.4812	1.7926	2.00E-58	gi|55243048|gb|EAA06899.2| ENSANGP00000001582 [Anopheles gambiae str. PEST] ENSANGP00000001582 [Anopheles gambiae str. PEST]
sw12914	BGIBMGA001839	0.09235	0.61305	0.6087	1.7825	No hits found	
sw00666	BGIBMGA007431	1.0868	1.16955	2.06445	1.77845	2.00E-70	gi|72014604|ref|XP_787398.1| PREDICTED: similar to Aladin (Adracalin) [Strongylocentrotus purpuratus]
sw12271	BGIBMGA013646	0.57505	0.4812	0.62255	1.7662	2.00E-58	gi|33989989|gb|AAH56253.1| Chromosome 6 open reading frame 71 [Homo sapiens]
sw03956	BGIBMGA004026	0.1861	0.593	0.66175	1.7579	1.00E-24	gi|49250900|gb|AAH74631.1| MGC69557 protein [Xenopus tropicalis] MGC82513 protein [Xenopus laevis] MGC69557 protein [Xenopus tropicalis]
sw18893	BGIBMGA004121	1.5707	1.47525	2.24395	1.74305	No hits found	
sw20121	BGIBMGA002626	0.4386	0.82735	0.4647	1.74195	8.00E-59	gi|87248601|gb|ABD36353.1| antennal binding protein [Bombyx mori]
sw16511	BGIBMGA010854	1.969	2.19685	1.14545	1.72535	Bmb042638	No hits found
sw11029	BGIBMGA010715	0.1905	1	0.678	1.7076	1.00E-24	gi|55644291|ref|XP_511124.1| PREDICTED: similar to dynein, cytoplasmic, light polypeptide 2B; dynein light chain 2B; bithoraxoid-like protein; roadblock/LC7-like [Pan troglodytes]
sw22410	BGIBMGA006052	0.6745	0.5745	0.46885	1.7041	No hits found	
sw22573	BGIBMGA011001	2.13365	2.94145	2.3798	1.6842	0	gi|87248447|gb|ABD36276.1| aminoacylase [Bombyx mori]
sw08108	BGIBMGA001333	2.2427	1.59245	1	1.6743	0	gi|76156035|gb|ABA39861.1| uricase [Bombyx mori]
sw13079	BGIBMGA005673	2.05965	2.7684	2.0015	1.67305	6.00E-91	gi|24583491|ref|NP_723607.1| CG31871-PA [Drosophila melanogaster] RE24765p [Drosophila melanogaster] CG31871-PA [Drosophila melanogaster]
sw21386	BGIBMGA010537	1.46275	2.0431	1.902	1.6639	e-155	gi|2970687|gb|AAC06038.1| beta-glucosidase precursor [Spodoptera frugiperda]
sw13039	BGIBMGA005820	0.04305	0.52395	0.49515	1.6501	No hits found	
sw07934	BGIBMGA010161	1.25645	3.1151	1	1.6297	2.00E-14	gi|28317050|gb|AAO39544.1| RE06501p [Drosophila melanogaster]
sw05120	BGIBMGA012291	2.11725	1.2521	1.67405	1.62755	No hits found	

sw15361	BGIBMGA004910	0.48405	0.96	0.72475	1.62115	6.00E-25	gi|27819749|gb|AAL68388.2| SD08771p [Drosophila melanogaster]
sw17642	BGIBMGA001796	0.45075	0.64005	0.72715	1.61055	2.00E-05	gi|89293948|gb|EAR91936.1| Viral A-type inclusion protein repeat containing protein [Tetrahymena thermophila SB210]
sw02976	BGIBMGA003178	0.51045	0.83475	1.232	1.61015	4.00E-59	gi|20070698|gb|AAH26131.1| Solute carrier family 7 (cationic amino acid transporter, y+ system), member 5 [Mus musculus] solute carrier family 7 (cationic amino acid transporter, y+ system), member 5 [Mus musculus]
sw14496	BGIBMGA004947	0.2469	1	1	1.6076	9.00E-50	gi|40789008|dbj|BAA76826.2| KIAA0982 protein [Homo sapiens]
sw01229	BGIBMGA005862	2.71685	2.74305	1.7736	1.6006	1.00E-37	gi|55650502|ref|XP_514458.1| PREDICTED: similar to Protein C20orf54 [Pan troglodytes]
sw19517	BGIBMGA004538	0.391	1.31275	1.037	1.57195	2.00E-45	gi|55238404|gb|EAA11457.2| ENSANGP00000007253 [Anopheles gambiae str. PEST] ENSANGP00000007253 [Anopheles gambiae str. PEST]
sw20638	BGIBMGA009632	0.37115	0.7689	0.59055	1.5684	8.00E-12	gi|5857|emb|CAA41354.1| chorion protein [Bombyx mori] Chorion class CA protein ERA.5 precursor
sw02025	BGIBMGA010053	0.38565	0.5425	0.50505	1.56835	No hits found	
sw11960	BGIBMGA006713	0.36595	0.68325	0.49725	1.56145	9.00E-69	gi|5139701|dbj|BAA81694.1| G protein a subunit 2 [Hydra magnipapillata]
sw02991	BGIBMGA013936	0.3368	0.67425	0.7889	1.55935	0	gi|13359380|dbj|BAB33388.1| testis specific tektin [Bombyx mori]
sw01901	BGIBMGA002236	0.28045	0.8298	0.31755	1.5482	3.00E-92	gi|28416323|gb|AAO42634.1| SD07139p [Drosophila melanogaster] Negative elongation factor A homolog
sw19507	BGIBMGA012332	0.0421	0.517	0.58365	1.5406	2.00E-06	gi|55235101|gb|EAL38938.1| ENSANGP00000005174 [Anopheles gambiae str. PEST] ENSANGP00000005174 [Anopheles gambiae str. PEST]
sw06169	BGIBMGA013520	2.0081	1	1	1.53735	No hits found	
sw16532	BGIBMGA000158	0.13745	0.15465	0.2374	1.5367	4.00E-40	gi|72019779|ref|XP_792493.1| PREDICTED: similar to choline dehydrogenase precursor [Strongylocentrotus purpuratus]
sw05294	BGIBMGA009058	0.1442	1	0.74055	1.536	8.00E-31	gi|66522084|ref|XP_624399.1| PREDICTED: similar to Mitochondrial 2-oxoglutarate/malate carrier protein (OGCP) [Apis mellifera]
sw15101	BGIBMGA000289	0.1329	0.5571	0.5306	1.53455	Bmb030144	5.00E-05
sw14925	BGIBMGA011658	2.0413	1.85725	1.2505	1.53155	e-143	gi|87248507|gb|ABD36306.1| glutathione S-transferase omega 2 [Bombyx mori]
sw08914	BGIBMGA001535	0.055	0.6105	0.67405	1.5292	No hits found	
sw05701	BGIBMGA009102	2.46035	1.435	1.73715	1.528	3.00E-64	gi|48108726|ref|XP_393098.1| PREDICTED: similar to ENSANGP00000022305 [Apis mellifera]
sw05687	BGIBMGA002005	0.3162	0.64595	0.7022	1.5251	e-128	gi|28207648|gb|AAO32325.1| ADP/ATP translocase [Manduca sexta]
sw05107	BGIBMGA004606	0.554	0.4493	0.64175	1.51345	8.00E-55	gi|56378319|dbj|BAD74196.1| heat shock protein hsp20.1 [Bombyx mori]
sw16452	BGIBMGA001837	0.25365	0.72675	0.7387	1.5087	No hits found	
sw01237	BGIBMGA011236	0.12595	0.406	0.38245	1.49555	No hits found	
sw16082	BGIBMGA011795	0.31255	1.01625	0.45005	1.49255	No hits found	
sw08854	BGIBMGA010328	2.7317	1.72955	1.1654	1.49245	Bmb020629	2.00E-53
sw13009	BGIBMGA011468	1	1.7228	3.34165	1.47585	e-180	gi|1276940|gb|AAC47018.1| juvenile hormone epoxide hydrolase Juvenile hormone epoxide hydrolase (Juvenile hormone-specific epoxide hydrolase) (JHEH) epoxide hydrolase
sw03184	BGIBMGA004650	0.08115	0.5321	0.45785	1.47155	3.00E-15	gi|44982930|gb|AAS52160.1| ADR240Cp [Ashbya gossypii ATCC 10895] ADR240Cp [Eremothecium gossypii]
sw13683	BGIBMGA014380	0.03185	0.5906	0.57315	1.46215	No hits found	

sw20205	BGIBMGA005784	0.19215	0.63865	0.7535	1.45735	9.00E-33	gi|56378319|dbj|BAD74196.1| heat shock protein hsp20.1 [Bombyx mori]
sw18395	BGIBMGA012952	1.75415	1.59635	2.36365	1.45295	1.00E-32	gi|72152404|ref|XP_792972.1| PREDICTED: similar to glucosamine (N-acetyl)-6-sulfatase (Sanfilippo disease IIID), partial [Strongylocentrotus purpuratus]
sw18642	BGIBMGA003409	2.094	1.27905	1.50525	1.45265	Bmb033794	1.00E-09
sw07308	BGIBMGA000677	0.4512	0.8003	0.7996	1.44925	3.00E-30	gi|62857673|ref|NP_001016778.1| EF hand calcium binding domain 1 [Xenopus tropicalis]
sw15263	BGIBMGA013409	0.01085	0.4317	0.54235	1.438	No hits found	
sw20421	BGIBMGA008633	0.37615	0.56785	0.47385	1.43765	7.00E-83	gi|73987978|ref|XP_860745.1| PREDICTED: similar to protein phosphatase methylesterase 1 isoform 4 [Canis familiaris] PREDICTED: similar to protein phosphatase methylesterase 1 isoform 1 [Canis familiaris]
sw08704	BGIBMGA007217	0.18045	0.5084	0.36645	1.43445	1.00E-95	gi|83306196|emb|CAH59462.1| ATP-binding cassette transporter sub-family A [Pecten maximus]
sw21141	BGIBMGA003347	0.4338	0.8682	0.7294	1.4326	2.00E-05	gi|27374266|gb|AAO01023.1| l(2)06496-PA [Drosophila erecta]
sw11832	BGIBMGA001003	2.4203	1.16765	1.91535	1.43025	8.00E-77	gi|58429859|gb|AAW78325.1| cytochrome P450 family 4 [Chironomus tentans]
sw04660	BGIBMGA003100	0.2274	0.56695	0.37145	1.42805	Bmb041587	8.00E-06
sw14045	BGIBMGA013186	0.16705	1	0.50235	1.41665	No hits found	
sw06179	BGIBMGA000997	0.0611	0.55095	0.589	1.40755	4.00E-13	gi|14625425|dbj|BAB61919.1| calmodulin NtCaM13 [Nicotiana tabacum]
sw12103	BGIBMGA004130	2.59595	1.17535	1.7725	1.40755	e-109	gi|55239333|gb|EAA44260.2| ENSANGP00000025275 [Anopheles gambiae str. PEST] ENSANGP00000025275 [Anopheles gambiae str. PEST]
sw06897	BGIBMGA003745	0.08205	0.54615	0.6153	1.40645	No hits found	
sw08745	BGIBMGA003530	0.23005	0.54185	0.4096	1.40515	7.00E-09	gi|24651106|ref|NP_733300.1| CG15506-PB, isoform B [Drosophila melanogaster] CG15506-PB, isoform B [Drosophila melanogaster]
sw00379	BGIBMGA000562	0.45865	1.03475	0.8985	1.4046	9.00E-44	gi|89243302|gb|ABD64815.1| Dvir_CG10440 [Drosophila virilis]
sw08167	BGIBMGA002337	0.23175	0.67295	0.65755	1.3936	e-130	gi|86450230|gb|ABC96322.1| enolase [Blattella germanica]
sw17553	BGIBMGA004115	2.43175	2.73085	4.9723	1.3839	No hits found	
sw20815	BGIBMGA000386	0.1438	0.5122	0.6586	1.3799	4.00E-27	gi|68585|pir||AJFF2C glutamate-ammonia ligase (EC 6.3.1.2) 2, cytosolic - fruit fly (Drosophila melanogaster)
sw17004	BGIBMGA012421	0.1193	0.50345	0.54575	1.375	No hits found	
sw01141	BGIBMGA007900	0.4986	0.83625	0.667	1.3698	1.00E-24	gi|30178562|gb|EAA45375.1| ENSANGP00000023091 [Anopheles gambiae str. PEST] ENSANGP00000023091 [Anopheles gambiae str. PEST]
sw03054	BGIBMGA000712	0.1957	0.6232	0.52695	1.3555	No hits found	
sw12305	BGIBMGA010642	0.3678	0.541	0.51485	1.3534	2.00E-40	gi|71122386|gb|AAH99752.1| Uridine phosphorylase 1 [Rattus norvegicus] uridine phosphorylase 1 [Rattus norvegicus]
sw09312	BGIBMGA012380	0.24605	0.7174	0.75325	1.35075	e-106	gi|38181637|gb|AAH61597.1| Solute carrier family 25 (mitochondrial carrier; phosphate carrier), member 3 [Xenopus tropicalis] solute carrier family 25 (mitochondrial carrier; phosphate carrier), member 3
sw15356	BGIBMGA007043	0.4859	0.88495	0.6543	1.3428	3.00E-21	gi|72153363|ref|XP_788906.1| PREDICTED: similar to Microsomal signal peptidase 12 kDa subunit (SPase 12 kDa subunit) (SPC12) [Strongylocentrotus purpuratus]
sw07075	BGIBMGA014151	0.12175	0.6052	0.52245	1.34165	Bmb042375	No hits found
sw20455	BGIBMGA003345	2.6338	1.11045	1.03945	1.33455	1.00E-77	gi|73992800|dbj|BAE43413.1| hypothetical protein [Bombyx mori]
sw07617	BGIBMGA009108	2.12965	1.62525	1.296	1.33405	No hits found	

sw06237	BGIBMGA004229	1.48985	1.6544	2.36655	1.3332	5.00E-48	gi|28317060|gb|AAO39549.1| RE03380p [Drosophila melanogaster]
sw07690	BGIBMGA002991	0.1687	0.5494	0.7426	1.3217	No hits found	
sw15429	BGIBMGA001230	2.81275	1.3617	1	1.3186	Bmb032572	1.00E-84
sw13032	BGIBMGA005677	3.0774	0.9714	1.12555	1.31795	Bmb015897	No hits found
sw17947	BGIBMGA006176	2.3659	2.10905	1.58395	1.31595	4.00E-41	gi|76779438|gb|AAI06213.1| LOC446918 protein [Xenopus laevis]
sw11317	BGIBMGA002073	2.0433	1	1	1.31415	3.00E-52	gi|27819801|gb|AAO24949.1| RE59468p [Drosophila melanogaster]
sw22679	BGIBMGA012648	0.10245	0.7368	0.39975	1.31075	9.00E-59	gi|66555620|ref|XP_624966.1| PREDICTED: similar to CG6113-PA [Apis mellifera]
sw04514	BGIBMGA001676	0.0491	0.4886	0.56765	1.3049	No hits found	
sw07989	BGIBMGA013726	0.26565	0.6998	0.74325	1.2958	6.00E-05	gi|66510443|ref|XP_397485.2| PREDICTED: similar to CG6653-PA [Apis mellifera]
sw22922	BGIBMGA003885	0.3736	0.6353	0.7347	1.2914	1.00E-22	gi|27374204|gb|AAN87276.1| CG13533 [Drosophila virilis]
sw14594	BGIBMGA010881	0.31	1.9238	0.83185	1.28695	5.00E-18	gi|28316894|gb|AAO39469.1| RH04286p [Drosophila melanogaster]
sw11277	BGIBMGA004311	0.07265	0.6839	0.60645	1.28575	9.00E-06	gi|56270499|gb|AAH87533.1| LOC496100 protein [Xenopus laevis]
sw07704	BGIBMGA007038	0.71165	0.49895	0.66005	1.2838	6.00E-56	gi|25012350|gb|AAN71285.1| RE06328p [Drosophila melanogaster]
sw11301	BGIBMGA007153	2.1025	1.39835	1.7561	1.27425	3.00E-32	gi|76258691|ref|ZP_00766345.1| Glycoside hydrolase, family 31 [Chloroflexus aurantiacus J-10-fl] Glycoside hydrolase, family 31 [Chloroflexus aurantiacus J-10-fl]
sw14637	BGIBMGA011698	0.4696	0.6479	0.5996	1.2709	7.00E-29	gi|66564529|ref|XP_623716.1| PREDICTED: similar to Ribonuclease UK114 (14.5 kDa translational inhibitor protein) (p14.5) (UK114 antigen homolog) [Apis mellifera]
sw14400	BGIBMGA005753	5.3759	1.3806	1.69195	1.2636	No hits found	
sw11507	BGIBMGA000891	0.20415	0.60395	0.8261	1.26105	1.00E-05	gi|72016498|ref|XP_783913.1| PREDICTED: similar to Gamma-aminobutyric-acid receptor alpha-5 subunit precursor (GABA(A) receptor) [Strongylocentrotus purpuratus]
sw14063	BGIBMGA010015	0.49955	0.8601	0.6799	1.25845	No hits found	
sw20654	BGIBMGA002525	0.45865	0.8692	0.49035	1.25505	e-112	gi|55239363|gb|EAL40125.1| ENSANGP00000029489 [Anopheles gambiae str. PEST] ENSANGP00000029489 [Anopheles gambiae str. PEST]
sw02096	BGIBMGA001320	0.2954	0.14645	0.75875	1.25435	1.00E-35	gi|51094358|gb|AAT95347.1| trypsin Ia precursor [Sesamia nonagrioides]
sw07346	BGIBMGA003969	1	1	4.19895	1.25115	3.00E-28	gi|25012479|gb|AAN71344.1| RE26896p [Drosophila melanogaster]
sw07062	BGIBMGA012939	0.45575	0.43955	0.6009	1.2498	4.00E-77	gi|66546491|ref|XP_624276.1| PREDICTED: similar to secretory component [Apis mellifera]
sw11397	BGIBMGA010747	2.25645	1.88045	1.55425	1.24855	3.00E-07	gi|14286129|sp|P32866|GPRK2_DROME G protein-coupled receptor kinase 2
sw11347	BGIBMGA009838	1.6618	2.4447	1.69925	1.24725	3.00E-98	gi|55235355|gb|EAA14774.2| ENSANGP00000019555 [Anopheles gambiae str. PEST] ENSANGP00000019555 [Anopheles gambiae str. PEST]
sw09094	BGIBMGA004546	2.01475	1.4162	1.34055	1.2466	No hits found	
sw06317	BGIBMGA007254	2.11605	1.1382	0.7737	1.2429	e-142	gi|86450717|gb|ABC96694.1| yellow1 [Bombyx mori]
sw03939	BGIBMGA008460	0.29135	0.4863	0.5618	1.2359	No hits found	
sw20164	BGIBMGA009865	0.4445	0.6979	0.56615	1.23175	Bmb043467	3.00E-37

sw07726	BGIBMGA010976	0.2541	0.4517	0.34085	1.22745	2.00E-76	gi|66560187|ref|XP_392698.2| PREDICTED: similar to carboxylesterase [Apis mellifera]
sw15683	BGIBMGA004120	0.2872	0.64095	0.90655	1.22245	5.00E-27	gi|68443719|ref|XP_701274.1| PREDICTED: similar to inhibitor of Brutons tyrosine kinase, partial [Danio rerio]
sw14467	BGIBMGA006789	0.4814	1.1096	1.04935	1.22225	2.00E-75	gi|76639219|ref|XP_593308.2| PREDICTED: similar to Protein KIAA0152 precursor [Bos taurus]
sw16168	BGIBMGA010213	2.07915	1.25435	1.11155	1.21205	1.00E-41	gi|441214|dbj|BAA03374.1| intracellular coagulation inhibitor precursor [Tachypleus tridentatus]
sw07562	BGIBMGA004942	0.40825	0.70605	0.7259	1.20885	1.00E-43	gi|1490232|emb|CAA67981.1| mitochondrial ATP synthase [Drosophila melanogaster]
sw02854	BGIBMGA007006	0.0522	0.5198	0.54485	1.2077	No hits found	
sw19774	BGIBMGA009646	0.33265	0.61405	0.73625	1.2015	No hits found	
sw12433	BGIBMGA008779	1.16325	2.61945	1.5224	1.2	2.00E-80	gi|50510635|dbj|BAD32303.1| mKIAA0788 protein [Mus musculus]
sw13529	BGIBMGA002669	1.23735	1.68715	2.2502	1.1997	e-120	gi|72172203|gb|AAZ66799.1| lipase [Samia cynthia ricini] lipase-1 [Bombyx mori] lipase [Bombyx mori]
sw22843	BGIBMGA014477	0.1448	0.473	0.3255	1.19745	6.00E-63	gi|32718005|gb|AAP86970.1| testicular microtubules-related protein 3 [Mus musculus] novel protein [Mus musculus] tektin 3 [Mus musculus] Tektin-3
sw01592	BGIBMGA005069	0.1816	0.64625	0.3886	1.1962	No hits found	
sw10457	BGIBMGA000700	0.1062	0.5622	0.5547	1.1916	No hits found	
sw04120	BGIBMGA013611	1.37525	1.4308	2.1573	1.1906	7.00E-10	gi|18087833|ref|NP_542393.1| peroxisomal biogenesis factor 11 gamma [Homo sapiens] Peroxisomal biogenesis factor 11 gamma [Homo sapiens] peroxin Pex11p gamma
sw03893	BGIBMGA010486	0.21955	0.6066	0.72475	1.1885	No hits found	
sw12994	BGIBMGA006747	0.35795	0.6103	1	1.18485	2.00E-21	gi|55235457|gb|EAA14923.2| ENSANGP00000010625 [Anopheles gambiae str. PEST] ENSANGP00000010625 [Anopheles gambiae str. PEST]
sw10535	BGIBMGA003665	0.4013	0.75605	0.77645	1.18405	2.00E-08	gi|55237378|gb|EAA12196.2| ENSANGP00000018278 [Anopheles gambiae str. PEST] ENSANGP00000018278 [Anopheles gambiae str. PEST]
sw11642	BGIBMGA006816	2.26595	2.0494	2.0041	1.17785	0	gi|3024509|sp|Q91437|PYR1_SQUAC CAD protein [Includes: Glutamine-dependent carbamoyl-phosphate synthase ; Aspartate carbamoyltransferase ; Dihydroorotase] multifunctional protein CAD
sw20515	BGIBMGA013783	0.0311	0.60135	0.5252	1.17535	1.00E-64	gi|66513601|ref|XP_623150.1| PREDICTED: similar to prophenoloxidase activating factor [Apis mellifera]
sw12992	BGIBMGA008810	1.9049	2.01105	1.622	1.17445	4.00E-13	gi|41618334|tpg|DAA03106.1| TPA: TPA_inf: HDC11369 [Drosophila melanogaster] CG33290-PA [Drosophila melanogaster] CG33290-PA [Drosophila melanogaster]
sw15899	BGIBMGA014307	0.09255	0.54925	0.5819	1.16765	No hits found	
sw17054	BGIBMGA000702	0.1157	0.56435	0.57215	1.16765	No hits found	
sw11328	BGIBMGA000465	0.1712	0.4817	1	1.16185	No hits found	
sw05302	BGIBMGA000336	1.95845	1.678	2.1477	1.15905	1.00E-21	gi|47605412|sp|Q7M4F2|CUD8_SCHGR Endocuticle structural glycoprotein SgAbd-8
sw22437	BGIBMGA001332	4.03835	2.46485	2.7747	1.15845	Bmb044299	2.00E-08
sw15726	BGIBMGA010895	0.16295	0.54885	0.5184	1.15665	No hits found	
sw19964	BGIBMGA004337	2.0586	1.40475	1.18085	1.1554	5.00E-06	gi|66507458|ref|XP_623142.1| PREDICTED: similar to ENSANGP00000016918 [Apis mellifera]
sw05251	BGIBMGA002976	0.03075	0.5112	0.6792	1.1518	2.00E-38	gi|8927389|gb|AAF82053.1| FOR I protein [Homo sapiens]
sw00199	BGIBMGA004540	0.313	0.70275	0.83285	1.1497	4.00E-98	gi|56378317|dbj|BAD74195.1| heat shock protein hsp 19.9 [Bombyx mori]

sw15544	BGIBMGA001099	0.0146	0.45185	0.41165	1.14825	No hits found	
sw09231	BGIBMGA001596	0.44045	0.6647	0.52425	1.14435	2.00E-08	gi|72023261|ref|XP_786653.1| PREDICTED: similar to Thioesterase superfamily member 2 (PNAS-27) (HT012) [Strongylocentrotus purpuratus]
sw00954	BGIBMGA009178	0.4428	0.5751	0.5327	1.1424	8.00E-31	gi|47117013|sp|Q7M4I6|PA2_MEGPE Phospholipase A2 (Phosphatidylcholine 2-acylhydrolase)
sw00971	BGIBMGA000755	0.1593	0.6338	0.60125	1.142	No hits found	
sw06143	BGIBMGA011342	2.03015	1.29205	1.48345	1.13975	2.00E-26	gi|2804262|dbj|BAA24442.1| cysteine proteinase [Sitophilus zeamais]
sw22125	BGIBMGA001620	0.43055	0.6882	0.58005	1.1396	2.00E-24	gi|70905642|gb|AAZ14281.1| proteophosphoglycan 5 [Leishmania major strain Friedlin] Leishmania major strain Friedlin proteophosphoglycan 5 [Leishmania major strain Friedlin]
sw08481	BGIBMGA001626	0.4682	0.7593	0.656	1.1365	No hits found	
sw03831	BGIBMGA008976	0.063	0.523	0.57735	1.1321	4.00E-41	gi|48097950|ref|XP_393932.1| PREDICTED: similar to molting fluid carboxypeptidase A [Apis mellifera]
sw13405	BGIBMGA005697	0.2918	0.9089	0.6814	1.1318	No hits found	
sw14786	BGIBMGA008167	2.74815	1.89655	1.7882	1.1283	6.00E-23	gi|58396460|ref|XP_321914.2| ENSANGP00000013861 [Anopheles gambiae str. PEST] ENSANGP00000013861 [Anopheles gambiae str. PEST]
sw21382	BGIBMGA005822	0.2016	0.6548	0.68865	1.12525	No hits found	
sw03565	BGIBMGA013574	0.38715	0.6879	0.87	1.12505	No hits found	
sw22881	BGIBMGA002604	0.13695	0.6485	0.60905	1.12435	3.00E-45	gi|66508910|ref|XP_623146.1| PREDICTED: similar to GA10372-PA [Apis mellifera]
sw12376	BGIBMGA000610	0.1414	0.6342	0.1196	1.12305	2.00E-44	gi|55241598|gb|EAA08488.2| ENSANGP00000014719 [Anopheles gambiae str. PEST] ENSANGP00000014719 [Anopheles gambiae str. PEST]
sw02067	BGIBMGA007216	0.2359	0.8122	0.48385	1.11865	2.00E-47	gi|48096263|ref|XP_394650.1| PREDICTED: similar to ENSANGP00000012511 [Apis mellifera]
sw02018	BGIBMGA001308	0.3424	1.5086	0.3915	1.11665	Bmb031686	4.00E-24
sw06049	BGIBMGA005403	0.14715	0.57695	0.63155	1.11635	5.00E-36	gi|387422|gb|AAA39509.1| malate dehydrogenase
sw16470	BGIBMGA012040	0.17645	0.49145	0.5204	1.115	1.00E-07	gi|73997460|ref|XP_853137.1| PREDICTED: similar to myosin regulatory light chain-like [Canis familiaris]
sw21899	BGIBMGA006623	2.30675	1.29985	0.79105	1.1135	e-112	gi|3510719|gb|AAC33576.1| immunolectin-A precursor [Manduca sexta]
sw08555	BGIBMGA000993	0.30845	0.6848	0.67395	1.1107	No hits found	
sw19720	BGIBMGA010834	0.13675	0.4788	0.7155	1.10995	No hits found	
sw12672	BGIBMGA003645	4.66495	1.6954	2.0591	1.1087	6.00E-62	gi|50730416|ref|XP_416893.1| PREDICTED: similar to lipoyltransferase 1 [Gallus gallus]
sw18729	BGIBMGA000731	3.2003	1.2652	0.97225	1.10685	9.00E-37	gi|74229818|ref|YP_309022.1| ecdysone glucose transferase (egt) [Trichoplusia ni SNPV] ecdysone glucose transferase (egt) [Trichoplusia ni SNPV]
sw14331	BGIBMGA007286	0.4465	0.64975	0.52605	1.10465	7.00E-61	gi|400673|sp|P31420|OMBP_MANSE Ommochrome-binding protein precursor (OBP) (YCP) ommochrome-binding protein
sw09897	BGIBMGA005795	0.18395	0.6919	0.7158	1.1045	No hits found	
sw06150	BGIBMGA011959	0.3919	0.9204	1	1.0985	No hits found	
sw18703	BGIBMGA010812	2.6714	2.10235	2.6688	1.09535	Bmb025436	e-169
sw12286	BGIBMGA003199	0.1101	0.64555	0.7385	1.0947	7.00E-54	gi|76632826|ref|XP_879048.1| PREDICTED: similar to polyamine oxidase isoform 1 isoform 11 [Bos taurus]

sw09814	BGIBMGA001021	0.04765	0.54655	0.4177	1.09235	No hits found	
sw16275	BGIBMGA009685	1.1433	1.42515	2.2439	1.08795	Bmb039916	No hits found
sw03059	BGIBMGA007637	0.46535	1.15335	1.0144	1.08745	0	gi|87248659|gb|ABD36382.1| glycyl-tRNA synthetase [Bombyx mori]
sw08664	BGIBMGA005704	0.4614	0.9525	0.9518	1.08545	No hits found	
sw15953	BGIBMGA014418	0.42565	0.93915	0.7447	1.0791	7.00E-22	gi|54642185|gb|EAL30934.1| GA14782-PA [Drosophila pseudoobscura]
sw22963	BGIBMGA001859	0.2281	0.7038	0.49695	1.07775	No hits found	
sw00035	BGIBMGA011472	0.43965	0.75515	0.66315	1.0756	No hits found	
sw13273	BGIBMGA007008	0.41435	0.7479	1	1.0635	No hits found	
sw04295	BGIBMGA003296	0.12305	0.2817	0.3204	1.0625	e-162	gi|402174|emb|CAA52906.1| beta-tubulin [Bombyx mori] Tubulin beta chain (Beta tubulin)
sw13389	BGIBMGA007398	0.21945	0.7087	0.7499	1.0623	No hits found	
sw17922	BGIBMGA001013	0.15385	0.50965	0.6292	1.05975	Bmb002484	1.00E-46
sw20612	BGIBMGA006165	0.10035	0.54025	0.61415	1.05025	No hits found	
sw01378	BGIBMGA012166	0.4526	0.7311	0.7529	1.0418	1.00E-22	gi|66525741|ref|XP_397234.2| PREDICTED: similar to WD repeat domain 34 [Apis mellifera]
sw08729	BGIBMGA000655	0.2195	0.60065	0.62075	1.0383	3.00E-05	gi|89301406|gb|EAR99394.1| hypothetical protein TTHERM_00133600 [Tetrahymena thermophila SB210]
sw17607	BGIBMGA005168	0.3294	0.5308	0.43475	1.03555	0	gi|13446608|emb|CAC35050.1| putative helicase Ski2 [Drosophila melanogaster]
sw19416	BGIBMGA007811	0.02825	0.45855	0.52315	1.02945	5.00E-18	gi|1226168|gb|AAA92249.1| ORF B (bases 1850-5560) first start codon at 2306
sw03486	BGIBMGA010818	0.0833	0.6134	0.60375	1.0279	No hits found	
sw12583	BGIBMGA011053	0.14935	0.7266	0.5551	1.02505	No hits found	
sw18898	BGIBMGA004541	0.4982	1.2154	0.3939	1.0237	2.00E-94	gi|11120618|gb|AAG30944.1| heat shock protein hsp20.8 [Bombyx mori] heat shock protein hsp20.8A [Bombyx mori]
sw09596	BGIBMGA010902	0.12315	0.45745	1	1.0222	No hits found	
sw14912	BGIBMGA004041	0.4351	0.91845	0.5957	1.01755	4.00E-29	gi|77415578|emb|CAJ01458.1| hypothetical protein [Bombyx mori]
sw20682	BGIBMGA004525	0.2889	0.82155	0.80395	1.0155	Bmb043387	1.00E-18
sw11415	BGIBMGA006854	0.68855	0.3664	0.24375	1.01535	No hits found	
sw18441	BGIBMGA004618	0.29725	0.78015	0.8078	1.0022	0	gi|72065171|ref|XP_791790.1| PREDICTED: similar to tubulin, beta, 2 [Strongylocentrotus purpuratus]
sw00748	BGIBMGA004906	2.6885	1	1	1	2.00E-61	gi|66508985|ref|XP_624902.1| PREDICTED: similar to ENSANGP00000010432 [Apis mellifera]
sw01458	BGIBMGA011005	2.0268	1	1	1	4.00E-15	gi|54639187|gb|EAL28589.1| GA18365-PA [Drosophila pseudoobscura]
sw03148	BGIBMGA006267	0.45615	1	0.5308	1	3.00E-54	gi|66499116|ref|XP_395333.2| PREDICTED: similar to Odorant receptor 83b, partial [Apis mellifera]
sw03415	BGIBMGA007255	2.94585	1	1	1	1.00E-61	gi|66516898|ref|XP_391826.2| PREDICTED: similar to yellow [Apis mellifera]

sw04223	BGIBMGA010082	0.134	0.54445	0.57525	1	No hits found	
sw05553	BGIBMGA000688	6.962	1	1	1	6.00E-51	gi|29788985|ref|NP_705933.1| solute carrier family 24 member 4 isoform 2 precursor [Homo sapiens] K-dependent Na/Ca exchanger NCKX4 [Homo sapiens]
sw06614	BGIBMGA007238	3.1439	1	1	1	e-131	gi|3158392|gb|AAC39036.1| MAP kinase kinase 4 [Drosophila melanogaster]
sw07086	BGIBMGA008333	2.6456	1	1	1	2.00E-35	gi|55237990|gb|EAA43983.2| ENSANGP00000023009 [Anopheles gambiae str. PEST] ENSANGP00000023009 [Anopheles gambiae str. PEST]
sw08448	BGIBMGA010063	4.36115	2.0319	3.1085	1	6.00E-38	gi|6690636|gb|AAF24228.1| trypsin-like PiT2c precursor [Plodia interpunctella]
sw08470	BGIBMGA005215	0.1696	1	0.71475	1	6.00E-52	gi|75516537|gb|AAI01520.1| Testis-specific gene A2 [Homo sapiens] testis-specific gene A2 [Homo sapiens] Testis-specific gene A2 protein (Male meiotic metaphase chromosome-associated acidic protein)
sw08901	BGIBMGA009827	1	2.2925	1	1	9.00E-25	gi|66531593|ref|XP_624704.1| PREDICTED: similar to GA21651-PA [Apis mellifera]
sw09295	BGIBMGA014054	1.32415	2.23735	0.73585	1	3.00E-17	gi|24644778|ref|NP_649706.1| CG14606-PA [Drosophila melanogaster] IP11886p [Drosophila melanogaster] CG14606-PA [Drosophila melanogaster]
sw09459	BGIBMGA011041	1.68865	2.0717	1	1	Bmb030337	No hits found
sw09515	BGIBMGA008985	0.4023	0.7586	0.80445	1	2.00E-08	gi|74145314|gb|AAZ99885.1| membrance occupation and recognition nexus protein 1 [Toxoplasma gondii]
sw10934	BGIBMGA013914	2.4836	1.82055	1	1	Bmb002530	4.00E-08
sw11020	BGIBMGA007654	0.08735	0.57135	0.6188	1	1.00E-09	gi|73995649|ref|XP_534748.2| PREDICTED: similar to Rhabdoid tumor deletion region protein 1 isoform 1 [Canis familiaris]
sw11494	BGIBMGA003053	1.4334	1.32725	2.0434	1	1.00E-52	gi|40674148|gb|AAH64831.1| HMT1 hnRNP methyltransferase-like 3 [Homo sapiens] HMT1 hnRNP methyltransferase-like 3 [Homo sapiens] HMT1 hnRNP methyltransferase-like 3
sw12194	BGIBMGA013504	1	0.4328	1	1	3.00E-11	gi|66524513|ref|XP_624132.1| PREDICTED: similar to MGC80265 protein [Apis mellifera]
sw12614	BGIBMGA007379	1	4.80935	3.816	1	3.00E-05	gi|90111982|sp|Q9H9A7|CI076_HUMAN Protein C9orf76
sw13012	BGIBMGA007058	0.08045	0.4751	1	1	e-162	gi|76617111|ref|XP_874104.1| PREDICTED: similar to tubulin tyrosine ligase-like 1 (predicted) isoform 2 [Bos taurus]
sw13094	BGIBMGA008101	1	1	0.34805	1	5.00E-10	gi|58533151|gb|AAW78933.1| trypsin [Anopheles sinensis]
sw13391	BGIBMGA004067	0.8717	1.17465	0.4881	1	1.00E-10	gi|77415706|emb|CAJ01522.1| hypothetical protein [Anopheles gambiae] putative sensory appendage protein SAP-2 [Anopheles gambiae] ENSANGP00000011748
sw13872	BGIBMGA009422	0.098	0.56255	0.67095	1	3.00E-16	gi|15218966|ref|NP_173553.1| ATEXT3 (EXTENSIN 3); structural constituent of cell wall
sw14075	BGIBMGA001231	2.7854	1.7448	1.32485	1	2.00E-25	gi|54644247|gb|EAL32988.1| GA17385-PA [Drosophila pseudoobscura]
sw14227	BGIBMGA005237	0.38035	0.64615	1	1	4.00E-05	gi|66547599|ref|XP_623499.1| PREDICTED: similar to Beat VII [Apis mellifera]
sw14502	BGIBMGA008031	0.10385	0.778	0.5543	1	7.00E-06	gi|53133880|emb|CAG32269.1| hypothetical protein [Gallus gallus] hypothetical protein LOC416598 [Gallus gallus]
sw14865	BGIBMGA005191	0.3363	0.6735	1	1	2.00E-06	gi|10444078|gb|AAG17699.1| mitochondrial malate dehydrogenase precursor [Nucella lapillus]
sw15568	BGIBMGA001156	0.43165	0.79945	0.72515	1	1.00E-49	gi|72025672|ref|XP_792004.1| PREDICTED: similar to mitochondrial malate dehydrogenase precursor [Strongylocentrotus purpuratus]
sw15581	BGIBMGA004598	0.13535	0.6073	1	1	9.00E-24	gi|9294518|dbj|BAB02780.1| dual-specificity protein phosphatase-like protein [Arabidopsis thaliana]
sw15966	BGIBMGA001757	0.0853	0.44275	0.5094	1	No hits found	
sw16060	BGIBMGA006472	0.09615	0.599	1	1	No hits found	
sw16556	BGIBMGA003746	0.318	0.6545	0.7532	1	No hits found	

sw16752	BGIBMGA012871	1	1.67115	2.5879	1	Bmb046498	5.00E-05	
sw19095	BGIBMGA014144	2.3389	1	1	1	4.00E-28	gi|20334294|dbj|BAB91145.1| beta-glucosidase [Neotermes koshunensis]	
sw20799	BGIBMGA014242	6.7311	2.5926	2.29675	1	Bmb006459	3.00E-93	
sw20883	BGIBMGA012265	2.34115	1.1885	0.92425	1	1.00E-12	gi|48474335|sp|Q60417|SCRB1_CRIGR Scavenger receptor class B member 1 (SRB1) (SR-BI) (HaSR-BI) haSR-BI	
sw22231	BGIBMGA004528	0.25075	1.2309	0.78955	1	2.00E-11	gi|79464734|ref|NP_192384.2| carbohydrate transporter/ sugar porter [Arabidopsis thaliana]	
sw22269	BGIBMGA001374	0.11	0.25565	0.21795	1	No hits found		
sw22271	BGIBMGA010680	0.44955	1.07535	0.468	1	2.00E-15	gi|9105718|gb|AAF83628.1| endo-1,4-beta-glucanase [Xylella fastidiosa 9a5c] endo-1,4-beta-glucanase [Xylella fastidiosa 9a5c]	
sw22288	BGIBMGA004708	2.7738	1	1	1	e-104	gi|87248427|gb|ABD36266.1| triacylglycerol lipase [Bombyx mori]	
sw22607	BGIBMGA010045	2.86025	1	1.78005	1	1.00E-16	gi|4239700|emb|CAA10770.1| reverse transcriptase-like [Bos taurus]	
sw22695	BGIBMGA003395	0.2266	0.65665	0.4827	1	1.00E-26	gi|55666027|emb|CAH74051.1| OTTHUMP00000065631 [Homo sapiens] OTTHUMP00000065631 [Homo sapiens] OTTHUMP00000065631	
sw22716	BGIBMGA010116	0.4707	0.80735	0.6005	1	No hits found		
sw00011	BGIBMGA009446	0.4385	0.694	0.7712	0.99595	No hits found		
sw11825	BGIBMGA003374	1.01005	2.24665	1.32105	0.98645	e-136	gi|27374348|gb|AAO01089.1| CG4733-PA [Drosophila willistoni]	
sw22171	BGIBMGA000423	0.2832	0.37345	0.32785	0.9827	No hits found		
sw09159	BGIBMGA008119	0.4277	0.61415	0.64435	0.98035	Bmb025309	7.00E-05	
sw08186	BGIBMGA009324	0.3687	0.7438	0.7524	0.98015	No hits found		
sw04095	BGIBMGA005598	1.5945	1.4924	2.095	0.97075	Bmb026464	2.00E-26	
sw13441	BGIBMGA000013	0.05605	0.8469	0.3692	0.9658	2.00E-10	gi|54638658|gb|EAL28060.1| GA13834-PA [Drosophila pseudoobscura]	
sw13920	BGIBMGA011965	0.494	0.83565	0.69615	0.9527	e-133	gi|66524404|ref|XP_623193.1| PREDICTED: similar to DEAD (Asp-Glu-Ala-Asp) box polypeptide 5, partial [Apis mellifera]	
sw13737	BGIBMGA011695	0.6495	0.65345	0.4226	0.94835	e-117	gi|1923274|gb|AAC47506.1| Tpr homolog [Drosophila melanogaster]	
sw04258	BGIBMGA011058	0.2539	0.7685	0.1437	0.9466	No hits found		
sw18399	BGIBMGA008824	2.293	4.27355	3.3477	0.9437	0	gi|102886|pir||S19607 alkaline phosphatase (EC 3.1.3.1) - silkworm	
sw13077	BGIBMGA007091	0.0998	0.6261	0.6535	0.9414	No hits found		
sw05455	BGIBMGA009467	1.9794	2.1042	1.91805	0.94125	1.00E-83	gi|73974968|ref|XP_858777.1| PREDICTED: similar to glucosamine-6-phosphate deaminase 2 isoform 3 [Canis familiaris]	
sw13753	BGIBMGA012101	0.6943	0.67175	0.37235	0.94	3.00E-26	gi|24649778|ref|NP_651285.1| CG13634-PA [Drosophila melanogaster] CG13634-PA [Drosophila melanogaster]	
sw20476	BGIBMGA007307	0.1575	0.64985	0.6155	0.91955	2.00E-38	gi|1490236|emb|CAA67980.1| oligomycin sensitivity conferring protein precursor [Drosophila melanogaster]	
sw15838	BGIBMGA001609	2.216	1.5924	1.59785	0.91905	e-152	gi|25012519|gb|AAN71363.1| RE31673p [Drosophila melanogaster]	
sw22856	BGIBMGA013408	0.07955	0.5735	0.63685	0.91525	No hits found		

sw09712	BGIBMGA010403	0.41895	0.53645	0.56755	0.91375	Bmb034569	e-116
sw18137	BGIBMGA007251	0.0844	0.5428	0.55365	0.91025	No hits found	
sw08476	BGIBMGA009245	0.2864	0.5403	0.41425	0.90475	6.00E-58	gi|85861035|gb|ABC86467.1| IP04174p [Drosophila melanogaster]
sw03028	BGIBMGA000089	0.39085	0.72265	0.62165	0.9037	2.00E-11	gi|19572382|emb|CAD27928.1| putative G-protein coupled receptor [Anopheles gambiae] ENSANGP00000018168 [Anopheles gambiae str. PEST] ENSANGP00000018168 [Anopheles gambiae str. PEST]
sw17363	BGIBMGA013260	0.3358	0.6193	0.3097	0.9037	2.00E-49	gi|55235212|gb|EAA14862.2| ENSANGP00000013993 [Anopheles gambiae str. PEST] ENSANGP00000013993 [Anopheles gambiae str. PEST]
sw12770	BGIBMGA009753	0.3557	0.58725	0.73245	0.90195	No hits found	
sw07921	BGIBMGA009061	0.0811	0.59375	0.5822	0.9012	No hits found	
sw15465	BGIBMGA003452	0.03905	0.63075	0.74495	0.89685	1.00E-12	gi|55244897|gb|EAA05228.3| ENSANGP00000012660 [Anopheles gambiae str. PEST] ENSANGP00000012660 [Anopheles gambiae str. PEST]
sw00599	BGIBMGA002774	0.1069	0.6094	0.4788	0.89665	1.00E-26	gi|56270199|gb|AAH87372.1| LOC495986 protein [Xenopus laevis]
sw03447	BGIBMGA013571	0.3203	0.6755	0.79085	0.8961	No hits found	
sw01620	BGIBMGA007228	2.23205	1.8417	1.6814	0.89375	Bmb025093	e-127
sw22932	BGIBMGA010889	0.76525	0.4989	0.68455	0.8937	3.00E-16	gi|72077548|ref|XP_796448.1| PREDICTED: similar to riddle like (86.6 kD) (5O189) [Strongylocentrotus purpuratus]
sw17796	BGIBMGA008829	0.0283	0.67575	0.2981	0.89065	6.00E-08	gi|17942678|pdb|1K9O|I Chain I, Crystal Structure Of Michaelis Serpin-Trypsin Complex
sw18048	BGIBMGA011843	0.07905	0.80435	1	0.8898	No hits found	
sw10040	BGIBMGA010807	0.0757	0.6579	0.6793	0.88965	No hits found	
sw13243	BGIBMGA006116	0.0718	0.63175	0.63205	0.88725	1.00E-13	gi|50751424|ref|XP_422391.1| PREDICTED: similar to adenylate kinase 5 isoform 1; adenylate kinase 6; ATP-AMP transphosphorylase [Gallus gallus]
sw18815	BGIBMGA001348	1.38275	0.473	0.57655	0.8861	1.00E-62	gi|50764028|ref|XP_422928.1| PREDICTED: similar to aldose reductase [Gallus gallus]
sw05653	BGIBMGA002038	0.04405	0.5796	0.63285	0.88555	1.00E-28	gi|24581791|ref|NP_608886.1| CG14043-PA [Drosophila melanogaster] CG14043-PA [Drosophila melanogaster]
sw13517	BGIBMGA008859	1	2.9164	1	0.88535	e-162	gi|2625150|gb|AAB86646.1| moderately methionine rich hexamerin precursor; MMtH [Hyalophora cecropia]
sw11316	BGIBMGA007850	0.0612	0.4622	0.43535	0.87355	No hits found	
sw15875	BGIBMGA006235	0.4254	0.85295	0.61605	0.87245	Bmb036048	5.00E-18
sw00170	BGIBMGA004943	0.1182	0.58105	0.66255	0.87225	5.00E-68	gi|73995987|ref|XP_850513.1| PREDICTED: similar to serine/threonine kinase 22B (spermiogenesis associated) (predicted) [Canis familiaris]
sw06190	BGIBMGA001122	0.2441	1	1	0.87175	e-107	gi|87248307|gb|ABD36206.1| peptidyl-prolyl cis-trans isomerase E [Bombyx mori]
sw11883	BGIBMGA002125	1.45315	2.02865	1.41775	0.8668	3.00E-18	gi|27374312|gb|AAO01060.1| ap-PA [Drosophila willistoni]
sw03301	BGIBMGA013242	2.9008	1.18495	1	0.86435	6.00E-05	gi|76791840|ref|ZP_00774344.1| General substrate transporter [Pseudoalteromonas atlantica T6c] General substrate transporter [Pseudoalteromonas atlantica T6c]
sw19465	BGIBMGA013329	0.3989	0.57155	0.5711	0.86405	7.00E-08	gi|72115693|ref|XP_782070.1| PREDICTED: similar to predicted CDS, reverse transcriptase family member (XB968) [Strongylocentrotus purpuratus]
sw09114	BGIBMGA010771	0.2106	1	0.73535	0.86395	No hits found	
sw00678	BGIBMGA006703	0.2436	0.6517	0.76625	0.85335	e-100	gi|68584|pir||AJFF1M glutamate-ammonia ligase (EC 6.3.1.2) 1, mitochondrial - fruit fly (Drosophila melanogaster)

sw08067	BGIBMGA004990	0.27105	0.68825	0.76495	0.8504	4.00E-27	gi|72130450|ref|XP_795609.1| PREDICTED: similar to TNF receptor-associated factor 3 interacting protein 1 (predicted), partial [Strongylocentrotus purpuratus]
sw13013	BGIBMGA005749	2.6142	2.5225	1	0.8455	2.00E-27	gi|10442636|gb|AAG17398.1| gamma-syntrophin-like protein SYN2 [Drosophila melanogaster]
sw16249	BGIBMGA012879	0.1498	0.66365	0.68655	0.8449	Bmb039561	1.00E-43
sw09076	BGIBMGA003577	0.09965	0.6134	0.63625	0.842	No hits found	
sw02176	BGIBMGA001201	0.6989	0.4896	0.7828	0.84025	3.00E-72	gi|90567700|emb|CAI30053.1| muscular protein 20 [Hydroporus erythrocephalus]
sw18850	BGIBMGA011059	0.1465	0.634	0.35765	0.8396	2.00E-46	gi|72067669|ref|XP_798203.1| PREDICTED: similar to reverse transcriptase family member (1F383) [Strongylocentrotus purpuratus]
sw03198	BGIBMGA011432	0.75545	0.8793	0.2894	0.83715	3.00E-05	gi|57972112|ref|XP_564944.1| ENSANGP00000027953 [Anopheles gambiae str. PEST] ENSANGP00000027953 [Anopheles gambiae str. PEST]
sw15814	BGIBMGA006125	0.42825	0.8472	0.7054	0.83455	Bmb035645	No hits found
sw03247	BGIBMGA013360	0.08545	0.60305	0.6612	0.8337	No hits found	
sw06863	BGIBMGA004028	0.19535	0.57295	0.58195	0.8315	3.00E-83	gi|72012777|ref|XP_780667.1| PREDICTED: similar to testis-specific serine kinase 4 [Strongylocentrotus purpuratus]
sw03217	BGIBMGA005787	3.3007	3.0253	1.67145	0.8298	2.00E-87	gi|66503826|ref|XP_392190.2| PREDICTED: similar to RH44796p [Apis mellifera]
sw09306	BGIBMGA010127	0.23905	0.5477	0.4526	0.82615	No hits found	
sw08418	BGIBMGA006385	0.4462	0.6361	0.64385	0.82525	3.00E-35	gi|86515380|ref|NP_001034519.1| abdominal-B [Tribolium castaneum] abdominal-B [Tribolium castaneum]
sw04137	BGIBMGA012680	1.921	1.64625	2.82885	0.8215	2.00E-34	gi|66517541|ref|XP_393694.2| PREDICTED: similar to CG32171-PD, isoform D [Apis mellifera]
sw03311	BGIBMGA008828	0.08985	0.66275	0.32335	0.8171	1.00E-07	gi|217291|dbj|BAA00639.1| antitrypsin precursor [Bombyx mori] Antitrypsin precursor (AT)
sw03590	BGIBMGA013868	0.49725	0.7061	0.53775	0.81525	1.00E-52	gi|66506650|ref|XP_625203.1| PREDICTED: similar to Dscam [Apis mellifera]
sw10691	BGIBMGA004051	0.11	0.5677	0.57975	0.8113	1.00E-09	gi|26454107|dbj|BAC44437.1| DNA topoisomerase IV subunit A [Mycoplasma penetrans HF-2] DNA topoisomerase IV subunit A [Mycoplasma penetrans HF-2]
sw16450	BGIBMGA012894	0.6217	0.87235	0.45415	0.81065	No hits found	
sw03575	BGIBMGA010438	0.4397	0.57415	0.31915	0.8051	2.00E-10	gi|68076941|ref|XP_680390.1| MAEBL [Plasmodium berghei strain ANKA] MAEBL, putative [Plasmodium berghei]
sw19086	BGIBMGA014542	0.0775	0.4695	0.7655	0.80205	1.00E-04	gi|77387268|gb|ABA78453.1| F1-ATP synthase beta subunit [Rhodobacter sphaeroides 2.4.1] ATP synthase subunit B
sw03882	BGIBMGA004398	0.4284	0.8142	0.6299	0.80055	8.00E-33	gi|126417|sp|P09336|LP3_BOMMO Low molecular 30 kDa lipoprotein PBMHPC-19 precursor 30K lipoprotein precursor [Bombyx mori]
sw00440	BGIBMGA005786	2.688	1.80735	1.4433	0.79945	5.00E-06	gi|76617327|ref|XP_585387.2| PREDICTED: similar to Inositol oxygenase (Myo-inositol oxygenase) (Aldehyde reductase-like 6) (Renal-specific oxidoreductase) (Kidney-specific protein 32) [Bos taurus]
sw16133	BGIBMGA013229	0.35435	0.8094	0.6852	0.79595	2.00E-06	gi|74096211|ref|NP_001027646.1| axonemal p66.0 [Ciona intestinalis] axonemal p66.0 [Ciona intestinalis]
sw02395	BGIBMGA010038	2.11825	1.4194	1.27615	0.79475	2.00E-28	gi|60393084|gb|AAX19492.1| slalom PAPS transporter [Lucilia sericata]
sw19831	BGIBMGA007986	0.48395	0.6244	0.60705	0.7911	3.00E-07	gi|37589499|gb|AAH59895.1| Pik4cb protein [Mus musculus]
sw15978	BGIBMGA010039	0.1037	0.4127	0.41545	0.78995	2.00E-05	gi|53148471|dbj|BAD52263.1| antennal binding protein [Plutella xylostella] sericotropin-like protein [Plutella xylostella]
sw04440	BGIBMGA006954	0.3074	0.50385	0.37915	0.7835	8.00E-06	gi|54636037|gb|EAL25440.1| GA10629-PA [Drosophila pseudoobscura]
sw12530	BGIBMGA008358	0.4194	0.5254	0.53745	0.7831	Bmb012777	7.00E-54

sw02266	BGIBMGA013770	0.35215	0.634	0.45255	0.78045	No hits found	
sw08839	BGIBMGA001797	0.47465	0.9065	0.71755	0.7802	2.00E-81	gi|56377671|dbj|BAD74067.1| chromosome 2 open reading frame 4 short form [Homo sapiens]
sw03818	BGIBMGA003662	0.17025	0.6453	0.7711	0.7733	2.00E-34	gi|72096309|ref|XP_797414.1| PREDICTED: similar to t-complex-associated testis expressed 1 [Strongylocentrotus purpuratus]
sw06412	BGIBMGA003032	0.4515	0.8195	0.7819	0.7704	e-104	gi|66519838|ref|XP_391911.2| PREDICTED: similar to GA10180-PA [Apis mellifera] PREDICTED: similar to GA10180-PA [Apis mellifera]
sw20316	BGIBMGA013237	2.0269	0.9817	1.6017	0.76835	2.00E-68	gi|81248546|gb|ABB69054.1| cytochrome P450 [Helicoverpa armigera]
sw14661	BGIBMGA010104	0.4134	0.61355	0.4949	0.76295	1.00E-32	gi|54648000|gb|AAH84947.1| LOC495430 protein [Xenopus laevis]
sw06770	BGIBMGA012737	1	1	0.35995	0.7628	Bmb034180	No hits found
sw17451	BGIBMGA011145	0.36095	0.7673	0.5179	0.76195	Bmb028728	1.00E-57
sw07866	BGIBMGA013235	0.4833	1	0.8409	0.76015	Bmb006539	3.00E-89
sw05679	BGIBMGA002689	0.1978	0.76385	0.34035	0.7599	No hits found	
sw12826	BGIBMGA003277	0.3082	0.675	0.5694	0.757	No hits found	
sw01327	BGIBMGA002780	0.42065	0.66845	0.5433	0.75615	3.00E-79	gi|68367804|ref|XP_695826.1| PREDICTED: similar to tripartite motif-containing 37 protein [Danio rerio]
sw00752	BGIBMGA006087	0.37415	1	1	0.75405	e-125	gi|2494216|sp|Q16960|DYI3_ANTCR Dynein intermediate chain 3, ciliary dynein intermediate chain 3 [Anthocidaris crassispina]
sw15315	BGIBMGA004826	0.44055	0.6637	0.98125	0.75375	8.00E-29	gi|72015103|ref|XP_781418.1| PREDICTED: similar to DNA polymerase epsilon, catalytic subunit A (DNA polymerase II subunit A) [Strongylocentrotus purpuratus]
sw15945	BGIBMGA004630	1.0991	1.4282	0.2501	0.7535	8.00E-57	gi|56378319|dbj|BAD74196.1| heat shock protein hsp20.1 [Bombyx mori]
sw14737	BGIBMGA006103	0.378	0.7459	0.5702	0.74705	8.00E-05	gi|68390118|ref|XP_694565.1| PREDICTED: similar to autoantigen RCD8 [Danio rerio]
sw00728	BGIBMGA012264	2.19	1.3607	1.3008	0.74565	5.00E-21	gi|27261765|gb|AAN86085.1| lysozyme [Penaeus semisulcatus]
sw03453	BGIBMGA005122	0.19445	0.5382	0.6389	0.74545	Bmb011340	No hits found
sw05367	BGIBMGA005623	0.1184	0.47105	0.55015	0.74425	No hits found	
sw04945	BGIBMGA009530	0.0442	0.50805	0.5175	0.74375	1.00E-37	gi|13365633|dbj|BAB39164.1| testis-specific ATPase inhibitor-like protein [Bombyx mori]
sw05534	BGIBMGA013115	1.8705	2.32265	2.10675	0.74345	4.00E-57	gi|13591614|dbj|BAB40959.1| prophenoloxidase-2s [Bombyx mori]
sw12357	BGIBMGA000895	0.0244	0.63975	0.68205	0.74275	2.00E-13	gi|14423760|sp|P93203|MFP1_LYCES MAR binding filament-like protein 1 MFP1 protein [Lycopersicon esculentum]
sw15671	BGIBMGA007787	0.3562	0.3586	0.3632	0.7351	0	gi|29420459|dbj|BAC66474.1| anceropsin [Antheraea pernyi]
sw09635	BGIBMGA012427	2.68015	3.387	2.53755	0.7349	Bmb033167	6.00E-55
sw16538	BGIBMGA004726	1	1.47415	0.47005	0.73375	Bmb042912	2.00E-28
sw06658	BGIBMGA002175	1.0037	0.3754	0.3937	0.73095	Bmb031781	2.00E-18
sw22862	BGIBMGA014231	0.0954	0.5196	0.6223	0.72205	No hits found	
sw13232	BGIBMGA010449	2.0431	1.1627	1	0.71775	e-151	gi|66506401|ref|XP_624756.1| PREDICTED: similar to CG11490-PA [Apis mellifera]

sw09626	BGIBMGA002379	0.4207	0.4669	0.58915	0.7168	2.00E-64	gi|72679333|gb|AAI00144.1| Dctn4 protein [Rattus norvegicus] p62 dynactin subunit [Rattus norvegicus]
sw13340	BGIBMGA009206	0.0898	0.68505	0.73985	0.71465	Bmb017778	3.00E-41
sw13090	BGIBMGA003269	0.46345	0.723	0.5443	0.71415	1.00E-06	gi|62739281|gb|AAH94228.1| Leucine-rich repeats and immunoglobulin-like domains 2 [Mus musculus] leucine-rich repeats and immunoglobulin-like domains 2
sw03241	BGIBMGA003865	5.5432	2.92495	1.80485	0.71	6.00E-36	gi|34366066|emb|CAE18120.1| glycine cleavage system protein H [Crassostrea gigas]
sw15658	BGIBMGA006290	0.0924	0.5991	0.66855	0.70775	Bmb034448	6.00E-67
sw13680	BGIBMGA013857	0.102	0.63605	0.5946	0.70765	No hits found	
sw12461	BGIBMGA000734	0.17305	0.5658	0.5333	0.70285	No hits found	
sw11741	BGIBMGA005330	0.3064	1	0.74855	0.70165	4.00E-13	gi|85857620|gb|ABC86345.1| IP13967p [Drosophila melanogaster]
sw10497	BGIBMGA005698	0.183	0.57625	0.6758	0.69485	No hits found	
sw15894	BGIBMGA011224	0.3714	0.7871	1	0.69035	5.00E-25	gi|55239363|gb|EAL40125.1| ENSANGP00000029489 [Anopheles gambiae str. PEST] ENSANGP00000029489 [Anopheles gambiae str. PEST]
sw22218	BGIBMGA004384	0.27695	0.6209	0.77425	0.68775	No hits found	
sw17493	BGIBMGA009393	0.46435	0.91245	0.72025	0.6802	Bmb037261	e-121
sw09048	BGIBMGA009927	1.44505	1.45305	2.09225	0.67945	1.00E-50	gi|68393171|ref|XP_688756.1| PREDICTED: similar to Calcitonin gene-related peptide type 1 receptor precursor (CGRP type 1 receptor) (Calcitonin receptor-like receptor) [Danio rerio]
sw00322	BGIBMGA004209	0.2499	0.5961	0.42265	0.67705	Bmb004470	2.00E-10
sw11700	BGIBMGA004190	0.9739	2.14125	2.39275	0.6745	1.00E-10	gi|66536215|ref|XP_624664.1| PREDICTED: similar to GA15552-PA [Apis mellifera]
sw04327	BGIBMGA007804	0.4216	0.69275	0.57505	0.66945	Bmb031754	e-131
sw06775	BGIBMGA000314	0.34175	0.7494	0.74205	0.665	No hits found	
sw13482	BGIBMGA000563	2.65235	2.2217	1.0097	0.66365	Bmb018665	2.00E-95
sw12823	BGIBMGA010944	2.1599	1.2749	1.1063	0.66325	5.00E-07	gi|28569894|dbj|BAC57926.1| reverse transcriptase [Bombyx mori]
sw17671	BGIBMGA002056	0.1666	0.64005	0.7073	0.66175	2.00E-12	gi|60551660|gb|AAH91500.1| ODF2 protein [Homo sapiens]
sw17454	BGIBMGA007622	0.4325	0.6906	0.59405	0.6565	1.00E-11	gi|72005907|ref|XP_781514.1| PREDICTED: similar to Nuclear pore complex protein Nup205 (Nucleoporin Nup205) (205 kDa nucleoporin) [Strongylocentrotus purpuratus]
sw15602	BGIBMGA014224	0.39895	0.7933	0.39165	0.6548	Bmb033954	2.00E-67
sw21169	BGIBMGA009782	0.1506	0.65105	0.73255	0.65335	1.00E-78	gi|474339|gb|AAA17752.1| reverse transcriptase [Bombyx mori]
sw18977	BGIBMGA011215	0.47375	0.68235	0.58855	0.6527	7.00E-19	gi|5911296|gb|AAD55740.1| BcDNA.LD27979 [Drosophila melanogaster]
sw10102	BGIBMGA007935	2.33715	2.156	1.82665	0.6523	Bmb043060	3.00E-14
sw03554	BGIBMGA006720	0.17395	0.65765	0.6171	0.646	2.00E-26	gi|76628450|ref|XP_606462.2| PREDICTED: similar to leucine rich repeat containing 45, partial [Bos taurus]
sw03396	BGIBMGA005653	0.4749	0.721	0.5734	0.64435	No hits found	
sw11929	BGIBMGA013306	0.4617	0.64245	0.56725	0.6423	No hits found	

sw05348	BGIBMGA006649	0.1481	1	1	0.6407	2.00E-27	gi|76628450|ref|XP_606462.2| PREDICTED: similar to leucine rich repeat containing 45, partial [Bos taurus]
sw18181	BGIBMGA012486	2.5286	2.0179	0.88725	0.64055	1.00E-59	gi|295756|emb|CAA33113.1| TU-36B protein [Drosophila melanogaster]
sw06613	BGIBMGA006213	2.2885	1.18715	1.744	0.6333	e-104	gi|28317017|gb|AAO39528.1| RE22242p [Drosophila melanogaster]
sw07419	BGIBMGA001368	0.2814	0.6726	0.70325	0.6304	No hits found	
sw13957	BGIBMGA012211	1	2.0871	1	0.6296	5.00E-24	gi|4158234|emb|CAA75614.1| MA3 [Suberites domuncula]
sw14437	BGIBMGA012586	0.3161	0.6954	0.5105	0.62755	Bmb025239	2.00E-47
sw03890	BGIBMGA014445	0.3841	0.6239	0.50195	0.62615	4.00E-13	gi|66514931|ref|XP_395624.2| PREDICTED: similar to broad complex isoform Z1 [Apis mellifera]
sw09008	BGIBMGA000224	0.4934	0.7554	1	0.621	4.00E-31	gi|72049850|ref|XP_787337.1| PREDICTED: similar to solute carrier family 2, (facilitated glucose transporter), member 8 [Strongylocentrotus purpuratus]
sw19541	BGIBMGA004402	0.1582	0.6129	0.3905	0.61895	6.00E-82	gi|10907|emb|CAA38531.1| 30K protein [Bombyx mori] Low molecular mass 30 kDa lipoprotein 21G1 precursor
sw13880	BGIBMGA014287	0.12105	0.56645	0.4446	0.61675	2.00E-06	gi|41407340|ref|NP_960176.1| PstA [Mycobacterium avium subsp. paratuberculosis K-10] PstA [Mycobacterium avium subsp. paratuberculosis K-10]
sw11356	BGIBMGA004681	0.0392	0.51035	0.5689	0.6145	2.00E-75	gi|62526540|gb|AAX84656.1| alpha-tubulin [Tyrophagus putrescentiae]
sw02940	BGIBMGA001213	0.13705	0.2046	0.13175	0.6062	No hits found	
sw11380	BGIBMGA005514	0.082	0.7278	0.88345	0.60455	No hits found	
sw16519	BGIBMGA004436	2.02545	1.3108	1.3945	0.60425	7.00E-16	gi|28316894|gb|AAO39469.1| RH04286p [Drosophila melanogaster]
sw03884	BGIBMGA013543	0.20895	0.50965	0.3297	0.6013	e-106	gi|48104652|ref|XP_395824.1| PREDICTED: similar to transcription factor IIB [Apis mellifera]
sw02475	BGIBMGA009791	0.41625	0.99555	0.80215	0.5964	Bmb041791	2.00E-22
sw20990	BGIBMGA011460	0.0266	0.09555	0.0586	0.59525	5.00E-13	gi|6560645|gb|AAF16700.1| juvenile hormone binding protein precursor-like protein [Manduca sexta]
sw19059	BGIBMGA014264	0.22095	0.6985	0.701	0.5904	1.00E-06	gi|89291452|gb|EAR89440.1| HMG box family protein [Tetrahymena thermophila SB210]
sw22464	BGIBMGA008361	0.08235	0.40145	0.6248	0.58875	3.00E-06	gi|74009366|ref|XP_849771.1| PREDICTED: similar to keratin associated protein 10-7 [Canis familiaris]
sw09993	BGIBMGA011628	0.29585	0.8751	0.5858	0.58695	Bmb039784	3.00E-12
sw12280	BGIBMGA008872	2.43545	2.3404	1.823	0.5816	4.00E-13	gi|72009524|ref|XP_785536.1| PREDICTED: similar to Sialin (Solute carrier family 17 member 5) (Sodium/sialic acid cotransporter) (AST) (Membrane glycoprotein HP59) [Strongylocentrotus purpuratus]
sw17422	BGIBMGA004400	0.3487	0.8991	0.51785	0.57735	e-139	gi|266438|sp|Q00802|L301_BOMMO Low molecular mass 30 kDa lipoprotein 19G1 precursor 30K protein [Bombyx mori]
sw19386	BGIBMGA012863	0.25035	0.71345	0.3407	0.57675	7.00E-63	gi|60099894|gb|AAX13081.1| glucose dehydrogenase [Drosophila miranda]
sw04565	BGIBMGA009874	0.47145	0.6308	0.69915	0.5756	Bmb038486	3.00E-50
sw08318	BGIBMGA014102	0.13375	0.5121	0.6607	0.573	1.00E-05	gi|57914149|ref|XP_554917.1| ENSANGP00000026385 [Anopheles gambiae str. PEST] ENSANGP00000026385 [Anopheles gambiae str. PEST]
sw06935	BGIBMGA009134	0.3764	0.68705	0.5667	0.5685	Bmb037859	7.00E-18
sw20743	BGIBMGA008668	2.05	1.4427	1.22315	0.56615	1.00E-83	gi|20372973|dbj|BAB91156.1| serine protease [Bombyx mori]
sw13801	BGIBMGA013753	0.22785	0.73475	0.5534	0.56395	4.00E-08	gi|48097752|ref|XP_393878.1| PREDICTED: similar to ENSANGP00000016890 [Apis mellifera]

sw19515	BGIBMGA003576	0.195	0.68785	0.80325	0.5639	8.00E-11	gi|83001004|ref|XP_150403.5| PREDICTED: similar to fibrous sheath interacting protein 2 [Mus musculus] PREDICTED: similar to fibrous sheath interacting protein 2 [Mus musculus]
sw02159	BGIBMGA009641	2.31815	1.3921	1.40145	0.5581	Bmb034419	6.00E-06
sw17599	BGIBMGA009093	0.3566	0.7186	0.5184	0.55215	5.00E-09	gi|1836157|gb|AAB46908.1| fungal protease inhibitor F; FPI-F [Bombyx mori] Fungal protease inhibitor F precursor (FPI-F) fungal protease-specific inhibitor-F [Bombyx mori]
sw15883	BGIBMGA006587	0.32385	0.66795	0.41435	0.5301	No hits found	
sw05186	BGIBMGA002691	0.4457	0.7238	0.62045	0.52915	4.00E-30	gi|66556657|ref|XP_392741.2| PREDICTED: similar to ENSANGP00000009256 [Apis mellifera]
sw22776	BGIBMGA011702	0.3676	0.8467	0.71455	0.5271	e-108	gi|266438|sp|Q00802|L301_BOMMO Low molecular mass 30 kDa lipoprotein 19G1 precursor 30K protein [Bombyx mori]
sw18255	BGIBMGA008059	1.1553	1.04595	1.2418	0.5262	0	gi|9802380|gb|AAF99701.1| aminopeptidase-N [Epiphyas postvittana]
sw00114	BGIBMGA002331	0.4792	0.68125	0.50825	0.5256	No hits found	
sw12328	BGIBMGA010679	2.2333	1.5491	2.3216	0.52185	6.00E-99	gi|25009792|gb|AAN71068.1| AT14391p [Drosophila melanogaster]
sw18516	BGIBMGA014051	2.25195	2.3268	1.5738	0.5191	Bmb038798	2.00E-26
sw08892	BGIBMGA013446	0.0227	0.563	0.61075	0.5174	2.00E-15	gi|89298896|gb|EAR96884.1| EF hand family protein [Tetrahymena thermophila SB210]
sw02367	BGIBMGA012795	0.3029	0.6772	0.4892	0.51495	No hits found	
sw14775	BGIBMGA003551	0.25935	0.6381	0.47755	0.5141	4.00E-08	gi|24584084|ref|NP_609635.1| CG15639-PA [Drosophila melanogaster] CG15639-PA [Drosophila melanogaster]
sw20411	BGIBMGA011039	1.10595	0.95445	1.13175	0.5123	0	gi|1335781|gb|AAC47078.1| Cap
sw09361	BGIBMGA014458	0.04405	0.605	0.6375	0.51145	No hits found	
sw10237	BGIBMGA011530	1	1	1	0.51075	Bmb047540	2.00E-19
sw00689	BGIBMGA009276	2.7305	1.3968	0.99665	0.5107	2.00E-63	gi|85070116|gb|ABC69733.1| vermilion [Mayetiola destructor]
sw08370	BGIBMGA011219	1	1	1	0.50845	7.00E-05	gi|89299669|gb|EAR97657.1| hypothetical protein TTHERM_00617800 [Tetrahymena thermophila SB210]
sw13495	BGIBMGA002543	0.8842	1.44845	1	0.50705	1.00E-27	gi|45554605|ref|NP_996387.1| Moesin CG10701-PJ, isoform J [Drosophila melanogaster] CG10701-PJ, isoform J [Drosophila melanogaster] Moesin/ezrin/radixin homolog 1
sw18226	BGIBMGA009415	0.1069	0.58365	0.73915	0.50605	3.00E-35	gi|74096331|ref|NP_001027762.1| protofilament ribbon protein [Ciona intestinalis] protofilament ribbon protein [Ciona intestinalis]
sw06874	BGIBMGA011827	0.9761	1.1317	1.1057	0.50535	Bmb036467	e-117
sw19093	BGIBMGA007583	0.8701	0.91145	0.88865	0.5049	0	gi|12006108|gb|AAG44738.1| IRA1 [Mus musculus]
sw02606	BGIBMGA004767	1	1	1	0.50475	Bmb044985	5.00E-07
sw13722	BGIBMGA007416	1.11015	1.26845	1.4392	0.50455	Bmb020359	4.00E-07
sw13248	BGIBMGA012800	1	1	1	0.50415	6.00E-59	gi|68369674|ref|XP_706825.1| PREDICTED: similar to Solute carrier family 13 (sodium-dependent dicarboxylate transporter), member 2 isoform 3 [Danio rerio]
sw02667	BGIBMGA003825	1	1.0819	1.6455	0.5032	4.00E-25	gi|66555679|ref|XP_396164.2| PREDICTED: similar to kinesin family member 3A [Apis mellifera]
sw19228	BGIBMGA010386	1	1	1	0.5028	No hits found	
sw15828	BGIBMGA006540	1.11345	0.97395	0.81945	0.50205	No hits found	

sw13776	BGIBMGA005847	1	1	1	0.502	2.00E-13	gi|55637015|ref|XP_508774.1| PREDICTED: similar to multi-transmembrane domain immunoglobulin-like protein [Pan troglodytes]
sw14006	BGIBMGA007332	0.71815	0.8286	1.0654	0.5015	3.00E-19	gi|51262008|gb|AAH80137.1| 26S protease regulatory subunit 7 [Xenopus tropicalis]
sw12787	BGIBMGA005014	1	0.8143	1.05785	0.5014	Bmb014393	8.00E-27
sw12759	BGIBMGA008725	0.96505	1.155	1.29915	0.5011	Bmb014178	0
sw12453	BGIBMGA007081	1	1	1	0.5009	2.00E-72	gi|6678403|ref|NP_033436.1| topoisomerase (DNA) III alpha [Mus musculus] Topoisomerase (DNA) III alpha [Mus musculus] topoisomerase (DNA) III alpha [Mus musculus] DNA topoisomerase III alpha topoisomerase III [Mus musculus]
sw13708	BGIBMGA002823	1	1	1	0.5005	2.00E-09	gi|55238357|gb|EAL39856.1| ENSANGP00000025455 [Anopheles gambiae str. PEST] ENSANGP00000025455 [Anopheles gambiae str. PEST]
sw15904	BGIBMGA011445	1.01865	1.0818	1.31745	0.5005	No hits found	
sw01994	BGIBMGA007728	0.69965	0.75045	0.9522	0.50025	Bmb031269	4.00E-50
sw16144	BGIBMGA008746	0.87265	1.0275	0.8905	0.4999	Bmb038552	4.00E-31
sw03617	BGIBMGA008719	1	1	1	0.4997	e-132	gi|50756593|ref|XP_415231.1| PREDICTED: similar to cain [Gallus gallus]
sw17479	BGIBMGA009941	0.8262	0.941	1.2255	0.4995	3.00E-24	gi|73997326|ref|XP_867414.1| PREDICTED: similar to Ubiquitin isopeptidase T isoform 4 [Canis familiaris]
sw21175	BGIBMGA000302	1	1	1	0.49925	9.00E-95	gi|49903743|gb|AAH76951.1| MGC89333 protein [Xenopus tropicalis] MGC89333 protein [Xenopus tropicalis]
sw09464	BGIBMGA003016	1	1	1	0.49915	e-105	gi|55237368|gb|EAA12425.2| ENSANGP00000018252 [Anopheles gambiae str. PEST] ENSANGP00000018252 [Anopheles gambiae str. PEST]
sw16277	BGIBMGA008061	1.8968	1.4994	1.8734	0.49915	2.00E-39	gi|2687733|emb|CAA10950.1| aminopeptidase N [Plutella xylostella]
sw02073	BGIBMGA011733	0.8892	0.94335	0.8051	0.4991	2.00E-38	gi|55614029|ref|XP_515921.1| PREDICTED: similar to G protein-coupled receptor 155 [Pan troglodytes]
sw14292	BGIBMGA007975	1	1	1	0.4988	6.00E-34	gi|82891212|ref|XP_915569.1| PREDICTED: similar to early estrogen-induced gene 1 protein [Mus musculus]
sw21282	BGIBMGA001739	1	1	0.91885	0.4988	Bmb035015	9.00E-39
sw19341	BGIBMGA007497	1.0785	0.8713	1.1468	0.4987	2.00E-13	gi|18204091|gb|AAH21382.1| Methylcrotonoyl-Coenzyme A carboxylase 1 (alpha) [Mus musculus] methylcrotonoyl-Coenzyme A carboxylase 1 (alpha) [Mus musculus]
sw13288	BGIBMGA010068	1	1	1	0.49865	4.00E-68	gi|37681881|gb|AAQ97818.1| N-ethylmaleimide-sensitive factor attachment protein, alpha [Danio rerio] N-ethylmaleimide sensitive fusion protein attachment protein alpha
sw19089	BGIBMGA013662	1.1869	1.1276	1	0.4985	1.00E-69	gi|72133877|ref|XP_788695.1| PREDICTED: similar to predicted CDS, reverse transcriptase family member (1O881) [Strongylocentrotus purpuratus]
sw18280	BGIBMGA013223	1.38775	0.9026	1.1243	0.49835	1.00E-40	gi|68697272|emb|CAJ14165.1| BEL12_AG transposon polyprotein [Anopheles gambiae]
sw12809	BGIBMGA008139	0.57605	0.80405	0.7336	0.49825	2.00E-76	gi|25009665|gb|AAN71009.1| AT01055p [Drosophila melanogaster]
sw19867	BGIBMGA007441	1	1	1	0.49785	e-112	gi|21483348|gb|AAM52649.1| GM13306p [Drosophila melanogaster]
sw13960	BGIBMGA008063	1	1	1	0.4978	Bmb021898	6.00E-13
sw03188	BGIBMGA002999	0.9438	1.0292	1.07095	0.49745	9.00E-13	gi|66734432|gb|AAY53605.1| tetraspanin family protein [Branchiostoma belcheri tsingtaunese]
sw18185	BGIBMGA014217	1.0471	1.107	1.30895	0.49735	2.00E-61	gi|67938380|ref|ZP_00530906.1| Acetyl-CoA hydrolase/transferase [Chlorobium phaeobacteroides BS1] Acetyl-CoA hydrolase/transferase [Chlorobium phaeobacteroides BS1]
sw08505	BGIBMGA003615	1	1	1	0.49695	4.00E-41	gi|28317242|gb|AAO39628.1| GH01001p [Drosophila melanogaster]
sw11338	BGIBMGA009280	1.0438	1.009	1.1422	0.4966	1.00E-11	gi|55661397|ref|XP_525616.1| PREDICTED: protein kinase C and casein kinase substrate in neurons 2 [Pan troglodytes]

sw20627	BGIBMGA002460	1.0743	1.01205	1.3058	0.49655	e-123	gi|13446610|emb|CAC35051.1| putative exoribonuclease DIS3 [Drosophila melanogaster]
sw03847	BGIBMGA002370	1.1255	1.08535	1.0468	0.49645	9.00E-22	gi|72011764|ref|XP_780861.1| PREDICTED: similar to ring finger protein 111 [Strongylocentrotus purpuratus]
sw06585	BGIBMGA000624	1	1	1	0.4963	0	gi|22474516|dbj|BAC10620.1| Titin-like protein [Bombyx mori]
sw20393	BGIBMGA007769	1	1	1	0.49625	8.00E-62	gi|62484234|ref|NP_609215.3| CG7627-PA [Drosophila melanogaster] CG7627-PA [Drosophila melanogaster]
sw11411	BGIBMGA004281	0.29325	0.63425	0.64015	0.4962	2.00E-06	gi|66546699|ref|XP_393497.2| PREDICTED: similar to ENSANGP00000007226 [Apis mellifera]
sw16215	BGIBMGA000715	0.68865	0.7152	0.7437	0.4961	Bmb039191	6.00E-43
sw09610	BGIBMGA013588	1	1	1	0.49595	2.00E-08	gi|89307050|gb|EAS05038.1| Protein kinase domain containing protein [Tetrahymena thermophila SB210]
sw10128	BGIBMGA001936	1	1	1	0.4956	Bmb043791	No hits found
sw04620	BGIBMGA007806	0.9626	0.7959	1.0125	0.4952	1.00E-07	gi|66506776|ref|XP_395965.2| PREDICTED: similar to CG5859-PA [Apis mellifera]
sw08112	BGIBMGA010701	1.09325	1.02725	1.24175	0.49515	1.00E-49	gi|72132938|ref|XP_794780.1| PREDICTED: similar to Sideroflexin-1 (Tricarboxylate carrier protein) [Strongylocentrotus purpuratus]
sw12526	BGIBMGA005140	1.08675	0.9006	1.04735	0.49515	3.00E-12	gi|1234789|gb|AAC59866.1| fos-related antigen-2
sw20057	BGIBMGA002286	1	1	1	0.4951	3.00E-24	gi|66532890|ref|XP_395425.2| PREDICTED: similar to GA13874-PA [Apis mellifera]
sw20466	BGIBMGA007283	0.0094	0.57985	0.7322	0.4951	2.00E-27	gi|50758492|ref|XP_425404.1| PREDICTED: similar to NaDC-2 [Gallus gallus]
sw18131	BGIBMGA007772	1.74765	1.34885	0.91275	0.49505	2.00E-31	gi|72043224|ref|XP_787838.1| PREDICTED: similar to TPR repeat containing protein (XH300), partial [Strongylocentrotus purpuratus]
sw08436	BGIBMGA013698	1.17255	1.0619	1.25225	0.495	1.00E-14	gi|71834271|gb|AAZ41808.1| AT28579p [Drosophila melanogaster]
sw11778	BGIBMGA003505	1	1	1	0.49455	7.00E-29	gi|48095153|ref|XP_392250.1| PREDICTED: similar to PDZ domain containing 3 [Apis mellifera]
sw11236	BGIBMGA010816	1	1	1	0.49445	Bmb004519	2.00E-18
sw03881	BGIBMGA000450	1	1	1	0.4938	4.00E-24	gi|18858733|ref|NP_571286.1| GATA-binding protein 3 [Danio rerio] GATA-binding protein 3 [Danio rerio] transcription factor; GATA 3 homolog [Danio rerio] Transcription factor GATA-3 (GATA-binding factor 3)
sw02097	BGIBMGA011797	1	1	1	0.49325	e-132	gi|904101|gb|AAA74931.1| helicase
sw10602	BGIBMGA007763	1	1	1	0.4931	No hits found	
sw08422	BGIBMGA012370	1	1	1	0.49305	2.00E-09	gi|72157704|ref|XP_787309.1| PREDICTED: similar to protein tyrosine phosphatase, receptor type, T, partial [Strongylocentrotus purpuratus]
sw11815	BGIBMGA000745	0.7771	1.1142	1.3569	0.49295	1.00E-18	gi|28972592|dbj|BAC65712.1| mKIAA1049 protein [Mus musculus]
sw11167	BGIBMGA008523	0.9054	0.82185	0.9167	0.49275	8.00E-34	gi|66562766|ref|XP_625122.1| PREDICTED: similar to ATP-binding cassette, sub-family B, member 10 [Apis mellifera]
sw12034	BGIBMGA011646	0.81095	0.8295	0.85655	0.4927	6.00E-46	gi|54643291|gb|EAL32035.1| GA14705-PA [Drosophila pseudoobscura]
sw20109	BGIBMGA005944	0.8033	0.81395	0.83995	0.4926	5.00E-70	gi|66529633|ref|XP_395559.2| PREDICTED: similar to PNUTSDm protein [Apis mellifera]
sw20807	BGIBMGA006561	0.6429	0.6714	0.96685	0.4924	1.00E-26	gi|600837|gb|AAC46947.1| mariner transposase mariner transposase
sw12259	BGIBMGA008926	0.76225	1	1	0.4921	1.00E-37	gi|28972347|dbj|BAC65627.1| mKIAA0684 protein [Mus musculus]
sw16574	BGIBMGA004976	1	1	1	0.4916	Bmb043588	No hits found

sw17562	BGIBMGA004344	0.9686	1.0089	1.13115	0.4916	e-118	gi|3913717|sp|O16118|GNAS_HOMAM Guanine nucleotide-binding protein G(s), alpha subunit (Adenylate cyclase-stimulating G alpha protein) G-protein alpha s subunit [Homarus americanus]
sw14906	BGIBMGA009912	1	1	1	0.4915	1.00E-62	gi|72050581|ref|XP_795530.1| PREDICTED: similar to Protein-S-isoprenylcysteine O-methyltransferase (Isoprenylcysteine carboxylmethyltransferase)
sw16397	BGIBMGA012910	1.01825	0.94705	0.9983	0.4914	Bmb041158	2.00E-18
sw02208	BGIBMGA007189	1	1	1	0.49125	3.00E-08	gi|68432309|ref|XP_687509.1| PREDICTED: similar to zinc finger protein ZFP235, partial [Danio rerio]
sw06259	BGIBMGA005386	1	1	1	0.49115	9.00E-61	gi|40254842|ref|NP_036419.2| centaurin, beta 2 [Homo sapiens] Centaurin, beta 2 [Homo sapiens]
sw18260	BGIBMGA009993	1.01145	1.0162	1.1017	0.4911	3.00E-17	gi|25012713|gb|AAN71450.1| RE59368p [Drosophila melanogaster]
sw20577	BGIBMGA004785	0.8212	0.88695	1.14845	0.4911	3.00E-35	gi|29122957|gb|AAO65770.1| SEL1L [Rattus norvegicus] Sel1 (suppressor of lin-12) 1 homolog [Rattus norvegicus]
sw00009	BGIBMGA004744	1.01155	1.0598	1.30105	0.49105	No hits found	
sw19571	BGIBMGA003292	0.5308	0.82355	0.6202	0.4909	No hits found	
sw17637	BGIBMGA001929	1.6046	1.32155	1.5941	0.49075	2.00E-82	gi|1881831|gb|AAB49519.1| 17 beta-hydroxysteroid dehydrogenase type IV; HSD IV [Rattus sp.] Peroxisomal multifunctional enzyme type 2 (MFE-2) (D-bifunctional protein) (DBP)
sw14699	BGIBMGA009420	0.85965	0.91855	0.99795	0.4901	e-126	gi|66540209|ref|XP_624722.1| PREDICTED: similar to Zgc:92093 [Apis mellifera]
sw08986	BGIBMGA011572	1	1	1	0.49005	5.00E-72	gi|66513007|ref|XP_392473.2| PREDICTED: similar to jumonji domain containing 1B [Apis mellifera]
sw05363	BGIBMGA002524	1.30135	1.11705	1.85605	0.48985	No hits found	
sw18679	BGIBMGA005426	1.0438	1.128	1.17635	0.48975	4.00E-12	gi|1519345|gb|AAB61684.1| Pdd1p [Tetrahymena thermophila] Pdd1p [Tetrahymena thermophila]
sw21533	BGIBMGA014091	1.2412	1.10745	1.2435	0.48975	Bmb031776	6.00E-39
sw10030	BGIBMGA014491	1	1	1	0.4897	Bmb040649	2.00E-22
sw17713	BGIBMGA008199	0.8495	0.7935	0.9031	0.4895	Bmb041949	No hits found
sw08310	BGIBMGA008133	1	1	1	0.4892	Bmb012854	5.00E-08
sw05659	BGIBMGA005625	1	1	1	0.4888	1.00E-46	gi|51094446|gb|EAL23707.1| unc-84 homolog A (C. elegans) [Homo sapiens]
sw03660	BGIBMGA000408	0.8416	0.81845	0.98335	0.4886	0	gi|6978595|ref|NP_036651.1| calcium/calmodulin-dependent protein kinase II, delta [Rattus norvegicus] Calcium/calmodulin-dependent protein kinase type II delta chain (CaM-kinase II delta chain) (CaM kinase II delta subunit)
sw15792	BGIBMGA007188	0.75305	0.8185	0.9222	0.48805	Bmb035519	6.00E-09
sw00343	BGIBMGA007515	1.18505	0.9829	1.01485	0.488	Bmb004810	5.00E-22
sw15583	BGIBMGA013228	0.8999	1.0036	1.17245	0.488	4.00E-22	gi|25012345|gb|AAN71283.1| RE05346p [Drosophila melanogaster]
sw08772	BGIBMGA013516	1	1	1	0.48785	No hits found	
sw18052	BGIBMGA001754	0.7906	0.9647	0.9049	0.4876	3.00E-14	gi|4574742|gb|AAD24195.1| fragmin A [Physarum polycephalum]
sw13622	BGIBMGA011708	0.90395	1.108	0.8801	0.48755	2.00E-16	gi|72085257|ref|XP_796113.1| PREDICTED: similar to cytochrome P450, family 2, subfamily U, polypeptide 1 [Strongylocentrotus purpuratus]
sw02944	BGIBMGA013134	0.85405	1	1.03555	0.4875	0	gi|2257629|dbj|BAA21483.1| Bm cdc2 [Bombyx mori]
sw08085	BGIBMGA002787	1	1	1	0.4875	No hits found	

sw12981	BGIBMGA011963	1.10685	1.0018	1.02085	0.48735	8.00E-18	gi|75766090|pdb|2A7L|B Chain B, Structure Of The Human Hypothetical Ubiquitin-Conjugating Enzyme, Loc55284 Chain A, Structure Of The Human Hypothetical Ubiquitin-Conjugating Enzyme, Loc55284
sw21120	BGIBMGA005931	1.3421	1.0924	1.5224	0.4868	e-104	gi|1705537|sp|P52826|CACP_COLLI Carnitine O-acetyltransferase precursor (Carnitine acetylase) (CAT) (Carnitine acetyltransferase) (CrAT) carnitine acetyltransferase
sw12975	BGIBMGA002013	1.39895	1.11425	1.2106	0.48645	2.00E-49	gi|28557675|gb|AAO45243.1| GH01576p [Drosophila melanogaster]
sw01551	BGIBMGA009184	0.7233	0.82515	0.7954	0.4863	1.00E-50	gi|6448469|dbj|BAA86911.1| homologue of Sarcophaga 26,29kDa proteinase [Periplaneta americana]
sw19182	BGIBMGA005899	1.15945	0.9304	1.1003	0.4863	0	gi|998377|gb|AAC60521.1| chitooligosaccharidolytic beta-N-acetylglucosaminidase; beta-GlcNAcase [Bombyx mori] Chitooligosaccharidolytic beta-N-acetylglucosaminidase precursor (Beta-GlcNAcase) (Beta-hexosaminidase)
sw21436	BGIBMGA004679	1	1	1	0.4855	Bmb015733	e-121
sw04342	BGIBMGA009045	0.9818	0.99325	1.0633	0.4854	7.00E-46	gi|54643209|gb|EAL31953.1| GA10697-PA [Drosophila pseudoobscura]
sw08981	BGIBMGA001031	0.32505	0.65995	0.5313	0.48535	No hits found	
sw01718	BGIBMGA003361	2.0572	1.40725	1.6631	0.48515	Bmb026803	e-163
sw14816	BGIBMGA009392	1.40235	1.09305	0.8807	0.48505	3.00E-19	gi|18921163|ref|NP_572733.1| CG1561-PA, isoform A [Drosophila melanogaster] HL08023p [Drosophila melanogaster] CG1561-PA, isoform A [Drosophila melanogaster]
sw15044	BGIBMGA008214	1.2036	1.3133	1.50075	0.48465	1.00E-10	gi|2429444|gb|AAB70982.1| Fumarase protein 1, isoform a [Caenorhabditis elegans] FUMarase family member (fum-1) [Caenorhabditis elegans] Probable fumarate hydratase, mitochondrial precursor (Fumarase)
sw14872	BGIBMGA005056	1	1	1	0.4846	1.00E-24	gi|68365252|ref|XP_683807.1| PREDICTED: similar to Potential helicase MOV-10 (Moloney leukemia virus 10 protein) [Danio rerio]
sw01916	BGIBMGA006246	0.7103	0.8057	1.0679	0.48455	Bmb029821	5.00E-07
sw06500	BGIBMGA007579	0.9858	0.9712	0.8829	0.4842	Bmb028550	4.00E-06
sw17505	BGIBMGA001982	1	1	1	0.4841	Bmb039542	2.00E-08
sw06013	BGIBMGA008612	1	1	1	0.48375	7.00E-67	gi|76654637|ref|XP_584546.2| PREDICTED: similar to phosphatidylinositol 4-kinase type II isoform 1 [Bos taurus]
sw19478	BGIBMGA013251	1.06245	1	0.8891	0.48345	No hits found	
sw12571	BGIBMGA010030	1.193	1.0443	0.93045	0.4834	3.00E-05	gi|54114942|tpg|DAA01820.1| TPA: TPA_inf: HN1-like protein [Anopheles gambiae]
sw00586	BGIBMGA001883	0.797	1.0591	1.02395	0.4831	No hits found	
sw18406	BGIBMGA005190	1	1	1	0.4831	2.00E-15	gi|66506685|ref|XP_392322.2| PREDICTED: similar to 1-phosphatidylinositol-4,5-bisphosphate phosphodiesterase beta 1 (Phosphoinositide phospholipase C) (PLC-beta-1) (Phospholipase C-beta-1) (PLC-I) (PLC-154) [Apis mellifera]
sw22526	BGIBMGA008831	0.2487	0.32995	0.2859	0.4831	Bmb006706	3.00E-12
sw11177	BGIBMGA009836	1	1	1	0.48265	8.00E-11	gi|71980134|gb|AAZ57345.1| RE33938p [Drosophila melanogaster]
sw12936	BGIBMGA012958	1.2202	1.05595	0.96765	0.48265	No hits found	
sw06996	BGIBMGA009453	1.0119	1.05165	1	0.4825	Bmb039791	1.00E-13
sw09256	BGIBMGA003034	0.6843	1	0.7545	0.48215	6.00E-26	gi|46329861|gb|AAH68420.1| Wu:fj56a04 protein [Danio rerio]
sw05389	BGIBMGA011675	1	0.88225	1	0.48135	1.00E-57	gi|66514417|ref|XP_396199.2| PREDICTED: similar to Nuclear pore complex protein Nup160 homolog [Apis mellifera]
sw16324	BGIBMGA013348	0.86955	1.0123	1.2337	0.48115	3.00E-60	gi|25282451|ref|NP_741992.1| damage-specific DNA binding protein 1 [Rattus norvegicus] damage-specific DNA binding protein 1 [Rattus norvegicus]
sw19824	BGIBMGA014503	1.41625	1.18185	1.4305	0.48115	e-135	gi|54261662|gb|AAH84614.1| LOC495282 protein [Xenopus laevis]

sw01281	BGIBMGA008442	1.8548	1.2141	1.42995	0.48095	0	gi|550486|gb|AAB64306.1| pyruvate carboxylase [Aedes aegypti]
sw21184	BGIBMGA010472	1	1	1	0.4809	e-165	gi|28416396|gb|AAO42670.1| AT10293p [Drosophila melanogaster]
sw00060	BGIBMGA003143	1	1	1	0.48065	8.00E-88	gi|24640586|ref|NP_572476.2| CG12125-PA [Drosophila melanogaster] RE13562p [Drosophila melanogaster] CG12125-PA [Drosophila melanogaster]
sw21481	BGIBMGA010159	1	1	1	0.48065	2.00E-41	gi|68697272|emb|CAJ14165.1| BEL12_AG transposon polyprotein [Anopheles gambiae]
sw11740	BGIBMGA000661	0.77255	0.9003	0.94075	0.4805	3.00E-48	gi|68366688|ref|XP_683991.1| PREDICTED: similar to Protein disulfide-isomerase A5 precursor (Protein disulfide isomerase-related protein) [Danio rerio]
sw08817	BGIBMGA000097	1	1	1.2215	0.48	2.00E-44	gi|66505197|ref|XP_394928.2| PREDICTED: similar to calpain B [Apis mellifera]
sw13228	BGIBMGA009370	0.82175	1.05055	1.07955	0.47985	4.00E-38	gi|74002990|ref|XP_545165.2| PREDICTED: similar to Carboxypeptidase N subunit 2 precursor (Carboxypeptidase N polypeptide 2) (Carboxypeptidase N 83 kDa chain) (Carboxypeptidase N regulatory subunit)
sw15267	BGIBMGA007626	0.37545	0.6649	0.5048	0.4798	6.00E-36	gi|422486|pir||A40734 Pas (Passover) protein - fruit fly (Drosophila melanogaster) passover
sw00085	BGIBMGA009829	1.1777	0.97055	1	0.4797	2.00E-27	gi|86563230|ref|NP_001033372.1| T15B12.1b [Caenorhabditis elegans]
sw18488	BGIBMGA011368	1.24315	1	1.44545	0.47965	Bmb036336	1.00E-88
sw19381	BGIBMGA002114	1	1	1	0.47945	0	gi|157804|gb|AAA28664.1| laminin B2 chain
sw08473	BGIBMGA014226	1	1	1	0.4792	0	gi|55236350|gb|EAA43619.2| ENSANGP00000025304 [Anopheles gambiae str. PEST] ENSANGP00000025304 [Anopheles gambiae str. PEST]
sw05210	BGIBMGA000175	0.91275	0.99645	1.05535	0.47915	7.00E-48	gi|72016095|ref|XP_786114.1| PREDICTED: similar to testis expressed gene 2 [Strongylocentrotus purpuratus]
sw01110	BGIBMGA003129	0.8711	1.2437	1.73625	0.479	2.00E-27	gi|55244191|gb|EAL41409.1| ENSANGP00000027433 [Anopheles gambiae str. PEST] ENSANGP00000027433 [Anopheles gambiae str. PEST]
sw10534	BGIBMGA003525	1.1379	1.0823	1.0144	0.47895	No hits found	
sw00266	BGIBMGA001435	1	1	1	0.47865	2.00E-97	gi|12053709|emb|CAC20419.1| a disintegrin-like and metalloprotease (reprolysin type) with thrombospondin type 1 motif, 12 [Homo sapiens] ADAMTS-12 precursor
sw20958	BGIBMGA003261	0.6892	1.13025	1.2142	0.47865	No hits found	
sw15943	BGIBMGA006848	1.09115	1.0954	1.1319	0.4786	3.00E-12	gi|87162631|gb|ABD28426.1| RNA-directed DNA polymerase (Reverse transcriptase); HMG-I and HMG-Y, DNA-binding [Medicago truncatula]
sw06716	BGIBMGA012439	0.7479	0.3609	0.85685	0.4785	5.00E-65	gi|66522495|ref|XP_393918.2| PREDICTED: similar to subtilisin-related protease SPC3 [Apis mellifera]
sw18519	BGIBMGA008389	1	1	1	0.47845	3.00E-57	gi|61815768|gb|AAX56336.1| ikk-like protein [Pinctada fucata]
sw09407	BGIBMGA006153	1.27705	1.0442	1.13265	0.478	6.00E-28	gi|76686050|ref|XP_598148.2| PREDICTED: similar to tubulin tyrosine ligase-like family, member 3 isoform 1 [Bos taurus]
sw03208	BGIBMGA007491	1.0491	0.9533	1.07075	0.47795	7.00E-26	gi|34098381|sp|O54916|REPS1_MOUSE RalBP1-associated Eps domain-containing protein 1 (RalBP1-interacting protein 1) RalBP1 associated Eps domain containing protein [Mus musculus] RalBP1-associated EH domain protein Reps1 [M
sw17476	BGIBMGA004017	1.04045	1	1.36385	0.47755	Bmb034239	2.00E-76
sw05148	BGIBMGA002404	1	1.3195	1	0.4774	7.00E-06	gi|72126814|ref|XP_790069.1| PREDICTED: similar to step II splicing factor SLU7 [Strongylocentrotus purpuratus]
sw12902	BGIBMGA013725	1.0536	0.8513	1.0187	0.4773	4.00E-14	gi|54644089|gb|EAL32831.1| GA10154-PA [Drosophila pseudoobscura]
sw21300	BGIBMGA011190	1	1	1	0.4773	2.00E-59	gi|83595261|gb|ABC25082.1| MAP kinase activated protein-kinase-2 [Glossina morsitans morsitans]
sw19519	BGIBMGA012042	0.7581	0.6963	0.6869	0.4772	Bmb009746	2.00E-23
sw18746	BGIBMGA009879	1	1	1	0.47715	1.00E-75	gi|55239964|gb|EAA10272.2| ENSANGP00000012355 [Anopheles gambiae str. PEST] ENSANGP00000012355 [Anopheles gambiae str. PEST]

sw07485	BGIBMGA009929	1	1	1	0.47695	Bmb001229	e-132
sw07781	BGIBMGA008409	0.27185	0.64455	0.5084	0.47675	No hits found	
sw22270	BGIBMGA001572	1	1	1	0.47675	2.00E-52	gi|72036140|ref|XP_797170.1| PREDICTED: similar to DEAH (Asp-Glu-Ala-His) box polypeptide 34 isoform 1, partial [Strongylocentrotus purpuratus]
sw08841	BGIBMGA006895	1	1	1	0.4767	6.00E-26	gi|72128018|ref|XP_782784.1| PREDICTED: similar to cell division cycle 27 homolog [Strongylocentrotus purpuratus]
sw20782	BGIBMGA000312	0.99745	0.9009	1.3984	0.4767	4.00E-46	gi|42542379|ref|NP_005830.2| serine/arginine repetitive matrix 1 [Homo sapiens] serine\/arginine repetitive matrix 1 [Homo sapiens] serine\/arginine repetitive matrix 1 [Homo sapiens] Serine/arginine repetitive matrix protein 1 (Ser/Arg-related
sw02310	BGIBMGA005381	0.74495	0.86105	1.00455	0.4764	4.00E-79	gi|72179523|ref|XP_786725.1| PREDICTED: similar to HIV-1 Rev binding protein 2 homolog [Strongylocentrotus purpuratus]
sw06061	BGIBMGA005520	1	1	1	0.47595	Bmb020029	1.00E-84
sw01968	BGIBMGA012310	1	1	1	0.4756	Bmb030765	No hits found
sw09834	BGIBMGA013098	1.1761	1.30185	1.0472	0.47545	3.00E-15	gi|4090968|gb|AAD09281.1| immune-related Hdd13 [Hyphantria cunea]
sw16590	BGIBMGA001038	1	1	1	0.47545	Bmb043759	2.00E-16
sw02009	BGIBMGA011071	1	1	1	0.4753	2.00E-49	gi|7242181|ref|NP_035246.1| phospholipase D family, member 3 [Mus musculus] Pld3 protein [Mus musculus] schwannoma-associated protein [Mus musculus]
sw17762	BGIBMGA008582	1.3874	1.40495	0.7273	0.4753	2.00E-45	gi|2058458|gb|AAB53257.1| p260 [Bombyx mori]
sw01173	BGIBMGA008336	1	1	1	0.47495	7.00E-24	gi|33604134|gb|AAH56332.1| Glycogen synthase kinase 3 alpha [Danio rerio] Glycogen synthase kinase 3 alpha [Danio rerio] glycogen synthase kinase 3 alpha [Danio rerio] glycogen synthase kinase 3 alpha [Danio rerio]
sw22349	BGIBMGA001979	0.7122	0.7865	0.9687	0.47495	Bmb033615	5.00E-33
sw16072	BGIBMGA012362	1	1	1	0.47475	2.00E-11	gi|1572721|gb|AAB09089.1| megakaryocyte stimulating factor; MSF [Homo sapiens]
sw00076	BGIBMGA009332	1.1022	1.16605	1.25125	0.4745	4.00E-05	gi|40675335|gb|AAH64870.1| Nolc1-prov protein [Xenopus tropicalis]
sw09439	BGIBMGA000189	1	1	1	0.4744	3.00E-08	gi|66564860|ref|XP_396938.2| PREDICTED: similar to Zgc:100945 [Apis mellifera]
sw12154	BGIBMGA004365	1	1	1	0.4744	e-136	gi|66546886|ref|XP_392978.2| PREDICTED: similar to A disintegrin and metalloproteinase with thrombospondin motifs like [Apis mellifera]
sw09934	BGIBMGA005037	1	1	1	0.47415	9.00E-91	gi|62859265|ref|NP_001016149.1| SCY1-like 1 [Xenopus tropicalis] SCY1-like 1 (S. cerevisiae) [Xenopus tropicalis]
sw04615	BGIBMGA004037	1.0333	0.91955	1.0599	0.474	No hits found	
sw11052	BGIBMGA006475	0.921	0.99005	1.02385	0.47385	1.00E-20	gi|58332274|ref|NP_001011285.1| Novel protein similar to ras-related C3 botulinum toxin substrate 1 (rho family, small GTP binding protein Rac1) [Xenopus tropicalis] Novel protein similar to ras-related C3 botulinum toxin substrate 1 (rho fam
sw19303	BGIBMGA013241	2.51715	1	5.5106	0.47385	1.00E-73	gi|81248546|gb|ABB69054.1| cytochrome P450 [Helicoverpa armigera]
sw00240	BGIBMGA011161	0.89105	0.8879	1.12185	0.4737	2.00E-26	gi|55637015|ref|XP_508774.1| PREDICTED: similar to multi-transmembrane domain immunoglobulin-like protein [Pan troglodytes]
sw04136	BGIBMGA001746	1.091	1.10715	1.19115	0.47365	9.00E-06	gi|25012376|gb|AAN71297.1| RE10019p [Drosophila melanogaster]
sw10762	BGIBMGA001091	1.655	1.1852	1.2883	0.47365	e-170	gi|58394466|ref|XP_320745.2| ENSANGP00000008856 [Anopheles gambiae str. PEST] ENSANGP00000008856 [Anopheles gambiae str. PEST]
sw16625	BGIBMGA003913	1.01355	1.02855	0.9757	0.4733	2.00E-06	gi|55242814|gb|EAA07246.2| ENSANGP00000016000 [Anopheles gambiae str. PEST] ENSANGP00000016000 [Anopheles gambiae str. PEST]
sw05774	BGIBMGA004413	1	1.2193	1	0.47325	8.00E-91	gi|6690786|gb|AAF24342.1| Short stop/Kakapo truncated isoform [Drosophila melanogaster]
sw17987	BGIBMGA006908	1	1	1	0.4731	4.00E-59	gi|6746588|gb|AAF27637.1| ecdysone-inducible gene E1 [Drosophila melanogaster]

sw06909	BGIBMGA004860	0.8923	0.8492	0.91325	0.473	No hits found	
sw07696	BGIBMGA012705	0.86975	1.03355	1.1208	0.47295	No hits found	
sw15449	BGIBMGA006374	1	1	1	0.4729	1.00E-27	gi|50417096|gb|AAH77105.1| Zgc:110543 protein [Danio rerio]
sw06556	BGIBMGA002539	1.15075	1.3131	1.49925	0.47285	e-134	gi|5852166|emb|CAB55500.1| vacuolar ATPase subunit a [Manduca sexta]
sw07516	BGIBMGA007912	0.933	0.94945	0.9173	0.47265	5.00E-55	gi|62857739|ref|NP_001017231.1| retinol dehydrogenase 14 (all-trans and 9-cis) [Xenopus tropicalis] retinol dehydrogenase 14 (all-trans and 9-cis) [Xenopus tropicalis]
sw01185	BGIBMGA006298	1.1829	0.7722	1.14265	0.4724	No hits found	
sw01562	BGIBMGA010477	1	0.86775	1	0.472	8.00E-48	gi|19920884|ref|NP_609121.1| CG12789-PB, isoform B [Drosophila melanogaster] GH23019p [Drosophila melanogaster] CG12789-PB, isoform B [Drosophila melanogaster]
sw12903	BGIBMGA003733	0.5505	0.7905	0.94585	0.47195	2.00E-54	gi|14318618|gb|AAH09107.1| Microtubule associated serine/threonine kinase-like [Homo sapiens] novel protein kinase domain containing protein [Homo sapiens] Microtubule-associated serine/threonine-protein kinase-like
sw18569	BGIBMGA006643	1	0.69605	1	0.47155	No hits found	
sw02688	BGIBMGA000002	1.0264	0.9973	1.09035	0.4714	No hits found	
sw20197	BGIBMGA002833	0.26265	0.6422	0.84225	0.47115	6.00E-32	gi|28569894|dbj|BAC57926.1| reverse transcriptase [Bombyx mori]
sw18354	BGIBMGA001675	1	1	1	0.47105	Bmb022212	1.00E-05
sw22656	BGIBMGA012864	1	1	1	0.47065	8.00E-05	gi|42780628|ref|NP_977875.1| peptidase, M23/M37 family [Bacillus cereus ATCC 10987] peptidase, M23/M37 family [Bacillus cereus ATCC 10987]
sw19421	BGIBMGA007182	1.11535	1.20385	1	0.47055	1.00E-05	gi|66514893|ref|XP_623699.1| PREDICTED: similar to hypothetical protein [Apis mellifera]
sw00734	BGIBMGA005695	1	1	1	0.47045	0	gi|87248427|gb|ABD36266.1| triacylglycerol lipase [Bombyx mori]
sw13075	BGIBMGA003718	0.6258	1.0061	1.28135	0.47035	2.00E-23	gi|57033136|gb|AAH88952.1| LOC398231 protein [Xenopus laevis]
sw04135	BGIBMGA002475	1.1144	0.9889	1.0629	0.47025	2.00E-06	gi|50748275|ref|XP_426430.1| PREDICTED: similar to papilin [Gallus gallus]
sw13650	BGIBMGA006843	1.21335	1.0163	0.96775	0.47	e-142	gi|82582269|sp|Q6NN85|SSH_DROME Protein phosphatase Slingshot MAP kinase phosphatase [Drosophila melanogaster]
sw05769	BGIBMGA005348	1	1	1	0.46975	3.00E-61	gi|77403901|gb|ABA81829.1| LP20363p [Drosophila melanogaster]
sw19875	BGIBMGA004580	1.44565	1.34015	1.89905	0.46935	8.00E-10	gi|66525417|ref|XP_393163.2| PREDICTED: similar to LIM domain protein [Apis mellifera]
sw21147	BGIBMGA009702	1	1	1	0.46935	7.00E-65	gi|55625576|ref|XP_518161.1| PREDICTED: mannosyl (alpha-1,3-)-glycoprotein beta-1,2-N-acetylglucosaminyltransferase [Pan troglodytes]
sw13631	BGIBMGA005815	1.3336	1.1196	1.0373	0.4693	2.00E-22	gi|27374226|gb|AAO00988.1| CG9715-PA [Drosophila erecta]
sw17508	BGIBMGA007020	0.95985	1.05165	0.9168	0.4693	Bmb040665	6.00E-16
sw11161	BGIBMGA007407	1.05785	1.0239	1.082	0.46925	9.00E-11	gi|28376664|ref|NP_777550.1| AN1, ubiquitin-like, homolog [Homo sapiens] ubiquitin-like fusion protein [Homo sapiens]
sw14431	BGIBMGA013163	1.3623	1.0402	0.8759	0.4687	4.00E-05	gi|48427980|sp|P82120|CUO7_BLACR Cuticle protein 7 (BcNCP15.0)
sw08189	BGIBMGA009770	1	1.1388	1	0.4684	Bmb011161	6.00E-16
sw08237	BGIBMGA011482	1.44795	0.834	1.1394	0.46795	1.00E-72	gi|62243588|ref|NP_001009573.2| unc-13 homolog D [Mus musculus]
sw18527	BGIBMGA009139	1.19625	1.0796	0.8368	0.46795	Bmb039398	3.00E-49

sw07654	BGIBMGA002463	1	0.9869	0.9511	0.4679	9.00E-08	gi|70795028|gb|AAZ08477.1| relish [Nasutitermes fumigatus]
sw06920	BGIBMGA009812	1	0.9618	0.94095	0.46775	2.00E-09	gi|76635779|ref|XP_613206.2| PREDICTED: similar to SWAP-70 protein, partial [Bos taurus]
sw12042	BGIBMGA010596	1.302	1.3354	1.49745	0.46735	e-172	gi|66504706|ref|XP_394350.2| PREDICTED: similar to Retinoblastoma-binding protein 2 (RBBP-2) [Apis mellifera]
sw18327	BGIBMGA010574	0.97565	0.95615	1.172	0.4669	Bmb019451	3.00E-11
sw06208	BGIBMGA004251	1	1	1	0.46685	5.00E-83	gi|18859145|ref|NP_571876.1| ornithine decarboxylase 1 [Danio rerio] Odc1 protein [Danio rerio] Ornithine decarboxylase 1 [Danio rerio] Odc1 protein [Danio rerio] ornithine decarboxylase [Danio rerio] ornithine decarboxylase [Danio rerio]
sw03056	BGIBMGA012226	1	1	1	0.4667	0	gi|18389427|dbj|BAB84191.1| flavoprotein subunit of succinate dehydrogenase [Ascaris suum]
sw05795	BGIBMGA006185	1.6475	1.45195	1.30615	0.46655	4.00E-34	gi|27374255|gb|AAO01012.1| CG30194-PA [Drosophila erecta]
sw08009	BGIBMGA004127	0.56895	0.7822	0.64375	0.4665	1.00E-13	gi|48102198|ref|XP_392753.1| PREDICTED: similar to CG8069-PB, isoform B [Apis mellifera]
sw18350	BGIBMGA008168	1.0012	0.84775	1.22735	0.4664	7.00E-16	gi|9635413|ref|NP_059311.1| ORF163 [Xestia c-nigrum granulovirus] ORF163 [Xestia c-nigrum granulovirus]
sw09524	BGIBMGA004373	1	1	1	0.46625	2.00E-46	gi|66500590|ref|XP_624299.1| PREDICTED: similar to methyltransferase like 3 [Apis mellifera]
sw15406	BGIBMGA004639	0.96035	0.9161	1.0308	0.46595	e-109	gi|83318899|emb|CAJ29888.1| putative frizzled receptor precursor [Paracentrotus lividus]
sw01243	BGIBMGA007449	1	1.00705	1.00905	0.4658	Bmb018637	e-123
sw09161	BGIBMGA014110	1	0.96715	1	0.4657	Bmb025329	1.00E-11
sw22913	BGIBMGA000807	1.31605	1.07965	1.35875	0.4656	e-132	gi|76613083|ref|XP_886710.1| PREDICTED: similar to adenosine monophosphate deaminase 2 (isoform L) isoform 2 isoform 6 [Bos taurus]
sw17551	BGIBMGA001752	1.05965	1.06145	1.03515	0.46545	No hits found	
sw18459	BGIBMGA006039	0.9727	1.1169	1.0061	0.46545	Bmb033073	1.00E-57
sw15031	BGIBMGA013462	1	1	1	0.46525	Bmb029562	e-108
sw05600	BGIBMGA011412	0.8806	0.96775	1.06805	0.46495	e-146	gi|55246316|gb|EAA03807.2| ENSANGP00000013512 [Anopheles gambiae str. PEST] ENSANGP00000013512 [Anopheles gambiae str. PEST]
sw12148	BGIBMGA003350	0.90505	0.8747	1.11095	0.4648	Bmb010467	7.00E-09
sw13431	BGIBMGA009605	1.0252	1.086	1	0.46475	Bmb018356	2.00E-39
sw05999	BGIBMGA009000	0.9841	0.9879	1.0848	0.46465	Bmb018748	9.00E-71
sw22935	BGIBMGA002149	0.81375	0.86875	0.9751	0.46465	1.00E-28	gi|72083766|ref|XP_786661.1| PREDICTED: similar to HLA-B associated transcript 5 [Strongylocentrotus purpuratus]
sw16481	BGIBMGA007922	1.2512	0.91545	1.1665	0.4644	No hits found	
sw22957	BGIBMGA007904	0.84965	1.0666	1.06945	0.4642	4.00E-81	gi|7672773|gb|AAF66635.1| thioredoxin-like protein TXL [Drosophila melanogaster]
sw12334	BGIBMGA008114	1.2777	1.22225	1.1134	0.4637	Bmb011600	e-139
sw12680	BGIBMGA005473	0.9568	0.905	1.06165	0.46365	0	gi|68354800|ref|XP_692515.1| PREDICTED: similar to ATP-binding cassette, sub-family B, member 6, mitochondrial precursor (Mitochondrial ABC transporter 3) (Mt-ABC transporter 3) (ABC transporter umat) [Danio rerio]
sw01726	BGIBMGA010918	1	1	1	0.46355	1.00E-52	gi|54637592|gb|EAL26994.1| GA18806-PA [Drosophila pseudoobscura]
sw14750	BGIBMGA005842	1	1	1	0.46335	Bmb027448	2.00E-23

sw00690	BGIBMGA003072	1	1.004	0.8325	0.463	4.00E-58	gi|28317277|gb|AAO39638.1| AT18914p [Drosophila melanogaster]
sw19070	BGIBMGA002857	1.27895	1.3453	1.1539	0.46295	2.00E-70	gi|55235344|gb|EAA15146.2| ENSANGP00000017210 [Anopheles gambiae str. PEST] ENSANGP00000017210 [Anopheles gambiae str. PEST]
sw14517	BGIBMGA009875	0.96455	1.08945	1.06355	0.4623	2.00E-17	gi|45382201|ref|NP_990133.1| dynamitin [Gallus gallus] dynamitin [Gallus gallus]
sw18111	BGIBMGA005360	1	1	1	0.4622	Bmb039864	1.00E-44
sw09113	BGIBMGA011813	1.06015	0.95365	1	0.4621	e-146	gi|66543768|ref|XP_624319.1| PREDICTED: similar to CG5547-PD, isoform D [Apis mellifera]
sw11861	BGIBMGA009576	0.9507	1.1735	1.03155	0.4617	Bmb008522	No hits found
sw16241	BGIBMGA002779	1.054	1.235	1.35	0.46155	e-163	gi|27544248|dbj|BAC54898.1| supressor of Ty element 16 [Drosophila melanogaster]
sw21613	BGIBMGA005468	0.36605	0.6952	0.56875	0.46125	Bmb043785	5.00E-11
sw06910	BGIBMGA014194	1.06625	1.0108	1.275	0.461	1.00E-84	gi|76640328|ref|XP_871841.1| PREDICTED: similar to vacuolar protein sorting 35 isoform 2 [Bos taurus]
sw18097	BGIBMGA006001	1	1	1	0.46095	Bmb037557	4.00E-24
sw12382	BGIBMGA014602	0.47865	0.62295	1	0.4609	3.00E-07	gi|4322670|gb|AAD16120.1| dentin phosphoryn [Homo sapiens]
sw03369	BGIBMGA008914	1.0034	0.9653	1.0706	0.46085	Bmb009582	0
sw00850	BGIBMGA007400	0.89675	0.8393	0.83085	0.46065	3.00E-11	gi|54637002|gb|EAL26405.1| GA14087-PA [Drosophila pseudoobscura]
sw14035	BGIBMGA000772	0.22435	0.4665	0.7387	0.46045	e-175	gi|73921301|gb|AAG42021.2| juvenile hormone esterase precursor [Manduca sexta]
sw09553	BGIBMGA007554	1	1	1	0.46015	Bmb031613	No hits found
sw15607	BGIBMGA005884	1	1	1	0.46015	1.00E-44	gi|437310|gb|AAA62850.1| nodulin
sw07919	BGIBMGA002675	0.8808	0.9358	1.10605	0.46	5.00E-50	gi|25012709|gb|AAN71448.1| RE59077p [Drosophila melanogaster]
sw14653	BGIBMGA005500	1	1	1	0.46	1.00E-19	gi|72009576|ref|XP_781168.1| PREDICTED: similar to Beta-glucuronidase precursor (Beta-G1) [Strongylocentrotus purpuratus]
sw05069	BGIBMGA007533	1	1	1	0.4597	No hits found	
sw13964	BGIBMGA009814	0.5042	0.9989	0.84195	0.4597	2.00E-06	gi|66564941|ref|XP_396130.2| PREDICTED: similar to Phosphoserine phosphatase (predicted) [Apis mellifera]
sw15492	BGIBMGA007760	1	1	1	0.4597	3.00E-07	gi|11037277|gb|AAG27544.1| BRCT-domain protein MUS101 [Drosophila melanogaster]
sw05761	BGIBMGA008140	1	1	1	0.4595	No hits found	
sw12431	BGIBMGA001064	0.5358	0.707	0.8628	0.4595	2.00E-33	gi|73993635|ref|XP_848946.1| PREDICTED: similar to GalNAc transferase 10 isoform a [Canis familiaris]
sw15990	BGIBMGA009705	1	1	1	0.45905	No hits found	
sw20454	BGIBMGA007507	1.47775	1	1	0.4586	4.00E-48	gi|66508571|ref|XP_395447.2| PREDICTED: similar to FAM [Apis mellifera]
sw18217	BGIBMGA014109	0.9927	1.11705	1.229	0.45855	7.00E-63	gi|8919867|emb|CAB96203.1| acGAP protein [Drosophila melanogaster]
sw12378	BGIBMGA001142	1	1	1	0.45815	Bmb011862	2.00E-25
sw12804	BGIBMGA011577	1	1	1	0.45795	No hits found	

sw10986	BGIBMGA012320	1.0015	1.7574	1.08755	0.45785	1.00E-37	gi|50752092|ref|XP_422648.1| PREDICTED: similar to ubiquitin-conjugating enzyme E2G 2; ubiquitin-conjugating enzyme 7 homolog [Gallus gallus]
sw13461	BGIBMGA009778	0.9711	1.0939	1.0962	0.4576	2.00E-28	gi|27526238|emb|CAC82378.1| Lasp protein [Drosophila melanogaster]
sw13819	BGIBMGA012296	0.4716	0.69235	0.7315	0.4574	No hits found	
sw11982	BGIBMGA009263	1.09445	1.007	1.023	0.45735	Bmb009304	8.00E-08
sw08742	BGIBMGA005508	1.18395	0.9792	0.93645	0.45715	3.00E-87	gi|85857668|gb|ABC86369.1| IP11908p [Drosophila melanogaster]
sw22878	BGIBMGA007971	0.7124	0.9394	1.05065	0.45715	3.00E-40	gi|72110057|ref|XP_795453.1| PREDICTED: similar to Vesicle trafficking protein SEC22b (SEC22 vesicle trafficking protein-like 1) [Strongylocentrotus purpuratus]
sw12475	BGIBMGA010521	1	1	1	0.457	1.00E-44	gi|27806053|ref|NP_776841.1| c-GMP stimulated phosphodiesterase [Bos taurus] cGMP-dependent 3',5'-cyclic phosphodiesterase (Cyclic GMP-stimulated phosphodiesterase) (CGS-PDE) (cGSPDE) cyclic nucleotide phosphodiesterase
sw16402	BGIBMGA005083	0.99265	1.0578	0.9791	0.4568	1.00E-67	gi|217346|dbj|BAA01464.1| prospero [Drosophila melanogaster]
sw02008	BGIBMGA001874	1	1	1	0.45605	3.00E-41	gi|76639910|ref|XP_876225.1| PREDICTED: similar to Synaptic vesicle membrane protein VAT-1 homolog [Bos taurus]
sw15251	BGIBMGA012514	0.5398	0.58165	0.8912	0.4559	Bmb031361	5.00E-83
sw11872	BGIBMGA000544	1	1	1	0.4554	3.00E-35	gi|28317039|gb|AAO39539.1| RE09158p [Drosophila melanogaster]
sw07047	BGIBMGA006909	1	1	1	0.45535	3.00E-11	gi|55245839|gb|EAA45449.2| ENSANGP00000023755 [Anopheles gambiae str. PEST] ENSANGP00000023755 [Anopheles gambiae str. PEST]
sw18001	BGIBMGA002649	1.13305	0.9909	1.11705	0.4551	3.00E-58	gi|66521630|ref|XP_393870.2| PREDICTED: similar to suppressor of forked protein; Su(f) protein [Apis mellifera]
sw04420	BGIBMGA009007	1	1	1	0.455	No hits found	
sw06038	BGIBMGA004394	0.2231	0.8486	0.42615	0.45485	e-137	gi|126415|sp|P09334|LP1_BOMMO Low molecular 30 kDa lipoprotein PBMHP-6 precursor 30K protein precursor [Bombyx mori]
sw18149	BGIBMGA001211	0.83675	0.9238	1.05735	0.45435	2.00E-47	gi|3024649|sp|Q60416|SRBP1_CRIGR Sterol regulatory element-binding protein 1 (SREBP-1) (Sterol regulatory element-binding transcription factor 1) sterol regulatory element binding protein-1
sw09259	BGIBMGA001645	1.00485	1.0848	1.12875	0.45425	No hits found	
sw10855	BGIBMGA007875	0.9541	0.9469	1.2003	0.45395	2.00E-05	gi|89001107|ref|NP_055023.2| dentin sialophosphoprotein preproprotein [Homo sapiens]
sw18633	BGIBMGA001300	1.0431	1.022	1.08125	0.4539	2.00E-98	gi|72034333|ref|XP_798150.1| PREDICTED: similar to Alpha-fetoprotein enhancer binding protein (AT motif-binding factor) (AT-binding transcription factor 1) [Strongylocentrotus purpuratus]
sw20312	BGIBMGA012488	1	1	1	0.4533	Bmb025867	0
sw22884	BGIBMGA002918	1.01065	1.07925	1.0936	0.45325	8.00E-65	gi|62661108|ref|XP_341289.2| PREDICTED: similar to ubiquitin-conjugating enzyme E2E 2 (UBC4/5 homolog, yeast) [Rattus norvegicus]
sw11474	BGIBMGA012907	1	1	1	0.4532	5.00E-50	gi|55243855|gb|EAA06063.2| ENSANGP00000015314 [Anopheles gambiae str. PEST] ENSANGP00000015314 [Anopheles gambiae str. PEST]
sw03540	BGIBMGA005036	1.1444	1.01915	1.0256	0.45285	8.00E-20	gi|73989867|ref|XP_850213.1| PREDICTED: similar to haloacid dehalogenase-like hydrolase domain containing 4 [Canis familiaris]
sw14021	BGIBMGA006781	1	1.13125	1	0.4528	No hits found	
sw20662	BGIBMGA006347	1	1.3296	1.0136	0.45275	Bmb036732	5.00E-69
sw07818	BGIBMGA009333	1	1	1	0.45225	5.00E-05	gi|1657752|gb|AAC50805.1| FE65-like protein
sw18603	BGIBMGA014265	0.98545	0.67695	0.7802	0.4522	3.00E-60	gi|66549046|ref|XP_393144.2| PREDICTED: similar to GA10777-PA [Apis mellifera]
sw09273	BGIBMGA008015	1	1	1	0.4521	3.00E-06	gi|14018320|emb|CAC38353.1| aminopeptidase [Aspergillus niger]

sw22530	BGIBMGA010250	1	1	1	0.45205	No hits found	
sw11040	BGIBMGA010913	1.7618	1.29225	1.41965	0.45185	Bmb003179	2.00E-10
sw12830	BGIBMGA006977	0.59365	1.05935	0.97935	0.45155	1.00E-38	gi|4557233|ref|NP_000008.1| acyl-Coenzyme A dehydrogenase, C-2 to C-3 short chain precursor [Homo sapiens] acyl-CoA dehydrogenase [Homo sapiens] short chain acyl CoA dehydrogenase [Homo sapiens] Acyl-CoA dehydrogenas
sw06913	BGIBMGA012977	1	1	1	0.4515	Bmb037282	5.00E-62
sw15487	BGIBMGA006464	0.99995	1.3431	1.05995	0.45095	Bmb033087	2.00E-15
sw18118	BGIBMGA010689	0.87785	1.2357	1.15185	0.45075	3.00E-29	gi|66558407|ref|XP_624445.1| PREDICTED: similar to ring finger protein 153 [Apis mellifera]
sw04759	BGIBMGA010476	1	1	1	0.4505	Bmb045545	4.00E-09
sw09549	BGIBMGA011040	1.2133	1.0022	1.1347	0.4505	Bmb031586	5.00E-77
sw12833	BGIBMGA008808	1	1	1	0.4503	4.00E-44	gi|1388166|gb|AAB17949.1| Bowel
sw08031	BGIBMGA003874	1.16975	1.07725	1.30215	0.45015	2.00E-18	gi|27820023|gb|AAO25043.1| GM10395p [Drosophila melanogaster]
sw00332	BGIBMGA002635	1.88475	1.33835	1.19615	0.45	1.00E-85	gi|55245443|gb|EAA04375.2| ENSANGP00000009437 [Anopheles gambiae str. PEST] ENSANGP00000009437 [Anopheles gambiae str. PEST]
sw05201	BGIBMGA006127	1	1	1	0.45	Bmb004590	7.00E-30
sw16250	BGIBMGA001583	0.9982	0.9428	1.03515	0.45	1.00E-20	gi|45551904|ref|NP_732009.2| Tropomyosin 1 CG4898-PI, isoform I [Drosophila melanogaster] Tropomyosin 1 CG4898-PC, isoform C [Drosophila melanogaster] GH09289p [Drosophila melanogaster] LD11194p
sw18283	BGIBMGA007137	0.8568	0.9406	1.0921	0.44995	6.00E-66	gi|66532439|ref|XP_396968.2| PREDICTED: similar to vacuolar protein sorting protein 18, partial [Apis mellifera]
sw08886	BGIBMGA007295	1	1	1	0.4498	4.00E-10	gi|2104845|emb|CAA93441.1| platelet glycoprotein V [Mus musculus] Platelet glycoprotein V precursor (GPV) (CD42D antigen)
sw18524	BGIBMGA004740	1	1	1	0.4496	2.00E-05	gi|37595925|gb|AAQ94739.1| signal transducer and activator of transcription [Penaeus monodon]
sw10943	BGIBMGA005916	0.90895	1.021	1.10355	0.4492	5.00E-92	gi|3005601|gb|AAC09329.1| katanin p80 subunit [Strongylocentrotus purpuratus] katanin p80 subunit [Strongylocentrotus purpuratus] Katanin p80 WD40-containing subunit B1 (Katanin p80 subunit B1) (p80 katanin)
sw11220	BGIBMGA001828	1.2557	1.1407	1.12055	0.449	No hits found	
sw09342	BGIBMGA007584	0.9094	0.8298	0.8951	0.44885	Bmb028131	1.00E-12
sw04210	BGIBMGA005356	1	1	1	0.44875	4.00E-06	gi|2618772|gb|AAC98527.1| cytochrome P450 CYP12A2 [Musca domestica] Cytochrome P450 CYP12A2 (CYPXIIA2)
sw05778	BGIBMGA002642	0.87625	0.94205	0.849	0.44865	4.00E-06	gi|72011023|ref|XP_780724.1| PREDICTED: hypothetical protein XP_775631 [Strongylocentrotus purpuratus]
sw03036	BGIBMGA014187	1.1031	1.0777	1.1397	0.4484	0	gi|87248313|gb|ABD36209.1| DnaJ-like protein isoform A [Bombyx mori]
sw01764	BGIBMGA008071	1	0.9724	1.26165	0.4478	1.00E-10	gi|11870000|gb|AAG40582.1| nuclear matrix transcription factor [Rattus norvegicus] Zinc finger protein 384 (Nuclear matrix transcription factor 4) (Cas-associated zinc finger protein)
sw06104	BGIBMGA002096	1	1	1	0.4477	3.00E-85	gi|66511984|ref|XP_392429.2| PREDICTED: similar to protein tyrosine phosphatase [Apis mellifera]
sw14706	BGIBMGA006997	1.00775	0.8661	1.0275	0.44745	Bmb027140	2.00E-11
sw03233	BGIBMGA006063	0.7349	0.9089	0.9792	0.4474	No hits found	
sw17381	BGIBMGA007072	0.63035	0.811	0.57535	0.44685	3.00E-66	gi|86450725|gb|ABC96698.1| yellow5 [Bombyx mori]
sw11286	BGIBMGA007516	0.25745	0.72145	0.7481	0.4466	0	gi|62526112|ref|NP_001014993.1| elongation factor 1-alpha [Apis mellifera] elongation factor-1alpha F2 [Apis mellifera]

sw14855	BGIBMGA006617	1.12115	1.1133	1.28	0.44625	2.00E-39	gi|60678219|gb|AAX33616.1| AT12602p [Drosophila melanogaster]
sw03338	BGIBMGA009555	1	1	1	0.44615	5.00E-14	gi|4972778|gb|AAD34784.1| unknown [Drosophila melanogaster]
sw14778	BGIBMGA000060	0.8216	0.87795	0.8176	0.44615	e-154	gi|76780833|ref|NP_001029121.1| sperm phosphodiesterase 5 [Strongylocentrotus purpuratus] sperm phosphodiesterase 5 [Strongylocentrotus purpuratus]
sw17812	BGIBMGA001313	0.56085	0.85055	0.78745	0.44615	Bmb020198	7.00E-18
sw03136	BGIBMGA002530	1.58365	1	1	0.4461	9.00E-25	gi|78366543|ref|ZP_00836822.1| Peptidyl-dipeptidase A [Shewanella sp. PV-4] Peptidyl-dipeptidase A [Shewanella sp. PV-4]
sw18772	BGIBMGA009946	0.58445	0.70295	1.1005	0.44605	5.00E-55	gi|55241158|gb|EAA08957.2| ENSANGP00000019635 [Anopheles gambiae str. PEST] ENSANGP00000019635 [Anopheles gambiae str. PEST]
sw09199	BGIBMGA000902	1.58635	1.4441	1.22915	0.4457	7.00E-06	gi|66511341|ref|XP_392407.2| PREDICTED: similar to mastermind [Apis mellifera]
sw02423	BGIBMGA008124	0.98405	1.15715	1.04075	0.44565	Bmb040286	2.00E-14
sw03643	BGIBMGA004745	1.3469	1.36415	0.83155	0.44555	5.00E-17	gi|72006355|ref|XP_787711.1| PREDICTED: similar to Preli [Strongylocentrotus purpuratus]
sw14332	BGIBMGA006865	1.32885	1.27605	1.36695	0.44555	7.00E-87	gi|87248303|gb|ABD36204.1| cAMP responsive element binding protein [Bombyx mori]
sw12007	BGIBMGA001190	1	0.9833	1	0.4455	6.00E-13	gi|7542567|gb|AAF63501.1| SP1173 [Drosophila melanogaster]
sw14222	BGIBMGA001607	0.9226	1.00365	1.0844	0.44535	3.00E-11	gi|38677997|emb|CAC83074.1| alpha-1,6-mannosyl-glycoprotein beta-1,2-N-acetylglucosaminyltransferase [Drosophila melanogaster]
sw19227	BGIBMGA009127	0.8953	0.9705	1.07955	0.44535	No hits found	
sw01949	BGIBMGA010566	1.05135	1.27025	1.30115	0.44525	3.00E-96	gi|68440549|ref|XP_708445.1| PREDICTED: similar to prolyl 4-hydroxylase, alpha I subunit isoform 1 precursor isoform 2 [Danio rerio]
sw04231	BGIBMGA001248	0.22045	0.64525	0.75745	0.4448	5.00E-15	gi|89309236|gb|EAS07224.1| Viral A-type inclusion protein repeat containing protein [Tetrahymena thermophila SB210]
sw16364	BGIBMGA011102	1.21405	1.0064	1.2786	0.44465	1.00E-55	gi|158485|gb|AAB04680.1| son of sevenless protein
sw17344	BGIBMGA007565	1.4527	1.35195	1.3651	0.44465	2.00E-47	gi|1226168|gb|AAA92249.1| ORF B (bases 1850-5560) first start codon at 2306
sw01032	BGIBMGA010235	1.1368	0.7347	1.07515	0.4446	0	gi|72083296|gb|AAZ66313.1| RE64894p [Drosophila melanogaster]
sw13109	BGIBMGA004430	1	1	1	0.4446	No hits found	
sw19642	BGIBMGA004728	0.37015	1.3325	0.51565	0.44445	Bmb038818	2.00E-29
sw00401	BGIBMGA004459	1	0.9357	1	0.4443	Bmb005751	No hits found
sw10452	BGIBMGA007223	0.02615	0.58955	0.3962	0.44395	No hits found	
sw13642	BGIBMGA008216	1.0031	1.2047	1.0115	0.4439	8.00E-13	gi|56541238|gb|AAH87598.1| Fumarate hydratase 1 [Rattus norvegicus]
sw15152	BGIBMGA009172	1	1	1	0.4439	1.00E-31	gi|68369754|ref|XP_691790.1| PREDICTED: similar to Low-density lipoprotein receptor-related protein 2 precursor (Megalin) (Glycoprotein 330) (gp330) [Danio rerio]
sw13038	BGIBMGA002339	1.0032	1.04185	0.89665	0.44385	No hits found	
sw19527	BGIBMGA007030	1	1	1	0.4438	e-173	gi|476949|pir||A47371 transcription initiation factor IID 230K chain - fruit fly (Drosophila melanogaster)
sw08906	BGIBMGA012839	0.3503	0.50755	1.0053	0.44325	1.00E-19	gi|52354744|gb|AAH82822.1| Formyltetrahydrofolate dehydrogenase [Xenopus tropicalis] formyltetrahydrofolate dehydrogenase [Xenopus tropicalis] aldehyde dehydrogenase 1 family, member L1 [Xenopus tropicalis]
sw15310	BGIBMGA010786	0.29005	0.5847	0.43845	0.44315	No hits found	

sw20792	BGIBMGA013122	1	1	1	0.4429	1.00E-27	gi|83005715|ref|XP_143000.5| PREDICTED: similar to Zinc finger protein Kr18 (HKr18) [Mus musculus]
sw19900	BGIBMGA006846	1.7273	1	1.49245	0.44275	Bmb034060	3.00E-27
sw10721	BGIBMGA011537	1	1	1	0.4427	3.00E-21	gi|66525753|ref|XP_392953.2| PREDICTED: similar to CG8379-PA, isoform A [Apis mellifera]
sw01339	BGIBMGA009645	0.4418	0.54935	0.83675	0.4424	3.00E-35	gi|85857466|gb|ABC86269.1| RE41071p [Drosophila melanogaster]
sw04333	BGIBMGA007475	1.39445	1.11215	1.2693	0.44235	4.00E-15	gi|29134779|dbj|BAC66140.1| projectin [Procambarus clarkii]
sw05831	BGIBMGA012160	1.08795	0.9636	1.102	0.44235	2.00E-06	gi|21483564|gb|AAM52757.1| SD02552p [Drosophila melanogaster]
sw15165	BGIBMGA012119	0.8777	0.82915	1.1373	0.4421	8.00E-24	gi|83595275|gb|ABC25089.1| integrin linked kinase [Glossina morsitans morsitans]
sw08199	BGIBMGA006857	1.19055	1.3342	1	0.44185	9.00E-08	gi|2117117|emb|CAA69649.1| neurotransmitter transporter [Drosophila melanogaster]
sw02084	BGIBMGA012997	1.09155	0.99	1.034	0.44125	8.00E-69	gi|55243915|gb|EAA06000.2| ENSANGP00000015052 [Anopheles gambiae str. PEST] ENSANGP00000015052 [Anopheles gambiae str. PEST]
sw14650	BGIBMGA004961	0.90735	1.06525	1.0583	0.44085	No hits found	
sw18754	BGIBMGA013513	1	1	1	0.4406	2.00E-42	gi|66803723|gb|AAY56634.1| mitochondrial ribosomal protein S11 [Drosophila simulans]
sw05130	BGIBMGA007946	1.03195	1.07905	1.16515	0.44015	2.00E-60	gi|7858|emb|CAA79313.1| Dpbx [Drosophila melanogaster]
sw08534	BGIBMGA005497	1	1	1	0.43985	4.00E-14	gi|85861125|gb|ABC86511.1| GH15984p [Drosophila melanogaster]
sw00649	BGIBMGA009219	1	1	1	0.4398	4.00E-25	gi|27374257|gb|AAO01014.1| l(2)k09913-PA [Drosophila erecta]
sw03795	BGIBMGA000776	0.70705	0.5776	0.9456	0.4393	Bmb019598	1.00E-66
sw11478	BGIBMGA008579	1.4728	1.3349	1	0.4389	Bmb006092	3.00E-90
sw13917	BGIBMGA000631	1	1	1	0.4385	No hits found	
sw12056	BGIBMGA012240	1.26145	0.955	1.0983	0.4383	3.00E-22	gi|82895058|ref|XP_919288.1| PREDICTED: similar to Basement membrane-specific heparan sulfate proteoglycan core protein precursor (HSPG) (Perlecan) (PLC) [Mus musculus]
sw01890	BGIBMGA013973	1	1.2235	1.5386	0.4382	4.00E-07	gi|55628412|ref|XP_519039.1| PREDICTED: similar to hypothetical protein FLJ22313 [Pan troglodytes]
sw07039	BGIBMGA008873	0.7184	0.9003	0.85165	0.43815	No hits found	
sw12574	BGIBMGA009994	1.0413	1.07585	1.2251	0.4381	4.00E-23	gi|54038195|gb|AAH84280.1| LOC495107 protein [Xenopus laevis]
sw17442	BGIBMGA010072	1.58555	1.3271	1.5659	0.4381	2.00E-52	gi|45382633|ref|NP_990799.1| cortactin [Gallus gallus] Src substrate protein p85 (p80) (Cortactin) p80/85
sw05330	BGIBMGA005717	0.38705	0.60445	0.4684	0.43805	1.00E-48	gi|66545426|ref|XP_392228.2| PREDICTED: similar to dihydropyrimidine amidohydrolase [Apis mellifera]
sw15180	BGIBMGA008314	1	1	1	0.43805	Bmb030779	3.00E-90
sw06384	BGIBMGA004713	1.01445	0.88365	1.08145	0.438	e-125	gi|28316835|gb|AAO39441.1| SD01504p [Drosophila melanogaster]
sw11176	BGIBMGA009911	1.06155	0.89825	1.108	0.43755	0	gi|11874759|dbj|BAB19357.1| ubiquitin activating enzyme [Xenopus laevis]
sw01733	BGIBMGA014009	1	1	1	0.4374	Bmb027059	e-144
sw13453	BGIBMGA012689	1.04135	1.07455	0.95305	0.43735	8.00E-29	gi|62859817|ref|NP_001016691.1| midnolin [Xenopus tropicalis] MGC97762 protein [Xenopus tropicalis] MGC97762 protein [Xenopus tropicalis] midnolin [Xenopus tropicalis]

sw03124	BGIBMGA001611	1.3215	0.84885	0.96485	0.4373	6.00E-08	gi|28850409|gb|AAO53177.1| similar to Dictyostelium discoideum (Slime mold). Adenylyl cyclase
sw12110	BGIBMGA008062	1.3642	1.13055	1.24825	0.4371	e-178	gi|2687733|emb|CAA10950.1| aminopeptidase N [Plutella xylostella]
sw08022	BGIBMGA003803	1.1984	1	1.08545	0.43695	No hits found	
sw03440	BGIBMGA007448	0.6501	0.9582	0.9548	0.4369	4.00E-20	gi|25288036|pir||C88700 protein K02B2.1 [imported] - Caenorhabditis elegans
sw15888	BGIBMGA004729	1.0586	1.06315	1.26525	0.43645	Bmb036205	4.00E-51
sw01148	BGIBMGA003624	1.55595	1	1	0.43615	6.00E-52	gi|55245484|gb|EAA04134.2| ENSANGP00000019058 [Anopheles gambiae str. PEST] ENSANGP00000019058 [Anopheles gambiae str. PEST]
sw18887	BGIBMGA004417	1.11795	1.20635	1.1671	0.43615	2.00E-18	gi|6690788|gb|AAF24343.1| Short stop/Kakapo long isoform [Drosophila melanogaster]
sw13698	BGIBMGA003614	1.2254	1.20025	1.19055	0.4361	1.00E-06	gi|57526849|ref|NP_001009623.1| tumor necrosis factor (ligand) superfamily, member 13 (predicted) [Rattus norvegicus] Tumor necrosis factor (ligand) superfamily, member 13 (predicted) [Rattus norvegicus]
sw15790	BGIBMGA011320	1.28225	1.1205	0.8797	0.4361	3.00E-24	gi|33620734|ref|NP_065924.2| leucine rich repeat neuronal 1 [Homo sapiens] Leucine rich repeat neuronal 1 [Homo sapiens]
sw03621	BGIBMGA011755	0.86805	0.945	1.0076	0.43575	e-124	gi|15082379|gb|AAH12103.1| 3-phosphoinositide dependent protein kinase-1 [Homo sapiens] 3-phosphoinositide dependent protein kinase-1 isoform 1 [Homo sapiens] PDPK1
sw16262	BGIBMGA002789	1.0872	0.8952	1.398	0.4354	1.00E-45	gi|14669808|dbj|BAB33159.2| paxillin [Drosophila melanogaster]
sw08877	BGIBMGA006810	1.15155	1.26175	1.3775	0.435	e-111	gi|38566288|gb|AAH62579.1| CDC2L1 protein [Homo sapiens]
sw04296	BGIBMGA007885	1.09325	0.90625	0.97845	0.4347	1.00E-14	gi|62659557|ref|XP_222780.3| PREDICTED: similar to Ras GTPase-activating protein nGAP (RAS protein activator like 1) [Rattus norvegicus]
sw12277	BGIBMGA011630	1	1	1	0.4347	e-144	gi|27806391|ref|NP_776615.1| supervillin [Bos taurus] supervillin; P205 [Bos taurus]
sw03823	BGIBMGA008162	1	1	1	0.4345	1.00E-14	gi|87248235|gb|ABD36170.1| tetraspanin E [Bombyx mori]
sw06632	BGIBMGA007210	1	1	1	0.43445	1.00E-37	gi|27374353|gb|AAO01094.1| CG4538-PA [Drosophila willistoni]
sw12652	BGIBMGA006161	0.90145	0.90345	1	0.4344	6.00E-26	gi|68438451|ref|XP_700621.1| PREDICTED: similar to Golgi apparatus protein 1 precursor (Golgi sialoglycoprotein MG-160) (E-selectin ligand 1) (ESL-1) (Selel) [Danio rerio]
sw16745	BGIBMGA008226	0.99255	1.099	1.2799	0.43405	Bmb046429	6.00E-25
sw07536	BGIBMGA009237	1.1474	1.0346	1.33305	0.43385	2.00E-07	gi|68365954|ref|XP_690947.1| PREDICTED: similar to cyclin-dependent kinase inhibitor 1B (P27) [Danio rerio]
sw18854	BGIBMGA011348	1	1	1	0.43355	3.00E-06	gi|55629778|ref|XP_527965.1| PREDICTED: similar to polycystic kidney disease 1 like 3; polycystic kidney disease 1-like 3 [Pan troglodytes]
sw15684	BGIBMGA006360	0.85505	1.1681	1.018	0.43345	1.00E-07	gi|27261275|gb|AAN87620.1| circumsporozoite protein [Plasmodium falciparum]
sw10936	BGIBMGA007120	1.12685	1.1308	1.2615	0.43325	8.00E-66	gi|55957148|emb|CAI17931.1| OTTHUMP00000042146 [Homo sapiens] OTTHUMP00000042146 [Homo sapiens] Aconitase 2, precursor [Homo sapiens] aconitase 2 precursor
sw05299	BGIBMGA007034	1.30965	1.20925	1.16205	0.43315	2.00E-08	gi|24649234|ref|NP_651131.2| Inwardly rectifying potassium channel CG6747-PA [Drosophila melanogaster] inwardly rectifying K+ channel protein [Drosophila melanogaster] CG6747-PA [Drosophila melanogaster]
sw18000	BGIBMGA005188	1	1	1	0.43305	4.00E-34	gi|54636303|gb|EAL25706.1| GA14185-PA [Drosophila pseudoobscura]
sw15395	BGIBMGA004932	0.7469	0.95955	1.127	0.4326	5.00E-07	gi|50978924|ref|NP_001003179.1| ribosome receptor [Canis familiaris] Ribosome-binding protein 1 (180 kDa ribosome receptor) (RRp) ribosome receptor [Canis familiaris]
sw21301	BGIBMGA000529	1.3782	0.87595	0.9376	0.43245	e-150	gi|13359138|dbj|BAB33300.1| neutral endopeptidase 24.11 [Bombyx mori]
sw00705	BGIBMGA000461	1.0795	1.04365	1.10715	0.4324	No hits found	
sw05298	BGIBMGA000103	1.1214	1.1881	1.14085	0.4324	2.00E-44	gi|46249989|gb|AAH68436.1| Heterogeneous nuclear ribonucleoprotein U-like 1 [Danio rerio] heterogeneous nuclear ribonucleoprotein U-like 1 [Danio rerio]

sw13915	BGIBMGA001475	1.014	1.05495	1.2454	0.43185	No hits found	
sw02654	BGIBMGA011259	1	1	0.91875	0.4318	5.00E-14	gi|34328063|ref|NP_058672.1| glutamate receptor, metabotropic 1 [Mus musculus] metabotropic glutamate receptor type 1 [Mus musculus] Metabotropic glutamate receptor 1 precursor (mGluR1)
sw12063	BGIBMGA003318	0.9395	0.8735	0.8275	0.4318	Bmb009923	9.00E-07
sw15458	BGIBMGA011631	1	1	1	0.4317	2.00E-67	gi|68430827|ref|XP_685621.1| PREDICTED: similar to ubiquitin specific protease 10 isoform 1 [Danio rerio]
sw19077	BGIBMGA007792	1	1	1	0.43155	6.00E-13	gi|474339|gb|AAA17752.1| reverse transcriptase [Bombyx mori]
sw10857	BGIBMGA001886	1	1	1	0.43125	7.00E-17	gi|76647578|ref|XP_588089.2| PREDICTED: similar to gamma tubulin ring complex protein (76p gene) [Bos taurus]
sw06801	BGIBMGA007288	1	1.0659	1	0.43095	Bmb035016	2.00E-40
sw09789	BGIBMGA012228	1	1	1	0.4308	4.00E-79	gi|68364958|ref|XP_683834.1| PREDICTED: similar to IQ motif containing with AAA domain isoform 1 [Danio rerio]
sw07504	BGIBMGA005299	1.44575	1.09975	0.7382	0.43065	5.00E-07	gi|15146352|dbj|BAB33387.2| XP8 [Xenopus laevis]
sw20448	BGIBMGA010298	1.0104	0.9638	0.8912	0.4306	4.00E-54	gi|4521269|dbj|BAA76304.1| endonuclease and reverse transcriptase-like protein [Bombyx mori]
sw03476	BGIBMGA005305	0.93165	0.9843	1.0086	0.4302	9.00E-06	gi|66518870|ref|XP_391883.2| PREDICTED: similar to zinc finger protein 236 [Apis mellifera]
sw12506	BGIBMGA005624	1	1	1	0.42995	No hits found	
sw17635	BGIBMGA000025	0.947	1.1011	0.88715	0.4299	No hits found	
sw08966	BGIBMGA009401	1	1	1	0.42975	No hits found	
sw02214	BGIBMGA012743	1	1	1	0.42965	Bmb035678	7.00E-40
sw04688	BGIBMGA009254	1	1	1	0.42965	Bmb042445	2.00E-19
sw18597	BGIBMGA011424	0.81125	0.62405	0.53695	0.42965	0	gi|136206|sp|P22297|TRF_MANSE Transferrin precursor transferrin
sw00514	BGIBMGA013343	1	1	1	0.42955	Bmb007345	6.00E-31
sw11487	BGIBMGA010637	0.8984	0.9238	0.86535	0.42945	4.00E-77	gi|5410429|gb|AAD43053.1| Recq helicase 5 [Drosophila melanogaster]
sw17432	BGIBMGA001402	1.21845	1.12715	1.307	0.42915	Bmb024182	9.00E-23
sw13610	BGIBMGA013851	1	1	1	0.4291	6.00E-07	gi|30696202|ref|NP_176103.2| ATP binding / ATP-dependent helicase/ helicase/ nucleic acid binding [Arabidopsis thaliana]
sw22041	BGIBMGA001781	1	0.9066	0.65205	0.4291	7.00E-13	gi|474339|gb|AAA17752.1| reverse transcriptase [Bombyx mori]
sw01716	BGIBMGA010554	1	1	1	0.42895	No hits found	
sw22759	BGIBMGA004610	0.99045	0.70665	0.86175	0.42895	No hits found	
sw15773	BGIBMGA004500	1.1694	1.19795	1.2427	0.42885	Bmb035374	2.00E-71
sw18575	BGIBMGA006415	1.20835	0.99925	0.9803	0.42875	0	gi|54401523|gb|AAV34566.1| chorion b-ZIP transcription factor [Bombyx mori]
sw19295	BGIBMGA003655	1.3887	1.57885	1.3436	0.42845	e-160	gi|66515547|ref|XP_394781.2| PREDICTED: similar to rTS beta protein isoform 2 [Apis mellifera]
sw04457	BGIBMGA013226	1.1865	1.0277	1.155	0.42815	3.00E-15	gi|52076246|dbj|BAD45014.1| egalitarian-like [Oryza sativa (japonica cultivar-group)]

sw05764	BGIBMGA001666	1	1	1	0.428	No hits found	
sw21639	BGIBMGA007415	0.94845	1.2438	1.33235	0.42795	5.00E-07	gi|3860051|gb|AAC72967.1| transcytosis associated protein p115 [Mus musculus]
sw17331	BGIBMGA006179	1.5174	1.17685	1.49015	0.4277	3.00E-06	gi|54645551|gb|EAL34289.1| GA10719-PA [Drosophila pseudoobscura]
sw04173	BGIBMGA008687	1	1	1	0.42715	3.00E-05	gi|66516317|ref|XP_392552.2| PREDICTED: similar to CG14307-PB, isoform B [Apis mellifera]
sw05614	BGIBMGA013995	1	1	1	0.42695	0	gi|29343830|gb|AAO81591.1| glycosyl hydrolase, family 31/fibronectin type III domain protein [Enterococcus faecalis V583] glycosyl hydrolase, family 31/fibronectin type III domain protein [Enterococcus faecalis V583]
sw13861	BGIBMGA011183	1.0025	1.02645	1.08985	0.42685	Bmb021215	No hits found
sw21531	BGIBMGA005523	1.08395	1.0056	1.02625	0.4268	No hits found	
sw14740	BGIBMGA012136	1.2669	1.11485	1.26335	0.42655	7.00E-58	gi|47086073|ref|NP_998413.1| ADP-ribosylation factor-like 7 [Danio rerio] ADP-ribosylation factor-like 7 [Danio rerio]
sw04125	BGIBMGA007784	1	1	1	0.4265	Bmb027120	4.00E-35
sw06036	BGIBMGA005475	0.98605	0.6934	0.89445	0.4264	5.00E-21	gi|72108948|ref|XP_800584.1| PREDICTED: hypothetical protein XP_795491 [Strongylocentrotus purpuratus]
sw20229	BGIBMGA000760	1	1	1	0.4262	0	gi|73953679|ref|XP_536399.2| PREDICTED: similar to polymerase (RNA) III (DNA directed) polypeptide A, 155kDa [Canis familiaris]
sw19878	BGIBMGA005522	1	1	1	0.42595	0	gi|6491999|gb|AAF14115.1| SPT6 protein [Drosophila melanogaster]
sw20117	BGIBMGA011625	0.6158	0.98855	0.7981	0.42585	6.00E-33	gi|28569894|dbj|BAC57926.1| reverse transcriptase [Bombyx mori]
sw05034	BGIBMGA012309	0.7929	1.0589	0.9183	0.4252	2.00E-38	gi|66803829|gb|AAY56642.1| unknown [Drosophila simulans]
sw18837	BGIBMGA005925	1.17905	1.02825	1.06705	0.4252	Bmb012364	1.00E-55
sw14827	BGIBMGA010711	1	1	1	0.42475	Bmb028066	1.00E-10
sw01328	BGIBMGA005268	1	1	1.2919	0.42465	No hits found	
sw16327	BGIBMGA002977	1	1	1	0.4244	Bmb040403	3.00E-09
sw13503	BGIBMGA014181	1.2032	1.14815	1.33615	0.4241	1.00E-47	gi|47522760|ref|NP_999131.1| long-chain 3-ketoacyl-CoA thiolase [Sus scrofa] long-chain 3-ketoacyl-CoA thiolase [Sus scrofa]
sw13202	BGIBMGA010004	1	1	1	0.42395	No hits found	
sw00781	BGIBMGA005536	1	1.0928	0.9943	0.42315	Bmb011411	0
sw02532	BGIBMGA009783	1.02155	0.9996	0.97655	0.42315	Bmb043166	No hits found
sw21215	BGIBMGA013894	1	1	1	0.42315	Bmb030178	3.00E-05
sw11197	BGIBMGA009156	1.14525	1.00925	1.12825	0.4227	Bmb004266	e-119
sw06889	BGIBMGA004301	1	1	1	0.4226	2.00E-22	gi|53766623|gb|AAU93471.1| ubiquitin ligase [Mus musculus] ring finger protein 123 [Mus musculus]
sw07468	BGIBMGA003020	1	1	1	0.4225	3.00E-90	gi|66519954|ref|XP_392911.2| PREDICTED: similar to CG2950-PB, isoform B [Apis mellifera]
sw19088	BGIBMGA007869	0.84605	1.06885	1.2732	0.4225	Bmb015999	e-124
sw20009	BGIBMGA010482	0.2766	0.7087	0.47965	0.42215	No hits found	

sw14947	BGIBMGA007604	1	1	1	0.42135	1.00E-08	gi|60677997|gb|AAX33505.1| LP14866p [Drosophila melanogaster]
sw19450	BGIBMGA007218	1.5766	1.828	1.60905	0.42085	Bmb037633	2.00E-67
sw06417	BGIBMGA001346	1	1	1	0.4207	4.00E-05	gi|25009850|gb|AAN71095.1| AT22221p [Drosophila melanogaster]
sw08876	BGIBMGA010177	1.25275	1.13295	1.1088	0.4207	Bmb020896	6.00E-14
sw05656	BGIBMGA013001	1	1	1	0.4205	0	gi|66499229|ref|XP_624835.1| PREDICTED: similar to CG9514-PA [Apis mellifera]
sw02098	BGIBMGA003532	1	1	1	0.42015	6.00E-16	gi|62663165|ref|XP_214420.3| PREDICTED: similar to nuclear ATP/GTP-binding protein [Rattus norvegicus]
sw11791	BGIBMGA006898	1.0213	0.9283	0.8165	0.42005	No hits found	
sw02234	BGIBMGA008858	1	1	1	0.4199	Bmb036249	8.00E-82
sw19332	BGIBMGA000508	1	1	1	0.4198	Bmb030748	7.00E-14
sw20506	BGIBMGA006491	1.0369	1.28255	1.03985	0.4195	6.00E-67	gi|37359874|dbj|BAC97915.1| mKIAA0261 protein [Mus musculus]
sw01081	BGIBMGA002821	1	1	1	0.41935	Bmb015839	7.00E-58
sw15708	BGIBMGA000864	1	1	1	0.41935	Bmb034920	2.00E-37
sw05903	BGIBMGA000387	1.1186	1.2067	1.48055	0.4192	2.00E-05	gi|27316716|gb|AAO05891.1| streptococcal hemagglutinin protein [Staphylococcus epidermidis ATCC 12228] streptococcal hemagglutinin protein [Staphylococcus epidermidis ATCC 12228]
sw00084	BGIBMGA009828	1	1	1	0.41905	2.00E-11	gi|49522839|gb|AAH73969.1| NEFH protein [Homo sapiens] neurofilament protein [Homo sapiens] Neurofilament triplet H protein (200 kDa neurofilament protein) (Neurofilament heavy polypeptide) (NF-H)
sw17919	BGIBMGA013623	0.85475	0.8692	1.0252	0.41885	3.00E-18	gi|73959079|ref|XP_864654.1| PREDICTED: similar to transcription elongation factor B (SIII), polypeptide 2 isoform 3 [Canis familiaris]
sw18960	BGIBMGA011754	1.1258	1.06705	1.1043	0.41885	e-165	gi|66524404|ref|XP_623193.1| PREDICTED: similar to DEAD (Asp-Glu-Ala-Asp) box polypeptide 5, partial [Apis mellifera]
sw16993	BGIBMGA011319	1.385	1.52995	1.3036	0.41875	5.00E-30	gi|73976657|ref|XP_539546.2| PREDICTED: similar to aminoadipate-semialdehyde synthase [Canis familiaris]
sw00820	BGIBMGA013327	0.8194	1.2256	1.5914	0.41865	2.00E-14	gi|72180773|ref|XP_798850.1| PREDICTED: similar to Staphylococcal nuclease domain containing protein 1 (p100 co-activator) (100 kDa coactivator) (4SNc-Tudor domain protein) [Strongylocentrotus purpuratus]
sw12892	BGIBMGA013088	1	1	1	0.41855	2.00E-09	gi|73966952|ref|XP_548257.2| PREDICTED: similar to growth arrest-specific 2 like 2 [Canis familiaris]
sw15682	BGIBMGA000703	1.15705	0.8534	1.16855	0.4183	5.00E-98	gi|89272476|emb|CAJ83048.1| procollagen-lysine, 2-oxoglutarate 5-dioxygenase 3 [Xenopus tropicalis]
sw17349	BGIBMGA001707	0.91605	0.9006	1.1551	0.4178	0	gi|2613141|gb|AAB84297.1| beta-1 tubulin [Manduca sexta] Tubulin beta-1 chain (Beta-1 tubulin)
sw13693	BGIBMGA000939	1	1	1	0.41765	5.00E-21	gi|25009692|gb|AAN71022.1| AT04406p [Drosophila melanogaster]
sw09883	BGIBMGA003393	1	1	1	0.4175	Bmb037812	2.00E-12
sw20669	BGIBMGA011222	1	1	1	0.41725	2.00E-18	gi|24586460|ref|NP_724636.1| CG11198-PB, isoform B [Drosophila melanogaster] GH12002p [Drosophila melanogaster] CG11198-PB, isoform B [Drosophila melanogaster]
sw21348	BGIBMGA011032	1	1.0171	1	0.41715	No hits found	
sw14007	BGIBMGA008169	0.87085	1.10905	1.0868	0.4164	7.00E-23	gi|72004452|ref|XP_779950.1| PREDICTED: similar to RN protein [Strongylocentrotus purpuratus]
sw22896	BGIBMGA007727	0.97935	0.82025	0.8806	0.41615	7.00E-49	gi|9992882|gb|AAG11414.1| nicastrin [Drosophila melanogaster]
sw00397	BGIBMGA002165	1	1	1	0.4161	2.00E-08	gi|66516917|ref|XP_391828.2| PREDICTED: similar to CG8312-PA, isoform A [Apis mellifera]

sw21135	BGIBMGA005745	1.0065	1.07295	0.99225	0.41515	Bmb025037	6.00E-38
sw04421	BGIBMGA008701	1	1	1	0.4151	No hits found	
sw10912	BGIBMGA007558	1.293	0.8929	1.14315	0.4149	4.00E-75	gi|74011918|ref|XP_848765.1| PREDICTED: similar to inter-alpha (globulin) inhibitor H4 (plasma Kallikrein-sensitive glycoprotein) [Canis familiaris]
sw18920	BGIBMGA013723	1	1	1	0.41455	9.00E-41	gi|83415184|ref|NP_787057.2| ARG99 protein [Homo sapiens]
sw13033	BGIBMGA000536	1	1	1	0.4142	No hits found	
sw19113	BGIBMGA013161	0.932	1.04925	1.02065	0.4139	2.00E-63	gi|7862150|gb|AAF70499.1| 3-dehydroecdysone 3alpha-reductase [Spodoptera littoralis]
sw22265	BGIBMGA012100	0.95345	0.9475	1	0.41355	2.00E-19	gi|56418289|gb|AAV91103.1| unknown protein [Grouper iridovirus]
sw20921	BGIBMGA003644	0.9631	1.1688	0.8609	0.41345	1.00E-33	gi|68394337|ref|XP_696675.1| PREDICTED: similar to Sorting nexin 17 [Danio rerio]
sw00374	BGIBMGA007028	1	1	1	0.41335	No hits found	
sw20984	BGIBMGA003288	0.9706	0.98345	1.1387	0.4128	No hits found	
sw00249	BGIBMGA007948	0.8155	0.8818	0.87985	0.41265	0	gi|82799714|ref|XP_929387.1| PREDICTED: similar to Spectrin alpha chain, brain (Spectrin, non-erythroid alpha chain) (Alpha-II spectrin) (Fodrin alpha chain) isoform
sw17817	BGIBMGA009635	0.0964	0.64495	0.6667	0.4122	No hits found	
sw20046	BGIBMGA003008	1	1	1	0.41215	0	gi|73949889|ref|XP_535315.2| PREDICTED: similar to Phosphorylase b kinase beta regulatory chain (Phosphorylase kinase beta subunit) isoform 1 [Canis familiaris]
sw22790	BGIBMGA002170	0.91875	0.82795	0.72375	0.4117	5.00E-11	gi|57163725|ref|NP_001009188.1| cauxin [Felis catus] carboxylesterase-like urinary excreted protein [Felis catus]
sw12437	BGIBMGA002767	1.1991	1.01905	1.23065	0.41135	4.00E-36	gi|89039345|gb|ABD60146.1| adipokinetic hormone receptor [Anopheles gambiae]
sw14479	BGIBMGA009409	0.9923	0.8632	1.1682	0.41135	Bmb025627	9.00E-99
sw09646	BGIBMGA005102	1	1	1	0.4113	e-140	gi|562793|emb|CAA57539.1| kinesin-like protein 1 [Xenopus laevis] Chromosome-associated kinesin KLP1 (Chromokinesin)
sw10757	BGIBMGA001512	1	1	1	0.41115	5.00E-80	gi|28557643|gb|AAO45227.1| LD28216p [Drosophila melanogaster]
sw09844	BGIBMGA013565	1	1	1	0.4109	Bmb037037	1.00E-09
sw17529	BGIBMGA007519	1.94665	1.8354	2.41355	0.4104	Bmb046033	5.00E-56
sw02168	BGIBMGA003145	1	1	1	0.4103	No hits found	
sw16234	BGIBMGA013799	1	1	1	0.40985	Bmb039414	1.00E-06
sw11241	BGIBMGA009731	1.0442	1.04215	1.19	0.40955	No hits found	
sw03799	BGIBMGA011489	1	1	0.86495	0.40885	2.00E-13	gi|88174321|gb|ABD39318.1| monocarboxylate transporter 2 [Bos taurus]
sw09134	BGIBMGA012483	1.2189	0.9463	1.1453	0.40885	2.00E-74	gi|50745792|ref|XP_420245.1| PREDICTED: similar to synaptotagmin-like 4 (granuphilin-a); granuphilin-a [Gallus gallus]
sw13141	BGIBMGA011983	0.7603	1.105	1.2581	0.40885	4.00E-44	gi|39595413|emb|CAE60451.1| Hypothetical protein CBG04059 [Caenorhabditis briggsae]
sw21110	BGIBMGA005199	0.95595	1.08785	1	0.40885	Bmb023509	e-175
sw01913	BGIBMGA003021	1.1366	0.8014	0.9879	0.4085	1.00E-30	gi|8855|emb|CAA30332.1| unnamed protein product [Drosophila melanogaster]

sw05562	BGIBMGA000455	1.33245	1.2124	1.64355	0.40825	No hits found	
sw15120	BGIBMGA002346	1.17395	1.14405	1.26515	0.4081	Bmb030342	e-176
sw12404	BGIBMGA014126	1.1069	0.98915	1.11075	0.40805	4.00E-16	gi|73988285|ref|XP_534038.2| PREDICTED: similar to tripartite motif-containing 3 isoform 1 [Canis familiaris]
sw13634	BGIBMGA008170	1	1	1	0.40765	2.00E-38	gi|45552683|ref|NP_995866.1| CG15609-PB, isoform B [Drosophila melanogaster] CG15609-PC, isoform C [Drosophila melanogaster] CG15609-PA, isoform A [Drosophila melanogaster] CG15609-PC, isoform C
sw01118	BGIBMGA013531	1.4801	1.11595	1.53265	0.4074	2.00E-56	gi|72065732|ref|XP_798089.1| PREDICTED: similar to Myosin light chain kinase, smooth muscle and non-muscle isozymes (MLCK) [Strongylocentrotus purpuratus]
sw07891	BGIBMGA011611	1	0.7623	1.2143	0.40695	1.00E-25	gi|30851445|gb|AAH52460.1| Activating transcription factor 7 interacting protein [Mus musculus] activating transcription factor 7 interacting protein [Mus musculus] Atf7ip protein [Mus musculus]
sw04003	BGIBMGA003015	1	1	1	0.4069	9.00E-80	gi|66519690|ref|XP_393825.2| PREDICTED: similar to ENSANGP00000018249 [Apis mellifera]
sw16308	BGIBMGA009674	1.7167	1.18	1.1083	0.4065	Bmb040261	4.00E-26
sw08046	BGIBMGA003752	1.02055	1.11205	1.12855	0.40635	4.00E-58	gi|71834261|gb|AAZ41803.1| GH05001p [Drosophila melanogaster]
sw15582	BGIBMGA008548	1.36985	1.1216	1	0.40635	2.00E-36	gi|66524943|ref|XP_394073.2| PREDICTED: similar to ENSANGP00000021271 [Apis mellifera]
sw15666	BGIBMGA006921	1.02995	0.93995	1.50955	0.40615	No hits found	
sw05916	BGIBMGA007041	1	1	1	0.4061	8.00E-11	gi|5834394|gb|AAD53951.1| sulfate transporter [Drosophila melanogaster]
sw15430	BGIBMGA003464	1	1	1	0.40595	2.00E-89	gi|74002920|ref|XP_545139.2| PREDICTED: similar to solute carrier family 12, member 8 [Canis familiaris]
sw17421	BGIBMGA006461	1.0303	1.01985	1.1226	0.4058	8.00E-52	gi|66564370|ref|XP_395279.2| PREDICTED: similar to GA14079-PA [Apis mellifera]
sw07693	BGIBMGA003939	1	1	1	0.4057	9.00E-35	gi|32997078|dbj|BAC79385.1| transcription factor BmEts [Bombyx mori]
sw14240	BGIBMGA003515	1.24425	1.13395	1.15845	0.4056	1.00E-38	gi|28317136|gb|AAD27865.2| LD24380p [Drosophila melanogaster]
sw18541	BGIBMGA006999	1	1	1	0.4056	Bmb040506	e-137
sw22022	BGIBMGA013519	1	1	1	0.4056	No hits found	
sw04273	BGIBMGA003831	1	1	1	0.4055	4.00E-32	gi|25072201|ref|NP_739563.1| oocyte-testis gene 1 [Mus musculus] Protein C10orf118 homolog (Oocyte-testis gene 1 protein) oocyte-testis gene 1 [Mus musculus]
sw08691	BGIBMGA001608	0.9081	1.05465	1.0573	0.40525	5.00E-36	gi|38677997|emb|CAC83074.1| alpha-1,6-mannosyl-glycoprotein beta-1,2-N-acetylglucosaminyltransferase [Drosophila melanogaster]
sw03623	BGIBMGA001237	0.93125	0.825	0.8514	0.40505	2.00E-39	gi|2342862|gb|AAB67672.1| branched chain aminotransferase precursor [Homo sapiens]
sw09354	BGIBMGA011542	1	1	1	0.4049	1.00E-60	gi|57236096|gb|AAW48725.1| dicer-2 [Aedes aegypti]
sw09068	BGIBMGA008554	0.9827	1.05085	1.0619	0.40485	2.00E-12	gi|50756101|ref|XP_415017.1| PREDICTED: similar to chromosome 20 open reading frame 72 [Gallus gallus]
sw00459	BGIBMGA009057	0.86885	0.8464	1.2403	0.40475	2.00E-85	gi|55644901|ref|XP_523558.1| PREDICTED: solute carrier family 25 (mitochondrial carrier; oxoglutarate carrier), member 11 [Pan troglodytes] solute carrier family 25 (mitochondrial carrier; oxoglutarate carrier), member 11 [Homo sapiens] Sol
sw13599	BGIBMGA007388	1	1	1	0.40395	3.00E-15	gi|28381027|gb|AAO41480.1| AT31792p [Drosophila melanogaster]
sw09253	BGIBMGA000504	0.1725	0.6545	0.7512	0.40385	9.00E-22	gi|72147038|ref|XP_794932.1| PREDICTED: similar to putative protein, with at least 6 transmembrane domains (53.0 kD) (XK70) [Strongylocentrotus purpuratus]
sw00359	BGIBMGA003481	1.21045	0.9945	1.179	0.40375	0	gi|17298115|dbj|BAB78526.1| wing disc-specific protein [Bombyx mori]
sw15106	BGIBMGA000146	0.9798	1.0924	1.1015	0.4037	9.00E-60	gi|54636311|gb|EAL25714.1| GA10531-PA [Drosophila pseudoobscura]

sw12091	BGIBMGA005747	1	1	1	0.4036	9.00E-96	gi|66505993|ref|XP_396307.2| PREDICTED: similar to GA16078-PA, partial [Apis mellifera]
sw12938	BGIBMGA008450	0.307	0.64765	0.4723	0.40315	No hits found	
sw10846	BGIBMGA011426	0.9065	0.9104	1.0221	0.40295	2.00E-12	gi|1877189|emb|CAA71853.1| mitochondrial protein [Drosophila melanogaster]
sw06982	BGIBMGA008730	1	1	1	0.4027	Bmb039305	No hits found
sw18019	BGIBMGA000976	1.1071	1.1556	1.14425	0.4025	Bmb023439	8.00E-17
sw01636	BGIBMGA008706	1.05915	0.98755	0.9317	0.40185	8.00E-07	gi|1125764|gb|AAA83588.1| Uncoordinated protein 78, isoform a [Caenorhabditis elegans] UNCoordinated family member (unc-78) [Caenorhabditis elegans] UNC-78 [Caenorhabditis elegans] Actin-interacting protein 1 (AIP1)
sw01139	BGIBMGA011703	1.1139	0.9233	0.9396	0.40165	1.00E-68	gi|87248125|gb|ABD36115.1| EN protein binding/engrailed nuclear homeoprotein-regulated protein [Bombyx mori]
sw12143	BGIBMGA003555	0.9772	0.9605	1.0779	0.4012	e-117	gi|49904239|gb|AAH76960.1| MGC89389 protein [Xenopus tropicalis] MGC89389 protein [Xenopus tropicalis]
sw07452	BGIBMGA012764	2.10635	1.62895	2.05445	0.40115	6.00E-55	gi|46396762|sp|Q27451|PRP1_BOMMO Phenoloxidase subunit 1 precursor (Tyrosinase 1) (PO 1) prophenoloxidase subunit 1 [Bombyx mori]
sw01082	BGIBMGA000691	0.7228	0.5409	0.8891	0.401	5.00E-33	gi|7106224|gb|AAF36090.1| flagelliform silk protein [Nephila clavipes]
sw12257	BGIBMGA008927	1.03015	0.93795	0.85505	0.4008	e-112	gi|74005720|ref|XP_536066.2| PREDICTED: similar to actin related protein 2/3 complex subunit 2 [Canis familiaris]
sw16701	BGIBMGA004029	1	1	1	0.40055	Bmb045710	2.00E-28
sw20918	BGIBMGA002709	1	0.9609	1.1617	0.4005	8.00E-37	gi|56269147|gb|AAH87370.1| LOC495984 protein [Xenopus laevis]
sw01814	BGIBMGA006603	1	1	1	0.4001	Bmb028324	0
sw14404	BGIBMGA002393	0.988	1.0235	1.22195	0.3997	1.00E-92	gi|6523177|emb|CAB62169.1| ARALAR 1 protein [Drosophila melanogaster]
sw15323	BGIBMGA004517	0.42785	0.8152	1	0.3995	2.00E-09	gi|72094483|ref|XP_792984.1| PREDICTED: similar to ACRC protein [Strongylocentrotus purpuratus]
sw21273	BGIBMGA009503	1	1	1	0.39945	e-113	gi|25009861|gb|AAN71100.1| AT23409p [Drosophila melanogaster]
sw10003	BGIBMGA012921	1	1	1	0.3991	Bmb040113	1.00E-46
sw08368	BGIBMGA002435	1.05155	1.04585	0.93535	0.39875	No hits found	
sw20163	BGIBMGA010338	1.313	1.42945	1.30385	0.3987	No hits found	
sw12788	BGIBMGA000145	1	1	1	0.3986	1.00E-18	gi|24655499|ref|NP_725862.1| CG10737-PA, isoform A [Drosophila melanogaster] CG10737-PA, isoform A [Drosophila melanogaster]
sw06810	BGIBMGA008146	0.9264	0.44585	0.4733	0.398	Bmb035169	2.00E-36
sw12413	BGIBMGA007032	1	1	1	0.39795	2.00E-06	gi|19921638|ref|NP_610120.1| General receptor for phosphoinositides 1 CG11628-PA [Drosophila melanogaster] RE34385p [Drosophila melanogaster] CG11628-PA [Drosophila melanogaster]
sw14428	BGIBMGA004018	1	1	1	0.39745	e-116	gi|11493775|gb|AAG35645.1| cyclin-dependent protein kinase 5 [Danio rerio] cyclin-dependent protein kinase 5 [Danio rerio]
sw09128	BGIBMGA002432	1.4285	1.12075	1.47905	0.39725	4.00E-94	gi|6815281|gb|AAF28475.1| V-ATPase 110 kDa integral membrane subunit [Aedes aegypti]
sw08102	BGIBMGA003647	1.11515	0.8406	1.1316	0.3966	8.00E-31	gi|1022831|gb|AAA79851.1| tyrosine kinase
sw10606	BGIBMGA012964	0.9896	0.906	1.2198	0.39625	e-117	gi|66535977|ref|XP_624437.1| PREDICTED: similar to Nardilysin precursor (N-arginine dibasic convertase) (NRD convertase) (NRD-C) [Apis mellifera]
sw09237	BGIBMGA000634	1	1	1	0.39615	No hits found	

sw15072	BGIBMGA002517	1	1	1	0.39615	No hits found		
sw09525	BGIBMGA007317	0.02855	0.56055	0.61545	0.3958		7.00E-88	gi|66520408|ref|XP_392848.2| PREDICTED: similar to Ubiquitin carboxyl-terminal hydrolase 7 (Ubiquitin thiolesterase 7) (Ubiquitin-specific processing protease 7) (Deubiquitinating enzyme 7) (Herpesvirus associated ubiquitin-specific proteas
sw02952	BGIBMGA006809	1.0099	0.94895	1.0096	0.3952	Bmb001061	6.00E-53	
sw21115	BGIBMGA005435	0.9213	1.1153	1.0446	0.3939		3.00E-31	gi|57506562|dbj|BAD86652.1| reverse transcriptase [Bombyx mori]
sw08191	BGIBMGA008857	1.2245	0.93105	1.22435	0.39345		5.00E-17	gi|58585102|ref|NP_001011581.1| cGMP-dependent protein kinase foraging [Apis mellifera] cGMP-dependent protein kinase foraging [Apis mellifera]
sw12272	BGIBMGA007791	0.0951	0.7013	1	0.3932		9.00E-05	gi|82704981|ref|XP_726778.1| chloroquine resistance marker protein [Plasmodium yoelii yoelii str. 17XNL] chloroquine resistance marker protein, putative [Plasmodium yoelii yoelii]
sw10247	BGIBMGA013007	1	1.0274	1.3095	0.39305	Bmb047837	2.00E-28	
sw05877	BGIBMGA011979	0.94745	0.96425	1.0062	0.39285	Bmb016516	7.00E-67	
sw06023	BGIBMGA007077	0.8272	1.05635	1.01395	0.3922	No hits found		
sw18955	BGIBMGA010022	0.1789	0.31565	0.07885	0.3918		7.00E-88	gi|415798|emb|CAA81643.1| blastopia polyprotein [Drosophila melanogaster]
sw17998	BGIBMGA003613	1.5973	1.3666	1.2492	0.39165	Bmb020186	No hits found	
sw03948	BGIBMGA008363	1	1	1	0.3912		7.00E-17	gi|70571741|dbj|BAE06812.1| zinc finger protein [Ciona intestinalis]
sw02398	BGIBMGA013707	1.11	0.9797	1.2912	0.3907	No hits found		
sw16826	BGIBMGA010300	1	1	1	0.3903	Bmb047820	No hits found	
sw03733	BGIBMGA008849	1.08825	1.03415	1.1158	0.3902		1.00E-14	gi|68441657|ref|XP_698520.1| PREDICTED: similar to G protein-coupled receptor 158 [Danio rerio]
sw19848	BGIBMGA002375	0.97145	1.02925	1.1207	0.38995	Bmb026314	5.00E-55	
sw07506	BGIBMGA005300	2.24045	0.82805	0.65335	0.38955		7.00E-71	gi|121150|sp|P07314|GGT1_RAT Gamma-glutamyltranspeptidase 1 precursor (Gamma-glutamyltransferase 1) (GGT 1) (CD224 antigen) [Contains: Gamma-glutamyltranspeptidase 1 heavy chain; Gamma-glutamyltranspeptidase 1 light chain
sw08808	BGIBMGA005404	0.0488	0.4103	0.363	0.389	No hits found		
sw15751	BGIBMGA009148	1.3972	1.36215	1.2607	0.38895		5.00E-18	gi|55237956|gb|EAA12905.3| ENSANGP00000002431 [Anopheles gambiae str. PEST] ENSANGP00000002431 [Anopheles gambiae str. PEST]
sw09672	BGIBMGA009282	0.98885	0.9689	1.0581	0.38835	Bmb033756	2.00E-22	
sw05837	BGIBMGA000699	1.02245	0.8858	0.8784	0.38735		2.00E-35	gi|38638820|gb|AAR25648.1| Abnormal cell migration protein 10, isoform c [Caenorhabditis elegans] abnormal cell MIGration family member (mig-10) [Caenorhabditis elegans] Abnormal cell migration protein 10
sw15402	BGIBMGA009090	1	1	1	0.38695	No hits found		
sw13978	BGIBMGA011687	1.06485	1.15235	1.1382	0.38595		2.00E-75	gi|68388015|ref|XP_692539.1| PREDICTED: similar to leukocyte receptor cluster (LRC) member 8 [Danio rerio]
sw19096	BGIBMGA001352	0.9658	1.09675	1.1177	0.38595		1.00E-47	gi|16758758|ref|NP_446338.1| lectin, mannose-binding, 1 [Rattus norvegicus] ERGIC-53 protein precursor (ER-Golgi intermediate compartment 53 kDa protein) (Lectin, mannose-binding 1) (p58) p58 cis-Golgi/intermediate compartment prote
sw09062	BGIBMGA000621	1	1	1	0.38545		2.00E-64	gi|3043692|dbj|BAA25510.1| KIAA0584 protein [Homo sapiens]
sw09797	BGIBMGA000710	1	1	1	0.38525		2.00E-30	gi|48138819|ref|XP_393426.1| PREDICTED: similar to vesicle-associated membrane protein, associated protein B and C [Apis mellifera]
sw16651	BGIBMGA003704	1.0482	1.12445	1.042	0.38465	Bmb044821	No hits found	
sw19010	BGIBMGA004010	1	1	1	0.3838		2.00E-96	gi|2058458|gb|AAB53257.1| p260 [Bombyx mori]

<table>
<tr><td>sw13989</td><td>BGIBMGA002847</td><td>1</td><td>1</td><td>1</td><td>0.3829</td><td>Bmb022061</td><td>4.00E-21</td></tr>
<tr><td>sw05946</td><td>BGIBMGA006585</td><td>1</td><td>1</td><td>1</td><td>0.38265</td><td>6.00E-14</td><td>gi|25009879|gb|AAN71108.1| AT27059p [Drosophila melanogaster]</td></tr>
<tr><td>sw19815</td><td>BGIBMGA009693</td><td>0.49995</td><td>0.68335</td><td>0.6096</td><td>0.38235</td><td>5.00E-23</td><td>gi|73965363|ref|XP_548034.2| PREDICTED: similar to Angiotensin-converting enzyme, testis-specific isoform precursor (ACE-T) (Dipeptidyl carboxypeptidase I) (Kininase II) [Canis familiaris]</td></tr>
<tr><td>sw00306</td><td>BGIBMGA014211</td><td>1.06055</td><td>0.95085</td><td>1.2978</td><td>0.38225</td><td>e-175</td><td>gi|87248239|gb|ABD36172.1| transketolase [Bombyx mori]</td></tr>
<tr><td>sw20350</td><td>BGIBMGA007121</td><td>0.92185</td><td>1.0731</td><td>1.08805</td><td>0.38225</td><td>Bmb034174</td><td>e-105</td></tr>
<tr><td>sw08116</td><td>BGIBMGA011429</td><td>1.09905</td><td>1.14945</td><td>1.19755</td><td>0.3815</td><td>e-167</td><td>gi|50751398|ref|XP_422379.1| PREDICTED: similar to cAMP-dependent protein kinase catalytic subunit beta isoform 1; PKA C-beta [Gallus gallus]</td></tr>
<tr><td>sw19561</td><td>BGIBMGA001173</td><td>1</td><td>0.86105</td><td>1</td><td>0.3812</td><td>No hits found</td><td></td></tr>
<tr><td>sw08164</td><td>BGIBMGA012313</td><td>1.1867</td><td>1.279</td><td>1.20485</td><td>0.3811</td><td>e-130</td><td>gi|60459962|gb|AAX20152.1| AMPK-gamma subunit [Aedes aegypti]</td></tr>
<tr><td>sw14529</td><td>BGIBMGA000901</td><td>1.035</td><td>0.9724</td><td>1.116</td><td>0.38105</td><td>No hits found</td><td></td></tr>
<tr><td>sw18325</td><td>BGIBMGA004276</td><td>0.52465</td><td>0.7175</td><td>0.77435</td><td>0.38085</td><td>4.00E-52</td><td>gi|73962512|ref|XP_859768.1| PREDICTED: similar to mitochondrial phosphoenolpyruvate carboxykinase 2 isoform 1 precursor isoform 8 [Canis familiaris]</td></tr>
<tr><td>sw22019</td><td>BGIBMGA009725</td><td>1</td><td>1</td><td>1</td><td>0.38065</td><td>Bmb003937</td><td>1.00E-24</td></tr>
<tr><td>sw22045</td><td>BGIBMGA013442</td><td>0.5464</td><td>0.9625</td><td>0.58535</td><td>0.38065</td><td>No hits found</td><td></td></tr>
<tr><td>sw11376</td><td>BGIBMGA011250</td><td>0.9746</td><td>1.0696</td><td>1.08905</td><td>0.3803</td><td>1.00E-87</td><td>gi|6946695|emb|CAB72316.1| Wee1-like CDK Tyrosine kinase [Daphnia pulex]</td></tr>
<tr><td>sw13878</td><td>BGIBMGA004453</td><td>0.9184</td><td>1.06425</td><td>1.06065</td><td>0.38025</td><td>e-110</td><td>gi|76626083|ref|XP_870049.1| PREDICTED: similar to Eyes absent homolog 4 isoform 2 [Bos taurus]</td></tr>
<tr><td>sw17684</td><td>BGIBMGA000623</td><td>1.73295</td><td>0.91525</td><td>0.9173</td><td>0.3795</td><td>0</td><td>gi|18700459|dbj|BAB85197.1| Titin-like protein [Bombyx mori]</td></tr>
<tr><td>sw01589</td><td>BGIBMGA011363</td><td>1.0359</td><td>0.931</td><td>0.94325</td><td>0.3787</td><td>Bmb024438</td><td>1.00E-06</td></tr>
<tr><td>sw10856</td><td>BGIBMGA007876</td><td>1.5477</td><td>1.15995</td><td>1.1587</td><td>0.37865</td><td>7.00E-62</td><td>gi|76650863|ref|XP_598872.2| PREDICTED: similar to Choline transporter-like protein 4 isoform 1 [Bos taurus]</td></tr>
<tr><td>sw10110</td><td>BGIBMGA007179</td><td>0.9661</td><td>1.03925</td><td>1.03565</td><td>0.37845</td><td>4.00E-16</td><td>gi|48109567|ref|XP_393109.1| PREDICTED: similar to ENSANGP00000018996 [Apis mellifera]</td></tr>
<tr><td>sw11629</td><td>BGIBMGA005718</td><td>1</td><td>1</td><td>1</td><td>0.3784</td><td>5.00E-19</td><td>gi|28194049|gb|AAO33382.1| dihydropyrimidine amidohydrolase [Drosophila melanogaster]</td></tr>
<tr><td>sw01251</td><td>BGIBMGA001988</td><td>1.67805</td><td>1.49135</td><td>0.837</td><td>0.3778</td><td>2.00E-07</td><td>gi|60678129|gb|AAX33571.1| HL05615p [Drosophila melanogaster]</td></tr>
<tr><td>sw00221</td><td>BGIBMGA001218</td><td>1.10285</td><td>1.16575</td><td>1.2889</td><td>0.37695</td><td>0</td><td>gi|73983932|ref|XP_533297.2| PREDICTED: similar to Heat shock 70 kDa protein 4L (Osmotic stress protein 94) (Heat shock 70-related protein APG-1) isoform 1 [Canis familiaris]</td></tr>
<tr><td>sw09572</td><td>BGIBMGA003642</td><td>2.0094</td><td>0.51615</td><td>1.26415</td><td>0.3768</td><td>No hits found</td><td></td></tr>
<tr><td>sw09593</td><td>BGIBMGA005106</td><td>1.1474</td><td>1.2813</td><td>1.2933</td><td>0.3766</td><td>Bmb032355</td><td>1.00E-21</td></tr>
<tr><td>sw00225</td><td>BGIBMGA004379</td><td>1.05635</td><td>1.07265</td><td>1.2847</td><td>0.37635</td><td>1.00E-58</td><td>gi|28972560|dbj|BAC65696.1| mKIAA0979 protein [Mus musculus]</td></tr>
<tr><td>sw20591</td><td>BGIBMGA001349</td><td>1</td><td>1</td><td>1</td><td>0.3763</td><td>Bmb026435</td><td>9.00E-44</td></tr>
<tr><td>sw16021</td><td>BGIBMGA009599</td><td>1.41965</td><td>0.9777</td><td>0.9311</td><td>0.3759</td><td>Bmb037573</td><td>No hits found</td></tr>
<tr><td>sw17845</td><td>BGIBMGA001776</td><td>1</td><td>1</td><td>1</td><td>0.3754</td><td>1.00E-55</td><td>gi|72092399|ref|XP_790598.1| PREDICTED: similar to helentron 3 helitron-like transposon replicase/helicase/endonuclease [Strongylocentrotus purpuratus]</td></tr>
<tr><td>sw20954</td><td>BGIBMGA006808</td><td>1</td><td>1</td><td>1</td><td>0.3753</td><td>2.00E-29</td><td>gi|72090091|ref|XP_788672.1| PREDICTED: similar to MondoA [Strongylocentrotus purpuratus]</td></tr>
</table>

sw01299	BGIBMGA004403	0.0916	0.43405	0.2457	0.37515	e-150	gi|10907|emb|CAA38531.1| 30K protein [Bombyx mori] Low molecular mass 30 kDa lipoprotein 21G1 precursor
sw18885	BGIBMGA007764	0.99015	0.9735	1.01455	0.3748	8.00E-05	gi|45382883|ref|NP_990840.1| fibroblast growth factor receptor 3 (achondroplasia, thanatophoric dwarfism) [Gallus gallus] Tyrosine kinase receptor CEK2 precursor cek2 protein
sw06565	BGIBMGA009417	1	1	1	0.3746	5.00E-06	gi|55770707|ref|XP_550395.1| putative MAR binding filament-like protein 1 [Oryza sativa (japonica cultivar-group)] putative MAR binding filament-like protein 1 [Oryza sativa (japonica cultivar-group)]
sw00708	BGIBMGA007315	1	1	1	0.37435	e-154	gi|76652847|ref|XP_592719.2| PREDICTED: similar to ubiquitin specific protease 7 (herpes virus-associated) [Bos taurus]
sw20332	BGIBMGA011069	1	1	1	0.37355	No hits found	
sw17956	BGIBMGA005889	1.03045	1.3253	1.3701	0.37345	No hits found	
sw19250	BGIBMGA008060	1.31375	1.03955	1.41085	0.3731	0	gi|27818925|gb|AAO23562.1| aminopeptidase N4 [Helicoverpa armigera]
sw18954	BGIBMGA014270	1.10395	0.94285	0.82785	0.3728	2.00E-54	gi|54643658|gb|EAL32401.1| GA15301-PA [Drosophila pseudoobscura]
sw03783	BGIBMGA013475	1.2286	1	1	0.37235	2.00E-05	gi|48137797|ref|XP_396816.1| PREDICTED: similar to inhibitory protein SMAD6 [Apis mellifera]
sw03891	BGIBMGA001730	0.9337	1.1112	1.2749	0.37215	4.00E-57	gi|1362608|pir||A53909 gelsolin, secreted form precursor - fruit fly (Drosophila melanogaster) The biology of this fly protein has not yet been explored. Its identification as a secretory gelsolin is based on sequence comparison to the vertebrate
sw01846	BGIBMGA005135	0.56165	0.61475	0.73475	0.372	Bmb028716	2.00E-32
sw09024	BGIBMGA005181	1.3062	1.11325	1.0238	0.37195	2.00E-22	gi|88698232|gb|AAY87162.2| trehalose 6-phosphate synthase [Helicoverpa armigera]
sw14296	BGIBMGA008429	0.83215	0.9954	1	0.37065	2.00E-62	gi|83015999|ref|XP_917609.1| PREDICTED: similar to Zinc finger protein Kr18 (HKr18) [Mus musculus]
sw17710	BGIBMGA009443	1	1	1	0.3703	Bmb041188	2.00E-38
sw14924	BGIBMGA001847	1	0.98715	1	0.3701	4.00E-08	gi|73987473|ref|XP_542180.2| PREDICTED: similar to nicalin [Canis familiaris]
sw13752	BGIBMGA003185	1	1	1	0.37005	Bmb020551	1.00E-21
sw04261	BGIBMGA008018	1	1.30745	1.61635	0.3698	Bmb030094	2.00E-77
sw14339	BGIBMGA005549	1.03755	0.98885	1.01945	0.3695	Bmb024531	1.00E-17
sw20886	BGIBMGA013812	0.4982	0.691	0.68365	0.3694	e-121	gi|24416622|dbj|BAC22502.1| putative pheromone-degrading enzyme [Antheraea polyphemus]
sw22364	BGIBMGA013477	1	1	1	0.3694	6.00E-60	gi|72133877|ref|XP_788695.1| PREDICTED: similar to predicted CDS, reverse transcriptase family member (1O881) [Strongylocentrotus purpuratus]
sw16197	BGIBMGA010698	0.80675	0.7929	0.9112	0.36905	No hits found	
sw07824	BGIBMGA007568	1	1	1	0.3688	5.00E-27	gi|15430244|gb|AAK98515.1| N-acetylglucosamine-6-sulfatase [Coturnix coturnix] Extracellular sulfatase Sulf-1 precursor (QSulf1)
sw12066	BGIBMGA000798	1	1	1	0.3685	1.00E-29	gi|25012554|gb|AAN71378.1| RE36563p [Drosophila melanogaster]
sw13097	BGIBMGA012783	1	1	1	0.3682	3.00E-19	gi|47124478|gb|AAH70104.1| KIAA1841 protein [Homo sapiens] KIAA1841 protein [Homo sapiens]
sw05336	BGIBMGA006870	1.5128	1.1495	1.63635	0.36805	9.00E-05	gi|54643010|gb|EAL31754.1| GA13991-PA [Drosophila pseudoobscura]
sw14492	BGIBMGA007569	1.44765	0.87255	1	0.3662	2.00E-90	gi|21429186|gb|AAM50312.1| SD04414p [Drosophila melanogaster]
sw01150	BGIBMGA009681	1.14395	0.96455	0.89145	0.36615	6.00E-10	gi|28317019|gb|AAO39529.1| RE22259p [Drosophila melanogaster]
sw05071	BGIBMGA003428	1.0397	0.96475	1.4743	0.36595	Bmb002410	0

sw17641	BGIBMGA011223	1	1	1	0.3658	2.00E-26	gi|45382859|ref|NP_990836.1| acetyl-Coenzyme A carboxylase alpha [Gallus gallus] Acetyl-CoA carboxylase (ACC) [Includes: Biotin carboxylase] acetyl-CoA carboxylase
sw10635	BGIBMGA008025	0.94345	0.9105	1.2705	0.3654	9.00E-80	gi|32141382|sp|Q9UBL3|ASH2L_HUMAN Set1/Ash2 histone methyltransferase complex subunit ASH2 (ASH2-like protein) similar to Drosophila ash2 gene [Homo sapiens] similar to Drosophila ash2 [Homo sapiens]
sw12375	BGIBMGA005279	1.0634	0.98885	1.5038	0.36505	0	gi|21464370|gb|AAM51988.1| RE10344p [Drosophila melanogaster]
sw03379	BGIBMGA006518	0.7941	0.67895	0.77895	0.3648	e-143	gi|66513288|ref|XP_392481.2| PREDICTED: similar to peroxinectin [Apis mellifera]
sw12753	BGIBMGA000950	1.23195	1.05465	1.1842	0.3648	0	gi|50745998|ref|XP_420332.1| PREDICTED: similar to THO complex subunit 2 (Tho2) [Gallus gallus]
sw05439	BGIBMGA013437	1	1	1	0.36405	2.00E-43	gi|5052592|gb|AAD38626.1| BcDNA.GH08773 [Drosophila melanogaster]
sw18089	BGIBMGA008568	0.7759	1.1345	1.05245	0.364	Bmb036359	5.00E-08
sw19870	BGIBMGA002295	1	1	1	0.3637	9.00E-32	gi|25012968|gb|AAN71569.1| RH35990p [Drosophila melanogaster]
sw09202	BGIBMGA004107	1	1	1	0.3636	2.00E-36	gi|40538865|ref|NP_954889.1| cAMP-specific cyclic nucleotide phosphodiesterase PDE8 [Rattus norvegicus] cAMP-specific cyclic nucleotide phosphodiesterase PDE8 [Rattus norvegicus]
sw12398	BGIBMGA013328	0.6684	1.3033	2.05725	0.36335	e-106	gi|50417388|gb|AAH77133.1| Staphylococcal nuclease domain containing 1 [Danio rerio]
sw11655	BGIBMGA009062	0.97215	0.98715	0.9721	0.36235	9.00E-11	gi|25013142|gb|AAN71684.1| SD18148p [Drosophila melanogaster]
sw06073	BGIBMGA011882	1	1	1	0.36215	No hits found	
sw18055	BGIBMGA006767	1.2769	1.1886	1.1233	0.3621	Bmb029038	e-123
sw06122	BGIBMGA013717	0.73255	0.86025	1.0664	0.36135	Bmb020986	e-103
sw22552	BGIBMGA003575	0.7604	0.85055	0.5712	0.36	No hits found	
sw14149	BGIBMGA005292	1.1142	0.95385	1.09055	0.35935	Bmb023143	2.00E-34
sw08784	BGIBMGA010377	1.17655	1.02225	0.9609	0.35835	0	gi|2282473|dbj|BAA21640.1| xanthine dehydrogenase [Bombyx mori]
sw03803	BGIBMGA011362	1	1	1	0.358	1.00E-19	gi|24585877|ref|NP_724424.1| scarface CG11066-PA, isoform A [Drosophila melanogaster] scarface CG11066-PB, isoform B [Drosophila melanogaster] GH05918p [Drosophila melanogaster] CG11066-PB, isoform B
sw19927	BGIBMGA012507	1.0925	1.3935	1.3543	0.3579	Bmb039369	4.00E-08
sw13968	BGIBMGA005226	0.2331	0.51245	0.20095	0.35755	9.00E-48	gi|73955036|ref|XP_853231.1| PREDICTED: similar to ATP-binding cassette, sub-family G, member 4 [Canis familiaris]
sw09217	BGIBMGA000630	1	1	1	0.35705	5.00E-82	gi|76613294|ref|XP_878881.1| PREDICTED: similar to WD-repeat protein 47 isoform 3 [Bos taurus]
sw11275	BGIBMGA014206	1.6095	1.36905	1.28195	0.3567	1.00E-42	gi|24662101|ref|NP_652439.1| dpr6 CG14162-PA [Drosophila melanogaster] LD13525p [Drosophila melanogaster] CG14162-PA [Drosophila melanogaster]
sw14337	BGIBMGA012195	1	1	1	0.35585	4.00E-06	gi|68366904|ref|XP_687110.1| PREDICTED: similar to adenosine deaminase [Danio rerio]
sw05552	BGIBMGA010602	1	1	1	0.3556	0	gi|66506280|ref|XP_394455.2| PREDICTED: similar to Protein KIAA0274 [Apis mellifera]
sw18675	BGIBMGA001723	0.19595	0.648	0.4237	0.35555	6.00E-33	gi|82950923|ref|XP_898519.1| PREDICTED: similar to zinc finger protein 709 [Mus musculus]
sw14618	BGIBMGA001540	1	1	1	0.3555	0	gi|76620868|ref|XP_612154.2| PREDICTED: similar to regulator of nonsense transcripts 1 [Bos taurus]
sw05220	BGIBMGA008017	1.68155	1.35665	1.76995	0.3552	0	gi|3402300|dbj|BAA32140.1| aminopeptidase N [Bombyx mori]
sw17594	BGIBMGA013548	0.76825	0.91825	1.05425	0.3549	Bmb011738	3.00E-84

sw21343	BGIBMGA014532	1	1	1	0.3548	2.00E-21	gi|62642463|ref|XP_219988.3| PREDICTED: similar to novel protein [Rattus norvegicus]
sw01505	BGIBMGA010999	0.84785	1.33385	1	0.3542	3.00E-25	gi|72172673|ref|XP_780956.1| PREDICTED: similar to transmembrane protein 39a [Strongylocentrotus purpuratus]
sw15513	BGIBMGA005105	1	1	1	0.3539	Bmb033312	No hits found
sw11530	BGIBMGA012781	1	1	1	0.35355	2.00E-26	gi|66555036|ref|XP_393093.2| PREDICTED: similar to ENSANGP00000013872 [Apis mellifera]
sw22430	BGIBMGA009441	1	1	1	0.35225	Bmb042980	6.00E-22
sw11624	BGIBMGA003720	1.42455	1.4187	0.70535	0.352	2.00E-08	gi|68367284|ref|XP_707274.1| PREDICTED: similar to sphingomyelin phosphodiesterase 1, acid lysosomal isoform 2 precursor isoform 3 [Danio rerio]
sw18171	BGIBMGA004550	0.8582	1.04685	1.28255	0.35195	Bmb003499	e-118
sw18944	BGIBMGA000809	1	1	1	0.35185	Bmb014713	2.00E-32
sw15731	BGIBMGA000204	0.9138	1.1025	1.1066	0.35155	Bmb035060	2.00E-22
sw13734	BGIBMGA011364	0.88665	1.00215	1.04925	0.3496	Bmb020467	8.00E-63
sw20725	BGIBMGA007679	1	0.77665	1	0.3492	7.00E-33	gi|66546388|ref|XP_392296.2| PREDICTED: similar to GA11585-PA [Apis mellifera]
sw11965	BGIBMGA005177	1.2406	1.03925	1.2109	0.3488	2.00E-91	gi|87248575|gb|ABD36340.1| PTB-associated splicing factor [Bombyx mori]
sw02650	BGIBMGA004178	1	1.01745	1.0318	0.34835	Bmb046489	No hits found
sw20256	BGIBMGA011405	1	1	1	0.34785	0	gi|55645159|ref|XP_511395.1| PREDICTED: similar to neurofibromin; Neurofibromin (neurofibromatosis, type I) [Pan troglodytes]
sw09418	BGIBMGA002695	1.0928	1.06965	1.0503	0.34765	No hits found	
sw01405	BGIBMGA009563	1.0599	1.0825	0.9961	0.3473	3.00E-39	gi|76654987|ref|XP_879917.1| PREDICTED: similar to cyclin M2 isoform 2 isoform 9 [Bos taurus]
sw12458	BGIBMGA006506	1	1	1.23695	0.3472	3.00E-62	gi|28380977|gb|AAO41456.1| RE20724p [Drosophila melanogaster]
sw03870	BGIBMGA000638	1	1	1	0.3471	1.00E-38	gi|47155829|gb|AAT11862.1| iroquois-like protein [Tribolium castaneum]
sw17926	BGIBMGA008304	1.07205	1.06445	1.12855	0.347	Bmb004111	e-125
sw09265	BGIBMGA006906	1	1	1	0.3468	Bmb026874	2.00E-15
sw02555	BGIBMGA000866	1.671	1.03705	1.29415	0.3467	Bmb043733	1.00E-07
sw07171	BGIBMGA011207	1.2207	0.81085	1.02685	0.3464	Bmb045644	1.00E-13
sw15971	BGIBMGA003037	1.4468	1.04835	1.2142	0.34585	7.00E-37	gi|89243264|gb|ABD64784.1| Bent [Drosophila virilis]
sw03125	BGIBMGA010725	0.74075	1.00915	1.26135	0.3451	e-104	gi|72167287|ref|XP_796805.1| PREDICTED: similar to Coatomer alpha subunit (Alpha-coat protein) (Alpha-COP) (HEPCOP) (HEP-COP) [Strongylocentrotus purpuratus]
sw22078	BGIBMGA008083	1	1	1	0.34495	8.00E-14	gi|72043882|ref|XP_793468.1| PREDICTED: similar to zn-finger, CCHC type and RNA-directed DNA polymerase and Integrase, catalytic domain containing protein (3J949) [Strongylocentrotus purpuratus]
sw12973	BGIBMGA003926	0.6738	1.2856	0.84715	0.3448	8.00E-95	gi|5759228|gb|AAD51036.1| cytochrome P450 [Manduca sexta]
sw06028	BGIBMGA005338	1	1	1	0.3443	Bmb019370	4.00E-33
sw20460	BGIBMGA001272	1.5142	1.5026	1.83055	0.34425	Bmb008625	6.00E-81

sw09820	BGIBMGA012753	1.0547	1.00155	1.46245	0.34335	0	gi|431944|emb|CAA53948.1| Ppk 98; a protein kinase [Sus scrofa] Endoplasmin precursor (94 kDa glucose-regulated protein) (GRP94) (GP96 homolog) (98 kDa protein kinase) (PPK 98) (ppk98)
sw20435	BGIBMGA013978	1	1	1	0.3428	4.00E-19	gi|8926400|gb|AAF81829.1| DISCS LOST; dlt [Drosophila melanogaster]
sw03965	BGIBMGA005182	1	1	1	0.3426	0	gi|88698232|gb|AAY87162.2| trehalose 6-phosphate synthase [Helicoverpa armigera]
sw00653	BGIBMGA012048	0.70625	0.7912	0.70135	0.341	1.00E-10	gi|1209648|gb|AAC47002.1| protein tyrosine phosphatase DLAR protein tyrosine phosphatase (DLAR) precursor
sw20095	BGIBMGA008834	1.1235	1.1027	0.852	0.341	No hits found	
sw22610	BGIBMGA000379	0.9618	0.868	0.92515	0.3406	2.00E-21	gi|68398837|ref|XP_697912.1| PREDICTED: similar to Zinc finger protein 184 (Kruppel-like) [Danio rerio]
sw17697	BGIBMGA004431	1	1	1	0.34035	Bmb036890	2.00E-21
sw04820	BGIBMGA012054	1.2277	1.18575	0.88915	0.33985	Bmb048235	2.00E-15
sw20255	BGIBMGA001126	0.48865	0.96765	1.0703	0.33975	2.00E-86	gi|10946670|ref|NP_067325.1| choroideremia-like [Mus musculus] rab escort protein-2 [Mus musculus]
sw02441	BGIBMGA009109	1	1	1	0.3397	No hits found	
sw07527	BGIBMGA001794	1.4797	1.23285	1	0.3393	2.00E-06	gi|66562084|ref|XP_395379.2| PREDICTED: similar to ENSANGP00000010681 [Apis mellifera]
sw11733	BGIBMGA001790	0.75355	0.85565	0.9777	0.3393	Bmb007728	2.00E-23
sw13774	BGIBMGA002092	0.34125	0.77245	0.38855	0.339	3.00E-18	gi|47086753|ref|NP_997808.1| solute carrier family 35, member C2 [Danio rerio] Ovarian cancer overexpressed 1 [Danio rerio]
sw10718	BGIBMGA011333	1	1	1	0.3386	4.00E-14	gi|51471144|gb|AAU04329.1| transformed mouse 3T3 cell double minute 2 [Mus caroli]
sw12815	BGIBMGA001579	1	1	1	0.3385	e-167	gi|4505343|ref|NP_002477.1| nuclear cap binding protein subunit 1, 80kDa [Homo sapiens] nuclear cap binding protein subunit 1, 80kDa [Homo sapiens] nuclear cap binding protein subunit 1, 80kDa [Homo sapiens] 80 kDa nuclear cap-bindin
sw01427	BGIBMGA008871	1.08125	0.93175	1.33025	0.3377	1.00E-58	gi|55242794|gb|EAA07084.3| ENSANGP00000016038 [Anopheles gambiae str. PEST] ENSANGP00000016038 [Anopheles gambiae str. PEST]
sw11321	BGIBMGA010911	1	1	1	0.3371	No hits found	
sw17936	BGIBMGA000672	1.323	1.14345	1.3928	0.3359	0	gi|41058938|gb|AAR98861.1| mitochondrial citrate synthase precursor [Xiphias gladius]
sw14253	BGIBMGA005736	1.45445	1.05765	1.0525	0.3354	4.00E-91	gi|55244645|gb|EAA05197.3| ENSANGP00000017954 [Anopheles gambiae str. PEST] ENSANGP00000017954 [Anopheles gambiae str. PEST]
sw12244	BGIBMGA007848	1.13745	1.03395	1.0081	0.3353	0	gi|73967590|ref|XP_857927.1| PREDICTED: similar to sarcosine dehydrogenase isoform 3 [Canis familiaris]
sw11643	BGIBMGA002894	1.14955	1.0263	1.09235	0.33455	0	gi|5764409|gb|AAD51291.1| DGP-1 protein [Drosophila melanogaster]
sw20970	BGIBMGA006959	0.8505	0.84895	0.92085	0.33395	0	gi|399056|sp|Q02453|ARM_MUSDO Armadillo segment polarity protein armadillo protein
sw08517	BGIBMGA011119	0.43055	0.7939	0.79055	0.33375	No hits found	
sw13580	BGIBMGA007512	0.25805	0.7325	0.55705	0.33345	e-168	gi|790585|gb|AAA85850.1| UDP-glucose:glycoprotein glucosyltransferase precursor
sw13955	BGIBMGA004691	1.3593	1.2494	1.0337	0.33345	1.00E-57	gi|22138110|gb|AAM93435.1| PARP-E protein [Drosophila melanogaster]
sw06062	BGIBMGA008185	1	1	1	0.3333	3.00E-43	gi|73961507|ref|XP_854829.1| PREDICTED: similar to F-box only protein 28 [Canis familiaris]
sw21674	BGIBMGA011334	1.2692	1.09745	0.90575	0.3332	2.00E-34	gi|66566395|ref|XP_623926.1| PREDICTED: similar to GA18544-PA [Apis mellifera]
sw11208	BGIBMGA012248	1	1	1	0.33265	No hits found	

sw17606	BGIBMGA000509	1	1	1	0.33255	3.00E-05	gi|66546685|ref|XP_395583.2| PREDICTED: similar to polyhomeotic 3 [Apis mellifera]
sw07932	BGIBMGA000147	1	1	1	0.3322	2.00E-25	gi|2467112|emb|CAA75228.1| Lola-like protein [Drosophila hydei]
sw11253	BGIBMGA003971	1	1	1	0.3316	1.00E-25	gi|76154169|gb|AAX25663.2| SJCHGC08483 protein [Schistosoma japonicum]
sw17651	BGIBMGA010274	0.0536	0.648	0.6212	0.33155	Bmb023886	2.00E-09
sw04234	BGIBMGA014531	1	1	1	0.33125	No hits found	
sw19907	BGIBMGA007184	1.676	0.9771	0.79115	0.33095	No hits found	
sw13690	BGIBMGA012030	0.65465	1.2146	1.1692	0.3301	No hits found	
sw19049	BGIBMGA002618	1.2562	1.0298	1.2259	0.3293	2.00E-28	gi|7682380|gb|AAF67245.1| gp150 [Drosophila virilis]
sw05088	BGIBMGA000120	1.0096	1.095	1.1647	0.32805	e-123	gi|55238955|gb|EAA10832.2| ENSANGP00000017705 [Anopheles gambiae str. PEST] ENSANGP00000017705 [Anopheles gambiae str. PEST]
sw07090	BGIBMGA012702	1	1	1	0.32765	Bmb042755	4.00E-31
sw19748	BGIBMGA004714	1	1	1	0.32675	1.00E-14	gi|72014020|ref|XP_783244.1| PREDICTED: similar to cytochrome P450, family 4, subfamily v, polypeptide 2 [Strongylocentrotus purpuratus]
sw22874	BGIBMGA005130	1.16045	1.27945	0.9834	0.3263	8.00E-06	gi|33354081|dbj|BAC81124.1| L1 cell adhesion molecule [Pongo pygmaeus]
sw09307	BGIBMGA008931	0.34445	0.65835	0.4997	0.32625	No hits found	
sw16006	BGIBMGA013378	1.0815	1.18605	0.81585	0.32615	Bmb037438	7.00E-38
sw00905	BGIBMGA007738	1	1	1	0.32605	3.00E-89	gi|76631800|ref|XP_593336.2| PREDICTED: similar to ATP-binding cassette, sub-family C, member 4 [Bos taurus]
sw12281	BGIBMGA007633	1.60215	1.64975	2.17665	0.3259	6.00E-06	gi|73967692|ref|XP_537809.2| PREDICTED: similar to adenylate kinase 3 isoform 1 [Canis familiaris]
sw14927	BGIBMGA008268	0.55355	0.57775	0.68505	0.3246	Bmb028811	e-152
sw20305	BGIBMGA004592	1	1.01145	0.9083	0.32445	4.00E-31	gi|50752056|ref|XP_422633.1| PREDICTED: similar to calsyntenin-2 [Gallus gallus]
sw11598	BGIBMGA001132	0.8814	0.827	0.89765	0.3237	1.00E-67	gi|551090|gb|AAA50238.1| DNA-binding protein
sw11048	BGIBMGA004712	1	1	1	0.32245	1.00E-08	gi|27316716|gb|AAO05891.1| streptococcal hemagglutinin protein [Staphylococcus epidermidis ATCC 12228] streptococcal hemagglutinin protein [Staphylococcus epidermidis ATCC 12228]
sw15141	BGIBMGA001012	1	1	1	0.32245	2.00E-58	gi|33880192|gb|AAH50772.2| Testis-specific serine kinase 1 [Mus musculus]
sw02984	BGIBMGA005477	1.16195	0.90025	1.0779	0.3224	0	gi|440144|emb|CAA52155.1| integrin [Drosophila melanogaster]
sw11166	BGIBMGA003938	1	1	1	0.32105	Bmb004077	0
sw06172	BGIBMGA006559	1	1.1237	1	0.32095	4.00E-30	gi|76654169|ref|XP_618212.2| PREDICTED: similar to neural stem cell-derived dendrite regulator [Bos taurus]
sw20313	BGIBMGA001183	1	1	1	0.32075	Bmb026077	2.00E-71
sw18783	BGIBMGA000528	1.1408	1.06255	1.2067	0.3204	No hits found	
sw20096	BGIBMGA000826	0.9601	0.93215	0.87915	0.32035	7.00E-38	gi|59939802|gb|AAQ62963.2| transferrin [Romalea microptera]
sw02978	BGIBMGA004568	1	1	1	0.3198	e-109	gi|28972886|dbj|BAC65859.1| mKIAA3002 protein [Mus musculus]

sw07109	BGIBMGA013544	1	1	1	0.3193	Bmb043396	2.00E-05	
sw04705	BGIBMGA004786	1	1	1	0.31905	Bmb043440	6.00E-52	
sw20203	BGIBMGA014105	1.4459	1.1992	1.67095	0.31885	3.00E-59	gi|3004821|gb|AAC39088.1| putative inorganic phosphate cotransporter [Drosophila ananassae] Putative inorganic phosphate cotransporter	
sw08603	BGIBMGA002043	0.44145	1.2861	0.6568	0.3188	No hits found		
sw12171	BGIBMGA012794	0.2108	0.57345	0.4372	0.3188	1.00E-26	gi|5815245|gb|AAD52614.1| SANT domain protein SMRTER [Drosophila melanogaster]	
sw15024	BGIBMGA000689	1.0561	1.05	1.2622	0.3188	Bmb029529	e-178	
sw06336	BGIBMGA009138	0.8087	1.14735	1	0.3187	5.00E-26	gi|62897749|dbj|BAD96814.1| aminopeptidase-like 1 variant [Homo sapiens]	
sw00477	BGIBMGA010943	0.95155	1.0448	0.8655	0.31715	2.00E-76	gi|87248265|gb|ABD36185.1| ADP-ribosylation factor-like protein [Bombyx mori]	
sw11389	BGIBMGA008251	1.3557	1.37055	1.77125	0.31695	No hits found		
sw20748	BGIBMGA011671	1	1	1	0.3168	4.00E-21	gi|68364550|ref|XP_690945.1| PREDICTED: similar to senataxin; DEAxQ-box helicase; tRNA splicing endonuclease regulator 1, partial [Danio rerio]	
sw20474	BGIBMGA000850	1.19925	0.9267	1.24305	0.31615	3.00E-10	gi|41235797|ref|NP_958738.1| SH3/ankyrin domain gene 2 isoform a [Rattus norvegicus] Shank2E [Rattus norvegicus]	
sw00648	BGIBMGA007835	1.0709	1.1118	1.1215	0.3145	2.00E-79	gi|73988919|ref|XP_542530.2| PREDICTED: similar to neuron navigator 2 isoform 2 [Canis familiaris]	
sw22969	BGIBMGA011427	1	1	1	0.3142	No hits found		
sw12469	BGIBMGA003472	1	1	1	0.31385	e-168	gi|2547046|dbj|BAA22868.1| bHLH-PAS transcription factor [Drosophila melanogaster]	
sw15709	BGIBMGA006226	1	1	1	0.31385	Bmb034922	e-169	
sw15794	BGIBMGA001836	0.7317	0.82395	0.69925	0.31375	1.00E-70	gi|1705995|sp|P49747|COMP_HUMAN Cartilage oligomeric matrix protein precursor (COMP) cartilage oligomeric matrix protein [Homo sapiens] matrix protein	
sw18498	BGIBMGA003458	1.2558	1.17535	1.21275	0.31365	Bmb037077	3.00E-61	
sw08951	BGIBMGA009402	1	1	1.4617	0.313	0	gi|55243234|gb|EAA06289.2| ENSANGP00000017306 [Anopheles gambiae str. PEST] ENSANGP00000017306 [Anopheles gambiae str. PEST]	
sw14920	BGIBMGA012759	1.14825	0.93115	0.9319	0.31275	No hits found		
sw00665	BGIBMGA002139	0.3204	0.67655	0.5236	0.31255	No hits found		
sw02031	BGIBMGA002544	0.8898	0.8823	1.1425	0.31205	e-121	gi|473595|gb|AAA19857.1| moesin/ezrin/radixin homolog	
sw15086	BGIBMGA004113	1	1	1	0.3108	No hits found		
sw20167	BGIBMGA013819	1.24005	1.02785	1.06105	0.30995	No hits found		
sw12513	BGIBMGA003789	1	1	1	0.308	e-153	gi|1373390|gb|AAB05596.1| SH2/SH3 adaptor protein	
sw06051	BGIBMGA001271	1.029	0.95115	1.04605	0.3076	No hits found		
sw05711	BGIBMGA006106	1	1	1	0.30685	2.00E-11	gi|68440173|ref|XP_683330.1| PREDICTED: similar to BTB/POZ domain containing protein 3 [Danio rerio]	
sw13179	BGIBMGA008337	1.0928	0.95285	1.0602	0.30675	1.00E-33	gi|55640947|ref|XP_510026.1| PREDICTED: similar to Butyrate response factor 1 (TIS11B protein) (EGF-response factor 1) (ERF-1) [Pan troglodytes]	
sw19370	BGIBMGA010289	1	1	1	0.30655	1.00E-57	gi|24645837|ref|NP_652625.2| Ugt86Dc CG4739-PA [Drosophila melanogaster] CG4739-PA [Drosophila melanogaster]	

sw06003	BGIBMGA010323	1	1	1	0.30595	7.00E-07	gi|54637562|gb|EAL26964.1| GA14328-PA [Drosophila pseudoobscura]
sw12898	BGIBMGA014227	1.33735	1.163	1.43615	0.30365	0	gi|28574239|ref|NP_523587.4| Myosin heavy chain CG17927-PH, isoform H [Drosophila melanogaster] CG17927-PH, isoform H [Drosophila melanogaster]
sw18813	BGIBMGA011052	1.1088	1.01315	1.23915	0.3033	No hits found	
sw10899	BGIBMGA010644	1.1544	0.93765	1.09765	0.3031	0	gi|4165140|gb|AAD08704.1| PIWI [Drosophila melanogaster]
sw04254	BGIBMGA010111	1	0.95585	1	0.303	1.00E-12	gi|27819785|gb|AAO24941.1| RE65015p [Drosophila melanogaster]
sw19986	BGIBMGA003574	0.7738	0.7956	0.58085	0.30245	6.00E-80	gi|61554518|gb|AAX46572.1| atlastin [Bos taurus]
sw20339	BGIBMGA002719	0.7915	0.8951	0.7901	0.30245	Bmb032487	No hits found
sw07497	BGIBMGA002882	1	1	1	0.302	6.00E-12	gi|29145087|gb|AAH49104.1| Oxoglutarate dehydrogenase (lipoamide) [Mus musculus] 2-oxoglutarate dehydrogenase E1 component, mitochondrial precursor (Alpha-ketoglutarate dehydrogenase)
sw19762	BGIBMGA005738	1.24585	1.1023	1.1462	0.30125	7.00E-38	gi|72004925|ref|XP_781695.1| PREDICTED: similar to ankyrin 3, epithelial isoform a [Strongylocentrotus purpuratus]
sw12943	BGIBMGA001971	0.13045	0.68745	0.3153	0.3002	No hits found	
sw01980	BGIBMGA014369	1.6899	1.00735	1.31545	0.29835	8.00E-05	gi|54641695|gb|EAL30445.1| GA21796-PA [Drosophila pseudoobscura]
sw10995	BGIBMGA011993	1	1	1	0.2975	1.00E-14	gi|50740576|ref|XP_419499.1| PREDICTED: similar to GTP binding protein 2 [Gallus gallus]
sw16843	BGIBMGA007680	1	1	1	0.29735	Bmb048080	No hits found
sw19701	BGIBMGA000132	0.68715	0.71415	0.9058	0.2972	5.00E-07	gi|76657688|ref|XP_591861.2| PREDICTED: similar to alpha glucosidase II alpha subunit isoform 2 [Bos taurus]
sw15347	BGIBMGA007924	1	1	1	0.2963	Bmb031972	e-144
sw16391	BGIBMGA010699	0.82455	0.92135	0.9053	0.29565	7.00E-06	gi|72098908|ref|XP_799322.1| PREDICTED: similar to Ileal sodium/bile acid cotransporter (Ileal Na(+)/bile acid cotransporter) (Na(+) dependent ileal bile acid transporter) (Ileal sodium-dependent bile acid transporter) (ISBT) (Sodium/taurocho
sw10866	BGIBMGA003411	1.2389	0.83	0.8238	0.29525	1.00E-43	gi|66515966|ref|XP_396216.2| PREDICTED: similar to corin [Apis mellifera]
sw10062	BGIBMGA006852	1.1463	1.05665	1.124	0.2951	Bmb041700	1.00E-10
sw08905	BGIBMGA008090	1.3788	0.8599	0.9402	0.29445	No hits found	
sw19810	BGIBMGA014221	1.1467	1.0394	1.01015	0.29445	0	gi|2575865|dbj|BAA23126.1| BmP109 [Bombyx mori]
sw18355	BGIBMGA004061	1	1	1	0.2943	0	gi|10801051|dbj|BAB16608.1| 210kDa protein [Sarcophaga peregrina]
sw19426	BGIBMGA002157	1	1	1	0.2941	4.00E-30	gi|1621281|emb|CAA70088.1| GATA-6 protein [Xenopus laevis] GATA-binding factor 6-B (Transcription factor xGATA-6B)
sw12537	BGIBMGA004896	1	1	1	0.2938	7.00E-19	gi|40788939|dbj|BAA13442.2| KIAA0013 [Homo sapiens]
sw03215	BGIBMGA005503	0.7993	0.89585	1.12805	0.2933	8.00E-56	gi|3983137|gb|AAC83821.1| Lis1 homolog [Drosophila melanogaster]
sw11257	BGIBMGA014069	0.95685	0.98045	1.0687	0.2933	No hits found	
sw11565	BGIBMGA000910	1.2666	0.9709	1.0301	0.2926	5.00E-97	gi|12657593|dbj|BAB21565.1| laminin [Bombyx mori]
sw08530	BGIBMGA000639	0.28835	0.64375	0.4299	0.29165	1.00E-36	gi|72049105|ref|XP_795008.1| PREDICTED: similar to Iroquois-class homeodomain protein IRX-4 (Iroquois homeobox protein 4) (Homeodomain protein IRXA3) [Strongylocentrotus purpuratus]
sw11595	BGIBMGA010929	1	1	1	0.29095	2.00E-05	gi|24645680|ref|NP_524775.1| CG17100-PA [Drosophila melanogaster] CG17100-PA [Drosophila melanogaster]

sw02946	BGIBMGA000622	1.71835	0.92215	1.50265	0.2909	0	gi|22474512|dbj|BAC10617.1| KETTIN [Bombyx mori] BMKETTIN [Bombyx mori]
sw17139	BGIBMGA001498	1.06285	1.56295	0.9359	0.2895	2.00E-20	gi|66499643|ref|XP_393415.2| PREDICTED: similar to ENSANGP00000012563 [Apis mellifera]
sw12077	BGIBMGA003667	1	0.96715	1	0.28945	e-117	gi|73999471|ref|XP_535098.2| PREDICTED: similar to organic anion transporter polypeptide-related protein 4 [Canis familiaris]
sw18008	BGIBMGA002296	0.78025	1.07965	0.94095	0.2894	2.00E-06	gi|25012968|gb|AAN71569.1| RH35990p [Drosophila melanogaster]
sw12755	BGIBMGA009101	1.4294	2.06265	1.4761	0.28915	No hits found	
sw20070	BGIBMGA004577	0.9036	1.0255	1.06665	0.2883	2.00E-46	gi|66513968|ref|XP_394726.2| PREDICTED: similar to ENSANGP00000015204 [Apis mellifera]
sw04109	BGIBMGA011124	1.31655	1.21995	1.19675	0.2878	3.00E-48	gi|7943|emb|CAA48327.1| elg [Drosophila melanogaster] DNA-binding protein D-ELG
sw00950	BGIBMGA003771	1.5056	1.06195	1	0.28755	9.00E-25	gi|37181975|gb|AAQ88791.1| LLPL [Homo sapiens] lysophospholipase 3 (lysosomal phospholipase A2) [Homo sapiens] Lysophospholipase 3 (lysosomal phospholipase A2) [Homo sapiens] 1-O-acylceramide synthase precursor (ACS)
sw17939	BGIBMGA000447	1	1	1	0.28705	0	gi|6580808|gb|AAF18300.1| N-ethylmaleimide sensitive fusion protein [Manduca sexta]
sw11085	BGIBMGA010939	0.80765	0.97425	0.9136	0.2867	No hits found	
sw16435	BGIBMGA008856	1	1	1	0.28625	4.00E-31	gi|58585102|ref|NP_001011581.1| cGMP-dependent protein kinase foraging [Apis mellifera] cGMP-dependent protein kinase foraging [Apis mellifera]
sw20223	BGIBMGA013099	1	0.2221	0.36355	0.2858	6.00E-23	gi|89243283|gb|ABD64800.1| Dvir_CG14130 [Drosophila virilis]
sw09808	BGIBMGA011525	1.3254	1.16315	0.8453	0.28515	Bmb036406	2.00E-21
sw08392	BGIBMGA001551	0.9424	0.8965	1.0503	0.28495	e-109	gi|73946504|ref|XP_858102.1| PREDICTED: similar to ubiquilin 1 isoform 1 isoform 3 [Canis familiaris]
sw11999	BGIBMGA012308	1.00385	0.83555	1.14115	0.28455	No hits found	
sw21986	BGIBMGA009325	1	1	1	0.28335	8.00E-51	gi|66505065|ref|XP_392243.2| PREDICTED: similar to rho-type GTPase-activating protein rhoGAPX-1 [Apis mellifera]
sw01142	BGIBMGA007303	0.2957	1.038	0.61325	0.28315	3.00E-09	gi|57087751|ref|XP_546987.1| PREDICTED: similar to Basic helix-loop-helix protein MIST1 (Muscle, intestine and stomach expression 1) [Canis familiaris]
sw13263	BGIBMGA009857	1.08285	1.2173	1	0.28305	5.00E-99	gi|466258|gb|AAA19591.1| myosin-IB
sw20505	BGIBMGA000613	1.1651	0.97025	1.3285	0.2823	3.00E-85	gi|10959|emb|CAA41557.1| paramyosin [Drosophila melanogaster]
sw17725	BGIBMGA006043	0.8737	0.8671	1	0.28225	e-137	gi|87248413|gb|ABD36259.1| stathmin [Bombyx mori]
sw18782	BGIBMGA014600	1	1	1	0.2822	5.00E-47	gi|66521636|ref|XP_396241.2| PREDICTED: similar to ENSANGP00000010275, partial [Apis mellifera]
sw01513	BGIBMGA009974	1.21595	0.9889	1.0316	0.28205	Bmb023073	6.00E-81
sw05425	BGIBMGA011984	2.1819	1	1	0.28115	e-115	gi|50753737|ref|XP_414111.1| PREDICTED: similar to alanine aminotransferase 2; glutamic-pyruvate transaminase 2 [Gallus gallus]
sw04154	BGIBMGA001570	1.14915	1.3521	0.95105	0.2792	e-141	gi|87248571|gb|ABD36338.1| phosphoribosyl pyrophosphate synthetase [Bombyx mori]
sw11956	BGIBMGA004343	0.4774	1.11505	0.72175	0.27885	1.00E-04	gi|48096382|ref|XP_392446.1| PREDICTED: similar to death-associated protein [Apis mellifera]
sw14909	BGIBMGA009161	1.36355	1.0846	0.9934	0.27845	No hits found	
sw02464	BGIBMGA010275	1.3635	0.94755	1.23415	0.2765	Bmb041571	No hits found
sw11512	BGIBMGA009962	1	1	1	0.27645	0	gi|66514814|ref|XP_394762.2| PREDICTED: similar to CG32498-PO, isoform O [Apis mellifera]

sw20303	BGIBMGA006227	0.8707	1.0601	1.06605	0.27585	e-124	gi|7677032|gb|AAF66996.1| timeless [Antheraea pernyi]
sw07785	BGIBMGA003506	1	1	1	0.27545	6.00E-23	gi|48095153|ref|XP_392250.1| PREDICTED: similar to PDZ domain containing 3 [Apis mellifera]
sw04404	BGIBMGA012440	0.6825	0.4683	0.6968	0.27515	8.00E-12	gi|66522495|ref|XP_393918.2| PREDICTED: similar to subtilisin-related protease SPC3 [Apis mellifera]
sw06057	BGIBMGA010761	1	1	1	0.2751	6.00E-66	gi|28626480|gb|AAO49159.1| LD15982p [Drosophila melanogaster]
sw18259	BGIBMGA004852	0.709	1.1294	1.3714	0.275	0	gi|25012545|gb|AAN71374.1| RE35250p [Drosophila melanogaster]
sw18818	BGIBMGA012221	1.03895	1.20855	1.3262	0.27485	5.00E-16	gi|34924891|sp|Q26643|TRF_SARPE Transferrin precursor transferrin precursor [Sarcophaga peregrina]
sw06018	BGIBMGA002647	1.2126	1.2699	1.11765	0.2744	4.00E-65	gi|87248315|gb|ABD36210.1| erythrocyte carbonic anhydrase [Bombyx mori]
sw19955	BGIBMGA012763	1.4839	1.91125	2.0136	0.2737	e-143	gi|46396762|sp|Q27451|PRP1_BOMMO Phenoloxidase subunit 1 precursor (Tyrosinase 1) (PO 1) prophenoloxidase subunit 1 [Bombyx mori]
sw10890	BGIBMGA005341	1.1641	1.3672	1.0451	0.2732	No hits found	
sw02334	BGIBMGA007111	0.72595	0.7508	0.7914	0.2714	2.00E-26	gi|1561730|gb|AAC47288.1| Dreg-3 protein [Drosophila melanogaster]
sw19471	BGIBMGA013173	1	1	1	0.27105	1.00E-30	gi|68398138|ref|XP_684604.1| PREDICTED: similar to ROX protein [Danio rerio]
sw19621	BGIBMGA014011	1	1	1	0.27085	9.00E-30	gi|26190489|gb|AAN17506.1| laccase 1 [Manduca sexta]
sw10272	BGIBMGA005876	1	1	1	0.2705	Bmb049073	1.00E-10
sw11770	BGIBMGA000608	1	1	1	0.2701	4.00E-07	gi|44985962|gb|AAS54573.1| AGR084Cp [Ashbya gossypii ATCC 10895] AGR084Cp [Eremothecium gossypii]
sw04957	BGIBMGA008292	1	1	1	0.2689	2.00E-33	gi|72008255|ref|XP_784488.1| PREDICTED: similar to polyglutamine-containing protein [Strongylocentrotus purpuratus]
sw20523	BGIBMGA000150	1	1	1	0.2688	8.00E-15	gi|72173283|ref|XP_791277.1| PREDICTED: similar to reverse transcriptase family member (1F383) [Strongylocentrotus purpuratus]
sw12010	BGIBMGA013537	0.75565	1.64065	1.55565	0.2682	e-128	gi|72083934|ref|XP_789316.1| PREDICTED: similar to ubiquitin-activating enzyme E1-domain containing 1 isoform 1, partial [Strongylocentrotus purpuratus]
sw20315	BGIBMGA012740	0.8417	0.76145	0.74415	0.2668	3.00E-63	gi|66513293|ref|XP_623940.1| PREDICTED: similar to peroxidase [Apis mellifera]
sw14309	BGIBMGA000391	0.68305	0.78005	0.77265	0.26665	e-146	gi|16877801|gb|AAH17135.1| Glycine decarboxylase [Mus musculus] glycine decarboxylase [Mus musculus] Glycine dehydrogenase [decarboxylating], mitochondrial precursor (Glycine decarboxylase) (Glycine cleavage system P-protein)
sw19884	BGIBMGA000223	1.34255	1.3305	1.2446	0.265	1.00E-08	gi|72049850|ref|XP_787337.1| PREDICTED: similar to solute carrier family 2, (facilitated glucose transporter), member 8 [Strongylocentrotus purpuratus]
sw15021	BGIBMGA013972	1	1	1.35835	0.2648	4.00E-21	gi|50732950|ref|XP_418840.1| PREDICTED: similar to hypothetical protein FLJ22313 [Gallus gallus]
sw20285	BGIBMGA006694	0.96155	1.22285	1.04335	0.2642	3.00E-99	gi|1346296|sp|P98092|HMCT_BOMMO Hemocytin precursor (Humoral lectin) humoral lectin prepropeptide [Bombyx mori] hemocytin
sw13346	BGIBMGA007502	0.78355	1.0569	1.41205	0.26415	4.00E-49	gi|50761621|ref|XP_424784.1| PREDICTED: similar to nicotinamide nucleotide transhydrogenase [Gallus gallus]
sw05082	BGIBMGA009435	0.7238	0.6165	0.5686	0.26225	5.00E-05	gi|1030731|emb|CAA32198.1| polyprotein [Drosophila melanogaster]
sw20514	BGIBMGA003617	1	1	1	0.26215	No hits found	
sw00551	BGIBMGA008989	1.14815	0.90035	1	0.2613	0	gi|77403867|gb|ABA81812.1| RE66325p [Drosophila melanogaster]
sw13345	BGIBMGA014441	1.0167	1.0672	1.1973	0.25815	6.00E-86	gi|66556915|ref|XP_395006.2| PREDICTED: similar to Expressed sequence AU040829, partial [Apis mellifera]
sw12019	BGIBMGA003830	1	1	1	0.25785	6.00E-10	gi|58255189|gb|AAV43426.1| surface protein [Lactobacillus acidophilus NCFM] surface protein [Lactobacillus acidophilus NCFM]

sw16198	BGIBMGA005101	1	1	1	0.25625	0	gi|559307|dbj|BAA07523.1| silk gland factor-1 [Bombyx mori] Silk gland factor 1 (SGF-1)
sw05452	BGIBMGA004950	2.3673	1.85645	1.50785	0.2559	0	gi|41016826|sp|Q27772|C1TC_SPOFR C-1-tetrahydrofolate synthase, cytoplasmic (C1-THF synthase) [Includes: Methylenetetrahydrofolate dehydrogenase ; Methenyltetrahydrofolate cyclohydrolase ; Formyltetrahydrofolate synthetase
sw00121	BGIBMGA009878	1.20685	0.9554	1.134	0.2554	No hits found	
sw11736	BGIBMGA013626	1	1	1	0.2551	e-100	gi|25013151|gb|AAN71688.1| SD20577p [Drosophila melanogaster]
sw19436	BGIBMGA014356	0.4461	0.66635	0.5184	0.2546	4.00E-23	gi|72133877|ref|XP_788695.1| PREDICTED: similar to predicted CDS, reverse transcriptase family member (1O881) [Strongylocentrotus purpuratus]
sw05100	BGIBMGA005144	0.9463	0.9797	0.83045	0.2535	Bmb002772	No hits found
sw21022	BGIBMGA001981	1.432	1.1299	1.3161	0.25345	1.00E-45	gi|59807717|gb|AAH89375.1| Zswim5 protein [Mus musculus]
sw11640	BGIBMGA013439	0.316	0.6005	0.4431	0.25285	1.00E-09	gi|5052592|gb|AAD38626.1| BcDNA.GH08773 [Drosophila melanogaster]
sw15109	BGIBMGA011852	1.04385	0.8361	0.9057	0.25225	Bmb030195	5.00E-27
sw13006	BGIBMGA001466	1	1	0.85855	0.25145	No hits found	
sw09717	BGIBMGA010171	1	1	1	0.2513	Bmb034639	3.00E-44
sw14141	BGIBMGA004984	0.87545	1.11835	1.1674	0.251	1.00E-06	gi|55235760|gb|EAA14411.2| ENSANGP00000020885 [Anopheles gambiae str. PEST] ENSANGP00000020885 [Anopheles gambiae str. PEST]
sw05218	BGIBMGA007027	0.94815	0.84935	0.97245	0.2502	0	gi|68370302|ref|XP_691456.1| PREDICTED: similar to sperm protein SSP411 [Danio rerio]
sw08786	BGIBMGA011477	1.1328	0.99725	1.12485	0.25005	3.00E-98	gi|77168470|gb|ABA63172.1| hexokinase [Culex pipiens quinquefasciatus]
sw15226	BGIBMGA002958	0.73225	0.48955	0.3357	0.2497	Bmb031168	e-116
sw17734	BGIBMGA004806	1.42785	1.09845	1.43305	0.2484	0	gi|82940307|emb|CAJ34654.1| chlorophyllide A binding protein precursor [Bombyx mori]
sw11270	BGIBMGA009303	1	1	1	0.24745	8.00E-31	gi|135084|sp|P24507|SY63_DISOM Synaptotagmin C (Synaptic vesicle protein O-p65-C) synaptic vesicle protein
sw16267	BGIBMGA010352	0.63845	0.70135	0.82035	0.2468	Bmb039809	1.00E-20
sw20719	BGIBMGA001913	0.64205	0.6944	0.6419	0.24665	4.00E-16	gi|66500529|ref|XP_392121.2| PREDICTED: similar to thrombospondin repeat protein 1 [Apis mellifera]
sw07960	BGIBMGA012935	0.9125	0.9697	0.9268	0.2465	0	gi|73966639|ref|XP_867220.1| PREDICTED: similar to Clathrin heavy chain 1 (CLH-17) isoform 6 [Canis familiaris]
sw11259	BGIBMGA007783	0.1047	0.62535	0.4015	0.24595	e-148	gi|55236881|gb|EAA13239.3| ENSANGP00000017707 [Anopheles gambiae str. PEST] ENSANGP00000017707 [Anopheles gambiae str. PEST]
sw03494	BGIBMGA012388	1.1328	1.12135	1.1253	0.24515	5.00E-87	gi|66510594|ref|XP_624428.1| PREDICTED: similar to Zfr protein [Apis mellifera]
sw02003	BGIBMGA003652	1.0545	0.90185	1.11195	0.2447	1.00E-74	gi|66530257|ref|XP_396670.2| PREDICTED: similar to nuclear lamin C protein, partial [Apis mellifera]
sw17350	BGIBMGA010471	1.45985	1.0922	1.34195	0.24445	e-135	gi|24417709|gb|AAN60442.1| nesprin-1 [Homo sapiens] nesprin 1 longest [Homo sapiens]
sw14078	BGIBMGA009933	1	1	1	0.24425	Bmb022640	2.00E-35
sw14384	BGIBMGA010744	1.16195	1.08415	1.18565	0.24315	1.00E-58	gi|12619290|dbj|BAB21529.1| cathepsin B mRNA 3'-untranslated-region-binding protein CBBP [Sarcophaga peregrina]
sw10845	BGIBMGA000424	1.24705	1.2404	1.10325	0.24095	4.00E-65	gi|66526767|ref|XP_393136.2| PREDICTED: similar to ENSANGP00000013077 [Apis mellifera]
sw05816	BGIBMGA004752	1	1	1	0.2407	3.00E-37	gi|58396182|ref|XP_321723.2| ENSANGP00000015183 [Anopheles gambiae str. PEST] ENSANGP00000015183 [Anopheles gambiae str. PEST]

sw17616	BGIBMGA007089	0.77045	0.85595	0.971	0.24015	Bmb016749	2.00E-71
sw15360	BGIBMGA004992	0.8451	0.8981	1.29	0.2396	1.00E-05	gi|61103060|gb|AAX38000.1| hemomucin [Drosophila melanogaster]
sw13635	BGIBMGA001504	1.12	1.12335	1.30395	0.2385	No hits found	
sw07222	BGIBMGA008144	1	1	1	0.23675	No hits found	
sw13143	BGIBMGA013342	1.18895	1.01905	1.02575	0.2365	0	gi|2498144|sp|Q25490|APLP_MANSE Apolipophorins precursor [Contains: Apolipophorin-2 (Apolipophorin II) (apoLp-2); Apolipophorin-1 (Apolipophorin I) (apoLp-1)] apolipophorin precursor protein
sw08444	BGIBMGA000612	1.3354	0.9275	1.472	0.23565	e-115	gi|10959|emb|CAA41557.1| paramyosin [Drosophila melanogaster]
sw13134	BGIBMGA002930	1.093	1.2911	1.59235	0.23565	No hits found	
sw05530	BGIBMGA008541	0.79265	0.8124	0.719	0.23525	8.00E-46	gi|62858603|ref|NP_001016344.1| fasciculation and elongation protein zeta 2 (zygin II) [Xenopus tropicalis] fasciculation and elongation protein zeta 2 (zygin II) [Xenopus tropicalis]
sw06323	BGIBMGA008545	1	0.7348	1	0.2345	2.00E-17	gi|76635459|ref|XP_613108.2| PREDICTED: similar to leucine rich repeat containing 35 isoform 1 [Bos taurus]
sw22217	BGIBMGA006419	0.81425	1.0472	1.0895	0.2314	Bmb021298	0
sw06194	BGIBMGA010176	1	1	1	0.2297	No hits found	
sw16793	BGIBMGA011309	1	1	1	0.22775	Bmb047304	No hits found
sw22209	BGIBMGA009523	0.0677	0.1103	0.0681	0.22725	2.00E-87	gi|66551411|ref|XP_397170.2| PREDICTED: similar to Probable cytochrome P450 301a1, mitochondrial precursor (CYPCCCIA1) [Apis mellifera]
sw18890	BGIBMGA006878	0.9342	0.92215	0.9817	0.22635	0	gi|157955|gb|AAA28714.1| myospheroid protein
sw01135	BGIBMGA014337	1.53955	1.1553	1.2914	0.22395	e-119	gi|68357106|ref|XP_709875.1| PREDICTED: similar to Farsla protein isoform 5 [Danio rerio]
sw13036	BGIBMGA003516	1.1891	1.08965	1.18515	0.22275	1.00E-19	gi|28317136|gb|AAD27865.2| LD24380p [Drosophila melanogaster]
sw14074	BGIBMGA011844	0.842	0.88585	0.95495	0.2226	e-139	gi|13775331|gb|AAK39152.1| Protein disulfide isomerase protein 2, isoform a [Caenorhabditis elegans] Protein Disulfide Isomerase family member (pdi-2) [Caenorhabditis elegans] Protein disulfide-isomerase 2 precursor (PDI 1)
sw19121	BGIBMGA004808	1	1	1	0.22215	Bmb026871	3.00E-49
sw15337	BGIBMGA014548	1	1	1	0.2221	5.00E-58	gi|35505243|gb|AAH57652.1| Moxd1 protein [Mus musculus]
sw00881	BGIBMGA002620	1.34165	1.2698	1.02155	0.22115	2.00E-21	gi|8421|emb|CAA34861.1| Ref(2)P protein [Drosophila melanogaster]
sw22831	BGIBMGA001599	1.6685	0.8587	1.07935	0.2205	2.00E-13	gi|55234273|gb|EAA00192.2| ENSANGP00000008929 [Anopheles gambiae str. PEST] ENSANGP00000008929 [Anopheles gambiae str. PEST]
sw09738	BGIBMGA014601	1	1	0.62335	0.21655	No hits found	
sw06110	BGIBMGA003681	1	1	1	0.2148	3.00E-40	gi|48097111|ref|XP_391844.1| PREDICTED: similar to yes-associated protein 2 [Apis mellifera]
sw16010	BGIBMGA012196	1	1	1	0.21375	5.00E-11	gi|50732285|ref|XP_418563.1| PREDICTED: similar to adenosine deaminase, RNA-specific, B2; RNA dependent adenosine deaminase 3; adenosine deaminase 3, RNA dependent [Gallus gallus]
sw19706	BGIBMGA003454	1	1	1	0.21365	0	gi|73948859|ref|XP_544234.2| PREDICTED: similar to myosin IIIA [Canis familiaris]
sw13742	BGIBMGA001793	0.05995	0.65895	0.2155	0.2111	1.00E-16	gi|67930197|ref|ZP_00523372.1| Protein kinase [Solibacter usitatus Ellin6076] Protein kinase [Solibacter usitatus Ellin6076]
sw22032	BGIBMGA000598	0.38865	1.02975	0.39315	0.2107	No hits found	
sw17590	BGIBMGA003994	1	1	1	0.2101	4.00E-05	gi|66514408|ref|XP_394746.2| PREDICTED: similar to CG13643-PA [Apis mellifera]

sw20054	BGIBMGA005531	1	1	1	0.20965	No hits found	
sw14533	BGIBMGA005418	1.12305	1.07145	1.09505	0.20945	9.00E-50	gi|66561019|ref|XP_393495.2| PREDICTED: similar to DNA excision repair protein ERCC-6 (Cockayne syndrome protein CSB) [Apis mellifera]
sw18151	BGIBMGA012002	0.03485	0.26765	0.0202	0.20925	No hits found	
sw14501	BGIBMGA003854	1.254	0.72985	0.7266	0.2076	e-106	gi|55236115|gb|EAA14378.2| ENSANGP00000014757 [Anopheles gambiae str. PEST] ENSANGP00000014757 [Anopheles gambiae str. PEST]
sw10274	BGIBMGA008051	1	1	1	0.2038	Bmb049080	2.00E-11
sw01062	BGIBMGA003866	1.7359	1.70025	0.72395	0.20305	Bmb015651	0
sw11666	BGIBMGA002961	1.28165	1.24775	1.32565	0.1984	e-122	gi|83033256|gb|ABB97082.1| chitin synthase [Ostrinia furnacalis]
sw21834	BGIBMGA000388	1.2504	0.91105	1.15205	0.197	2.00E-26	gi|28380950|gb|AAO41442.1| RE39251p [Drosophila melanogaster]
sw07898	BGIBMGA011165	0.09145	0.09495	0.1129	0.19255	Bmb006912	5.00E-07
sw00791	BGIBMGA011479	1.02885	1.0462	0.7668	0.19225	Bmb011517	e-144
sw22899	BGIBMGA004474	1.44295	1.0727	1.3878	0.191	0	gi|82940307|emb|CAJ34654.1| chlorophyllide A binding protein precursor [Bombyx mori]
sw07281	BGIBMGA000788	0.51735	0.6741	0.4633	0.1898	2.00E-06	gi|18447174|gb|AAL68178.1| AT31946p [Drosophila melanogaster]
sw09703	BGIBMGA010573	1	1	1	0.18775	6.00E-47	gi|55243450|gb|EAA06323.3| ENSANGP00000021951 [Anopheles gambiae str. PEST] ENSANGP00000021951 [Anopheles gambiae str. PEST]
sw20915	BGIBMGA001976	1.03665	0.97425	0.8362	0.1866	1.00E-93	gi|66510575|ref|XP_392939.2| PREDICTED: similar to protein-glutamine gamma-glutamyltransferase (EC 2.3.2.13) - horseshoe crab (Tachypleus tridentatus) [Apis mellifera]
sw18903	BGIBMGA002462	2.334	1.98525	0.98925	0.18635	0	gi|45384004|ref|NP_990509.1| 5-aminoimidazole-4-carboxamide ribonucleotide formyltransferase/IMP cyclohydrolase [Gallus gallus] 5-aminoimidazole-4-carboxamide-ribonucleotide transformylase-IMP cyclohydrolase; ATIC
sw00215	BGIBMGA005270	0.8315	1.06925	0.93515	0.1859	7.00E-42	gi|56427966|gb|AAV91250.1| translocation protein 1 [Drosophila santomea]
sw18978	BGIBMGA009809	1.26105	1.1657	1.20845	0.17975	4.00E-08	gi|9630950|ref|NP_047547.1| AcMNPV orf150 [Bombyx mori nucleopolyhedrovirus] AcMNPV orf150 [Bombyx mori nuclear polyhedrosis virus]
sw19806	BGIBMGA000917	0.4702	0.9886	0.4897	0.17705	No hits found	
sw18858	BGIBMGA007208	2.77705	1.8736	1.22815	0.17355	4.00E-84	gi|76639351|ref|XP_870725.1| PREDICTED: similar to neurofilament, heavy polypeptide 200kDa isoform 2 [Bos taurus]
sw07856	BGIBMGA013818	1.1412	0.97095	1.13135	0.17245	2.00E-07	gi|26454107|dbj|BAC44437.1| DNA topoisomerase IV subunit A [Mycoplasma penetrans HF-2] DNA topoisomerase IV subunit A [Mycoplasma penetrans HF-2]
sw20215	BGIBMGA014116	0.44205	0.26285	0.44215	0.16365	7.00E-32	gi|51243505|gb|AAT99456.1| beta-N-acetylglucosaminidase isoform B [Bombyx mori]
sw07683	BGIBMGA007649	1	1	1	0.163	5.00E-68	gi|24641454|ref|NP_727583.1| CG2467-PB, isoform B [Drosophila melanogaster] CG2467-PA, isoform A [Drosophila melanogaster] GH09980p [Drosophila melanogaster] CG2467-PB, isoform B [Drosophila melanogaster] CG2467-PA, isofor
sw20536	BGIBMGA011896	0.4913	0.8532	0.67825	0.1505	No hits found	
sw03123	BGIBMGA003682	1	1	1.2979	0.14665	5.00E-59	gi|48097111|ref|XP_391844.1| PREDICTED: similar to yes-associated protein 2 [Apis mellifera]
sw16247	BGIBMGA007250	1	1	1	0.14545	Bmb039547	2.00E-67
sw17712	BGIBMGA005539	1	1	1	0.14205	Bmb041853	e-105
sw14648	BGIBMGA010419	1.1818	1.09295	1.1658	0.1354	3.00E-81	gi|2575865|dbj|BAA23126.1| BmP109 [Bombyx mori]
sw22025	BGIBMGA002386	1	1	1	0.1345	3.00E-99	gi|68989041|dbj|BAE06190.1| glycine rich protein [Bombyx mori]

sw13842	BGIBMGA010392	0.02785	0.18335	0.0366	0.1161	Bmb021115	2.00E-53
sw16501	BGIBMGA010765	1	1	1	0.11385	3.00E-15	gi|66500196|ref|XP_393508.2| PREDICTED: similar to ENSANGP00000003319 [Apis mellifera]
sw19437	BGIBMGA000159	1	1	1	0.1118	No hits found	
