# Supplementary material for: A Genome-Wide Survey for Host Response of Silkworm, Bombyx mori during Pathogen Bacillus bombyseptieus Infection
Source: PLoS One. 2009 Dec 1;4(12):e8098. doi: 10.1371/journal.pone.0008098 (PMC2780328; doi:10.1371/journal.pone.0008098)
Supplement: Table S3 — Multiple tissues expression data of the induced genes. (0.17 MB PDF) [file pone.0008098.s003.pdf]

Table S3

Multiple tissues expression data of the induced genes

probe	gene	3 h	6 h	12 h	24 h	Ovary	Testis	Head	Integument	Fat body	Midgut	Hemocyte	Malpighian tubule-F	Malpighian tubule-M	A/MSG-F	A/MSG-M	PSG-M	PSG-F
sw09154	BGIBMGA000068	0.4821	0.46355	0.76555	14.93285	114	125	143	-11	19	519	-8	599	773	151	148	23	52
sw03051	BGIBMGA009043	1.78365	1.2098	1.7273	2.68585	1467	24448	1748	5498	10981	17614	1199	8423	9813	140	147	131	138
sw06147	BGIBMGA009052	1.2195	1.03885	1.04735	2.22025	2799	1973	4569	2935	2469	3057	2469	7605	6449	4992	4598	3532	3779
sw15120	BGIBMGA002346	1.17395	1.14405	1.26515	0.4081	898	956	795	428	375	447	459	149	245	431	432	295	454
sw19688	BGIBMGA007361	1.41945	1.22995	0.99695	2.01845	248	181	441	460	443	1904	126	2471	2590	1642	2162	448	390
sw15014	BGIBMGA007408	0.8938	0.829	0.7816	3.244	3402	2820	7300	5035	2519	10889	2650	8214	9826	1800	1726	1085	1387
sw07985	BGIBMGA000419	1.628	1.7097	1.2878	2.13515	5831	3106	9397	17968	14115	47	59	7203	5346	1534	2178	1837	1195
sw08622	BGIBMGA000668	0.87785	0.9543	0.70695	2.14	1170	1576	1875	1164	980	1265	663	4124	3693	1783	1386	1581	2085
sw00791	BGIBMGA011479	1.02885	1.0462	0.7668	0.19225	330	607	322	433	913	275	190	340	566	168	250	257	236
sw19870	BGIBMGA002295	1	1	1	0.3637	316	882	284	343	151	255	79	174	224	413	450	533	515
sw13376	BGIBMGA001464	0.94405	0.9608	1.17395	3.02825	4060	5696	5892	4444	2820	6641	2374	4758	6776	4472	3211	2797	4728
sw22252	BGIBMGA010793	0.87065	0.78745	1	2.1977	76	246	1360	739	107	103	128	49	45	67	26	36	22
sw15671	BGIBMGA007787	0.3562	0.3586	0.3632	0.7351	242	890	176	222	82	623	417	139	205	130	241	73	88
sw18633	BGIBMGA001300	1.0431	1.022	1.08125	0.4539	1003	836	2162	1125	1167	1238	796	556	864	361	350	254	364
sw12437	BGIBMGA002767	1.1991	1.01905	1.23065	0.41135	233	1573	197	882	1085	238	16	50	61	40	65	20	8
sw03733	BGIBMGA008849	1.08825	1.03415	1.1158	0.3902	5164	5657	12213	8368	5029	9889	5655	5690	7542	4402	4498	3364	3872
sw16230	BGIBMGA002445	0.83335	1.2076	1.26985	3.1344	1452	820	376	285	246	253	211	155	191	484	692	730	686
sw16010	BGIBMGA012196	1	1	1	0.21375	1837	1066	997	728	697	589	317	296	570	338	487	440	418
sw05265	BGIBMGA001278	0.99985	1.1231	0.95765	3.6181	388	437	285	218	302	130	201	175	224	330	398	441	472
sw22913	BGIBMGA000807	1.31605	1.07965	1.35875	0.4656	37574	42925	48613	45017	21688	46696	48603	47756	67276	58092	56766	39475	45167
sw07062	BGIBMGA012939	0.45575	0.43955	0.6009	1.2498	389	386	131	188	114	213	84	51	65	329	310	1014	985
sw16450	BGIBMGA012894	0.6217	0.87235	0.45415	0.81065	114	40	163	349	226	9	34	3358	4900	191	206	177	208
sw21957	BGIBMGA000086	1.02405	1.2405	1.1237	3.57555	246	341	869	495	161	229	84	502	523	330	391	1074	1309
sw02976	BGIBMGA003178	0.51045	0.83475	1.232	1.61015	568	257	216	59	26	55	433	28	33	697	532	1069	1581
sw18603	BGIBMGA014265	0.98545	0.67695	0.7802	0.4522	127	135	3396	1868	88	57	18	948	1577	198	158	29	45

sw15430	BGIBMGA003464	1	1	1	0.40595	271	503	259	221	158	937	249	326	516	248	274	298	321
sw13806	BGIBMGA014209	1.0371	1.156	1.1833	2.39775	-10	-34	606	1149	34	2354	146	9806	19330	-15	83		-18
sw14501	BGIBMGA003854	1.254	0.72985	0.7266	0.2076	32	-5	1078	1855	183	30	20	855	2768	193	232	42	37
sw13873	BGIBMGA007726	1.08005	0.9678	0.9294	2.23555	2940	5862	3844	1981	1121	3608	1060	3635	5158	1436	1224	1349	1891
sw14006	BGIBMGA007332	0.71815	0.8286	1.0654	0.5015	953	1076	1461	861	478	808	824	377	910	440	467	791	821
sw08704	BGIBMGA007217	0.18045	0.5084	0.36645	1.43445	381	853	481	529	416	639	270	735	959	658	705	3018	2283
sw20393	BGIBMGA007769	1	1	1	0.49625	515	1282	225	169	124	66	220	132	210	68	76	194	236
sw12680	BGIBMGA005473	0.9568	0.905	1.06165	0.46365	1416	9983	1469	1342	1138	3343	1426	1173	1498	1096	1203	1507	1681
sw19088	BGIBMGA007869	0.84605	1.06885	1.2732	0.4225	2132	3729	1871	1530	1546	1652	1018	533	740	1397	1229	1396	2020
sw19450	BGIBMGA007218	1.5766	1.828	1.60905	0.42085	181	1250	165	189	146	1328	68	150	142	83	83	181	285
sw00905	BGIBMGA007738	1	1	1	0.32605	113	3799	349	191	51	60	52	55	18	434	617	3229	2596
sw10815	BGIBMGA003838	0.97085	1.018	0.67025	4.44385	1496	2790	647	597	847	732	674	1354	1049	934	937	814	723
sw21866	BGIBMGA006264	1.04065	1.0008	0.8121	2.82135	3184	2116	2752	2542	2544	6286	1183	6069	7319	1854	2398	1980	1977
sw08127	BGIBMGA013114	0.542	0.7798	0.8022	2.39615	1386	11355	1154	648	656	734	823	791	1033	985	1062	947	1127
sw11692	BGIBMGA006875	0.9063	1.05945	1.0775	2.3613	178	501	1073	727	601	1159	217	635	843	314	354	322	440
sw13079	BGIBMGA005673	2.05965	2.7684	2.0015	1.67305	97	365	91	94	41	5450	64	102	158	45	45	37	37
sw20421	BGIBMGA008633	0.37615	0.56785	0.47385	1.43765	152	437	241	179	72	98	131	121	145	112	113	87	112
sw22679	BGIBMGA012648	0.10245	0.7368	0.39975	1.31075	-26	47	-23	15	-8	-10	-13	21	34	1000	1569	115	87
sw22288	BGIBMGA004708	2.7738	1	1	1	252	440	349	1075	1700	-37	1022	27	37	16	35	14	13
sw00734	BGIBMGA005695	1	1	1	0.47045	399	392	216	1600	3185	51	57	106	131	55	59	46	55
sw22935	BGIBMGA002149	0.81375	0.86875	0.9751	0.46465	583	1748	985	491	329	1397	482	562	1021	399	381	581	582
sw14985	BGIBMGA000506	0.68965	0.9115	0.85555	3.2169	1376	5803	990	1054	743	1372	1148	1539	1594	1598	1670	1504	1664
sw06684	BGIBMGA002353	0.99935	0.9917	1.161	2.08535	4102	2093	3542	2203	2057	3631	2283	1508	2253	1571	1677	1389	1920
sw18185	BGIBMGA014217	1.0471	1.107	1.30895	0.49735	2129	2928	2488	2473	1070	7715	1351	8244	17551	1088	1051	797	1070
sw04603	BGIBMGA003390	0.7291	0.9446	0.9034	3.675	726	2435	1060	398	266	519	517	761	590	671	620	793	832
sw08771	BGIBMGA011824	0.69305	0.90815	0.7418	3.42335	5009	8906	6249	3998	3327	4467	4747	5335	4353	6102	4794	4688	5344
sw06503	BGIBMGA011204	1.03045	0.9386	1.02885	2.06245	815	3284	31292	6579	1372	1540	290	1328	1855	674	885	796	660
sw17947	BGIBMGA006176	2.3659	2.10905	1.58395	1.31595	100	-27	1577	1402	63	1174	-14	1219	2067	1180	1695	42	96
sw01980	BGIBMGA014369	1.6899	1.00735	1.31545	0.29835	17	41	74	61	39	1437	38	44	29	105	186	49	98

sw20350	BGIBMGA007121	0.92185	1.0731	1.08805	0.38225	4280	16658	30288	13935	2459	6179	1188	7560	9031	3672	3608	2789	3071
sw12837	BGIBMGA002083	0.9991	0.8748	0.67545	2.29445	750	13835	274	1009	584	-34	-26	88	72	-13	-11	3	9
sw15259	BGIBMGA001921	0.8379	0.93205	1.05495	2.17905	1432	2183	1385	899	627	2747	2428	1085	1017	920	770	706	1016
sw18276	BGIBMGA000236	0.75375	0.83945	0.65015	2.3043	310	156	277	299	614	1964	220	1025	1729	132	186	38	81
sw16215	BGIBMGA000715	0.68865	0.7152	0.7437	0.4961	982	881	3936	2265	1525	2431	761	1812	4286	2594	2595	867	1065
sw12830	BGIBMGA006977	0.59365	1.05935	0.97935	0.45155	339	311	1123	174	239	1053	176	502	870	119	193	123	188
sw20719	BGIBMGA001913	0.64205	0.6944	0.6419	0.24665	193	310	1360	500	272	5267	0	5151	6149	5330	6882	680	816
sw13511	BGIBMGA005615	0.66375	0.8502	0.7176	3.15825	517	733	513	338	520	397	477	533	628	739	826	633	859
sw15442	BGIBMGA002979	0.8267	1.1066	1.02635	8.68885	1255	1696	768	700	734	446	496	415	432	453	370	301	494
sw21920	BGIBMGA006855	0.94105	1.13475	0.8208	5.4299	389	1098	376	276	252	406	213	302	376	232	274	276	372
sw05953	BGIBMGA007258	1.1382	1.11185	1.11955	4.09205	338	1634	238	1242	2853	864	20	130	100	163	162	67	108
sw08933	BGIBMGA013364	0.8714	1.1236	1.14995	3.9358	1753	3194	824	767	399	475	789	957	963	949	742	628	935
sw05879	BGIBMGA005208	1.4507	0.8478	0.8166	3.8173	34	54	1683	2072	71	83	59	82	62	150	291	370	217
sw03726	BGIBMGA007259	0.8022	1.08195	1.0462	3.6712	1091	5792	748	5581	8409	2807	100	399	254	161	133	151	239
sw04453	BGIBMGA009033	1.10195	1.2061	1.01635	3.4918	25	25	48	28	-2	5145	19	44	62	21	35	19	20
sw11723	BGIBMGA008621	1.2601	1.21135	1.3012	3.44515	1304	713	614	546	757	678	469	854	720	1304	974	1075	1676
sw07293	BGIBMGA010193	0.6423	0.9298	0.8057	3.3647	555	399	803	576	197	523	391	961	1062	360	322	316	366
sw18213	BGIBMGA006083	0.7592	0.8457	1.0532	3.34255	144	99	2543	493	205	3534	15	335	344	36215	38657	3508	5631
sw08356	BGIBMGA002886	1.06135	1.1467	0.78055	3.15895	2153	1101	1209	1264	1480	1182	1297	3191	2960	1462	1447	1451	1379
sw21851	BGIBMGA013220	0.6794	1.38565	1.42155	2.75175	43	504	143	66	22	5104	20	121	122	116	97	46	48
sw12099	BGIBMGA002943	0.9217	0.92815	1.1606	2.56225	-5	2883	9	4	135	-10	-18	5909	7174	1340	1231	187	253
sw04364	BGIBMGA008096	0.7581	0.69685	0.95475	2.4095	1800	2396	1322	1823	3857	1869	247	7596	7936	5902	4787	2707	3232
sw22975	BGIBMGA008095	0.7365	0.74645	0.77405	2.39555	1661	1824	1542	2581	2516	3911	236	7074	6512	5376	4245	3912	4157
sw12660	BGIBMGA000813	1.24105	0.9184	1.39915	2.36435	775	912	813	497	635	735	462	596	1178	668	735	992	1041
sw06175	BGIBMGA002730	1.22005	1.227	1.23535	2.3577	593	920	240	197	327	2655	153	1577	2110	221	262	411	400
sw12097	BGIBMGA002944	1.12845	1.35575	0.48835	2.2824	40	28	337	200	85	76	122	8847	8898	34	36	36	43
sw14783	BGIBMGA002939	0.7981	1.1418	0.96835	2.135	167	342	258	555	1747	927	1	2859	4787	-11	-3		8
sw11627	BGIBMGA011108	1.43175	1.48205	1.2007	2.11715	130	230	664	580	476	3436	15	1255	1800	73	64	14	81
sw13775	BGIBMGA002945	1.28555	1.1993	1.07835	2.0768	363	228	277	1265	5452	9877	68	623	694	69	69	33	55

sw18990	BGIBMGA005827	0.8581	1.011	1.0819	2.06895	-23	-49	3138	110	3	-17	-20			41057	47006	5655	7710
sw22099	BGIBMGA005830	0.4342	1.049	0.8879	1.83205	-16	65	238	221	24	12	-8	31	8	28017	31605	11367	8668
sw07516	BGIBMGA007912	0.933	0.94945	0.9173	0.47265	761	682	1368	747	749	338	293	778	1218	480	418	434	557
sw19113	BGIBMGA013161	0.932	1.04925	1.02065	0.4139	820	802	1102	872	454	416	487	559	997	556	576	489	508
sw16738	BGIBMGA005505	0.8071	1.3127	0.94295	2.2469	428	713	605	1063	2173	3478	264	2212	2586	241	279	170	148
sw11720	BGIBMGA007100	0.9727	0.867	0.80195	2.16975	35	24	125	20	7	3065	-20		3	9	38		4
sw01796	BGIBMGA012009	1.2347	0.9317	0.9907	2.0652	566	1081	924	664	474	688	261	1075	958	658	621	488	470
sw09590	BGIBMGA007098	1.172	1.11775	0.8985	2.02955	6357	3132	8370	8682	6218	2753	5077	3875	3233	3331	3178	1248	1346
sw07039	BGIBMGA008873	0.7184	0.9003	0.85165	0.43815	367	3952	530	259	168	209	261	236	302	281	416	436	390
sw01149	BGIBMGA006032	1.38115	0.97885	1.10795	2.15705	74	2410	2374	1846	84	666	53	121	114	158	170	63	106
sw20745	BGIBMGA000364	0.9692	1.0694	0.986	3.9094	957	1102	1900	814	335	1082	355	727	1183	294	401	362	413
sw09911	BGIBMGA002406	0.95795	1.27435	1.10525	2.3022	1586	1675	1324	1261	1633	7960	1425	1731	2417	1595	1978	2297	2847
sw09712	BGIBMGA010403	0.41895	0.53645	0.56755	0.91375	1790	8601	776	1147	3055	65	326	66	109	932	1369	25	149
sw17432	BGIBMGA001402	1.21845	1.12715	1.307	0.42915	1399	1930	2862	3982	10002	2813	839	3913	7053	840	1096	1768	1940
sw16033	BGIBMGA014453	1.93	1.14875	0.78355	3.923	8848	681	195	10	82	34	31	60	33	14	17	4	18
sw20657	BGIBMGA009800	1.07005	0.86935	0.871	2.50665	10	-29	113	545	1861	171	-15	12	21	11	21	58	52
sw20846	BGIBMGA012268	0.8286	1.0328	0.8875	2.3856	5953	734	717	683	415	1150	904	211	217	45	7	25	13
sw22929	BGIBMGA001351	1.8716	1.08665	1.16855	2.09995	5665	1759	5875	23832	23657	151	50	1861	2839	212	130	146	364
sw03637	BGIBMGA009232	1.4407	1.0586	1.0158	2.0473	120	92	141	526	1459	1197	202	1271	1765	36	50	35	37
sw18399	BGIBMGA008824	2.293	4.27355	3.3477	0.9437	3	27	-6	27	30	8735	13	42	116	19	17	8	46
sw03742	BGIBMGA007424	0.93305	1.00635	1.45905	2.84965	72	145	2590	1237	114	25	98	108	121	1304	1495	493	799
sw21727	BGIBMGA007987	1.1756	1.15925	1.0606	4.88565	-20	-41	-8	61	55	15455	8	44	56	8	3	27	23
sw20314	BGIBMGA012866	1.54545	0.83625	0.338	3.50685	6354	1692	2175	13080	12114	6066	369	2589	954	168	172	193	239
sw01725	BGIBMGA006812	1.106	1.0812	1.1884	2.36345	311	145	177	582	739	53	225	103	53	60	74	74	67
sw05330	BGIBMGA005717	0.38705	0.60445	0.4684	0.43805	377	746	1071	610	502	488	356	802	945	403	387	354	352
sw13398	BGIBMGA002540	0.92545	1.0469	1.0954	3.6243	2109	3872	618	418	311	608	421	779	981	1097	924	428	717
sw02528	BGIBMGA003564	0.72985	0.6492	0.8163	2.1418	2126	3146	1025	210	78	77	149	84	71	648	583	397	752
sw03623	BGIBMGA001237	0.93125	0.825	0.8514	0.40505	289	831	642	596	1331	381	114	351	1141	79	59	68	69
sw14459	BGIBMGA007146	1.3606	1.47215	1.1077	3.33585	1	-36	-1	43	23	156	-18	31201	51931	-19	-17		-10

sw05795	BGIBMGA006185	1.6475	1.45195	1.30615	0.46655	60	305	917	377	475	1220	-5	26	22	1172	1240	222	328
sw03379	BGIBMGA006518	0.7941	0.67895	0.77895	0.3648	395	530	516	264	348	147	116	286	691	104	90	133	351
sw20315	BGIBMGA012740	0.8417	0.76145	0.74415	0.2668	-1	21	2876	224	255	-3	-2	31	37	36443	50068	2470	7245
sw15849	BGIBMGA008868	1.21075	1.1895	1.0667	3.91485	1762	1125	996	872	642	1398	366	795	874	936	790	582	883
sw11603	BGIBMGA011856	0.96625	1.02235	0.83465	3.41155	3175	1935	2190	1244	1231	1323	1347	1312	1143	1735	1623	1526	1519
sw16392	BGIBMGA002349	0.84825	1.02495	0.77275	2.087	2544	1596	5111	5655	7546	15613	13942	62516	51045	500	603	248	292
sw12210	BGIBMGA000948	1.0209	1.18395	0.74875	3.59435	1094	2004	1026	682	424	559	728	296	217	422	530	442	499
sw15293	BGIBMGA007878	1.0656	1.2752	0.9476	3.9297	2026	1647	1545	1405	418	1259	769	866	1098	876	1024	995	1115
sw12031	BGIBMGA007005	1.02945	0.90165	0.89675	2.6802	9480	9103	5681	6730	5911	11722	4668	7202	7650	9310	7827	7289	8994
sw14740	BGIBMGA012136	1.2669	1.11485	1.26335	0.42655	568	266	908	705	246	1147	259	99	106	149	122	126	153
sw00477	BGIBMGA010943	0.95155	1.0448	0.8655	0.31715	588	877	412	310	279	306	336	342	447	280	300	341	354
sw20523	BGIBMGA000150	1	1	1	0.2688	767	3864	40	49	53	3	-1	63	37	682	955	1522	1001
sw19964	BGIBMGA004337	2.0586	1.40475	1.18085	1.1554	660	1684	1034	438	449	1360	496	527	950	471	652	631	555
sw09544	BGIBMGA007716	1.2122	1.0999	0.9352	2.15855	1666	6896	219	2048	6348	-18	-7	39	42	16	39	23	42
sw16574	BGIBMGA004976	1	1	1	0.4916	719	798	325	306	249	154	145	107	148	390	479	384	383
sw20970	BGIBMGA006959	0.8505	0.84895	0.92085	0.33395	12967	8120	3967	4232	3195	3533	3284	1257	1558	3611	3605	3741	3392
sw03443	BGIBMGA010459	1.02285	1.1099	1.401	2.657	66	2671	137	42	29	1008	32	30	48	123	71	28	38
sw13748	BGIBMGA001943	0.8259	1.11345	0.932	3.44715	2681	5884	3565	2449	1987	5611	3126	2671	3248	3247	3999	3866	3885
sw04526	BGIBMGA007025	1.2407	0.98165	1.0806	3.33975	76	307	287	135	150	1229	167	257	357	15	28	44	28
sw22048	BGIBMGA013200	0.7698	1.0694	1.07635	4.27535	2524	6529	1363	1076	1072	1479	953	906	893	2097	2126	2288	2500
sw04717	BGIBMGA013458	1.0529	1.0996	0.9505	2.26815	1647	1511	2866	1125	683	1185	697	2170	1697	998	1015	1231	1314
sw19086	BGIBMGA014542	0.0775	0.4695	0.7655	0.80205	145	15959	44	38	22	11	20	73	61	12	13	15	16
sw07485	BGIBMGA009929	1	1	1	0.47695	14	9767	19	31	31	24	-17	11	7	-6	8	11	15
sw17805	BGIBMGA002747	0.56795	0.4119	0.5943	1.85365	4416	4992	1036	7224	14026	458	248	827	554	85	76	86	92
sw05946	BGIBMGA006585	1	1	1	0.38265	0	4375	-1	-10	10	-15	-13			-5	-8		-1
sw11317	BGIBMGA002073	2.0433	1	1	1.31415	20	166	2030	679	55	36	15	41	67	9	27	7	6
sw13495	BGIBMGA002543	0.8842	1.44845	1	0.50705	256	244	324	505	399	509	317	476	527	239	342	269	234
sw12176	BGIBMGA008312	0.80895	0.75175	0.999	4.00105	555	440	533	380	475	680	403	898	934	603	434	447	697
sw15960	BGIBMGA006158	0.9567	0.90485	1.1805	2.28965	6257	4994	6198	6107	5376	12040	4445	11394	10341	8676	6746	5608	8825

sw10855	BGIBMGA007875	0.9541	0.9469	1.2003	0.45395	3759	5954	2560	1789	1194	1838	1678	1401	1754	2432	2187	1722	2174
sw18770	BGIBMGA012376	0.95855	1.00605	1.0005	3.71535	1369	848	695	428	268	283	487	72	53	680	666	429	474
sw15929	BGIBMGA013069	1.10575	0.99185	1.06605	2.3519	1874	738	1808	1212	876	751	680	682	713	1276	1559	1570	1261
sw10585	BGIBMGA006143	1	1.01855	1	2.33505	667	284	456	479	312	122	469	234	300	534	378	408	401
sw13482	BGIBMGA000563	2.65235	2.2217	1.0097	0.66365	104	145	22196	9011	306	449	89	645	84	53	62	27	35
sw01062	BGIBMGA003866	1.7359	1.70025	0.72395	0.20305	1254	798	3179	1369	3315	138	365	155	166	119	97	90	162
sw00316	BGIBMGA003815	1	1.08595	1.22755	2.01795	452	693	3857	1362	548	1393	339	1380	1632	579	626	779	772
sw03909	BGIBMGA008211	1.17635	0.95555	1.1138	2.1587	1766	4227	3104	2269	986	3567	1257	2091	2264	2290	2120	1368	2206
sw12672	BGIBMGA003645	4.66495	1.6954	2.0591	1.1087	220	325	158	408	1140	471	173	370	884	50	59	53	64
sw14711	BGIBMGA007955	1.32535	1.44	1.63805	3.6877	1467	2429	770	700	1018	994	1047	607	461	2007	1484	1846	2932
sw09307	BGIBMGA008931	0.34445	0.65835	0.4997	0.32625	842	842	3336	1732	2596	1611	1554	2440	1273	720	589	642	571
sw14758	BGIBMGA007642	0.9575	1.17845	1.0088	4.71	1783	4677	2344	1700	1014	1214	1211	1569	1271	1597	1668	1753	1962
sw21738	BGIBMGA009907	0.7359	0.8809	1.16655	2.10295	924	1768	680	2905	3948	4183	23	12545	10991	28	22	4	24
sw00299	BGIBMGA007734	1.02835	1.1505	1.25265	3.51875	2128	2730	1558	1215	1093	1418	1646	1080	1283	2748	2582	2919	3815
sw12526	BGIBMGA005140	1.08675	0.9006	1.04735	0.49515	1075	1584	3798	1978	313	890	1083	364	513	545	410	357	382
sw08031	BGIBMGA003874	1.16975	1.07725	1.30215	0.45015	338	165	1517	434	347	383	100	328	626	260	343	518	399
sw18575	BGIBMGA006415	1.20835	0.99925	0.9803	0.42875	7628	3949	14118	15152	7591	13877	6977	6414	9452	9916	9938	4309	4054
sw08752	BGIBMGA000157	1.2406	1.11285	0.95245	2.10705	1840	1901	1392	1224	1378	1203	1333	1070	1302	1387	1495	1254	1361
sw11361	BGIBMGA012068	1.22505	0.82295	1.05535	2.4977	-17	-32	80	63	13	953	-28	20	2	37	4		5
sw08237	BGIBMGA011482	1.44795	0.834	1.1394	0.46795	1822	2241	1726	1985	681	359	104	3012	5316	1008	1019	842	991
sw09134	BGIBMGA012483	1.2189	0.9463	1.1453	0.40885	3303	1170	5081	3350	262	1154	4940	2106	2220	1050	1603	1735	1379
sw08046	BGIBMGA003752	1.02055	1.11205	1.12855	0.40635	1332	1652	732	564	340	1002	361	411	713	250	202	233	301
sw15106	BGIBMGA000146	0.9798	1.0924	1.1015	0.4037	2103	1865	9719	4979	1218	3280	3291	1304	2018	2118	2559	350	436
sw11270	BGIBMGA009303	1	1	1	0.24745	224	824	434	31	9	-65	945			-13	-10		-33
sw20305	BGIBMGA004592	1	1.01145	0.9083	0.32445	696	786	1589	978	469	1473	370	530	893	915	1002	963	1104
sw17156	BGIBMGA000931	0.99775	1.12355	0.9198	2.6357	1597	2675	1677	1237	1036	1498	1174	870	904	2083	2353	1765	1784
sw15939	BGIBMGA001217	0.9121	1.05805	0.96765	2.41805	2864	11862	2510	1760	1109	1504	1091	1639	1669	1879	1969	1593	1684
sw13036	BGIBMGA003516	1.1891	1.08965	1.18515	0.22275	901	2119	2247	1185	307	2547	1695	475	882	407	331	363	549
sw14240	BGIBMGA003515	1.24425	1.13395	1.15845	0.4056	358	661	735	319	95	616	420	158	304	159	108	141	198

sw06018	BGIBMGA002647	1.2126	1.2699	1.11765	0.2744	420	433	1802	807	753	121	147	451	934	150	183	148	162
sw21120	BGIBMGA005931	1.3421	1.0924	1.5224	0.4868	384	529	747	639	140	1594	33	1598	3642	321	311	270	319
sw20346	BGIBMGA007678	1.5597	0.8628	1.3605	3.63995	2152	1218	16044	16632	196	321	12	211	421	440	685	68	101
sw10798	BGIBMGA007677	1.92275	1.15735	2.6117	1.87125	928	281	23170	27275	316	426	22	275	608	1071	1011	127	246
sw01141	BGIBMGA007900	0.4986	0.83625	0.667	1.3698	43	61	2005	877	13	90	78	93	78	134	116	80	127
sw02159	BGIBMGA009641	2.31815	1.3921	1.40145	0.5581	80	92	160	159	62	3451	78	109	98	88	67	89	100
sw13635	BGIBMGA001504	1.12	1.12335	1.30395	0.2385	241	255	177	68	36	12495	149	105	157	158	139	149	159
sw18978	BGIBMGA009809	1.26105	1.1657	1.20845	0.17975	23	-4	20	169	-2	6693	-11	2	13	-9	3		4
sw08164	BGIBMGA012313	1.1867	1.279	1.20485	0.3811	929	1766	812	916	603	625	330	391	533	262	321	247	280
sw10803	BGIBMGA012262	2.1921	1.13055	0.76535	2.78335	462	296	106	907	1061	1635	-5	105	75	0	-17	3	6
sw20178	BGIBMGA012263	2.46505	0.9876	0.54905	1.96755	-7	43	7	60	-6	3285	-11	39	46	2	3		-8
sw20883	BGIBMGA012265	2.34115	1.1885	0.92425	1	211	127	46	155	65	1944	109	107	102	278	200	134	237
sw11640	BGIBMGA013439	0.316	0.6005	0.4431	0.25285	521	143	436	143	54	320	12	209	284	52	74	499	316
sw21459	BGIBMGA000039	0.7354	0.1754	0.29065	3.3526	118	165	161	414	603	91	54	97	88	51	62	41	53
sw02176	BGIBMGA001201	0.6989	0.4896	0.7828	0.84025	243	253	31242	42573	4547	12357	184	755	533	327	496	192	256
sw12863	BGIBMGA010210	0.7222	0.96735	1.013	2.53605	1383	2372	1634	1555	1064	1461	968	1233	1372	2315	2394	1770	2186
sw14910	BGIBMGA009706	1.07145	0.9293	0.91015	3.05515	1932	1449	9487	3323	1885	5320	1708	8160	8326	2663	2779	2919	2981
sw05062	BGIBMGA005455	1.12675	0.96825	0.934	2.55115	3548	18400	26129	10580	5561	32250	4826	16749	16947	5936	5108	4867	7140
sw05584	BGIBMGA009067	1.1001	1.34675	1.27775	2.2997	5	6	19	11	8	15783	41	248	29	23	25	38	36
sw10647	BGIBMGA000281	2.9171	1.47705	1.1584	6.53775	-7	-17	65295	2254	-2	-24	13	72	84	448	307	5	12
sw17771	BGIBMGA003095	2.5833	1.3329	1.97195	4.23325	-22	-46	31280	8517	4	1350	8	36	43	3	-12		-15
sw22684	BGIBMGA000276	2.0627	1.6147	1	3.90655	133	178	16395	1281	142	217	101	234	290	269	229	86	117
sw00595	BGIBMGA003062	1.8259	1.33625	1.12435	3.6383	78	310	91032	6191	178	299	312	170	127	145	88	52	88
sw00137	BGIBMGA010231	3.29465	0.5963	1.481	3.02175	41	78	1126	116	25	104	136	253	113	13525	10836	248	2246
sw13351	BGIBMGA000332	1.44705	0.6839	1.02845	2.9922	19	27	1203	7466	98	54	-5	20	30	26	25		-17
sw12206	BGIBMGA000338	1.2652	0.6625	1.1206	2.97355	102	197	10955	29593	442	645	69	138	199	308	308	136	111
sw00738	BGIBMGA000339	1.1584	0.9385	0.5097	2.8127	21	46	2127	1921	14	127	42	96	89	134	114	136	122
sw17943	BGIBMGA003065	1.5741	0.5357	0.66185	2.80295	151	130	72060	31933	305	134	41	223	284	321	291	89	104
sw11846	BGIBMGA000250	1.3523	1.39345	1.40135	2.63295	-2	22	5946	10132	23	1423	8		51	-1	6		3

sw03003	BGIBMGA010141	0.78245	0.97275	1	2.56215	489	1044	908	334	188	1216	207	255	239	241	184	153	227
sw00139	BGIBMGA010142	1.33735	0.85485	1.02335	2.3804	110	321	11486	1382	124	233	126	150	113	345	248	67	79
sw02931	BGIBMGA000282	0.7687	0.88885	0.6396	2.1551	412	218	1545	721	399	217	397	1226	929	395	388	512	505
sw19244	BGIBMGA000269	1.6252	0.66225	0.91305	2.07955	53	268	7804	1672	113	-23	6	30	29	149	133	15	16
sw13350	BGIBMGA000331	3.0576	0.79175	1.4101	2.0093	206	409	1196	16958	153	192	447	234	247	100	128	140	167
sw05302	BGIBMGA000336	1.95845	1.678	2.1477	1.15905	722	418	29023	45536	3548	10344	97	4309	11488	4160	4408	1882	2065
sw07086	BGIBMGA008333	2.6456	1	1	1	1	29	29160	1102	28	-25	-6	13	13	-2	-12		9
sw14431	BGIBMGA013163	1.3623	1.0402	0.8759	0.4687	83	62	14919	14202	218	237	27	425	481	224	391	42	39
sw20009	BGIBMGA010482	0.2766	0.7087	0.47965	0.42215	180	332	6330	807	1374	206	48	2320	278	1318	799	504	409
sw13734	BGIBMGA011364	0.88665	1.00215	1.04925	0.3496	422	1241	627	372	380	355	311	787	1718	188	229	128	137
sw17936	BGIBMGA000672	1.323	1.14345	1.3928	0.3359	4862	15354	28916	11649	6697	29888	11695	12115	23137	5524	6677	10970	8032
sw01563	BGIBMGA009760	0.5754	0.7759	0.8862	2.6227	983	1709	863	797	1086	1257	921	1194	1121	1734	1429	1747	2197
sw22924	BGIBMGA001060	0.7506	0.8669	0.79815	2.13015	17428	15016	16403	17320	11643	25529	19660	17213	20240	15181	12816	10207	10995
sw07960	BGIBMGA012935	0.9125	0.9697	0.9268	0.2465	2038	3184	1610	822	775	1214	1207	538	1191	871	915	812	891
sw08984	BGIBMGA010658	0.9033	1.0095	0.9799	2.26085	1105	2121	1064	641	511	2285	657	1353	2794	488	531	704	845
sw14040	BGIBMGA001536	0.8994	1.15275	0.94105	2.5227	3650	3272	1984	2459	4197	1131	1683	4419	3712	2220	1995	1528	1932
sw22410	BGIBMGA006052	0.6745	0.5745	0.46885	1.7041	367	232	795	1605	1170	7	-2	361	146	0	11		22
sw08056	BGIBMGA002243	1.11865	1.17655	0.94905	2.89105	1660	6957	2418	1209	1194	1496	1269	1463	1161	1300	1987	1774	1396
sw21466	BGIBMGA000837	1.5345	1.15445	1.46355	7.79595	-15	-40	39	100	13	2334	5	123	140	7	2	1	9
sw16042	BGIBMGA004870	1.10735	1.25075	1.2345	3.12435	16	-31	-19	48	18	20436	17	10	30	-9	-18		-15
sw22224	BGIBMGA014599	1.6495	1.45385	1.63975	2.69265	10989	5177	4975	7051	8300	4443	4871	4297	4511	9147	10746	10848	10551
sw07946	BGIBMGA000875	1.1881	1.3617	1.47805	2.4811	12	17	28	37	-7	1620	30	49	63	67	62	52	51
sw15265	BGIBMGA001964	0.95475	1.2052	1.05325	2.3975	156	429	67	176	921	16097	28	695	898	49	79	17	31
sw11949	BGIBMGA007545	0.84555	1.2806	1.6103	2.03015	33	96	60	45	41	841	-16	17	22	41	25	47	68
sw13618	BGIBMGA000777	0.8499	0.9974	1.09255	2.01735	1469	1051	1079	844	663	975	871	1074	1059	1499	1604	1442	1508
sw08854	BGIBMGA010328	2.7317	1.72955	1.1654	1.49245	17	1076	194	52	16	872	30	908	1504	198	232	27	28
sw06237	BGIBMGA004229	1.48985	1.6544	2.36655	1.3332	175	63	3475	1657	26	218	234	158	121	202	166	170	206
sw07726	BGIBMGA010976	0.2541	0.4517	0.34085	1.22745	60	69	62	63	75	51	52	99	72	377	765	1159	968
sw14035	BGIBMGA000772	0.22435	0.4665	0.7387	0.46045	492	139	610	5479	5898	709	3	81	247	208	112		-5

sw03795	BGIBMGA000776	0.70705	0.5776	0.9456	0.4393	29	-9	1873	136	48	10	4	47	19	28316	31202	3838	6150
sw22790	BGIBMGA002170	0.91875	0.82795	0.72375	0.4117	1518	1837	1719	2080	1021	2239	1226	2107	3140	1301	1453	1062	1124
sw20886	BGIBMGA013812	0.4982	0.691	0.68365	0.3694	170	143	7544	17259	122	47	4823	59	54	8643	12202	539	515
sw14169	BGIBMGA014214	0.4841	0.7689	0.8131	2.12525	1158	6255	1521	1668	1381	3244	5925	929	1109	129	122	103	108
sw15888	BGIBMGA004729	1.0586	1.06315	1.26525	0.43645	278	222	544	320	192	520	162	140	202	182	148	188	242
sw21834	BGIBMGA000388	1.2504	0.91105	1.15205	0.197	222	372	1208	2224	152	143	49	458	1082	113	139	114	119
sw14986	BGIBMGA007019	1.00825	0.94285	0.95315	2.2761	3314	4864	3944	3033	1829	4401	1707	8496	7362	2754	1921	1839	2425
sw19416	BGIBMGA007811	0.02825	0.45855	0.52315	1.02945	649	41440	42	47	18	21	15	69	18	27	12	9	7
sw03184	BGIBMGA004650	0.08115	0.5321	0.45785	1.47155	646	21028	41	71	33	53	21	81	58	74	74	74	73
sw05196	BGIBMGA009700	1.2477	1.08665	0.976	2.3236	274	3554	266	112	158	46757	196	269	344	76	119	46	58
sw12116	BGIBMGA012032	0.83225	1.0832	1.1484	2.2123	3133	11403	1759	2880	3777	4407	3033	6927	5126	3119	1733	1550	3074
sw04521	BGIBMGA011508	0.74295	0.94105	1.0988	2.36555	807	1160	469	609	381	646	540	701	778	1013	708	787	1399
sw19341	BGIBMGA007497	1.0785	0.8713	1.1468	0.4987	312	453	182	334	697	1030	76	1039	1766	112	118	77	114
sw01486	BGIBMGA013268	0.74455	1.0512	0.8324	3.99825	87	183	11847	7615	182	36	29	75	52	59	48	67	103
sw20320	BGIBMGA005955	0.7528	1.07655	0.9219	3.20585	60	-20	167	55	35	2410	21	2231	1968	105	125	165	185
sw22950	BGIBMGA000940	1.1445	1.19465	0.926	2.9866	2022	2131	1229	250	361	33	35	139	85	4464	5871	4770	3942
sw22686	BGIBMGA005953	1.2381	1.1816	1.1779	2.0831	15	434	27	53	9	15858	16	70	66	10	13	4	10
sw06412	BGIBMGA003032	0.4515	0.8195	0.7819	0.7704	3772	1312	8393	2718	862	1709	2256	618	458	5339	3205	948	2872
sw01110	BGIBMGA003129	0.8711	1.2437	1.73625	0.479	691	600	213	504	959	1925	164	62	82	24	49	26	58
sw07146	BGIBMGA007739	0.8679	0.94455	1.1636	4.54955	1400	2047	1333	527	488	682	693	224	256	343	233	245	601
sw16472	BGIBMGA010577	1.323	1.2836	1.13815	4.4428	2247	2815	1580	1589	822	757	1169	529	466	1368	1167	1000	1353
sw15610	BGIBMGA006376	0.6895	0.76355	0.87075	3.8069	7449	19969	11951	12285	7235	11815	9660	7364	6641	9028	6585	5362	7498
sw04574	BGIBMGA007972	1.13345	1.1866	1.1417	2.8245	514	587	360	283	248	360	313	358	356	455	357	431	613
sw09113	BGIBMGA011813	1.06015	0.95365	1	0.4621	90	533	145	125	66	142	109	194	95	82	100	45	54
sw08742	BGIBMGA005508	1.18395	0.9792	0.93645	0.45715	1796	1928	1660	1298	939	2995	1048	2851	4910	1162	1278	1089	1345
sw07281	BGIBMGA000788	0.51735	0.6741	0.4633	0.1898	3868	4371	1979	1085	140	9975	2691	491	1031	681	885	914	1105
sw06150	BGIBMGA011959	0.3919	0.9204	1	1.0985	485	178	200	992	790	72	36	319	75	16	38	23	45
sw16308	BGIBMGA009674	1.7167	1.18	1.1083	0.4065	5807	5951	6054	3929	2820	3180	2313	3366	3144	3179	3808	3629	2694
sw17594	BGIBMGA013548	0.76825	0.91825	1.05425	0.3549	1886	17437	1054	840	583	702	510	530	614	1122	945	860	938

sw19621	BGIBMGA014011	1	1	1	0.27085	72	33	681	155	35	294	-12	222	513	8	5	10	33
sw09007	BGIBMGA014563	1.2087	1.2125	1.28455	3.44805	696	375	144	111	205	207	175	78	97	395	452	739	699
sw16561	BGIBMGA012567	0.888	1.03705	0.9021	2.2345	1024	3687	1301	949	855	1212	700	558	608	830	727	780	896
sw16074	BGIBMGA008956	0.9734	1.23915	0.90935	2.14855	2693	4481	2055	2532	2973	1213	1280	2328	2643	1822	2077	2471	2640
sw13830	BGIBMGA004023	0.7419	0.8781	0.9364	3.31335	5559	11211	2361	2312	1708	1850	3909	2683	2139	5413	3748	4323	5724
sw08766	BGIBMGA011338	0.94795	0.9728	1.0491	3.09535	1005	824	653	536	528	676	723	595	834	716	738	772	1073
sw04389	BGIBMGA008671	0.9382	1.17755	0.89555	2.5121	342	1163	185	129	67	146	166	160	149	134	159	155	238
sw12378	BGIBMGA001142	1	1	1	0.45815	1148	2785	476	283	170	123	470	285	531	256	268	204	244
sw11733	BGIBMGA001790	0.75355	0.85565	0.9777	0.3393	309	1467	415	216	119	262	187	141	290	272	310	239	295
sw12652	BGIBMGA006161	0.90145	0.90345	1	0.4344	4732	4719	3271	2655	1742	2805	1989	3733	6681	3525	3895	3669	3977
sw17297	BGIBMGA006604	0.87295	0.9047	0.86655	2.935	539	745	678	542	362	510	448	575	790	424	529	723	644
sw09668	BGIBMGA003014	1.03705	0.82735	0.67155	2.5296	1203	301	1076	2361	3608	470	310	580	667	39	51	2	45
sw16069	BGIBMGA008416	0.89735	0.8564	0.9172	2.412	1351	3296	1480	1136	720	3547	1049	1902	3583	1213	1093	1100	1432
sw11800	BGIBMGA008749	1.3631	1.06635	1.1529	2.11235	803	389	1940	568	43	30	121	34	68	92	88	50	68
sw16412	BGIBMGA009964	1.12705	1.17355	1.3214	5.8593	1186	4111	2239	872	367	1804	525	552	655	485	526	496	560
sw04870	BGIBMGA008492	1.0187	1.80415	1.7541	3.17275	-5	-1	37	33	29	19848	40	42	95	7	9	10	9
sw15825	BGIBMGA009012	0.79415	0.81475	1.02315	2.32895	4537	14011	60693	32498	5470	32933	5734	8021	7290	4638	2952	5432	6709
sw18227	BGIBMGA008049	0.9308	1.04315	1.4078	2.1396	818	-1	73	52	34	497	132	209	634	67	77	644	696
sw14956	BGIBMGA005943	0.8959	0.95345	0.8299	3.5745	2299	3651	2989	1717	1491	2306	836	4108	4899	2164	1955	2139	2404
sw17034	BGIBMGA009262	0.8918	1.0836	1.1378	5.77555	664	594	498	337	351	244	328	408	446	967	752	738	1098
sw17190	BGIBMGA011501	0.74675	1.07105	0.812	2.64555	3633	7023	4366	4042	2636	3038	2403	1513	1684	3347	3053	2838	3149
sw18672	BGIBMGA013624	1.09625	1.06015	1.25025	2.183	3384	4817	2743	2415	2569	5591	1653	2329	2409	3991	2876	2868	4124
sw15446	BGIBMGA014522	1.06435	1.40155	1.1249	2.3851	616	857	556	404	290	501	371	454	484	556	543	447	620
sw15536	BGIBMGA013340	0.8645	1.1495	1.0016	2.3627	2825	2677	1413	1819	2368	1662	1306	1639	1117	3210	2295	2328	3502
sw06218	BGIBMGA011887	0.90465	0.9005	0.9633	2.26015	1711	2797	872	584	773	588	804	728	917	1002	1046	1313	1616
sw06899	BGIBMGA009307	0.79465	1.0541	0.8801	3.9246	1680	4327	2880	1441	1099	1454	1593	1314	1147	1220	1189	1102	1239
sw20207	BGIBMGA000305	0.73605	0.9895	0.8516	2.5966	723	621	1562	1639	827	558	177	555	464	1273	1123	1127	1414
sw00926	BGIBMGA005991	1.2557	1.1018	1.15155	4.0685	950	969	248	133	153	223	348	145	135	412	279	322	572
sw17940	BGIBMGA006931	1.2198	1.31025	1.2858	4.269	2181	2469	580	441	617	334	660	436	506	1105	1168	1621	1548

sw01709	BGIBMGA010387	0.74205	1.03645	0.91	3.64905	687	688	800	368	221	88	718	139	79	345	301	267	374
sw07225	BGIBMGA012569	0.951	1.1153	1.0194	2.87355	1156	1224	637	523	385	710	419	594	815	906	834	1004	1273
sw14703	BGIBMGA011314	0.8734	1.27365	1.17655	2.34955	2229	3639	1583	1773	1961	4273	1403	1436	2334	1827	1506	1726	3123
sw01081	BGIBMGA002821	1	1	1	0.41935	137	1887	162	117	61	330	155	239	239	196	162	159	209
sw03654	BGIBMGA003153	0.92385	1.06795	1.1794	2.83825	4552	10548	4767	7814	7563	20843	5135	3797	4454	11907	4439	4421	9651
sw03560	BGIBMGA002721	0.83235	0.98635	1.00825	2.3436	470	1955	354	189	235	136	353	163	143	394	272	241	448
sw09973	BGIBMGA003909	0.81455	1.07925	0.8694	2.17955	574	330	463	290	133	273	308	473	515	418	326	390	603
sw18111	BGIBMGA005360	1	1	1	0.4622	191	239	429	653	93	128	66	232	344	348	260	178	208
sw04971	BGIBMGA011776	0.6742	1.02205	0.93225	2.40225	7935	7102	8709	9030	4525	7274	6423	3814	3351	9147	7364	5657	8892
sw04986	BGIBMGA004441	1.2459	0.964	1.1721	2.36615	22	71	1350	849	40	216	35	101	95	108	118	78	88
sw15581	BGIBMGA004598	0.13535	0.6073	1	1	674	15451	14	5	-3	4	-2	38	15	44	26		9
sw09418	BGIBMGA002695	1.0928	1.06965	1.0503	0.34765	152	247	340	482	137	104	191	119	142	43	62	51	39
sw22672	BGIBMGA003402	0.61755	0.73525	0.7417	2.05635	2080	2766	3438	3140	1572	4293	1969	799	374	3133	2453	1347	2023
sw01229	BGIBMGA005862	2.71685	2.74305	1.7736	1.6006	2032	2722	1737	1801	1058	4379	1841	3244	3508	2977	2482	959	1075
sw10040	BGIBMGA010807	0.0757	0.6579	0.6793	0.88965	60	14619	15	28	40	-20	-27	25	11	-13	-22		-13
sw11741	BGIBMGA005330	0.3064	1	0.74855	0.70165	199	4826	76	77	27	47	43	138	86	76	48	85	56
sw11657	BGIBMGA003536	0.80345	1.01455	0.83785	2.85745	632	2289	453	451	269	253	337	440	761	491	578	1034	1223
sw20167	BGIBMGA013819	1.24005	1.02785	1.06105	0.30995	2709	1949	5375	5276	5105	33149	8736	4708	10491	85	93		20
sw09446	BGIBMGA002417	0.8522	1.17685	0.85535	3.27375	812	2699	1156	858	705	1195	367	549	612	1029	949	602	672
sw13530	BGIBMGA006177	0.718	0.81695	0.66475	4.4744	379	382	857	400	215	499	326	1390	1067	594	485	411	651
sw06122	BGIBMGA013717	0.73255	0.86025	1.0664	0.36135	1225	2048	932	770	760	918	731	836	797	1801	1320	1609	1964
sw13441	BGIBMGA000013	0.05605	0.8469	0.3692	0.9658	-22	-40	0	10	1	-29	-44	16	2	14266	36146	412	266
sw05146	BGIBMGA002439	1.2721	1.37505	1.5179	5.04485	6119	2836	2165	1430	1755	2280	1942	1535	1548	4900	3388	3808	5416
sw18920	BGIBMGA013723	1	1	1	0.41455	264	345	410	353	149	331	75	773	1258	176	240	177	194
sw10418	BGIBMGA010137	0.7156	1.08425	0.89055	2.93975	910	2436	610	491	289	197	448	444	433	732	721	571	699
sw15413	BGIBMGA005336	1.4101	1.2596	1.1751	2.13555	7	829	32	33	14	1302	9	1778	1911	30	19	11	27
sw15831	BGIBMGA006266	1	1	1	2.0635	110	360	503	897	167	140	83	110	118	808	663	89	168
sw14816	BGIBMGA009392	1.40235	1.09305	0.8807	0.48505	62	80	21	32	160	8222	23	416	553	22	9	32	37
sw01123	BGIBMGA011459	1.05665	1.13105	0.9106	3.1648	9	18	1433	363	17	63	45	87	32	34	25	18	33

sw15691	BGIBMGA011457	1.93685	1.22695	0.85835	2.7992	245	111	4799	14551	135	61	52	79	51	103	56		-1
sw14879	BGIBMGA011458	2.3441	1.43735	1.63085	2.5535	105	0	29738	23645	187	72	31	730	903	119	115	91	112
sw13451	BGIBMGA003404	0.484	1.24305	1.0731	2.48725	56	415	4960	369	21	10	9	13	45	3	3	9	-5
sw06424	BGIBMGA011079	0.374	1.59225	0.24925	2.40665	7	109	1393	1498	23	23	23	8	5	24	24	33	19
sw20455	BGIBMGA003345	2.6338	1.11045	1.03945	1.33455	1530	4042	5810	156	34	25	0	94	88	155	181		22
sw02018	BGIBMGA001308	0.3424	1.5086	0.3915	1.11665	551	453	1061	830	810	471	351	275	456	633	706	575	803
sw20990	BGIBMGA011460	0.0266	0.09555	0.0586	0.59525	53	-16	143	63	46	43	13	111	78	273	424	2334	2109
sw08480	BGIBMGA000681	4.27915	1.57315	2.479	3.6104	62	3066	6563	27961	264	2	2	15	2	3	-1	2	1
sw08246	BGIBMGA000520	1	1	1	2.18275	361	893	410	302	139	106	90	149	140	187	158	164	209
sw19527	BGIBMGA007030	1	1	1	0.4438	317	495	196	154	127	157	130	59	102	187	195	141	148
sw05218	BGIBMGA007027	0.94815	0.84935	0.97245	0.2502	487	4542	1432	428	200	166	225	258	604	223	259	319	247
sw18237	BGIBMGA005557	0.72085	1.2755	1.35295	3.64915	310	748	359	338	224	384	95	114	145	210	221	214	271
sw15429	BGIBMGA001230	2.81275	1.3617	1	1.3186	28	35	348	263	91	905	-15	1737	2840	86	206	0	24
sw14075	BGIBMGA001231	2.7854	1.7448	1.32485	1	-2	-10	190	12	2	815	-8	689	539	256	277		14
sw06692	BGIBMGA007632	0.97965	1.07365	1	2.113	957	2062	877	401	414	204	380	293	399	401	543	459	418
sw16997	BGIBMGA007299	0.85585	1.21805	0.9166	7.5102	1810	5945	1798	1642	1096	1341	684	1311	1695	2198	2137	1886	2255
sw07768	BGIBMGA007559	0.7547	0.83265	0.75115	4.396	932	2019	859	617	525	2068	501	1598	1570	775	699	290	405
sw15708	BGIBMGA000864	1	1	1	0.41935	937	1876	357	301	125	207	278	89	159	112	152	170	204
sw13022	BGIBMGA002880	0.814	1.10085	0.70235	4.87635	6804	9399	24419	15830	5911	20201	8574	16276	15084	8285	9590	7947	8809
sw07058	BGIBMGA011328	0.97915	1.0938	0.8651	4.74945	1788	2517	823	417	344	396	494	364	480	766	923	992	884
sw10856	BGIBMGA007876	1.5477	1.15995	1.1587	0.37865	419	131	285	506	489	5758	205	513	755	61	58	74	43
sw12193	BGIBMGA013506	1.25545	1.28805	1.1581	2.1506	1877	2088	1517	1462	849	1055	1309	1009	1347	1506	1472	1778	1936
sw07775	BGIBMGA010385	0.83045	1.0916	0.93815	2.0764	866	735	1061	504	257	814	630	427	450	475	504	393	528
sw09785	BGIBMGA010318	1.312	1.33425	1.29985	4.77525	1182	2172	497	487	792	428	416	320	190	1569	1142	1270	1965
sw11815	BGIBMGA000745	0.7771	1.1142	1.3569	0.49295	598	700	286	267	227	213	133	103	152	199	272	260	225
sw03956	BGIBMGA004026	0.1861	0.593	0.66175	1.7579	159	4890	41	43	23	49	20	44	32	47	41	52	51
sw03884	BGIBMGA013543	0.20895	0.50965	0.3297	0.6013	2504	17505	1771	768	847	748	1206	1117	810	1859	2358	1759	1816
sw00581	BGIBMGA011651	1.05865	0.99945	0.96025	2.3198	470	1087	381	332	206	563	422	476	431	524	452	458	479
sw03217	BGIBMGA005787	3.3007	3.0253	1.67145	0.8298	165	167	490	345	4247	244	817	167	220	139	101	58	96

sw13862	BGIBMGA009465	0.92615	1.16985	1.06955	2.01035	835	993	537	400	301	321	356	408	387	1044	810	794	1272
sw13521	BGIBMGA011070	0.8927	1.1285	0.9746	3.8009	2010	2242	1397	1252	678	1308	1117	846	799	1530	1294	1639	1925
sw00547	BGIBMGA002335	0.66465	0.99205	0.78555	5.72835	954	1329	1536	1056	611	1906	820	1455	1263	1088	898	715	1145
sw02239	BGIBMGA000664	0.7748	1.6878	1.283	2.94785	208	409	413	299	265	656	207	355	457	347	328	513	750
sw11663	BGIBMGA001222	0.9246	1.0078	0.81735	3.98315	642	2067	536	207	203	193	260	182	245	302	356	333	292
sw05343	BGIBMGA001221	1.11345	1.02125	0.976	2.6966	310	721	320	107	119	114	113	115	153	137	172	251	231
sw15779	BGIBMGA005949	0.7595	1.07345	1.02775	2.37555	5029	10468	1883	5519	4432	5527	3681	2402	2104	7984	6084	5213	7043
sw10315	BGIBMGA006200	0.90015	1.2047	0.80575	6.2053	1531	1449	2187	1611	1543	2139	1503	3363	3538	2459	2375	3158	3896
sw08850	BGIBMGA009355	0.9743	1.13865	0.84715	2.40515	4086	4415	4267	3540	1876	3266	2784	2449	1974	3842	3411	2678	3247
sw09459	BGIBMGA011041	1.68865	2.0717	1	1	227	319	79	727	1097	-4	9	92	48	-5	8		-12
sw17048	BGIBMGA003698	0.6904	1.1089	0.8297	3.62485	1142	1370	934	573	499	371	440	316	328	908	999	1154	1341
sw17045	BGIBMGA000375	1.0199	1.2083	1.0551	3.9354	1187	1006	539	449	419	584	376	401	558	771	1199	1610	1500
sw05506	BGIBMGA001335	1.555	1.35185	1.31225	2.27985	773	281	352	209	341	221	265	182	243	468	446	384	585
sw09626	BGIBMGA002379	0.4207	0.4669	0.58915	0.7168	4338	6445	8069	5337	4309	8751	5483	3213	5026	24437	20179	3244	6769
sw14517	BGIBMGA009875	0.96455	1.08945	1.06355	0.4623	597	1464	590	486	336	653	276	362	635	437	480	441	528
sw22963	BGIBMGA001859	0.2281	0.7038	0.49695	1.07775	272	19202	35	68	-2	33	9	78	64	43	34	30	33
sw19411	BGIBMGA009554	0.82175	0.73335	0.98795	2.0039	700	727	399	431	420	308	372	329	396	585	483	501	529
sw03494	BGIBMGA012388	1.1328	1.12135	1.1253	0.24515	2413	2369	2019	1100	891	1374	1492	940	1341	1358	1518	1244	1827
sw21623	BGIBMGA006899	0.9358	1.0279	0.9017	4.8919	1198	2295	628	358	267	449	287	183	210	495	548	542	631
sw14565	BGIBMGA008983	0.85765	0.8998	1.0321	2.5808	1676	4963	11402	5025	1419	4517	997	15445	28985	1258	1374	1377	1543
sw04136	BGIBMGA001746	1.091	1.10715	1.19115	0.47365	170	234	426	316	135	36014	368	626	921	212	266	91	118
sw21707	BGIBMGA004038	0.8866	1.09775	1.1704	2.73205	571	1384	464	274	213	365	610	195	251	573	431	242	340
sw03580	BGIBMGA010930	1.31075	1.2657	1.7451	6.4873	2361	1194	1588	1092	1713	1138	1102	533	362	3849	2851	3764	6070
sw01324	BGIBMGA004489	1.1834	1.02695	0.92925	2.38455	688	2212	1073	868	512	623	372	1040	1099	703	648	610	808
sw11688	BGIBMGA008813	1.23705	1.08965	1.33775	4.0178	-15	-15	166	27	104	36529	27	1009	651	6	-3		9
sw04517	BGIBMGA008815	1.0526	1.17035	0.9006	3.1204	121	140	46	79	1360	592	43	12344	13740	104	136	53	96
sw01225	BGIBMGA002001	1.05705	1.09535	0.9823	2.0794	631	1382	825	391	315	919	533	521	524	590	426	389	759
sw20321	BGIBMGA000232	0.7756	0.90565	0.8371	2.0538	1326	22985	4991	3167	1117	909	1199	872	1011	780	932	854	815
sw20478	BGIBMGA001956	1.0064	1.03045	1.08095	2.01005	44	22	72	28	35	1595	359	18	17	512	489	571	543

sw06179	BGIBMGA000997	0.0611	0.55095	0.589	1.40755	388	23075	26	14	78	26	0	105	23	11	-9	16	7
sw08892	BGIBMGA013446	0.0227	0.563	0.61075	0.5174	1391	58688	16	43	25	-18	-24	75		-23	-7		-28
sw17551	BGIBMGA001752	1.05965	1.06145	1.03515	0.46545	2048	2501	1821	1821	1135	2821	920	1280	1972	1308	1361	1101	1201
sw12574	BGIBMGA009994	1.0413	1.07585	1.2251	0.4381	2740	5615	1416	1041	1135	4884	1770	4812	6146	2256	1888	2086	2347
sw14253	BGIBMGA005736	1.45445	1.05765	1.0525	0.3354	7177	3045	776	5985	7565	-67	228	369	327	8664	11489	10092	9615
sw13009	BGIBMGA011468	1	1.7228	3.34165	1.47585	738	32	116	402	111	818	20	150	314	7	25	20	43
sw03532	BGIBMGA009360	0.9198	1.1711	0.90865	3.1235	1166	1386	973	757	648	1228	928	527	513	1563	1339	1344	2158
sw10552	BGIBMGA006840	0.6821	0.9187	1.1223	2.5722	8270	11298	7490	9938	6439	11280	7759	5346	4316	10219	7007	6410	10827
sw14041	BGIBMGA001437	0.87095	0.9736	1.0348	2.10255	12417	12635	8070	10517	5313	12828	14895	5200	6064	13186	10354	11121	15760
sw10747	BGIBMGA009830	1.1275	1.45765	1.40725	3.5298	8877	8162	4060	4712	4352	4036	4172	4770	4386	8089	7218	8452	9129
sw10030	BGIBMGA014491	1	1	1	0.4897	108	10876	81	43	28	-16	52	69	84	21	14	31	31
sw21012	BGIBMGA006507	0.6543	0.93395	0.78225	3.5569	2353	4127	4183	4528	4297	12335	2962	6565	6102	4243	3269	2700	4295
sw14293	BGIBMGA010919	1.42745	1.7126	1.1242	2.97145	5	632	3897	1055	40	168	49	148	72	38	45	38	45
sw10845	BGIBMGA000424	1.24705	1.2404	1.10325	0.24095	301	581	3746	1341	181	386	167	310	572	204	197	236	373
sw15361	BGIBMGA004910	0.48405	0.96	0.72475	1.62115	3	51	2926	1529	1277	342	-5	1530	107	28906	30035	27679	32331
sw11167	BGIBMGA008523	0.9054	0.82185	0.9167	0.49275	713	7053	711	389	313	495	242	495	787	881	1152	442	427
sw19561	BGIBMGA001173	1	0.86105	1	0.3812	445	321	409	399	248	166	131	273	216	505	480	1111	944
sw08167	BGIBMGA002337	0.23175	0.67295	0.65755	1.3936	531	13752	33	57	50	52	44	106	77	71	63	47	68
sw12651	BGIBMGA006160	0.5586	1.03655	1.44745	2.96225	1023	413	2642	1037	526	-30	5	188	69	14751	15631	8939	8485
sw10878	BGIBMGA010285	1.51855	1.08915	1.03225	2.41055	1701	6244	1363	12106	15696	22	53	894	490	45	54	17	54
sw16286	BGIBMGA006754	0.84045	1.08315	0.87625	3.3052	1629	4325	1943	1697	1066	1129	811	1240	1102	1029	1064	925	1141
sw08598	BGIBMGA007010	0.80945	1.2399	0.71395	3.9806	2738	3538	3875	2028	1780	5680	2530	2796	2947	1531	1721	1418	1525
sw15011	BGIBMGA007723	0.7364	0.72785	0.8108	2.5673	10008	7938	12977	23162	13683	22874	8601	12726	11618	12145	11553	8910	11056
sw03796	BGIBMGA004009	0.7396	0.8941	0.8191	3.59665	3122	10353	15040	6354	4001	7590	2114	10408	7905	3546	3731	3676	3945
sw05929	BGIBMGA003562	0.94725	0.93135	0.8557	2.08405	1077	1946	3650	1462	757	2361	672	3383	2564	1095	915	936	1190
sw07693	BGIBMGA003939	1	1	1	0.4057	151	75	449	450	36	-4	1060	34	44	6	4	3	30
sw02182	BGIBMGA014475	0.9184	1.04185	0.9647	2.82585	132	632	166	128	111	248	130	124	95	125	148	160	213
sw14729	BGIBMGA005936	1.1297	1.1862	1.0978	2.62255	1783	1626	1062	1225	606	837	822	1648	1648	1660	1389	1475	2078
sw00440	BGIBMGA005786	2.688	1.80735	1.4433	0.79945	38	88	50	49	65	2315	43	1310	3691	135	136	165	210

sw13880	BGIBMGA014287	0.12105	0.56645	0.4446	0.61675	65	83	234	223	1011	63	13	1188	1285	7619	13561	9121	6304
sw02398	BGIBMGA013707	1.11	0.9797	1.2912	0.3907	842	1085	961	785	203	386	318	386	331	356	380	352	393
sw17911	BGIBMGA011592	1.0353	1.0663	1.18465	4.5845	444	346	577	311	298	406	205	263	250	355	328	308	376
sw14639	BGIBMGA011782	1.0933	1.07795	1.0757	3.13045	2537	3848	1029	875	977	841	1148	795	856	1647	1900	1973	1775
sw18291	BGIBMGA013676	1.0292	1.2041	0.9982	2.85545	1562	1921	708	1253	1870	7526	925	1976	2181	1757	2000	862	1021
sw17801	BGIBMGA001829	1.0979	1.24205	1.1656	2.1714	2643	2477	1266	1221	947	1524	1130	1221	1167	3288	2118	2592	4104
sw18181	BGIBMGA012486	2.5286	2.0179	0.88725	0.64055	798	36	570	2090	12714	-53	-18	356	756	21	17		-9
sw11583	BGIBMGA001068	1.0169	1.02585	1.18075	3.00565	-19	20	4	-28	5	8845	-11	3363	4237	-29	-12		-23
sw20415	BGIBMGA012624	1.52565	1.11905	1.34265	4.272	741	193	166	268	253	6710	196	2696	3439	55	33	16	23
sw18815	BGIBMGA001348	1.38275	0.473	0.57655	0.8861	14685	839	5733	7923	9440	586	19191	375	276	36	20		39
sw11338	BGIBMGA009280	1.0438	1.009	1.1422	0.4966	361	661	639	328	265	2106	348	1822	1713	1913	1625	603	731
sw13340	BGIBMGA009206	0.0898	0.68505	0.73985	0.71465	543	45510	150	176	76	220	165	383	414	80	125	57	117
sw08784	BGIBMGA010377	1.17655	1.02225	0.9609	0.35835	3683	3449	1720	5560	7182	3713	60	4235	3842	1029	1250	1154	1044
sw14927	BGIBMGA008268	0.55355	0.57775	0.68505	0.3246	13	45	6128	262	15	6	11	189	8	62547	57892	5174	13636
sw00924	BGIBMGA003924	0.83575	0.823	0.8703	2.2332	448	917	3452	1494	707	2068	394	1932	2057	689	674	898	1103
sw05530	BGIBMGA008541	0.79265	0.8124	0.719	0.23525	768	127	622	633	873	139	316	155	100	197	204	139	194
sw12797	BGIBMGA006727	1.0466	1.4222	1.19375	2.2653	755	1758	303	615	567	540	86	2141	3371	206	205	329	318
sw07852	BGIBMGA005306	0.39475	1.0466	0.65895	3.11385	820	755	334	289	260	274	277	179	155	849	611	343	598
sw20748	BGIBMGA011671	1	1	1	0.3168	635	342	309	123	137	117	171	65	105	155	163	468	508
sw15595	BGIBMGA001490	0.8492	1.18575	1.81515	3.14135	11402	5395	5487	5964	7588	7730	7838	3083	4767	11372	9547	9990	15203
sw18585	BGIBMGA010745	1.37205	1.0557	1.40475	3.52605	2439	10849	915	819	1214	1356	1034	921	737	4185	3040	3718	4662
sw09451	BGIBMGA007508	2.2004	1.42785	1.2052	2.0384	341	177	250	2363	3509	6	153	99	100	-5	-5		11
sw21135	BGIBMGA005745	1.0065	1.07295	0.99225	0.41515	7206	7103	4068	7697	3884	6831	4060	4835	6716	6495	6402	4597	5311
sw07891	BGIBMGA011611	1	0.7623	1.2143	0.40695	3864	3300	1248	575	432	388	980	157	247	629	685	873	806
sw15513	BGIBMGA005105	1	1	1	0.3539	565	325	595	814	283	-12	-4	39	82	365	415	47	86
sw16198	BGIBMGA005101	1	1	1	0.25625	56	74	183	62	70	28	34	507	1253	1275	1446	1036	884
sw08641	BGIBMGA008494	0.94315	1.1391	0.9906	3.0993	925	1451	1007	919	420	585	604	504	586	513	582	761	752
sw17819	BGIBMGA010975	0.8697	1.19725	1.1201	3.566	1889	1771	1516	1547	1170	1788	1086	973	1248	1793	1367	1321	2079
sw22978	BGIBMGA005460	0.9712	1.10585	1.1601	3.5258	2406	3461	1722	1862	1413	1729	981	1776	1861	3721	3540	3471	4345

sw15847	BGIBMGA003466	0.7652	0.92435	0.7104	2.67485	10981	14775	19085	16708	3983	9129	6624	10778	9172	8001	6753	5440	6957
sw06913	BGIBMGA012977	1	1	1	0.4515	541	1061	261	126	153	261	299	242	257	148	173	178	212
sw15406	BGIBMGA004639	0.96035	0.9161	1.0308	0.46595	1895	2072	919	1470	908	1486	471	604	978	299	342	249	254
sw07506	BGIBMGA005300	2.24045	0.82805	0.65335	0.38955	870	511	2073	1823	205	377	135	488	542	116	142	100	133
sw16983	BGIBMGA006984	0.78	0.9604	0.8421	3.8827	2721	5914	2171	1885	1029	2222	1884	2008	1571	3368	2500	2223	3073
sw15076	BGIBMGA004021	1.11555	1.20605	1.3461	2.09185	1240	740	677	439	410	779	788	505	567	276	273	218	298
sw17562	BGIBMGA004344	0.9686	1.0089	1.13115	0.4916	1630	2406	1833	1280	1054	2499	2869	2846	3690	1124	1210	1130	1137
sw20497	BGIBMGA013985	0.91345	0.91905	0.9171	2.8431	2793	3357	1248	1244	720	1107	504	1134	1002	1660	1479	1227	1422
sw05224	BGIBMGA004064	1.10595	1.0799	0.65365	2.0362	191	417	681	252	127	147	40	108	111	201	273	216	201
sw19986	BGIBMGA003574	0.7738	0.7956	0.58085	0.30245	1348	703	4473	72	15	-4	23733	108	142	-9	-16		-1
sw09815	BGIBMGA007711	0.87205	1.1595	1.0248	2.9055	550	714	486	229	244	292	213	154	182	295	301	335	505
sw12216	BGIBMGA012075	0.78255	1.05675	1.0026	3.70265	1446	1889	1472	1408	1245	1372	818	1113	968	1704	1243	1243	2046
sw03241	BGIBMGA003865	5.5432	2.92495	1.80485	0.71	654	1877	263	449	769	365	191	190	321	49	50	71	89
sw09637	BGIBMGA005145	1.102	1.04335	0.92655	2.1949	153	999	162	88	50	134	94	197	246	98	101	85	72
sw12244	BGIBMGA007848	1.13745	1.03395	1.0081	0.3353	3971	3072	1704	9787	14245	247	119	725	703	195	231	230	224
sw14309	BGIBMGA000391	0.68305	0.78005	0.77265	0.26665	685	1875	639	1302	1671	472	442	262	420	177	202	133	174
sw20255	BGIBMGA001126	0.48865	0.96765	1.0703	0.33975	1817	2163	1371	586	644	566	400	347	542	1569	1715	2265	2729
sw16854	BGIBMGA007767	1.41845	0.73505	0.87545	3.0115	1330	1143	1963	5359	6929	8441	8629	938	1235	585	644	27	69
sw04248	BGIBMGA007766	3.6876	2.844	2.26695	2.901	12	37	55	34	30	912	34	527	1089	54	75	57	67
sw18052	BGIBMGA001754	0.7906	0.9647	0.9049	0.4876	93	5	1256	1791	103	186	146	41	115	15	23	23	18
sw21098	BGIBMGA003658	0.90435	1.05615	1.1157	2.64815	1411	5776	511	2636	5493	6859	185	4419	4979	332	321	899	991
sw18194	BGIBMGA009614	1.3675	1.2788	0.9787	3.79655	6351	801	4183	5699	6368	114	3141	529	430	77	85	51	72
sw13853	BGIBMGA011074	1.66445	1.1724	1.5427	2.525	8	34	74	22	19	27866	105	254	410	117	90	108	146
sw17949	BGIBMGA003741	0.7984	0.73215	0.7132	2.10615	3770	866	859	5146	15610	44	75	480	274	33	9	13	63
sw16663	BGIBMGA004848	1.28045	1.2879	1.224	2.14725	2682	1015	1024	841	664	904	588	531	662	634	678	646	840
sw07091	BGIBMGA004280	1.2673	0.96145	1.0756	3.11805	1007	195	2979	1934	2918	168	22	149	219	27	30	18	30
sw20815	BGIBMGA000386	0.1438	0.5122	0.6586	1.3799	75	18764	16	132	46	49	9	58	19	14	14		1
sw00678	BGIBMGA006703	0.2436	0.6517	0.76625	0.85335	220	12430	64	53	31	410	41	115	73	89	73	96	113
sw05455	BGIBMGA009467	1.9794	2.1042	1.91805	0.94125	2164	969	5011	426	228	134	8687	142	134	57	57	112	140

sw03106	BGIBMGA008525	0.79295	0.9173	0.8743	2.8264	960	4215	4627	2046	1617	1861	1085	3355	2749	2291	2138	1522	2109
sw08557	BGIBMGA014189	0.99785	1.17085	1.32215	2.80645	7	10	83	770	34	3146	4	37	61	35	46	53	29
sw01530	BGIBMGA014149	1	0.76955	1.0619	2.7225	19	40	69	94	35	812	52	696	1226	134	138	53	78
sw21386	BGIBMGA010537	1.46275	2.0431	1.902	1.6639	-19	-42	-18	-5	-12	6649	-15	44	69	-10	-1		1
sw18703	BGIBMGA010812	2.6714	2.10235	2.6688	1.09535	-3	-28	206	20	14	102057	160	162	964	42	54	18	20
sw19095	BGIBMGA014144	2.3389	1	1	1	308	450	210	56	215	11	-10	52	13	2744	4296	2604	2239
sw03036	BGIBMGA014187	1.1031	1.0777	1.1397	0.4484	3866	7539	2336	1368	1583	1379	1490	1369	2305	1634	2146	1850	1702
sw17712	BGIBMGA005539	1	1	1	0.14205	655	765	47	332	877	44	18	49	45	45	22		37
sw19182	BGIBMGA005899	1.15945	0.9304	1.1003	0.4863	316	363	711	718	153	149	517	96	152	19	41	39	40
sw20215	BGIBMGA014116	0.44205	0.26285	0.44215	0.16365	685	3667	448	4759	9124	1702	2	218	277	65	53	48	71
sw10670	BGIBMGA013129	0.3553	1.1206	0.7204	2.0257	-14	-20	13	0	10	14	-11	11	0	2756	3542	1082	893
sw11301	BGIBMGA007153	2.1025	1.39835	1.7561	1.27425	234	636	796	778	193	16142	197	351	611	138	158	105	161
sw05614	BGIBMGA013995	1	1	1	0.42695	200	561	245	115	76	68	95	132	190	75	61	23	16
sw09024	BGIBMGA005181	1.3062	1.11325	1.0238	0.37195	218	180	155	457	723	42	16	51	57	68	79	62	60
sw09062	BGIBMGA000621	1	1	1	0.38545	554	407	421	310	391	74	587	107	142	67	77	129	97
sw18769	BGIBMGA012827	0.8926	1.0213	0.9166	2.3879	609	700	235	379	601	3925	303	7005	6155	184	218	146	130
sw15711	BGIBMGA004218	0.7422	0.99545	0.796	3.5789	1782	1019	1143	981	1387	1420	1259	1243	1362	1094	1037	559	757
sw13402	BGIBMGA001115	0.904	1.2773	1.06385	2.40795	2577	3892	2466	2655	2027	5161	2175	3893	5818	3766	2993	3039	3606
sw05319	BGIBMGA005692	1.0139	1.1851	1.53875	2.2423	408	146	50	38	14	756	29	71	127	49	53	35	35
sw22347	BGIBMGA012999	1.8581	2.6587	4.22965	4.9108	18	13	25	405	233	27	16	110	107	772	867	62	140
sw20836	BGIBMGA005710	0.4171	0.77975	1.0747	2.94	53	173	106	132	20	9	-1	81	52	24248	24329	3714	3583
sw21310	BGIBMGA009925	1.16125	0.89465	1	2.09175	16	40	22	24	214	-12	-3	2321	3113	104	100	49	81
sw16532	BGIBMGA000158	0.13745	0.15465	0.2374	1.5367	8	21	7127	257	10	31	21	49	57	52250	37283	2502	14016
sw14437	BGIBMGA012586	0.3161	0.6954	0.5105	0.62755	37	1160	285	514	196	81	16	203	46	94	48	24	24
sw19386	BGIBMGA012863	0.25035	0.71345	0.3407	0.57675	452	14168	780	148	28	-13	107		2	39	44	25	30
sw02084	BGIBMGA012997	1.09155	0.99	1.034	0.44125	792	293	4354	42405	25552	377	221	303	486	199	240	180	220
sw10247	BGIBMGA013007	1	1.0274	1.3095	0.39305	396	717	690	417	356	433	291	202	306	364	289	359	439
sw05478	BGIBMGA009559	0.65585	0.895	0.77325	3.4921	1799	1928	1624	1719	1671	6235	2128	3773	3989	2651	2136	2277	3755
sw03207	BGIBMGA007490	1.02715	1.0692	1.072	3.425	2126	1217	6535	4972	2675	3787	1627	2245	2275	1359	1696	1643	1497

sw00343	BGIBMGA007515	1.18505	0.9829	1.01485	0.488	487	733	403	305	179	466	194	219	364	297	353	431	499
sw17956	BGIBMGA005889	1.03045	1.3253	1.3701	0.37345	781	1217	784	510	230	414	245	264	434	358	394	520	638
sw05257	BGIBMGA009618	0.8471	0.78115	0.9092	3.26655	574	1554	524	492	493	1016	558	1962	2331	883	754	787	1043
sw13043	BGIBMGA011820	1.41415	1.07185	1.06015	2.6623	402	238	126	1579	2462	10474	8	6967	7037	-12	9		-6
sw18220	BGIBMGA009106	1.4047	1.16185	0.98535	2.1309	5041	6979	5203	69299	35429	1407	76	206	313	36	48	17	31
sw14925	BGIBMGA011658	2.0413	1.85725	1.2505	1.53155	5866	2356	7209	7356	5766	6673	8407	16349	19176	6180	9127	11737	7781
sw03335	BGIBMGA009111	1.0233	1.05355	0.97665	2.17545	291	558	293	153	137	209	127	230	240	232	219	213	252
sw18489	BGIBMGA010278	1.46225	1.54515	1.5254	2.16855	1568	847	876	919	1073	1629	709	430	612	2278	2412	2642	3122
sw10128	BGIBMGA001936	1	1	1	0.4956	137	675	145	88	94	112	43	93	110	87	90	65	72
sw09947	BGIBMGA001265	0.79585	0.9675	1.21435	2.6198	1555	2056	1583	1188	802	1207	1399	582	690	1592	1105	1049	1939
sw22695	BGIBMGA003395	0.2266	0.65665	0.4827	1	7887	1575	1291	48228	36138	1326	33	3153	650	43	61	40	164
sw11286	BGIBMGA007516	0.25745	0.72145	0.7481	0.4466	1380	23791	670	58	17	1	-16	22	14	-5	-10	4	6
sw11643	BGIBMGA002894	1.14955	1.0263	1.09235	0.33455	394	651	1592	687	264	335	140	151	167	221	270	328	422
sw05778	BGIBMGA002642	0.87625	0.94205	0.849	0.44865	1206	1067	2452	1770	1064	2201	1013	1171	1720	914	957	701	724
sw15490	BGIBMGA002064	0.99615	1.20675	1.0183	2.565	597	707	413	381	372	437	311	613	514	678	582	527	738
sw19539	BGIBMGA006159	0.758	0.96695	1.0401	2.15725	3557	4528	2423	2515	2252	2502	1873	1881	1706	3182	2926	2631	3202
sw04421	BGIBMGA008701	1	1	1	0.4151	402	180	425	365	261	200	178	212	218	646	614	613	683
sw01718	BGIBMGA003361	2.0572	1.40725	1.6631	0.48515	11320	20529	8928	6122	6051	5234	7631	5674	6133	10564	13849	13349	12803
sw09439	BGIBMGA000189	1	1	1	0.4744	208	784	209	116	77	68	93	122	238	106	161	132	124
sw15251	BGIBMGA012514	0.5398	0.58165	0.8912	0.4559	908	12480	539	350	425	399	530	247	467	295	353	314	360
sw01819	BGIBMGA012772	0.80015	0.8565	1.19915	3.69495	3662	3732	1840	1475	1144	1435	2304	1012	850	3613	2756	3154	4641
sw22014	BGIBMGA009885	1.1845	1.1234	1.54995	2.8019	1758	6354	731	758	686	959	855	417	553	2129	1812	1673	2121
sw21778	BGIBMGA011804	1.1491	1.39195	1.42375	2.31255	3489	4011	1219	1051	502	813	742	667	821	1509	1197	1360	1834
sw11664	BGIBMGA001223	0.94985	1.1198	1.06915	2.25735	5398	5128	5399	3861	3590	3082	3416	3531	3072	3858	4076	3865	4669
sw14091	BGIBMGA002654	1.44375	1.17685	1.50235	2.24375	1913	3417	978	744	973	692	868	564	643	2387	1904	2285	3213
sw13724	BGIBMGA006525	1.01035	1	0.88775	2.07735	877	1319	477	549	461	533	387	582	680	606	527	514	665
sw17607	BGIBMGA005168	0.3294	0.5308	0.43475	1.03555	341	1545	259	260	231	392	156	215	276	197	209	420	405
sw13920	BGIBMGA011965	0.494	0.83565	0.69615	0.9527	642	1501	792	546	588	592	387	594	539	1257	1593	1554	1529
sw03369	BGIBMGA008914	1.0034	0.9653	1.0706	0.46085	1002	1048	331	268	196	386	403	308	334	718	502	443	740

sw11487	BGIBMGA010637	0.8984	0.9238	0.86535	0.42945	3641	13109	3552	2500	1390	2226	2306	1282	2236	2682	3034	2385	2306
sw18960	BGIBMGA011754	1.1258	1.06705	1.1043	0.41885	5032	11997	6355	4494	2094	5750	2712	2405	3701	2023	1841	1972	2220
sw13199	BGIBMGA013998	0.7642	0.95805	0.7933	2.8666	1606	2097	1391	955	1113	1606	1078	1295	1770	1074	1044	1348	1540
sw07452	BGIBMGA012764	2.10635	1.62895	2.05445	0.40115	84	341	325	56	45	45	1263	73	84	57	42	47	50
sw05534	BGIBMGA013115	1.8705	2.32265	2.10675	0.74345	13241	5700	41333	2699	952	401	64826	231	272	132	135	120	168
sw19955	BGIBMGA012763	1.4839	1.91125	2.0136	0.2737	302	301	638	503	182	461	1942	315	242	208	184	188	194
sw15596	BGIBMGA005274	1.08425	1.16075	0.77275	2.0861	2128	2754	2110	1440	1184	1396	1569	1280	1247	1018	1304	1248	1001
sw08786	BGIBMGA011477	1.1328	0.99725	1.12485	0.25005	69	97	754	317	200	329	137	159	164	145	136	119	106
sw08118	BGIBMGA013449	0.77845	0.90915	1.1347	2.82895	15218	9410	10394	6790	5647	8017	11460	5035	4473	12326	9315	6212	11052
sw16101	BGIBMGA011899	0.78455	1.1833	0.88885	2.96975	2057	7264	2556	2166	1756	3921	1079	744	867	5502	6703	3325	3167
sw01142	BGIBMGA007303	0.2957	1.038	0.61325	0.28315	150	179	396	272	243	540	29	141	104	571	665	1889	1807
sw15877	BGIBMGA007493	1.2232	1.0576	0.98165	2.83415	1750	1914	703	556	586	578	445	845	1028	678	646	541	680
sw15943	BGIBMGA006848	1.09115	1.0954	1.1319	0.4786	1015	904	452	325	264	619	419	183	300	489	425	437	532
sw14827	BGIBMGA010711	1	1	1	0.42475	339	722	141	68	87	-9	86	39	97	73	87	49	118
sw11598	BGIBMGA001132	0.8814	0.827	0.89765	0.3237	2578	2591	2786	1008	593	1002	1397	514	1145	924	930	1028	1174
sw20582	BGIBMGA004001	0.97395	1.17135	1.1154	2.88085	702	736	262	252	184	359	96	157	136	382	278	153	343
sw13243	BGIBMGA006116	0.0718	0.63175	0.63205	0.88725	605	48099	67	42	13	31	7	87	25	22	2	13	36
sw08418	BGIBMGA006385	0.4462	0.6361	0.64385	0.82525	1668	2598	3492	2992	1430	2988	2494	1718	2145	1088	1145	1022	978
sw07781	BGIBMGA008409	0.27185	0.64455	0.5084	0.47675	464	342	3022	755	976	251	153	1740	185	684	445	121	128
sw08530	BGIBMGA000639	0.28835	0.64375	0.4299	0.29165	1090	2759	11812	2855	5253	2088	948	10053	1498	4170	2520	2653	2042
sw19580	BGIBMGA010370	1.6497	0.88645	0.71975	3.07385	889	207	273	1858	1461	2792	62	413	265	99	42	37	36
sw09048	BGIBMGA009927	1.44505	1.45305	2.09225	0.67945	399	359	250	522	601	380	393	270	270	215	224	204	247
sw13175	BGIBMGA004515	0.9027	1.67455	1.44745	2.3107	3580	16540	678	384	1076	13829	2610	2106	2099	739	399	163	311
sw05107	BGIBMGA004606	0.554	0.4493	0.64175	1.51345	334	295	3969	8413	501	1171	77	161	160	144	86	66	139
sw20205	BGIBMGA005784	0.19215	0.63865	0.7535	1.45735	765	47420	865	249	79	59	13	135	47	86	49	45	43
sw00199	BGIBMGA004540	0.313	0.70275	0.83285	1.1497	1969	15591	641	562	147	383	1565	712	550	4683	1677	536	1480
sw18898	BGIBMGA004541	0.4982	1.2154	0.3939	1.0237	3849	8802	1238	1821	343	604	621	421	372	3771	593	190	961
sw15945	BGIBMGA004630	1.0991	1.4282	0.2501	0.7535	1096	773	581	400	154	333	400	397	656	504	656	818	703
sw14679	BGIBMGA006574	1.0909	1.04885	1.35755	3.2331	1542	1554	749	676	871	584	605	514	435	1121	852	1013	1306

sw00221	BGIBMGA001218	1.10285	1.16575	1.2889	0.37695	782	1918	539	414	293	811	1030	875	905	536	434	472	632
sw01900	BGIBMGA004612	1.94495	1.5786	1.92665	2.21125	710	1588	750	785	462	982	1123	514	374	1307	488	488	1410
sw09820	BGIBMGA012753	1.0547	1.00155	1.46245	0.34335	3322	6120	2296	2682	3424	12418	3126	870	1718	2996	1324	1021	1819
sw18679	BGIBMGA005426	1.0438	1.128	1.17635	0.48975	325	379	501	426	344	422	248	340	388	214	205	174	231
sw00830	BGIBMGA002078	0.88505	1.1494	0.8921	4.12245	561	551	458	327	312	210	237	251	245	307	312	302	377
sw13964	BGIBMGA009814	0.5042	0.9989	0.84195	0.4597	712	4251	968	1195	3706	5313	585	770	1604	1704	2378	2115	2254
sw03540	BGIBMGA005036	1.1444	1.01915	1.0256	0.45285	1835	1166	8288	6018	3943	4940	1825	4995	5678	3470	5904	5947	4459
sw13878	BGIBMGA004453	0.9184	1.06425	1.06065	0.38025	724	901	1405	1221	705	1266	673	2304	3744	470	611	754	564
sw20245	BGIBMGA001372	1.4071	1.28445	1.52265	2.61495	-27	-70	12	34	16	20721	11	1033	1013	7	14	3	-7
sw21082	BGIBMGA005521	1.1005	1.1813	1.2816	2.07265	949	785	586	1298	1143	1468	1015	800	1319	634	727	560	594
sw04945	BGIBMGA009530	0.0442	0.50805	0.5175	0.74375	512	22793	113	85	38	47	23	154	55	57	72	105	109
sw02003	BGIBMGA003652	1.0545	0.90185	1.11195	0.2447	615	348	573	610	278	477	520	253	292	326	328	431	545
sw08325	BGIBMGA006462	0.8277	1.12195	1	7.92665	1722	2086	652	779	784	601	568	750	553	1696	1439	1628	2109
sw20339	BGIBMGA002719	0.7915	0.8951	0.7901	0.30245	1013	1196	1231	1245	506	672	285	495	1482	766	976	673	772
sw13299	BGIBMGA013605	1.01995	1.16855	1.66615	2.92025	3546	2481	1126	1075	1234	1917	2194	731	1084	3496	2000	2114	4229
sw01501	BGIBMGA008552	0.53265	1	1	2.02405	2180	1142	2373	1282	971	1489	807	1650	2113	1030	1035	1243	1433
sw03590	BGIBMGA013868	0.49725	0.7061	0.53775	0.81525	101	709	174	119	179	102	82	144	83	81	122	147	176
sw02606	BGIBMGA004767	1	1	1	0.50475	116	59	315	105	31	63	605	85	74	77	78	103	100
sw06810	BGIBMGA008146	0.9264	0.44585	0.4733	0.398	4892	295	1373	340	171	7075	57	206	190	107	77	88	107
sw11275	BGIBMGA014206	1.6095	1.36905	1.28195	0.3567	980	1525	592	1887	3048	132	45	300	258	251	203	143	194
sw15267	BGIBMGA007626	0.37545	0.6649	0.5048	0.4798	378	416	592	840	525	633	553	530	760	242	304	65	106
sw09660	BGIBMGA004919	1.03815	1.1799	0.9264	3.7032	386	310	240	295	201	363	175	295	323	282	266	291	305
sw02984	BGIBMGA005477	1.16195	0.90025	1.0779	0.3224	152	133	503	439	289	832	306	269	339	157	193	143	196
sw18890	BGIBMGA006878	0.9342	0.92215	0.9817	0.22635	1655	1636	2644	2074	1025	439	1724	957	1753	451	528	476	611
sw01251	BGIBMGA001988	1.67805	1.49135	0.837	0.3778	1145	1137	3181	1516	3844	166	555	171	181	124	126	104	115
sw03160	BGIBMGA013274	0.94245	0.88725	0.82915	2.67365	762	1079	760	727	381	382	425	340	360	429	407	375	597
sw20057	BGIBMGA002286	1	1	1	0.4951	29	68	2719	1076	61	86	36	72	82	66	28	12	20
sw14149	BGIBMGA005292	1.1142	0.95385	1.09055	0.35935	308	1823	331	135	75	576	153	114	324	63	100	107	118
sw03453	BGIBMGA005122	0.19445	0.5382	0.6389	0.74545	194	5013	47	50	43	62	44	61	55	130	128	181	215

sw11177	BGIBMGA009836	1	1	1	0.48265	275	2062	229	117	39	343	103	116	200	32	47	44	42
sw05299	BGIBMGA007034	1.30965	1.20925	1.16205	0.43315	584	118	1162	580	169	368	234	187	327	371	314	345	432
sw09671	BGIBMGA006920	0.74975	0.92775	0.69595	3.2443	703	1719	952	481	468	355	383	393	399	371	448	510	529
sw09094	BGIBMGA004546	2.01475	1.4162	1.34055	1.2466	460	533	5573	3494	258	1149	210	293	374	304	267	199	228
sw12091	BGIBMGA005747	1	1	1	0.4036	525	605	214	179	183	139	212	252	444	67	107	105	82
sw17684	BGIBMGA000623	1.73295	0.91525	0.9173	0.3795	234	28	13307	21041	431	2201	10	73	108	458	607	288	371
sw22430	BGIBMGA009441	1	1	1	0.35225	16	5	75	51	10	33	618	7	15	12	19	23	23
sw00653	BGIBMGA012048	0.70625	0.7912	0.70135	0.341	1503	1544	3791	3746	1860	4706	2453	2379	2780	2152	2130	1572	1793
sw02946	BGIBMGA000622	1.71835	0.92215	1.50265	0.2909	64	75	887	1138	53	214	61	118	119	101	130	139	184
sw05600	BGIBMGA011412	0.8806	0.96775	1.06805	0.46495	260	1339	2496	726	177	1188	197	578	1384	178	194	235	311
sw18742	BGIBMGA009671	0.8869	0.90415	1.00255	3.27705	636	1025	1209	966	871	1774	142	1476	1230	836	925	638	652
sw01022	BGIBMGA003330	1.19345	2.03655	1	2.8663	44	146	124	231	146	108	64	1335	1670	77	83	48	60
sw08986	BGIBMGA011572	1	1	1	0.49005	235	302	273	134	69	157	1342	184	251	69	73	84	107
sw18757	BGIBMGA003438	1	1.0499	1.422	2.35945	1387	1396	396	372	313	269	706	271	310	1861	1573	1519	2563
sw21881	BGIBMGA014064	1.16355	1.7853	1.11005	4.82495	8019	6998	6405	6796	7574	5666	4425	5118	5497	13420	15202	16263	15099
sw02310	BGIBMGA005381	0.74495	0.86105	1.00455	0.4764	1220	1357	694	327	165	468	1456	132	148	587	588	364	533
sw07468	BGIBMGA003020	1	1	1	0.4225	155	5681	178	98	88	55	46	223	367	89	111	134	123
sw09646	BGIBMGA005102	1	1	1	0.4113	341	4378	108	46	38	-36	71	37	46	10	18	19	5
sw02952	BGIBMGA006809	1.0099	0.94895	1.0096	0.3952	2071	3687	2332	1497	1318	1354	688	1081	1324	1417	1229	1435	1828
sw09115	BGIBMGA013923	0.7921	0.89075	0.8561	3.42315	3567	5316	6602	4072	2228	4748	3393	5534	4999	5976	4658	4269	5383
sw21243	BGIBMGA009696	0.9637	1.10795	0.92385	2.2131	1850	1590	1222	1075	367	1120	693	658	661	1665	1368	1327	1869
sw15512	BGIBMGA004019	0.68675	0.98245	0.81985	2.06715	2646	7970	1563	1208	1475	1430	1136	979	1522	1292	1285	1242	1472
sw13706	BGIBMGA010819	0.90285	1.0661	0.50775	4.15685	1213	2261	1371	1221	790	1694	793	2018	2116	1360	1108	1087	1499
sw15615	BGIBMGA012200	0.80445	1.01505	1.09215	2.1191	1952	2175	2655	2251	1620	1368	846	1656	1599	2175	1730	1711	2264
sw14533	BGIBMGA005418	1.12305	1.07145	1.09505	0.20945	779	527	1361	965	426	1017	839	2957	7411	233	231	493	543
sw11565	BGIBMGA000910	1.2666	0.9709	1.0301	0.2926	6546	2438	6392	2977	1974	280	14704	150	186	41	41	32	36
sw03233	BGIBMGA006063	0.7349	0.9089	0.9792	0.4474	8735	8965	6856	3417	2328	8115	11814	3272	9215	2718	2628	2490	3870
sw10478	BGIBMGA001554	1.50905	2.07925	1.9729	2.26425	1407	937	752	481	642	522	646	398	569	875	1099	1723	1603
sw06049	BGIBMGA005403	0.14715	0.57695	0.63155	1.11635	363	18401	28	40	8	41	26	155	180	89	62	54	72

sw14865	BGIBMGA005191	0.3363	0.6735	1	1	64	18815	78	187	26	64	30	108	76	20	42	7	16
sw15568	BGIBMGA001156	0.43165	0.79945	0.72515	1	290	15982	168	232	120	345	33	21	29	29	19	33	41
sw12103	BGIBMGA004130	2.59595	1.17535	1.7725	1.40755	22	311	43	113	98	8	28	2858	5880	12	13	19	21
sw15109	BGIBMGA011852	1.04385	0.8361	0.9057	0.25225	543	581	553	207	189	527	64	334	930	744	665	221	334
sw22325	BGIBMGA002288	1.0308	1.0051	0.4899	2.1828	2323	545	1253	9036	13609	257	135	275	351	97	79	82	94
sw19096	BGIBMGA001352	0.9658	1.09675	1.1177	0.38595	766	1049	897	1165	1721	3212	493	730	1659	585	639	868	1115
sw10846	BGIBMGA011426	0.9065	0.9104	1.0221	0.40295	940	1436	1289	680	600	1504	1043	1252	2069	649	605	636	801
sw17493	BGIBMGA009393	0.46435	0.91245	0.72025	0.6802	69	418	40345	1135	41236	189	45	31675	493	38600	41136	39442	45922
sw10237	BGIBMGA011530	1	1	1	0.51075	5519	3884	2221	2541	975	1007	3900	1477	1957	2022	2339	1551	1780
sw18085	BGIBMGA012780	0.9419	1.09045	1.29165	4.88875	1056	1902	3794	2027	1647	2959	616	1700	3008	1072	1074	1407	1684
sw11259	BGIBMGA007783	0.1047	0.62535	0.4015	0.24595	333	10566	14	37	38	19	-2	184	175	896	1899	373	271
sw21951	BGIBMGA001202	1.739	2.17935	2.6849	2.3214	357	340	42321	59232	2317	12053	14	477	275	207	210	27	53
sw04137	BGIBMGA012680	1.921	1.64625	2.82885	0.8215	48	46	2023	1980	73	290	60	102	89	88	78	61	106
sw19875	BGIBMGA004580	1.44565	1.34015	1.89905	0.46935	133	83	737	803	149	89	23	45	76	68	62	37	28
sw16262	BGIBMGA002789	1.0872	0.8952	1.398	0.4354	1567	1550	1944	1404	689	661	1118	295	381	432	405	359	395
sw06780	BGIBMGA001507	1.3057	2.0168	2.0887	4.89775	259	384	256	180	109	2105	94	102	144	162	158	152	149
sw12396	BGIBMGA003920	0.8528	0.84435	0.65565	2.89165	395	345	928	520	566	3702	196	9020	6181	186	210	60	108
sw13946	BGIBMGA001506	1.1311	2.08765	2.1563	2.63455	51	60	176	173	90	1858	41	80	107	46	67	23	23
sw13529	BGIBMGA002669	1.23735	1.68715	2.2502	1.1997	-1	38	90	72	32	91086	312	20	99	10	42		4
sw06172	BGIBMGA006559	1	1.1237	1	0.32095	303	181	571	214	39	258	45	231	294	213	236	187	174
sw18899	BGIBMGA014171	0.8411	0.3805	0.77045	2.23705	345	1364	280	3481	6277	8	9	86	75	262	359	4	17
sw03882	BGIBMGA004398	0.4284	0.8142	0.6299	0.80055	1096	313	1016	5842	8411	101	53	1161	402	75	99	160	266
sw19541	BGIBMGA004402	0.1582	0.6129	0.3905	0.61895	1021	188	422	2048	1925	8	-6	870	225	3	7	8	35
sw17422	BGIBMGA004400	0.3487	0.8991	0.51785	0.57735	1358	2416	1226	1385	1577	945	641	890	1425	1293	1382	958	1103
sw06038	BGIBMGA004394	0.2231	0.8486	0.42615	0.45485	9066	1803	2204	17884	16861	184	17	4834	921	45	108	139	301
sw01299	BGIBMGA004403	0.0916	0.43405	0.2457	0.37515	6161	1141	508	2227	2916	50	65	627	127	35	39	52	81
sw22899	BGIBMGA004474	1.44295	1.0727	1.3878	0.191	58	176	192	160	9	47301	48	270	295	81	77	59	69
sw14502	BGIBMGA008031	0.10385	0.778	0.5543	1	325	11886	17	25	32	-13	19	57	22	18	42	16	22
sw14615	BGIBMGA005519	0.87205	0.95965	1.08345	3.16445	2981	11452	4116	3582	2461	4156	2865	3591	3239	2009	1569	1306	2451

sw09566	BGIBMGA010322	1.34975	2.16305	1.6701	2.7115	119	2188	4721	112	1	-32	254	76	164	371	388	67	107
sw21874	BGIBMGA005655	1.1068	1.02915	1.00085	2.35555	637	4005	595	451	322	250	208	220	344	322	380	324	270
sw13068	BGIBMGA008020	0.95905	0.9941	1.134	2.11635	721	3005	1338	862	383	679	1258	294	423	226	625	252	361
sw13090	BGIBMGA003269	0.46345	0.723	0.5443	0.71415	212	419	240	327	257	331	198	287	310	154	163	131	102
sw03554	BGIBMGA006720	0.17395	0.65765	0.6171	0.646	149	17662	79	39	14	35	37	77	39	19	13	8	24
sw05348	BGIBMGA006649	0.1481	1	1	0.6407	62	3587	19	11	21	30	16	28	5	28	23	16	36
sw13228	BGIBMGA009370	0.82175	1.05055	1.07955	0.47985	163	581	133	136	136	295	129	183	290	162	197	251	234
sw07919	BGIBMGA002675	0.8808	0.9358	1.10605	0.46	14833	8627	6672	4105	3996	2969	1456	10539	19700	3635	3559	4150	3934
sw08886	BGIBMGA007295	1	1	1	0.4498	19	480	260	936	23	84	12	20	17	47	28	75	77
sw15790	BGIBMGA011320	1.28225	1.1205	0.8797	0.4361	17	-21	30	59	138	4258	68	259	476	5	8	13	6
sw05903	BGIBMGA000387	1.1186	1.2067	1.48055	0.4192	50	583	567	87	265	4785	66	523	835	66	67	89	120
sw19049	BGIBMGA002618	1.2562	1.0298	1.2259	0.3293	1820	1175	2588	1174	322	2423	606	2359	6424	637	558	766	690
sw14297	BGIBMGA003840	0.70715	0.66265	1.01615	3.62745	5114	6367	1516	1485	1516	1648	3198	981	931	3052	2338	2152	3281
sw03582	BGIBMGA010723	0.752	0.8222	0.99735	3.44095	4966	4183	2994	2377	2322	3237	4048	1727	1660	3621	2539	2169	4289
sw19005	BGIBMGA003004	0.69715	0.85725	1.19785	2.8606	13231	10135	4808	5150	6081	6931	5068	3203	3936	14053	9338	11433	15919
sw16419	BGIBMGA008493	0.79095	1.03635	0.87555	2.83305	3065	10686	5052	4798	2566	4960	3293	5549	4103	4092	4294	3971	3968
sw13455	BGIBMGA013100	0.8726	0.9892	1.28455	2.6363	763	701	541	372	477	526	583	337	330	727	473	466	845
sw04110	BGIBMGA004573	0.66985	0.73375	0.8717	2.3079	6256	15797	5057	5116	3477	8600	7982	5272	4417	9512	7079	6360	9639
sw04011	BGIBMGA011062	0.7367	1.0387	0.752	2.22815	741	1925	1217	617	330	582	448	438	283	653	670	613	751
sw14689	BGIBMGA014140	1.5652	1.2809	1.57725	2.03415	1651	883	514	592	524	492	664	561	758	2103	2132	2657	2578
sw15044	BGIBMGA008214	1.2036	1.3133	1.50075	0.48465	17	904	352	69	103	157	92	65	213	28	35	64	90
sw00728	BGIBMGA012264	2.19	1.3607	1.3008	0.74565	39	53	552	2734	73	143	51	112	111	154	181	185	206
sw18036	BGIBMGA003762	0.7267	0.9089	0.9568	4.11825	1419	2296	1047	943	777	2608	829	863	783	2434	1586	1555	2796
sw11250	BGIBMGA003398	0.6223	0.80095	0.72625	2.37225	5928	6592	3179	3719	2364	3182	4412	3350	2575	6184	4727	5548	6913
sw05314	BGIBMGA001283	1.06515	1.3651	1.4077	3.24645	1900	1694	1057	938	1286	1081	1224	568	389	2966	2565	2978	4575
sw01916	BGIBMGA006246	0.7103	0.8057	1.0679	0.48455	77	804	821	374	31	448	112	148	177	201	221	191	234
sw22217	BGIBMGA006419	0.81425	1.0472	1.0895	0.2314	133	5142	223	79	259	-2	304	51	142	36	40	14	33
sw07899	BGIBMGA006869	0.9021	1.1187	0.3674	2.2625	432	129	805	693	560	131	4423	75	84	883	968	664	799
sw15808	BGIBMGA013786	1.1918	1.0391	1.0639	2.48235	3301	2251	1822	960	664	1076	712	1258	1322	2530	3041	2395	2058

sw03543	BGIBMGA007702	0.90555	1.09455	1.1075	3.14595	3839	8374	6516	5433	4611	5558	4218	4122	3408	6351	5764	4742	5780
sw08134	BGIBMGA003351	1.13415	0.98995	1.3533	2.2636	504	441	711	432	348	446	1478	439	418	512	375	348	588
sw20662	BGIBMGA006347	1	1.3296	1.0136	0.45275	1166	1743	423	328	200	199	152	260	361	265	288	312	239
sw11173	BGIBMGA010908	0.90935	1.1003	1.00565	2.37975	469	3459	947	533	216	552	543	331	353	563	694	486	488
sw18095	BGIBMGA010291	1.4322	1.48075	1.6351	2.2649	9	-25	16	13	21	1151	-12	628	1637	7	22		-7
sw16752	BGIBMGA012871	1	1.67115	2.5879	1	-21	1633	-17	-3	15	1628	-18			13	2		17
sw11624	BGIBMGA003720	1.42455	1.4187	0.70535	0.352	251	2397	160	621	1802	4	77	19	45	384	82	8	8
sw22960	BGIBMGA010563	2.26375	1.46315	2.29005	3.48925	573	231	414	6163	9372	61	18	120	84	39	45	56	74
sw07571	BGIBMGA004776	1	1	1	2.2287	10518	5061	5779	3986	4106	3122	4449	2912	3719	6763	6039	5524	7721
sw04532	BGIBMGA014244	1.5091	1.6585	1.9517	7.15805	689	127	674	269	23	1201	-2	57	70	-2	-12	0	7
sw14801	BGIBMGA014257	1.4169	1.44805	0.9596	2.9625	139	105	863	826	1151	263	61	20026	21318	35	74	33	18
sw05128	BGIBMGA001008	1.361	1.00995	1.0833	2.87775	26	-15	1	44	97	868	11	1091	1255	5	6		18
sw21570	BGIBMGA004527	0.42065	2.07875	1.3684	2.72475	-6	-34	601	28	1194	-17	-8	908	48	7985	12909	18071	19035
sw03812	BGIBMGA007280	1.0947	1.46615	1.2448	2.7039	76	136	233	140	264	3622	127	6438	10539	94	104	78	127
sw07664	BGIBMGA011093	0.965	1.2346	1.18425	2.4837	89	440	342	187	50	1542	40	136	128	109	142	72	88
sw06498	BGIBMGA001907	1.42715	0.99955	1.14375	2.36085	-19	-29	5974	5173	25	-28	18	3		-1	-7		-27
sw01621	BGIBMGA013585	0.84575	0.9463	0.9632	2.32165	641	813	608	385	358	483	763	401	430	429	417	384	481
sw04041	BGIBMGA002430	1.19375	0.97915	1.25405	2.2018	1276	87	3134	45	122	26029	80	1049	1244	50	51	29	44
sw19962	BGIBMGA010938	1.44945	1.369	0.7186	2.13725	28	46	24	58	778	176	-18	31261	44581	22	12		15
sw07605	BGIBMGA001007	2.0245	1.3865	1.4812	1.7926	55	134	56	48	42	1070	69	3759	4609	61	35	23	38
sw18642	BGIBMGA003409	2.094	1.27905	1.50525	1.45265	1970	521	698	7834	8726	2152	-5	240	277	24	24	22	37
sw14594	BGIBMGA010881	0.31	1.9238	0.83185	1.28695	24	45	273	49	312	46	21	282	61	2114	2937	7918	8553
sw20654	BGIBMGA002525	0.45865	0.8692	0.49035	1.25505	-10	-25	42	97	197	43	16	4836	10499	40	37	19	28
sw22231	BGIBMGA004528	0.25075	1.2309	0.78955	1	-12	-19	40	12	48	52	10	58	19	272	474	824	939
sw20799	BGIBMGA014242	6.7311	2.5926	2.29675	1	97	290	347	262	13	956	11	63	43	341	356	47	45
sw03301	BGIBMGA013242	2.9008	1.18495	1	0.86435	73	117	2956	445	248	92	61	179	235	134	135	143	201
sw12280	BGIBMGA008872	2.43545	2.3404	1.823	0.5816	28	183	7	6	0	13495	-2	3	85	22	15	6	40
sw15828	BGIBMGA006540	1.11345	0.97395	0.81945	0.50205	69	2532	4721	393	28	56	41	49	55	303	648	41	42
sw17508	BGIBMGA007020	0.95985	1.05165	0.9168	0.4693	6633	6395	8848	8491	4959	8350	3995	5510	8006	7072	8327	4789	5285

sw12804	BGIBMGA011577	1	1	1	0.45795	363	363	442	603	453	55	391	347	1230	354	302	724	845
sw03799	BGIBMGA011489	1	1	0.86495	0.40885	2373	443	726	107	55	203	299	58	38	974	828	1238	1641
sw22019	BGIBMGA009725	1	1	1	0.38065	107	442	28	12	33	106	-2	389	510	30	42	23	31
sw22364	BGIBMGA013477	1	1	1	0.3694	375	547	435	297	231	330	176	200	360	212	234	213	274
sw01427	BGIBMGA008871	1.08125	0.93175	1.33025	0.3377	99	272	452	404	105	602	75	164	199	301	332	544	568
sw20203	BGIBMGA014105	1.4459	1.1992	1.67095	0.31885	1268	1343	10486	1664	502	12232	815	4067	4365	2689	2061	2082	3170
sw17139	BGIBMGA001498	1.06285	1.56295	0.9359	0.2895	23	20	156	122	501	62	12	4722	18723	12	27		22
sw22831	BGIBMGA001599	1.6685	0.8587	1.07935	0.2205	495	76	11912	18327	819	3142	15	161	223	492	658	404	438
sw18903	BGIBMGA002462	2.334	1.98525	0.98925	0.18635	4633	2554	2008	3247	10841	572	1326	798	1238	2393	2445	3933	3948
sw14566	BGIBMGA008982	0.8542	1.00465	0.9087	2.8022	566	904	466	523	293	571	525	1186	1548	567	578	626	592
sw18746	BGIBMGA009879	1	1	1	0.47715	106	1003	2532	969	182	207	16	445	1046	252	253	650	693
sw18783	BGIBMGA000528	1.1408	1.06255	1.2067	0.3204	2811	2956	2026	2527	1798	3470	2055	1328	2450	1587	1576	1169	1347
sw12688	BGIBMGA012456	1.3023	1.5136	1.43105	2.7603	2042	403	12148	3937	7781	47754	9861	27658	37899	134	164	215	159
sw10500	BGIBMGA012093	0.8445	0.8319	0.97855	3.0333	680	1001	307	453	403	754	388	687	750	395	346	341	488
sw11916	BGIBMGA006700	1.31625	1.1321	1.0831	2.87155	143	525	496	322	537	928	234	4842	6704	255	290	225	249
sw05294	BGIBMGA009058	0.1442	1	0.74055	1.536	390	36245	19	39	19	15	24	104	20	-4	-13		-6
sw05687	BGIBMGA002005	0.3162	0.64595	0.7022	1.5251	1374	27951	469	272	177	280	347	317	312	243	250	217	295
sw09312	BGIBMGA012380	0.24605	0.7174	0.75325	1.35075	210	9938	29	60	50	49	31	178	102	56	66	53	78
sw00459	BGIBMGA009057	0.86885	0.8464	1.2403	0.40475	173	261	750	334	579	453	104	320	361	179	197	203	226
sw14404	BGIBMGA002393	0.988	1.0235	1.22195	0.3997	493	351	2067	877	547	837	107	222	337	499	511	469	559
sw16002	BGIBMGA002835	1.33445	1.1532	1.05165	4.3348	1341	1849	664	445	552	540	406	923	879	696	859	922	969
sw09541	BGIBMGA006802	1.2295	1.2016	1.11895	2.18415	4502	6413	4135	3006	1878	1693	2662	2909	3011	2480	2830	2464	2663
sw20681	BGIBMGA012873	0.8395	1.0868	0.8646	3.23285	1512	2112	2168	1924	1124	1379	1067	1365	1482	1073	1242	1084	1186
sw15815	BGIBMGA011495	2.1127	1.5315	0.66685	8.1517	5002	3349	878	10172	11043	262	323	35189	20403	69	77		4
sw22885	BGIBMGA006723	0.9003	1.37795	1.19165	2.621	319	336	1158	941	529	354	272	317	373	4460	6392	1401	1237
sw08470	BGIBMGA005215	0.1696	1	0.71475	1	290	16490	430	42	-6	2	-2	63	4	22	30	21	15
sw09515	BGIBMGA008985	0.4023	0.7586	0.80445	1	408	9948	166	64	44	257	127	193	257	72	91	90	68
sw09643	BGIBMGA006822	1.09555	1.4226	0.84605	2.453	1883	6384	1376	860	698	1050	1250	912	1044	1118	1531	1333	1115
sw13056	BGIBMGA011645	1.1104	1.28025	1.39555	4.2538	215	325	312	216	208	3104	82	203	188	181	146	96	176

sw00457	BGIBMGA006631	0.96355	1.14375	1.01485	3.30645	624	749	748	606	618	2472	669	968	937	1130	1116	545	650
sw19295	BGIBMGA003655	1.3887	1.57885	1.3436	0.42845	1326	642	1324	10288	18346	80	27	995	742	1889	2585	81	218
sw14359	BGIBMGA001581	1.0146	1.0942	1.286	2.9049	6168	6077	3666	2511	1675	2399	3078	2007	2283	4626	4194	3471	3960
sw06402	BGIBMGA007256	1.65215	1.42945	0.9402	2.406	73	1439	3253	5961	134	111	2	140	224	78	84	70	137
sw06317	BGIBMGA007254	2.11605	1.1382	0.7737	1.2429	2596	8140	18426	20446	2785	640	3973	261	480	136	190	132	145
sw17381	BGIBMGA007072	0.63035	0.811	0.57535	0.44685	101	446	723	299	130	578	56	251	400	1817	3735	447	287
sw09140	BGIBMGA000440	1.1209	1.1479	0.91705	2.51055	3784	5144	6249	3646	2273	3993	2010	4153	5792	2742	2598	2241	3051
sw07562	BGIBMGA004942	0.40825	0.70605	0.7259	1.20885	601	18387	281	178	193	164	78	91	122	41	41	29	51
sw08112	BGIBMGA010701	1.09325	1.02725	1.24175	0.49515	203	1147	152	265	515	359	177	575	1296	149	109	123	189
sw13309	BGIBMGA007688	0.98595	1.07175	0.8774	3.18185	4028	8221	3019	2339	947	1090	1646	2004	1874	3841	5267	4103	2973
sw12171	BGIBMGA012794	0.2108	0.57345	0.4372	0.3188	153	124	3506	470	1892	306	123	3472	312	1245	717	520	377
sw00781	BGIBMGA005536	1	1.0928	0.9943	0.42315	225	912	249	144	86	387	402	205	205	215	208	201	303
sw12898	BGIBMGA014227	1.33735	1.163	1.43615	0.30365	30	146	1370	1170	85	151	29	44	52	43	50	43	27
sw20505	BGIBMGA000613	1.1651	0.97025	1.3285	0.2823	308	1027	15257	16608	1010	2473	123	284	455	164	172	122	129
sw08444	BGIBMGA000612	1.3354	0.9275	1.472	0.23565	79	351	8804	7886	381	940	50	76	94	71	116	93	102
sw13248	BGIBMGA012800	1	1	1	0.50415	403	2990	258	329	289	147	188	564	789	55	40	12	68
sw12746	BGIBMGA011007	0.67415	0.93555	0.66105	4.62415	1388	1013	1571	880	400	383	798	444	615	472	595	726	523
sw19867	BGIBMGA007441	1	1	1	0.49785	1153	1938	1626	281	129	153	302	203	195	157	196	123	172
sw13390	BGIBMGA007367	1.11365	1.22515	0.861	4.30325	654	1871	512	318	353	209	131	268	257	330	372	322	439
sw18133	BGIBMGA009182	0.97195	1.10875	0.88315	2.01425	12858	25335	49494	37737	16977	57066	12418	57250	48931	19591	19729	20740	20770
sw16441	BGIBMGA009987	0.7401	1.0698	0.7422	2.7014	4155	2591	13513	10031	6012	12538	2871	13599	11715	8194	8438	11666	11961
sw11507	BGIBMGA000891	0.20415	0.60395	0.8261	1.26105	494	26013	110	60	24	51	1	98	34	38	37	1	-9
sw06240	BGIBMGA011822	0.98065	1.07085	1.1869	2.66615	1391	2517	1115	747	676	830	830	564	670	1277	923	1132	1587
sw13631	BGIBMGA005815	1.3336	1.1196	1.0373	0.4693	406	617	242	424	328	378	374	292	452	437	518	586	457
sw22896	BGIBMGA007727	0.97935	0.82025	0.8806	0.41615	6234	8837	4103	2637	1712	4314	2336	4338	7073	3403	3138	2937	3248
sw17423	BGIBMGA014117	0.9256	0.9292	0.9705	2.93545	1450	2783	571	625	468	2021	741	513	1101	367	351	332	513
sw06396	BGIBMGA006806	0.97785	1.0539	1.00415	5.104	346	286	497	261	275	354	188	555	556	352	347	400	394
sw06803	BGIBMGA012859	0.89855	1.01225	0.9068	2.0851	1682	904	8830	4285	2424	5665	1112	4304	6283	1058	1217	1054	1388
sw12271	BGIBMGA013646	0.57505	0.4812	0.62255	1.7662	1719	3369	496	518	794	876	1562	1012	1893	249	215	109	75

sw15425	BGIBMGA012547	1.11695	1.2948	1.32355	2.9124	4116	5198	1685	3152	1258	2080	2395	1935	1262	6137	3994	5276	8181
sw20968	BGIBMGA009469	0.95715	1.109	1.23735	2.70195	3346	4941	1746	1947	1049	1307	1356	1240	1352	3501	2883	2407	2964
sw21303	BGIBMGA002783	1.25765	1.42015	1.5501	5.9309	1013	573	956	737	559	665	399	336	449	1260	1065	1037	1195
sw04333	BGIBMGA007475	1.39445	1.11215	1.2693	0.44235	42	70	1726	1158	63	246	30	70	72	82	77	103	134
sw15666	BGIBMGA006921	1.02995	0.93995	1.50955	0.40615	458	1538	259	172	164	450	70	125	201	180	190	258	289
sw04142	BGIBMGA006085	1.37935	1.0062	1.707	2.1625	2277	1024	885	886	1390	811	983	575	338	2594	1707	2307	3078
sw09598	BGIBMGA010739	0.99455	1.1308	1.70585	2.12935	9306	4982	3217	3424	5040	2701	5696	2022	1924	11410	9822	10609	12187
sw18132	BGIBMGA006966	0.896	1.10025	0.8674	4.36665	5043	5474	6156	5201	4470	5075	3796	1468	1810	2824	3631	4037	4116
sw04647	BGIBMGA004933	0.751	0.97595	0.7222	3.64715	1500	1843	1650	873	758	785	962	1170	773	1239	1219	1050	1174
sw19824	BGIBMGA014503	1.41625	1.18185	1.4305	0.48115	529	524	294	303	173	293	216	185	274	384	536	558	602
sw22871	BGIBMGA002309	1.08325	1.1373	1.47225	2.29595	36239	21865	16870	20478	13716	24123	21525	13463	12912	37429	24791	21679	33924
sw10407	BGIBMGA010569	1.30985	1.38105	1.21125	9.4806	1185	1562	2373	1358	1067	1985	941	2170	2526	1561	1468	1482	1585
sw04194	BGIBMGA007193	0.9186	1.14815	1.0677	3.5941	1244	1728	496	354	280	185	259	460	582	574	493	778	772
sw09583	BGIBMGA009113	0.6736	1.1058	0.98635	3.12725	1404	14020	1080	818	562	251	493	709	644	1190	1129	943	1120
sw14170	BGIBMGA001806	0.9467	1.20165	1.1921	3.1078	1614	1053	1120	872	747	886	1365	647	760	960	901	704	1045
sw20751	BGIBMGA007261	1.22165	1.184	1.0856	2.8273	326	812	225	283	242	300	195	178	190	163	150	171	252
sw06772	BGIBMGA011509	0.92775	1.2461	0.9406	2.6677	354	356	570	288	238	579	83	150	260	287	326	340	453
sw22830	BGIBMGA005485	1.12465	1.25555	0.86575	2.5913	7036	9485	6092	6874	4152	8429	3477	7000	9259	8019	8133	7741	6949
sw07662	BGIBMGA007301	1.23265	1.1855	0.9169	2.1181	780	1205	1171	583	634	1228	405	1134	1201	486	496	568	597
sw16116	BGIBMGA009046	1.26135	1.47885	1.2874	2.08155	1569	1319	1198	532	533	922	472	421	531	877	894	712	786
sw18089	BGIBMGA008568	0.7759	1.1345	1.05245	0.364	175	638	671	235	213	469	122	133	253	228	224	1024	1185
sw12077	BGIBMGA003667	1	0.96715	1	0.28945	445	1311	302	484	335	368	123	544	719	426	441	329	344
sw22922	BGIBMGA003885	0.3736	0.6353	0.7347	1.2914	1245	27808	498	387	267	664	312	615	740	287	302	311	329
sw22349	BGIBMGA001979	0.7122	0.7865	0.9687	0.47495	649	407	2377	124	36	54	4412	9	26	-7	-3		-10
sw04090	BGIBMGA012496	0.8735	0.6389	0.9352	2.71875	1300	1726	849	625	405	656	540	378	450	709	620	780	1199
sw20476	BGIBMGA007307	0.1575	0.64985	0.6155	0.91955	217	10408	60	84	30	43	25	40	30	33	22	16	37
sw13391	BGIBMGA004067	0.8717	1.17465	0.4881	1	15	23	1090	1627	28	70	20	119	79	31	9	36	46
sw18176	BGIBMGA001303	0.6632	0.77195	0.78345	2.3922	1648	2826	1969	2018	1817	5542	1372	2296	5086	2241	2169	2114	2887
sw11642	BGIBMGA006816	2.26595	2.0494	2.0041	1.17785	10486	2949	2949	3382	6401	514	6649	390	285	8044	9959	10499	8301

sw20309	BGIBMGA005439	0.89935	0.86295	0.77505	2.66875	7924	3660	21484	14118	5819	17366	4168	21459	22539	8189	9063	10036	9462
sw03199	BGIBMGA013120	0.7967	0.87775	0.81305	2.90045	1243	2491	1450	997	910	1772	1463	1765	1166	1182	1092	824	1016
sw13832	BGIBMGA012241	0.2067	0.5821	0.7234	2.0136	222	20112	91	151	96	148	64	197	127	87	81	100	71
sw12257	BGIBMGA008927	1.03015	0.93795	0.85505	0.4008	994	703	897	1053	427	1709	1357	539	1000	446	556	492	435
sw21123	BGIBMGA000056	1	1.0062	1	2.03525	46	268	80	559	195	23	9	123	147	55	51	8	23
sw16511	BGIBMGA010854	1.969	2.19685	1.14545	1.72535	-6	-4	384	80	30	126	12	729	938	24	28	18	31
sw11832	BGIBMGA001003	2.4203	1.16765	1.91535	1.43025	137	353	105	2042	947	125	94	1216	1851	1761	2098	403	374
sw20316	BGIBMGA013237	2.0269	0.9817	1.6017	0.76835	905	357	27462	18495	2995	2172	132	3091	4050	9161	12773	818	1472
sw13622	BGIBMGA011708	0.90395	1.108	0.8801	0.48755	1975	1878	2394	2567	1377	2845	1544	1996	2819	1390	1418	1114	1149
sw04210	BGIBMGA005356	1	1	1	0.44875	835	876	663	417	231	254	356	375	533	733	836	766	716
sw12973	BGIBMGA003926	0.6738	1.2856	0.84715	0.3448	956	751	316	1290	1785	-12	-41	66	136	5	-2		-5
sw19907	BGIBMGA007184	1.676	0.9771	0.79115	0.33095	218	245	1397	6823	120	91	33	998	1285	79	140	28	36
sw19748	BGIBMGA004714	1	1	1	0.32675	1740	1159	1243	967	459	699	290	819	1286	862	1173	849	804
sw09479	BGIBMGA011469	0.68955	0.79465	0.7999	3.8204	501	610	430	377	682	692	384	457	619	504	459	685	841
sw15024	BGIBMGA000689	1.0561	1.05	1.2622	0.3188	191	211	207	208	264	537	186	137	159	189	178	211	141
sw15731	BGIBMGA000204	0.9138	1.1025	1.1066	0.35155	536	402	729	481	361	338	261	243	424	294	409	450	403
sw06559	BGIBMGA012123	1.08265	0.9453	1	2.70315	1591	5503	1174	108	104	100	68	5847	12297	255	229	237	265
sw00748	BGIBMGA004906	2.6885	1	1	1	182	212	148	372	971	286	116	188	177	181	174	243	281
sw05369	BGIBMGA008620	1.08005	1.0117	1.0926	4.6519	435	577	850	457	363	1056	469	976	785	597	508	539	726
sw20117	BGIBMGA011625	0.6158	0.98855	0.7981	0.42585	871	2930	1148	662	378	454	430	307	701	379	432	356	372
sw17363	BGIBMGA013260	0.3358	0.6193	0.3097	0.9037	1134	1202	752	744	689	3133	776	2684	3694	2380	4659	804	753
sw15426	BGIBMGA010010	0.9272	0.47635	0.86345	2.4807	13	152	33095	1547	820	-6	-9	223	16	261	294	179	654
sw20121	BGIBMGA002626	0.4386	0.82735	0.4647	1.74195	346	294	4808	7396	724	1328	201	1908	1945	1530	1652	1490	1487
sw15978	BGIBMGA010039	0.1037	0.4127	0.41545	0.78995	1188	42999	35	77	1411	168	50	201	182	45	36	639	141
sw07840	BGIBMGA001209	1.00695	1.09295	0.95845	2.5746	3236	3692	5987	2919	1946	1745	1524	2227	1930	2035	2109	2634	2446
sw14966	BGIBMGA003669	0.8499	1.1517	1.0892	2.37475	1702	1185	2775	2112	2158	2131	1904	1413	2505	3017	3676	5207	5348
sw00966	BGIBMGA010906	0.8838	0.8988	1.14165	2.71445	4647	3654	1029	876	889	1525	2929	478	329	4018	2727	2593	4041
sw12475	BGIBMGA010521	1	1	1	0.457	52	10728	38	88	106	106	19	23	27	-14	-7		-37
sw14778	BGIBMGA000060	0.8216	0.87795	0.8176	0.44615	658	1172	991	806	652	847	604	754	868	613	592	417	418

sw08603	BGIBMGA002043	0.44145	1.2861	0.6568	0.3188	479	261	89	111	180	93	60	196	188	99	92	76	94
sw11512	BGIBMGA009962	1	1	1	0.27645	476	3045	1176	536	568	205	184	157	312	89	124	156	83
sw09749	BGIBMGA002917	1.0399	1.0831	0.89385	2.3855	1754	3711	1750	1264	332	1541	1498	1383	1221	781	680	645	963
sw03337	BGIBMGA004596	0.89835	1.031	1.12695	2.27585	656	381	795	303	111	1316	629	1107	987	1099	967	759	1053
sw07785	BGIBMGA003506	1	1	1	0.27545	616	281	1202	832	1131	7	1817	88	147	90	92	107	147
sw04091	BGIBMGA002546	0.9758	1	0.76305	3.4214	695	690	1052	526	351	741	485	715	535	698	524	355	448
sw18283	BGIBMGA007137	0.8568	0.9406	1.0921	0.44995	1315	2564	539	417	316	1520	316	400	632	599	680	501	591
sw18325	BGIBMGA004276	0.52465	0.7175	0.77435	0.38085	395	564	1193	2483	1858	819	480	625	1329	199	179	316	468
sw17791	BGIBMGA006893	1.81845	1.4161	0.64985	9.20615	-24	-15	3206	49	-9	-31	12471		11	9	-6		10
sw06143	BGIBMGA011342	2.03015	1.29205	1.48345	1.13975	2249	1859	2271	1497	2225	258	2224	267	525	415	703	647	704
sw01551	BGIBMGA009184	0.7233	0.82515	0.7954	0.4863	37	59	1655	3577	65	143	73	111	100	117	102	64	88
sw18527	BGIBMGA009139	1.19625	1.0796	0.8368	0.46795	494	825	403	846	1465	120	1609	118	278	203	219	177	268
sw12476	BGIBMGA002640	0.6549	0.7802	0.78455	2.033	5285	19684	4213	5253	3247	4394	4725	4987	3286	5729	5242	3459	4299
sw08817	BGIBMGA000097	1	1	1.2215	0.48	585	1224	449	388	191	83	355	284	188	111	127	70	115
sw05428	BGIBMGA004926	1.025	1.14345	1.0486	2.00845	408	1138	318	156	201	361	211	221	199	175	211	165	241
sw18255	BGIBMGA008059	1.1553	1.04595	1.2418	0.5262	48	24	187	83	42	147035	213	373	885	64	80	37	65
sw12328	BGIBMGA010679	2.2333	1.5491	2.3216	0.52185	398	1852	626	228	275	895	219	82	193	210	244	251	227
sw16277	BGIBMGA008061	1.8968	1.4994	1.8734	0.49915	-3	-18	50	50	14	5735	-2	23	166	7	17	7	15
sw09273	BGIBMGA008015	1	1	1	0.4521	-22	-79	756	106	30	-34	203	31	5	4	15		7
sw12110	BGIBMGA008062	1.3642	1.13055	1.24825	0.4371	41	-12	-3	-15	-2	11780	-4	42	152	-9	16	1	29
sw19250	BGIBMGA008060	1.31375	1.03955	1.41085	0.3731	7	13	126	120	89	57729	77	165	469	10	26	8	27
sw04261	BGIBMGA008018	1	1.30745	1.61635	0.3698	-2	12	22	-2	-11	809	14	27	8	19	23	18	12
sw05220	BGIBMGA008017	1.68155	1.35665	1.76995	0.3552	29	7	52	45	28	2763	101	70	63	41	11	23	77
sw21301	BGIBMGA000529	1.3782	0.87595	0.9376	0.43245	1966	573	1082	1604	721	33	352	709	3223	29	18	26	56
sw03365	BGIBMGA004830	1.57225	1.4632	1.4773	2.5897	4	36	82	11	14	3204	1	18	43	24	33	34	13
sw20598	BGIBMGA004800	2.03385	2.15015	1.67795	2.52615	42	2162	20	56	36	18404	11	81	175	160	220	224	172
sw12317	BGIBMGA009476	1.1448	1.21215	0.84735	2.16955	-35	-50	-25	12	20	24418	-4	1821	1138	-14	5		4
sw03831	BGIBMGA008976	0.063	0.523	0.57735	1.1321	582	23211	21	34	5	34	28	94	26	27	18	9	38
sw05148	BGIBMGA002404	1	1.3195	1	0.4774	117	1685	303	77	214	225	-5	205	444	46	24	115	155

sw02098	BGIBMGA003532	1	1	1	0.42015	206	6005	325	101	7	39	37	50	80	45	44	24	31
sw15971	BGIBMGA003037	1.4468	1.04835	1.2142	0.34585	423	914	11932	12769	437	1318	109	257	356	141	223	101	104
sw10606	BGIBMGA012964	0.9896	0.906	1.2198	0.39625	3296	7468	2513	1704	1204	3134	1771	1507	1497	1954	1745	1404	1746
sw19815	BGIBMGA009693	0.49995	0.68335	0.6096	0.38235	19372	19101	17677	30928	16628	32545	13836	23020	27868	22525	22930	19390	19903
sw22573	BGIBMGA011001	2.13365	2.94145	2.3798	1.6842	1026	20461	1954	89	17	395	5124	21		6	1		11
sw01994	BGIBMGA007728	0.69965	0.75045	0.9522	0.50025	817	1123	778	514	1168	1156	615	1160	1964	299	323	415	476
sw13311	BGIBMGA012038	0.82825	0.961	0.9307	7.12655	1691	1925	845	751	562	809	713	811	718	1497	1239	1116	1317
sw20302	BGIBMGA000287	0.933	1.1841	1.14595	2.5286	1093	7847	784	533	574	756	643	683	745	978	1138	1304	1143
sw04913	BGIBMGA003663	1	1.085	1	2.98565	449	1207	496	268	163	287	184	353	492	247	275	389	319
sw09429	BGIBMGA002394	0.87505	1.0975	1.32725	2.68055	1247	10598	584	578	310	521	694	493	658	645	552	843	1231
sw19410	BGIBMGA001027	0.89605	1.2749	1.09135	4.3253	2046	4034	2115	1505	829	2315	829	1157	1124	1544	1344	1038	1646
sw16289	BGIBMGA012810	0.8472	1.0839	1.1496	2.26025	2545	17875	1754	1540	1139	1667	510	1447	1462	2052	1726	1181	1693
sw12542	BGIBMGA012452	1.4982	0.85765	0.9194	2.61475	7	39	50	61	34	7088	4	87	124	12	8		-12
sw14786	BGIBMGA008167	2.74815	1.89655	1.7882	1.1283	189	226	127	152	191	3445	102	60	88	46	82	44	58
sw12323	BGIBMGA003141	0.93965	1.0655	0.92035	2.9058	2344	1918	2071	1454	918	4102	2123	1957	1587	1981	1907	1606	1771
sw17331	BGIBMGA006179	1.5174	1.17685	1.49015	0.4277	73	62	120	141	6	6332	134	66	114	50	41	54	58
sw20460	BGIBMGA001272	1.5142	1.5026	1.83055	0.34425	199	169	224	259	314	7349	65	362	447	91	97	111	135
sw08750	BGIBMGA010225	1.1596	1.1734	1.0893	5.58125	572	630	546	308	330	488	352	249	309	354	378	411	441
sw15884	BGIBMGA006588	1.46795	1.48185	1.4751	2.36505	2512	2823	1327	1134	2362	1764	1397	636	1085	1996	2510	3702	4080
sw04120	BGIBMGA013611	1.37525	1.4308	2.1573	1.1906	18	285	9	29	12	521	35	296	587	16	9	30	31
sw09618	BGIBMGA008478	0.79275	1.25265	1.09185	3.09505	307	752	446	306	373	803	238	682	838	492	473	474	510
sw02423	BGIBMGA008124	0.98405	1.15715	1.04075	0.44565	558	817	1888	1449	1033	1040	716	674	992	373	426	303	319
sw09593	BGIBMGA005106	1.1474	1.2813	1.2933	0.3766	356	355	620	379	142	260	523	200	275	163	148	157	200
sw03948	BGIBMGA008363	1	1	1	0.3912	486	287	134	81	63	115	67	80	117	124	110	94	110
sw20046	BGIBMGA003008	1	1	1	0.41215	312	370	400	347	172	906	189	235	323	154	264	167	174
sw09793	BGIBMGA000803	0.91535	1.0547	1.07175	2.01055	587	684	911	1018	475	755	766	471	706	459	453	439	561
sw00954	BGIBMGA009178	0.4428	0.5751	0.5327	1.1424	70	112	804	772	166	246	87	298	304	203	240	200	196
sw16397	BGIBMGA012910	1.01825	0.94705	0.9983	0.4914	38	542	88	36	12	6	26	81	153	53	48	10	56
sw19831	BGIBMGA007986	0.48395	0.6244	0.60705	0.7911	349	1037	897	737	275	884	320	593	750	230	208	284	255

sw13997	BGIBMGA003583	1	1.6518	1	2.05765	589	707	441	314	237	229	240	357	374	260	321	274	278
sw08189	BGIBMGA009770	1	1.1388	1	0.4684	465	363	449	254	89	24	182	164	165	161	167	79	94
sw10448	BGIBMGA008074	1.01585	0.90745	0.8168	2.5312	2276	4753	3941	2368	2709	7127	1779	6434	7397	3335	3248	3295	4120
sw18406	BGIBMGA005190	1	1	1	0.4831	953	925	724	898	494	385	269	561	1149	561	664	605	571
sw10899	BGIBMGA010644	1.1544	0.93765	1.09765	0.3031	14352	8961	3318	1797	1438	1493	2319	756	1005	1632	1684	1635	2080
sw14332	BGIBMGA006865	1.32885	1.27605	1.36695	0.44555	1746	1725	1998	1771	1566	5226	4085	2858	2335	1822	1346	1137	1846
sw15045	BGIBMGA013171	1.1032	0.65385	0.7609	2.77715	123	744	760	1078	140	94	106	101	94	54	57	59	54
sw07381	BGIBMGA004055	0.94385	1.1345	0.9608	2.4871	467	1346	527	322	262	361	250	264	349	412	454	486	466
sw08391	BGIBMGA001550	0.82105	0.89435	0.9106	2.4451	331	1609	169	137	184	222	242	152	135	252	239	212	248
sw21899	BGIBMGA006623	2.30675	1.29985	0.79105	1.1135	3830	587	903	11541	19961	67	68	414	540	59	47	39	50
sw17922	BGIBMGA001013	0.15385	0.50965	0.6292	1.05975	564	23827	53	68	33	46	110	73	38	11	26	12	35
sw06614	BGIBMGA007238	3.1439	1	1	1	142	725	84	83	69	57	89	72	68	59	55	49	73
sw00170	BGIBMGA004943	0.1182	0.58105	0.66255	0.87225	323	10787	124	161	75	64	92	194	202	145	139	121	134
sw06863	BGIBMGA004028	0.19535	0.57295	0.58195	0.8315	122	4802	52	60	71	66	25	110	100	69	87	75	83
sw03660	BGIBMGA000408	0.8416	0.81845	0.98335	0.4886	467	130	671	732	182	18	244	108	98	152	156	158	182
sw02944	BGIBMGA013134	0.85405	1	1.03555	0.4875	318	466	161	84	79	96	208	75	104	142	154	180	247
sw18519	BGIBMGA008389	1	1	1	0.47845	167	1053	312	259	162	225	71	119	160	71	61	72	55
sw01173	BGIBMGA008336	1	1	1	0.47495	197	616	211	100	66	165	96	147	145	130	97	96	158
sw01185	BGIBMGA006298	1.1829	0.7722	1.14265	0.4724	159	123	9055	6157	335	740	106	190	142	142	175	140	174
sw12903	BGIBMGA003733	0.5505	0.7905	0.94585	0.47195	3335	17236	1006	625	466	521	1146	367	381	575	590	402	518
sw03621	BGIBMGA011755	0.86805	0.945	1.0076	0.43575	1305	3238	1266	523	326	652	674	244	364	245	179	62	121
sw08877	BGIBMGA006810	1.15155	1.26175	1.3775	0.435	722	1019	582	503	256	289	316	317	511	491	527	535	694
sw10757	BGIBMGA001512	1	1	1	0.41115	93	2589	75	59	39	189	51	56	53	9	35	17	36
sw17421	BGIBMGA006461	1.0303	1.01985	1.1226	0.4058	900	1350	1232	1115	879	1025	542	1009	1560	549	511	446	477
sw14428	BGIBMGA004018	1	1	1	0.39745	80	173	434	412	70	-14	9	20	40	45	62	60	66
sw22045	BGIBMGA013442	0.5464	0.9625	0.58535	0.38065	6635	6313	7028	5795	2581	4286	2287	4894	7655	6114	6459	5476	5582
sw11376	BGIBMGA011250	0.9746	1.0696	1.08905	0.3803	834	1123	638	292	163	199	226	65	86	207	159	207	256
sw02441	BGIBMGA009109	1	1	1	0.3397	68	2727	162	77	43	59	41	38	68	28	32	34	40
sw11048	BGIBMGA004712	1	1	1	0.32245	724	880	1027	804	239	340	539	236	454	182	257	222	277

sw15141	BGIBMGA001012	1	1	1	0.32245	19	8314	12	8	18	5	20	24	10	24	2	8	15
sw19706	BGIBMGA003454	1	1	1	0.21365	592	383	344	87	65	403	494	183	261	148	168	166	250
sw09920	BGIBMGA001163	1	1	1	3.0581	234	17819	12	27	13	15	-12	32	10	-4	-17		-4
sw08116	BGIBMGA011429	1.09905	1.14945	1.19755	0.3815	681	766	1955	734	240	496	241	363	681	212	177	99	149
sw17926	BGIBMGA008304	1.07205	1.06445	1.12855	0.347	697	775	690	483	424	183	349	169	222	207	232	218	193
sw07663	BGIBMGA006799	1.1023	1.2427	1.6249	3.83725	88	170	148	74	74	18003	81	144	213	56	61	73	89
sw15165	BGIBMGA012119	0.8777	0.82915	1.1373	0.4421	1230	1565	1515	1061	501	411	1531	263	419	267	271	360	401
sw12388	BGIBMGA001556	0.74865	1.04245	0.73865	2.3791	4904	5323	5693	4154	2293	4533	3317	4023	3459	3783	3936	2957	3685
sw06115	BGIBMGA005687	0.91045	1.2451	1.28705	2.0118	383	1118	280	202	346	483	247	261	343	380	332	328	381
sw09330	BGIBMGA013774	1.44635	1.12355	1.5468	2.7999	812	1688	410	721	853	241	517	433	181	291	384	225	197
sw12305	BGIBMGA010642	0.3678	0.541	0.51485	1.3534	4272	1376	318	211	118	341	-1	1852	927	4	23	50	23
sw13346	BGIBMGA007502	0.78355	1.0569	1.41205	0.26415	92	336	103	111	305	314	114	1100	3084	269	250	813	940
sw12428	BGIBMGA006568	0.8183	0.8749	0.92195	3.10595	998	4617	5069	2185	1204	3096	691	1350	1140	1330	955	928	1481
sw06613	BGIBMGA006213	2.2885	1.18715	1.744	0.6333	1335	1147	37333	42807	668	775	42	991	2265	222	332	191	134
sw09703	BGIBMGA010573	1	1	1	0.18775	-16	951	-18	-4	-15	-33	-11			-28	-30		-25
sw15235	BGIBMGA011810	1.08815	1.0286	1.293	2.30455	776	789	293	134	201	192	203	131	157	338	350	459	551
sw16324	BGIBMGA013348	0.86955	1.0123	1.2337	0.48115	1908	2608	1086	841	798	691	773	451	478	955	1174	1021	1148
sw18382	BGIBMGA003336	0.7564	1.0092	0.95625	2.19025	2341	3525	1824	1501	955	1262	1148	1745	1887	1984	1962	1732	2354
sw00009	BGIBMGA004744	1.01155	1.0598	1.30105	0.49105	492	1031	851	424	277	679	602	337	392	292	258	388	428
sw18206	BGIBMGA006055	0.87525	1.01585	0.7624	2.7377	1766	905	2028	2620	4757	6209	829	24753	25242	676	734	579	618
sw17103	BGIBMGA001561	0.7134	0.68985	0.7184	5.21745	329	466	164	124	152	127	105	129	151	193	209	204	231
sw09558	BGIBMGA011493	0.8274	1.24935	0.89325	3.41835	899	4607	1274	788	896	1220	940	5611	10587	1277	1581	1621	1713
sw01903	BGIBMGA006154	0.6878	0.9907	0.79025	4.12635	1802	2028	3178	2246	2102	4023	1726	3819	4850	1899	1965	1960	2925
sw06168	BGIBMGA006477	0.6352	0.7954	0.994	3.2348	1086	1986	1978	1544	909	1430	1339	564	665	1269	965	1140	1908
sw03236	BGIBMGA005544	0.7791	0.9581	0.90535	2.6582	1159	871	1050	672	609	872	722	786	877	1148	850	867	1402
sw09044	BGIBMGA005628	0.9761	1.3278	1.0366	2.27155	5081	5539	2780	2303	3226	2627	2864	2085	2182	4562	5239	6598	7791
sw04154	BGIBMGA001570	1.14915	1.3521	0.95105	0.2792	169	1542	142	1794	3937	0	-3	60	57	4	6		2
sw11494	BGIBMGA003053	1.4334	1.32725	2.0434	1	532	136	176	285	417	91	298	70	63	148	131	150	257
sw18289	BGIBMGA007842	1.1387	1.13865	1.34265	2.39445	6505	4741	2132	2367	2903	2869	2948	2215	1936	5358	4195	4339	5202

sw12429	BGIBMGA006771	0.6558	0.781	0.8036	3.7781	3646	5100	1226	1686	794	1853	3297	1948	1520	3545	2411	1848	3383
sw03016	BGIBMGA002151	0.8939	1.0902	1.0261	3.6391	1928	3647	1559	1044	1211	1640	1653	1603	853	2137	1381	1101	2088
sw12245	BGIBMGA007849	0.91025	1.11085	0.79125	2.82445	4419	7535	5932	4695	1933	3827	3061	5497	4509	3982	4386	3798	3896
sw09645	BGIBMGA004059	0.7145	0.76295	0.89315	2.6074	11535	10171	11886	16413	10365	28349	14432	9146	11439	11667	9590	5470	8182
sw12581	BGIBMGA003415	0.905	0.89835	0.9727	2.2112	4875	7115	2861	2298	1461	1669	3816	2578	2304	6532	6602	5089	5059
sw06190	BGIBMGA001122	0.2441	1	1	0.87175	124	9244	28	32	14	52	13	44	31	49	54	18	51
sw05818	BGIBMGA009816	1.0969	1.2013	1.45765	3.21265	678	685	395	212	224	246	461	142	159	405	318	300	367
sw09073	BGIBMGA004657	0.67855	0.7135	0.7093	2.4815	5100	7892	5311	5303	2895	4145	4461	4798	4644	6041	4636	4523	5396
sw15132	BGIBMGA003335	0.6283	0.74015	0.71225	2.39805	11125	15743	15245	14129	8310	11737	11671	10840	9299	10613	8974	7051	8772
sw16402	BGIBMGA005083	0.99265	1.0578	0.9791	0.4568	390	267	360	268	229	311	589	132	214	160	154	134	97
sw08964	BGIBMGA001766	1.5144	1.0683	1.17375	2.5555	1266	1607	1314	812	364	1400	1364	1735	1762	817	740	774	940
sw04267	BGIBMGA008689	1.4616	1.25695	1.34655	4.0281	2465	2036	1489	937	923	1039	1109	847	1199	1625	1639	1524	2270
sw20518	BGIBMGA011987	0.42725	0.7452	0.87945	1.92895	2133	41986	846	987	959	641	918	988	1022	3318	3421	3658	2990
sw20342	BGIBMGA004100	0.724	0.98425	0.8814	2.93145	543	3656	263	157	142	68	120	245	200	254	253	163	185
sw14855	BGIBMGA006617	1.12115	1.1133	1.28	0.44625	2192	3109	1859	850	1081	1305	1331	506	704	1077	942	888	1278
sw10402	BGIBMGA005926	0.7454	0.9876	0.8976	2.41755	977	2764	748	699	414	1499	838	779	1790	793	633	732	1121
sw03143	BGIBMGA001444	1	0.8157	1.31645	3.21425	7	-8	3054	9100	57	-5	10	26	17	1	9	16	15
sw02328	BGIBMGA001396	0.7687	0.95055	0.74415	2.07175	465	1010	455	309	309	4581	331	648	715	437	482	365	384
sw20782	BGIBMGA000312	0.99745	0.9009	1.3984	0.4767	592	742	464	349	376	327	162	187	393	594	623	1135	1083
sw20921	BGIBMGA003644	0.9631	1.1688	0.8609	0.41345	420	466	468	615	640	234	294	152	209	188	247	296	278
sw09265	BGIBMGA006906	1	1	1	0.3468	348	1461	217	203	114	79	50	176	344	143	140	177	145
sw01281	BGIBMGA008442	1.8548	1.2141	1.42995	0.48095	310	768	1060	453	319	1309	280	1349	1117	254	283	210	238
sw14699	BGIBMGA009420	0.85965	0.91855	0.99795	0.4901	421	398	2201	854	900	530	112	325	885	199	209	199	257
sw02334	BGIBMGA007111	0.72595	0.7508	0.7914	0.2714	207	440	285	835	2907	265	58	427	1192	236	325	265	320
sw00338	BGIBMGA005391	0.88295	1.0498	0.9083	3.8798	1802	2078	2372	1707	1245	1825	1026	3071	3147	2387	1909	2047	2596
sw15217	BGIBMGA012934	0.99465	1.24035	1.1165	3.6656	1591	3650	1925	1223	719	2821	1868	1445	1690	1252	955	985	1726
sw12286	BGIBMGA003199	0.1101	0.64555	0.7385	1.0947	308	23187	99	175	115	12	131	87	79	1	14	6	-9
sw15226	BGIBMGA002958	0.73225	0.48955	0.3357	0.2497	562	176	2544	655	90	54	-8	70	92	63	131	23	48
sw05837	BGIBMGA000699	1.02245	0.8858	0.8784	0.38735	759	527	361	381	493	89	401	190	122	230	188	147	175

sw11377	BGIBMGA012905	0.8598	1.0147	1.10975	2.0158	547	1305	349	405	330	267	216	210	245	649	386	570	837
sw16856	BGIBMGA004846	0.87925	0.97295	1.12755	3.3802	3289	6448	8003	5250	2019	6450	3346	1928	2260	2799	2728	2042	2632
sw12423	BGIBMGA006751	0.8287	1.0399	1.31105	2.722	11742	12600	8930	7470	8993	7573	7901	5123	5473	9036	9232	9022	10223
sw18664	BGIBMGA001966	1.06205	0.9283	1.1479	2.3422	1386	843	1246	525	523	1429	937	553	734	293	264	205	327
sw05142	BGIBMGA000365	1.1546	1.1735	1.2839	2.1251	4412	7335	4484	2938	1684	10537	3029	3687	2980	3591	3315	1953	2399
sw03975	BGIBMGA006641	0.73255	1.1262	0.89385	2.04065	1393	5509	1910	1045	794	331	931	311	417	651	909	1185	1068
sw11052	BGIBMGA006475	0.921	0.99005	1.02385	0.47385	460	442	419	364	296	234	506	100	135	285	296	290	315
sw04296	BGIBMGA007885	1.09325	0.90625	0.97845	0.4347	1596	634	1064	526	210	1061	1491	344	490	172	239	221	232
sw07546	BGIBMGA005799	1.04505	0.89325	1.05215	2.0451	3393	5743	1901	1750	870	1505	1702	2761	3170	3221	2518	2297	3119
sw06572	BGIBMGA003596	0.8626	1.07595	0.98995	2.81455	1094	2409	777	780	850	1882	762	1655	1975	1452	1057	983	1551
sw18921	BGIBMGA009495	0.7584	0.9024	0.877	3.1722	3600	4211	777	813	735	801	831	547	493	1354	1075	986	1247
sw18784	BGIBMGA003377	1.0114	0.996	1.0742	2.0736	2296	4726	866	683	414	945	687	1114	882	886	748	764	928
sw22941	BGIBMGA005488	0.7896	0.85845	0.9228	2.0508	1467	2307	459	338	305	1017	469	254	310	883	944	570	715
sw08359	BGIBMGA009817	0.6547	0.94475	0.67015	2.8016	1634	1656	1129	1139	639	1588	1501	1009	800	858	638	584	1033
sw12154	BGIBMGA004365	1	1	1	0.4744	228	430	618	499	24	-1	-22	57	67	25	36		-12
sw14618	BGIBMGA001540	1	1	1	0.3555	433	1350	430	223	222	-23	186	120	214	127	167	210	181
sw15040	BGIBMGA013693	0.87775	0.9875	0.96005	2.50375	1031	782	1796	1252	974	2974	912	2718	2508	1242	1076	1033	1214
sw20935	BGIBMGA006615	1.14065	1.1393	0.9764	2.3792	3907	21755	8943	6196	3292	6701	2538	3576	4031	3314	3798	2860	2807
sw12063	BGIBMGA003318	0.9395	0.8735	0.8275	0.4318	6586	4651	3538	1943	952	1508	2372	1582	2196	2618	2338	1792	2028
sw06207	BGIBMGA002407	0.49905	1.2055	0.8424	2.77105	879	4294	826	465	314	393	268	772	682	697	883	707	696
sw09424	BGIBMGA002501	1.00535	0.95805	0.74515	2.72905	2235	3519	4138	2990	3126	9033	1680	29063	26699	2175	2603	2619	2754
sw18217	BGIBMGA014109	0.9927	1.11705	1.229	0.45855	351	1085	463	291	283	343	156	109	196	249	288	290	381
sw06384	BGIBMGA004713	1.01445	0.88365	1.08145	0.438	1719	3920	1103	553	442	290	433	574	501	969	998	702	743
sw12537	BGIBMGA004896	1	1	1	0.2938	2844	4351	342	118	58	-7	662	79	51	49	31		0
sw13345	BGIBMGA014441	1.0167	1.0672	1.1973	0.25815	287	430	325	189	79	299	211	134	279	136	191	135	140
sw16364	BGIBMGA011102	1.21405	1.0064	1.2786	0.44465	359	1183	1155	547	376	1070	466	506	910	269	268	385	354
sw01118	BGIBMGA013531	1.4801	1.11595	1.53265	0.4074	237	3741	13243	13444	472	1500	98	169	171	96	72	80	92
sw22610	BGIBMGA000379	0.9618	0.868	0.92515	0.3406	2810	3377	1559	1294	928	1409	1071	905	1832	1389	1693	2078	1980
sw16297	BGIBMGA000936	0.75095	0.98915	1.0466	2.9376	2623	1685	1281	1771	3009	615	862	2312	2199	1881	1572	1703	2416

sw18226	BGIBMGA009415	0.1069	0.58365	0.73915	0.50605	83	15478	51	27	5	-26	24	5	8	-2	1	57	62
sw14637	BGIBMGA011698	0.4696	0.6479	0.5996	1.2709	3739	16978	1590	12206	16657	104	52	940	350	113	97	74	109
sw16645	BGIBMGA010621	1	0.94375	1.375	2.9801	1160	430	284	356	180	302	287	191	270	1018	529	596	1345
sw09354	BGIBMGA011542	1	1	1	0.4049	857	776	361	375	237	545	694	420	323	236	269	153	198
sw20480	BGIBMGA002208	1.11415	1.0367	1.1288	2.71755	1317	2581	1044	874	489	1380	438	1538	2013	1183	1094	1020	1334
sw18512	BGIBMGA005162	0.83115	0.9651	1.1618	4.2965	1358	1400	1489	1615	1023	1709	647	1860	1452	1532	920	931	2095
sw09737	BGIBMGA006975	0.8017	0.9009	1.08255	2.4999	1617	1732	2145	2160	1149	3099	1761	3796	2926	1974	1513	1391	2065
sw08947	BGIBMGA004112	0.7442	0.91845	0.69535	3.93305	2000	2594	2807	2032	1495	3838	1680	5385	4835	2356	1717	1748	2688
sw11935	BGIBMGA008301	0.84525	0.9884	0.84605	2.1113	4916	9564	7078	5872	2103	9087	5396	11848	8365	7267	5405	4279	6128
sw12191	BGIBMGA006919	0.99875	1.01995	1.09275	4.0259	2834	8870	3868	2946	2098	3647	2443	6956	6221	2946	2187	2200	3074
sw13300	BGIBMGA008477	0.7111	1.03885	0.75545	2.0567	1137	1143	2738	1266	1072	1878	665	1393	1257	1298	1065	1373	1818
sw20069	BGIBMGA000924	0.91415	1.0906	1.0202	4.013	2522	4039	2673	2575	2229	2187	1262	3436	3071	3360	2548	3013	3845
sw14128	BGIBMGA000882	0.57585	0.9283	0.7977	3.22165	5757	8612	6148	6515	5077	8503	5314	6097	5212	8405	6912	7393	9388
sw09702	BGIBMGA003155	1.0923	1.0193	0.9663	4.73675	2528	5567	2846	2383	1372	4854	1874	7103	8967	2279	2331	3375	4393
sw21955	BGIBMGA013567	1.39025	1.25035	1.2365	2.5275	2881	1474	3203	2322	2493	2275	2399	2013	2184	6748	10495	10549	8545
sw01054	BGIBMGA013884	0.9779	1.0388	1.16345	2.0235	364	784	363	316	268	523	409	653	618	607	549	743	763
sw18754	BGIBMGA013513	1	1	1	0.4406	1031	713	447	398	241	253	253	377	449	681	646	747	823
sw05296	BGIBMGA008248	1.0007	0.83925	1.13795	2.251	566	899	647	377	337	649	333	784	881	493	509	603	768
sw21888	BGIBMGA013792	1.39595	1.0894	1.1388	2.21445	7391	5515	9832	6667	651	2779	5302	346	317	234	194	283	405
sw03985	BGIBMGA009934	1.1318	0.95975	0.7892	2.98015	2222	2491	2052	1571	1186	1750	1366	4613	3765	2220	2198	2305	2245
sw08978	BGIBMGA007363	1.1663	1.2517	1.3585	2.35665	33960	15571	40573	41102	27199	54338	37951	37266	31286	32516	32309	29054	30190
sw09461	BGIBMGA004078	1.08165	0.912	1.11435	2.31865	1741	2069	1906	1351	787	1588	869	2439	2949	1725	1245	1255	1866
sw12375	BGIBMGA005279	1.0634	0.98885	1.5038	0.36505	128	539	418	222	32	250	345	349	573	311	295	182	213
sw05413	BGIBMGA007413	0.8786	1.02235	0.8544	2.4385	427	716	318	193	180	179	168	203	231	286	248	358	373
sw03170	BGIBMGA012069	1.36325	1.44845	1.47215	2.0364	1740	809	1424	902	1279	1333	1294	995	726	2033	1649	1694	1978
sw01772	BGIBMGA000464	1.1085	1.0477	1.04175	5.0383	167	657	204	143	119	262	146	200	165	245	234	196	271
sw10427	BGIBMGA010129	1.11935	1.10495	1.26995	2.9818	2625	1981	2131	1904	1535	1896	1956	1221	2141	1985	2134	1806	2057
sw03360	BGIBMGA004913	1.23895	1.39015	1.29165	5.7308	1306	1188	749	570	691	852	683	660	539	1837	1517	1564	2375
sw08168	BGIBMGA004994	1.02585	1.09985	1.1477	2.14525	5331	4597	3492	2877	2562	2354	2332	1594	1527	5033	3261	2988	5147

sw17844	BGIBMGA005739	0.4853	0.83325	0.6231	3.03025	2613	2009	2498	1837	1631	2327	2120	1627	1336	4345	3550	3308	4900
sw06600	BGIBMGA011753	0.67895	1.00685	0.79895	2.88895	943	1076	836	516	544	712	523	1055	848	2051	1622	1681	2256
sw13591	BGIBMGA005469	0.90375	1.1894	0.95545	2.38955	2263	2985	1655	1398	1491	1858	1652	1304	1133	2548	2063	1883	2535
sw04512	BGIBMGA001898	0.94095	0.9373	0.8535	2.2757	5261	2407	3835	2776	2264	4328	2802	1979	2050	5274	3830	2847	4778
sw08472	BGIBMGA010234	0.7818	0.9708	0.8741	2.19625	4324	3287	3589	6214	3803	4213	4813	1748	1623	4356	3964	3504	4675
sw03046	BGIBMGA011994	0.7632	1.00395	0.6681	2.22375	381	732	443	295	226	221	378	427	297	597	637	650	678
sw11597	BGIBMGA001131	1.0424	1.17535	1.2059	5.94735	1615	3072	1051	1083	764	1170	968	888	736	2041	2079	3026	3419
sw08769	BGIBMGA013896	0.8972	0.9976	1.2133	3.0229	2311	4407	1388	1257	763	1062	1676	1242	1222	2645	1752	2063	3184
sw07509	BGIBMGA001188	0.9717	1.028	0.98545	2.6676	1780	4044	1462	740	739	724	734	1216	1131	1868	1877	1896	2307
sw20627	BGIBMGA002460	1.0743	1.01205	1.3058	0.49655	905	1491	670	308	284	357	487	319	629	769	850	826	1048
sw14233	BGIBMGA007266	1.00725	1.28295	0.9557	3.3296	2521	2851	2604	2194	1409	2841	3314	4263	3110	3308	4093	3578	3634
sw11029	BGIBMGA010715	0.1905	1	0.678	1.7076	99	3892	40	22	20	46	33	53	62	52	67	59	55
sw09876	BGIBMGA008291	1.2653	1.22725	1.12595	2.7179	845	641	484	504	526	550	372	713	966	606	582	715	798
sw17903	BGIBMGA009888	1.1189	0.96665	1.178	4.0295	2406	2651	1058	1225	851	1073	1458	806	641	2410	1731	1286	1977
sw15166	BGIBMGA012118	1.04305	1.1025	1.11055	2.9672	9458	6007	4955	4293	3982	4636	5899	4087	3213	10900	7257	6370	9640
sw09177	BGIBMGA007961	0.8957	1.69955	1.47515	2.9148	885	1349	502	484	238	379	452	641	524	771	793	736	881
sw20650	BGIBMGA003462	1.06595	1.13555	1.0171	2.7119	4582	3568	2892	2878	2117	2834	2096	2206	2455	3728	3352	3516	3402
sw09557	BGIBMGA006260	1	1.1749	1.3798	2.45075	259	3952	89	73	64	110	117	77	138	174	207	255	294
sw08909	BGIBMGA005205	1.0697	0.88905	1.192	2.40175	22573	27519	29072	28611	10068	34690	33878	17188	25291	21488	21613	17449	19830
sw10000	BGIBMGA007518	0.80125	0.91265	0.9714	2.3867	4191	4073	3697	2600	1643	2591	2033	2507	2616	2988	2621	2306	2772
sw08193	BGIBMGA008284	0.80255	0.90275	1.0213	2.3167	1309	2609	354	377	457	461	473	269	320	573	523	500	945
sw20862	BGIBMGA009285	0.91965	1.0347	1.1505	2.126	4687	2365	2244	2294	1849	2149	1645	895	921	5823	3877	3425	4486
sw03095	BGIBMGA009277	1.1129	0.9976	0.90815	2.04345	1227	922	678	441	289	368	442	463	480	625	578	515	603
sw21942	BGIBMGA006982	0.3242	0.8611	0.8828	2.02655	940	18936	174	115	40	-8	273	34	13	201	137	172	243
sw13915	BGIBMGA001475	1.014	1.05495	1.2454	0.43185	1151	1809	1165	1038	849	1656	558	452	525	571	470	578	656
sw14479	BGIBMGA009409	0.9923	0.8632	1.1682	0.41135	2933	3008	1574	716	844	963	1224	454	732	1128	942	905	1427
sw11965	BGIBMGA005177	1.2406	1.03925	1.2109	0.3488	5382	4123	3481	2548	1040	1964	2178	2532	3957	2857	3322	3491	3512
sw12539	BGIBMGA004834	1.01355	1.2302	1.3682	6.0197	5337	4219	3911	3173	2601	3760	3767	1964	1518	6542	4464	4011	5662
sw11296	BGIBMGA009849	1.51205	1.1164	1.4853	3.6599	2768	1907	797	794	988	878	1290	816	692	2893	2468	2664	3170

sw09873	BGIBMGA002492	0.86235	1.07675	0.827	7.0131	2418	3800	2285	1955	1000	1990	2351	1531	2095	1938	2422	2570	2791
sw00917	BGIBMGA004302	0.79015	1.0056	1.2162	2.0709	2240	1935	1605	2048	1394	2886	2067	1199	1294	3248	2099	2431	3581
sw15742	BGIBMGA007962	0.82635	0.99125	0.80525	2.8109	4070	5888	3470	2926	2715	4064	3665	3317	2687	4607	4006	3268	4544
sw12743	BGIBMGA008595	0.66385	0.6982	0.74595	2.0638	7815	15475	9607	6392	5017	4996	5187	5883	5006	8312	7226	6794	7879
sw13978	BGIBMGA011687	1.06485	1.15235	1.1382	0.38595	1993	1472	1865	1057	574	830	842	635	1219	902	1075	1033	1154
sw20163	BGIBMGA010338	1.313	1.42945	1.30385	0.3987	39	73	61	187	551	1487	-4	56	135	24	29		20
sw05659	BGIBMGA005625	1	1	1	0.4888	583	370	486	321	241	456	221	179	331	273	278	266	339
sw10102	BGIBMGA007935	2.33715	2.156	1.82665	0.6523	20339	10067	11713	33950	31855	8934	10271	13004	17336	18024	15948	14053	16019
sw04109	BGIBMGA011124	1.31655	1.21995	1.19675	0.2878	82	268	107	69	-28	1658	70	60	67	144	168	124	136
sw15517	BGIBMGA010229	0.98975	1.1503	1.0609	3.3931	4875	2909	3536	3087	1919	2860	3009	2538	2252	3218	2493	2693	3315
sw21174	BGIBMGA010227	0.8875	1.146	1.2645	2.81625	2002	3071	1295	1298	677	1162	701	582	673	1835	1897	1436	1831
sw19810	BGIBMGA014221	1.1467	1.0394	1.01015	0.29445	6148	7071	6940	12019	11106	4599	9392	3895	6291	3340	4610	7445	5546
sw14648	BGIBMGA010419	1.1818	1.09295	1.1658	0.1354	1408	3304	2511	1677	2126	1118	4361	701	1862	916	993	1598	1493
sw16458	BGIBMGA006735	0.84335	1.04675	0.87775	5.1433	1804	2438	1699	1004	798	649	720	1082	951	1321	1217	1220	1265
sw16391	BGIBMGA010699	0.82455	0.92135	0.9053	0.29565	33	18	118	40	52	622	36	69	170	144	169	34	37
sw13320	BGIBMGA010325	1.06055	1.03885	1.05545	2.59295	1345	1385	777	668	693	1830	722	1211	1128	1176	828	788	1235
sw17637	BGIBMGA001929	1.6046	1.32155	1.5941	0.49075	976	782	1359	1329	1032	2766	391	2586	7365	741	729	455	499
sw22147	BGIBMGA003616	1.65545	1.78335	1.1662	3.24465	77	598	9	19	26	776	94	63	69	26	27	27	66
sw14835	BGIBMGA002933	0.402	0.92545	0.68325	3.26435	255	12185	279	175	158	243	114	155	141	127	137	86	121
sw09717	BGIBMGA010171	1	1	1	0.2513	1514	1424	676	534	306	426	404	545	698	941	902	924	1050
sw07544	BGIBMGA013713	0.36805	0.8332	0.788	2.63	3106	2438	2720	2278	4628	5196	2158	1496	1314	4659	4352	5559	7922
sw09161	BGIBMGA014110	1	0.96715	1	0.4657	599	1607	480	456	214	943	1271	2576	876	264	245	206	265
sw14229	BGIBMGA013895	0.9117	0.9107	1.1458	2.42715	903	812	486	752	958	682	674	1241	1096	1069	844	1042	1254
sw16168	BGIBMGA010213	2.07915	1.25435	1.11155	1.21205	3861	2053	24545	23662	1505	537	10578	1100	978	1306	1819	286	345
sw16538	BGIBMGA004726	1	1.47415	0.47005	0.73375	22	79	16	50	12	-2	-6	27	20	2367	4828	960	555
sw22526	BGIBMGA008831	0.2487	0.32995	0.2859	0.4831	30	53	375	127	42	185	-3	59	49	122	222	1359	1073
sw14650	BGIBMGA004961	0.90735	1.06525	1.0583	0.44085	304	544	480	280	182	373	258	202	324	171	213	147	169
sw11427	BGIBMGA011500	0.96845	0.9231	1.4399	2.07995	2066	2601	1662	1334	1480	2169	1966	737	1239	2396	1764	1781	2956
sw22908	BGIBMGA009021	0.86305	1.07115	0.9062	2.95935	4621	2649	4201	3449	1765	3963	2168	2111	2348	3808	3598	3731	3414

sw21531	BGIBMGA005523	1.08395	1.0056	1.02625	0.4268	2652	4231	2702	3244	2724	3838	2155	1819	2796	1473	1389	1324	1511
sw06585	BGIBMGA000624	1	1	1	0.4963	6	745	1185	2405	18	219	11	29	19	40	43	13	16
sw13461	BGIBMGA009778	0.9711	1.0939	1.0962	0.4576	616	507	1120	719	367	1782	1000	1696	2615	609	481	350	489
sw17442	BGIBMGA010072	1.58555	1.3271	1.5659	0.4381	933	1453	637	652	439	2595	907	620	530	403	364	308	354
sw09672	BGIBMGA009282	0.98885	0.9689	1.0581	0.38835	357	747	935	494	374	1288	368	744	1034	305	343	312	302
sw18171	BGIBMGA004550	0.8582	1.04685	1.28255	0.35195	466	612	921	1189	338	3716	636	655	1087	613	528	434	595
sw12513	BGIBMGA003789	1	1	1	0.308	365	1330	608	468	115	214	200	329	539	218	229	204	224
sw13440	BGIBMGA002304	1.1867	1.0756	1.23555	2.31215	1166	702	310	283	322	262	210	190	196	386	343	480	778
sw18010	BGIBMGA012134	1.18375	1.35815	1.2874	3.80225	10	-16	3740	3829	83	7875	663	412	512	34	17	11	31
sw14429	BGIBMGA012834	1.01075	1.1058	1.01435	2.851	566	448	578	472	197	527	307	439	540	334	339	276	409
sw04361	BGIBMGA007744	0.8762	0.8888	0.6847	3.2076	338	1850	260	154	95	132	98	169	137	213	172	203	225
sw20411	BGIBMGA011039	1.10595	0.95445	1.13175	0.5123	1840	3087	716	655	360	816	473	450	631	974	811	598	766
sw09549	BGIBMGA011040	1.2133	1.0022	1.1347	0.4505	1670	3345	812	639	307	929	941	488	627	755	692	652	871
sw01620	BGIBMGA007228	2.23205	1.8417	1.6814	0.89375	56	108	169	63	37	18081	45	181	415	15	0	7	21
sw22862	BGIBMGA014231	0.0954	0.5196	0.6223	0.72205	1382	42777	389	377	200	266	248	320	242	184	173	169	156
sw15180	BGIBMGA008314	1	1	1	0.43805	399	699	292	112	50	95	189	106	194	131	128	119	207
sw05948	BGIBMGA005489	2.23905	1.84965	1.3703	4.8233	-5	-13	11	16	20	1275	10	4150	3152	6	7	12	33
sw00530	BGIBMGA001307	0.70065	0.6014	0.70435	2.3935	504	466	766	851	636	724	590	958	810	550	511	334	474
sw15356	BGIBMGA007043	0.4859	0.88495	0.6543	1.3428	10580	9192	14651	16580	15163	22881	15444	19225	13921	16754	14297	11903	14983
sw09342	BGIBMGA007584	0.9094	0.8298	0.8951	0.44885	1592	3198	1107	703	420	387	576	463	524	660	648	506	509
sw22214	BGIBMGA012171	0.93855	1.0765	1.1641	2.36475	8442	28219	6504	6052	2915	6857	5283	6776	6200	5779	6931	5415	4796
sw00249	BGIBMGA007948	0.8155	0.8818	0.87985	0.41265	1210	1746	1627	1577	817	1456	1028	1633	1823	1173	1024	993	1394
sw17350	BGIBMGA010471	1.45985	1.0922	1.34195	0.24445	304	319	1000	739	222	190	55	137	315	670	797	445	864
sw18883	BGIBMGA005897	0.79005	1.3136	1.1784	3.01135	309	239	369	520	928	64	380	232	208	439	371	396	443
sw05172	BGIBMGA012707	1.2016	0.8592	1.21955	2.39055	1553	1927	617	476	710	611	476	304	461	235	224	210	316
sw05298	BGIBMGA000103	1.1214	1.1881	1.14085	0.4324	17509	14401	14326	9855	6774	13382	16514	6696	9061	17088	15410	10429	15573
sw10635	BGIBMGA008025	0.94345	0.9105	1.2705	0.3654	631	538	509	300	325	425	434	228	361	268	254	412	473
sw16241	BGIBMGA002779	1.054	1.235	1.35	0.46155	1249	1399	522	326	311	434	340	282	331	591	517	611	806
sw14167	BGIBMGA003386	0.6473	1.14135	1.03695	3.4988	546	307	566	437	276	712	385	287	288	515	454	540	838

sw21110	BGIBMGA005199	0.95595	1.08785	1	0.40885	330	919	551	554	172	566	160	275	436	110	121	134	144
sw13384	BGIBMGA012571	0.52445	0.9904	0.65455	3.73465	2204	8735	2977	2669	2486	4772	2904	2619	1921	3227	2554	2521	3518
sw14427	BGIBMGA000925	0.68505	1.00685	0.7108	5.75855	1716	4636	2772	1896	1625	2278	1742	1461	1704	1780	2022	1991	2065
sw17725	BGIBMGA006043	0.8737	0.8671	1	0.28225	1310	1747	304	142	148	77	178	42	54	47	36	45	48
sw15360	BGIBMGA004992	0.8451	0.8981	1.29	0.2396	1890	1506	1860	1797	1514	2426	802	771	2007	3180	3266	3618	4111
sw05071	BGIBMGA003428	1.0397	0.96475	1.4743	0.36595	1418	2556	1799	1766	1604	2628	1076	721	1195	1031	1162	1045	1693
sw21578	BGIBMGA013054	0.811	1.17275	1.2009	3.1246	1817	2586	899	1346	1270	1212	925	1285	1144	2164	1937	2278	2620
sw03056	BGIBMGA012226	1	1	1	0.4667	93	8316	24	48	37	61	31	62	51	76	56	69	71
sw05999	BGIBMGA009000	0.9841	0.9879	1.0848	0.46465	1025	1019	6931	2006	491	1864	324	819	1284	480	551	671	769
sw08430	BGIBMGA010740	0.75325	1.033	1.9917	2.46195	193	85	654	216	63	986	120	84	76	-10	-1	81	58
sw00146	BGIBMGA001664	1	1.08615	1.02705	2.35825	75	371	231	128	81	1511	132	652	666	314	308	280	304
sw18380	BGIBMGA004567	2.4561	2.6275	2.37325	1.99215	13	15	33	47	13	1277	12	34	32	22	28	1	4
sw07934	BGIBMGA010161	1.25645	3.1151	1	1.6297	205	44	381	529	470	27	27	190	45	48	52	2	-2
sw19517	BGIBMGA004538	0.391	1.31275	1.037	1.57195	-12	329	236	51	284	-33	-16	52	5	3342	5687	9028	7449
sw20682	BGIBMGA004525	0.2889	0.82155	0.80395	1.0155	-19	-31	150	-29	218	-33	-1	114		1058	2206	3252	3276
sw09295	BGIBMGA014054	1.32415	2.23735	0.73585	1	3	48	31	28	42	74	7	14325	25251	1	3		20
sw09008	BGIBMGA000224	0.4934	0.7554	1	0.621	128	10217	152	169	96	78	82	90	73	52	67	41	67
sw16519	BGIBMGA004436	2.02545	1.3108	1.3945	0.60425	646	1158	315	643	789	865	327	134	124	123	72	81	92
sw00332	BGIBMGA002635	1.88475	1.33835	1.19615	0.45	1543	970	3277	2660	2799	1141	1323	2835	3324	2380	3623	3794	2628
sw19884	BGIBMGA000223	1.34255	1.3305	1.2446	0.265	311	389	8849	1537	196	298	238	3615	5692	468	617	191	207
sw03808	BGIBMGA005319	0.5746	0.95895	0.92705	3.95505	1728	3412	2240	1885	1760	2623	1263	1076	1025	2004	1567	1525	2560
sw07824	BGIBMGA007568	1	1	1	0.3688	495	555	749	290	237	147	37	99	103	14	37	28	48
sw14492	BGIBMGA007569	1.44765	0.87255	1	0.3662	1665	1439	1575	866	436	627	59	292	518	33	59	141	88
sw07704	BGIBMGA007038	0.71165	0.49895	0.66005	1.2838	971	1238	2928	2227	1866	664	458	574	620	2230	2920	341	655
sw14362	BGIBMGA000722	0.829	0.89965	0.87285	2.24855	1633	2617	2710	1987	1154	2347	1327	3140	3655	1817	2004	1949	2132
sw21457	BGIBMGA007844	1.0404	1.2234	1.10845	2.2618	4506	7574	3619	2227	3139	3622	1996	1776	2697	4322	3622	4147	5131
sw20312	BGIBMGA012488	1	1	1	0.4533	209	539	560	368	55	23	11	86	124	41	58	21	25
sw20070	BGIBMGA004577	0.9036	1.0255	1.06665	0.2883	1286	3175	1253	1030	651	1435	552	878	1419	939	942	706	915
sw08022	BGIBMGA003803	1.1984	1	1.08545	0.43695	246	687	237	252	144	518	138	223	440	134	175	182	164

sw22878	BGIBMGA007971	0.7124	0.9394	1.05065	0.45715	538	812	524	516	879	1199	595	333	531	711	674	1016	1261
sw10381	BGIBMGA010620	0.78545	1.10255	0.89415	2.9361	893	1278	790	580	683	935	595	634	697	876	888	781	1036
sw06500	BGIBMGA007579	0.9858	0.9712	0.8829	0.4842	931	1759	760	391	271	557	497	349	455	378	402	343	395
sw10405	BGIBMGA013918	0.82425	0.9437	0.73695	2.278	4194	4297	2431	1750	964	2142	2858	3774	2228	4003	3822	3526	4042
sw01504	BGIBMGA001273	1.23195	1.26535	1.3782	2.8458	442	1360	292	502	691	2593	178	165	215	68	63	53	64
sw13232	BGIBMGA010449	2.0431	1.1627	1	0.71775	1335	1733	1114	734	517	298	453	291	557	420	798	783	520
sw22880	BGIBMGA010437	1.1852	1.17225	1.06705	4.60875	4114	15748	5251	3295	2156	3959	1935	1803	2498	2510	2538	1843	2091
sw18837	BGIBMGA005925	1.17905	1.02825	1.06705	0.4252	1472	984	1104	755	603	859	631	543	868	568	639	638	653
sw02991	BGIBMGA013936	0.3368	0.67425	0.7889	1.55935	594	25780	392	343	137	302	198	453	361	273	284	306	292
sw22843	BGIBMGA014477	0.1448	0.473	0.3255	1.19745	909	14157	1073	1304	650	1133	487	704	641	1251	1268	3551	2711
sw15658	BGIBMGA006290	0.0924	0.5991	0.66855	0.70775	568	22969	36	55	19	-33	-13	24		-1	15		5
sw10865	BGIBMGA001022	1.2468	1.08635	1.17635	2.42775	1110	160	860	10422	6350	49453	81	22533	31292	110	55	31	52
sw15683	BGIBMGA004120	0.2872	0.64095	0.90655	1.22245	266	11281	170	99	41	126	118	164	145	159	165	190	201
sw03188	BGIBMGA002999	0.9438	1.0292	1.07095	0.49745	1508	1842	2326	1548	932	2278	741	1012	1402	1037	1047	1021	1218
sw07451	BGIBMGA011737	0.8821	0.9949	1.00635	3.73595	1660	8968	1293	876	634	1088	962	903	1423	1075	967	993	1269
sw11034	BGIBMGA010172	0.8264	0.96515	0.79255	2.59665	525	1040	998	661	409	611	662	650	512	582	471	376	548
sw09767	BGIBMGA012459	0.7214	0.9415	0.5915	3.6876	408	3441	994	537	365	668	659	586	576	517	570	505	519
sw06082	BGIBMGA005878	0.9099	1.0804	1.1478	2.13235	1128	824	727	541	405	319	419	440	415	826	535	475	842
sw14681	BGIBMGA006292	0.9122	1.22015	1.0464	3.88025	1035	1647	1017	925	500	1047	949	629	507	984	804	730	1257
sw05749	BGIBMGA006173	1.0239	1.36025	1.18215	2.92505	3404	4055	1726	1737	776	1910	1860	1518	1248	3414	2353	2903	4280
sw18441	BGIBMGA004618	0.29725	0.78015	0.8078	1.0022	1443	39432	359	343	146	164	177	656	286	225	268	213	174
sw16046	BGIBMGA011324	0.83605	1.01135	0.9949	4.6096	2389	3843	732	755	910	678	545	309	247	1617	1068	992	1540
sw05452	BGIBMGA004950	2.3673	1.85645	1.50785	0.2559	636	1015	299	1776	5263	28	36	51	106	136	188	208	226
sw18568	BGIBMGA002973	0.8514	1.01655	0.92365	2.7661	2717	4460	2430	1703	1564	1913	1707	1058	1376	1898	2445	2334	2318
sw14472	BGIBMGA002755	0.7449	0.94925	0.8946	3.83025	6250	3512	2211	2050	1550	1953	3340	1832	1950	3300	2850	2439	3249
sw13266	BGIBMGA008342	1.0062	1.1024	1.1623	2.39865	731	1439	566	528	345	288	450	440	410	504	348	256	404
sw12010	BGIBMGA013537	0.75565	1.64065	1.55565	0.2682	91	822	402	203	227	631	136	109	377	214	166	210	341
sw13503	BGIBMGA014181	1.2032	1.14815	1.33615	0.4241	452	492	1201	728	284	612	249	299	521	201	223	206	254
sw08920	BGIBMGA009051	0.73615	0.93465	0.74585	4.21495	685	528	484	648	442	926	532	1184	1230	626	600	593	637

sw16228	BGIBMGA006401	0.5594	0.67325	0.84125	3.01805	3386	9602	5258	4874	2845	6497	2489	5771	3910	11822	8437	4679	5649
sw20012	BGIBMGA012172	0.77885	0.82385	1.0531	2.211	682	2020	366	297	167	302	293	357	382	188	186	171	249
sw18808	BGIBMGA012505	0.78265	0.85265	0.8679	2.1932	1077	1921	1418	1299	842	1869	335	2127	1450	1936	1808	1763	2310
sw11674	BGIBMGA001711	0.8581	0.9512	0.88055	2.1683	5056	19359	7753	5907	3049	5281	7388	4253	2922	6126	6383	5822	5669
sw17713	BGIBMGA008199	0.8495	0.7935	0.9031	0.4895	654	2011	445	687	644	3554	1829	2010	2765	679	606	604	802
sw11740	BGIBMGA000661	0.77255	0.9003	0.94075	0.4805	609	4022	181	286	463	373	200	79	206	137	164	177	207
sw22957	BGIBMGA007904	0.84965	1.0666	1.06945	0.4642	412	7498	368	442	267	385	169	269	478	200	222	167	221
sw14074	BGIBMGA011844	0.842	0.88585	0.95495	0.2226	3148	2750	2573	4413	6184	4190	2189	1757	3635	3836	4402	12115	11952
sw11486	BGIBMGA000412	0.7044	0.97255	1.03	3.7749	1162	1586	1422	1050	755	752	574	767	911	1077	838	842	1336
sw16054	BGIBMGA001137	0.7686	0.87105	1.2503	5.96815	2002	1492	519	575	478	842	591	159	125	1581	978	2433	2742
sw21674	BGIBMGA011334	1.2692	1.09745	0.90575	0.3332	3917	4328	2245	2667	1908	6323	2961	954	955	739	796	434	492
sw18459	BGIBMGA006039	0.9727	1.1169	1.0061	0.46545	1412	1432	1684	2854	2065	2758	1382	1271	1850	1233	1367	865	1025
sw17578	BGIBMGA010496	1.0121	0.90995	1.3058	0.4059	512	757	1367	1970	459	1245	393	635	930	395	404	383	399
sw06845	BGIBMGA009073	0.7973	1.0846	0.78215	2.15985	213	148	883	746	351	225	122	270	226	4724	5155	405	726
sw18400	BGIBMGA004727	0.22395	0.6916	0.4914	2.1383	88	62	81	72	50	8	4	47	62	1938	2697	669	733
sw15875	BGIBMGA006235	0.4254	0.85295	0.61605	0.87245	478	716	10011	735	7277	554	234	2946	816	50383	47506	39469	43276
sw19642	BGIBMGA004728	0.37015	1.3325	0.51565	0.44445	38	109	157	102	63	37	25	65	49	2013	4291	1096	597
sw03300	BGIBMGA013243	0.72195	0.91495	0.85715	5.07955	1334	1777	1730	1838	827	1954	856	2291	1969	1224	739	662	1047
sw06001	BGIBMGA002215	0.91935	0.9811	1.30515	4.05285	604	1049	817	622	366	1082	577	527	563	505	283	400	1148
sw13347	BGIBMGA004278	1.07305	1.0967	1.0178	3.837	4253	4185	7311	4184	3583	4883	2934	16024	15349	5930	5722	5193	6569
sw17077	BGIBMGA004734	1.09235	1.0698	0.9154	2.61615	880	1133	1454	1385	1022	1103	453	933	969	633	815	1184	1072
sw11111	BGIBMGA008714	1.05795	0.97815	1.08745	2.24695	1784	2187	1177	735	681	910	840	2805	2913	1117	949	957	1024
sw15709	BGIBMGA006226	1	1	1	0.31385	467	199	490	390	419	83	109	474	1163	30	40	21	18
sw18001	BGIBMGA002649	1.13305	0.9909	1.11705	0.4551	1011	1126	532	309	447	997	483	378	699	511	502	419	474
sw17344	BGIBMGA007565	1.4527	1.35195	1.3651	0.44465	1790	1938	2331	1312	917	2152	266	848	1649	681	1038	937	779
sw14920	BGIBMGA012759	1.14825	0.93115	0.9319	0.31275	828	1400	749	725	554	1653	472	543	767	370	356	309	389
sw06731	BGIBMGA004099	0.86205	1.03945	0.7686	3.6723	3368	4132	2348	2360	1646	4335	2900	4493	5271	2748	2993	3259	2959
sw13698	BGIBMGA003614	1.2254	1.20025	1.19055	0.4361	2612	1626	2349	1931	225	604	962	397	525	776	970	413	369
sw10616	BGIBMGA000801	1.31455	1.258	0.976	3.20745	1889	2183	2351	1381	1010	3392	1302	1468	1946	1396	1899	1923	1572

sw17121	BGIBMGA012220	0.8696	1.08255	0.99725	2.0715	2088	2772	1991	1624	1265	1881	964	1496	1721	1739	1381	1349	1666
sw02046	BGIBMGA009240	1.24405	1.06895	0.7687	2.04055	308	499	472	293	207	744	290	335	393	179	206	156	216
sw21138	BGIBMGA007107	1.0425	0.9319	1.2476	2.02195	1691	2951	975	699	519	421	509	614	749	1115	879	772	940
sw18131	BGIBMGA007772	1.74765	1.34885	0.91275	0.49505	1732	4101	1058	541	834	1134	729	1255	2171	417	789	669	533
sw22877	BGIBMGA010595	0.9663	1.06165	1.19205	2.40045	7318	12674	3418	5286	2686	3774	3179	6032	5927	5177	4749	3999	4378
sw06874	BGIBMGA011827	0.9761	1.1317	1.1057	0.50535	1122	1660	747	383	382	421	394	340	537	436	516	506	643
sw20332	BGIBMGA011069	1	1	1	0.37355	183	570	82	53	207	236	266	94	250	66	66	67	82
sw13737	BGIBMGA011695	0.6495	0.65345	0.4226	0.94835	605	2336	107	869	1090	53	14	639	544	114	168	889	501
sw16063	BGIBMGA012718	0.8898	1.1182	1.2806	2.12285	842	3214	1061	873	552	1317	610	522	736	684	707	661	885
sw15101	BGIBMGA000289	0.1329	0.5571	0.5306	1.53455	278	13531	15	20	6	1	-5	54	9	8	0	9	24
sw13075	BGIBMGA003718	0.6258	1.0061	1.28135	0.47035	2770	5508	5860	8706	9406	18315	5995	2484	4280	9723	11380	17297	21055
sw14215	BGIBMGA000926	0.6608	0.81265	0.79755	3.0561	7837	5039	10424	13957	20078	11524	5759	13240	12227	9586	10086	7685	9236
sw18597	BGIBMGA011424	0.81125	0.62405	0.53695	0.42965	2715	2465	6559	26680	25393	933	2061	519	1057	281	356	239	267
sw18818	BGIBMGA012221	1.03895	1.20855	1.3262	0.27485	881	378	734	1285	900	6109	704	870	2301	174	177	88	116
sw17505	BGIBMGA001982	1	1	1	0.4841	170	958	161	106	57	123	30	97	59	84	96	85	115
sw20915	BGIBMGA001976	1.03665	0.97425	0.8362	0.1866	4263	2610	6411	15524	1661	1445	408	230	288	191	318	96	90
sw01513	BGIBMGA009974	1.21595	0.9889	1.0316	0.28205	228	627	695	455	179	426	199	336	454	307	277	295	393
sw11955	BGIBMGA002750	1.11465	0.8622	1.08295	2.02895	688	1553	4464	2115	1277	2148	428	12401	15624	611	756	1059	1041
sw11197	BGIBMGA009156	1.14525	1.00925	1.12825	0.4227	10432	11092	12126	11295	8723	15231	8817	8344	11542	10677	11199	10955	12444
sw00306	BGIBMGA014211	1.06055	0.95085	1.2978	0.38225	3845	1683	3015	3488	8515	4213	2821	2278	3350	3502	4149	5361	5446
sw18482	BGIBMGA009726	0.95295	0.923	1.2149	2.9461	8329	10698	3646	4196	2995	5215	3534	3257	3087	7999	7439	5461	5199
sw07804	BGIBMGA004644	0.95735	1.14935	1.00835	2.8039	3019	2873	1953	1557	1154	979	1040	1225	869	2027	1896	1781	2026
sw08745	BGIBMGA003530	0.23005	0.54185	0.4096	1.40515	1068	4397	5051	796	337	539	806	1818	1648	930	1035	996	901
sw14453	BGIBMGA011281	0.8466	1.1639	0.7589	3.7645	2057	2899	1435	1294	992	2038	955	2497	2086	1819	1427	1440	2005
sw14663	BGIBMGA005158	0.7342	0.9889	0.7119	2.40195	3338	11942	4726	2640	1872	3719	2580	2891	2868	2942	3189	2493	2696
sw04522	BGIBMGA002766	1.18965	1.3139	1.38135	3.04575	649	641	401	239	229	261	253	223	229	427	363	455	571
sw17734	BGIBMGA004806	1.42785	1.09845	1.43305	0.2484	32	20	148	40	8	57752	123	405	728	15	8	2	10
sw01677	BGIBMGA011771	0.6309	0.759	0.9597	3.80325	380	706	343	188	167	401	246	220	165	368	319	479	743
sw13821	BGIBMGA011019	0.7455	1.00075	1.0145	3.58675	1772	1347	1603	1757	729	1543	958	1322	1234	2593	1930	2209	3292

sw16619	BGIBMGA011775	0.7545	0.98095	0.8485	2.713	910	1173	632	337	474	865	500	385	550	598	612	699	852
sw17132	BGIBMGA005126	0.7895	0.9502	1.1252	2.4587	4206	4130	1670	1373	1407	2205	1156	1159	1266	2643	2258	2190	2954
sw20893	BGIBMGA003440	0.8657	0.843	0.88935	2.545	2851	4584	1499	1116	845	1034	802	1717	1715	2116	1745	1802	2193
sw01135	BGIBMGA014337	1.53955	1.1553	1.2914	0.22395	8405	7336	12489	7560	4185	6719	2271	5306	8249	3085	4806	5620	4934
sw08981	BGIBMGA001031	0.32505	0.65995	0.5313	0.48535	1071	1338	4401	1503	1271	983	442	1962	949	576	556	240	252
sw00689	BGIBMGA009276	2.7305	1.3968	0.99665	0.5107	4496	10219	1224	5384	12667	1324	527	644	584	125	125	114	124
sw03972	BGIBMGA008513	1.31615	1	0.9673	5.1106	4	3	38	43	-5	1450	24	12	27	38	50	36	41
sw17483	BGIBMGA010061	2.1242	1.42655	1.43805	4.0534	104	114	57	76	13	16080	12	130	156	63	61	48	61
sw07771	BGIBMGA012478	0.7941	0.8599	1.05255	3.1573	11	47	9	21	11	10712	9	45	68	-14	-16		-12
sw06092	BGIBMGA008514	1.02765	1.024	1.07705	2.8975	34	41	207	45	23	39147	103	8	32	46	45	28	39
sw20945	BGIBMGA010303	1.14895	1.0944	1.13545	2.73285	164	171	1121	1439	337	-9	3255	0		1	8		9
sw19175	BGIBMGA008280	0.6639	0.82295	0.77955	2.5218	-33	-70	7	13	-9	4394	-16	6	1	4	-4		-24
sw01248	BGIBMGA009750	1.43475	1.3775	1.53135	2.4111	67	472	166	154	82	19147	290	397	1391	60	66	29	42
sw01127	BGIBMGA010276	1.25865	1.26425	1.34445	2.3877	62	94	146	102	53	15692	168	472	441	307	386	252	252
sw03707	BGIBMGA010584	2.0209	1.4018	1.62485	2.37195	-1	6	46	30	5	73282	144	337	842	12	18		-7
sw01671	BGIBMGA007377	0.8036	0.5139	0.5572	2.239	8	30	61	51	34	29300	109	205	188	190	195	102	97
sw21935	BGIBMGA003566	1.1065	1.4469	1.3035	2.09185	26	40	49	50	9	133611	161	286	1572	60	56	36	41
sw00838	BGIBMGA010590	0.9947	1.8814	1.8615	2.0716	127	1014	212	113	66	8493	225	240	530	88	91	158	162
sw02096	BGIBMGA001320	0.2954	0.14645	0.75875	1.25435	23	52	109	61	28	37145	110	252	750	119	104	111	170
sw12994	BGIBMGA006747	0.35795	0.6103	1	1.18485	1313	35417	95	115	232	-35	-11	6217	6926	-1	7	24	15
sw13094	BGIBMGA008101	1	1	0.34805	1	935	144	99	957	2108	16	14	50	63	3	-7		11
sw08448	BGIBMGA010063	4.36115	2.0319	3.1085	1	-32	-74	926	946	15	10835	-14	7	40	-31	-19		-32
sw09635	BGIBMGA012427	2.68015	3.387	2.53755	0.7349	131	106	1971	1655	71	129	28	140	198	8	36	8	24
sw20743	BGIBMGA008668	2.05	1.4427	1.22315	0.56615	140	164	1325	1274	206	45	9	29	75	9	12	25	16
sw08436	BGIBMGA013698	1.17255	1.0619	1.25225	0.495	514	429	543	576	471	709	442	356	552	254	287	231	293
sw10762	BGIBMGA001091	1.655	1.1852	1.2883	0.47365	18594	3376	3559	13476	18818	59	8671	605	1001	68	70	52	96
sw15794	BGIBMGA001836	0.7317	0.82395	0.69925	0.31375	907	575	1745	2274	4069	174	2557	57	82	19	31	26	32
sw13012	BGIBMGA007058	0.08045	0.4751	1	1	380	20922	8	19	28	-26	-14	30	6	-9	-7		-14
sw09407	BGIBMGA006153	1.27705	1.0442	1.13265	0.478	1145	1130	1283	976	738	827	388	405	803	643	854	1021	858

sw04295	BGIBMGA003296	0.12305	0.2817	0.3204	1.0625	142	2661	98	91	28	124	55	145	84	111	139	105	121
sw11356	BGIBMGA004681	0.0392	0.51035	0.5689	0.6145	1259	59121	88	101	45	-18	-10	443	15	10	-13		-3
sw17349	BGIBMGA001707	0.91605	0.9006	1.1551	0.4178	6262	9752	5769	4755	3546	4766	5343	2182	3903	4809	7532	5926	5417
sw12398	BGIBMGA013328	0.6684	1.3033	2.05725	0.36335	580	582	478	611	755	1113	528	425	588	1697	1473	2267	3607
sw15985	BGIBMGA012059	1.26765	1.276	1.35535	3.08205	946	937	480	527	610	499	275	434	510	928	879	1319	1680
sw04425	BGIBMGA012348	0.67655	1.09585	1.2681	2.6233	299	391	443	470	590	2102	377	289	414	630	595	617	746
sw02395	BGIBMGA010038	2.11825	1.4194	1.27615	0.79475	118	379	1092	759	115	376	57	153	245	52	72	63	76
sw04644	BGIBMGA003119	1.23915	1.07805	1.16075	2.199	1950	2220	2560	1706	1420	1783	702	2190	2284	1361	1141	1226	1551
sw17051	BGIBMGA012550	0.51685	0.79585	0.74475	2.15955	10061	10400	9650	17921	11326	28258	12968	12384	10302	16873	13177	14173	18536
sw11176	BGIBMGA009911	1.06155	0.89825	1.108	0.43755	12374	25283	11241	9669	5859	10765	12196	6142	8778	9298	10855	7872	7906
sw10652	BGIBMGA012761	1.03195	0.93345	1.0812	2.4694	258	383	452	271	185	468	176	658	723	332	292	289	350
sw06760	BGIBMGA012554	0.9475	0.88025	0.78175	3.01105	1787	4490	2907	1290	1101	2861	934	2160	2136	1777	1694	1502	1929
sw13572	BGIBMGA012671	0.9722	1.11595	0.885	2.21885	2200	3596	2090	1161	1136	848	869	1050	1062	817	1080	1141	1164
sw15487	BGIBMGA006464	0.99995	1.3431	1.05995	0.45095	958	1681	2539	1527	1010	2141	479	1120	2214	756	867	1117	1208
sw13453	BGIBMGA012689	1.04135	1.07455	0.95305	0.43735	2791	3419	1221	1216	802	1535	1699	948	1118	962	726	862	1273
sw08392	BGIBMGA001551	0.9424	0.8965	1.0503	0.28495	3117	5612	2914	1960	1906	3436	2034	1278	1785	1741	1802	1648	1846
sw15021	BGIBMGA013972	1	1	1.35835	0.2648	115	576	239	155	126	356	69	145	597	144	124	146	113
sw12259	BGIBMGA008926	0.76225	1	1	0.4921	643	542	457	294	204	280	276	83	156	204	201	227	264
sw06028	BGIBMGA005338	1	1	1	0.3443	377	1059	275	221	220	384	181	176	224	303	294	236	353
sw15802	BGIBMGA010446	1.3912	1.21075	1.29625	2.0597	5365	7491	2741	3612	2625	3193	2482	2107	2241	4691	4308	2927	2899
sw12164	BGIBMGA007676	1.49775	1.3862	1.09835	3.3473	518	787	253	218	235	173	180	198	202	329	312	381	463
sw18729	BGIBMGA000731	3.2003	1.2652	0.97225	1.10685	20	3	2573	1656	605	78	200	226	406	89	83	64	77
sw18488	BGIBMGA011368	1.24315	1	1.44545	0.47965	94	1113	435	294	26	137	21	99	110	124	110	87	98
sw15458	BGIBMGA011631	1	1	1	0.4317	597	1008	293	246	171	265	119	290	475	216	187	181	282
sw09525	BGIBMGA007317	0.02855	0.56055	0.61545	0.3958	269	44310	2	19	-8	2	-14	98	24	3	7		-8
sw00708	BGIBMGA007315	1	1	1	0.37435	230	559	251	230	153	336	178	172	206	249	231	262	339
sw09861	BGIBMGA012857	1.09565	1.08055	1.02055	2.9023	4223	3515	17159	9195	4691	13101	3668	14377	11773	4407	4325	4378	5257
sw15838	BGIBMGA001609	2.216	1.5924	1.59785	0.91905	122	1438	394	591	129	419	72	108	110	146	153	213	187
sw12759	BGIBMGA008725	0.96505	1.155	1.29915	0.5011	1540	778	2599	1668	1987	1745	1120	703	766	728	735	1931	2128

sw13662	BGIBMGA010288	1	1.3618	2.75155	9.87	-29	-63	1	-10	-6	4485	-26			-33	-23		-23
sw19970	BGIBMGA013834	2.0471	1.67165	2.15655	8.8402	-13	-33	19	14	7	1969	7	26	17	-11	8		-9
sw21445	BGIBMGA013862	1.8573	2.11865	0.8267	2.8915	87	68	129	97	59	1660	41	873	1093	61	58	33	45
sw06973	BGIBMGA014622	1.4251	1.1281	1.4232	2.47775	676	862	318	427	213	1071	260	881	1275	478	452	507	591
sw09395	BGIBMGA004965	0.20365	0.63825	0.65895	2.01865	-6	-19	166	20	232	21	16	110	36	1951	1572	3664	4384
sw19370	BGIBMGA010289	1	1	1	0.30655	-12	-15	49	27	45	3	4	91	86	338	388	963	1015
sw14285	BGIBMGA013625	1.15125	1.01765	1.01745	4.84265	5391	7819	1443	2402	1130	2676	3622	2402	2752	3460	2308	1890	3541
sw10650	BGIBMGA013784	0.76385	1.30625	0.8591	2.33295	1458	2779	745	552	480	354	717	466	501	837	870	835	1075
sw08839	BGIBMGA001797	0.47465	0.9065	0.71755	0.7802	349	503	383	134	173	123	72	241	164	159	141	162	174
sw18363	BGIBMGA011884	0.88905	1.073	0.9493	4.0937	1160	1093	1074	844	818	1585	588	1111	1073	1155	1223	1169	1270
sw21407	BGIBMGA006219	0.7739	1.0804	0.75605	3.0108	1101	2903	3008	1698	1531	2998	1023	2192	2586	647	573	624	860
sw09837	BGIBMGA009504	0.69285	1.04265	0.8559	3.41405	432	353	409	353	351	399	342	296	216	566	515	436	602
sw17152	BGIBMGA009118	0.72305	1.0022	0.9122	4.42585	2418	3970	1998	1535	1163	1233	1399	626	575	1299	1214	888	1028
sw17304	BGIBMGA001343	1.2077	1.09835	0.9301	3.0005	2576	2582	2407	1383	1251	1083	979	866	1107	1006	982	784	808
sw10526	BGIBMGA000627	0.882	1.0975	0.88945	2.47395	3296	4707	2924	1354	793	1424	1773	1357	1886	1467	1610	1426	1430
sw03474	BGIBMGA005425	0.80235	0.92485	1.1197	2.1232	3449	2371	1789	1381	1380	1124	2439	1240	1392	2548	1981	2381	3467
sw16679	BGIBMGA009157	0.97995	1.05955	0.8194	2.10765	3741	8161	2158	2106	1347	1983	1985	1882	1941	1724	1917	1415	1397
sw12981	BGIBMGA011963	1.10685	1.0018	1.02085	0.48735	591	2097	471	286	161	649	120	242	330	143	207	156	210
sw10986	BGIBMGA012320	1.0015	1.7574	1.08755	0.45785	1098	2516	977	1078	2582	843	649	353	553	446	511	538	457
sw22884	BGIBMGA002918	1.01065	1.07925	1.0936	0.45325	2630	5485	2026	1147	815	1376	849	950	1414	880	838	860	1001
sw08108	BGIBMGA001333	2.2427	1.59245	1	1.6743	416	756	423	2203	4914	95	7	82	95	0	-10		-9
sw03204	BGIBMGA000937	0.70975	0.89055	0.8685	2.3642	348	1650	491	213	312	425	232	488	395	743	676	636	760
sw10850	BGIBMGA013703	1.0191	1.18615	1.0361	2.4993	3498	2686	2140	1827	1474	1328	2025	2000	1804	4768	5504	5881	4271
sw04095	BGIBMGA005598	1.5945	1.4924	2.095	0.97075	1128	795	399	256	342	334	364	261	264	522	507	587	769
sw09128	BGIBMGA002432	1.4285	1.12075	1.47905	0.39725	691	362	765	132	181	7090	302	2858	9268	189	319	197	163
sw06061	BGIBMGA005520	1	1	1	0.47595	920	1613	703	260	126	184	486	203	331	314	312	293	343
sw04649	BGIBMGA013375	1.6956	0.97145	1.3158	2.48755	62	254	699	1423	98	37	966	82	87	82	94	53	64
sw05088	BGIBMGA000120	1.0096	1.095	1.1647	0.32805	1134	1708	1260	599	497	581	733	357	474	426	472	371	364
sw13760	BGIBMGA001159	0.94765	0.99935	0.82945	2.17015	4576	14524	5298	4446	3161	4524	2451	3985	3845	4541	4322	3996	4407

sw09540	BGIBMGA001920	0.7609	1.01835	0.82335	2.90675	2084	3582	2917	1511	1073	2499	1558	2193	2662	1171	1292	1140	1270
sw12530	BGIBMGA008358	0.4194	0.5254	0.53745	0.7831	165	335	1560	1878	396	408	69	506	740	272	339	208	209
sw12056	BGIBMGA012240	1.26145	0.955	1.0983	0.4383	1991	712	1321	3421	5985	358	112	355	544	1361	2043	2026	2034
sw10912	BGIBMGA007558	1.293	0.8929	1.14315	0.4149	4238	6954	9808	18438	23854	298	149	568	915	109	78	54	74
sw13989	BGIBMGA002847	1	1	1	0.3829	1632	1394	367	234	23	40	162	60	98	280	196	67	175
sw04436	BGIBMGA001197	1.10135	1.1996	1.07385	3.6625	805	4309	709	445	389	423	436	359	373	557	567	642	871
sw16745	BGIBMGA008226	0.99255	1.099	1.2799	0.43405	954	3196	1204	1186	705	1107	517	464	960	921	814	1223	1598
sw17661	BGIBMGA008016	1.49455	0.8778	0.8958	2.5569	3580	5249	387	3678	7670	73	48	187	133	36	47	36	48
sw22874	BGIBMGA005130	1.16045	1.27945	0.9834	0.3263	3002	478	5906	568	962	328	8981	62	80	6	22	14	27
sw20506	BGIBMGA006491	1.0369	1.28255	1.03985	0.4195	486	747	219	261	148	134	92	105	172	170	210	155	126
sw04084	BGIBMGA006805	0.9338	1.0415	0.8619	7.77445	584	523	687	334	305	502	281	616	532	374	408	478	454
sw10771	BGIBMGA003177	1.41385	1.16195	1.2861	6.0671	2065	5411	1541	1311	1543	1587	1166	1028	919	3241	2220	2379	3590
sw22332	BGIBMGA004847	0.65875	0.9139	0.99995	4.9277	1134	1186	1224	968	855	1375	534	960	1313	884	1071	1228	1361
sw09105	BGIBMGA009395	1.15365	1.3337	1.70365	4.55455	4415	1980	1785	2066	1379	1622	2318	1291	1152	3849	3441	4120	5613
sw03347	BGIBMGA005636	1.05755	1.03405	1.2643	3.8256	1793	1242	622	531	449	640	838	448	362	1518	1058	915	1556
sw11364	BGIBMGA006632	0.91005	1.0876	1.17785	3.2657	2599	2200	831	900	1040	914	1375	678	455	2683	1593	1355	2544
sw17810	BGIBMGA012127	0.88685	1.1207	1.52025	2.97695	2623	1620	1600	1613	1660	2415	1449	1247	1890	3993	2997	3569	5227
sw15388	BGIBMGA010998	1.12125	1.28755	0.78255	2.7585	4721	7017	3207	2524	2163	2192	799	2791	3457	1370	2062	1631	1448
sw14514	BGIBMGA007239	1.43545	1.1856	1.5324	2.6101	1858	2659	958	575	991	531	685	378	478	1308	1586	1856	1932
sw15879	BGIBMGA012025	1.4312	1.44445	1.45095	2.5154	5779	6663	2306	2738	2731	2791	2462	1370	1543	8307	4932	5375	8817
sw13472	BGIBMGA013491	1.09075	1.06145	0.92675	2.46795	2401	1545	1629	1201	1056	1011	1220	668	865	1248	1229	1317	1587
sw16263	BGIBMGA006887	0.9271	0.9869	0.93835	2.46545	2776	2253	1133	1732	2137	1611	1706	810	884	940	1007	846	1085
sw14403	BGIBMGA007889	0.8986	1.1072	1.04815	2.41055	11719	12573	9731	9265	6910	7124	10772	9634	10270	11572	12607	13911	12907
sw17127	BGIBMGA002361	0.7056	0.66355	0.7648	2.34735	554	1478	310	240	183	317	267	326	406	780	651	752	937
sw03736	BGIBMGA001509	1.2058	1.08815	1.1597	2.32825	3855	14114	2852	2086	3010	1888	2268	2235	2331	5229	5017	4624	5397
sw10352	BGIBMGA005930	0.94875	1.14605	1.1182	2.29525	1291	964	556	487	509	649	506	619	763	432	416	367	567
sw18379	BGIBMGA010388	1.1116	1.13135	1.309	2.0945	4063	3444	1701	1979	2183	1644	2034	1038	863	4496	2959	2867	4507
sw00666	BGIBMGA007431	1.0868	1.16955	2.06445	1.77845	1405	2334	448	230	205	487	399	199	218	500	335	312	687
sw14496	BGIBMGA004947	0.2469	1	1	1.6076	181	213	569	333	878	160	62	1106	157	647	549	401	317

sw01378	BGIBMGA012166	0.4526	0.7311	0.7529	1.0418	545	16245	209	169	67	105	103	160	182	194	190	227	240
sw00752	BGIBMGA006087	0.37415	1	1	0.75405	285	13499	282	169	94	153	74	107	179	127	123	150	175
sw19093	BGIBMGA007583	0.8701	0.91145	0.88865	0.5049	2026	3358	3009	2143	1444	1940	824	1506	2222	4620	6807	1365	1466
sw16590	BGIBMGA001038	1	1	1	0.47545	909	903	198	210	230	430	226	185	310	164	176	184	604
sw10943	BGIBMGA005916	0.90895	1.021	1.10355	0.4492	849	4294	560	360	256	560	250	251	390	402	434	423	472
sw01636	BGIBMGA008706	1.05915	0.98755	0.9317	0.40185	1440	1704	2918	1968	1294	1425	701	1404	2080	980	997	935	1000
sw19848	BGIBMGA002375	0.97145	1.02925	1.1207	0.38995	3683	4346	4200	4210	2231	3989	1974	1854	2448	2607	2392	1872	2334
sw03215	BGIBMGA005503	0.7993	0.89585	1.12805	0.2933	241	760	281	131	93	268	111	134	175	131	136	218	216
sw03059	BGIBMGA007637	0.46535	1.15335	1.0144	1.08745	6104	3627	3637	2719	2444	3832	2266	2943	4078	5839	3941	12450	16655
sw20515	BGIBMGA013783	0.0311	0.60135	0.5252	1.17535	399	17856	7	26	10	28	-7	65	28	7	15	12	17
sw03458	BGIBMGA010016	0.97745	0.9744	1.0889	3.1805	3426	5667	1776	1398	1632	1964	2650	885	875	2979	2599	2796	3539
sw03123	BGIBMGA003682	1	1	1.2979	0.14665	298	145	824	246	76	444	13	175	240	205	193	317	297
sw09679	BGIBMGA011697	0.8127	1.0748	0.8774	3.2649	1064	2275	823	396	379	293	523	315	398	588	636	990	682
sw06104	BGIBMGA002096	1	1	1	0.4477	1101	1001	652	368	317	448	360	336	349	288	198	177	246
sw10979	BGIBMGA005216	1.174	1.51195	1.3552	2.90235	1773	2082	1965	1305	1871	4844	1641	2085	3804	1558	1842	1780	2394
sw10137	BGIBMGA005565	0.5099	1.22355	0.54005	2.04355	1487	3331	1557	1301	590	1224	1295	1838	1584	1515	1537	1427	1522
sw08576	BGIBMGA012606	1.87615	0.71925	0.99895	3.434	26	112	11610	8923	66	46	19	127	142	131	166	94	101
sw22041	BGIBMGA001781	1	0.9066	0.65205	0.4291	854	917	1179	1077	511	689	460	726	1274	728	822	750	607
sw06996	BGIBMGA009453	1.0119	1.05165	1	0.4825	286	434	420	233	215	124	227	149	203	164	166	159	178
sw11161	BGIBMGA007407	1.05785	1.0239	1.082	0.46925	114	850	158	160	73	295	169	121	156	147	102	72	140
sw01327	BGIBMGA002780	0.42065	0.66845	0.5433	0.75615	878	3311	4209	4146	1169	1546	730	1653	2241	1300	1275	1077	1331
sw05605	BGIBMGA001623	0.9114	1.05535	0.9138	3.82895	2767	2292	1777	1035	676	1168	1418	986	1059	1850	2702	1993	1707
sw22947	BGIBMGA009009	1.0098	1.1762	1.30555	3.4869	1065	614	701	802	1041	583	377	672	669	1216	1130	1362	1578
sw17253	BGIBMGA012850	0.82675	1.1189	0.92825	3.39575	1167	6391	726	524	438	673	562	510	457	767	980	1458	1315
sw18021	BGIBMGA002948	0.996	1.10075	1.10435	2.63925	797	887	441	356	309	396	156	224	230	113	154	156	149
sw12065	BGIBMGA007864	0.7271	0.6181	0.9974	2.6208	1832	534	385	1821	1709	774	2005	10950	4691	2786	2399	490	632
sw10495	BGIBMGA002752	1.06775	1	0.9236	2.21945	1496	1283	809	673	585	1163	1156	714	957	767	747	567	901
sw14722	BGIBMGA010720	0.98345	1.04865	1.2907	2.211	1454	1128	757	489	488	768	832	559	724	706	661	611	811
sw16144	BGIBMGA008746	0.87265	1.0275	0.8905	0.4999	1642	1607	1418	1382	687	937	825	673	985	863	991	667	828

sw02208	BGIBMGA007189	1	1	1	0.49125	501	1718	199	126	89	135	163	129	136	200	218	194	287
sw15792	BGIBMGA007188	0.75305	0.8185	0.9222	0.48805	851	3505	460	237	162	234	233	116	222	269	319	256	303
sw16625	BGIBMGA003913	1.01355	1.02855	0.9757	0.4733	245	227	393	256	238	190	145	158	259	372	318	449	596
sw15492	BGIBMGA007760	1	1	1	0.4597	873	621	365	263	278	108	75	74	160	133	188	127	118
sw12833	BGIBMGA008808	1	1	1	0.4503	1057	301	679	422	49	352	131	260	453	32	34	24	34
sw20792	BGIBMGA013122	1	1	1	0.4429	424	745	505	269	88	201	120	169	237	229	204	196	185
sw03476	BGIBMGA005305	0.93165	0.9843	1.0086	0.4302	462	676	224	155	134	191	170	117	167	108	160	260	209
sw01328	BGIBMGA005268	1	1	1.2919	0.42465	127	1066	304	134	175	253	63	196	451	91	107	137	167
sw02532	BGIBMGA009783	1.02155	0.9996	0.97655	0.42315	4625	4516	8738	7024	3041	4023	2875	5011	7263	3694	3994	2857	3267
sw14296	BGIBMGA008429	0.83215	0.9954	1	0.37065	1611	6784	666	295	212	436	223	232	369	153	271	186	153
sw18675	BGIBMGA001723	0.19595	0.648	0.4237	0.35555	613	638	2797	747	1747	607	364	3007	501	1995	1321	1548	1214
sw17697	BGIBMGA004431	1	1	1	0.34035	137	809	119	134	34	50	18	79	105	154	190	191	174
sw07932	BGIBMGA000147	1	1	1	0.3322	361	1064	574	453	203	339	421	307	487	204	216	138	157
sw17616	BGIBMGA007089	0.77045	0.85595	0.971	0.24015	2667	3958	1938	1217	908	1543	1035	886	1036	1298	1406	1738	1892
sw03847	BGIBMGA002370	1.1255	1.08535	1.0468	0.49645	977	4613	509	289	268	322	380	203	254	322	496	393	333
sw06889	BGIBMGA004301	1	1	1	0.4226	206	12316	32	-3	11	-9	-6	22		-7	-5		-5
sw12404	BGIBMGA014126	1.1069	0.98915	1.11075	0.40805	380	397	2545	3475	271	853	214	230	387	176	208	113	140
sw18055	BGIBMGA006767	1.2769	1.1886	1.1233	0.3621	215	221	227	455	616	511	158	140	279	112	120	110	102
sw12042	BGIBMGA010596	1.302	1.3354	1.49745	0.46735	661	1998	575	234	130	1022	231	165	165	181	201	115	148
sw06023	BGIBMGA007077	0.8272	1.05635	1.01395	0.3922	817	1635	497	388	298	380	270	237	324	301	406	515	391
sw13179	BGIBMGA008337	1.0928	0.95285	1.0602	0.30675	1727	3739	2073	2240	706	655	1607	564	1037	655	742	487	490
sw09102	BGIBMGA009901	1.24245	1.1374	1.58715	4.21515	364	838	360	349	410	429	282	684	726	484	414	497	701
sw11158	BGIBMGA011920	0.8187	1.19005	1.58025	2.38175	13993	6371	8701	8258	11106	6992	15247	5587	5573	29802	18049	16188	28600
sw22882	BGIBMGA005064	1.22665	1.05575	0.9807	2.19385	4718	3617	4703	9740	1867	7085	1873	5236	6012	1967	2199	1592	1681
sw12534	BGIBMGA000555	1.43135	0.9433	1.0677	2.6939	199	1335	2560	2655	114	68	35	133	192	62	104	63	60
sw04869	BGIBMGA011061	0.9434	0.9536	0.81315	3.30495	630	1344	667	359	359	606	507	363	377	465	386	394	650
sw05309	BGIBMGA006778	0.8327	1.0596	0.71245	2.12115	193	1210	221	145	80	121	139	116	183	199	256	285	282
sw01104	BGIBMGA000320	1.0181	0.8504	1.1564	3.07275	782	1040	543	415	525	595	340	379	403	829	860	912	1087
sw18354	BGIBMGA001675	1	1	1	0.47105	698	697	143	73	30	74	62	36	63	107	155	159	193

sw13955	BGIBMGA004691	1.3593	1.2494	1.0337	0.33345	343	334	602	331	314	182	175	106	211	96	119	133	118
sw18259	BGIBMGA004852	0.709	1.1294	1.3714	0.275	1723	1924	1097	973	1526	1765	925	980	1948	1586	1495	1928	2797
sw05264	BGIBMGA014607	0.75155	0.9502	0.71735	3.1974	4461	6638	7131	4880	4492	9286	4919	15324	9114	6087	6102	6389	7796
sw06665	BGIBMGA003070	0.8652	0.99665	0.8712	3.1649	1354	1501	1643	1415	1234	2406	1382	3662	2302	2270	1865	2413	3009
sw17479	BGIBMGA009941	0.8262	0.941	1.2255	0.4995	289	670	214	81	69	170	166	738	659	175	128	150	267
sw15773	BGIBMGA004500	1.1694	1.19795	1.2427	0.42885	1924	8228	1172	974	778	1294	1152	822	1002	906	755	783	1185
sw17529	BGIBMGA007519	1.94665	1.8354	2.41355	0.4104	20	25	148	160	39	82	393	58	68	52	38	34	39
sw11453	BGIBMGA012371	1	1	1	2.72095	443	114	941	65	91	769	3095	411	821	31	15	17	28
sw20508	BGIBMGA002971	0.91105	1.099	1.0143	2.26785	9543	17323	12495	15927	12646	32353	11462	23650	21361	14991	12659	10856	15602
sw06638	BGIBMGA005004	0.95575	1.06035	1.4765	2.10475	511	326	924	394	152	666	135	155	264	34	33	5	23
sw19766	BGIBMGA007829	1.0696	1.36325	1.40585	2.0475	77	75	121	114	199	3125	58	107	283	44	36	40	39
sw01150	BGIBMGA009681	1.14395	0.96455	0.89145	0.36615	282	166	4912	2926	187	137	66	215	202	665	1037	1058	918
sw07683	BGIBMGA007649	1	1	1	0.163	61	32	565	369	23	-2	-12	50	41	195	209	129	182
sw19762	BGIBMGA005738	1.24585	1.1023	1.1462	0.30125	1533	1221	6495	5969	1308	2439	1661	946	1504	438	674	478	438
sw00881	BGIBMGA002620	1.34165	1.2698	1.02155	0.22115	3561	3642	3201	6118	2452	4515	2599	1206	2131	1430	1273	746	883
