# Supplementary material for: A Genome-Wide Survey for Host Response of Silkworm, Bombyx mori during Pathogen Bacillus bombyseptieus Infection
Source: PLoS One. 2009 Dec 1;4(12):e8098. doi: 10.1371/journal.pone.0008098 (PMC2780328; doi:10.1371/journal.pone.0008098)
Supplement: Table S4 — The ratios of genes mentioned in this report. (0.03 MB PDF) [file pone.0008098.s004.pdf]

Table S4

The ratios of genes mentioned in this report

Pentose and glucuronate interconversions

Gene code	3h	6h	12h	24h
BGIBMGA004965	0.20365	0.6383	0.659	2.01865
BGIBMGA005500	0.52775	0.6429	0.6427	0.25505
BGIBMGA014453	1.93	1.1488	0.7836	3.923
BGIBMGA007258	1.1382	1.1119	1.1196	4.09205
BGIBMGA006727	1.0466	1.4222	1.1938	2.2653
**Citrate cycle (TCA cycle)**				
Gene code	3h	6h	12h	24h
BGIBMGA011412	0.8806	0.9678	1.0681	0.46495
BGIBMGA003815	1	1.086	1.2276	2.01795
BGIBMGA007783	0.1047	0.6254	0.4015	0.24595
BGIBMGA007121	0.92185	1.0731	1.0881	0.38225
BGIBMGA004276	0.36435	0.5732	0.6391	0.5798
BGIBMGA004130	2.59595	1.1754	1.7725	1.40755
BGIBMGA012780	0.9419	1.0905	1.2917	4.88875
BGIBMGA012226	1	1	1	0.4667
BGIBMGA008442	1.8548	1.2141	1.43	0.48095
BGIBMGA000672	1.323	1.1435	1.3928	0.3359
**Pyruvate metabolism**				
Gene code	3h	6h	12h	24h
BGIBMGA002750	1.11465	0.8622	1.083	2.02895
BGIBMGA001966	0.88185	0.9552	1.3227	0.42715
BGIBMGA006419	0.81425	1.0472	1.0895	0.2314
BGIBMGA004276	0.36435	0.5732	0.6391	0.5798
BGIBMGA001068	1.0169	1.0259	1.1808	3.00565
BGIBMGA014453	1.93	1.1488	0.7836	3.923
BGIBMGA004130	2.59595	1.1754	1.7725	1.40755
BGIBMGA008983	0.85765	0.8998	1.0321	2.5808
BGIBMGA007508	2.2004	1.4279	1.2052	2.0384
BGIBMGA008442	1.8548	1.2141	1.43	0.48095

**Pentose phosphate pathway**				
gene code	3h	6h	12h	24h
BGIBMGA001570	1.14915	1.3521	0.9511	0.2792
BGIBMGA014211	1.06055	0.9509	1.2978	0.38225
BGIBMGA009156	1.14525	1.0093	1.1283	0.4227
BGIBMGA000936	0.75095	0.9892	1.0466	2.9376
BGIBMGA008096	0.7581	0.6969	0.9548	2.4095
BGIBMGA000926	0.6608	0.8127	0.7976	3.0561
BGIBMGA008478	0.79275	1.2527	1.0919	3.09505
**Butanoate metabolism**				
gene code	3h	6h	12h	24h
BGIBMGA002750	1.11465	0.8622	1.083	2.02895
BGIBMGA007408	0.8938	0.829	0.7816	3.244
BGIBMGA001966	0.88185	0.9552	1.3227	0.42715
BGIBMGA004001	0.97395	1.1714	1.1154	2.88085
BGIBMGA004229	1.48985	1.6544	2.3666	1.3332
BGIBMGA008633	0.37615	0.5679	0.4739	1.43765
BGIBMGA008621	1.2601	1.2114	1.3012	3.44515
BGIBMGA012226	1	1	1	0.4667
BGIBMGA014599	1.6495	1.4539	1.6398	2.69265
BGIBMGA004489	1.1834	1.027	0.9293	2.38455
BGIBMGA001929	1.6046	1.3216	1.5941	0.49075
**Tryptophan metabolism**				
gene code	3h	6h	12h	24h
BGIBMGA007408	0.8938	0.829	0.7816	3.244
BGIBMGA001966	0.88185	0.9552	1.3227	0.42715
BGIBMGA004776	1	1	1	2.2287
BGIBMGA005733	1.1634	1.2771	1.0759	2.0274
BGIBMGA001068	1.0169	1.0259	1.1808	3.00565
BGIBMGA008621	1.2601	1.2114	1.3012	3.44515
BGIBMGA009276	2.7305	1.3968	0.9967	0.5107
BGIBMGA007842	1.1387	1.1387	1.3427	2.39445
BGIBMGA007424	0.93305	1.0064	1.4591	2.84965

BGIBMGA007146	1.3606	1.4722	1.1077	3.33585
BGIBMGA004489	1.1834	1.027	0.9293	2.38455
BGIBMGA001929	1.6046	1.3216	1.5941	0.49075
BGIBMGA002958	0.73225	0.4896	0.3357	0.2497
BGIBMGA012866	1.54545	0.8363	0.338	3.50685
**Histidine metabolism**				
gene code	3h	6h	12h	24h
BGIBMGA001966	0.88185	0.9552	1.3227	0.42715
BGIBMGA004776	1	1	1	2.2287
BGIBMGA007842	1.1387	1.1387	1.3427	2.39445
BGIBMGA002958	0.73225	0.4896	0.3357	0.2497
BGIBMGA003199	2.24395	1.8658	1.4726	1.14575
**Valine, leucine and isoleucine degradation**				
gene code	3h	6h	12h	24h
BGIBMGA007408	0.8938	0.829	0.7816	3.244
BGIBMGA001966	0.88185	0.9552	1.3227	0.42715
BGIBMGA014181	1.2032	1.1482	1.3362	0.4241
BGIBMGA004001	0.97395	1.1714	1.1154	2.88085
BGIBMGA008621	1.2601	1.2114	1.3012	3.44515
BGIBMGA001237	0.79085	0.7905	0.6198	0.29885
BGIBMGA004489	1.1834	1.027	0.9293	2.38455
BGIBMGA001929	1.6046	1.3216	1.5941	0.49075
BGIBMGA007497	1.1581	0.8857	1.1833	0.40885
BGIBMGA001237	0.93125	0.825	0.8514	0.40505
**Urea cycle and metabolism of amino groups**				
gene code	3h	6h	12h	24h
BGIBMGA001966	0.88185	0.9552	1.3227	0.42715
BGIBMGA007424	0.93305	1.0064	1.4591	2.84965
BGIBMGA011001	2.13365	2.9415	2.3798	1.6842
BGIBMGA012866	1.54545	0.8363	0.338	3.50685
BGIBMGA007716	1.2122	1.0999	0.9352	2.15855
**Aminophosphonate metabolism**				
gene code	3h	6h	12h	24h

BGIBMGA004776	1	1	1	2.2287
BGIBMGA007842	1.1387	1.1387	1.3427	2.39445
BGIBMGA011813	1.06015	0.9537	1	0.4621
**Nitrogen metabolism**				
gene code	3h	6h	12h	24h
BGIBMGA014491	1	1	1	0.4897
BGIBMGA002647	1.2126	1.2699	1.1177	0.2744
BGIBMGA007111	0.7617	0.7618	0.7218	0.24875
BGIBMGA007025	1.2407	0.9817	1.0806	3.33975
BGIBMGA006507	0.6543	0.934	0.7823	3.5569
BGIBMGA006703	0.2436	0.6517	0.7663	0.85335
BGIBMGA013477	1	1	1	0.3694
**Pyrimidine metabolism**				
gene code	3h	6h	12h	24h
BGIBMGA013054	0.74495	1.0787	1.0351	3.05985
BGIBMGA010642	0.3678	0.541	0.5149	1.3534
BGIBMGA005628	0.9761	1.3278	1.0366	2.27155
BGIBMGA010234	0.7818	0.9708	0.8741	2.19625
BGIBMGA006292	0.9122	1.2202	1.0464	3.88025
BGIBMGA011753	0.67895	1.0069	0.799	2.88895
BGIBMGA005739	0.4853	0.8333	0.6231	3.03025
BGIBMGA005469	0.90375	1.1894	0.9555	2.38955
BGIBMGA001137	0.7686	0.8711	1.2503	5.96815
BGIBMGA010291	1.4322	1.4808	1.6351	2.2649
BGIBMGA007367	1.11365	1.2252	0.861	4.30325
BGIBMGA011887	0.90465	0.9005	0.9633	2.26015
BGIBMGA003377	1.0114	0.996	1.0742	2.0736
BGIBMGA004913	1.23895	1.3902	1.2917	5.7308
BGIBMGA006931	1.2198	1.3103	1.2858	4.269
BGIBMGA004994	1.02585	1.0999	1.1477	2.14525
BGIBMGA009495	0.7584	0.9024	0.877	3.1722
BGIBMGA001898	0.94095	0.9373	0.8535	2.2757
BGIBMGA005991	1.2557	1.1018	1.1516	4.0685

BGIBMGA007005	1.02945	0.9017	0.8968	2.6802
BGIBMGA006816	2.26595	2.0494	2.0041	1.17785
BGIBMGA005717	0.38705	0.6045	0.4684	0.43805
**Purine metabolism**				
gene code	3h	6h	12h	24h
BGIBMGA001333	2.2427	1.5925	1	1.6743
BGIBMGA002462	2.334	1.9853	0.9893	0.18635
BGIBMGA000807	1.31605	1.0797	1.3588	0.4656
BGIBMGA014227	1.33735	1.163	1.4362	0.30365
BGIBMGA001570	1.14915	1.3521	0.9511	0.2792
BGIBMGA000060	0.8216	0.878	0.8176	0.44615
BGIBMGA010637	0.8984	0.9238	0.8654	0.42945
BGIBMGA007835	1.0709	1.1118	1.1215	0.3145
BGIBMGA007935	2.4708	1.893	1.7835	0.52765
BGIBMGA010177	1.25275	1.133	1.1088	0.4207
BGIBMGA010377	1.17655	1.0223	0.9609	0.35835
BGIBMGA009962	1	1	1	0.27645
BGIBMGA002654	1.44375	1.1769	1.5024	2.24375
BGIBMGA001898	0.94095	0.9373	0.8535	2.2757
BGIBMGA001278	0.99985	1.1231	0.9577	3.6181
BGIBMGA004913	1.23895	1.3902	1.2917	5.7308
BGIBMGA006931	1.2198	1.3103	1.2858	4.269
BGIBMGA005991	1.2557	1.1018	1.1516	4.0685
BGIBMGA007005	1.02945	0.9017	0.8968	2.6802
BGIBMGA001068	1.0169	1.0259	1.1808	3.00565
BGIBMGA003377	1.0114	0.996	1.0742	2.0736
BGIBMGA006812	1.106	1.0812	1.1884	2.36345
BGIBMGA004994	1.02585	1.0999	1.1477	2.14525
BGIBMGA009495	0.7584	0.9024	0.877	3.1722
BGIBMGA010291	1.4322	1.4808	1.6351	2.2649
BGIBMGA012772	0.80015	0.8565	1.1992	3.69495
BGIBMGA005168	0.3294	0.5308	0.4348	1.03555
BGIBMGA005469	0.90375	1.1894	0.9555	2.38955

BGIBMGA005739	0.4853	0.8333	0.6231	3.03025	
BGIBMGA011753	0.67895	1.0069	0.799	2.88895	
BGIBMGA006292	0.9122	1.2202	1.0464	3.88025	
BGIBMGA010234	0.7818	0.9708	0.8741	2.19625	
BGIBMGA013054	0.74495	1.0787	1.0351	3.05985	
BGIBMGA005485	1.03645	1.1471	0.7493	2.4844	
BGIBMGA007367	1.11365	1.2252	0.861	4.30325	
BGIBMGA003330	1.19345	2.0366	1	2.8663	
BGIBMGA010715	0.1905	1	0.678	1.7076	
**RNA polymerase**					
gene	gene code	3h	6h	12h	24h
Pol II B4	BGIBMGA005469	0.9038	1.1894	0.95545	2.3896
Pol I A12	BGIBMGA006292	0.9122	1.2202	1.0464	3.8803
Pol III C11	BGIBMGA013054	0.745	1.0787	1.0351	3.0599
archaeal N	BGIBMGA005739	0.4853	0.8333	0.6231	3.0303
Pol III C25	BGIBMGA011753	0.679	1.0069	0.79895	2.889
Pol II B6	BGIBMGA010234	0.7818	0.9708	0.8741	2.1963
Pol III C5	BGIBMGA004913	1.239	1.3902	1.29165	5.7308
Pol II B3	BGIBMGA004994	1.0259	1.0999	1.1477	2.1453
Pol II B5	BGIBMGA001898	0.941	0.9373	0.8535	2.2757
**Basal transcription factors**					
gene	gene code	3h	6h	12h	24h
TFIID11	BGIBMGA007030	0.7214	0.9415	0.5915	3.6876
TFIID1	BGIBMGA011737	1	1	1	0.4438
TFIIA1	BGIBMGA012459	0.8821	0.9949	1.00635	3.736
TFIIE2	BGIBMGA010172	0.9099	1.0804	1.1478	2.1324
TFIID10	BGIBMGA005878	0.8264	0.9652	0.79255	2.5967
**Porphyrin and chlorophyll metabolism**					
gene code	3h	6h	12h	24h	
BGIBMGA004965	0.20365	0.6383	0.659	2.01865	
BGIBMGA009964	1.12705	1.1736	1.3214	5.8593	
BGIBMGA005500	0.52775	0.6429	0.6427	0.25505	
BGIBMGA013998	0.7642	0.9581	0.7933	2.8666	

BGIBMGA007912	0.933	0.9495	0.9173	0.47265
BGIBMGA003440	0.8657	0.843	0.8894	2.545
BGIBMGA000937	0.70975	0.8906	0.8685	2.3642
BGIBMGA002540	0.92545	1.0469	1.0954	3.6243
**Pantothenate and CoA biosynthesis**				
gene code	3h	6h	12h	24h
BGIBMGA002243	1.11865	1.1766	0.9491	2.89105
BGIBMGA001340	0.84125	0.963	0.8362	3.03355
BGIBMGA001237	0.93125	0.825	0.8514	0.40505
BGIBMGA005508	1.18395	0.9792	0.9365	0.45715
BGIBMGA005717	0.38705	0.6045	0.4684	0.43805
**One carbon pool by folate**				
gene code	3h	6h	12h	24h
BGIBMGA004950	2.18195	1.6869	1.4181	0.1773
BGIBMGA001137	0.7686	0.8711	1.2503	5.96815
BGIBMGA002462	2.334	1.9853	0.9893	0.18635
**2,4-Dichlorobenzoate degradation**				
gene code	3h	6h	12h	24h
BGIBMGA004229	1.48985	1.6544	2.3666	1.3332
BGIBMGA008633	0.37615	0.5679	0.4739	1.43765
BGIBMGA014599	1.6495	1.4539	1.6398	2.69265
BGIBMGA003842	0.75425	0.6444	0.7357	6.05845
**Benzoate degradation via hydroxylation**				
gene code	3h	6h	12h	24h
BGIBMGA014181	1.2032	1.1482	1.3362	0.4241
BGIBMGA003655	1.3887	1.5789	1.3436	0.42845
BGIBMGA001068	1.0169	1.0259	1.1808	3.00565
BGIBMGA003842	0.75425	0.6444	0.7357	6.05845
**Styrene degradation**				
gene code	3h	6h	12h	24h
BGIBMGA005064	1.22665	1.0558	0.9807	2.19385
BGIBMGA007424	0.93305	1.0064	1.4591	2.84965
BGIBMGA012866	1.54545	0.8363	0.338	3.50685

osynthesis and metabolism related gene					
gene	gene code	3 h	6 h	12 h	24 h
HMGS	BGIBMGA004001	0.974	1.1714	1.1154	2.8809
MevPK	BGIBMGA001556	0.7487	1.0425	0.73865	2.3791
SDR	BGIBMGA002886	1.0614	1.1467	0.78055	3.159
FAMeT1	BGIBMGA002604	0.137	0.6485	0.60905	1.1244
JHAMT2	BGIBMGA010392	0.0279	0.1834	0.0366	0.1161
JHAMT5	BGIBMGA010563	2.2638	1.4632	2.29005	3.4893
JHE1	BGIBMGA000772	0.2244	0.4665	0.7387	0.4605
JHEH5	BGIBMGA011468	1	1.7228	3.34165	1.4759
JHEH	BGIBMGA013929	0.824	2.0304	0.8701	0.6429
JHDK1	BGIBMGA008813	1.2371	1.0897	1.33775	4.0178
JHDK3	BGIBMGA008815	1.0526	1.1704	0.9006	3.1204
JHBP	BGIBMGA011457	1.9369	1.227	0.85835	2.7992
JHBP	BGIBMGA011458	2.3441	1.4374	1.63085	2.5535
FKBP39	BGIBMGA001490	0.8492	1.1858	1.81515	3.1414

**Trypsin**				
gene	3 h	6 h	12 h	24 h
BGIBMGA010063	4.36115	2.0319	3.1085	1
BGIBMGA012427	2.68015	3.387	2.5376	0.7349
BGIBMGA010061	2.1242	1.4266	1.4381	4.0534
BGIBMGA008668	2.05	1.4427	1.2232	0.56615
BGIBMGA010584	2.0209	1.4018	1.6249	2.37195
BGIBMGA009750	1.43475	1.3775	1.5314	2.4111
BGIBMGA008513	1.31615	1	0.9673	5.1106
BGIBMGA010276	1.25865	1.2643	1.3445	2.3877
BGIBMGA013698	1.17255	1.0619	1.2523	0.495
BGIBMGA010303	1.14895	1.0944	1.1355	2.73285
BGIBMGA003566	1.1065	1.4469	1.3035	2.09185
BGIBMGA008514	1.02765	1.024	1.0771	2.8975
BGIBMGA008101	1	1	0.3481	1
BGIBMGA010590	0.9947	1.8814	1.8615	2.0716
BGIBMGA008278	0.88665	0.8061	0.8839	2.2906

BGIBMGA007377	0.8036	0.5139	0.5572	2.239
BGIBMGA012478	0.7941	0.8599	1.0526	3.1573
BGIBMGA008280	0.6639	0.823	0.7796	2.5218
BGIBMGA006747	0.35795	0.6103	1	1.18485
BGIBMGA001320	0.2954	0.1465	0.7588	1.25435
**Serine proteases**				
gene	3 h	6 h	12 h	24 h
BGIBMGA001027	0.89605	1.2749	1.0914	4.3253
BGIBMGA012810	0.8472	1.0839	1.1496	2.26025
BGIBMGA008167	2.74815	1.8966	1.7882	1.1283
BGIBMGA012452	1.4982	0.8577	0.9194	2.61475
BGIBMGA003141	0.93965	1.0655	0.9204	2.9058
BGIBMGA012439	0.7479	0.3609	0.8569	0.4785
BGIBMGA006179	1.5174	1.1769	1.4902	0.4277
BGIBMGA001272	1.5142	1.5026	1.8306	0.34425
**Zinc carboxypeptidase**				
gene	3 h	6 h	12 h	24 h
BGIBMGA004800	2.03385	2.1502	1.678	2.52615
BGIBMGA004830	1.57225	1.4632	1.4773	2.5897
BGIBMGA003037	1.4468	1.0484	1.2142	0.34585
BGIBMGA009476	1.1448	1.2122	0.8474	2.16955
**Peritrophic membrane structural proteins**				
gene	3 h	6 h	12 h	24 h
BGIBMGA009641	2.31815	1.3921	1.4015	0.5581
BGIBMGA009809	1.26105	1.1657	1.2085	0.17975
BGIBMGA001504	1.12	1.1234	1.304	0.2385
BGIBMGA007250	1	1	1	0.14545
**Aminopeptidase N**				
gene	3 h	6 h	12 h	24 h
BGIBMGA010679	2.2333	1.5491	2.3216	0.52185
BGIBMGA008061	1.8968	1.4994	1.8734	0.49915
BGIBMGA008017	1.68155	1.3567	1.77	0.3552
BGIBMGA008062	1.3642	1.1306	1.2483	0.4371

BGIBMGA008060	1.31375	1.0396	1.4109	0.3731	
BGIBMGA008059	1.1553	1.046	1.2418	0.5262	
BGIBMGA008063	1	1	1	0.4978	
BGIBMGA008015	1	1	1	0.4521	
BGIBMGA008018	1	1.3075	1.6164	0.3698	
**Na/Ca exchage**					
BGIBMGA000688	6.962	1	1	1	
**ABC_tran/ABC2_membrane**					
BGIBMGA007218	1.5766	1.828	1.6091	0.42085	
BGIBMGA007769	1	1	1	0.49625	
BGIBMGA007792	1	1	1	0.43155	
BGIBMGA012743	1	1	1	0.42965	
BGIBMGA007784	1	1	1	0.4265	
BGIBMGA009503	1	1	1	0.39945	
BGIBMGA007738	1	1	1	0.32605	
BGIBMGA005473	0.9568	0.905	1.0617	0.46365	
BGIBMGA007869	0.84605	1.0689	1.2732	0.4225	
BGIBMGA007217	0.18045	0.5084	0.3665	1.43445	
BGIBMGA010761	1	1	1	0.2751	
BGIBMGA005226	0.2331	0.5125	0.201	0.35755	
**Nucleoplasmin protein**					
BGIBMGA009067	1.1001	1.3468	1.2778	2.2997	
**lysozyme**					
gene	gene	3 h	6 h	12 h	24 h
Lys	BGIBMGA012264	2.19	1.3607	1.3008	0.7457
BTL-LP1)	BGIBMGA012866	1.5455	0.8363	0.338	3.5069
**Lectin**					
gene	gene	3 h	6 h	12 h	24 h
ctl1	BGIBMGA002288	1.0308	1.0051	0.4899	2.1828
ctl2	BGIBMGA011634	1	1	1	2.315
ctl3	BGIBMGA006623	2.3068	1.2999	0.79105	1.1135
ctl4	BGIBMGA002289	1.0308	1.0051	0.4899	2.1828
**Scavenger receptor cysteine-rich**					

gene	gene	3 h	6 h	12 h	24 h
SRCR	BGIBMGA005199	0.956	1.0879	1	0.4089
SCRB10	BGIBMGA012263	2.4651	0.9876	0.54905	1.9676
SCRB12	BGIBMGA013439	0.316	0.6005	0.4431	0.2529
SCRC1	BGIBMGA004577	0.9036	1.0255	1.06665	0.2883
**SOD**					
gene	gene	3 h	6 h	12 h	24 h
SOD1	BGIBMGA005489	2.2391	1.8497	1.3703	4.8233
SOD2	BGIBMGA001307	0.7007	0.6014	0.70435	2.3935
**IgSF**					
gene	gene	3 h	6 h	12 h	24 h
dpr9	BGIBMGA014206	1.6095	1.3691	1.28195	0.3567
klg2	BGIBMGA008133	1	1	1	0.4892
titin8	BGIBMGA007388	1	1	1	0.404
imp-l2-1	BGIBMGA008146	0.9264	0.4459	0.4733	0.398
boi	BGIBMGA008552	0.5327	1	1	2.0241
dscam5	BGIBMGA013868	0.4973	0.7061	0.53775	0.8153
bent	BGIBMGA004546	2.0148	1.4162	1.34055	1.2466
titin1	BGIBMGA000623	1.733	0.9153	0.9173	0.3795
sls	BGIBMGA000622	1.7184	0.9222	1.50265	0.2909
ncam1	BGIBMGA010554	1	1	1	0.429
dscam2	BGIBMGA005747	1	1	1	0.4036
hemicentin2	BGIBMGA009441	1	1	1	0.3523
cg12484	BGIBMGA004752	1	1	1	0.2407
lrig1	BGIBMGA006920	0.7498	0.9278	0.69595	3.2443
lar1	BGIBMGA012048	0.7063	0.7912	0.70135	0.341
trol	BGIBMGA012240	1.2615	0.955	1.0983	0.4383
beat	BGIBMGA005237	0.3804	0.6462	1	1
dpr4	BGIBMGA007216	0.2359	0.8122	0.48385	1.1187
**Peptidoglycan recognition protein**					
gene	gene	3 hr	6 hr	12 hr	24 hr
PGRP-S2	BGIBMGA007987	1.1756	1.1593	1.0606	4.8857
PGRP-S5	BGIBMGA012866	1.5455	0.8363	0.338	3.5069
**TEP**					

TEP3	BGIBMGA013565	1	1	1	0.419
**CLIP**					
gene	clip	3 h	6 h	12 h	24 h
clip1	BGIBMGA008668	2.05	1.4427	1.22315	0.5662
clip2	BGIBMGA014603	0.7915	0.8951	0.7901	0.3025
**SPN**					
gene	gene	3 h	6 h	12 h	24 h
spn1	BGIBMGA010213	2.0792	1.2544	1.11155	1.2121
spn2	BGIBMGA004726	1	1.4742	0.47005	0.7338
spn3	BGIBMGA008831	0.2487	0.33	0.2859	0.4831
**PPO**					
gene	gene	3 h	6 h	12 h	24 h
ppo1	BGIBMGA012764	2.1064	1.629	2.05445	0.4012
ppo-2s	BGIBMGA013115	1.8705	2.3227	2.10675	0.7435
ppo-1s	BGIBMGA012763	1.4839	1.9113	2.0136	0.2737
**AMPs**					
gene	gene	3 hr	6 hr	12 hr	24 hr
attacin	BGIBMGA002739	0.568	0.4119	0.5943	2.0537
enbocin	BGIBMGA000039	0.7354	0.1754	0.29065	3.3526
gloverin	BGIBMGA013865	1.0945	0.5922	0.5785	3.4076
lebocin	BGIBMGA006775	1.0683	0.9359	0.95405	2.0759
moricin	BGIBMGA011495	2.1127	1.5315	0.66685	8.1517
