# Supplementary material for: A Genome-Wide Survey for Host Response of Silkworm, Bombyx mori during Pathogen Bacillus bombyseptieus Infection
Source: PLoS One. 2009 Dec 1;4(12):e8098. doi: 10.1371/journal.pone.0008098 (PMC2780328; doi:10.1371/journal.pone.0008098)
Supplement: Table S5 — Primers used for cloning the 16S rRNA gene clone, real time PCR analysis. (0.01 MB PDF) [file pone.0008098.s005.pdf]

Table S5 Primers used for cloning the 16S rRNA gene clone, real time PCR and RT-PCR analysis.	
**The primers used for 16S rRNA gene clone**	
**16S rRNA**	
sense primer	5' AGAGTTTGATCCTGGCTCAG 3'
antise primer	5' AAGGAGGTGATCCAGCCGCA 3'
**The primers used for real time PCR analysis to validate the microarray data**	
**Sw22934**	
sense primer	5' TTCGTACTGGCTCTTCTCGT 3'
antise primer	5' CAAAGTTGATAGCAATTCCCT 3'
**PPO-1**	
sense primer	5' ATGCCTCTGGACGAAG 3'
antise primer	5' CAGGTCTCCCATCACG 3'
**PPO-2**	
sense primer	5‘ TTCCAGAGGCTTACTTCCC 3‘
antise primer	5‘ GTTTCTTCTCCAGCGTTCC 3‘
**CecD1**	
sense primer	5’ TCAGGATCGGCTCCGTCAG 3’
antise primer	5’ GCGGGAAGTGCCTCTGGAA 3’
**A012864**	
sense primer	5‘ CCAGTGCCCATAGTAGTGATTC 3’
antise primer	5‘ CGGATGTGACCGAGCCT 3’
**Attacin2**	
sense primer	5' GTTCAAACAGAAGGTGGGC 3'
antise primer	5' GCGAGCTGGACGGAGAT 3'
**Caspase3**	
sense primer	5' TGCCGACCAACCATAC 3
antise primer	5' CACCATCCAGCACCAC 3'
**Dpr**	
sense primer	5' TCTACACGGGACCCTACT 3'
antise primer	5' GACACCGACTTGTTGCTC 3'
**The primers used for real time PCR analysis involved in immune pathways**	
**Spz1**	
sense primer	5‘ AGGATTCGCCTCACAGTCAC 3‘
antise primer	5‘ ATTTTCAGTTCGGGATGCTT 3‘
**Toll1**	
sense primer	5‘ CTGCTTCACTTCGGCTGGA 3‘
antise primer	5‘ CGCTCTCAAGGGTTCGGAT 3‘
**Toll6**	
sense primer	5‘ TTGGAAGTATTGCGATTGG 3‘
antise primer	5‘ CACGGCGTTCGTTACATTG 3‘
**Myd88**	
sense primer	5' TAATAGACTCGGAGGAATG 3'
antise primer	5' CGATACTAATAGCCTGTGC 3'

**Tube**	
sense primer	5' AACGCTGAAGTCAAATC 3'
antise primer	5' TTAGTCCCTCTGCTGTC 3'
**RelA**	
sense primer	5‘ CCAGCAAAGCCCTCAGA 3‘
antise primer	5‘ GTTGTGCGGGTGCGGTT 3‘
**Domeless**	
sense primer	5‘ TTACACTGGCATTGAACACC 3‘
antise primer	5‘ TGAATCACTAAGCACATCGG 3‘
**Hop**	
sense primer	5‘ TGTGGCAAAACGGCAGTGAGA 3‘
antise primer	5‘ CAGGATGGTGCGGATGAAAAG 3‘
**Stat1**	
sense primer	5‘ TGAGGCATTGTTTGGCGT 3‘
antise primer	5‘ CTCCTGGGGGGCGTGACT 3‘
