# Supplementary material for: A Genome-Wide Survey for Host Response of Silkworm, Bombyx mori during Pathogen Bacillus bombyseptieus Infection
Source: PLoS One. 2009 Dec 1;4(12):e8098. doi: 10.1371/journal.pone.0008098 (PMC2780328; doi:10.1371/journal.pone.0008098)
Supplement: Table S6 — The microarray data and Real-Time PCR data of innate immune signaling genes. (0.01 MB PDF) [file pone.0008098.s006.pdf]

Table S6

The microarray data and Real-Time PCR data of innate immune signaling genes

gene	gene number	probe	Microarray data (Averaged sample signal values/Averaged control signal values)				Real-time PCR ratios	
			3 h	6 h	12 h	24 h	6 h	12 h
**Toll pathway**								
Spz1	BGIBMGA002397	sw08256	383/278	283/251	293/260	146/324	6.12	5.21
Toll1	BGIBMGA011037	sw08028	99/98	300/74	86/93	111/102	3.54	3.8
Toll6	BGIBMGA011084	sw12719	78/-17	74/13	64/51	45/129	3.27	3.02
Myd88	BGIBMGA002869	sw05796	305/282	393/250	269/206	250/375	5.22	4.81
Tube	BGIBMGA002494	sw20628	321/266	377/239	343/274	342/414	6.01	4.82
RelA	BGIBMGA010496	sw17578	263/174	445/170	558/104	911/494	6.21	9.13
**JAK/STAT1 pathway**								
Hop	BGIBMGA004082	sw00739	86/52	76/122	154/77	49/180	0.48	1.44
Dome	BGIBMGA005642	sw14077	110/192	142/177	335/234	175/318	0.12	1.87
Stat1	BGIBMGA001739	sw15699	56/91	112/115	120/54	44/277	0.23	1.69
